# Patterns of Intron Gain and Loss in Fungi

**DOI:** 10.1371/journal.pbio.0020422

**Published:** 2004-11-30

**Authors:** Cydney B Nielsen, Brad Friedman, Bruce Birren, Christopher B Burge, James E Galagan

**Affiliations:** **1**Department of Biology, Massachusetts Institute of TechnologyCambridge, MassachusettsUnited States of America; **2**The Broad Institute of Massachusetts Institute of Technology and Harvard UniversityCambridge, MassachusettsUnited States of America; **3**Department of Mathematics, Massachusetts Institute of TechnologyCambridge, MassachusettsUnited States of America

## Abstract

Little is known about the patterns of intron gain and loss or the relative contributions of these two processes to gene evolution. To investigate the dynamics of intron evolution, we analyzed orthologous genes from four filamentous fungal genomes and determined the pattern of intron conservation. We developed a probabilistic model to estimate the most likely rates of intron gain and loss giving rise to these observed conservation patterns. Our data reveal the surprising importance of intron gain. Between about 150 and 250 gains and between 150 and 350 losses were inferred in each lineage. We discuss one gene in particular (encoding 1-phosphoribosyl-5-pyrophosphate synthetase) that displays an unusually high rate of intron gain in multiple lineages. It has been recognized that introns are biased towards the 5′ ends of genes in intron-poor genomes but are evenly distributed in intron-rich genomes. Current models attribute this bias to 3′ intron loss through a poly-adenosine-primed reverse transcription mechanism. Contrary to standard models, we find no increased frequency of intron loss toward the 3′ ends of genes. Thus, recent intron dynamics do not support a model whereby 5′ intron positional bias is generated solely by 3′-biased intron loss.

## Abstract 2

A comparative study of four fungal genomes reveals the patterns of intron gain and loss over several hundred million years of evolution

## Introduction

Over a quarter of a century after the discovery of introns, fundamental questions about their function and evolutionary origins remain unanswered. Although intron density differs radically between organisms, the mechanisms by which introns are inserted and deleted from gene loci are not well understood. A correlation has been observed between intron density and positional bias (Mourier and Jeffares 2003). Introns are evenly distributed within the coding sequence of genes in intron-rich organisms, but are biased toward the 5′ ends of genes in intron-poor organisms. This bias is particularly pronounced in the yeast Saccharomyces cerevisiae. It has been suggested that both the paucity and positional bias of introns in yeast may be due to intron loss through a mechanism of homologous recombination of spliced messages reverse-transcribed from the 3′ poly-adenylated tail (Fink 1987). This reverse transcription mechanism was first demonstrated in experiments with intron-containing Ty elements in yeast (Boeke et al. 1985). More recently, Mourier and Jeffares (2003) concluded that homologous recombination of cDNAs is the simplest explanation for the positional bias observed in all intron-poor eukaryotes. However, few data exist concerning the actual mechanisms and dynamics of intron evolution.

Fungal genomes are in many ways ideal for exploring questions of intron evolution. The fundamental aspects of intron biology are shared between fungi and other eukaryotes, making fungi appropriate model organisms for intron study. They are gene dense with relatively simple gene structures compared with plants and animals, making gene prediction more accurate. Fungi also display a wide diversity of gene structures, ranging from far less than one intron per gene for *S. cerevisiae,* to approximately 1–2 introns per gene on average for many recently sequenced ascomycetes (including the organisms in this study), to roughly seven introns per gene on average for some basidiomycetes (e.g., *Cryptococcus*). Finally, fungi display a strong 5′ bias in intron positions, enabling us to investigate the processes underlying this phenomenon.

In principle, a 5′ intron bias could arise through various combinations of intron gain and loss, and a complete understanding of intron positional bias requires an assessment of the contributions of both of these processes. A number of studies demonstrate the occurrence of intron gain and loss in individual genes or gene families. Logsdon et al. (1995) offered early examples of well-supported intron gain by comparing triose-phosphate isomerase genes from diverse eukaryotes and demonstrated that numerous introns could be most parsimoniously explained by a single gain with no subsequent losses. O'Neill et al. (1998) later provided evidence for de novo intron insertion into the otherwise intron-less mammalian sex-determining gene *SRY*. Evidence for the occurrence of multiple independent intron losses has also been reported in studies such as those by Robertson (2000), who inferred gain and loss events in a family of chemoreceptors in Caenorhabditis elegans.

More recently, a number of genome-wide studies of intron dynamics have been conducted. Roy et al. (2003) described genome-wide comparisons between human and mouse (with *Fugu* as an outgroup) and between mouse and rat (with human as an outgroup), and observed a sparseness of intron loss and complete absence of intron gain in these closely related organisms. On the other hand, Rogozin et al. (2003) observed an abundance of lineage-specific intron loss and gain when analyzing clusters of orthologous genes in deeply branching eukaryotes. Similarly, Qiu et al. (2004) analyzed ten protein families in distantly related eukaryotes, with a single prokaryotic outgroup, and obtained evidence that extant introns are predominantly the result of intron gains. In search of clues to understand the mechanism of intron gain, Fedorov et al. (2003) aligned introns from various eukaryotes, and Coghlan and Wolfe (2004) applied a similar approach in a comparative study of nematodes. None of these studies addressed the positional bias of intron gain and loss events. Here we report the results of a genome-wide comparative analysis of intron evolution in organisms that have a strong 5′ bias in intron location and are at an appropriate evolutionary distance to reveal positional trends in intron gain and loss.

## Results

**Figure 1:**
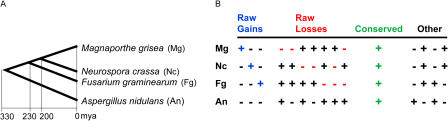
Phylogenetic Tree and Intron Conservation Patterns (A) Phylogenetic tree of the four fungal organisms studied *(M. grisea, N. crassa, F. graminearum,* and *A. nidulans)* with estimated time scale in millions of years ago. The rooted organismal tree was constructed using an unweighted pair group method using arithmetic averages based on a concatenated alignment of 2,073 orthologous gene sets. Estimated dates of divergence from Taylor et al. (1999), Berbee and Taylor (2000), and Heckman et al. (2001).(B) Classification of intron presence (+) and absence (−) patterns across the four fungal species. A blue “+” indicates a raw intron gain in the corresponding organism, a red “−” indicates a raw intron loss in the corresponding organism, a green “+” indicates a conserved intron, and all other introns are indicated in black.

**Figure 2:**
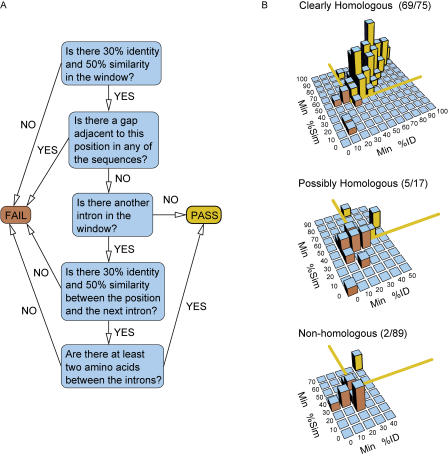
Alignment Filtering Protocol (A) Schematic of filtering protocol applied to a ten-residue window on each side of every intron position. If either side failed the filter, the position was discarded. (B) Distributions of minimum percent identity and similarity in ten-residue windows around 181 randomly selected intron positions, for three manual classifications. The minima were taken between the left and right windows. The yellow lines indicate the chosen thresholds of at least 50% similarity and 30% identity, and bars are colored yellow if they fall above the thresholds (pass) or orange if they fall below the thresholds (fail). Parentheses indicate the number of introns in each class that pass the cutoff and the total number of introns in that class. The five lowest-percent identity and similarity bars, containing 77 positions, in the “non-homologous” plot are omitted so as to not obscure the rest of the histogram.

To investigate the roles of both gain and loss in intron evolution, we compared the genomes of four recently sequenced fungi spanning at least 330 million years of evolution (Taylor et al. 1999; Berbee and Taylor 2000; Heckman et al. 2001) (Figure 1): Aspergillus nidulans, Fusarium graminearum, Magnaporthe grisea, and Neurospora crassa. Ortholog sets composed of one gene from each of the four genomes were identified as pairwise best bidirectional BLAST hits satisfying stringent overlap criteria. Orthologs in each set were subsequently aligned, and the locations of introns were marked. These intron positions (regions of the multiple sequence alignment containing an intron in at least one of the four sequences) were subjected to rigorous alignment quality filtering to eliminate alignment and annotation errors (Figure 2A). To set the filtering thresholds, we manually classified ten residue alignment windows on either side of 181 randomly selected intron positions as “clearly homologous,” “possibly homologous,” or “non-homologous.” Requiring 30% identity and 50% similarity in these windows captured 92% of the clearly homologous positions, 29% of the possibly homologous positions, and only 2% of the non-homologous positions (Figure 2B). Passing intron positions were split into five quintiles according to their relative position within the annotated coding sequence.

### Genome-Wide Characterization of Intron Conservation

**Figure 3:**
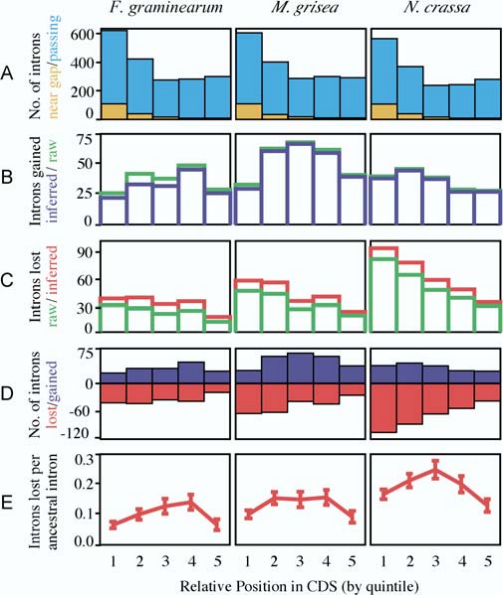
Positional Biases in Intron Gain and Loss Relative intron positions were defined as the number of bases in the coding sequence upstream of the intron divided by the total length of the coding sequence. These relative positions were binned into five categories (quintiles), each representing one-fifth of the coding sequence length (quintiles numbered 1–5 on the x-axis). (A) Introns passing quality filter (light blue, back) and introns adjacent to gaps in the protein alignment that were removed by our quality filter (orange, front). (B) Raw and inferred gains. Raw gains (green, back) are those introns present in exactly one organism (excluding the outgroup, A. nidulans). Inferred gains (blue, front) are corrected for the estimated number of cases that arose by other combinations of gain and loss events. Inferred gains are thus slightly lower than raw gains. (C) Raw and inferred losses. Raw losses (green, front) are those introns absent in the organism in question but present in at least one of its siblings (descendants of its parent in the phylogenetic tree) and one of its cousins (non-descendants of its parent). Inferred losses (red, back) are corrected for the estimated number of introns lost along multiple lineages, or gained and then lost. Inferred losses are thus slightly higher than raw losses. (D) Number of introns gained (blue) and lost (red) since last common ancestor (losses shown as negative numbers). (E) Intron loss rate at each position since last common ancestor (introns lost per ancestral intron). Error bars represent binomial standard deviation.

We applied our analysis protocol to 2,073 putative ortholog sets that included 9,352 intron positions. Of these initial intron positions, 5,811 were removed because of low conservation surrounding the intron, or because of an adjacent gap, or both. It is possible that some of the positions neighboring gaps may in fact reflect intron gain or loss events that occurred simultaneously with coding sequence insertion or deletion (Llopart et al. 2002). However, removing these positions did not significantly impact our results, as the number of positions adjacent to gaps was only about one-tenth of the number of positions that passed the quality filter, and the removal of these introns did not alter the apparent positional bias of the overall distribution (Figure 3). An additional 92 introns had nearby introns with insufficient conservation between the two introns and were thus also rejected.

In the end, a total of 3,450 intron positions (roughly 37% of intron positions considered) passed the quality filter. The complete set of aligned orthologs with passing and failing intron positions is provided in Table S1. These data constitute a genome-wide survey of high-confidence aligned intron positions and their patterns of conservation over at least 330 million years of evolution.

**Figure 4:**
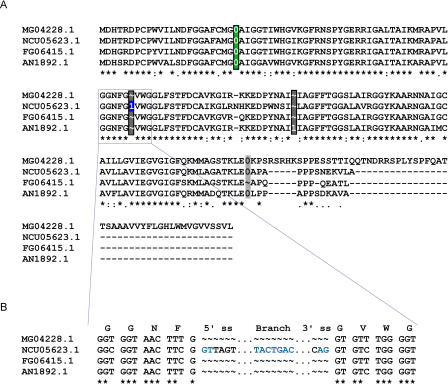
Example Ortholog Alignment (A) Alignment of protein sequences for orthologs MG04228, NCU05623, FG06415, and AN1892 with intron characters inserted. “0,” “1,” and “2” indicate the phase of an intron. A black-edged rectangle indicates an intron position passing our quality filters; an unedged gray rectangle indicates an intron position that was removed by our filter. The green rectangle indicates conserved introns, the blue box marks a raw intron gain, and the gray boxes within black-edged rectangles highlight all other introns. The consensus (bottom) line characters are as follows: asterisk, identical residue in all four sequences; colon, similar residue; and period, neutral residue. (B) Nucleotide alignment of the region flanking the gained intron in (A). Putative 5′ and 3′ splice sites and a branch point sequence are highlighted in blue.

An example of an alignment of putative orthologs with three passing intron positions is shown in Figure 4A. In each passing intron position (black-edged rectangles), individual introns are labeled according to the classes previously outlined in Figure 1B. One intron position is conserved across all four species (green rectangle), one is a raw gain in N. crassa (blue box), and the third is present only in *A. nidulans,* and, because of the ambiguity in inferring gain or loss in this case, is classified as “Other” (black-edged gray rectangle). Examining the region around the one raw gained intron in N. crassa at the nucleotide level (Figure 4B) reveals a clean insertion of the intron sequence within a highly conserved region. The gained intron has consensus terminal dinucleotides (GT…AG) and a putative branch point sequence that matches the yeast consensus ( TACTAAC) at six of seven positions. In addition, this set of orthologs contained one poorly aligned intron position (Figure 4A, unedged gray rectangle) that was excluded by our filters. All three passing positions (black-edged rectangles) display high amino acid sequence conservation on both sides flanking the intron, supporting the correctness of the alignment. In contrast, the failing intron position (unedged gray rectangle) is adjacent to a region of the alignment that lacks significant conservation. The 3′ flank of this intron position displays considerable variation, especially with respect to the M. grisea gene, which was predicted to have a much longer 3′ coding region. In such an alignment region, it is difficult to distinguish true differences in intron conservation from potential annotation or alignment errors. Our filtering process thus eliminated this position from further analysis.

### Calculation of Raw Gains and Losses

We calculated “raw gains” and “raw losses” by positional quintile for each organism other than the outgroup, A. nidulans. We defined raw gains as those introns present in only a single organism (see Figure 1B). We defined raw losses as those introns that are absent in the organism in question, present in some other descendant of the organism's parent (a “sibling”), and present in some non-descendant of the parent (a “cousin”) (Figure 1B). Intron positions are considered conserved if present across all four organisms. Patterns of intron presence and absence that are not captured by the above definitions were excluded from the raw counts because of the ambiguity in inferring intron gain or loss events in such cases (marked as “Other” in Figure 1B).

### Probabilistic Model of Intron Gain and Loss

Raw gain and loss counts are based on parsimony and may differ somewhat from the true number of gain and loss events. The set of raw gains may include introns that were lost in multiple lineages, thus overcounting the true number of gains in a given lineage. Similarly, the set of raw losses excludes introns lost in the given organism and also lost in all cousins or siblings (marked as “Other” in Figure 1B).

We used a probabilistic model to correct for these inaccuracies. Our model assumes that all loss and gain events occur independently and uniformly within each quintile. In particular, we assume Dollo's postulate (Dollo 1893): any introns that align to the same position must have a common ancestor (no “double gains”), as in Nei and Kumar (2000) and Rogozin et al. (2003). Our method differs from the Dollo parsimony method described in Farris (1977) and applied in Rogozin et al. (2003) in that we do not artificially minimize loss events by assuming that gains occurred at the latest possible point in evolution. It also differs in that we allow different branches of the phylogenetic tree to have different rates of loss and gain. We applied our method separately to each of the five positional quintiles for each organism other than the outgroup, A. nidulans.

First we estimate two types of intron loss rates. The organismal loss rate, *q,* is calculated by dividing the number of raw losses in an organism by the total number of introns present in at least one sibling and at least one cousin. This represents the fraction of introns in the parent that did not survive to the present day in that organism. For instance, the organismal loss rate in F. graminearum is given by


\eqalignno*q* = (*AM* + *AN* + *AMN*) / ((*AM* + *AN* + *AMN*) + \cr(*AFM* + *AFN* + *AFMN*)) (1)

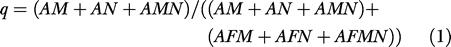



where *AM,* for example, represents the number of intron positions with an intron present in A. nidulans (A) and M. grisea (M) but absent from F. graminearum (F) and N. crassa (N).

The sibling loss rate, *r* is defined for a given organism as the fraction of introns in the parent that did not survive in any sibling. We define “sibling raw losses” for an organism as the number of introns that are present in the organism and at least one cousin but in no sibling. This quantity is then divided by the number of introns present in that organism and at least one cousin to give the sibling loss rate. For example, the sibling loss rate for F. graminearum is given by


*r* = (*AF*) / (*AF* + *AFM* + *AFN* + *AFMN*).

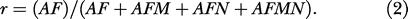



We next correct the raw gains for each organism. Raw gains include some introns that were in fact lost in all but one lineage. We use the loss rates to calculate the expected number of these multiple losses, *m,* and subtract this quantity from the raw gains to obtain “inferred gains.” To calculate *m* we first count *B*_0_, the number of introns conserved in the organism and at least one sibling, but in no cousin. The quantities *m* and *B*_0_ are related through the variable*n*_0_, the number of introns present in an organism's parent but not in any cousin, by the equations


*m* = *n*_0_*r*(1 − *q*)

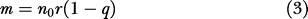



and


*B*_0_ = *n*_0_(1 − *r*)(1 − *q*).

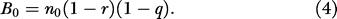



This follows from our assumption of independent gains and losses. Thus, we can calculate the expected number of multiple losses as


*m* = *B*_0_*r* / (1 − *r*).

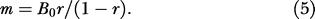



We use the loss rates to estimate the number of introns in each organism's parent. To do so, we estimate separately the number of parental introns present in at least one cousin *n*_1_, and the number not present in any cousin *n*_0_ (introduced above). To estimate the size of the set of parental introns present in at least one cousin, we first count the subset of these introns that are presently observable. An intron is in this set if it is present in at least one cousin and at least one sibling, or is present in at least one cousin and in the organism in question. We call this number of introns *B*_1_. By the assumption that gains and losses are independent, we have


*B*_1_ = *n*_1_(1 − *qr*).

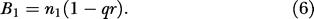



Using this relation and the one in equation 4 above, we calculate the number of introns in the phylogenetic parent as



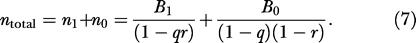



Finally, we correct raw losses. Our definition of raw losses undercounts the true number by omitting those introns not conserved in at least one cousin and at least one sibling. Taking F. graminearum as an example, the true number of losses would also include some introns conserved in the patterns *A, M, N,* and *MN*. We calculate the number of inferred losses as *n*_total_ *q*.

This method can be extended to any phylogenetic tree and to any organism with at least one cousin.

### Abundance of Intron Gains

One immediate conclusion stemming from our analysis is the importance of intron gain. A summary of all raw and inferred gains and losses is shown in Figure 3. Substantial numbers of gained introns were observed in all three organisms—more than 100 independent inferred gains in each lineage, with over 200 in M. grisea (Figure 3B). The total numbers of gains that have occurred in each genome are likely to be substantially higher, since only predicted orthologs in all four species were considered, and roughly a third of the introns in these genes passed our quality filters. Differences in intron dynamics between lineages are also apparent, with the numbers of gained and lost introns approximately balanced in M. grisea and *F. graminearum,* but with roughly twice as many losses as gains in N. crassa (Figure 3D). It is thus apparent from these data that the process of intron gain plays a significant role in intron evolution.

### 1-Phosphoribosyl-5-Pyrophosphate Synthetase Genes Display Lineage-Specific Increases in Intron Gain Rate

**Figure 5:**
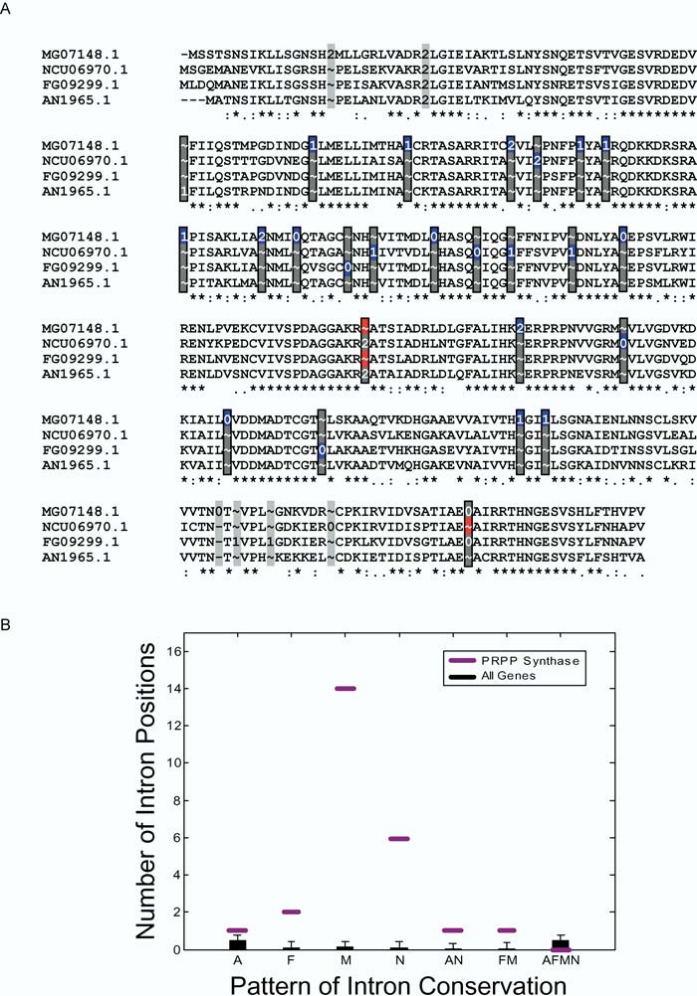
Intron Conservation in the PRPP Synthetase Gene (A) Alignment of PRPP synthetase putative orthologs MG07148, NCU06970, FG09299, and AN1965. A black-edged rectangle indicates an intron position passing our quality filters, whereas an unedged gray rectangle indicates an intron position that was removed by our filter. Blue boxes mark raw intron gains, red boxes indicate raw intron losses, and gray boxes within black-edged rectangles highlight all other introns. We manually corrected an annotation error in the first intron of the last row of the alignment. (B) Phylogenetic conservation pattern of introns in the PRPP sythetase gene. Each passing intron position was categorized as being present in A. nidulans (A), F. graminearum (F), M. grisea (M), N. crassa (N), A. nidulans and N. crassa (AN), F. graminearum and M. grisea (FM), or all four organisms (AFMN). There are no passing cases of conservation in three or four species. The number of introns in each category is shown with a purple line. The black error bar plot shows the mean and standard deviation for each category for all 2,008 ortholog sets after fitting to a Poisson distribution (see Materials and Methods). The number of introns in M. grisea and N. crassa is significantly higher, at the *p* < 1 × 10^−9^ level.

A striking example of intron gain occurs in a set of putative orthologous 1-phosphoribosyl-5-pyrophosphate (PRPP) synthetase genes. These genes encode a widely conserved protein that catalyzes the production of PRPP, a precursor in the nucleotide biosynthesis pathway. In contrast to the majority of orthologs that displayed fewer than two gained introns, the set of PRPP synthetase genes displayed a total of 22 raw gains (Figure 5A, blue boxes) that passed our alignment quality filters: six in *N. crassa,* 14 in *M. grisea,* and two in *F. graminearum.* The number of raw gains in the PRPP synthetase genes in M. grisea and N. crassa was significantly higher (*p* < 3 × 10^−22^ and *p* < 4 × 10^−9^, respectively) than the average for other genes analyzed, resulting in unusually large numbers of introns in these genes (Figure 5B). In comparison, the numbers of introns in PRPP synthetase genes in available animal genomes were within the typical range for the respective organisms, e.g., five in *C. elegans,* and six in fruitfly, human, mouse, rat, and Fugu. Thus the rate of intron gain for the PRPP synthetase gene in some fungi is unusually high. This gene represents an extreme example of the impact of intron gain and illustrates the variability of gain rates in different lineages.

### Fungal Introns Display Phase Bias, but Lack Observable Sequence Preference

For each in-group lineage *(M. grisea,* *N. crassa, F. graminearum),* we determined the frequency of phase 0, 1, and 2 introns in the set of all intron positions (Table 1). In contrast to recent reports based on a much smaller sample size indicating that phase frequencies for extant fungal introns do not differ significantly from a uniform distribution (Qiu et al. 2004), our genome-wide dataset demonstrates a clear bias for phase 0 introns in each of the three fungal in-group lineages examined (*p* < 4 × 10^−9^ for N. crassa and *p* < 1 × 10^−12^ for M. grisea and *F. graminearum;* in Table 1, “all passing,” and similar biases were seen in the unfiltered set). The phase distributions of raw gains and raw losses for each of the three organisms are not significantly different from a uniform distribution at *p* < 0.01; however, the datasets for these subclasses were much smaller (Table 1). Finally, we examined the exon sequences flanking gained introns, and observed no clear sequence bias (Table 2).

**Table 1:**
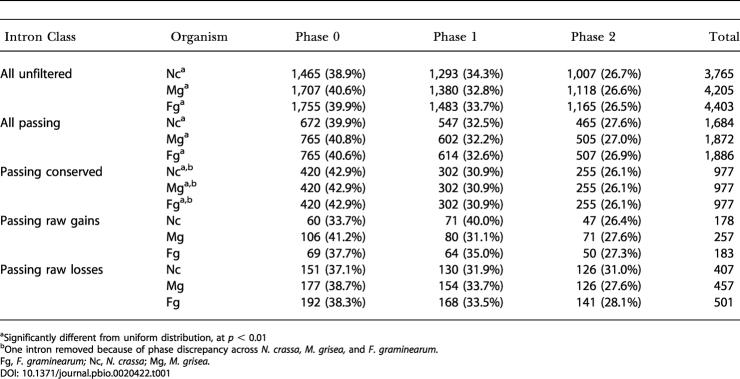
Intron Phase Distribution for Filtering and Conservation Classes

^a^Significantly different from uniform distribution, at *p* < 0.01

^b^One intron removed because of phase discrepancy across *N. crassa, M. grisea,* and *F. graminearum.*

Fg, *F. graminearum;* Nc, *N. crassa*; Mg, *M. grisea.*

**Table 2:**
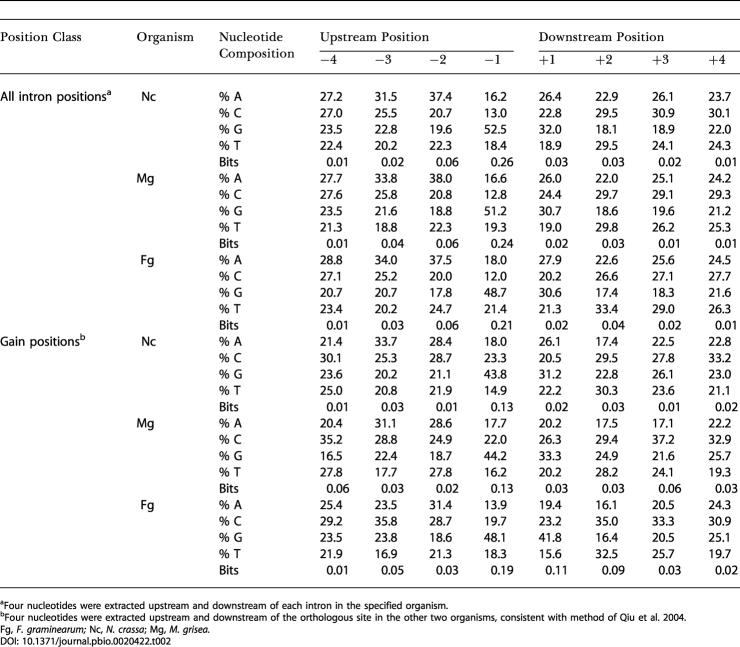
Exonic Nucleotide Composition near Introns

^a^Four nucleotides were extracted upstream and downstream of each intron in the specified organism

^b^Four nucleotides were extracted upstream and downstream of the orthologous site in the other two organisms, consistent with method of Qiu et al. 2004

Fg, *F. graminearum;* Nc, *N. crassa*; Mg, *M. grisea.*

### Absence of 3′ Bias in Intron Losses

To determine whether the pattern of intron loss in these fungi might account for the observed bias in intron position, we examined the pattern of loss as a function of position within the gene (see Figure 3E). Contrary to what would be expected if intron loss primarily involved homologous recombination of poly-adenosine-primed reverse transcripts, the rate of intron loss tends to be lower, rather than higher, at the 3′ ends of genes. Moreover, the highest rates of intron loss occur in the middles of genes in all three organisms. We found no evidence that this pattern was affected by our filtering methods. These findings suggest either other mutational mechanisms (e.g., reverse transcription primed internally) or the presence of selective pressure to preferentially conserve introns near the 5′ and 3′ ends of genes.

## Discussion

We developed a system that automatically identifies evolutionary and positional patterns of intron conservation on a genome-wide scale. The core of the system is a process for stringently filtering alignments of orthologous genes to exclude potential annotation or alignment errors. The result of the filtering process is a high-confidence set of aligned intron positions. Differences in intron conservation at each individual position can be characterized as gains or losses (or ambiguous) based on parsimony. However, this does not accurately account for the possibility of multiple gain or loss events. We have developed a probabilistic model that allows for multiple events, providing a corrected estimate of the total number of gains and losses within the dataset. Our probabilistic method allows for a more accurate assessment of rates of gain and loss. In our dataset, allowing for multiple events results in only modest corrections to the rates estimated using parsimony.

Our analysis demonstrates a significant role for intron gain over the past few hundred million years in the fungi analyzed. Previous analyses of specific gene families have provided evidence of specific instances of gained introns (Logsdon et al. 1998; Robertson 2000; Hartung et al. 2002; Qiu et al. 2004). However, the relative importance of intron gain versus loss is not well understood. Recent large-scale analyses have suggested that intron gain may play a predominant role in shaping gene structures (Qiu et al. 2004), although lineage-specific differences are apparent (Rogozin et al. 2003). In particular, intron gain appears to occur rarely if at all in mammalian genes (Roy et al. 2003). Our data suggest that intron gain is a significant driving force in the evolution of genes in fungi. In F. graminearum and M. grisea the number of introns gained was on par with the number lost and similar in magnitude to the number of introns gained in N. crassa.

The mechanisms underlying intron gain are not known. We analyzed the set of predicted intron gains for possible signatures that might shed light on this process. No statistically significant bias was detected in the positions of gained introns along the coding sequence (see Figure 3 data not shown). Similarly, no preferred insertion site sequence was detectable (Table 2), and no significant phase bias for gained introns was observed (see Table 1). The lack of an insertion site preference and absence of significant phase bias for gained introns in fungi is consistent with previous investigations and may set fungi apart from other organisms (Qiu et al. 2004).

Our data further indicate that intron gain can vary substantially between different gene families in a lineage-specific fashion. The PRPP synthetase gene is a particularly striking example, exhibiting significant increases in gained introns in two of the four lineages investigated. Moreover, the paucity of intron positions shared between N. crassa and M. grisea suggests the possibility of independent increases in gain rate in the two species. Alternatively, the apparent high intron gain rate exhibited by this gene may have arisen just prior to the last common ancestor of N. crassa and M. grisea. Although it is premature to speculate about possible mechanisms, one possibility is that a factor or factors responsible for intron insertion evolved to associate with the PRPP synthetase gene locus, transcript, or message at this point, leading to a higher rate of intron insertion in this gene.

Finally, our results do not support the mechanism commonly proposed to account for the 5′ positional bias of introns in intron-poor organisms (Mourier and Jeffares 2003). Contrary to what would be expected if intron loss primarily involved recombination of poly-adenosine-primed reverse transcripts, the rate of intron loss tends to be lower at the 3′ ends of genes. Instead, the highest rates of intron loss occur in the middles of genes in all three organisms. (This result is consistent with the results of Roy et al. (2003) in their analysis of intron evolution in mammals. Although their report describes only six instances of loss, in each case it was an internal intron.) The preference for internal introns may reflect a process of reverse transcription primed internally. Alternatively, there may be pressure to preferentially conserve introns near the 5′ and 3′ ends of genes. In particular, there is strong evidence for a functional role for the 5′-most intron in many genes. What remains clear is that the pattern of loss in these fungi over the last 330 million years cannot be explained solely by a mechanism involving 3′-end-primed reverse transcription of spliced messages. Instead, fungal intron dynamics appear to reflect a more complex interplay between intron gain and loss, an interplay that is likely to shape intron evolution in other eukaryotes.

## Materials and Methods

### 

#### Sequences and annotations

All sequences and annotations were taken from the Broad Institute Fungal Genome Initiative website (http://www.broad.mit.edu/annotation/fungi/fgi). The following datasets were used: A. nidulans (Assembly 1, 18 February 2003), *N.* crassa (Assembly 3, 1 February 2001), *F. graminearum* (Assembly 1, 11 March 2003), and M. grisea (Assembly 2, 18 July 2002).

#### Ortholog identification

A group of four proteins, one from each organism, was considered an ortholog set if each pair was a pairwise best bidirectional BLAST hit in the respective genomes, and all the BLAST hits overlapped by at least 60% of the length of the longest protein. This yielded 2,073 sets of orthologs (out of an average of 10,500 genes in the four organisms). We repeated our analysis, requiring that each best bidirectional hit also be the only BLAST hit in each genome (spanning 60% the length of the longest protein). This protocol yielded only 1,178 ortholog sets, but gave qualitatively similar results for intron gains and losses (Figure S1).

#### Ortholog alignment

The proteins in each ortholog set were aligned using ClustalW 1.82 (Chenna et al. 2003), and intron position characters were inserted into the alignments, using “0,” “1,” or “2” to indicate the intron phase. Phase 0 intron characters were inserted between the amino acids coded for by the codons adjacent to that intron, and phase 1 and 2 intron characters were inserted immediately following the amino acid coded for by the codon interrupted by the intron. If an intron was not present in all the sequences at a given position, special intron gap characters, were inserted in the other sequences in order to maintain the downstream amino acid alignment. A total of 9,352 intron positions were aligned. At only 28 (0.3%) of these positions were introns of different phases aligned, making it reasonable to ignore “phase shifting” in our analysis.

#### Alignment filtering

Regions of low alignment quality were eliminated with a filter that required at least 30% identity and 50% similarity in a window of ten residues on each side of the intron position. These parameters were determined following manual classification of a set of 181 randomly selected intron positions as “clearly homologous,” “ambiguous/possibly homologous,” or “non-homologous” (see Figure 2B). Using the parameters above, 92% of the homologous positions, 29% of the ambiguous positions and only 2% of the non-homologous positions passed the filter.

To further exclude likely annotation and alignment errors, intron positions were also filtered by eliminating positions adjacent to gaps in the amino acid alignment and by eliminating positions with nearby introns but low evidence of homology in the intervening sequence. It is possible that some of these positions may in fact reflect intron gain or loss events that occurred simultaneously with coding sequence insertion or deletion. However, removing these positions did not significantly impact our results, as the number of positions adjacent to gaps was only about one-tenth of the number of positions that passed the quality filter, and the introns removed did not have an apparent positional bias (see Figure 3A)

#### Statistical significance of high gain rate in PRPP synthetase

We modeled the number of gains in a particular organism as a Poisson distribution under two different null hypotheses. One null hypothesis was that the gains were spread uniformly across all genes. The other was that the number of gains in each gene was proportional to the length of the gene. In the first case the Poisson parameter λ is given by the total number of raw gains observed in that organism divided by the total number of ortholog sets *( p* < 3 × 10^−22^ for *M. grisea,* *p* < 4 × 10^−9^ for *N. crassa,* and *p* < 0.007 for *F. graminearum).* In the second case λ is given by the total number of raw gains observed in that organism multiplied by the length of that gene in amino acids and divided by the total number of amino acids in all genes in that organism *( p* < 7 × 10^−25^ for *M. grisea,* *p* < 3 × 10^−10^ for *N. crassa,* and *p* < 0.003 for *F. graminearum)*. We reported the less significant of the two *p*-values in the results.

#### Analysis of intron gain phase and sequence preference

For each of the three in-group lineages, the frequency of phase 0, 1, and 2 introns was determined for five different datasets: for each class of conservation (conserved, raw gains, and raw losses), for all introns passing our filter, and for all introns in the ortholog set. The *p*-value for the significance of phase 0 bias was determined by the χ^2^ test with two degrees of freedom using equal expected phase frequencies. To detect sequence bias at intron insertion sites, we examined gained introns separately in *F. graminearum,* *M. grisea,* and N. crassa. For each gained intron, we extracted four bases upstream and downstream of orthologous sites in the other two sequences, consistent with Qiu et al. (2004). The results are shown in Table 2.

## Supporting Information

Figure S1 Intron Gains and Losses Inferred from Best-Only BLAST Hit Orthologs Positional biases in intron gain, loss, and current distribution in three fungal genomes determined using orthologs predicted by a “bidirectional only hit” method. (A), (B), and (C) are roughly analogous to (D), (E), and (A), respectively, in Figure 3.
(78 KB DOC).

Table S1 Database of Alignments of All 1,447 Ortholog Sets with at Least One Passing Intron Position Also available at http://genes.mit.edu/NielsenEtAl/.
(4.3 MB ZIP).

## Abbreviations

PRPP: 1-phosphoribosyl-5-pyrophosphate
