# Supplementary material for: Patterns of Intron Gain and Loss in Fungi
Source: PLoS Biol. 2004 Nov 30;2(12):e422. doi: 10.1371/journal.pbio.0020422 (PMC532390; doi:10.1371/journal.pbio.0020422)
Supplement: Figure S1 — Positional biases in intron gain, loss, and current distribution in three fungal genomes determined using orthologs predicted by a “bidirectional only hit” method. (A), (B), and (C) are roughly analogous to (D), (E), and (A), respectively, in Figure 3. (78 KB DOC). [file pbio.0020422.sg001.doc]

**
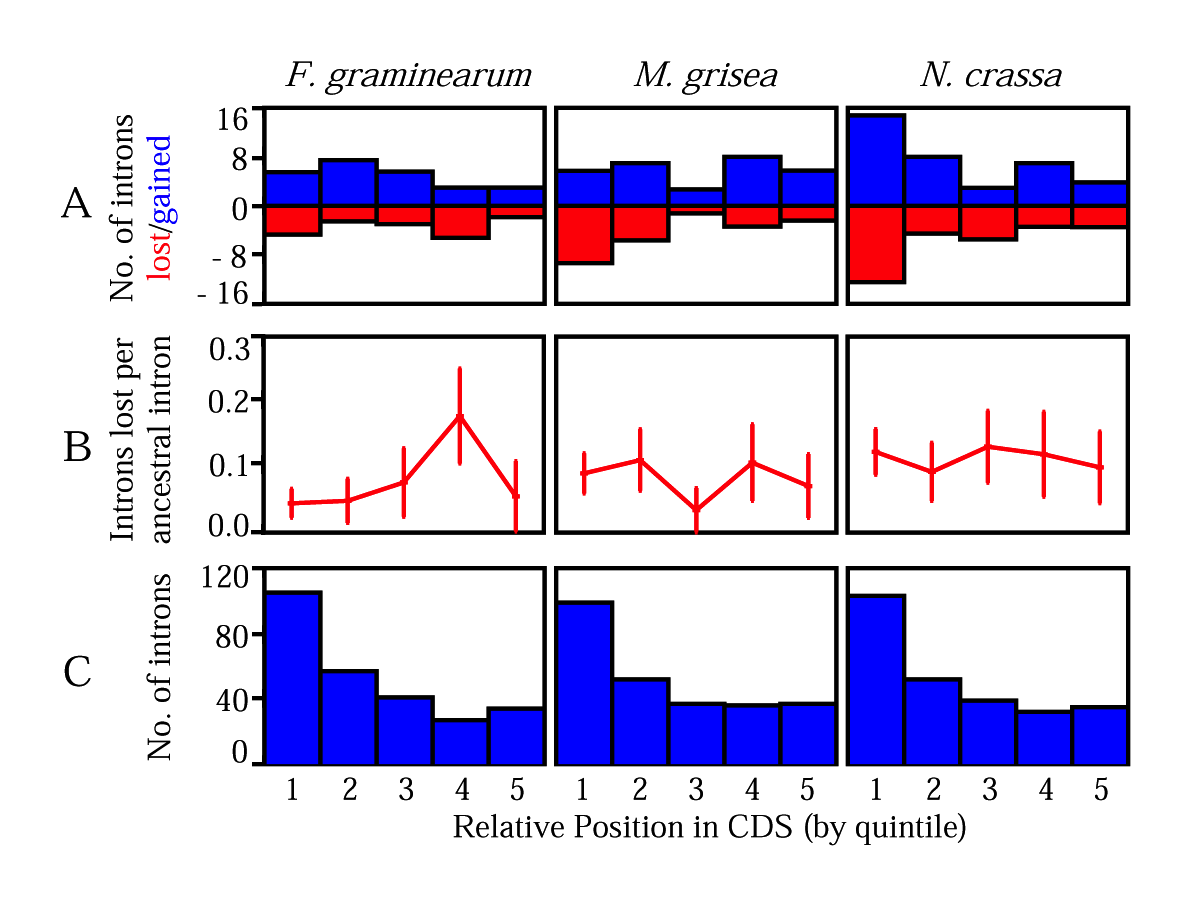
**

**Figure S1.** Positional biases in intron gain, loss and current distribution in three fungal genomes using orthologs predicted by a "Bi-directional Only Hit" method. Panels A, B and C are roughly analogous to panels D, E and A in Figure 4 of main text.
