# Supplementary material for: Patterns of Intron Gain and Loss in Fungi
Source: PLoS Biol. 2004 Nov 30;2(12):e422. doi: 10.1371/journal.pbio.0020422 (PMC532390; doi:10.1371/journal.pbio.0020422)
Supplement: Table S1 — Also available at http://genes.mit.edu/NielsenEtAl/. (4.3 MB ZIP). [file pbio.0020422.st001.zip › NielsenEtAl/html/1.html]

AN8059.1.NCU03798.1.MG07469.1.FG06120.1


```
 CLUSTAL W (1.82) Multiple Sequence Alignments - Introns Inserted


Sequence 1: AN8059.1	181 aa
Sequence 2: FG06120.1	178 aa
Sequence 3: NCU03798.1	194 aa
Sequence 4: MG07469.1	201 aa
Alignment Length: 215 aa
Number Identitical Residues: 79 aa
Alignment Score (without introns) 3952


MG07469.1 	MNFPGSSGLP----------GRGPAP---DPNEEQMKK0LKSFGESCAAKTVMSGVAGLG
NCU03798.1	MNFPGMPGG--------AAPSGGAAPGGYDPNDPNIKM0MQKAMESCFAKTVMSGGAGFA
FG06120.1 	MNFPGMTPP-------VG--GAAAPPGIGGPQDPNIKA0VQAAMESCFGKSVMSGVMGFG
AN8059.1  	MNFPGTSGSSAANMTGFGGMGAGGTQGMSEQEQAMVKM0MQNAMESCPLKTVISGVMGFG
          	***** .  .::. :. .. . . . .    ::  :*  ::   ***  *:*:**  *:.

MG07469.1 	LGAVFGLFMAS0MAYDTPFHHPTPDAAKAAAPKPPYAAGTGMVLPRGNYSPPTITPPPMS
NCU03798.1	LGGVFGMFMAS0MAYDTPYHSPTTPGTGPGANPAAAGI-------------PGYKPVDLS
FG06120.1 	MGGLFGMFMAS0MSYDTPYHTAAPGSP----------------------------QNTVT
AN8059.1  	LGGLFGMFMAG0MSYDS---SLTPQS------------------------------QTIA
          	:*.:**:***. *:**:     :. .                                ::

MG07469.1 	SLPMKTQIAAGFRDMGARSVSTGKNFGKVGAMFSGIECGIEGLRAKNDAGNGVAAGCVTG
NCU03798.1	SMPLKEQLKHGFKDMGQRSYSTAKNFAKVGALFSGIECGIEGLRAKNDLGNGVAAGCLTG
FG06120.1 	SLPLKQQLKIGFKDMGTRSWSMAKNFGKVGALYSGVECGIEGLRAKNDLTNSVAAGCLTG
AN8059.1  	NLPWRQQLKHGFKDMGSRSWSSAKNFGIVGALYSGTECCIEGLRAKNDLTNSVSAGCITG
          	.:* : *:  **:*** ** * .***. ***::** ** *********  *.*:***:**

MG07469.1 	AILARNGGPQAAAIGCAGFAAFSAAIEMWLRSPKDE-
NCU03798.1	AILAKNGGPQAAAVGCAGFAAFSAAIDAWMRMPSEED
FG06120.1 	GILAKNAGPQAAAGGCLAFAAFSAAIDAYMRSPPKDD
AN8059.1  	GILGAKAGPQAAALGCAGFAAFSAAIDAYMRMPESD-
          	.**. :.****** ** .********: ::* * .:
```
