# Supplementary material for: Patterns of Intron Gain and Loss in Fungi
Source: PLoS Biol. 2004 Nov 30;2(12):e422. doi: 10.1371/journal.pbio.0020422 (PMC532390; doi:10.1371/journal.pbio.0020422)
Supplement: Table S1 — Also available at http://genes.mit.edu/NielsenEtAl/. (4.3 MB ZIP). [file pbio.0020422.st001.zip › NielsenEtAl/html/10.html]

AN7105.1.NCU07831.1.MG05243.1.FG02544.1


```
 CLUSTAL W (1.82) Multiple Sequence Alignments - Introns Inserted


Sequence 1: MG05243.1	865 aa
Sequence 2: FG02544.1	859 aa
Sequence 3: NCU07831.1	872 aa
Sequence 4: AN7105.1	860 aa
Alignment Length: 878 aa
Number Identitical Residues: 455 aa
Alignment Score (without introns) 21701


MG05243.1 	MSRFFRGGNESSSESSSDEEELYS-EEEEEELEDEGDDSDNDGSGDDDSD--SDSDADAG
NCU07831.1	MSRFFRGGDDSSTDSSSEEEEVYTSEEEEEKVQAEDESSSEEESDEEESDEESSSDEEEG
FG02544.1 	MSRFFRGGDDSSSDSSSEEEELYS-EEEEE---EEQQDSQAESSENDDSD--DSDSDDEG
AN7105.1  	MSRFFYGNDSDSDSSGSDEEELYSDEEVEQ---SEEESSEEDASSEEESS------EDED
          	***** *.:..* .*.*:***:*:.** *:    * :.*. : * :::*.       : .

MG05243.1 	GKKKGAAKFLVDDDSSDEED---SDAEVTTKVKSAKDKRLDELESTVTAIANGMKINDWG
NCU07831.1	TKKKGASRFLQSDDESEEEEEEQSDDEATTKVKSAKDKRFDELESTISQIQNGQKINDWS
FG02544.1 	GK--GINKFLKD-ASSDSDD---SDDEVRAKVKSAKDKRLDELESSIKQIENGQKNGDWT
AN7105.1  	AGKAGASRFMKDVSDSEESE----EEDVVKVVKSAKNKRLEELESTIKLIDNAQKINDWA
          	  . *  :*: .  .*:..:    : :.   *****:**::****::. * *. * .** 

MG05243.1 	SIATE1FDKLNRQAEKLRTGTTAPKMYIKSIADLEDFMNETLAKQKVTPKKMNATNARGL
NCU07831.1	LIANE1FDKLNRQVVKLQDGSKAPKSYIKAIADLEDFMNETLAKQKVTPKKMNATNARGL
FG02544.1 	LIAAE1YEKLNRQVAKMP-GSRPPKPYIRILAELEDFMNESLAKQKVTPKKMNAIQNRSL
AN7105.1  	VISSE1FDKMNRQVVKVLQSGPVPKIYVKTVADLEDFVNETVAKQKSSNKKMNASNAKGF
          	 *: * ::*:***. *:  .   ** *:: :*:****:**::**** : ***** : :.:

MG05243.1 	NAVKQRIKKNNKEYQAQIDAYKADEFEFMMDTEDEEEPAPKPKKAAKVSFD-EATAEDEE
NCU07831.1	NAVKQRIRKNNKEYQTQIDAYRKDA-DAFMES-DDEVAAPKVVSKVRFEAP-VVSAEQQE
FG02544.1 	NAVKQKVKKTNKEYQSQIDAYRADK-DAFMDSSDNEPVVPTVSKPKKVSLQ-VDAAPAAE
AN7105.1  	NAIKQKIKKNNKEYAAQIEKYRADK-DSYMESDEEEEKKPAIAAPRLSKLERVEAPVAVA
          	**:**:::*.**** :**: *: *  :  *::.::*   *        .     :.    

MG05243.1 	-DDEGFARVGKGGKTLQFTPESIFKHLRSILESRGKKNTDRGEQIKIMEKLGEIA-QTPY
NCU07831.1	EDDKGFSTVDSRGKVVQYTPESILKHLRAIIESRGKKNTDRLEQIKVMETLNKVVPITPY
FG02544.1 	GDDDGFATVGKGGRTLQYTPESIFKHLRTIMESRGKKNTDRTEQIKTMEKLSEIA-NTPY
AN7105.1  	GDDDGFETVVR-GKTLQYTPESILKHLRVIVESRGKKNTDRLEQIKTMEKLLEVA-QTPY
          	 **.**  *   *:.:*:*****:**** *:********** **** **.* ::.  ***

MG05243.1 	QRIRVLLALVSSRFDIGT-SAGAALSVEHWKAAEKELSLLLTVLDENKDHIVLESAEEWD
NCU07831.1	QKIRVLQTLISARFDLGA-GGAAQMPLDQWKAAERDLASLLEILEKEKDHVVVEGAEEWD
FG02544.1 	QKIRVLLTLVSTRFDIGS-GGASAMTVENWKAAEKELSSLLQVLEENHDYVVVENAEEWD
AN7105.1  	QRIRVYLTLISTRFDLSTTSTAAYMSVEQWKAAEQELSTLLSVLEKERNYVVSEGAEEWE
          	*:***  :*:*:***:.::. .: :.:::*****::*: ** :*:::::::* *.****:

MG05243.1 	DDEKPPTLEKGEKYIKIPGSIVSYTERLDDELTRSLQAIDPHTSEYIDRLSDEGALYNII
NCU07831.1	DDDKLPTIPEGEKYLKVPGSVVSLIERLDDELTRSLQAIDPHTSEYIDRLTDEGSLYNTI
FG02544.1 	DDEKPPTLQEGEKHIKVPGSIVSYIERLDDELVRSLQSIDPHTSEYIERLQDEGSLYNII
AN7105.1  	DDEKQPQVAAGETFY-IPGSIVSYVERLDDELTRSLQHIDPHTAEYIERLSDEKQLYTNL
          	**:* * :  **..  :***:**  *******.**** *****:***:** **  **. :

MG05243.1 	FRAQLYYEGLRKDASLEIPQESLNRAIMRRLDHVYFK0PAQVIKILEENSWKAVGD-KAS
NCU07831.1	FRGLLYYEHLRKDASLEVPQESLNRIIQRRLDHVYYK0PAQVVKILEENAWKQVSAEADS
FG02544.1 	FRGLLYYEYLRKDDSLEIPQDSINRIVMRRLEHVYFK0PAQVVQTFEENCWKPVGDSVES
AN7105.1  	VRTQIYVEGLTKLEKTELRQDSLNRVVMRRLEHIYFK~PSQVITILEEGTDKALPSELET
          	.*  :* * * *  . *: *:*:** : ***:*:*:* *:**:  :**.  * :  .  :

MG05243.1 	NITPREQTVEAAQLVNVLCNYLFANSEGIHRARAMLCQIYFLALHDDYYKSRDMMLMSHL
NCU07831.1	EITPRSQSGDAGKLINILSNYLFENSEGIIRARAMLCQIYFLALHDEYYKSRDLMLTSHL
FG02544.1 	VITPRDQAQNAGNLVNVLCNYLFSNSEGIIRARAMLCQIYFLALHGEYYKARDMMLMSHL
AN7105.1  	SITTRGNS-DAQTLVQTLCNYLFRNSDGILRARAMLAQIYFLALHDQYYRARDLMLMSHL
          	 **.* :: :*  *:: *.**** **:** ******.********.:**::**:** ***

MG05243.1 	QETISSFDVLTQILYNRTLVQVGLCAFRKGLVYDAQNTLQDICGSGRQKELLAQGVMIQR
NCU07831.1	QETIANFDIATQILYNRTLVQVGLCAFRKGLVYDAQNTLQEICGSGRQKELLAQGVMIQR
FG02544.1 	QENIPNFDVQSQILYNRTLVQVGLCAFRKGLVYEAQNTLQEICGSGRQKELLAQGVMMQR
AN7105.1  	SENIANFDVSSQILFNRTLVQIGLCAFRAGLIYEAQNTLSEICGSGRQKELLAQGIILQR
          	.*.*..**: :***:******:****** **:*:*****.:**************:::**

MG05243.1 	FNQVSPEQERLERQRQLPFHMHINLELLECVYLTCSMLLEIPLLAQTGSSPDIKKRIISK
NCU07831.1	YSQVTPEQERLEKQRQLPFHMHINLELLECVYLTCSMLLEIPLLAQTGSSPDVKKRIISK
FG02544.1 	YNQVSPEQERLEKQRQLPFHMHINLELLECVYLTCSMLLEIPLLAQTGSSPDVKKRVISK
AN7105.1  	YSTVSPEQERLERQRQLPFHMHINLELLECIYLTSSMFLEVPLMAQTSSSPEMKRRVISK
          	:. *:*******:*****************:***.**:**:**:***.***::*:*:***

MG05243.1 	TYRRMLEYHERQIFTGPPENTRDHVMQASKALAAGEWKKATSFIHSIKIWELMPNADAIK
NCU07831.1	TYRRMLEYHERQIFTGPPENTRDHVMQASKALAAGEWKKATDFIHSIKIWDLMPNTEGIK
FG02544.1 	TYRRMLEYHERQIFTGPPENTRDHVMQASKALAAGEWKKSTDFIHSIKIWELMPGAEEIK
AN7105.1  	TFRRMLDYNERQVFTGPAENTRDGVIMSAKFLAAGDWKKAAEMLNSIKIWDLMPQPEKIK
          	*:****:*:***:****.***** *: ::* ****:***::.:::*****:*** .: **

MG05243.1 	TMLAKQIQEEGLRTYLFTYAPFYDTLSIETLSNMFELESTKISAVVSKMISHEELAAALD
NCU07831.1	TMLAKQIQEEGLRTYLFTYAPFYDTLAIATLSSMFELDSRKVSAVVSKMISHEELAAALD
FG02544.1 	AMLSKQIQEEGLRTYLFTYAPFYDTLSTETLSAMFELDSAKVAAVISKMISHEELAASLD
AN7105.1  	EMLSQQIQEEGLRTYLFTYAPFYDSLSISTLSTMFELSEKKIAAIISRMISHEELGAALD
          	 **::*******************:*:  *** ****.. *::*::*:*******.*:**

MG05243.1 	QVKQTVIFRKGVELSRLQSLALTLSDKASSLIESNERTLEQRTQGTSNAFER-QGGRGGR
NCU07831.1	QVTETVIFRKGVELSRLQSLALTLSDKASSLIETNERTLEQKTQGSANAFSR-KDNRGGG
FG02544.1 	QVTSTVIFRKGVELSRLQSLALALSDKASALIESNERTLEQKTQGTSNAFER-QGGRGRG
AN7105.1  	QVNDAIVFRKGVELSRLQSQIVTLADKSMNLLEANEKTLEQRTQGMANAFQRDQGAGARG
          	**..:::************  ::*:**:  *:*:**:****:*** :***.*.:.  .  

MG05243.1 	GGG-RGRGGGRGGPRFGGNTQRQAGGTQFTGGALGAAVRA
NCU07831.1	QRG-GGQRGGRGGARTGGNPQRQAGGTQFTGGALGNAVRG
FG02544.1 	GRG-RGGGQGRGGARAGGNTQRQAGGTQFTGGALGAAVRG
AN7105.1  	GRGPRGGGQARGGPRLPGGQQRRPGGQQFGGGALGGAIKA
          	  *. *   .***.*  *. **:.** ** ***** *::.
```
