# Supplementary material for: Patterns of Intron Gain and Loss in Fungi
Source: PLoS Biol. 2004 Nov 30;2(12):e422. doi: 10.1371/journal.pbio.0020422 (PMC532390; doi:10.1371/journal.pbio.0020422)
Supplement: Table S1 — Also available at http://genes.mit.edu/NielsenEtAl/. (4.3 MB ZIP). [file pbio.0020422.st001.zip › NielsenEtAl/html/100.html]

AN5996.1.NCU00979.1.MG10827.1.FG05605.1


```
 CLUSTAL W (1.82) Multiple Sequence Alignments - Introns Inserted


Sequence 1: NCU00979.1	110 aa
Sequence 2: FG05605.1	109 aa
Sequence 3: MG10827.1	109 aa
Sequence 4: AN5996.1	109 aa
Alignment Length: 111 aa
Number Identitical Residues: 73 aa
Alignment Score (without introns) 2886


MG10827.1 	MKHLAAYLLLGLAGNESPSASDIKTVLESVGIEADDERLEKLISELKGKDIKE0LIAEGS
NCU00979.1	MKHLAAYLLLTLGGNTAPSAADVKAVLESVGIEADSERLDKLISELEGKDLNE0LIAEGS
FG05605.1 	MKHLAAYLLLGLGGNTSPSAADVKAVLTSVGIDADEDRLNKLISELEGKDIQQ0LIAEGS
AN5996.1  	MKHLAAYLLLALAGNESPSASDIKEVLSSVGVDADDERLEKLIAELQGKDINE0LIAEGT
          	********** *.** :***:*:* ** ***::**.:**:***:**:***::: *****:

MG10827.1 	AKLASVPSGGGGG--GGAAAAAGGAAPEAAKEEEKEEE1KEESDEDMGFGLFD
NCU00979.1	SKLASVPSGGAAAPAAGGAAAAAGGAAEE-KKEEKVEE1KEESDEDMGFGLFD
FG05605.1 	EKLASVPSGGAGG-ASGGAAAAGGAAEEA-KEEEKEEE~KEESDEDMGFGLFD
AN5996.1  	TKLASVPSGGAGG--AAPAAAAGGAAAAEAPAAEKEEE~KEESDEDMGFGLFD
          	 *********...  .. ****.*.*   :   ** ** **************
```
