# Supplementary material for: Patterns of Intron Gain and Loss in Fungi
Source: PLoS Biol. 2004 Nov 30;2(12):e422. doi: 10.1371/journal.pbio.0020422 (PMC532390; doi:10.1371/journal.pbio.0020422)
Supplement: Table S1 — Also available at http://genes.mit.edu/NielsenEtAl/. (4.3 MB ZIP). [file pbio.0020422.st001.zip › NielsenEtAl/html/1000.html]

AN2308.1.NCU06624.1.MG06104.1.FG05303.1


```
 CLUSTAL W (1.82) Multiple Sequence Alignments - Introns Inserted


Sequence 1: NCU06624.1	909 aa
Sequence 2: FG05303.1	815 aa
Sequence 3: MG06104.1	892 aa
Sequence 4: AN2308.1	828 aa
Alignment Length: 951 aa
Number Identitical Residues: 419 aa
Alignment Score (without introns) 20204


MG06104.1 	--------MT~--SRTSSYSSQVPRQP---SRRPTLNATLSTSLSSADQVEDADVQGAAV
NCU06624.1	MSRGGPGAMN~SQPRSTSFSTSVPQIPNQRARRPSVSSRFSYAVSIAEQGEAQTAQGVAA
FG05303.1 	--------MN~--VRQSSFSLAPPGP----ERRPSVVSRLSSVFSNADR-EAGTTTSTGA
AN2308.1  	--------MT0FNKAMSSMPVSSSSRSPLPSPSPLTERRLFRTFSGLSASSRPRSPTGNG
          	        *.  .   :* .   .  .      *     :   .*  .  .         

MG06104.1 	ESQIEQEIAEIKRYE~VRLIQPATPVPQCPEFGQLTFLPQDFTTID1WVQDAAQERLRRK
NCU06624.1	QHQIEEEIAQIKRYE0------------------------DFTTID1WVQDAAREQLRRK
FG05303.1 	G-LIEEEIAEIKRYE0------------------------DFTTID1WVQDAAREQAKRK
AN2308.1  	HAPVTEEISEIKRYE~------------------------DFTTID~WVQDAVHEQARRR
          	   : :**::*****                         ****** *****.:*: :*:

MG06104.1 	ARRKRRQSSVYDN-SYVGWRSWAAESYEAAQGWIVVTIIGIAIGLNAAFLNIVSEWLSDI
NCU06624.1	ARRKR-NAGLWDA-GRFDWRHRIRESYDAAQGWIVVTIIGAAIGVNAAFLNIITEWLADI
FG05303.1 	VRRKQ-AAGLYDK-GQPGWRYQLWKSYDAAQAWIVVTIIGVAIGLNAALLNIITEWLSDV
AN2308.1  	IKRQE-GSGFWDKEGTFKWRLKVRESYDAGQAWLVITIVGAVIGLIAAVLNIITEWLSDI
          	 :*:.  :..:* ..   **    :**:*.*.*:*:**:* .**: **.***::***:*:

MG06104.1 	KLGHCKTAFYLNENFCCWGEDNG1CNDWQPWTNFGPINYIIYIIFA0TIFACTAATLVKS
NCU06624.1	KLGHCKTAFYLNENFCCWGEDNG~CDDWQKWTGFSPINYLIYILFA0ILFACTSATLVKS
FG05303.1 	KMGYCETGFYLNENFCCWGEGNG1CDQWHRWTGFEPFNYFVYLVFA0------------S
AN2308.1  	KLGYCTTAFYLNQQFCCWGAEGA1AK--------------------~---------LVKS
          	*:*:* *.****::*****  .. ..                                .*

MG06104.1 	YAPYAAGSGISEIKCIIAGFVMKGFLGSWTLLIKSVGLPLTIGSGLSVGKEGPSVHYAVC
NCU06624.1	YAPYAAGSGISEIKCIIAGFVMKGFLGFWTLVIKSLALPLAIGSGLSVGKEGPSVHYAVC
FG05303.1 	FAPYAAGSGISEIKCIIAGFVMKGFLGWWTLIIKSICLPLAIASGLSVGKEGPSVHYAVC
AN2308.1  	FAPYAAGSGISEIKCIIAGFVMKGFLGGWTLLIKSIALPLAIASGLSVGKEGPSVHFAVC
          	:************************** ***:***: ***:*.*************:***

MG06104.1 	TGNVISRLFAKYRSNASKTREILSACAAAGVAVAFGSPIGGVLFSLE~EMSSYFPLKTLW
NCU06624.1	TGNVISRLFAKYRRNASKTREILSACAAAGVAVAFGSPIGGVLFSLE~EMSSYFPLKTMW
FG05303.1 	TGNVISRLFDKYKRNASKTREFLSASAAAGVAVAFGSPIGGVLFSLE~EMSNQFPLKTLW
AN2308.1  	TGNVISRFFTKYKRSASKTREVLTATAAAGVAVAFGSPIGGVLFSLE0EVASYFPLKTLW
          	*******:* **: .******.*:* ********************* *::. *****:*

MG06104.1 	RSYFCALVATSVLA~AMNPFRTGQLVMFQVHYDRTWHFFEIAFFAILGIFGGLYGAFVIK
NCU06624.1	RSYFCALVATAVLS~AMNPFRTGQLVMFQVHYDRSWHFFEILFFILLGIFGGLYGAFVMK
FG05303.1 	RSYFCALVATAVLA~AMNPFRTGQLVMFQVEYKNDWHFFELLFYVLIGIFGGLYGAFVIK
AN2308.1  	RSYFCALVATGVLS0VMNPFRTGQLVMFQVRYDRSWHFFELIFFVIIGIFGGLYGALVIK
          	**********.**: .**************.*.. *****: *: ::*********:*:*

MG06104.1 	WNLKVAAFRKKYLKEYPIVEASLLAFITAILCFPNVFLRIEMTESMEILFLECEGGEDYH
NCU06624.1	WNLRVQAFRKKYLTNYAILEATLLAAATAIVCYPNSFLRIDMTESMEILFLECEGAEDYQ
FG05303.1 	WNLRVQSFRKRYLKDYAVLEATLLAAGTAIIAYPNAFLRIDMTESMEMLFSECGHGESYH
AN2308.1  	WNLRVQAFRKKYLSQHAVVESVILAVVTAVICFPNMFLKINMTEMMEILFQECEGEHDYH
          	***:* :***:**.::.::*: :**  **::.:** **:*:*** **:** **   ..*:

MG06104.1 	GLCE~AKNRAWNIMSLLIALILRTGLVIISYGCKVPAGIFVPSMAIGALFGRTIGIVVQA
NCU06624.1	GLCE~RDHRFRNVVSLLLATVIRIFFVIISYGCKVPAGIFVPSMAIGASFGRSVGIIVQA
FG05303.1 	GLCE~PSKRWWNITSLFLATFLRLFLVILSYGCKVPAGIFVPSMAVGASFGRMVGIIVQA
AN2308.1  	GLCE2SKYRWSMVFSLATATILRIFLVIISYGCKVPAGIFVPSMAIGASFGRMVGIMVQA
          	****  . *   : **  * .:*  :**:****************:** *** :**:***

MG06104.1 	LQESFPSSSFFSSCAPDVPCITPGTYAFLGAAAALSGIMHITVSVVVIMFELTGALDYIL
NCU06624.1	LHEANPQSPFFAACLPDVPCITPGTYAFLGAAAALSGIMHITVSVVVIMFELTGALTYIL
FG05303.1 	IHEANPTSVFFSACKPDEPCITPGTYALLGAAAALSGIMHITISVVVIMFELTGALNYIL
AN2308.1  	LHESFPDSKFFAACEPDLPCITPGTYAFLGAGAALSGIMHLTISVTVIMFELTGALTYIL
          	::*: * * **::* ** *********:***.********:*:**.********** ***

MG06104.1 	PTM~IVVGITKMVSDRLGHGGIADRMIWFNGFPYLDNKEEHNLGLPVSAAMTSDLDTIPI
NCU06624.1	PTM~IVVGVTKAVSELFGKGGIADRMIWFSGFPFLDNKEDHNLGVPVSHAMIKDVTSIPT
FG05303.1 	PTM~IVVGVTKAVSELFGKGGIADRMIWFSGMPFLDSKEEHNFGVPVSAVMRTSVVSMPV
AN2308.1  	PTM0IVVGVTKAVGDRFGNGGIADRMIWANGFPFLDNKEDHVFNVPVSHAMTTDPVSLPA
          	*** ****:** *.: :*:********* .*:*:**.**:* :.:*** .* ..  ::* 

MG06104.1 	AGMTMESVERLLAKDNYQGFPIVEENNNAAESSNNARRGRNLVGFIGRTELRYAVDRARR
NCU06624.1	NGMTLQQIEGLLAEDNYQGFPIVED-----------EHSKILVGYIGRTELRYAVDRAKR
FG05303.1 	HGLTLGEVQRLLADDRYQGFPVVED-----------KHTKVLIGYIGSIELRYAIDKMSR
AN2308.1  	SDFPVREAEHLLNDNKFQGFPIIED-----------RSSKILVGYIGRTELRYAIDRARR
          	 .:.: . : ** .:.:****::*:           .  : *:*:**  *****:*:  *

MG06104.1 	ERQISPSAKCNFMPTDVNAVTP------------------ITPAGNSRRASFLGDAYTVD
NCU06624.1	ERTLSPQAKCTFAPPPSADVTTPGTDIITPGLARMDSFNTIGFAEPSTTASAS-SSNFIN
FG05303.1 	TSPLSETSRCTFAPSSSNLSTT------------------------SLHGDSS-HSSTLD
AN2308.1  	EGMISPSAQCVFTKDAAEASVARR--------ASSTLQRTLLTPDTFDNIESSSGASFVD
          	   :*  ::* *        ..          :      :   .      .  . :  ::

MG06104.1 	FSRFVDSTPVTVHPRLPLETVMELFRKIGPRVILIEHRGRLLGLVTVKDCLKYQFKAEAA
NCU06624.1	FSRYVDTTPVSAHPRLPLETVMELFRKIGPRVILIEYHGKLMGLVTVKDCLKYQFKVEAM
FG05303.1 	FSRYVDSTPVTAHPRLPLETVMELFQKIGPRVILIEYHGKLTGLVTVKDCLRYQFKVEAA
AN2308.1  	FSRYIDNTPLTVHPRLPLETVMEIFKKMGPRVILVEHRGRLTGLVTVKDCLKYQFKVEAE
          	***::*.**::.***********:*:*:******:*::*:* *********:****.** 

MG06104.1 	ENGSSSRADD--------DDEVQEKIWAFLQRAAWWLGDKVS-LASGGRISLNGSFDETS
NCU06624.1	EEVANNGQHSGHGQGNGAEQQGDERLWELMQRVAGWVSDKVS-IASGGRIRLRDSLDLPR
FG05303.1 	ENPKDNHRME----------EGQEQLWNLLLRAGNWFSCRVS-RYSGGRIRLSSS-----
AN2308.1  	EQALAATHHP------------ELPLGAYQAKDNGTLEERIWNLMQKIGSRFSKS-----
          	*:                    :  :     :    .  :: .  .     :  *     

MG06104.1 	RVSR--------TGHRAGNSGNSRGSTGQILEGTEDEMGDEAGGVEMEYR----------
NCU06624.1	ETLAGGAGARVGSGTNRTVRSQGGARDDQILDGTEDED---EDGVELENRQTYPHGSTSR
FG05303.1 	------------SGDERPLGR------GQILDGDEEVL---DEGVELESRR---------
AN2308.1  	------------SGQPRDAMPLPQDDQSPIGVGNDADG----RMVELEERP---------
          	            :*             . *  * :         **:* *          

NCU06624.1	
FG05303.1 	
MG06104.1 	
AN2308.1  	
          	
```
