# Supplementary material for: Patterns of Intron Gain and Loss in Fungi
Source: PLoS Biol. 2004 Nov 30;2(12):e422. doi: 10.1371/journal.pbio.0020422 (PMC532390; doi:10.1371/journal.pbio.0020422)
Supplement: Table S1 — Also available at http://genes.mit.edu/NielsenEtAl/. (4.3 MB ZIP). [file pbio.0020422.st001.zip › NielsenEtAl/html/1001.html]

AN5140.1.NCU09098.1.MG02722.1.FG08810.1


```
 CLUSTAL W (1.82) Multiple Sequence Alignments - Introns Inserted


Sequence 1: MG02722.1	458 aa
Sequence 2: FG08810.1	449 aa
Sequence 3: NCU09098.1	520 aa
Sequence 4: AN5140.1	454 aa
Alignment Length: 527 aa
Number Identitical Residues: 237 aa
Alignment Score (without introns) 10675


MG02722.1 	-------------------------------------------~----------MAVVDA
NCU09098.1	MALSTSPFWEGNREDSEDVVVIELNQVGHGGSDLDILQATQLH2NKKGSFSAGMTTPVEP
FG08810.1 	-------------------------------------------~----------MAAFDP
AN5140.1  	-------------------------------------------~-----------MAVPA
          	                                                         . .

MG02722.1 	AARKRVLRVIFISLLLDL0ISFTFILPLFPKLLEFYRNSEAPTGLLDKRPRTLLSNVLDY
NCU09098.1	ATRKRVLKVIFISLLLDL0ISFTFILPLFPKLLEFYRNVEAPLDPSAPPSKTLLSSVLGY
FG08810.1 	AARKRVLRVIAISLLLDL0ISFTFILPLFPQLLEFYRDREGQVPLDKDAPQTLLQHVLSG
AN5140.1  	EQRKKILKVLMTSLLLDL0ISFTFILPLFPSLLSFYRERD-------PSPSSPLNTVFHY
          	  **::*:*:  ****** ***********.**.***: :         . : *. *:  

MG02722.1 	LNAYKAAFARPIDSRYDIVLLGGALGSLFS2LLQAIASPIIGQLSDRYGRRTALLSSMMG
NCU09098.1	LNAYKASFARPIDSRYDIVLLGGALGSLFS2LLQAIASPIIGHFSDRYGRRTALLLSMTG
FG08810.1 	LHRYKASFSRPIESRHDIVLLGGALGSLFS2LLQAIASPLIGALSDRYGRRKALLASMCG
AN5140.1  	LNAYKNAFAKPIDSRYDIVLLGGALGSLFS~LLQAIAAPVIGRLSDKHGRRKALLASMLG
          	*: ** :*::**:**:************** ******:*:** :**::***.*** ** *

MG02722.1 	NILSVLLWVMAVDFRTFVLSRVVGGLSEGNVQLATAIATDISDESSRGSTMALIGACFSI
NCU09098.1	NILSVLLWVMATDFRTFLASRIVGGLSEGNVQLATAIATDISDPSKRGSTMALIGACFSI
FG08810.1 	NILSVLLWVAAVDFRTFIASRIVGGLSEGNVQLATAMASDISDESSRGATMALIGACFSI
AN5140.1  	NTLSVALWVAATDFRTFLASRIVGGLSEGNVQLAHAIATDISEPSQRGSTMALVGACFSI
          	* *** *** *.*****: **:************ *:*:***: *.**:****:******

MG02722.1 	AFTFGPALGAYLSSIPLVAANPFATAAGFSLFLIVAETIYLYAALPETLPALTETKKSAS
NCU09098.1	AFTFGPALGAWLSSFSTVAANPFATAAGVSLTLIVVETLYLYFCLPETLPALTQKTEPTG
FG08810.1 	AFTFGPGLGAWLSTFSTFTANPFAAAAGFSLALIVTETVYLYFSLPETLPSMRDTGAKGD
AN5140.1  	AFTFGPALGAYLSGIMTVKANPFATAAGVSLGLILLETVYIYACLPETHPQITGSSSTSA
          	******.***:** :  . *****:***.** **: **:*:* .**** * :  .     

MG02722.1 	AKGNDTTTKPKS------------AVRTNSHFMLNATHLFFLLFFSGMEFSLPFMTYDLF
NCU09098.1	TQVSSTKSEEKKPATTPTTTKSTAVQRTNSHFLLNFTHFSFLLFFSGMEFSLPFMTYDLF
FG08810.1 	AK---KKVAPKK------------IERTNSHFLLNAIHFVFLLFFSGMESSLSFMTYELF
AN5140.1  	PTLNEDSKKTKEAP----KAKSSTYQYSNNPTTLNILHLLFLLPFSGMEFSLPFLTATFY
          	.  .. .   *...    .:.:::   :*.   **  *: *** ***** **.*:*  ::

MG02722.1 	GYTSAK----NGRLLGYIGLVASILQGGVTRRLPPLMTVRVGVVACLLAFGILSRASSVA
NCU09098.1	AYDSAK----NGRLLGFVGLIASLLQGGVTRRLPPLLSVRIGVIACLLAFIMLGRITSVL
FG08810.1 	SFTSGK----NGRLLGYVGLVASILQGGVTRRLPPLMSVRVGTLACLASFILLGRVNTIG
AN5140.1  	TNSTASPAALNGRLLSTMGLIASLLQGTVVRRLPPLLVVRIGVASATISFFLLSRVTSIA
          	   :...:: *****. :**:**:*** *.******: **:*. :.  :* :*.* .:: 

MG02722.1 	ALYVAATCLATTSATVVTGLNTLSSLEAGEAERGGKLGNLRSWGQLGRGLGPVLFTSIYW
NCU09098.1	GLYGAAALLATTSATVVTGLNALSSFESHEGERGGKLGMLRSWGQLGRGLGPVLFTSIYW
FG08810.1 	GLYLAATCLAVTSATVVTGLNALSSFEAHEDERGNKLGMLRSWGQLGRGLGPILFTSVYW
AN5140.1  	GLYTAATFLAVTSASVVTGLNALGSLEAREEERGVVLGRLRSWGQVGRAAGPVLFCTLFW
          	.** **: **.***:******:*.*:*: * ***  ** ******:**. **:** :::*

MG02722.1 	WAGRETAYTIGALGIAGVSAGVFMGLKSPPGFGKAGASQAQNGAGEKKDL
NCU09098.1	WAGREVAYGIGALGMSWVALLVMYALKTPPGSERVRKESHVAEKKEL---
FG08810.1 	WAGREVAYTMGATGIAVVAAAVFGGLKTPKGMNTKGKKVETKAQ------
AN5140.1  	WVGREVAYLTGSVVMAGVCMGVFLGLRAPAVPPAAGEGKPVR--------
          	*.***.**  *:  :: *.  *: .*::*
```
