# Supplementary material for: Patterns of Intron Gain and Loss in Fungi
Source: PLoS Biol. 2004 Nov 30;2(12):e422. doi: 10.1371/journal.pbio.0020422 (PMC532390; doi:10.1371/journal.pbio.0020422)
Supplement: Table S1 — Also available at http://genes.mit.edu/NielsenEtAl/. (4.3 MB ZIP). [file pbio.0020422.st001.zip › NielsenEtAl/html/1002.html]

AN3065.1.NCU02283.1.MG00925.1.FG00337.1


```
 CLUSTAL W (1.82) Multiple Sequence Alignments - Introns Inserted


Sequence 1: NCU02283.1	452 aa
Sequence 2: FG00337.1	380 aa
Sequence 3: MG00925.1	400 aa
Sequence 4: AN3065.1	404 aa
Alignment Length: 461 aa
Number Identitical Residues: 259 aa
Alignment Score (without introns) 10685


MG00925.1 	MATTQ--TTQGQIQPCRYKVGKTLGAGSYSVVKECVHIDTGRYYAAKVINKRLMAGREHM
NCU02283.1	MSAAN--GRQPEVQPCRYKVGKTLGAGSYSVVKECVHIDTGRYYAAKVINKRLMAGREHM
FG00337.1 	MDSAA--RQQPQVQPCRYKVGKTLGAGSYSVVKECVHIDTGRYYAAKVINKRLMAGREHM
AN3065.1  	MASQVQPGQKPKVQPCRYKTGKTLGAGSYSVVKECVHIDTGQYYAAKVINKRLMVGREHM
          	* :  ..  : ::******.*********************:************.*****

MG00925.1 	0VRNEIAVLKKVSMGHQNILTLVDYFETMNNL~YLVTDLALGGELFDRICRKGSYYESDA
NCU02283.1	0VRNEIAVLKKVSMGHQNILTLVDYFETMNNL~YLVTDLALGGELFDRICRKGSYYESDA
FG00337.1 	0VRNEIAVLKKVSMGHQNILTLVDYFETMNNL~YLVTDLALGGELFDRICRKGSYFESDA
AN3065.1  	0VRNEIAILKQVSTGHQNILTLVDYFETMNNL1YLVTDLALGGELFDRICRKGSYYESDA
          	 ******:**:** ****************** **********************:****

MG00925.1 	VDLIRATLSAVAYLHDHGIVHRDLKPENLLFRTPEDNADLLIADFGLSRIMDEEQFHVLT
NCU02283.1	ADLIRATLSAVAYLHDHGIVHRDLKPENLLFRTPEDNADLLIADFGLSRIMDEEQFHVLT
FG00337.1 	ADLVRATLSAVAYLHDHGIVHRDLKPENLLFRTPEDNADLLIADFGLSRIMDEEQFHVLT
AN3065.1  	ADLVRAILSAVAYLHDHGIVHRDLKPENLLFRTPEDNADLLIADFGLSRIMDEEQLHVLT
          	.**:** ************************************************:****

MG00925.1 	TTCGTPGYMAPEIFKKTGHGKPV~DIWALGVITYFLLCGYTPFDRDSDLEEMQAILNADY
NCU02283.1	TTCGTPGYMAPEIFKKTGHGKPV~DIWALGVITYFLLCGYTPFDRDSDFEEMQAILNADY
FG00337.1 	TTCGTPGYMAPEIFKKTGHGKPV~DLWALGVITYFLLCGYTPFDRDSDFEEMQAILNADY
AN3065.1  	TTCGTPGYMAPEIFDKSGHGKPV2DIWAIGVITYFMLCGYTPFDRETNLEEVQAIATANF
          	**************.*:****** *:**:******:*********::::**:*** .*::

MG00925.1 	SFTPIEFWRGVSDSAKDFIRRCLTIDPTKRMSAHEALQHPFVAGYLA-------DKGANL
NCU02283.1	SFTPLEYWRGVSDNAKDFIRRCLTIDPAKRMTAHEALQHPFVAGWARGTDGAEADKGANL
FG00337.1 	SFTPIEYWRGVSAHAKDFIQRCLTIDSTKRITAHEALQHPFVAGFIN----AEGES-QNL
AN3065.1  	SFTPVEYWRGVSQEARDFIKRCLTVNPKKRMTAHQALQHPWINPPYDTT--DDLGSGEDL
          	****:*:*****  *:***:****::. **::**:*****::      :   .  .. :*

MG00925.1 	LPTVKKNFNARRTLHAAIDTVRAINKLREGQGLNGQRLMAGVKSREPNKAAAAA~AAAAS
NCU02283.1	LPTVKKNFNARRTLHAAIDTVRAINKLREGQ------FMNGGRSREPAKKAAAA1LGPTL
FG00337.1 	LPNIKKNFNARRTLHAAIDTVRAINKLREAQSG----LMDGARSKEPSRGAAQQ~T-PTN
AN3065.1  	LPNIKKNFNARRTLHKAIDTVRAINKLRENGGL----MMDGIMSVDPKPEHVNG~SEVVE
          	**.:*********** *************  .     :* *  * :*    .      . 

MG00925.1 	IP~TSTGEVGTVGAATHKDSGYGSTR--PDA1LR----------------------~---
NCU02283.1	GK~EAS-MVSTASSNVTKDSGYATQPEGEGG~SRDGDDVLMKDASVPAPTSSSPAP0CRV
FG00337.1 	RK0HPT-SHPRCNP-ATTEIELSKLPKVSGA~QR----------------------~---
AN3065.1  	DR~TTP-RERENEDAMEIDSRSNARGQTEQQ~IR----------------------~---
          	    ..            :       .      *                          

MG00925.1 	---PG~VETNKVVETSKGLWTGVGSRG----------------------
NCU02283.1	SYGRA~VSRIRLSRLVKACGMGLVLSDDQFLGLSSLTNGGWFCSIVIIF
FG00337.1 	---RA1V-RIR--------------------------------------
AN3065.1  	-----~EQERKVKETVAGLWSRTAPRSER--------------------
          	       .  :      .        ..
```
