# Supplementary material for: Patterns of Intron Gain and Loss in Fungi
Source: PLoS Biol. 2004 Nov 30;2(12):e422. doi: 10.1371/journal.pbio.0020422 (PMC532390; doi:10.1371/journal.pbio.0020422)
Supplement: Table S1 — Also available at http://genes.mit.edu/NielsenEtAl/. (4.3 MB ZIP). [file pbio.0020422.st001.zip › NielsenEtAl/html/1003.html]

AN0797.1.NCU03139.1.MG01309.1.FG00865.1


```
 CLUSTAL W (1.82) Multiple Sequence Alignments - Introns Inserted


Sequence 1: NCU03139.1	870 aa
Sequence 2: MG01309.1	865 aa
Sequence 3: FG00865.1	854 aa
Sequence 4: AN0797.1	867 aa
Alignment Length: 884 aa
Number Identitical Residues: 440 aa
Alignment Score (without introns) 20142


MG01309.1 	MESTLPLPFIVDASVN--LNG-EAGLSKEQLACLGTIFFEVTPQNLGDVRSFLQPGTSAF
NCU03139.1	METTLPLPFLVGVSVPPGLNDIKEGLSREEVSCLGCVFFEVKPQTLEKILRFLKRHNVEF
FG00865.1 	METTLPLPFLVSVASANDQPT-NDGLNRQEIALLGAPFYETSSKDWG-------RPSAGT
AN0797.1  	----MATPFLVSYDPA----SASGGLSLQQIAYFGRVLIKAT--DLAQAETFIRQNFRLL
          	    :. **:*.          . **. :::: :*  : :..     .            

MG01309.1 	EPYLDVTQLESANDILSLLDSGARKVFVKPEQLKDYEEHGSRVGQAVDG--TSLQVSAAE
NCU03139.1	EPYFDVTALESIDDIITLLDAGARKVFVKTEQLADLSAYGSRVAPIVTGSSAALLSSATE
FG00865.1 	NVHMDATGLSDPDDVVALLDSGVRTVFVISESYSEYEQYGARVIPAVSS---LILSSATE
AN0797.1  	DIYVDATGISATGDLVDILNAGAAKIFISLDQLNALSEEQSVPSSRLVVYTSSNDQVEAF
          	: :.*.* :.  .*:: :*::*. .:*:  :.    .   :     :   ::      : 

MG01309.1 	NGLLVSGIDASG-DVSTLVQQFNSKKGSP-------LFIRPADGADLELCAALARQVHAT
NCU03139.1	SGLLLSGFDQTASEAAQFLEEARDKKITP-------FFIKPVPGADLEQFIQVAAKANAI
FG00865.1 	HGLLVKDFDVSSSDVDKFIEVAQSKQIKS-------LYVKPTPETDIEKFIEVAKKANAI
AN0797.1  	QKWVVKHIEREEAGLCTDSAVVHSISVKLGLNPEAQLLYRTYSGDVTEDAVKDTMKQGGV
          	   ::. ::   :         .. . . . ...:.:  :.      *     : :  . 

MG01309.1 	VILPSSRLTACTKDATGGKVSISKLLASNWTSDRGDKLLPTVVTDDNGIALGLVYSSEES
NCU03139.1	PILPSTGLT--TKKDEAGKLAISTILSSVWKSDRPDGLLPTVVVDEHDTALGLVYSSAES
FG00865.1 	PIIPSTRLT--TDKNDSSRLLLSKLIASYWKSDRTDGLIPTVVTDDAGIALGLAYTSEES
AN0797.1  	SIVPAAALT-ISREESSGKIQAGSLIAARGVKDQGNGLYATTVTDERGTCLGFVWSSDES
          	 *:*:: **  : .  ..::  ..::::   .*: : * .*.*.*: . .**:.::* **

MG01309.1 	IGEALRTCTGVYQSRKRGLWYKGATSGDTQELVRISLDCDNDALKFVVRQKGR-~FCHLD
NCU03139.1	VNEALRTQTGVYQSRKRGLWYKGATSGDTQELVRISLDCDNDALKFVVKQKGR-~FCHLD
FG00865.1 	ILEALRTQTGVYQSRKRGLWVKGLTSGDTQELLRIGLDCDNDTIKFVVNQKGR-~FCHLQ
AN0797.1  	IAEALRTGTGVYQSRKRGLWYKGQSSGDVQELIRIGFDCDSDCLVFIVKQIGRG1FCHLG
          	: ***** ************ ** :***.***:**.:***.* : *:*.* **. **** 

MG01309.1 	QFSCFGNLGGIAKLEQTLTQRRESAPAGSYTARLFSDEKLLRAKIMEEAEELCDAKTKEN
NCU03139.1	QSGCFGQLKGLPKLEQTLISRKQSAPEGSYTARLFSDEKLVRAKIMEEAEELCTAQTPQE
FG00865.1 	QFGCFGDLNGISALEQTLKSRKESAPEGSYTARLFSDEKLLRAKIMEEAEELCDGKTKEN
AN0797.1  	TASCFGPYTGLSRLQKTLQARKADAPAGSYTARLFNEPKLTQAKIMEEADELCRAETKED
          	  .***   *:. *::**  *: .** ********.: ** :*******:*** .:* ::

MG01309.1 	VAFEAADLIYFALTKAVASGVSLSDIERNLDAKSWKVKRRTGDAKGKWAEKEGIKPSAPS
NCU03139.1	IAFEAADLFYFALTRAVAAGVTLADIERSLDAKSWKVKRRTGDAKGKWAEKEGIKP-AAS
FG00865.1 	IAFEAADLIYFALTKAVGAGVSLADIEANLDAKSLKVKRRTGNAKGKWAEKEGIKT--EE
AN0797.1  	IAFEAADLLYFALTRCVAAGVSLEDVERNLDLKSLKVKRRKGDAKGPWAEKAGLAE-KPA
          	:*******:*****:.*.:**:* *:* .** ** *****.*:*** **** *:      

MG01309.1 	ALAPAPAP----AATEATSDRIAMKVLDVSQSSVADIQEALKRPSQKSSDAIMKIIGPIV
NCU03139.1	ALAATSAPVTKEAAQETTPEKITMRRFDASKVSTEELDAALKRPAQKSSDAIYKIIVPII
FG00865.1 	TPAKAPQP----EAEKPADGRIAMERVDSTKISQADLVEKLKRPSQKSPDAILKIIKPII
AN0797.1  	EAKPAPKP----EEPKEDTSRIEMTRVATASTPAEKVQEYLKRPSQKSNDAIVGLVKPII
          	    :. *       :    :* *  .  :. .  .:   ****:*** ***  :: **:

MG01309.1 	DDVHTNGDKAVLSYTHKFEKATSLTSPVLKAPFPEEMMRLSPETAKAIDISFENIRKFHA
NCU03139.1	EDVRKNGDKAVLSYTHKFEKATSLTSPVLKAPFPKELMQLPEETIAAIDVSFENIRKFHA
FG00865.1 	EEVRTGGDKAVLSYTHKFEKATSLTSPVLKAPFPKELMDISPETIEAIDISFENIKKFHS
AN0797.1  	QDVREQGDAGVLKYTHKFEKATSLTSPVLKAPFPAELMKLSPEVQEAIDVSISNIARFHS
          	::*:  ** .**.********************* *:* :. *.  ***:*:.** :**:

MG01309.1 	AQK-EDKPLRVETMPGVVCSRFSRPIERVGLYVPGGTAVLPSTALMLGVPAMVAGCQRIV
NCU03139.1	AQK-EEKPLQVETMPGVVCSRFSRPIEAVGCYIPGGTAVLPSTALMLGVPAMVAGCNKIV
FG00865.1 	AQQ-EEKSLQVETMPGIVCSRFSRPIERVGLYIPGGTAVLPSTALMLGVPAMVAGCQKIV
AN0797.1  	AQKGSNDALSMETMPGVVCSRFSRPIERVGCYIPGGTAVLPSTAMMLGVPAMVAGCKKIV
          	**:..:..* :*****:********** ** *:***********:***********::**

MG01309.1 	LASPPRQDGTVTPEIVYVAHKVGAESIVLAGGAQAVAAMAYGTESVTKVDKILGPGNQFV
NCU03139.1	FASPPRADGTITPEIVYVAHKVGAESIVLAGGAQAVAAMAYGTESITKVDKILGPGNQFV
FG00865.1 	FASPPRSDGRITPEIVYVAHKVGAESIVLAGGAQAVAALAYGTESVTKVDKILGPGNQFV
AN0797.1  	FASPPRADGSITPEIVYVAHKVGAESIVLAGGAQAVAAMAYGTESVSKVDKILGPGNQFV
          	:***** ** :***************************:******::*************

MG01309.1 	TAAKMLVSNDTNAGVGIDMPAGPSEVLVIADCDANPAFVASDLLSQAEHGVDSQVVLIAV
NCU03139.1	TAAKMFVSNDTNAAVGIDMPAGPSEVLVIADKDANPAFVASDLLSQAEHGVDSQVILIAI
FG00865.1 	TAAKMHVSNDTNAGVGIDMPAGPSEVLVVADKDANPAFVASDLLSQAEHGVDSQVILIAV
AN0797.1  	TAAKMLVSNDTSAGVSIDMPAGPSEVLVIADKAANPAFVASDLLSQAEHGVDSQVILIAI
          	***** *****.*.*.************:**  **********************:***:

MG01309.1 	DLDEAGLKAIEDEVHRQAMALPRVDIVRGSIKHSITISVRNIEEAMRISNDYAPEHLILQ
NCU03139.1	DLDEEHLQAIEDEVHRQATELPRVQIVRGSIAHSITVQVKTVEEAMELSNKYAPEHLILQ
FG00865.1 	DLSEQELQAIEDEVHNQAVALPRVDIVRGSIAHSVTVQVKDITDAMRISNDYAPEHLILQ
AN0797.1  	DLNEQELKAIEDEVDRHARALPRMDIVRGSLAHSVTFVVRDLDEAMALSNDYAPEHLILQ
          	**.*  *:******..:*  ***::*****: **:*. *: : :** :**.*********

MG01309.1 	LKNAEAVVDMVMNAGSVFIGQWTPESVGDYSAGVNHSLP1TYGYAKQYSGVNLGSFVKHI
NCU03139.1	IKEAEKAVDLVMNAGSVFIGAWTPESVGDYSAGVNHSLP1TYGFAKQYSGVNLASFVKHI
FG00865.1 	IKDAEKAVDQVMNAGSVFIGHWTPESVGDYSAGVNHSLP1TYGFAKQYSGVNLGSFQKHI
AN0797.1  	IQNAEAAVEKVQNAGSVFIGQWTPESVGDYSAGVNHSLP1TYGYAKQYSGVNLGSFLKHI
          	:::** .*: * ******** ****************** ***:*********.** ***

MG01309.1 	TSSNLTADGLRNVGEAVMQLAKVEELEAHRRAVSIRMEYMNKQANQ
NCU03139.1	TSSNLTAEGLKNVGQAVMQLAKVEELEAHRRAVSIRLEHMSKSN--
FG00865.1 	TSSNLTADGLKNVGTAVMQLAKVEELEAHRRAVEIRLNYLKQQQ--
AN0797.1  	TSSNLTADGLLRLSKTVETLAAVEGLDAHKRAVSIRVAAMKQEQL-
          	*******:** .:. :*  ** ** *:**:***.**:  :.:.
```
