# Supplementary material for: Patterns of Intron Gain and Loss in Fungi
Source: PLoS Biol. 2004 Nov 30;2(12):e422. doi: 10.1371/journal.pbio.0020422 (PMC532390; doi:10.1371/journal.pbio.0020422)
Supplement: Table S1 — Also available at http://genes.mit.edu/NielsenEtAl/. (4.3 MB ZIP). [file pbio.0020422.st001.zip › NielsenEtAl/html/1004.html]

AN0261.1.NCU01318.1.MG06910.1.FG01917.1


```
 CLUSTAL W (1.82) Multiple Sequence Alignments - Introns Inserted


Sequence 1: NCU01318.1	775 aa
Sequence 2: FG01917.1	771 aa
Sequence 3: MG06910.1	763 aa
Sequence 4: AN0261.1	771 aa
Alignment Length: 777 aa
Number Identitical Residues: 588 aa
Alignment Score (without introns) 25646


MG06910.1 	MDYENLKEQWSEVEDRDGVRLSWNVFPSTRM0EASRLVVPIGALYTPLKEKPDTPLLQFE
NCU01318.1	MDYEALKEQWGEVEDRDGVRLSWNVFPSTRM0EASRLVVPIGALYTPLKEKPDTPLLQFD
FG01917.1 	MDYEAIKEQWSEVEDRDGVRLSWNVFPSSRM0EASRLVVPIGALYTPLKEKPDTPLLHFE
AN0261.1  	MDYEGLKDQWSDVEDRDGIRLSWNTFPSSRM0EASRLVVPIGAIYTPLKERPDAPLLQYE
          	**** :*:**.:******:*****.***:** ***********:******:**:***:::

MG06910.1 	PVTCKQPCRSVLNPFC2QVDVRARLWICPFCLSRNPLPPHYKDITANAIPPELHPSNTTI
NCU01318.1	PVSCKQPCRSVLNPYC2QVDVRARLWICPFCLSRNPLPPHYKDITANAIPPELHPSNTTI
FG01917.1 	PVTCKQPCRSVLNPFC2QVDVRARVWICPFCLSRNQLPPHYKDITANAIPPELHPANTTI
AN0261.1  	PVTCKAPCRAVLNPYA2NVDVRARIWICPFCLMRNPLPPHYKDITESTIPPELHPLSTTI
          	**:** ***:****:. :******:******* ** ********* .:******* .***

MG06910.1 	EYRLSRPAPSPPIFLYVVDTCQEDDSLNALKESLVMSLSLLPENALVGLITYGTM0TQVH
NCU01318.1	EYRLSRPAPAPPIFLYVVDTCQEEDSLAALKESLIMSLSLLPEHALVGLITYGTM~AQVH
FG01917.1 	EYRLSRPAPAPPIFLYVVDMCQEADSLASLKESLVMSLSLLPENALVGLITYGTM0AHVH
AN0261.1  	EYQLARPAPAPPIFVFVVDTCQEDDSLKAVKDSLILSLSLLPPNALVGLITFGTM0AQVH
          	**:*:****:****::*** *** *** ::*:**::****** :*******:*** ::**

MG06910.1 	EIGYTECAKSYVFRGSKDYAPKQVQEMLGLGQMPVRPGM--QPQ-PGRPMPMGPASRFLM
NCU01318.1	EIGYTECPKSYVFRGNKEYAAKQVQEMLGLVQPAMRPGM--PMQQPGRPFPAGPASRFLL
FG01917.1 	EIGYEECAKSYVFRGSKEYAAKQVQEMLGLSTSGVRPGM--QPQ-PGRPFPAGPASRFLL
AN0261.1  	ELGYTECAKSYVFRGSKDYNAKQVQEMLGLAS-GIRPNMPNMPQQPVRP-PLGAAARFLL
          	*:** **.*******.*:* .*********    :**.*..  *.* ** * *.*:***:

MG06910.1 	PVSQCEFQLTKALEQLQKDPWPVANDRRPLRCTGVALSVAVGLLESSFQNSGGRIMLFAA
NCU01318.1	PVSQAEFQLTKAIEQLQKDPWPVAGNLRALRCTGVALSVAVGLLETSFQNAGGRIMLFAG
FG01917.1 	PVQQAEFQLTKALESLQKDPWPVANDRRNLRCTGVALSVAVGLLESSFQNAGGRIMLFAG
AN0261.1  	PVQQAEFQITNMLEQLQRDPWPVANDKRPLRCTGVALNVAVGLLESSFQNAGAHIMLFTS
          	**.*.***:*: :*.**:******.: * ********.*******:****:*.:****:.

MG06910.1 	GPATEGPGMVVSSELREPMRSHHDIDRDNIKYYKKALK0FYDTLAKRTAHNGHIIDIFAG
NCU01318.1	GPATEGPGMVVGPELREPIRSHHDIDRDNIKYYKKALK0FYDNLAKRTAHNGHTIDIFAG
FG01917.1 	GPATEGPGMVVGPELREPIRSHHDIDRDNVKYYKKALK0FYENLAKRTAHNGHIIDIFAG
AN0261.1  	GPATEGPGLVVSPELKEPIRSHHDIDRDNIKYYKKALK0FYDALAKRAANNGHVVDLFAG
          	********:**..**:**:**********:******** **: ****:*:*** :*:***

MG06910.1 	CLDQVGLLEMKGLSNSTGGHMILVDSFTSSMFKQSFVRVFEKDGDDNLLMGFNGILEVLT
NCU01318.1	CLDQVGLLEMKGLCNSTGGHMILTDSFTSSMFKQSFVRIFEKDADDNLLMGFNAVLEVLT
FG01917.1 	CLDQVGLLEMKGLCNSTGGHMILTDSFTSSMFKQSFVRIFEKDGDDNLLMGFNAVLEVLT
AN0261.1  	CLDQVGLLEMKNLANYTGGHILLTDSFTSSQFKQSFIRVFDKDANDNLLMGFNASLEVLT
          	***********.*.* ****::*.****** *****:*:*:**.:********. *****

MG06910.1 	TKELKVTGLIGHAVSMNKKSTSVGETECGIGNTCSWKMCGIDPTSSYGIYFEVAQGG--P
NCU01318.1	TKELKVTGLIGHAVSLNKKSTSVGETECGIGNTCTWKMCGIDPSSSYGIYFEIANQGGPS
FG01917.1 	TKELKVTGLIGHAVSLNKKSISVGESECGIGNTCSWKMCGIDPKSSYGIYFEIAGQG-PA
AN0261.1  	TKELKVTGLIGHAVSLNKKSSSVGETECGIGNTCAWKMCGIDPSSSYGIYFEIANQGGPA
          	***************:**** ****:********:********.********:*  *...

MG06910.1 	SHAQPAQKGMMQFLTYYQHSSGQFHLRVTTIARNIGGPAGDPAIAQSFDQEAAAVLMSRI
NCU01318.1	QNMQSPQKGMMQFLTYYQHSSGHFHLRVTTIARNLSGPAGDPAIAQSFDQEAAAVLMSRI
FG01917.1 	THQQAPQKGMMQFLTYYQHSSGQFHLRVTTVARNLSSPAGDPAIAQSFDQEAAAVLMSRI
AN0261.1  	AVQPGPQRGMMQFLTYYQHSSGHFHLRVTTVARNLSGPAGDPTLAQSFDQEAAAVLMARI
          	     .*:**************:*******:***:..*****::*************:**

MG06910.1 	AVFKAEVDDGPDVLRWVDRMLISLCSRFADYRKDDPSSFRLEKNFTLYPQFMFHLRRSQF
NCU01318.1	AVFKAEVDDGPDVLRWVDRMLIRLCARFADYRKDDPSSFRLEKNFTLYPQFMFHLRRSQF
FG01917.1 	AVFKAEVDDGPDVLRWVDRMLIRLCSRFADYRKDDPSSFRLEKNFTLYPQFMFHLRRSQF
AN0261.1  	GVFKAEVDDGPDVLRWVDRMLIRLCSRFADYRKDDPTSFRLEKNFTLYPQFMFHLRRSQF
          	.********************* **:**********:***********************

MG06910.1 	LQVFNNSPDETAFYRHVLNHEDVSNSLIMIQPTLDTYTFDQEGGQPVLLDSASIQPTHIL
NCU01318.1	LQVFNNSPDETAFYRHVLNHEDVSNSLIMIQPTLDSYTFDQEGGVPVLLDSTSIQPTHIL
FG01917.1 	LQVFNNSPDETAFYRHVLNHEDVSNSLVMIQPTLDSYTFDQDGGQPVLLDSASIQPTHIL
AN0261.1  	LQVFNNSPDETAFYRHVLNHEDVGDSLIMIQPTLDSYSLEHEGSLPVLLDSASIQPTHIL
          	***********************.:**:*******:*:::::*. ******:********

MG06910.1 	LLDTFFHILIFHGETIAQWKKAGYQDQEGYENFAQLLQQPKEDAM0ELITDRFPLPRFIV
NCU01318.1	LLDTFFHILIFHGETIAEWRKAGYQDQEGYENFAALLEQPKEDAR0DLITDRFPLPRFIV
FG01917.1 	LLDTFFHILIFHGETIAEWRKAGYQEQEGYENFAGLLEQPKEDAR0DLITDRFPLPRFIV
AN0261.1  	LLDTFFHILIFHGETIAEWRKAGYQDQEGYENLKALLDLPKEDAR0ELISERFPLPRFIV
          	*****************:*:*****:******:  **: *****  :**::*********

MG06910.1 	CDAGGSQARFLLSKLNPSTTHTTGA--YGGVGAQTAQTIFTDDVSLQTFMDHLMK~----
NCU01318.1	CDQGGSQARFLLSKLNPSTTHTSGAGAYGGVGAQSAQTIFTDDVSLQTFMEHLMK~LAVS
FG01917.1 	CDAGGSQARFLLSKLNPSTTHTTGP--YGGVGATTAQTIFTDDVSLQTFMDHLMK2LAVS
AN0261.1  	CDAGGSQARFLLSKLNPSTTHTTGG--YGGG--VSSQTIFTDDVSLQTFMDHLMK2LAVS
          	** *******************:*   ***    ::**************:****  : :

MG06910.1 	---
NCU01318.1	GTN
FG01917.1 	GAN
AN0261.1  	GTS
          	.:.
```
