# Supplementary material for: Patterns of Intron Gain and Loss in Fungi
Source: PLoS Biol. 2004 Nov 30;2(12):e422. doi: 10.1371/journal.pbio.0020422 (PMC532390; doi:10.1371/journal.pbio.0020422)
Supplement: Table S1 — Also available at http://genes.mit.edu/NielsenEtAl/. (4.3 MB ZIP). [file pbio.0020422.st001.zip › NielsenEtAl/html/1005.html]

AN4255.1.NCU04728.1.MG00623.1.FG08399.1


```
 CLUSTAL W (1.82) Multiple Sequence Alignments - Introns Inserted


Sequence 1: NCU04728.1	565 aa
Sequence 2: MG00623.1	561 aa
Sequence 3: FG08399.1	520 aa
Sequence 4: AN4255.1	549 aa
Alignment Length: 585 aa
Number Identitical Residues: 130 aa
Alignment Score (without introns) 7781


MG00623.1 	-MTSLRDIIIAAFKSLTRGKSFIRALLAFWISPNVIKECQ--ESNDGSSSPRLLEDFLLE
NCU04728.1	-MASLKLLIINALKSLSRGRSFLQALLALWISP--KSNPN--KLKKIKVFRRTREEFLKE
FG08399.1 	MSTFQKKLLAAIIKSLLRGKSLVQSLLAYWSSSLGHDTSS--KPRVGNTPRRSIQEFLKE
AN4255.1  	-MSSPLVKVLHRMQALFSNIVAALETMLLFPSLFRDSSRGGGKSRAAFRRRRALDALADE
          	  :     :   :::*  .       :  : *    .   ..: .      *  : :  *

MG00623.1 	VEHLLSQPIEP------------AALLGFSGNLKKQFRQRLWSNRACMLPSYNYQLPNGS
NCU04728.1	AKKLLLGPIGSNHFIKNSGPTPDDPLLLLSKKLEAQFRQGLQTSPACMLPSYNHQLPGGH
FG08399.1 	AETLFLDPIGQ------------DSLQQLSRNLRKQLLQRLEADMECMLPSYSHQLPRGT
AN4255.1  	IEIIFSKPLTL------------KNMLAMSEKIREQFRAGLESSPINMLPSYNHALPTGL
          	 : ::  *:                :  :* ::. *:   * :.   *****.: ** * 

MG00623.1 	EQGRYLSVDVGGSTLRVALVELKGLEEGGYDGCSRIVRIDNFRIDVGVKALVGRAFFDWM
NCU04728.1	ESGQYLAVDIGGSTLRVGVVDLKGRQTTDDD-DSTIVHMDSYKIDRNVRLLRGAFFFKWM
FG08399.1 	EVGRFVALDVGGSTLRVALVELCGRMSNIGE-ESKIASMRNFRITPDIKALEGMAFFDWM
AN4255.1  	EQGTFLALDVGGSTMRVALIELCGQGK------MEVLRVSSSLIDNDVKLLEGTSFFDWM
          	* * ::::*:****:**.:::* *           :  : .  *  .:: * *  **.**

MG00623.1 	AARILETLKSASEGGQNNKEEFNGSPQAPIPMGLAWSFPIE2QTSLKGGLLQNMGKGFLA
NCU04728.1	ARKVETSLKKAIDAGHISKAEYELG--NPMPMGLAWSFPLE2QTSPNGGNICPMGKSFLA
FG08399.1 	AEKILETLSEELE--QDGRSD------GPLPMSMAWSFPIE2QTSLAGGKLQGMGKGFCA
AN4255.1  	AEKIEEMLREVGT--NYGREE------APLSMGLSWSFPIE~QTSISSGLVIHMGKGFRC
          	* ::   * .     : .: :       *:.*.::****:* ***  .* :  ***.* .

MG00623.1 	ADGLLGQDLGEIVKQACSGHNLHVEVAAIINDSSAALLSESYRRPSTRFGLILGTGFNIA
NCU04728.1	CDGLMGQDLGATCNKILQELRVNVEVVSIVNDSNATLLSSAYSSPSARFGLILGTGVNIA
FG08399.1 	CDGLLGWDLGDIVRTACLNRGLNVELRAIVNDSSACLLSESYNHPTTRFGLILGTGVNLA
AN4255.1  	SMGTVGQELGSLIVQSCQKRGLNVRVDAIVNDSSAALLSRAYVDPTTRMSLILGTGTNVA
          	. * :* :**           ::*.: :*:***.* *** :*  *::*:.****** *:*

MG00623.1 	AYMPVTTIERPKFGVRPASWFEKASHVIVNTEMGMFGQGFLPMTRWDEQLKAAHPKPEFQ
NCU04728.1	AHLPVNLIGKSKFGERPDSWYEQASHVMVNTELGMFGRDILPLTRWDKILKAGHERPDFQ
FG08399.1 	AYLPVSAIGRVKFGQRPPQWFEKATHVIINSEISMMGRDILPLTRWDRQLLANHARPEFQ
AN4255.1  	IHFPVHAIGLGKFGKRPQGWFDYAKHVIINSEMSMFGGGVLPMTRWDDILNRTHLRPDYQ
          	 ::**  *   *** **  *:: *.**::*:*:.*:* ..**:****  *   * :*::*

MG00623.1 	PLEHFVAGL~YIGELARLIMIDAIETTGLFGGVVPPSLVGPYTLETETLSILES2DDSVN
NCU04728.1	PLEHLVSGY~YLGEVFRIALLEAIKTTGLLGGKIPPLLLDAYSMDTGTMSLIIN2GRDAR
FG08399.1 	PLEHMVSGI2EF----------------------------DHSVDYDGTLMMNR~DTTSG
AN4255.1  	PLEYMATGR~YLGEIVRLIIVDAVETAQLFGGELPHSMRDAYSLDTSIVAFIEA~DTSPF
          	***::.:*   :..       .: .::   ..  .    . ::::     ::   .    

MG00623.1 	LENALAAFTSRHPSSQKPIVSDILALRTISKLVTRRSAALVAAALYGLWELKRETENEYL
NCU04728.1	GKAEAVFSQLYHLPAGYPSTEDMRFMQQLAGYIAQRSASIVAASVFALWELKNEAEQNVL
FG08399.1 	LEEARKQFSSCHPAKHAPTTADMELLKQLAGFISKRSSALVATGVHAFWNLRIESQNNFV
AN4255.1  	LTASAALLQKEHTMSRPPSPEDLRFLLRVCRTISKRAAGYLATAIHSMWCLRNEAEISQG
          	           *     *   *:  :  :.  :::*::. :*:.:..:* *: *:: .  

MG00623.1 	EPQETVKSTDMPFAQETRAELDLGRVVVGFNGSVIEQYPGYRTNCQIYIDALFKRSQEQL
NCU04728.1	NNLE----PESPFSEETALELMMEQTTVGYTGSVLECFPGYKETLQRYLDQLMMSSGHDL
FG08399.1 	QTMS----TESPERESAEADRDLAETTVAYNGGVIESYPGYLDSCQSYLDELVAGDKREK
AN4255.1  	PPSPS--FKGPRDVTVTESGSNSDCLSIACDGSVINKYPGFRDRCQAYLDQLTQETNTSK
          	    :           :          :.  *.*:: :**:    * *:* *      . 

MG00623.1 	SQGGGLSREEALIELVPAKESSLLGAAVALA-CLERGKAN--------
NCU04728.1	K-----GRR---IDLVPAKESSLLGAAVSLAGVVEEERMGLNEKKGIA
FG08399.1 	S-----GNR--TIKLVSAKESSLMGAAVALASLEEVVEGPLGVVG---
AN4255.1  	VS--QISEEGSCIRLEPAPEGAIFGAAVAVAVAVAGKLEQTIV-----
          	 .    ... : * * .* *.:::****::*
```
