# Supplementary material for: Patterns of Intron Gain and Loss in Fungi
Source: PLoS Biol. 2004 Nov 30;2(12):e422. doi: 10.1371/journal.pbio.0020422 (PMC532390; doi:10.1371/journal.pbio.0020422)
Supplement: Table S1 — Also available at http://genes.mit.edu/NielsenEtAl/. (4.3 MB ZIP). [file pbio.0020422.st001.zip › NielsenEtAl/html/1006.html]

AN4905.1.NCU04109.1.MG06747.1.FG02000.1


```
 CLUSTAL W (1.82) Multiple Sequence Alignments - Introns Inserted


Sequence 1: NCU04109.1	253 aa
Sequence 2: MG06747.1	252 aa
Sequence 3: FG02000.1	253 aa
Sequence 4: AN4905.1	250 aa
Alignment Length: 256 aa
Number Identitical Residues: 120 aa
Alignment Score (without introns) 6409


MG06747.1 	MG--IKTDIHLYTTGTPNGIKVSILLEELG~LEYQ~VTKIDIMKNVQKEQ~WFLDINPNG
NCU04109.1	MAS-QNSDIHLYTAQTPNGIKVSILLEELG~VPYK0VTAIDISKDVQKEP~WFLEINPNG
FG02000.1 	MAQKIKTDITLYTTNTPNGIKPSILLEELN~LEYK~VHAIKMTENEQKEE~WFLKINPNG
AN4905.1  	MS---RPDITLYTAQTPNGIKISIALEELG2IPYK~VEKIDISKNVQKEP2WFLEINPNG
          	*.   ..** ***: ****** ** ****. : *: *  *.: :: ***  ***.*****

MG06747.1 	RIPALTDTFTDGKTINLFESASIMQYLVDRYDTEHKVSYPHGSREHYQVQNWVYWQMGGL
NCU04109.1	RIPALTDKLEDGTPIALFESGAIMQYLVERYDKDHKVSYPQGSKEYYQTQSWLFWQMGGL
FG02000.1 	RIPALTDTLE-GKQIRVFESGAMLQYLVDRYDKDHKFSFPQGSAEHWEMTSWLMWQMGGL
AN4905.1  	RIPALTDTFTDGQKIRLFESGSILTYLAEQYDKDYKISYPRGTREYYETISWLYFQNAGV
          	*******.: .*  * :***.::: **.::**.::*.*:*:*: *:::  .*: :* .*:

MG06747.1 	GPMQGQANHFSR1YAPEKIQYGIDRYQNETHRLYGVMETQLASNPSGYLVGDKATIADFA
NCU04109.1	GPMQGQANHFTR~YAPEKIEYGINRYQNETRRLYRVMDAQLAKN--EYLVGDRPTIADFS
FG02000.1 	GPMQGQANHFKR1YAPEKIEYGINRYTNETRRLYRTLDTHLSKQESGYIVGDKVTIADIS
AN4905.1  	GPMQGQANHFSR~YAPERIEYGVNRYVNETRRLYGVLDKHLANSKSGYLVGDHITIADIS
          	**********.* ****:*:**::** ***:*** .:: :*:.. : *:***: ****::

MG06747.1 	CWGWVAGYS1WCGID--IEPFPHLKAWLWKLKERPGLDKGRNVPTPHTALDHVGKSQEEL
NCU04109.1	CWGWVAAHG1WCGIKNFEAQFPHLNAWLNRLLERPGLEKGRHVPSKHTALELNKLSEEEL
FG02000.1 	IWGWVAAAK1WAGVD--LSEFPHLEKWLYKLLERPGFEAGRHVPTPHTAFDLNKLSEAEL
AN4905.1  	HWGWVAAAG~WAGVD--IDEFPHLKAWEERLAAREGVEKGRHVPSPHTIKDLLK-DKKKA
          	 *****.   *.*:.     ****: *  :*  * *.: **:**: **  :    .: : 

MG06747.1 	DKMAEESRKWIQAGMAADAKK
NCU04109.1	EAKAVSSRAWVQKGMAEDAKK
FG02000.1 	EEKATGAKNWVQSGMKEDAKK
AN4905.1  	EEIAAQGRAWVQEGMKNDAAK
          	:  *  .: *:* **  ** *
```
