# Supplementary material for: Patterns of Intron Gain and Loss in Fungi
Source: PLoS Biol. 2004 Nov 30;2(12):e422. doi: 10.1371/journal.pbio.0020422 (PMC532390; doi:10.1371/journal.pbio.0020422)
Supplement: Table S1 — Also available at http://genes.mit.edu/NielsenEtAl/. (4.3 MB ZIP). [file pbio.0020422.st001.zip › NielsenEtAl/html/1007.html]

AN0930.1.NCU00589.1.MG10269.1.FG08690.1


```
 CLUSTAL W (1.82) Multiple Sequence Alignments - Introns Inserted


Sequence 1: NCU00589.1	579 aa
Sequence 2: MG10269.1	605 aa
Sequence 3: FG08690.1	723 aa
Sequence 4: AN0930.1	644 aa
Alignment Length: 768 aa
Number Identitical Residues: 249 aa
Alignment Score (without introns) 11354


MG10269.1 	----------------------------------------~-------------------
NCU00589.1	----------------------------------------~-------------------
FG08690.1 	MTACVAHHRGPTTPASEREASGNVPDSTMTKVGATPKCNR1GVWSAWLCKKLFLGESNRS
AN0930.1  	----------------------------------------~---------MMFRWDSRIG
          	                                                       .:  .

MG10269.1 	--~---------~------------------------~-----~-----~----------
NCU00589.1	--~---------~------------------------~-----~-----~----------
FG08690.1 	LL0LPHSGRWHI~AEAASGEDRWFSKELKARIEVTQQ0LKVIN~RELGI2WLFCAPDLNT
AN0930.1  	FA~DSISDTHEL2LQSTINYQGFFIVRFQSSHLFELT~LLPPS2DKLAP~---------L
          	    . :.      .:: . .       .:            .  . .            

MG10269.1 	--MASF-FTSPAS----FRVNNMFDNPALPP-ALMAQN----------------------
NCU00589.1	--MGLLNIRSPTS----LRTAVTTAGDSLNDFSISTTA----------------------
FG08690.1 	SRTKALSVASPRTRAQRYKHFIILRFAPILDFHLTTNNNSKQFNYLMASWQSFSVPSFRL
AN0930.1  	YPPSMAFFSSPRS----FQNAQSLTSRQFVAQTQMGVN----------------------
          	       . ** :     :         :                               

MG10269.1 	----------PFEIFKKKPHDSHPSLPHLVLLVFEAVMEVVCVALPGYIIARLGHFDAEK
NCU00589.1	----------PFTVFAPGEHDSHPSLPHIVLLVFGAVLEVVCVSLPGYIIARLGHFDAEK
FG08690.1 	TQLAQDAYELPLQTFAN-THDTHPSIGHLCLLVFEAVLEVVCVSLPGYIVARLGHFDAEK
AN0930.1  	---------PGILQDISSPHSNHPPFLHLVLLVFEAVLEVVCVSLPGYIAARVGMFDADA
          	           :       *..**.: *: **** **:*****:***** **:* ***: 

MG10269.1 	QKFLANLNIQLFTPCLI~FTKLASQLNADKLVDLGIIPVIFVIMTFVSYMVGLGVTKAFG
NCU00589.1	QKFLANLNVMLFTPCLI1FTKLASQLNADKLIELGVIPIIFVIQTFVSYMVSVGVAKCFG
FG08690.1 	QKFLANLNVMLFTPCLI1FTKLASQLTAEKLSDLAIIPVIFVIQTFVSWVVSVAVGKLFG
AN0930.1  	QKFVANLNVALFTPCLI1FTKLGSQLTAEKLTDLAIIPLIFIVQTAVSYSCAFVVSRCFR
          	***:****: ******* ****.***.*:** :*.:**:**:: * **:  .. * : * 

MG10269.1 	FGRRPANFVIAMG0VFGNSNSLPISLVISLSQTISGLHWDRIKGDNDDEVAARGILYLLV
NCU00589.1	FNKRASNFVTAMG~VFGNSNSLPISLVISLSQTLKGLHWDRIPGDNDDEVAARGILYLMV
FG08690.1 	FNRRASNFVTAMG~VFGNSNSLPISLVLSLSQTLKGLHWDKVPGDNDDEVGARGILYLLI
AN0930.1  	LKKRPSNFVAAMA0VFGNSNSLPISLVISLSQTLNGLHWDRIPNDNDDEVAARGILYLLI
          	: :*.:*** **. *************:*****:.*****:: .******.*******::

MG10269.1 	FQQLGQLVRWSWGYHVLLAPKDKYEEYNQEQAEAGRLRSGSVDGDSVSERRGLLENGSIH
NCU00589.1	FQQLGQLVRWSWGFHVLLAPKSKYDEYNNETIEEGRYRDEPDEDEEAAQLIQGLD--STH
FG08690.1 	FQQLGQLVRWSWGYHVLLAPKEKYPEYREEIAEEG-QRYHDDEDSETAALIDGLDG----
AN0930.1  	FQQLGQLVRWSWGYRVLLAPKERYIEEGERDNGETVVAQGRERYTDNPEQVDPDEP---L
          	*************::******.:* *  :.               . .      :     

MG10269.1 	ES-EPGVMNSDDEGTMHTD-SDSYVPAGRTPIAN--HSRASPADTDDDDSDGSRRPSSAP
NCU00589.1	EIGEESYAHGYRSPTMQSDNSEVYEPAGRTPVIG--SSRTSPSDSGEDDSDTIRKVNNDN
FG08690.1 	--------ETEDEGDRLSIDSQNYDPAGRTPIAS--ASRVSLAVSSDDDLPK-KSMPKGK
AN0930.1  	IRTRDSSDGSTEQATGSNDDSDVFHSGEATPVNTRSYSYTKLPHSGHEDQAHDQSPILGP
          	     .      .    . .*: : ..  **:   : * .. . :..:*    :      

MG10269.1 	GGRKSRKTSKHGNAGPRNHSIGPGPTNGHIPQPHENGDDLHIQSFPRIRNNDEQDMSGRV
NCU00589.1	SAVSDRDERPNG-----------------------------IMSFPRISHAHEREVP---
FG08690.1 	QGQTDIAAPLNGNAG-------------------------SMDSFPPVPSLEDDEEP---
AN0930.1  	PPSGPFLPRQDSRGD--------------------------ILYFPNVEVNPQDNTLE--
          	          .. ..                          :  ** :    : :     

MG10269.1 	KGLKTRVAMLKRKSTN----SIKAFGSRQFSRLPVTMQRGFRMLGRGLEKANVFLWEFMN
NCU00589.1	KGFPARVKALAKKAAVSVGASVTGVARTVFSALPAPLQTVLIKIYRGMGRFGAGLWEFMN
FG08690.1 	VGIAGRTKSAVKSPFIRLHKATSKTLCNWYQKSPAPVKSTLRFTKRAAGKFNNFIWEFMN
AN0930.1  	QGPLSRFKTSMGRLRVRIASGWARWTSALYSRLPPSLQKVLSKCSDSTSRFVRGVWDFMN
          	 *   *              .        :.  * .::  :     .  :    :*:***

MG10269.1 	PPLWAMLLAIVVASIPSLQRLFFEEGSFVRNSVTSAVSQTGGVAVPLILVVLGANLARNT
NCU00589.1	PPLWAMLFAVIVASIPDLQELFFKDDTFVKTSITSAINSSAGVAVPLILVVLGANLARNT
FG08690.1 	PPLWAMLIAILVASIPSLQRLFFEEGSFVQNSVTNAIRSSGNVAVPLILVVLGANLARNT
AN0930.1  	PPLWAMLVAIIVASVPSLQRLFFDDGTFIPNSVTRAINQNGQVAVPLILVVLGANLERNT
          	*******.*::***:*.**.***.:.:*: .*:* *: ... ************** ***

MG10269.1 	QKHDS-VDPEEREIGTKLLVASLMCRMLLPTIIMTPILAFFAKYVPISILDDPIFVIVCF
NCU00589.1	QKRDE-VDAEEKEIGTKLLVASLISRMLLPTLIMAPILALFAKYVPVSILDDPIFVIVCF
FG08690.1 	MAKDEALDPEEERIGNKLLVASLLCRMVLPTAIMAPMLALIAKYVNVSILDDPIFVIVCF
AN0930.1  	IPQEALEDTEDAETEKKLIIASLLARMLLPTIIMAPFLALIAKYAPVSIVDDPIFIIVCF
          	  ::   *.*: .  .**::***:.**:*** **:*:**::***. :**:*****:****

MG10269.1 	LLTGAPSALQLAQICQLNDVYEGVMGKILFQSYVVW~ILPSTLILVMLALEVVEAAK
NCU00589.1	LLTGAPSALQLAQICQINNVYEVVMGKILFQSYVIW2ILPSTLMLVMCALEVVEWAA
FG08690.1 	LLTGAPSALQLAQICQINNVFEKTMGRILFQSYVIW2ILPSTLILVMMALEVVEWAR
AN0930.1  	LLTGAPSALQLAQICQINNTYVGAMSKLLFQSYVVW2ILPSTLILVMCALEVVEWAA
          	****************:*:.:  .*.::******:* ******:*** ****** *
```
