# Supplementary material for: Patterns of Intron Gain and Loss in Fungi
Source: PLoS Biol. 2004 Nov 30;2(12):e422. doi: 10.1371/journal.pbio.0020422 (PMC532390; doi:10.1371/journal.pbio.0020422)
Supplement: Table S1 — Also available at http://genes.mit.edu/NielsenEtAl/. (4.3 MB ZIP). [file pbio.0020422.st001.zip › NielsenEtAl/html/1008.html]

AN6217.1.NCU06483.1.MG06372.1.FG05455.1


```
 CLUSTAL W (1.82) Multiple Sequence Alignments - Introns Inserted


Sequence 1: MG06372.1	728 aa
Sequence 2: FG05455.1	673 aa
Sequence 3: NCU06483.1	650 aa
Sequence 4: AN6217.1	618 aa
Alignment Length: 748 aa
Number Identitical Residues: 307 aa
Alignment Score (without introns) 15310


MG06372.1 	MFKNWSRPRGFVDPGPGNVLQKSSSQSSDPPQGGHWPVQQLAARKSSQG1ASPEATQSAA
NCU06483.1	-------------------------------------------------~----------
FG05455.1 	-------------------------------------------------~----------
AN6217.1  	-------------------------------------------------~----------
          	                                                            

MG06372.1 	PMASYHRPLSFSERKGSMFYGDELCLNTAGG-------VKLVQP-RPPRGPPTPSMSTPA
NCU06483.1	-MSPYGKHQSISERRGSVLG-EELSLNTHSA--------KLAQQHRPQP-PLTPLGMNPS
FG05455.1 	-MAGYQRSMSFSERRGSALS-DDLSLSTTTSPMINHRLEKLPEQQRHLRGPPTPSMSTPA
AN6217.1  	-MGSHSKAIPPSDK--------------------------------------------PC
          	 *. : :  . *::                                            *.

MG06372.1 	ADFDQTLTG-SPPPPPTPAASPGPSHSQLDWSNADVGEDFFLAKVRQHFQHSTGPQRTRI
NCU06483.1	ADFNQQLSAGSPPPPPTPAASPGPSQTQPDWSNAEEDESIVLSLMRENFLKATTPEKKRI
FG05455.1 	ADFDQQLTG-SPPPPPTPAASPGPSHFQPDWSDAADDEDFFLAKVRQHFKNCSGPQRTRV
AN6217.1  	TSIPPNLLPSAPASPPTPAPSPTPHQRLPSWRAPEQEEDALLLNT-KFFESLSGPRRQQY
          	:.:   *  .:*..*****.** * :   .*  .   *. .*    : *   : *.: : 

MG06372.1 	LADLLNLCTSQQLSFVHQFVSPLLKKDPFTSLPDELCLR0ILSFIDDPKVLARASQVSRR
NCU06483.1	LEDIVNMCNSQQLSFILQLVSPRLKKDPMMSLPDELCLR0VLSFIDDPKDLARASQVSRR
FG05455.1 	LADLLNLCTSQQLSFVHQFVSPLLKKDPFTSLPDELCLR0ILSFIDDPKVLARASQVSKR
AN6217.1  	LEAILSLCSSQQLSFVSSYVAPRLRKDPFRVFPTEISLR0VLSFVEDPKTLARASQVSKR
          	*  ::.:*.******: . *:* *:***:  :* *:.** :***::*** ********:*

MG06372.1 	WRDLLSDDMTWKNLCVKHDY-ERRLSEVQPPVGICLTRPAALPLSGSYSDSANRSFPGAL
NCU06483.1	WRDLVSDDMTWKNLCLKHDY-QRRLSEVQT--------------SMQYSNFLRPDPTSSF
FG05455.1 	WRDLLSDDMTWKNLCVKHDY-GRRLSEVYTHAPNFSPRPSAQPLHGLDADLANMNVSSTR
AN6217.1  	WHALLEDDITWKNLCEKHAFTHRRPAEDNQDIIESIHNHRTNSLSGLQRRPNSATIQSRD
          	*: *:.**:****** ** :: ** :*             : .              .  

MG06372.1 	PTPSVRMSASSSFDGASSS-----RPTLKTYKSHFKQRYLVDAAWRTGGRNVTRNITQEG
NCU06483.1	YQPYSAPAASVTFDDASSLR----RP--RSYKSHFKQRYLVDAAWRTGGRNITKNITQEG
FG05455.1 	PYS--YASGTRSFDGQSASG----RPRLRTYKSHFKQRYLVEAAWRSGGTSTTRNITQEG
AN6217.1  	GFADIPRSLSGDWIASSSIQTRKRRVRPLSYKYHFKQKYMIESAWSKGGRCTQRHISPDQ
          	  .    : :  :   *:  : . *    :** ****:*::::** .**    ::*: : 

MG06372.1 	GVVTSLHLTPKYIIVALDNAKIHVFDTEGNAQRTLQGHVMGVWAMVPWDDLLVSGGCDRD
NCU06483.1	GVVTSLHLTKKYIIVALDNTKIHVFDTEGNELRTLRGHMMGVWAMVPWDDVLVSGGCDRD
FG05455.1 	GVVTSLHLTPKYIIVALDNAKIHVFDTEGDSQRTLQGHVMGVWAMVPWDDTLVSGGCDRD
AN6217.1  	GVVTSLHLTPKYIVASLDNAKIHVYDTNGENQKTLEGHVMGVWAMVPWDNILVSGGCDRE
          	********* ***:.:***:****:**:*:  :**.**:**********: ********:

MG06372.1 	VRVWDLSTG2ACLHTLRGHTSTVRCLKMSDSTTAISGSRDTTLRIWDIRSGLCRNVLVGH
NCU06483.1	VRVWDLTTG2VCMHTLRGHTSTVRCLKMSDANTAISGSRDTTLRVWDIKNGVCRNVLVGH
FG05455.1 	VRVWNLKTG2ACLHTLRGHTSTVRCLKMADANTAISGSRDTTLRIWDIRTGLCKNVLVGH
AN6217.1  	VRVWNMATG~ESIYLLRGHTSTVRCLKMSDKNTAISGSRDTTLRIWDLRTGTCRSVLVGH
          	****:: **  .:: *************:* .************:**::.* *:.*****

MG06372.1 	QASVRCLEIKGDIVVSGSYDTTAKVWSISEGRCIHTLSGHYSQIYAIAFDGARVATGSLD
NCU06483.1	QASVRCLEIHGDIVVSGSYDTTAKVWSISEGRCLHTLSGHYSQIYAIAFDGYRVATGSLD
FG05455.1 	QSSVRCLEIKGDIVVSGSYDTFARVWSISEGRCLQTLQGHFSQIYAIAFDGKRVVTGSLD
AN6217.1  	QASVRCLAVHGDIVVSGSYDTTARVWSISEGRFLRALSGHFSQIYAIAFDGRRIATGSLD
          	*:***** ::*********** *:******** :::*.**:********** *:.*****

MG06372.1 	TSVRIWNAATG2ECQAVLQGHTSLVGQLQMRGETLVTGGSDGSVRVWSLSKFCPIHRLAA
NCU06483.1	TSVRIWNAATG2ECQAVLQGHTSLVGQLQMRGGTLVTGGSDGSVRVWSLERFCPIHRLAA
FG05455.1 	TNVRIWDPTSG2ECLAILQGHTSLVGQLQMRGDTLVTGGSDGSVRVWSLEKMCPIHRLAA
AN6217.1  	TSVRIWDPNTG~QCHAILQGHTSLVGQLQMSGDTLVTGGSDGSVRVWSLTRMAPIHRLAA
          	*.****:. :* :* *:************* * **************** ::.*******

MG06372.1 	HDNSVTSLQFDDTRVVSGGSDGRVKIWDLKTGHLVRELVAQCDAVWRVAFEDEKCIAMAS
NCU06483.1	HDNSVTSLQFDDTRIVSGGSDGRVKVWDLKTGQLVRELISQGEAVWRVAFEAEKCVAMAL
FG05455.1 	HDNSVTSLQFDDTRVVSGGSDGRVKIWDLKTGHLVRELIAQGEAVWRVAFEDEKCVALAL
AN6217.1  	HDNSVTSLQFDNNRIVSGGSDGRVKVWCLRTGQLLRELSTPSDTVWRVTFEEEKAVIMSS
          	***********:.*:**********:* *:**:*:*** :  ::****:** **.: :: 

MG06372.1 	RNGRTIME0LFSFSPP-DEMLYERPVSLPQRPHEAS-DRPMSALPLDYTKTNTLIPGLPR
NCU06483.1	RNSRTVME0VWSFSPP-EDVLLDR----HERQPFMLTPRPTIEAPVPERPSSAMDDSLIG
FG05455.1 	RQGRTVME0VWSFSPP-EEVLYDRPLTLQQRVLEDDPSRPLSAMAIDYRSSQQTLAGPSR
AN6217.1  	RSGRTVME0VWSFSPPPEDTEFDAA---TIGASSSISEPPPVRDSHPIRP--LVLPDLPQ
          	*..**:** ::*****.::   : .           .  *    .           .   

MG06372.1 	-RDCPDVDMRDAGPSTAPLQQCNSTFFHEED--
NCU06483.1	-TGMQDVDMPDAGPATAPLRP-NASFFQASRGA
FG05455.1 	DASAQDVDTHDAGPSTAPLQG--VTFFHDD---
AN6217.1  	-RSDGDQVMVDVPP-------------------
          	  .  *    *. *
```
