# Supplementary material for: Patterns of Intron Gain and Loss in Fungi
Source: PLoS Biol. 2004 Nov 30;2(12):e422. doi: 10.1371/journal.pbio.0020422 (PMC532390; doi:10.1371/journal.pbio.0020422)
Supplement: Table S1 — Also available at http://genes.mit.edu/NielsenEtAl/. (4.3 MB ZIP). [file pbio.0020422.st001.zip › NielsenEtAl/html/1009.html]

AN6629.1.NCU00634.1.MG02659.1.FG08561.1


```
 CLUSTAL W (1.82) Multiple Sequence Alignments - Introns Inserted


Sequence 1: NCU00634.1	142 aa
Sequence 2: FG08561.1	147 aa
Sequence 3: MG02659.1	146 aa
Sequence 4: AN6629.1	218 aa
Alignment Length: 219 aa
Number Identitical Residues: 62 aa
Alignment Score (without introns) 3205


MG02659.1 	------~-----------------------------------------------------
NCU00634.1	------~-----------------------------------------------------
FG08561.1 	------~-----------------------------------------------------
AN6629.1  	MGLNAS2PRPHRTEDVESCYWLVSESVGNTEIRDPVSRVRTCAFWTTSTTPTFDNNFHHP
          	 . .:: . .  :.. .:.    :.: ..:.  .. :   :.:  :::::.: ...   .

MG02659.1 	------~----MGDISIVAAQWPQVEVGRVLLINDG-PNAGKLATIVEIIDHKR0VLVDG
NCU00634.1	------~----MADIQIGSSAWRLVEVGRVLKLEG-----GSLATIVEIVDHKR0VLVDG
FG08561.1 	------~----MGDAVIEGSNWRLVEVGRVVVINGDHPFAGHLATIVEIIDHKR0ILVDG
AN6629.1  	SPELST0EAFAMADIDVKIAQWKLVEVGRVVLIRRG-PFTGKLATIVEIVDHKR0VLVDG
          	:.. :: .: :*.*  :  : *  ******: :. . . :* *******:**** :****

MG02659.1 	PSSDSNLAIARQSVPLSQAVLTGHVIPNLPRGARSGAVKAAWEKAEVDSKWKNSNWAKKA
NCU00634.1	PSSDPKLAAPRGVVSLSRTLLTPLVVEKLPRGARTGAVKKAWEAAGIDAKWKESNWAKKQ
FG08561.1 	PSANASLATPRQAVPLNKVLLSSLVVEGLNRGSRTGVVRKLWEKSEIDSKWEQTNWAKKR
AN6629.1  	PSTEEAKIVPRHVLPLSHATLTHFVIPKLPRAAGTGPVKKLWAQNEIDGKWAKSSIAQKA
          	**::     .*  :.*.:. *:  *:  * *.: :* *:  *    :*.** ::. *:* 

MG02659.1 	DRQVRRGALTDFDRFKVMRLKKR~ARFEERKALAKVKASA---
NCU00634.1	LQQERRKALTDFDRFKVMRLKKQ~RRFEERKALAKIKASA---
FG08561.1 	DQMERRKALTDFERFQVLRLKKQ~RRFEERKALAKVKASA---
AN6629.1  	DINNRRKNLTDFERFKVLRLRKQ0ARFEVQKAHAKIRAAAPKA
          	    **  ****:**:*:**:*:  *** :** **::*:*..:
```
