# Supplementary material for: Patterns of Intron Gain and Loss in Fungi
Source: PLoS Biol. 2004 Nov 30;2(12):e422. doi: 10.1371/journal.pbio.0020422 (PMC532390; doi:10.1371/journal.pbio.0020422)
Supplement: Table S1 — Also available at http://genes.mit.edu/NielsenEtAl/. (4.3 MB ZIP). [file pbio.0020422.st001.zip › NielsenEtAl/html/101.html]

AN2761.1.NCU02289.1.MG06562.1.FG10805.1


```
 CLUSTAL W (1.82) Multiple Sequence Alignments - Introns Inserted


Sequence 1: AN2761.1	147 aa
Sequence 2: FG10805.1	139 aa
Sequence 3: MG06562.1	154 aa
Sequence 4: NCU02289.1	147 aa
Alignment Length: 154 aa
Number Identitical Residues: 107 aa
Alignment Score (without introns) 4458


MG06562.1 	MALKRINKELTDLGR~YVINEHIARVLLRCIGGATRPV0FHWQATIMGP0SDSPYAGGVF
NCU02289.1	--MHSVS-AVVEYDA~DVLLSVAIRPP----LALLALR0FHWQATIMGP~ADSPYTGGVF
FG10805.1 	--------MLTLFHR~DPPSSCSA-------GPVGEDL0FHWQATIMGP0SDSPYSGGVF
AN2761.1  	MALKRINKELTDLGR2DPPSSCSA-------GPAGEDL0FHWQATIMGP0GDSPYSGGVF
          	 :    .. :.         .                  ********** .****:****

MG06562.1 	FLAIHFPTDYPFKPPKVNFTTRIYHPNINSNGSICLDILRDQWSPALTISKV1LLSICSM
NCU02289.1	FLNIQFPTDYPFKPPKVSFTTRIYHPNINSNGSICLDILRDQWSPALTISKV1LLSICSM
FG10805.1 	FLAIHFPTDYPFKPPKVNFTTRIYHPNINSNGSICLDILRDQWSPALTISKV1LLSICSM
AN2761.1  	FLTIHFPTDYPFKPPKVNFNTRIYHPNINSNGSICLDILRDQWSPALTISKV1LLSICSM
          	** *:************.*.******************************** *******

MG06562.1 	LTDPNPDDPLVPEIAHVYKTARAQYESTAREWTRKYAI
NCU02289.1	LTDPNPDDPLVPEIAHVYKTDRARYEATAREWTRKYAI
FG10805.1 	LTDPNPDDPLVPEIAHVYKTDRPRYEATAREWTRKYAI
AN2761.1  	LTDPNPDDPLVPEIAHVYKTDRPRYEATAREWTRKYAI
          	******************** *.:**:***********
```
