# Supplementary material for: Patterns of Intron Gain and Loss in Fungi
Source: PLoS Biol. 2004 Nov 30;2(12):e422. doi: 10.1371/journal.pbio.0020422 (PMC532390; doi:10.1371/journal.pbio.0020422)
Supplement: Table S1 — Also available at http://genes.mit.edu/NielsenEtAl/. (4.3 MB ZIP). [file pbio.0020422.st001.zip › NielsenEtAl/html/1010.html]

AN8874.1.NCU09808.1.MG06361.1.FG01320.1


```
 CLUSTAL W (1.82) Multiple Sequence Alignments - Introns Inserted


Sequence 1: MG06361.1	802 aa
Sequence 2: FG01320.1	770 aa
Sequence 3: NCU09808.1	801 aa
Sequence 4: AN8874.1	794 aa
Alignment Length: 840 aa
Number Identitical Residues: 465 aa
Alignment Score (without introns) 20451


MG06361.1 	MAALGDDLLSTVNKLQDLVFNTIGNDSLDLPQI0VVVGSQSAGKSSVLENIVGRDFLPRG
NCU09808.1	MAALGDDLLGVVNKLQDLVFNTIGNDSLDLPQI0VVVGSQSAGKSSVLENIVGRDFLPRG
FG01320.1 	--------------MTDRIFR------------~---------LSSVPS--------PR-
AN8874.1  	MAALGENLLVTVNKLQDLVFNTIGNDSLDLPQI0VVVGSQSSGKSSVLENIVGRDFLPRG
          	 :: ...     ..: * :*.: ...: . ..     .:.::. *** ..  . .  **.

MG06361.1 	SGIVTRRPLILQLINVPEED-----GAAVDVGYRSPDAARP~GEWAEFHHIPNRRFTDFG
NCU09808.1	SGIVTRRPLILQLINVPADDD---AEDATHPSYRNPNAAGR~NEWAEFHHIPNRRFTDFG
FG01320.1 	-------------ANLPSSK-----TSSVETFYR----VEP0--WAEFHHIPNRRFNDFG
AN8874.1  	SGIVTRRPLILQLINIPSERHDKPETDEVHVPHTAASVAGQ~NEWAEFHHQPGRKFDDFA
          	:.  :  .   .  *:* .  ...    ..  :  .. .   ..****** *.*:* **.

MG06361.1 	DVKREIENETARIAGTNKGINRQPINLKVYSPHVLNLTLVDLPGLTK0VPIGDQPGDIEK
NCU09808.1	DVKREIENETARVAGTNKGINRQPINLKIFSPHVLNLTLVDLPGLTK0VPIGDQPTDIEK
FG01320.1 	DVKREIENETSRVAGNNKGINRQPINLKIYSPHVLNLTLVDLPGLTK0VPIGDQPTDIEK
AN8874.1  	LVKQEIEAETARIAGNNKGINRQPINLKIFSPHVLNLTMVDLPGLTK~VPIGDQPSDIEK
          	 **:*** **:*:**.************::********:******** ******* ****

MG06361.1 	QTRTLISEYIAKPNSIILAVSPANVDIVNSEALKLARHVDGLGRRTIGVLTKVDLMDHGT
NCU09808.1	QTRNLISEYIAKPNSIVLAVSPANVDIVNSEALKLARHVDPMGRRTIGVLTKVDLMDHGT
FG01320.1 	QTRNLISEYIAKPNSIVLAVSPANVDIVNSEALKLARHVDPLGRRTIGVLTKVDLMDHGT
AN8874.1  	QTRALILEYIAKPNSIILAVSPANVDLVNSESLKLARQVDPMGRRTIGVLTKLDLMDHGT
          	*** ** *********:*********:****:*****:** :**********:*******

MG06361.1 	NALDILSGRVYPLKLGWVGVVNRSQQDIQGNKPMEDALKAESDFFKHHPAYRNISTRCGT
NCU09808.1	NALDILSGRVYPLKLGWIGVVNRSQQDIQGNKPMEEALKSESEFFRHHPAYRNISTRCGT
FG01320.1 	NALDILSGRVYPLKLGFIGVVNRSQQDIQGNKPMEDALQAETDFFKHHPAYRNISTRCGT
AN8874.1  	NAMDILSGRVYPLKLGFIGVVNRSQQDIQSGKSLSEALQAELDFFRHHPAYRNMANRCGT
          	**:*************::***********..*.:.:**::* :**:*******::.****

MG06361.1 	YFLAKTLNQTLMSHIRDRLPDIKARLNTLMGQTQQELASYGDMHFHGKEHRGSLILQLMT
NCU09808.1	QFLARTLNTTLMAHIRERLPDIKARLNTLMGQTQQELASYGDMHFSGKEHRGSLILQLMT
FG01320.1 	HYLAKTLNTTLMGHIRERLPDIKARLNTLMGQTQQELASYGDMHFSGKEHRGSLILQQMT
AN8874.1  	QFLAKTLNSTLMSHIRDRLPDIKARLNTLMGQTQQELASYGNKQFSGKEHRGSLILQLMT
          	 :**:*** ***.***:************************: :* *********** **

MG06361.1 	RFATSFISSIDGTSTEISTKELCGGARIYYIFNSVFGSSLESIDPTSNLTALDIRTAIRN
NCU09808.1	RFASSFIASIDGTSTEISTKELCGGARIYYIFNSVFGSSLESIDPTSNLTALDIRTAIRN
FG01320.1 	RFANSFISSIDGTSTEISTKELCGGARIYYIFNSVFGSALDTIDPTSNLSALDIRTAIRN
AN8874.1  	RFASSFISSIDGTSSEISTKELCGGARIYYIFNSVFGNSLDTIDPTQNLSISDIRTAIRN
          	***.***:******:**********************.:*::****.**:  ********

MG06361.1 	STGPRPSLFVPELAFDLLVKPQIKLLELPSQRCVELVYEELIKICHTCGSTELSRFPRLQ
NCU09808.1	STGPRPSLFVPEMAFDLLVKPQIKLLEPPSQRCVELVYEELIKICHTCGSTELSRFPRLQ
FG01320.1 	STGPRPSLFVPEMAFDLLVKPQIKLLEIPSHRCVELVYEELIKICHTCGSTELSRYPRLQ
AN8874.1  	STGPRPSLFVPELAFDLLVKPQIKLLEPPSQRCVELVYEELIKICHTCGSQELLRFPRLQ
          	************:************** **:******************* ** *:****

MG06361.1 	AKLIEVVSDLLRERLGPASTYVESLISIQRAYINTNHPNFLGATAAMSNVVSEKQERERK
NCU09808.1	AKLIEVVSDLLRERLGPASNYVESLISIQRAYINTNHPNFLGAAAAMSHVVSNKQERERK
FG01320.1 	AKLIETVSDLLRERLGPASSYVESLISIQRAYINTNHPNFLGAAAAMSNVVSAKQERERK
AN8874.1  	AKLIEVVSDLLRERLGPCSSYVESLISIQRAYINTNHPNFLGAAAAMSSIIQSKQDQERK
          	*****.***********.*.***********************:**** ::. **::***

MG06361.1 	KIILEERERREKRRLKELGN-GDNAEDQDDTASVAGGDSAGGTALRKQVA-KAGRSLSPG
NCU09808.1	RLIQEERERRERRRLKELGT--NATETPIEGEEESTTLEKTDNVHVRKTAGKGARSMSPA
FG01320.1 	RLIQEERERREKRRLQELEVEGDGPEGEDGVNGPSEKLEKTKSGRTRATK-QPHRSISPA
AN8874.1  	AALAEERRKREKRRIKELGGPNGNAPTHPEDE---EEQPETKNLPIRTQSSKGTRSMSPH
          	  : ***.:**:**::**   .. .                 .   :   .:  **:** 

MG06361.1 	--LRENGASGIASTLNGMG----RSASPARFNG-QGLGNAKDSFLTYFFGKDGP--GGLP
NCU09808.1	--VRENGPGSIASALNGAR-----SNSPSRFNGGQGVGNAKESFLNYFFGKDGAIVPGPS
FG01320.1 	ASVRENGTSSLAAHMNATHLNGLRSNSPARLNQ-QGLGGARDSFLNYFFGKDGQTIGGPL
AN8874.1  	--IGKISDTGITATLNGTH-----SNPPTAFGG---ANTTRDSFLNYFFGKDGA---QPQ
          	  : : .  .::: :*.       * .*: :.     . :::***.*******       

MG06361.1 	TSQPSAP--RHIAQNSHSALAPSSRRN-------------EDRMLRSPIQTYRDTEQEMG
NCU09808.1	NS-GNIG--RHVNQAMEPTFSQSMRRP-------------DDRQVRAPAQSMKVDDDMDY
FG01320.1 	PSPGAIPNGRHISQNSETSFPIIRREREFGRPSTATTVAPEDDMFDRTGKNYGLASQMVS
AN8874.1  	LTTSASPNQNQTRQSIHEVNVSSNARR---------------TEMLSPVDDYTTVPEYNE
          	 :     . .:  *  .        .                  .  . .      :   

MG06361.1 	LE----PG---GGDSFAQP~F0GHSGEPALTDREAMETELIRALISSYFNIVRESIADTV
NCU09808.1	VG-----------NAKGTE~L0SNDGEPAMTDREAMEAELIRALISSYFNIVRESIADQV
FG01320.1 	SEDIQVPGLLFSHQTNPHY~Q~GESAEPAMTEREAMETELIRALISSYFNIVRESIADQV
AN8874.1  	AS---------------SV0L~KDDSEPAISERELMETELIRRLISSYFTIVRETIADQV
          	                       ...***:::** **:**** ******.****:*** *

MG06361.1 	PKAIMHLLVNHCKDVVQNRLVSELYKEALFEELLYEDDGVVKEREKCEKLLQTYREAAKI
NCU09808.1	PKAIMHLLVNHCKDVVQNRLVSELYKETLFEELLYEDDAVKKEREKCEKLLQTYREAAKI
FG01320.1 	PKAVMHLLVNHCKDVVQNRLVSELYKESLFEELLYEDDGVKKEREKCERLLQTYREAAKI
AN8874.1  	PKAIMHLLVNHSKDVVQNRLVSELYKEEFFAELLYEDDGIKAEREKCERLLETYKAAAKI
          	***:*******.*************** :* *******.:  ******:**:**: ****

MG06361.1 	ISEVV
NCU09808.1	IGEVV
FG01320.1 	IGEVL
AN8874.1  	VGEVL
          	:.**:
```
