# Supplementary material for: Patterns of Intron Gain and Loss in Fungi
Source: PLoS Biol. 2004 Nov 30;2(12):e422. doi: 10.1371/journal.pbio.0020422 (PMC532390; doi:10.1371/journal.pbio.0020422)
Supplement: Table S1 — Also available at http://genes.mit.edu/NielsenEtAl/. (4.3 MB ZIP). [file pbio.0020422.st001.zip › NielsenEtAl/html/1011.html]

AN2266.1.NCU06183.1.MG06325.1.FG05469.1


```
 CLUSTAL W (1.82) Multiple Sequence Alignments - Introns Inserted


Sequence 1: NCU06183.1	963 aa
Sequence 2: MG06325.1	984 aa
Sequence 3: FG05469.1	964 aa
Sequence 4: AN2266.1	963 aa
Alignment Length: 1012 aa
Number Identitical Residues: 415 aa
Alignment Score (without introns) 23041


MG06325.1 	MALDPANGFVAGSSVANLNGDTAALLIFDVERVSLEFSIAADFVAAQVANNVLVLALSNG
NCU06183.1	MAFDPSNGFVASSDVPGLA--DELLPIFDVEQVQLQFSIAADFVSAQTANNVLILALSNG
FG05469.1 	MALNLQDGFDAANGVSDLE-----EPIFTIESVQLQFSVAADFVAAQVANNVIVLALSNG
AN2266.1  	MALDTSSGYAAHSSLIEPD----PLPMFDVRHVQLQFPLAADFVAAQVADNVLILALSTG
          	**::  .*: * ..:           :* :. *.*:*.:*****:**.*:**::****.*

MG06325.1 	RILRIDLNRPQDIDD~ID----------------------LPKKPSEVGVTRRMFLDPTA
NCU06183.1	RILRIDLNKPEDIDG~RQKHGSQRCGWTCFANKQTIQISTFPRSRPKLGVIRRMFLDPTA
FG05469.1 	RILRIDLERPEDIDD1ID----------------------LPKKPSEIGMIRRMFLDPTA
AN2266.1  	RILRIDLNNPEHIDD1VD----------------------LPKKSSETGVIRRMFLDPSA
          	*******:.*:.**.  :                      :*:. .: *: *******:*

MG06325.1 	SHLIICTTQGENYYLHSQSRQPRPLSRLRNVIIESVAWNPAQPNASTREILIGAADGNIY
NCU06183.1	SHLIICTSQGENYYLHSQSRHPRPLARLRGVSIESIAWNPSLPTASTREILIGASDGNVY
FG05469.1 	SHLIVCTTLGENYYLHSQSKHPRPLGRLRGVSIESVAWNPSLPTASTREILIGASDGNIY
AN2266.1  	SHLIITTTLGENYYLHTQSRHPKPLSRLKGLLIESVAWSPSLPTASTREILLGTTDGQVW
          	****: *: *******:**::*:**.**:.: ***:**.*: *.*******:*::**:::

MG06325.1 	ETFIETSTEFYKKEEKYFKHLQKLPDG-PVTGLWVDAVPGR-PDDKVVLITTQTRIFHLA
NCU06183.1	EGYIEHSTEFYRKEEKYLKVLHKLPDG-PVTGLWVDTLPGAGTDTRRILISTQSRLFHLV
FG05469.1 	EAFIETSKEFYKKEMKHLKNLHKLPDG-PITGLWVDNLQNNKSDLRRVVIATQTRLFHLV
AN2266.1  	ETYIEPSTEFYRREERYASSIYRTSDGSPVTGIWAETVPTK-AEQRRILVATHGKLSCFL
          	* :** *.***::* :: . : : .**:*:**:*.: :    .: : ::::*: ::  : 

MG06325.1 	GKAGRSHHHHHDGSTYTKLFESEEPTIHELPRPTSSSGINKASMQPASSLVVSPDPQQDP
NCU06183.1	GKVGK---NDGGGSIYAKLFEAEQPVVHELPRST-------AATAAASDLVISPDHPQDT
FG05469.1 	GRVGYG--HDGSGSIYTRLFESEQPVVHELSRTT---------SGAPSCLAVSPDAPD--
AN2266.1  	GRTGRSG-KEGAGPIYADLLSRETPVIHEIGQPS---------SSAPSNLVVSPSSSD--
          	*:.* .  :.  *. *: *:. * *.:**: :.:           ..* *.:**.  :  

MG06325.1 	AKPFQAPLPERVFAWLSSQGIFHGVLLNGPPGPELGTRVFSESKMLPRSQITSTLDFSGR
NCU06183.1	SRPHDGDVNERVFAWLSSHGVYHGQLLLSPFTSELGNKVFNEAQLLPRAQLMTPERVGGR
FG05469.1 	SGPYDDDVPDRAYAWLSYQGVFHGKLSNTPADSNLGTKVFSESKMLSRAQILSPEES--E
AN2266.1  	-AHHLDGHRDKEFAWLSSEGVYHGQLPYS---LDMLHKPFESSSMLPRSIFPATESARGG
          	   .     :: :**** .*::** *       ::  : *..:.:*.*: : :.    . 

MG06325.1 	KKTST-DAIEMIALTQWHVVCLVGRRVVAVNRLTGAIVYDQVILEMGQKAAGLCVDLQKN
NCU06183.1	RMTASNDYINAIALTHWHIISLIGDRVVAANRLTGDIVYDQVILNQGQKAIGLCVDIQKN
FG05469.1 	RRLATTEAIDAIALTQWHIVHLVGGRVITTNRLTGKMVSEHNVIGQGQKAIGFSVDMQKN
AN2266.1  	KKLIQ-NPLTAMTLSEWHILVLVEGRVLAVNRMNDEIVFDQEVLEPGQAALGLVTDAMKG
          	:     : :  ::*:.**:: *:  **::.**:.. :* :: ::  ** * *: .*  *.

MG06325.1 	TFWLFTAQEIFEIVVRDEDRDIWKIMLAAQRFDAALQYAHTPAQRNAVATASGDYLVAKG
NCU06183.1	TYWLFTSQEIFEIVPRDEDRDIWKIMLKLKKFDAALKHAHTPAQKDAVAIASGDYLLSKG
FG05469.1 	TFWLFTSEEIFEIVVRDEERNIWEIMTKLQQFEPALQHARTPLQKEIVAAAYGDHLASKG
AN2266.1  	TYWLFTSQDIFEICVQDEDRDVWKIFLRKQKFEEALRYARTSSQKDAVSTASGDFLASKG
          	*:****:::****  :**:*::*:*:   ::*: **::*:*. *:: *: * **.* :**

MG06325.1 	LFDEAAGVYGKSNKPFEEVALTFVDNNQPDALRKYLLSKLTTFKKGSVMQRVMIATWLVE
NCU06183.1	QYNEAAGVYGKSSKPFEEVALAFIDHNQPDALRKYLLGKLSTFKKSYIMQRQMIASWLIE
FG05469.1 	HWIEAATVYGRSNKPFEDIALSIIDNNQPDALRKFLLTKLASLKKPAVMQRMMIAGWLIE
AN2266.1  	RYIEAAGVWGKSSKAFEDVCLTLIKRGQHDALRKYLLSQLSVYKKSSSMQRTMVASWLIE
          	 : *** *:*:*.*.**::.*:::...* *****:** :*:  **   *** *:* **:*

MG06325.1 	IFMAKLNSLDDAIITKAELSEAMNPAETKERLDTVRGEYHDFVTKYKTDLDRKTVYDVIS
NCU06183.1	IFMAKLNSLDDTIITRAELSETLNPTQTREQLDVVRAEYQEFVNRHKSDLDRKTVYAIIG
FG05469.1 	VFMSKLNSLDDTINTQADPSENVNSTESRKLLESVRKEFRDFVDKYKGDLDRRMVYDVVS
AN2266.1  	VFMTKLNSLDDNITTKAELAEGSSTEEIEHELQAVRNEFQEFVTKYKSDLDQKTAYDIIS
          	:**:******* * *:*: :*  .. : .. *: ** *:::** ::* ***:: .* ::.

MG06325.1 	SHGREEELLFFANAVNDYNYVLSYWVQRENWTEALNVLKRQTDADVFYRYSSVLMTHVAT
NCU06183.1	SHGREEELLYYADAINDYHFVLSYWVQRERWSEALRVLQRQTDPEVFYSYSSVLMTHVAA
FG05469.1 	SHGREGELLYFANAVNDYNYVLSYWVQRERWSEVLNVLKKQTDPEVFYRYSSVLMTYVAP
AN2266.1  	SHGREKELLFFATVVNDHNYVLSYWIQRENWSEALNVLQRQSDPEVFYKHSSVLMTHAAT
          	***** ***::* .:**:::*****:***.*:*.*.**::*:*.:*** :******:.*.

MG06325.1 	DLVEILMRQSASLKPRNLIPALLEYDRNFKG-SLAQNQAIRYLQYVVNQLGSTDSAVHNT
NCU06183.1	ELVDILMRQ-ANLEPRNLIPALLEYDRNYKG-PLSQNQAIRYLLYVVNQLQSTDSAVHNT
FG05469.1 	ELVEILMRH-SDLKPRNLIPAFLEYNRTFTGGPNAQNQAIRYLNYAVYQLNSKDAAVHNT
AN2266.1  	GLVNILMRQ-TNLEPEKLIPALLNYNSTVSV-PLSQNQAVRYLNFIIVNHPRPTAAVHNT
          	 **:****: :.*:*.:****:*:*: . .  . :****:*** : : :     :*****

MG06325.1 	LVSMYASVPS-SKDETALLSYLESQ-GDEPRYDPDFALRLCIQHKRVLACVHIYTSLGQY
NCU06183.1	LVSIYAAHPSTSKDESALLSYLESQ-GDEPRFDPDFALRLCIQHHRVLSCAHIYTSMGQY
FG05469.1 	LVSIYASHPS--RDESGLLSYLQAQ-GDEPRYDPDFALRLCIQHHRTLSCVHIYTSMGQY
AN2266.1  	LISIHASSRS--SSEAGLLTYLQSQPSSPPPYDADFALRLCIQHQRFQSCIHIYSAMGQY
          	*:*::*:  *   .*:.**:**::*... * :*.**********:*  :* ***:::***

MG06325.1 	LQAVDLALAHGELELASIVADRPISNPTLRKRLWLAVARKVISQSDGGIKSAIEFLRRCD
NCU06183.1	LQAVQLALAHDEIDLAIIVAERAHSNPPLRKKLWLAVAKKVISQSNG-IKTAIDFLRRCD
FG05469.1 	LQAVDLALSHGEVELAAVIADRPMSNPQLRKRLWLAVARKVISQSNG-IKTAIEFLKRCD
AN2266.1  	LQAVELALQHDDIELAAIIADRPEGNNKLRKKLWLLVAEKKIRQSDTGIKDAIEFLRRCE
          	****:*** *.:::** ::*:*. .*  ***:*** **.* * **: .** **:**:**:

MG06325.1 	LLRIEDLIPFFPDFVVIDDFREEICTALEDYGRSIESLRREMEESSQTAANIRVDIAALD
NCU06183.1	LLKIEDLIPFFPDFVVIDDFKEEICAALEEYSRNIDSLRREMDESAATATNIKVDIAALD
FG05469.1 	LLKIEDLIPFFPDFVVIDDFKEEICAALEDYSRNIDNLKKEMDESSQTATNIKVDIAALD
AN2266.1  	LLRIEDLIPFFPDFVVIDDFKDEICSALEDYSRHIDALRQEMDSSAQTARQIRSEIAALD
          	**:*****************::***:***:*.* *: *::**:.*: ** :*: :*****

MG06325.1 	QRYAIVEPGEKCY~VCGLPLLSRQFFVFPCQHAFHSDCLGRKVMEQAGVGKGRRIKELQV
NCU06183.1	QRYAIVEPGEKCY0--------------------------------AGPGKAKRIKECQV
FG05469.1 	HRYAIVEPGEKCY~TCGLPLLSRQFFVFPCQHSFHSDCLGRKVLEQAGVGKSSRIRELQM
AN2266.1  	TRYAIVEPGEKCW~TCSLPVLSRQFFVFPCQHAFHSDCLGREVLEGAG-GKKKYIRDLQS
          	 ***********:  .. .  : .    ... :  :.. . .  . ** **   *:: * 

MG06325.1 	QISKGLVSGTKKAEMIAELDALVAAAC2ILCSEYAIKRIDEPFIRAEDEKSEWAL
NCU06183.1	QISRGLVKGRKREEMIGELDGLVGEAC2ILCSEYAIKRIDEPFVKENEDKEEWAL
FG05469.1 	QIQKGLVSGTQRETVVAELDALVASSC2ILCSDLAIKRIDEPFITHNDNVNEWIL
AN2266.1  	QLNEGALTSSQREEVVKELDGLIAEAC2ILCGDHAIKQIDKPFITATDNVDEWCL
          	*:..* :.. ::  :: ***.*:. :* ***.: ***:**:**:   :: .** *
```
