# Supplementary material for: Patterns of Intron Gain and Loss in Fungi
Source: PLoS Biol. 2004 Nov 30;2(12):e422. doi: 10.1371/journal.pbio.0020422 (PMC532390; doi:10.1371/journal.pbio.0020422)
Supplement: Table S1 — Also available at http://genes.mit.edu/NielsenEtAl/. (4.3 MB ZIP). [file pbio.0020422.st001.zip › NielsenEtAl/html/1012.html]

AN5520.1.NCU04779.1.MG04612.1.FG08478.1


```
 CLUSTAL W (1.82) Multiple Sequence Alignments - Introns Inserted


Sequence 1: MG04612.1	261 aa
Sequence 2: FG08478.1	262 aa
Sequence 3: NCU04779.1	262 aa
Sequence 4: AN5520.1	259 aa
Alignment Length: 264 aa
Number Identitical Residues: 182 aa
Alignment Score (without introns) 7801


MG04612.1 	M0PP-KTGK~KVAPAPFSAGKAS--KKAAK0NPLLEKRSRNFGIGQDIQPRRNLSRMVKW
NCU04779.1	M0PQ-KSGK~KAAPAPFPQGKAGS-KKAPK0NPLIEKRPRNFGIGQAIQPKRNLSRMVKW
FG08478.1 	M0PPNKSGK~KVAPAPFPQGKAG--KKAAK0NPLLEKRPRNYGIGQDIQPKRNVSRMVKW
AN5520.1  	-~----MGC0KAAPLPYPQGKAGSSKKGPK0NPLIEKRSRNFGIGQDIQPKRNLGRFVKW
          	       *  *.** *:. ***.::**..* ***:***.**:**** ***:**:.*:***

MG04612.1 	PEYVRLQRQKKILSMRLKVPPALAQFQHVLDKNTAAQAFKLLNKYRPETKAEKKERLLKE
NCU04779.1	PEYVRLQRQKKILNMRLKVPPALAQFQQVLDKNTAAQAFKLLNKYRPETKTEKKERLLQE
FG08478.1 	PEYVRLQRQKKILQMRLKVPPALAQFQHVLDRNTAAQAFKLLNKYRPETKAEKKERLLQE
AN5520.1  	PEYVRLQRQKKILNLRLKVPPSIAQFQNTLDRNTAAQTFKFLNKYRPETKVEKKERLHAE
          	*************.:******::****:.**:*****:**:*********.******  *

MG04612.1 	ATAVKEGKKKEDVSKKPYTAKYGLNHVVGLIENKKASLVLIPNDVDPVELVVFLPALCRK
NCU04779.1	ATAIKEGKKKEDVSKKPYVVKYGLNHVVGLIENKKASLVLIPNDVDPIELVVFLPALCRK
FG08478.1 	ATAVKEGKKKEDVSKKPYTVKYGLNHVVGLIENKKASLVLIPNDVEPIELVVFLPSLCKK
AN5520.1  	ATAVAEGKKKEDVSKKPYNVKYGLNHVVGLVENKKASLVLIAHDVDPIELVVFLPALCRK
          	***: ************* .**********:**********.:**:*:*******:**:*

MG04612.1 	MGVPYAIIKGKARLGTVVHKK0TAAVLAITEVRSEDKTELSKLISAIKDGYLEKSESARR
NCU04779.1	MGIPYAIIKGKARLGTVVHKK0TAAVLALTEVRAEDKNELAKLVSAIKEGYLEKNEQARR
FG08478.1 	MGIPYAIVKGKARLGTVVHKK0TAAVLAITEVRSEDKTELSKLISAVKDGYLEKHDQARR
AN5520.1  	MGVPYAIVKGKARLGTVVHKK0TSAVLAITEVRSEDKAEFAKLLSAIKEGYSDKTEESRR
          	**:****:************* *:****:****:*** *::**:**:*:** :* :.:**

MG04612.1 	TWGGGIMGYKAQQRELKKQKALDSAIKV
NCU04779.1	QWGGGIMGVKSQMKIAKRKKALENAIKV
FG08478.1 	QWGGGIMGAKAQMKIIKKQKALEAATKI
AN5520.1  	HWGGGIMGAKAVARQEKKRRAAEAAIRV
          	 ******* *:  :  *:::* : * ::
```
