# Supplementary material for: Patterns of Intron Gain and Loss in Fungi
Source: PLoS Biol. 2004 Nov 30;2(12):e422. doi: 10.1371/journal.pbio.0020422 (PMC532390; doi:10.1371/journal.pbio.0020422)
Supplement: Table S1 — Also available at http://genes.mit.edu/NielsenEtAl/. (4.3 MB ZIP). [file pbio.0020422.st001.zip › NielsenEtAl/html/1013.html]

AN1270.1.NCU07929.1.MG05156.1.FG05614.1


```
 CLUSTAL W (1.82) Multiple Sequence Alignments - Introns Inserted


Sequence 1: MG05156.1	427 aa
Sequence 2: FG05614.1	364 aa
Sequence 3: NCU07929.1	359 aa
Sequence 4: AN1270.1	364 aa
Alignment Length: 432 aa
Number Identitical Residues: 179 aa
Alignment Score (without introns) 8867


MG05156.1 	MADQSVTPAVES1EKSDVLPPKYSAPGLND~DDDDHPDPETAPEIAQIDTQSTVFSKDKQ
NCU07929.1	------------~--------------MAE~VQKDAP-----------------------
FG05614.1 	------------~--------------MAD~AQNDAP-----------------------
AN1270.1  	------------~--------------MAE2KEV-TP-----------------------
          	                           : :  :   *                       

MG05156.1 	PNRTATMADTFKESPIKSVQVEAL0VVMKIAKHCSSSFPTIATGSIVGMDNDTLVEVTNS
NCU07929.1	---------------IKSVQVEAL0VVMKIVKHCSTSFPTVATGSIVGMDNNGAIEVTNS
FG05614.1 	-----------ATAPFKAVQVEAL0VIMKIAKHCSSAFPSVATGAIVGMESEGLLEVTNT
AN1270.1  	---------------LTAVKVEAL0VVMKIIKHCSQVFPTTATGSIVGMDVDGVLEITNT
          	               :.:*:**** *:*** ****  **: ***:****: :  :*:**:

MG05156.1 	LNFPTVDVANVDSHQSERDASAQAAAAPRSKANLMYQAEMIKHLREVNVDANCVGWYTSA
NCU07929.1	FQFPSVDVSSSDSHSD---ASSLAAAAPRAKANIVYQNEMIRHLKEVNVDANNVGWYTSA
FG05614.1 	FPFPTVDPATTDGHQS--DASQLAAAAPRQQKNITYQNEMIRHLKEVNVDANNVGWYTSA
AN1270.1  	FPFPVVEVPPESHFDN--AAPNPAAAAPRAKANTVYQAEMIRMLREVNVDANNVGWYTSA
          	: ** *: .  . ...   *.  ****** : *  ** ***: *:******* *******

MG05156.1 	TMGNFVTMSFIENQAHYQRE-NPKAIALVHDVSRSSQASLSLRAFRLSTAFMTALKENKF
NCU07929.1	TMGNFINMSFIENQYHYQKE-NEKTVALVHDVSRSSQGALSLRAFKLSPEFMTAYKEAKF
FG05614.1 	TMGNFVNMNFVENQFHYQSA-NENAVALVYDASKSSQGNLTFRAFRLSPAFMSAYKEGKF
AN1270.1  	NMGNFVNMNVIENQFFYQKEMNERTVALVHDPSRSAQGSLSLRAFRLSPKFMAAFKDNKF
          	.****:.*..:*** .**   * .::***:* *:*:*. *::***:**. **:* *: **

MG05156.1 	TTEN2LQKTKLTYKDILIEMPVVIHNSHLLTTYLHQIPSAPAAGSETTIPTSLAALQREP
NCU07929.1	TTES2LRNSKLTYKDIFVELPVNVHNSHLLTSFLHQIP-APPKSAEIPMPASLDDIRRDP
FG05614.1 	TTEI2LQKSKLTFKDILAEVSVSVHNSHLLTTFLHQIPSAPVKG-AIEQPTSLDDLHRNA
AN1270.1  	TSDE2LQKSNLKYQDILVELPVEIHNSHLITSFIHQLQNQTQAT-PAEIPTSLATLESSP
          	*::  *::::*.::**: *:.* :*****:*:::**: . .        *:**  :. ..

MG05156.1 	VNIP--PYPSIDSLELSIDPFLEKTCDLLLDSIEAHYTDLNNHQYYQRQMTREQAKITAW
NCU07929.1	VQIP--AHPGFESLDLSIDPFLEKTCDLLLDSIESHYTDLNNHQYYQRQLTREQFKITQW
FG05614.1 	LEPP--LYPSIDNLDLAIDPFLEKTCDLLLESIESHYTDLNNFQYYQRQLGREQTKITQW
AN1270.1  	FAKQTILAPNFDNLSLSIDPFLEKNCDLLLDSIETHHTETSNFQYYQRSLAREQAKITAW
          	.   :   *.::.*.*:*******.*****:***:*:*: .*.*****.: *** *** *

MG05156.1 	QAKRKAENAARAAAKQEPLPDDEWKRLFKLPQEPSRLEGMLNARQVEQYSKQVDGFTANV
NCU07929.1	QAKRKAENAARLAAKQSPLPEDEWQRLFKLPQEPSRLEGMLNARQVDQYARQVDAFTANI
FG05614.1 	QAKRKAENAQRAAAKQEPLPEDEWQRLFKLPQEPSRLEGMLNAKQVEQYSKQVDGFTANV
AN1270.1  	QAKRKAENATRATLKQPPLPEDEWQRLFKLPQEPSRLDSMLNSRQVEQYARQIDSFVSST
          	********* * : ** ***:***:************:.***::**:**::*:*.*.:. 

MG05156.1 	SA~KMFAVRGSLLTE--
NCU07929.1	TA~KMFAVRGNLLPE--
FG05614.1 	SA~KMFAVREDLMPK--
AN1270.1  	TG1IRQRSSLSLSSSPE
          	:.        .* ....
```
