# Supplementary material for: Patterns of Intron Gain and Loss in Fungi
Source: PLoS Biol. 2004 Nov 30;2(12):e422. doi: 10.1371/journal.pbio.0020422 (PMC532390; doi:10.1371/journal.pbio.0020422)
Supplement: Table S1 — Also available at http://genes.mit.edu/NielsenEtAl/. (4.3 MB ZIP). [file pbio.0020422.st001.zip › NielsenEtAl/html/1014.html]

AN2256.1.NCU07975.1.MG06094.1.FG09828.1


```
 CLUSTAL W (1.82) Multiple Sequence Alignments - Introns Inserted


Sequence 1: NCU07975.1	950 aa
Sequence 2: FG09828.1	882 aa
Sequence 3: MG06094.1	879 aa
Sequence 4: AN2256.1	972 aa
Alignment Length: 994 aa
Number Identitical Residues: 289 aa
Alignment Score (without introns) 16004


MG06094.1 	---------------------------~----------MGPVGKRSHAAIIDLTGDDDST
NCU07975.1	-MPKRPYTSGVGAAASDRRDEARRRRL~NADSSSSGPSQSQYVPQSTS-TNGLSSSSQTS
FG09828.1 	---------------------------~----------MSKSTKRAHD-FVDLTSDDESE
AN2256.1  	MAERSKAPDSRKRFSYLNKVSGLRKRA2PGWYILIRLQLTRSPTKLEVPLQPKIMESRST
          	       ...    :     ..       .       .      :   .      .. : 

MG06094.1 	TP----SKHPRHGSSSSARKSRPGQPSSPKPLAASSQAPSSTQFYG--------------
NCU07975.1	SGRRQLPWQTPPGTQGAQSRHNTQGATSTYSARSSQATPSSQHRPPEHVQAIHDTLNWLS
FG09828.1 	TR------QKRPALNGPASQHQHQDRGS-----SSQNAPSSTAEP---------------
AN2256.1  	KR----SIHLVDYAPGEVHEGAPAPPPKYPRIAGQRFGQDTTFIPLS-----------QA
          	.     . :      .   .       .     ..    .:     .            :

MG06094.1 	--------VDDPNSRALNQSD------NSPQLELYGTLA1---------TGKYIPGK~HL
NCU07975.1	TQPDILESDDEAEVIDLTQAD------PGPVLEFYGHFD1GKIVGVRYYNGVASPGE~VV
FG09828.1 	------------DYLDLTQDD------ECPPLELYGTFH1GKIVGVRYYAGYASPGE1AV
AN2256.1  	SQVSLQEDEDDAAAADLLQDNQVVDDFSDTTQLHYGDLN~TKIVGVRYYRGTATIGE~HV
          	:. .  .. ...    * * :.  ..   .    ** :   .  .     *    *:  :

MG06094.1 	TTR-----K~YDVNAIRVDNVQGAQIGHFPRKIVEKLAPYI0DANDIAIEAKIMGEKQTF
NCU07975.1	VCKREPQNQ~YDPNAIRVDNVLGTQIGHIPRTVAAKLAPYM0DNGDLVVEGMLTGEKEFY
FG09828.1 	LCRREPNNS0YDRNAIRVDNVVGDQIGHLPRKVVEKIAPYV0DRGDIVLEAQLIGEKAYY
AN2256.1  	VLKREPHNQ~YDRNAIRVDNVMGTQIGHIPRNMAAKLATYM0DNRLLVVEGVLTGVIGAF
          	  : .. .. ** ******** * ****:**.:. *:*.*: *   :.:*. : *    :

MG06094.1 	DCPAKMFIFGTSDPLARAQLENRLKNDKL--------------LKATQLKQTKRESELRR
NCU07975.1	DCPVRLYFYGTSASLQRARLEERLKKDKL--------------VKATQLNQTRKANEEQR
FG09828.1 	DCPIKLFFYGSSDPQERSRIEESLKKDRL--------------VKAMELKNTRKEAEARR
AN2256.1  	DCPIVLKLYGPSDLERREALKQRMVQDKLPLNHLKKAERDEKKVQKEREKAVKEAAKRAR
          	***  : ::*.*    *  ::: : :*:*. .  ..:. ....::  . : .:.  :  *

MG06094.1 	--LPLEITGTSTQS-LKNMRPGQKNPLVAMESLIEQSQVVKARSTDDLVKSLAMDEEALS
NCU07975.1	KKQTLELRGNGTYG-FPSQTQEPE-PQVTMEQLAKMSEVISFRSGGDMIKSLAMSEEDLA
FG09828.1 	KAALGLVNGSSTHG-VGSELAVPQKPEITMDNVLQKSEAVEMRKGGDAIKSLAIGEDELE
AN2256.1  	KGQLLEAATNLGYSNLSQPFGEGIITEESLDELINQSSTFNPREINRVTESFGLKESDLE
          	.        .   ... .       .  :::.: : *.... *. .   :*:.: *. * 

MG06094.1 	ALPCADQPQELKSQLLPYQLQ~GLAWLVKKENPEFPVTGSDENTQLWKVDAKGRYRNLAT
NCU07975.1	NLPMASQPEKLRAKLLPYQLQ~GLAWMISKENPTMPAKGSTDSVQLWQHTADGRYHNMAT
FG09828.1 	KIPMAEQPEDLKAQLLPYQLQ0GLAWMTSKEKPQLPAEGSQDSVQLWLHQSKKKFFNVAS
AN2256.1  	NMPMVESPSSLSTTLLPYQRQ~GLAWMISKENPGLPTS-DNDVVQLWKKEGN-KFTNIAT
          	 :* ...*..* : ***** * ****: .**:* :*.  . : .***   .. :: *:*:

MG06094.1 	EFTTADAPKLLSGGILADDMGLGKTLQIIGLILTG-------GPGPTLIV1APMTVMSNW
NCU07975.1	GFYNKSPPQLLSGAICADDMGLGKTIQIISLIMTEGL-----GTGPTLIV~APVGVMSNW
FG09828.1 	GFVTSIAPKLLSGGILADDMGLGKTLQIISLILTG-------GKGPTLIV~APVSVMSNW
AN2256.1  	NFSTTAPPSLASGGILADDMGLGKTIQIISLILSNSQPKTKESSKATLII~SPVGIMSNW
          	 * .  .*.* **.* *********:***.**:: . ..:...  .***: :*: :****

MG06094.1 	SQQIENHVYEDERPSVYIHHGPSRLRDSEEVESYGVVITTYGTMTSEGSKGPLS------
NCU07975.1	KQQIRRHVHEEHQPKIVIYHGSKRKEFAKALQDQDVVITSYGTLS--DDALVK-------
FG09828.1 	SQQIKRHVRGDKQPSIITYHGSE-KATAKQLQGYDVVITSYGRLARERDQGVKRALTS--
AN2256.1  	RNQIQEHTNPEQAPRVLIYHGPGRKEDAN-LDHYDVVVTSYGTLATEYKTESKATPQKGL
          	 :**..*.  :. * :  :**.     :: ::  .**:*:** :: . .     :  .. 

MG06094.1 	-KIQWRRVVLDEGHT~IRNSDTLTALAACELKATSRWVLSGTPI~VNNIRDLYSLLKFLK
NCU07975.1	--TRWRRVVLDEGHS2IRNAKAQVAQNACKLEAKSRWVLTGTPI2INSIRDLHSLLKFLR
FG09828.1 	EDIKWRRVVLDEGHT~IRNSSTKVAQAACEINAESRWVLTGTPI2VNSVKDLHSLVKFLH
AN2256.1  	FSVKWRRVVLDEGHT~IRNPRSKGFSAACALRADSRWALTGTPI~VNTLKDLYSQIRFLG
          	 . :**********: ***. :     ** :.* ***.*:**** :*.::**:* ::** 

MG06094.1 	ITGGLESLEVFRSVIERGLSYGDSRAESLLQALMGDLCLRRNKSMKFVDLKLPPKTHYVH
NCU07975.1	ITGGIEQSEIFNTVLTRPLANGEPKGEALLKSLMKDLCIRRKKDMKFVDLKLPEKTEHMS
FG09828.1 	ITGGIEQSEIFNAQITRRLAVGDKTGEKLLQALMHDLCLRRKKDMKFVDLKLPAKKEYVH
AN2256.1  	LTGGLEDFAVFNSVLIRPLMSDDPDSRLLLQALMSTICLRRRKDMGFVNLRLPTLTSRVL
          	:***:*.  :*.: : * *  .:  .. **::**  :*:**.*.* **:*:**  .  : 

MG06094.1 	RIAFTEAEQKKYDALL~CEAKGVLNDIRKNPKTIQHGGFTSVLERLLRLRQM~CCHWTLC
NCU07975.1	RITFWPDEQKKYDALL2SEAQGVLENYRTQSKRSQ-GQFQGVLERLLRLRQT2CNHWVLC
FG09828.1 	RISFRKDEKRKYDALL2DEARGELEQWQASSQVGQKGRFQNVLERLLRLRQI2CNHWSLC
AN2256.1  	RIKFHPHEKEKYDMFQ2SEAKGMLLDFKSNNKTGT--TYSHLLEVILRLRQV~CNHWALA
          	** *   *:.*** :   **:* * : : . :      :  :** :*****  * ** *.

MG06094.1 	KERVKAVLSILEGQKVVELTPENRQILEEALRLLVESQDDCAVCLDTLDDPVITHCKHAF
NCU07975.1	KKRITEVLELLADKDVVDLTDENRAILQQALQLYIESQEECPICIDPLSNPIITHCKHVF
FG09828.1 	KERVSDILKLLDEHEVVPLNEKNRGLLQEALRLYIESQEECAICYDNPNDPVITTCKHVF
AN2256.1  	KNRLDKLAAILDKHQTVPLTPDNIKALQDMLQIRIESQEICPICLDILETPVITACAHAF
          	*:*:  :  :*  :..* *. .*   *:: *:: :***: *.:* *  . *:** * *.*

MG06094.1 	CRKCIMQVVEVQHRCPLCRTELSEDKLVEPAKEDNGRSVQVDDMDESAGSSKTDALLKIL
NCU07975.1	CRGCIDKVIEVQQKCPMCRAPLSEDKLLEPAPEHSAT-QDEEELESETKSSKTEAVLALV
FG09828.1 	CRGCIIRAIQIQHKCPMCRNKLDESSLLEPAPEDAG---DEEDFDAESQSSKTEAMMQIL
AN2256.1  	DHDCIEQVIVRQHKCPICRAEIENKSSLVAPAADLGENTDDVSADPDNPSSKIEALIKIL
          	 : ** :.:  *::**:**  :.:.. : ..  . . . :  . : .  *** :*:: ::

MG06094.1 	DGTLLKNSSSKVIIFSQWTSFLNVIQRQLEEHTTYGYTRIDGTMKPVARDDAMRKLETDP
NCU07975.1	KGTLDKEGS-KIIIFSQWTSFLTIIQHQLDEAG-YTYTRIDGSMNAAQRDAAIRALDYDP
FG09828.1 	KATMRKEGS-KVVVFSQWTSFLNIIEAQLKADG-MGYTRIDGSMKADKRDKAIEALDSDP
AN2256.1  	TAHGQVEAT-KTVIFSQWTSFLTLVEPHLQNAG-IQFARIDGKMTSIARDRSMRRFSTDP
          	 .    :.: * ::********.::: :*.      ::****.*..  ** ::. :. **

MG06094.1 	DTRILLASLGVCSVGLNLVTADTVILADSW~WAPAIEDQAIDRVHRLGQTRPTTVWRLVM
NCU07975.1	NTRILLASLGVCSVGLNLVSADTVILADSW~WAPAIEDQAVDRVHRLGQTRPTTVWRLVM
FG09828.1 	ETRVMLASLAVCSVGLNLVAADTVILSDSW1WAPAIEDQAIDRVHRLGQTRETTIFRLVM
AN2256.1  	KCTVLLASLSVCSVGLNLVAANQAILADSW~WAPAIEDQAVDRVYRLGQTRETTVWRLVM
          	.  ::****.*********:*: .**:*** *********:***:****** **::****

MG06094.1 	EDTVEERVLDVQSEKRDLVSKAFQEK-GKKTKAKETRMADIMKLLG
NCU07975.1	DNSIEERVLDIQKEKRELVGKAFQEKQDGKKKVKETRMADIMKLLS
FG09828.1 	EGSVEERVLDVQSEKRELVTKAFQEKNSRNKKRQNTRAADISKLLG
AN2256.1  	EDSIEDRVLAIQEQKRKLMLAAFREKASKKVDDRATRVADLEKLLT
          	:.::*:*** :*.:**.*:  **:** . : . : ** **: ***
```
