# Supplementary material for: Patterns of Intron Gain and Loss in Fungi
Source: PLoS Biol. 2004 Nov 30;2(12):e422. doi: 10.1371/journal.pbio.0020422 (PMC532390; doi:10.1371/journal.pbio.0020422)
Supplement: Table S1 — Also available at http://genes.mit.edu/NielsenEtAl/. (4.3 MB ZIP). [file pbio.0020422.st001.zip › NielsenEtAl/html/1015.html]

AN3933.1.NCU08616.1.MG01572.1.FG05627.1


```
 CLUSTAL W (1.82) Multiple Sequence Alignments - Introns Inserted


Sequence 1: NCU08616.1	1234 aa
Sequence 2: MG01572.1	1192 aa
Sequence 3: FG05627.1	1201 aa
Sequence 4: AN3933.1	1231 aa
Alignment Length: 1268 aa
Number Identitical Residues: 640 aa
Alignment Score (without introns) 32877


MG01572.1 	MAP---SATSTQWDHEYNTLRRENLFRNPPTDHSAYPALQTAVNPHIESFNALFRDDGKP
NCU08616.1	MAPQQPQPTSQDWDVEFNQVRREKLFRDPPTDRTAYPALQAAVDPHIESFNALFRDDGKP
FG05627.1 	MAP---TATETEWDHQFNTLRRENLFRNPPTDHSAYPALQLAVNPHIESFNAIFRDDGKP
AN3933.1  	MAP---AATDTNWSVNYDVLKRENLFRNPPQDKTAYPSLAESIKPHVDSFNALFE---KG
          	***    .*. :*. ::: ::**:***:** *::***:*  ::.**::****:*.   * 

MG01572.1 	GLIDHAIRDIGTKYFLDGDASMPSSE------------RNRLS0I---------------
NCU08616.1	SLLDHALAEIGTKTFLDGDERADPQG------------KNKLT~IRYKSIELQKSQVPPT
FG05627.1 	GLLTHAIADIGTRTFLDGDDRAPSDG------------KNILT~VRYKDVFLQKPQVPPS
AN3933.1  	GIIEAGLKDIGTRTFLDEVVETAEQKQQRLAEGRRAPRRNKLH~VRIKEVFLEKPTIPLT
          	.::  .: :***: ***       . ..  :..  :. :* *  :  ..   ...  . :

MG01572.1 	----------------------------------NNGELKECVRDIGQMPIMVK0SNRCH
NCU08616.1	NRW-AKNREIFPAECRERHVSYRGKLSATFEYRINDGEPHEFVRELGQMPIMVK0SNKCH
FG05627.1 	NKL-ARNRQVFPAECRERHVTYRGRLSATLEYRINGGDPVEFTREFGQVPVMIK0SNRCH
AN3933.1  	NKYTARNRNIYPSECRERHATYRGKLRAKIEYRVNNGDWTEQVRELGQVPIMLR0TNRCY
          	.  :: . .  .:.. .   :  .   :. .   *.*:  * .*::**:*:*:: :*:*:

MG01572.1 	LEGNSPALLVQRKEESEELGGYFIVNGNEKIIRLLLLNRRNFPLAINRPSFQNRGPAYTP
NCU08616.1	LQNNSPAQLVARKEESEELGGYFIVNGIEKLIRMLLVNRRNFPLAIVRPSFQNRGASYTP
FG05627.1 	LEGNSPALLVERKEESEELGGYFVVNGIEKIIRMLQLNKRNFPMAINRPSFQNRGPGYTP
AN3933.1  	LEKATPAELVQHKEESEELGGYFIVNGNEKLIRMLIVGRRNYPMAIVRNTFTNRGHAYSK
          	*:  :** ** :***********:*** **:**:* :.:**:*:** * :* *** .*: 

MG01572.1 	YGIILRSVRPDETSQTNVLHYLNDGNVTFRFSWRKNEYLVPVMMILKALVETNDSEIFEG
NCU08616.1	YGIIMRSVRPDETSQTNVLHYLSDGNVTFRFSWRKNEYLIPVMMIMKALVETNDREIFEG
FG05627.1 	YGIILRAVRPDETSQTNVLHYLNDGNMTFRFSWRKNEYLVPVMMILKALVETNDREIFEG
AN3933.1  	FGIQIRSVRPDQTSQTNVLHYLNDGNVTFRFSWRKNEYIIPVVMILKALVETNDREIFEG
          	:** :*:****:**********.***:***********::**:**:******** *****

MG01572.1 	LCGPPGSKGVGNSFLTDRVELLLRTYKSYGLYSKTKTRAYLGEKFRVVLGVQDSMSHYDV
NCU08616.1	LVGPPQSKGVANTFLTDRVELLLRTYKKYGLYSKTQTRAYLGQKFRVVLGVPDTMSDYEV
FG05627.1 	LVGPAGSKAAGNTFLTDRIELLLRTYKSYNLYSKSDTRAFLGEKFRVVLGVPDTMTNYEV
AN3933.1  	IVGSASSEGINNTFVTDRVELLLRTYKAYKLHSRSECRAFIGEKFKPVLGVPADTPNEEA
          	: *.. *:.  *:*:***:******** * *:*::. **::*:**: ****    .. :.

MG01572.1 	GTEFLKKIVLVHLGSHDVTEQEDADKFRMILVMMRKLYALVAGECAVDNPDAVQNQEILL
NCU08616.1	GTEFLRKIVLVHLGSQDVTEQQDADKFNMLLFMCRKLYALVAGDCAVDNPDAVQNQEILL
FG05627.1 	GTEFLRKIVLVHLGNVDVTEEQDNEKYKLILFMIRKLYALVAGECAVDNPDAVQNQEILL
AN3933.1  	GTEFLRKVVLPHLGNQNVTETQDYDKFKMLMFMIRKLYALVAGDCAPDNPDAVSNQEILL
          	*****:*:** ***. :*** :* :*:.:::.* *********:** ******.******

MG01572.1 	GGYLYGMILKERLEEFVAVSLRAALRDYLRRSPANTFTSAAFEKDFPANIFRRTNENLGN
NCU08616.1	GGFLYGQIIKERLEELLTVSFRASLRDYLRRNPTVSFQSDTFLKDFPIAIFRRANENIGQ
FG05627.1 	GGFLYGQILKERFDEFLSVNVRGSMRDYFRRNPGIPFTSEEFRKEFPNNIFRKANENLGN
AN3933.1  	GGFLYGMLLKERLDEWVRS-FGPILRDWSNRNHGARFTDPAFERDFVSKVIRRSNENIGG
          	**:*** ::***::* :   .   :**: .*.    * .  * ::*   ::*::***:* 

MG01572.1 	SLEYFLSTGNLQSPSGLDLQQTSGFVVVAEKLNFLRFISHFRMVHRGSFFAQLKTTTVRK
NCU08616.1	SLEYFLSTGNLVSPSGLDLQQVSGFTVVAEKLNFLRFISHFRMVHRGSFFAQLKTTTVRK
FG05627.1 	ALEYFMSTGNLQSQSGLDLQQTAGFTVVAEKLNFTRFISHFRMVHRGAFFAQLKTTAVRK
AN3933.1  	AMEYFLSTGNLVSPTGLDLQQTSGYTVMAEKINFYRFISHFRMIHRGSFFAQLKTTTVRK
          	::***:***** * :******.:*:.*:***:** ********:***:********:***

MG01572.1 	LLPESWGFLCPVHTPDGSPCGLLNHLAHKCKIMTDAVDVSAIPRLATELGVVDTSSADTE
NCU08616.1	LLPESWGFLCPVHTPDGSPCGLLNHLAHKCKIMTESVDASTISRLAFELGVVNISSAATS
FG05627.1 	LLPESWGFMCPVHTPDGSPCGLLNHLARKCSITTDYLDVSHIATLAAELGVVDVSSASTD
AN3933.1  	LLPESWGFLCPVHTPDGSPCGLLNHLAHKCLVATSDTDVSHLPKLLVQLGVRNESSVSLD
          	********:******************:** : *.  *.* :. *  :*** : **.  .

MG01572.1 	ESVPVMLDGKILGWCSPKQSRTIADTLRYWKVEGSHGVPVHMEIGLVPPSNGGSYPGIYM
NCU08616.1	ESVVVMLDGRIVGWCTPEECKSIAETLRYWKVNGENGVPLQLEIGYVPPSNGGSYPGLYM
FG05627.1 	ENVVVMMDGKILGYCTPKESVRIADCFRYWKVEGTHGVPLQLEIGYVPPSRGGSYPGVYL
AN3933.1  	ESVTVQLDGRIIGYCSPKQARVIASTLRHWKVSGTNNVPLGLEIGYVPNSNGGQYPGIYM
          	*.* * :**:*:*:*:*::.  **. :*:***.* :.**: :*** ** *.**.***:*:

MG01572.1 	ASQPSRMVRPVKYLPLEKEDYVGTHEQPYMSIACTEPEIVSGESTHVEFDPTNMLSILAN
NCU08616.1	SSQPARMVRPVKYLPLQKEDFVGPQEQPYMSIACTEQEVIPGDSTHVEFDPTNILSILAN
FG05627.1 	TSTPARMVRPVKYLPLQKEDWVGPYEQPYMSIAVVPQEIESGKSTHVEFDPTNILSILAN
AN3933.1  	FSQAARMYRPVKYLPLDKLDYVGPFEQPFMEIACLPSDLVKGLSTHIEFTPTNILSIVAN
          	 * .:** ********:* *:**. ***:*.**    ::  * ***:** ***:***:**

MG01572.1 	MTPFSDFNQSP~RNMYQCQMGKQTMGTPGAAISRRTD~NKMYRIQTGQTPIVRAPLHNEY
NCU08616.1	MTPFSDFNQSP~RNMYQCQMGKQTMGTPATALAHRTD~NKMYRLQTGQTPVVRAPLHNTY
FG05627.1 	MTPFSDFNQSP0-----CQMAKQTMGTPGTASVYRTD1NKSYQIQTGQTPIVRAPLHNTY
AN3933.1  	MTPFSDYNQSP~RNMYQCQMSKQTMGTPGTAIDYRTD~NKLYRLQTGQTPIVRPPLYNAY
          	******:****  .  .***.*******.:*   *** ** *::******:**.**:* *

MG01572.1 	GFDNFPNGMNAVVAVISYTGYDMDDAMIINKSAHERGFGHGTIYKTKKITL--KDDSRTR
NCU08616.1	GFDNFPNGMNAVVAVISYTGYDMDDAMILNKSAHERGFGHGSIYKTKKVSL--KDDSRTR
FG05627.1 	GFDNFPNGFNAVVAVISYTGYDMDDAMILNKSAHERGFGHGTIYKTKKISL--KDDSRTK
AN3933.1  	GLDNFPNGTNAVVAIISYTGYDMDDAMIINKSSHERGFGYGTVYKTKVHSLDEKDSRRTK
          	*:****** *****:*************:***:******:*::****  :*..**. **:

MG01572.1 	STKHITKMFGFAPNTTVKARDLEKLDLDGLPRIGSKVEQGDIIAAWHTVSADYS-DQLVN
NCU08616.1	SAKSIVKMFGFAPNSTIRESTRDMLDNDGLPRVGRLLREGDVICAWHTVSADYN-GQLVN
FG05627.1 	ATKSVTKAFGFAPHSHVSAHHQGMLDDDGLPHVGRLIQEGDVICAWHTVTPDYN-GKLVN
AN3933.1  	SKQAVQKLFGFAPGSEIRAEWRATLDEDGFPHIGAEIKEGSIVAAYHTVRYDATSDSYIN
          	: : : * ***** : :       ** **:*::*  :.:*.::.*:***  * .:.. :*

MG01572.1 	RDGLTHYEKYKDSETAFVEEIRLVGNENGTEPLQTVSIKFRIPRSPVIGDKFSSRHGQKG
NCU08616.1	RDGVTHYERYKDSEDAFVEEVRVIGADNGTEPLQTVSIKLRIPRSPVIGDKFSSRHGQKG
FG05627.1 	LDGITHYEKYKDSEEGFIETVRLIGAESGNEPLQTVSIKFRIPRSPIVGDKFSSRHGQKG
AN3933.1  	VDGITHFVKYKDSERAYIDSIRIMGSETGTEPAQAISVKYRIPRKPIIGDKFSSRHGQKG
          	 **:**: :***** .::: :*::* :.*.** *::*:* ****.*::************

MG01572.1 	VASQKWPMIDMPFSESGMQPDVIINPHAFPSR~MTIGMFVESLA~GKSGALHGLAQDSTP
NCU08616.1	VLSQKWPATDMPFSETGIQPDVIINPHAFPSR~MTIGMFVESLA~GKAGALHGLAQDSTP
FG05627.1 	VASQKWPTMDLPFSET----------------~----------A1GKAGALHGLAQDSTP
AN3933.1  	VCSQLWPAVDMPFSESGIQPDLIINPHAFPSR1MTIAQMIESMA~GKAGALHGHPQDCTP
          	* ** **  *:****:. ...   .. : .:   : .   .: * **:***** .**.**

MG01572.1 	FKFDEQNTAADYFGHQLMKAGYNYHGNEPMYSGITGQELAADIYIGVVYYQRLRHMVNDK
NCU08616.1	FKFDEQNTAGDYFGHQLMKAGYNYHGNEPLYSGITGEEFQADIYIGVVYYQRLRHMVNDK
FG05627.1 	FKFDEENTAGDYFGHQLMKAGYNYHGNEPMYSGITGEELQADIYIGVVYYQRLRHMVNDK
AN3933.1  	FQFSEEYTATDYFGEQLRRAGYNYYGNEPLYSGITGKEFAADIFIGVVHYQRLRHMVNDK
          	*:*.*: ** ****.** :*****:****:******:*: ***:****:***********

MG01572.1 	FQVRTTGPVVPTTGQPIKGRKRGGGIRVGEMERDALLAHGTAFLLQDRLLNCSDYTRSWI
NCU08616.1	YQVRTTGPVVPTTGQPIKGRKKGGGIRVGEMERDALLAHGTSFLLQDRLLNCSDYSKSWM
FG05627.1 	YQVRTTGPVVPTTGQPIKGRKRGGGIRVGEMERDALLAHGTAFLLQDRLLNCSDYSKSWI
AN3933.1  	FQVRTTGPVNNLTGQPVKGRAKGGGIRVGEMERDSLIAHGAAYILQDRLMNCSDSQRAWI
          	:********   ****:*** :************:*:***::::*****:****  ::*:

MG01572.1 	CRTCGTFLSVQPTVSPFV-GKRK-VNTVRCRSCAQ-------RLDRLDEDVDLATLEGEI
NCU08616.1	CRQCGSFLSTQPTVSPFI-GKRKAVSTVRCRNCAV-------RLDDM-EDVDLMQIDGEI
FG05627.1 	CRRCGSFLSVQPTVSQFAPGKKKAPSIVRCRACAI-------KLDDA-DAVDLTEVQGEI
AN3933.1  	CRDCGSFLSTQVAVSSAGSSKARMAAKNSSGSAALGGNAGIVRCRRCAREAVFDDSRAVV
          	** **:***.* :**   ..* :      .  .* ...:.  :       . :    . :

MG01572.1 	WEDGQGNQWIGGDNTTVVVVPGALKFLDVELAAMGVKLKYRVDPKDAPRKGPTKRAGVDG
NCU08616.1	WEDGSGTQWIGGENTTIVAVPGALKYLDVELAAMGIKLKYKVDKKDEIRRG-----QLVG
FG05627.1 	WEDGLGSSWVGGDQTTQVVVPGALKYLDVELAAMGVKLKYRVDRNDEPRKGPMKPMALDG
AN3933.1  	WEDGEGRRFVGGDNVTVVAVPGVLRYLDVELAAMGIRMKFRVDN----------------
          	**** *  ::**::.* *.***.*::*********:::*::**                 

MG01572.1 	FRVGNSTVALPPVH
NCU08616.1	KKAGDLMLTA----
FG05627.1 	VRVGK---------
AN3933.1  	--------------
          	
```
