# Supplementary material for: Patterns of Intron Gain and Loss in Fungi
Source: PLoS Biol. 2004 Nov 30;2(12):e422. doi: 10.1371/journal.pbio.0020422 (PMC532390; doi:10.1371/journal.pbio.0020422)
Supplement: Table S1 — Also available at http://genes.mit.edu/NielsenEtAl/. (4.3 MB ZIP). [file pbio.0020422.st001.zip › NielsenEtAl/html/1016.html]

AN4957.1.NCU08687.1.MG05440.1.FG04085.1


```
 CLUSTAL W (1.82) Multiple Sequence Alignments - Introns Inserted


Sequence 1: NCU08687.1	495 aa
Sequence 2: MG05440.1	524 aa
Sequence 3: FG04085.1	526 aa
Sequence 4: AN4957.1	524 aa
Alignment Length: 552 aa
Number Identitical Residues: 195 aa
Alignment Score (without introns) 10491


MG05440.1 	MSGP---VPQARSLADIYPEA------ALDVQRTRWQNLVSRFESLYGHPPAFIARSPGR
NCU08687.1	MSGP---VPIANSLEDIYTRD------ALDTQTKRWNNLLSKFESTYGHQPEFVSRSPGR
FG04085.1 	MSSS---VPVANALSDIYPQD------ALAEQGPRWNNLLTKFESTYGHAASFVARSPGR
AN4957.1  	MATPQELVPRTESIAEVYATDNASATTASPEHVKRFNNLVSHFHKQYNHSPDFVARSPGR
          	*: ... ** :.:: ::*.  .:::::*   :  *::**:::*.. *.* . *::*****

MG05440.1 	VNIIGEHIDYSLYSVLPMAITADCLIAVSPTAAAG-SDTYKVRLANVLPEKFAAKDLDLP
NCU08687.1	VNIIGEHIDYSLYSVLPMAITADTIIAVSTHLPAPKEGTFRIQIANVLDSKFKSREFDIP
FG04085.1 	VNIIGEHIDYSLYSVLPMAITADTLLAVSATPAASDAKSFRIRIANVEDDKFEAADFEVP
AN4957.1  	VNIIGEHIDYNLYDVLPTAVSVDVIIAVKVVPTES--SESAVKIANVLPDKFPTREFSVP
          	**********.**.*** *::.* ::**.   .        :::***  .** : ::.:*

MG05440.1 	VKGDVEIDSTVHEWTNYFKCGLKGATELLRKKHGEG-FVPTGMEVLLDGSVPAGGGLSSS
NCU08687.1	YD-SVDIDATVHEWTNYFKSGLRGALELLRKKHGAN-FKPKSMQILMDGTVPAGGGLSSS
FG04085.1 	FDGEVSIDATKLEWTNYFKSGLRGVMDLLRKKHGKD-FKPCNMELLMDGTVPVGGGLSSS
AN4957.1  	KDSDVEIDPKKHEWVNYFKAGLVGALKVLRKGAADGSFAPASMEVLVDGNVPPGGGISSS
          	 ...*.**..  **.****.** *. .:***  . .:* * .*::*:**.** ***:***

MG05440.1 	AAFTSTTALAVMVANGEKNIDKKELTELAIVSERGVGVNSGG2MDQSASVFSQRGSALFV
NCU08687.1	AAFVTASALAVMAANGEQTVDKKELTELAIVSERAVGVNSGG2MDQSASVFSERGSALFV
FG04085.1 	AAVVSTSSLAIMLANGEKTVDKTELTELAIVNERAVGVNSGG2MDQAASVFSEKGAATFV
AN4957.1  	AAFVCSSALAVMKAN-NHDVSKQDLLDLAVVSERAVGVYSGG2MDQAASIFSRRGYLLYT
          	**.. :::**:* ** :: :.* :* :**:*.**.*** *** ***:**:**.:*   :.

MG05440.1 	SFTPTLTAKPVSFPTTNPELCFVIAQSFVTADKFVTGPIHYNLRVVECSLAAAYLNAVLN
NCU08687.1	SFTPTLLARPVSFPPTTPELTFLIAQSFVTADKFVTGPIHYNLRVVECTLAAAYLNAVLN
FG04085.1 	SFNPSLKAQPVHFPPTNPEITFVIVQSFVTSNKQVTGPIHYNLRVVECSIAASCLNAVLN
AN4957.1  	QFFPNFSVQHVAIPKAAEEITFLMAQSFVTSNKAETAPRHYNLRVAECTLASVVLAKAN-
          	.* *.: .: * :* :  *: *::.*****::*  *.* ******.**::*:  *  .  

MG05440.1 	APGHTLPTDSSPLGSPLHGFHETYF-ALREAD-GQKVPIAVPEQLALLLALTRATLTKDE
NCU08687.1	PPGTLLPGDASPLGISLQGFHETYF-ALSEHNSGATSSKSVTEQLEHLLELTKQTLTKEE
FG04085.1 	PPGTLLPEDAGPLGVSLGGFHDTFFYHLNGSDYSAAKTLTKEEELEKLIEITEKTLTQEE
AN4957.1  	--GLTLPKDNSSLGYSLRTFHNELM------RKEGRLGDPLEYQIDSVIQATLDILTQEQ
          	  *  ** * ..** .*  **:  :              .   ::  ::  *   **:::

MG05440.1 	GYTRDEVAAALGIT-VDELNARFTSRFPVRAERFKLRQRAEHVFSEALRVLEFMSLLQQE
NCU08687.1	GYTRSEIAAVLGLPSTSELDQKFTSRFPVRAERFKLRQRAIHVFSEALRVLKLMDLLETS
FG04085.1 	GYTREEVAKVLNVT-VEDLEKRFMSKLPVRAERFKLRQRALHVFREAHRVIRFMKLLENP
AN4957.1  	GYTREEIAQLLSIS-VPELETTYLSSFPVQAERFLLRQRALHCFKEARRVLDFKACLAN-
          	****.*:*  *.:. . :*:  : * :**:**** ***** * * ** **: :   *   

MG05440.1 	PAAGSADDTAVY----------NALLGAKLNETQDSCRDLYECSCPEIDTICATARSAGS
NCU08687.1	SSSSSSSSSSSSSTITSTPKDLNSRLGALLNETQDSCREVYECSCEEIDRICAIARQAGS
FG04085.1 	VHTGASDTTKFN-----------AELGSLLNETQASCRDLYECSSPELDEICAISLREGS
AN4957.1  	--ASTLDDKRIH------------YLGQLLNESQDSCRDVYECSAPQVDEICNIARKAGT
          	  :.: . .                **  ***:* ***::****. ::* **  :   *:

MG05440.1 	YGSRLTGAGWGGCSVHLVPADK~VDAVKEAWEKEYYSK--MDLTPEQREGAVVVSR~PGS
NCU08687.1	YGSRLTGAGWGGCSVHLVPADK0P----------------------------VVEL0P--
FG04085.1 	YGARVTGAGWGGCSVHMVPADK~VAAVTRALEKEYFAK--RDLTEDQKKGSVVVSR~PAT
AN4957.1  	WGSRLTGAGWGGCTVHMLPQSK~VEAVTKALTEEYYLKYFPDISEEKLKEAMVISK~PSN
          	:*:*:********:**::* .*   : . :  ..   .   . : .. . : *:.  *..

MG05440.1 	GSAV~FVIGEGSLS--
NCU08687.1	----~-----------
FG04085.1 	GSAI~YYIQNGVKP--
AN4957.1  	GSFL2ITGAAISQVDV
          	.:            .
```
