# Supplementary material for: Patterns of Intron Gain and Loss in Fungi
Source: PLoS Biol. 2004 Nov 30;2(12):e422. doi: 10.1371/journal.pbio.0020422 (PMC532390; doi:10.1371/journal.pbio.0020422)
Supplement: Table S1 — Also available at http://genes.mit.edu/NielsenEtAl/. (4.3 MB ZIP). [file pbio.0020422.st001.zip › NielsenEtAl/html/1017.html]

AN1851.1.NCU07567.1.MG00135.1.FG06761.1


```
 CLUSTAL W (1.82) Multiple Sequence Alignments - Introns Inserted


Sequence 1: NCU07567.1	547 aa
Sequence 2: FG06761.1	547 aa
Sequence 3: MG00135.1	547 aa
Sequence 4: AN1851.1	581 aa
Alignment Length: 581 aa
Number Identitical Residues: 395 aa
Alignment Score (without introns) 16693


MG00135.1 	MSLNIPNAPNAGLFKGGYNN2YDSEDGAVLRNIEACRAIASTVQTSLGPYGRNKIVINHL
NCU07567.1	MSLNIPNAPNAGLFKGGYNN2YDSEDGAVLRNIDACRAISSTVQTSLGPYGRNKIVINHL
FG06761.1 	MSLNIPNAPNAGLFKQGYNN2YDSEDGAVLRNIDACRAIASTVQTSLGPYGRNKVVINHL
AN1851.1  	MSLSLPGPSQAGLFKPGYQS2HDAEDGAVIRNIEACQAISQTVQTSLGPYGRNKIVINHL
          	***.:*...:***** **:. :*:*****:***:**:**:.*************:*****

MG00135.1 	QKMILTSDAATILRELDVVHPAAKLLVMASQQQDAEMGDATNLVIVLAGELLRKAEELLR
NCU07567.1	QKMILTSDAATILRELDVVHPAAKLLVMASQQQESEMGDATNLVIILAGELLKKAEDLLR
FG06761.1 	QKMILTSDAATILRELEVVHPAAKLLVMASQQQEAEMGDATNLVIVLAGELLRKAEDLLR
AN1851.1  	QKMILTSDAATILRELEVVHPAAKLLVMASQQQDAEMGDGTNLVIVLAGELLKKAEELLR
          	****************:****************::****.*****:******:***:***

MG00135.1 	MGLKTADIVTGYERAQNFALETLEE~LEVDKVEELRSEEELSKALRTVIASKQNGNEDFL
NCU07567.1	MGLKTSDIVTGYERAQKIALDTLEE~LVVDKVEDLRSQAGLSKAIRTVIASKQNGSEDFL
FG06761.1 	MGLKTSDIVIGYEKAQKFALETLEE~LSVDKVEHMRDQEELSKAISTVIASKQNGNEAFL
AN1851.1  	LGLKTSDIVQGYEKAQNFALKVLED1LEVDRLQDLRSQTELTKALRTVVASKQSGTEQLL
          	:****:*** ***:**::**..**: * **:::.:*.:  *:**: **:****.*.* :*

MG00135.1 	ADLVAEAVLAVMPKNPANFNVDNVRVVKIMGGGLEQSRVVKGMVFSKEPDGSVKKAKKAK
NCU07567.1	ADLVAEAILAVLPKNPANFNVDNIRVVKIMGGNLEQSRVVKGMVFPKEPSGTVKKAKKAK
FG06761.1 	ADLVAEAVLTVLPKNPANFNVDNIRVVKIMGGSLEQSKVVKGMVFPKEPDGSIKKASHAK
AN1851.1  	ASLVAEAVLSVLPKNPVNFNVDNVRVVKIMGGSLEQSRVIKGMVFGREPEGSIKKAHKAK
          	*.*****:*:*:****.******:********.****:*:***** :**.*::*** :**

MG00135.1 	VGVYTCPIDISQTETKGTVLLKNAKEMLDFSKGEEAQLEAAIKELHDVGLRVVVVGSTVG
NCU07567.1	VGVYTCPIDTSQTETKGTVLLHNAQEMLDFTKGEENQLETMIKELYNSGLRVVVAGSTVG
FG06761.1 	VGVFTCPIDAGQTETKGTVLLHNAKEMMDFTKGEESQLETSIKELYDSGLRVVICGERVG
AN1851.1  	VGVFSCPIDISQTETKGTVLLKNAQDMLDFTKGEEERLEAAIKELYDSGLRVVVAGAQVG
          	***::**** .**********:**::*:**:**** :**: ****:: *****: *  **

MG00135.1 	ELAMHYLNRYGILCIKILSKFELRRVCRVVGATPLARLGAPMPDEMGSVDIVETVEIGGD
NCU07567.1	ELAMHYLNRYGILVLKVLSKFELRRVCRVVGATPLARLGAPMPDEMGSIDVVETMEIGGD
FG06761.1 	DLAMHYLNRFGILCIRILSKFELRRVCRVVGATPLARLGAPMPDEMGSIDVVETLEIGGD
AN1851.1  	DLALHYLNRFNILVIKILSKFELRRLCRVVGATPLARLGAPMPDEMGSVDVVETTEIGGD
          	:**:*****:.** :::********:**********************:*:*** *****

MG00135.1 	RVTVFRQEDD--VTRTATVVLRGATQNHLDDIERAVDDGVNVVKAITKDPRLVPGAGCTE
NCU07567.1	RVTVFRQEDE--VTRTATLVLRGATQNHLDDVERAVDDGVNVVKAITKDARLVPGAGATE
FG06761.1 	RVTVFRQEDE--VTRTATLVLRGATQNHLDDIERAVDDGVNVVKAITRDPRLVPGAGATE
AN1851.1  	RVTVFRQEDATAVTRTATIVLRGATQNHLDDVERAIDDGVNAVKAITKDPRLVPGAGATE
          	********* ::******:************:***:*****.*****:*.*******.**

MG00135.1 	IQLVERLQAFGDKTAGLSQYSIKKFGEAFEVVPRTIAESAGLDATEVLSRLYAAAHKKEK
NCU07567.1	IQLVERIQAIGERTQGLAQYSIKKFGEAFEVVPRLLAESAGLDATEVLSRLYAAHQKKDT
FG06761.1 	VELVERIQAYGEKTPGLSQYAIKKFGEAFEVVPRTIAESAGLDATEVLSRLYAAHANSDR
AN1851.1  	IQLVERISAFADRTPGLPQHAIRKYAEAFEVIPRTLAESAGLEATEVLSRLYTAHHQANA
          	::****:.* .::* **.*::*:*:.*****:** :******:*********:*  : : 

MG00135.1 	--------------------------------WTTGVDIE0NEDGTGTLDAQEEGILDLL
NCU07567.1	--------------------------------WSTGVDIE0NNDNTGTLDAVKEGILDLL
FG06761.1 	--------------------------------WDTGIDIE0NDDNTGLLDAKDEGILDLL
AN1851.1  	GVKSTDGDDDDEEESEDSEEERSSGGGEAEAYWTTGVDLE~GSSSSGTLDTVEEGILDLL
          	. .::.........:..:... ::....:.: * **:*:* ....:* **: .*******

MG00135.1 	VSKQWAIKLATEAARTVLSVDQIIVARQAGGPKPPGPNP0NWDED
NCU07567.1	VTKQWAIKLATEAARTVLSVDQIIVARQAGGPKPPGPNP0NWDED
FG06761.1 	ISKQWAIKLATEAARTVLSVDQIIVARQAGGPKPPAPNP0NWDED
AN1851.1  	SSKSWAIRLASESARTVLSVDQIIVARQAGGPKPPGPNP~NWDED
          	 :*.***:**:*:**********************.*** *****
```
