# Supplementary material for: Patterns of Intron Gain and Loss in Fungi
Source: PLoS Biol. 2004 Nov 30;2(12):e422. doi: 10.1371/journal.pbio.0020422 (PMC532390; doi:10.1371/journal.pbio.0020422)
Supplement: Table S1 — Also available at http://genes.mit.edu/NielsenEtAl/. (4.3 MB ZIP). [file pbio.0020422.st001.zip › NielsenEtAl/html/1018.html]

AN5187.1.NCU01724.1.MG08580.1.FG07860.1


```
 CLUSTAL W (1.82) Multiple Sequence Alignments - Introns Inserted


Sequence 1: NCU01724.1	653 aa
Sequence 2: MG08580.1	524 aa
Sequence 3: FG07860.1	535 aa
Sequence 4: AN5187.1	605 aa
Alignment Length: 683 aa
Number Identitical Residues: 196 aa
Alignment Score (without introns) 10469


MG08580.1 	------MPTLAITNFNIVLTVLGAWITLFGLVSYLSKDRLYLSD--~------------P
NCU01724.1	------MPALAITNFNIVLSVLGGWISLFGLVSYLCKESYYMSEAF1ISLLVGVAFSPRA
FG07860.1 	MAYSIWEIPLQTTNFNIVVAVLGGFISLFGLVSYLLKENYYLSEAL1ISLLAGVAFGPNG
AN5187.1  	------MPTLSLINFNIVCATLGGFISLFGLVSYLFKERFYLSEAL1ISLLAGVVFSPHG
          	        .*   ***** :.**.:*:******** *:  *:*::   :   .   ..  

MG08580.1 	P1NLIRPLEYVGGSEVT-------LDEVTLGFSRLVLGVQLVMAGVHLPSRYLRRQWRPL
NCU01724.1	A~NFIRPLAYADYDPLT-------LQAITLAFSRLVLGVQLVLAGVQLPSRYLQQQWRPL
FG07860.1 	A~NFIRPEDYASCNVLSREACETDLSAITLNFSRLVLGVQLVLAGVQLPSKYLVKEWKSI
AN5187.1  	A~NFIRPLDYALGADQN-------LDQITLCFTRLVLGVQLVLAGVQLPKRYLQLEWKSL
          	. *:***  *.     .       *. :** *:*********:***:**.:**  :*:.:

MG08580.1 	TILVGPVMTGMWLMTSLLVWGLVVVPFRGSTVDGTGVLWALAIAACVTPTDPVLSGVIVK
NCU01724.1	SMLLGPIMFMMWIATSLLVWALVPS-FG---PNGIGFLKALVVGSCVTPTDPVLSNVIVK
FG07860.1 	SLLLGPGMTSMWLATSVLVWALAGQ---------PPFLHALAIGSCVTPTDPVLSAVIVK
AN5187.1  	SLLLGPGMAAMWMCSALVIWALVPN---------LSFLHALAVGACVTPTDPVLSNSIVK
          	::*:** *  **: :::::*.*.             .* **.:.:**********  ***

MG08580.1 	GRFADDNVPQQLQRLIIAESGANDGLGYPFLFLPLFLLKYVAAGGGNHSAPAPAPVQGGA
NCU01724.1	GRFADHNVPKELQRIIVAESGANDGLGYPFLFFALYLIKYMG-----HDYPGDG---AGS
FG07860.1 	GKFADHNIPQDLQYLITAESGANDGLGYPFLFLALYLIKFTG-------AGATS---GGA
AN5187.1  	GKFADKNVPQPLQRIIIAESGANDGLGYPFLFFALYLIQYIG-------MDGEG-FSGGA
          	*:***.*:*: ** :* ***************:.*:*::: .         . .  ..*:

MG08580.1 	STAMGLWFGETWGYVVILGAVYGAVVGWVAKELLHWADRRKFVDRESFLVSSIAMA~LFI
NCU01724.1	GRAMALWFYETWVYTIILSIVYGAAVGWIAKELLHWAEERKFVDRESFLVFAISLA~LFI
FG07860.1 	GDAMGLWFGMTWGYTIILSVIYGAVVGWVGKEMLHFAEKRNYVDRESFLVFAIALA~LFV
AN5187.1  	GKAMGLWFYETWAYTILLSVGYGVTVGWVSRELLHWAEEKHYVDRESFLVFAIALA0LFI
          	. **.***  ** *.::*.  **..***:.:*:**:*:.:::******** :*::* **:

MG08580.1 	IG~TGGMLGSDDVLACFVAGNVFTWDDWFRLETLDDMLEPTVDMLL1NVTIFMWYGAICP
NCU01724.1	TG~TCGMIGSDDVLACFIAGNVFTWDDWFRLETLDDSLQPTIDMLL~NVTIFMWYGAVCP
FG07860.1 	LG~TCGMVGTDDVLACFIAGNVFTWDDWFRLETKDDSLQPTIDMLL~NVTIFLWYGAYIP
AN5187.1  	VV1RS---------PCSMANVKLTNSDWFRLETMDDSLQPTIDMLL~NLAVFMWFGAVCP
          	              .* :*.  :* .******* ** *:**:**** *:::*:*:**  *

MG08580.1 	WNSFLHNDV-VPIWRLVLLGVLVLVLRRLPFVLAAHRYIPQISQGHNGGGGGSSLRQALF
NCU01724.1	WEMFLRNGVGMQIYRLIVLGILVLLLRRLPWVFIAH----KIPWGRKMIPQIEGATQAIF
FG07860.1 	WSDFNTTTV-ISIERLVALGICVLLLRRLPWVFAMH----------KWIRQIEEVKQAVF
AN5187.1  	WHLFLENNV-IPIYRLIPLGILILLVRRMPIIFAMH----------KYIEQIESLFQTTF
          	*  *  . * : * **: **: :*::**:* ::  *          :     .   *: *

MG08580.1 	VGFFGPVGVSAIFYLQVALEFVAA-LGEMGLGDHDSIKGLTEMLRVVVWFIAVCSI0VVH
NCU01724.1	VGFFGPVGVSAIFYLYITLEFLRG-MDGPDGKPREDVKDLAETVTVVVWFIAICSI~VVH
FG07860.1 	VGFFGPIGVSAIFYLFITVEFIETHLSDEDGRPRSDVKDLAEQTRIIVWFLTVCSI0IVH
AN5187.1  	VGFFGPIGVGAVFYLSVSREYLNR--ITVNGEIRADAQQVSDTIEVVVWFLVICSI~VVH
          	******:**.*:*** :: *::       .   : . : :::   ::***:.:*** :**

MG08580.1 	GLSIPFGKIFFRLPQQFLG-----------------------------------------
NCU01724.1	GLSIPVGKLGFHLPRTLSKGLKSGAASPSRSVFPDSRRSSGGPLPSFNVGGRVSSFFTGR
FG07860.1 	GLSIPVGKLGYFVPRTLSQ-----------------------------------------
AN5187.1  	GLSIPFAKAGYHLPRTISQVISTSTGDNEPIPLARN--------------------SHTH
          	*****..*  : :*: :    .:.:.. .    . .                        

MG08580.1 	-ASWGHALSRSSTAAILTP--------SQGLPITAP------------------------
NCU01724.1	TSGWARPAERQSDDEHNTERHGETDYGQQGIPLTRPVYRIGGTIIRNPPASDTDAVESEP
FG07860.1 	--AVSDSVINAPDDSLRRR-----------IPFVGKYFG---------------------
AN5187.1  	STATHDNVEATSRRARRHDTSLPTSMSRSHTPQPVAFQIGRSVILSASPSSDAQIGLGSG
          	::.        .           :. . .  *         .     ..::.:.   .. 

MG08580.1 	----------------------~PTTRP----------------PSSRDGE-~--SRPRP
NCU01724.1	KDVAAVGDVEGPSQSQSRLDTT~PTNSSNMTLDDGRRGRGVSATPAAREAAG~SKSHSRA
FG07860.1 	---------------------R~NNDRR----------------PSGPIVIT~GGRSLRA
AN5187.1  	EEPARPVNLVPKSETVGELTRI1RSNDDACG-----QGDRSGIVKSSSIVDA1VSSHDTL
          	.. :   .    :.: .       .            .   .   :.             

MG08580.1 	AGRGISFQE--SGRPQSA-------------
NCU01724.1	PGRSIRFPDDNSQGVGSAPTLVGENTAGVKQ
FG07860.1 	EPHAPAHAGDETPPVRREIRFGDEV------
AN5187.1  	PKYAAQWGPGKQEKLIRIESLYSPTSMDLK-
          	   .     ..           .  : . .
```
