# Supplementary material for: Patterns of Intron Gain and Loss in Fungi
Source: PLoS Biol. 2004 Nov 30;2(12):e422. doi: 10.1371/journal.pbio.0020422 (PMC532390; doi:10.1371/journal.pbio.0020422)
Supplement: Table S1 — Also available at http://genes.mit.edu/NielsenEtAl/. (4.3 MB ZIP). [file pbio.0020422.st001.zip › NielsenEtAl/html/1019.html]

AN9460.1.NCU06677.1.MG07324.1.FG06800.1


```
 CLUSTAL W (1.82) Multiple Sequence Alignments - Introns Inserted


Sequence 1: NCU06677.1	227 aa
Sequence 2: FG06800.1	223 aa
Sequence 3: MG07324.1	233 aa
Sequence 4: AN9460.1	247 aa
Alignment Length: 250 aa
Number Identitical Residues: 87 aa
Alignment Score (without introns) 4606


MG07324.1 	MHSSLVRLQNTFGFFTTVAFVVAAVIAVSDLFAARTPTAPTLRPTSVSV~NKGRPHYYSS
NCU06677.1	MYTSVVRLQNVFGFFTTVAFVVAAFIAASDFLTERAPIVRTLKTTSVQV2VRGRPHYYSS
FG06800.1 	MYNSLTRIQNTFGFFTTVAFVVAAFIAASDFIAPRAPDAGIFGVTNSQV2VKGRPHYYST
AN9460.1  	MHSTLNRAQAVFGFFTTVALFVAGFAALSVLLFPTDKINTEVSLRDVKV2IKGRPHYYST
          	*:.:: * * .********:.**.. * * ::         .   . .*  :*******:

MG07324.1 	KKEEYASIRFDLEAD~LSSLFTWNTKQVFVYVTAEWDERGSSRSSDSSNVTAANQAVIWD
NCU06677.1	KKEEYAVINFSLDAD~LSSLFTWNTKQVFVYVTAEWP------AADHAKTNATNEAVIWD
FG06800.1 	KKEEYAIIRFNLDAD~LRSLFTWNTKQVFIYVTAEWP------GPN----NSTNEAVIWD
AN9460.1  	KKEEYAQMRFDLDAD1LSPLFNWNTKQVFVYVYATYS---SSNKPSENSLTPQSQAIIWD
          	****** :.*.*:** * .**.*******:** * :    ::  ..  . .. .:*:***

MG07324.1 	SIITSP-SSDHLANLGP--------HTLKKLKKSAQGKTIDPSR2-----GILKLKNQRP
NCU06677.1	TIITSP-SADHLANIGP--------VAMKKLRKSAGGKSIDPNR2-----GKLSLKNQRH
FG06800.1 	KIITNP-SADHLQNIGP--------VAMKKLKRSAEGKTIDPDR2-----GFLGLKNQKP
AN9460.1  	TIIEAPESPYSFANLREQFFPTKSGKSASSRKRSSTNKRNDSNK~QDAVPGTLRLRNQKS
          	.**  *.*.  : *:  .  .:.:. : .. ::*: .*  *..: ..: .* * *:**: 

MG07324.1 	KYQITHPTGKLAQTPEVRLRLHYNVQPWVGVLAWNQVRDIAKWKAMDKGLSKPFPLPAIK
NCU06677.1	KYKITHPSGKLAMTDNVTLRLHYNVQPWVGLLTWDQDVDYGYWKALKNGVSKRFKLPAIK
FG06800.1 	KYQITHPTGKVAATEDVKLKLHYNVQPWVGFLTWDQTRDLGHWKALGNGESPKFNLPAIK
AN9460.1  	KYQISDITGKMAERTNVTLSVGWNVQPWVGALWWAPGTGAVPRTGGSTGVSRAFDLPALK
          	**:*:. :**:*   :* * : :******* * *    .    ..  .* *  * ***:*

MG07324.1 	VKETPKAKKA---
NCU06677.1	EKKPASAGAR---
FG06800.1 	TKKKDTKKSY---
AN9460.1  	GTKPKTEGAEASV
          	 .:  .    ::
```
