# Supplementary material for: Patterns of Intron Gain and Loss in Fungi
Source: PLoS Biol. 2004 Nov 30;2(12):e422. doi: 10.1371/journal.pbio.0020422 (PMC532390; doi:10.1371/journal.pbio.0020422)
Supplement: Table S1 — Also available at http://genes.mit.edu/NielsenEtAl/. (4.3 MB ZIP). [file pbio.0020422.st001.zip › NielsenEtAl/html/102.html]

AN2056.1.NCU06200.1.MG05188.1.FG06749.1


```
 CLUSTAL W (1.82) Multiple Sequence Alignments - Introns Inserted


Sequence 1: NCU06200.1	498 aa
Sequence 2: MG05188.1	449 aa
Sequence 3: FG06749.1	429 aa
Sequence 4: AN2056.1	436 aa
Alignment Length: 503 aa
Number Identitical Residues: 147 aa
Alignment Score (without introns) 8637


MG05188.1 	--------------------------------------------------MADIVKNLFG
NCU06200.1	MPYQRPYLFGGSVHQFPGREFPRHKFHVASSSSKSSNCGHIAPTTPANYTMANVINNLFG
FG06749.1 	--------------------------------------------------MASFFKNAFG
AN2056.1  	--------------------------------------------------MAGVFKNVFG
          	                                                  **...:* **

MG05188.1 	VGKAPAKQP-AGGDSD1FADFASKVPDPTPAAAPLTSDSTPTLGGSQP--TAVPWTKWYN
NCU06200.1	GAKPASVIPDKSGDSD1FADFAEGA-DPSPIP---ISPITTTLAGAQPEKTAVPYTKWYN
FG06749.1 	GEKAPEAAS---PDSD1FADFAEAP---SPAP--EVGTQDATVGSPAAAATQAPYTKWYN
AN2056.1  	GAQPSNSAA--LEDGD1FADFVEAP-EPSPAP---ILNTQSAPSLGQNGVEPVVYTKWYR
          	  :..   .    *.* ****..   ..:* .        .: .        . :****.

MG05188.1 	IHERHSLSEFKNEGIVLACIIVVLVLHLIGASMNRTKARKWMRAHAKPLASEFSLVGYAG
NCU06200.1	VHERHSLSEFKAEGFILAAIIVVLILHLFGARLNRSKAKKWIRAHASTLGSEFALVGFSG
FG06749.1 	VHERHSISEFRMEGIILLISSFIFLFHMIGARRNRSRAKGWMRAHAPIMQKEYALVGFGG
AN2056.1  	VWERTSPKDFMQEAMVMPIILLIIIFHFWGTRKNRRRATEWAAAHASALRDEFAVVGFDG
          	: ** * .:*  *.:::    .::::*: *:  ** :*  *  ***  : .*:::**: *

MG05188.1 	VPANVGDKEGDELMAALQTLNESRGEKLLREASLFEFATYATGRQNVAFMDVKMTLLKRF
NCU06200.1	VPRALSDKSGDELTQALADANAQKGDAILKEKSLFEFATYATGRVNVAFVDVKLTLVKRF
FG06749.1 	VPTVNNE-----------NLN---TDTLIKEKSLFEFATYATGRQNTAFTDVKLTLTKKF
AN2056.1  	IQKSEES----------ISVDVTAPNSILKEKSPQEFSTYATGRQNVAFLDVAIRLPKRA
          	:     .             :    : :::* *  **:****** *.** ** : * *: 

MG05188.1 	NPLITFPEMILSFLIDSIPTPEDTVEATIYPFDGKENLVVPAVPGSTELRAKDNKSTYDN
NCU06200.1	NPFVTLAENVIGFFWDSYAQPSDSVEATLYPFDGKEALTVPAMPGAAELRQNDKKSTFDG
FG06749.1 	NPIVNCFEHLAGFFVESVAAPKDAAEVLTYPFDGKESLTVPSIPGAPETR-KEGKSTYDG
AN2056.1  	NPVTYWMDQVFAFFFDSWPSPEETFEATAYTFDGKEKDLIP-VLGKDTSSLKVNNTSYDG
          	**.    : : .*: :* . *.:: *.  *.*****   :* : *      :  ::::*.

MG05188.1 	FVWALVNKDRMKELREDRYDVSITFTKDHPKLPAWLSVMSESAEITDALLTPQLIEAAKQ
NCU06200.1	FVWAIVHKESMKQVRDERYDVSLTYTKDNNKLPQWLTVMTESAEITDALLTPELIKAAES
FG06749.1 	FVWGIVHKDVMRRVRDERYDVSLTFTKDNPKLPVWLTVMSESAEITDTLLTPELIAAVKA
AN2056.1  	FIFAIVHKNHMRNFRNDRYDASMTFTRDHAKLPQWVTVMTENAEITETLLTPELIQAVEQ
          	*::.:*:*: *:..*::***.*:*:*:*: *** *::**:*.****::****:** *.: 

MG05188.1 	AGDLLDYIIVSDQPVEKPTT2VAQTTPRKRIILK-YRLPGNNDYTNLLPLFQYFVRLPDH
NCU06200.1	AGDLLEYLIVSDQPLDKPKT2VEETNPRKRIFLK-YRLPSDNNYEPLLPIFQYFLRMTDQ
FG06749.1 	AGDNFEYLIISDQPVDKPLT~LEETTPRKRLFLK-YSLPSGENYDTLLPLFSHYLQLPDV
AN2056.1  	AGKNFKYLIVSDQPVDKPTK2IEETAPRKRVQLAGYLAPSASGYASTLPLFNQFLRFPDR
          	**. :.*:*:****::** . : :* ****: * .*  *. ..*   **:*. ::::.* 

MG05188.1 	LVQVGHFRAEVMRKVKVVRDETIRQIQKAGEDEKAEERAAEREKARKAKRDAELNALDAK
NCU06200.1	LVQVAHFRPEVLRKVKSVRDEMIKGIQKANEQEKAEELAIEREKQRKAKRDAELAAMDAK
FG06749.1 	LVKVGHFRPEVTKKVRTIREHAISEIKKTAESQRQEELLLEKEKARKAKRDAELKGLDAK
AN2056.1  	LVSHAHFRPEVMRKIRNVREEEIKKLRRLDEQEKAEERRLAAEKIKKEERERTLRGMNAE
          	**. .***.** :*:: :*:. *  :::  *.:: **     ** :* :*:  * .::*:

MG05188.1 	AQKKYLEKEREKQLRKSNKRMTQRA
NCU06200.1	AQKKYLEKEREREAKRQTKKMTTRA
FG06749.1 	AQKRYLEKEREKEMRKNQKRQTQRA
AN2056.1  	EQRKYLEREQQKEQRRSMKRYTKRA
          	 *::***:*:::: ::. *: * **
```
