# Supplementary material for: Patterns of Intron Gain and Loss in Fungi
Source: PLoS Biol. 2004 Nov 30;2(12):e422. doi: 10.1371/journal.pbio.0020422 (PMC532390; doi:10.1371/journal.pbio.0020422)
Supplement: Table S1 — Also available at http://genes.mit.edu/NielsenEtAl/. (4.3 MB ZIP). [file pbio.0020422.st001.zip › NielsenEtAl/html/1020.html]

AN1063.1.NCU02472.1.MG03073.1.FG09547.1


```
 CLUSTAL W (1.82) Multiple Sequence Alignments - Introns Inserted


Sequence 1: NCU02472.1	183 aa
Sequence 2: MG03073.1	173 aa
Sequence 3: FG09547.1	171 aa
Sequence 4: AN1063.1	159 aa
Alignment Length: 184 aa
Number Identitical Residues: 83 aa
Alignment Score (without introns) 4563


MG03073.1 	MASRKPI2TNQGILIDTTPLPESIPKVKELGASSAPLLSASFFIGARCRDYNDDYMQCKT
NCU02472.1	MASRIPQ2FNQQVLYDTTPLPDSIPKVKELGASSAPLMSAAYFIGARCRDYNDDFMQCKN
FG09547.1 	MSTRRPQ2FSQQVLIDTTPLPSDIPAVKEVGASSAPLLSASFFIGARCRDYNDDYMQCKN
AN1063.1  	MSRRDPQ2FNQQNLYDPTPMPDGIPKVAEIGATSAPLTSAAYFIGDRCKAFNDDYMKCKE
          	*: * *   .*  * *.**:*..** * *:**:**** **::*** **: :***:*:** 

MG03073.1 	ENSGNGEAACLKEGRRVTRCARSV~VEDINKSCLEEFRKHWQCLDNNNHQLWQCRPAEWK
NCU02472.1	ENPGKGEFECLKEGRRVTRCARSV2IADINKSCLEEFRKHWTCLEDNNQQLWQCRPAEWK
FG09547.1 	ENPGRGEFECLKEGRRVTRCATSV2IKDINTHCLAEFRKHWECLDDRNHQLWQCRPAEWK
AN1063.1  	EANGRGEIECLREGRKVTRCAASV2IKDINTHCLKQFNTHWECLENNNHRLWECRKQEMD
          	*  *.**  **:***:***** ** : ***. ** :*..** **::.*::**:**  * .

MG03073.1 	LNKCVYENL0GLEKTIPDQPTNKTPVHLRRHQIYAHYDI-PRNQLPFIP-----EKKDAP
NCU02472.1	LNKCVFENL0GLKKEIPDQPPNVTPVHLRKQMIYAHWPI-PRSAEPFVPPTQTGDNNKAP
FG09547.1 	LNKCVFDNM0KLEKKIPDQPTNSTPVHLRPVQTFADVRIGPGDGKPFVP-----AQEDA-
AN1063.1  	LNKCVFDKL0GLKKTIPGAPENQTPVHLRPKQLYASFPG-PQY-----------------
          	*****::::  *:* **. * * ******    :*     *                   

MG03073.1 	SS-----
NCU02472.1	AAASSSS
FG09547.1 	-------
AN1063.1  	-------
          	
```
