# Supplementary material for: Patterns of Intron Gain and Loss in Fungi
Source: PLoS Biol. 2004 Nov 30;2(12):e422. doi: 10.1371/journal.pbio.0020422 (PMC532390; doi:10.1371/journal.pbio.0020422)
Supplement: Table S1 — Also available at http://genes.mit.edu/NielsenEtAl/. (4.3 MB ZIP). [file pbio.0020422.st001.zip › NielsenEtAl/html/1021.html]

AN9080.1.NCU09331.1.MG04426.1.FG00609.1


```
 CLUSTAL W (1.82) Multiple Sequence Alignments - Introns Inserted


Sequence 1: NCU09331.1	124 aa
Sequence 2: MG04426.1	122 aa
Sequence 3: FG00609.1	125 aa
Sequence 4: AN9080.1	125 aa
Alignment Length: 126 aa
Number Identitical Residues: 50 aa
Alignment Score (without introns) 2827


MG04426.1 	----MTAINSKDAAPP1AGPY0SHAIKTPHAIYCSGSIPVDAQGNMVEGTIQQKTEACIK
NCU09331.1	MS--ATPVFSKDAAPP1AGPY~SHAIKTPAAIYCSGQIPCDSEGNLVEGTIQEKTAACIK
FG00609.1 	MSSDQQIVFTKNAPAA1LGPY0SQAIKTPHMIYCSGQIPLTPEGELVQG-ITEQTRQACK
AN9080.1  	MS-DLTNIFTPNACPP1VGPY0SQAVKANGQIFLSGQIPADKNGNLVEGDIRTKTQACCD
          	 : .   : : :* ..  *** *:*:*:   *: **.**   :*::*:* *  :*  . .

MG04426.1 	NLAAVLKEAGSSIEKVVKVN~IFLADMDNFA0AMNEEYAKWFTHKPARSCVAVKTLPKNV
NCU09331.1	NLKAVLEAAGSSIEKVVKVN~AFLTDMSNFA0AMNEEYSKWFTHKPARSCVAVYQLPKGV
FG00609.1 	NVQAVVEEAGSSLSKVVKTT~VFISDMSYFA0EINTEYEKWFSHKPARSCVAVKTLPKNV
AN9080.1  	NIKAILDAAGSSVDKIIRVN0VFLDDMSNFA~EMNAQYEKFFTHKPARSCIAAKQLPKGV
          	*: *::. ****:.*:::..  *: **. **  :* :* *:*:*******:*.  ***.*

MG04426.1 	DVELECIAMP
NCU09331.1	DVEIECIALP
FG00609.1 	DVEVEVIALP
AN9080.1  	PVEIECIALA
          	 **:* **:.
```
