# Supplementary material for: Patterns of Intron Gain and Loss in Fungi
Source: PLoS Biol. 2004 Nov 30;2(12):e422. doi: 10.1371/journal.pbio.0020422 (PMC532390; doi:10.1371/journal.pbio.0020422)
Supplement: Table S1 — Also available at http://genes.mit.edu/NielsenEtAl/. (4.3 MB ZIP). [file pbio.0020422.st001.zip › NielsenEtAl/html/1022.html]

AN6654.1.NCU02479.1.MG02538.1.FG00178.1


```
 CLUSTAL W (1.82) Multiple Sequence Alignments - Introns Inserted


Sequence 1: NCU02479.1	484 aa
Sequence 2: MG02538.1	481 aa
Sequence 3: FG00178.1	690 aa
Sequence 4: AN6654.1	478 aa
Alignment Length: 702 aa
Number Identitical Residues: 291 aa
Alignment Score (without introns) 14124


MG02538.1 	MAS-------ITLESLPDVLANDTKVKLAGIDVDGILRGKLVSKKKFLSIAKDGFGFCSV
NCU02479.1	MATNGNAPKEITAAQLPDLLANDNSVKLAGIDVDGQLRGKLVSKKKFLSIAESGFGFCSV
FG00178.1 	MASS----QTVTVDNLAQVLENDNMVKLAGVDVDGILRGKLVSKKKFLSIAEAGFGFCSV
AN6654.1  	MSAT-----EVTSENVAQILQNDTRVKLAGVDADGMLRGKLVSKKKFLSVVDEGFGFCSV
          	*::.      :*  .:.::* **. *****:*.** *************:.. *******

MG02538.1 	IFGWDMHDQTYFRELEISNAGNGYRDLVAIPDLASFRRVPWENDIPFFLVSFHDPDTMEP
NCU02479.1	IFGWDMHDQTYIKELKVSNKENGYRDIIAIPDLNSFRRIPWENNVPFFLISFHDPDTMEP
FG00178.1 	IFGWDMHDRTYVRELKISNAENGYHDLLAIPDLSTFRRIPWEDNVPLFLVDFLDPDSQKP
AN6654.1  	IFGWDMHDRTYFRELGISNKENGYRDLLAKPDLSSFRRIPWENNVPFFLVSFYDPDTKEP
          	********:**.:** :**  ***:*::* *** :***:***:::*:**:.* ***: :*

MG02538.1 	ICACPRGLLRTQLDKIKSEGYGAMAGA1EYEFYQFRTPSSDPAASSAPSTAAYLRENPPH
NCU02479.1	VSACPRGLLRKQLAKLSEKGYGAMAGA1EYEFFQFRAPSEN----ASDSTASYLKENPPH
FG00178.1 	ICACPRGLVKTQLAKLKEHGYGAMAGA1EYEFYQFKSPDPS-----SSSPAAYLQDNPPH
AN6654.1  	LFACPRSLLRMALRKPEAQGYRAMAGA1EYEFYQFATPNRN-----ASSTASFLKENPVE
          	: ****.*::  * * . .** ***** ****:** :*. .     : *.*::*::** .

MG02538.1 	SLPALTEGMFGYSLTRPVHNQDYFYDIFDTCEKFNCNIEGWHTESGPGVFEA~ALEFGEI
NCU02479.1	SLPSLTEGMFGYSLTRPVHNKDYYYDIFNTCEQFQCNIEGWHTESGPGVFEA~ALEFGEV
FG00178.1 	QLPALTEGMFGYSLTRPVHNQDYYYDVFNTCAKFSCNIEGWHTESGPGVFEA~ALEFGEI
AN6654.1  	ALPSITDGMFGYSLTRPIHNQDYYYGIFDACEQFNCEIEGWHTESGPGVYEA0ALQFGEA
          	 **::*:**********:**:**:*.:*::* :*.*:************:** **:*** 

MG02538.1 	TGMADRASLFK2YAVKSVATKYGITPCFMAKPKQGLPGNSGHMHISLVDNSGKNLFARET
NCU02479.1	KQMADRASLFK2YVVKSVATKYGITPCFMAKPKQGLPGNSGHMHISLVDKDGKNLLAREE
FG00178.1 	AQMADRAALFK~YVVKSVSTKYGITPCFMAKPKQGLPGNSGHMHVSIVDKEGKNLFARET
AN6654.1  	KGMADKAGLFK2YVVKSIGTKHGITPTFMAKPREGLPGNSGHMHISLVNSDDTNAFHRST
          	  ***:*.*** *.***:.**:**** *****::**********:*:*:....* : *. 

MG02538.1 	PDENAPWPDVAQLSDIGRHFLAGLLEGLPDVMPIVAPTINSYKRLVENFWAPVTVSWGLE
NCU02479.1	VDPNAPYPDVAYLSDLGRHFLAGILEGLPDVMPMVAPTINSYKRLVENFWAPVTVSWGLE
FG00178.1 	KDENPKWRDIANLSDMGRHFLAGILVGLPDIMPILAPTINSYKRLVENFWAPVTVSWGLE
AN6654.1  	PDPSPPYPDVAYLSDLGRCFLAGILTGLPDIMPMFAPTVNSYKRLVENFWAPVTVSWGLE
          	 * .. : *:* ***:** ****:* ****:**:.***:*********************

MG02538.1 	HRAASIRLIAPPTSKPGATRFEVRVPGADTNPSLVLAAILALGWRGVQKKLEIPCPPLGK
NCU02479.1	HRAASIRLIGPPGSKAGATRFEIRVPGADANPFYVLSAVLALGWRGVEKKLEIPCPPLGK
FG00178.1 	HRAASIRLICP---KPSATRFEVRVPGADTNPHLVLSAILGCGWRGVEKKLEIPTPPLAM
AN6654.1  	HRAASIRLITPPTASPKATRLEVRVPGADANPHFVLAAIVALGWWGVEKKLEIPVPPLSK
          	********* *. :.. ***:*:******:**  **:*::. ** **:****** ***. 

MG02538.1 	GEQVGGESDTGIRLAKSLREATDRFMRKESVAREAFGDAFVDHYGGTREHEIRQWDEAVT
NCU02479.1	GEQVGGVTDAGARLAKSLKEATDRFMSKNSIAREVFGDEFVDHYGGTREHEIRLWDEAVT
FG00178.1 	GQDVGGDADQGERLAKSLKEATARFMAKDSIAREVFGDDFVEHFGGTREHEVRLFDEAVT
AN6654.1  	GEDMGGESDKGVRLAKNLGAAIATFTRKDSVAREVFGDAFVDHFGGTREHELRLWEEAVT
          	*:::** :* * ****.*  *   *  *:*:***.*** **:*:*******:* ::****

MG02538.1 	DW2EMKRYIETV-------------------------~-------------------~--
NCU02479.1	DW2EMKRYIETV-------------------------~-------------------~--
FG00178.1 	DC2NETRQTEHDTKYPPPLLHDSESFVTLVHSMIEAM2KSYQNVCLHCRKYCKRASS1VQ
AN6654.1  	DW2EVRRYIETV-------------------------~-------------------~--
          	*  :  *  *                                                  

MG02538.1 	------------------------------------------------------------
NCU02479.1	------------------------------------------------------------
FG00178.1 	RTWESAERLTRPKDALIDGVGIVAILAHSHSPKIVLQKQFRPPVNKVVIEVPAGLIDEGE
AN6654.1  	------------------------------------------------------------
          	                                                            

MG02538.1 	------------------------------------------------------------
NCU02479.1	------------------------------------------------------------
FG00178.1 	TAEECAVRELREETGYVGVVTETSPIMFNDPGFCNTNLKMVHVSIDMTLEENKNPKPNLE
AN6654.1  	------------------------------------------------------------
          	                                                            

MG02538.1 	------------------------------------------------
NCU02479.1	------------------------------------------------
FG00178.1 	PGEFIETFTVELKDLWKECERLEAQGHVIDARVATIAEGILLAQRFKL
AN6654.1  	------------------------------------------------
          	
```
