# Supplementary material for: Patterns of Intron Gain and Loss in Fungi
Source: PLoS Biol. 2004 Nov 30;2(12):e422. doi: 10.1371/journal.pbio.0020422 (PMC532390; doi:10.1371/journal.pbio.0020422)
Supplement: Table S1 — Also available at http://genes.mit.edu/NielsenEtAl/. (4.3 MB ZIP). [file pbio.0020422.st001.zip › NielsenEtAl/html/1023.html]

AN0686.1.NCU08946.1.MG06004.1.FG10306.1


```
 CLUSTAL W (1.82) Multiple Sequence Alignments - Introns Inserted


Sequence 1: NCU08946.1	276 aa
Sequence 2: MG06004.1	279 aa
Sequence 3: FG10306.1	280 aa
Sequence 4: AN0686.1	280 aa
Alignment Length: 284 aa
Number Identitical Residues: 191 aa
Alignment Score (without introns) 7782


MG06004.1 	MRHTMAASQFRLP-FLAGAGALAFATAQASLYDVKGGTRAVIFDRLSGVKDTVVNEGTHF
NCU08946.1	----MAARGLDMITKFAIPATVGVALLQNSIYDVRGGSRAVIFDRVAGVKDTVVNEGTHF
FG10306.1 	--MAGAARALGFMYRMAVPASAAVFLGSQALYDVKGGTRAVIFDRLSGVKEEVINEGTHF
AN0686.1  	----MAANGLYNLQRLAIPIGLGAMAVNASLYDVKGGTRAVIFDRLSGVQEQVVNEGTHF
          	     **  :     :* .   .    . ::***:**:*******::**:: *:******

MG06004.1 	LIPWLHRAIIFDVRTKPRMIATTTGSKDLQMVSLTLRVLHRPEVKALPKIYQ0NLGTDYD
NCU08946.1	LIPWLQKAIIFDVRTKPRIIPTTTGSKDLQMVSLTLRVLHRPEVQALPKIYQ0NLGPDYD
FG10306.1 	LIPWLQKSIIFDVRTKPRNIATTTGSKDLQMVSLTLRVLHRPNVKALPKIYQ0NLGADYD
AN0686.1  	LIPWLQKAVIYDVRTKPRNISTTTGSKDLQMVSLTLRVLHRPEVPKLPAIYQ0SYGTDYD
          	*****::::*:******* *.*********************:*  ** *** . *.***

MG06004.1 	ERVLPSIGNEVLKSIVAQFDAAELITQREAVSQRIRTDLMKRASEFNIALEDVSITHMTF
NCU08946.1	ERVLPSIGNEVLKSIVAQFDAAELITQREAVSQRIRADLVKRAAEFNIALEDVSITHMTF
FG10306.1 	ERVLPSIGNEVLKAIVAQFDAAELITQREAVSDRIRNDLTLRAAEFNIALEDVSITHMTF
AN0686.1  	ERVLPSIGNEVLKAIVAQFDAAELITQREAVSNRIRTDLMKRASQFNIALEDVSITHMTF
          	*************:******************:*** **  **::***************

MG06004.1 	GKEFTKAVEQKQIAQQDAERARFIVEKAEQERQANVIRAEGEAESAETISRAIAKSGDGL
NCU08946.1	GKEFTKAVEQKQIAQQDAERARFIVERAEQERQANVIRAEGEAESAETISKSIAKAGDGL
FG10306.1 	GREFTKAVEQKQIAQQDAERARFIVERAEQERQANVIRAEGESESAEAISKAIQKAGDGL
AN0686.1  	GKEFTRAVEQKQIAQQDAERARFIVEKAEQERQANVIRAEGEAESADIISKAVAKAGNGL
          	*:***:********************:***************:***: **::: *:*:**

MG06004.1 	VQIRKIEASREIAQTLASNPNVAYLPGG--KQGTN--ILLNAGRA
NCU08946.1	IQIRKIEASREIAQVLAANPNVAYLPGG--GKGTN--LLMNVGRA
FG10306.1 	IQIRKIEASREIAATLSSNPNVAYLPGGSGKQGGQ--YLLSVGRA
AN0686.1  	IEIRRIEASKDIAHTLASNPNVTYLPGGEGKDGGKSTSLLLGLRS
          	::**:****::** .*::****:*****.. .* ::: *:   *:
```
