# Supplementary material for: Patterns of Intron Gain and Loss in Fungi
Source: PLoS Biol. 2004 Nov 30;2(12):e422. doi: 10.1371/journal.pbio.0020422 (PMC532390; doi:10.1371/journal.pbio.0020422)
Supplement: Table S1 — Also available at http://genes.mit.edu/NielsenEtAl/. (4.3 MB ZIP). [file pbio.0020422.st001.zip › NielsenEtAl/html/1024.html]

AN4947.1.NCU07965.1.MG05077.1.FG04044.1


```
 CLUSTAL W (1.82) Multiple Sequence Alignments - Introns Inserted


Sequence 1: AN4947.1	244 aa
Sequence 2: FG04044.1	240 aa
Sequence 3: NCU07965.1	244 aa
Sequence 4: MG05077.1	246 aa
Alignment Length: 251 aa
Number Identitical Residues: 144 aa
Alignment Score (without introns) 6733


MG05077.1 	MAPAAKTSSGKDLYSVILPTFNERQNLPIVTWLLNKTFTEN2NINWELIIVDDGSPDGTQ
NCU07965.1	MAPTKTT--GKDVYSVILPTFNERQNLPIITWLLNRTFSEQ2NIDWELVIVDDGSPDGTQ
FG04044.1 	MAPKGNK------YSVILPTYNERKNLPIITWLLNRTFTEN2NLDWELIIVDDGSPDGTQ
AN4947.1  	MA-KDNK------YSVILPTYNERRNLPIICWLLERTFREN2KLDWEVIIVDDGSPDGTL
          	**   ..      *******:***:****: ***::** *: :::**::********** 

MG05077.1 	DVAKQLVEVF-KPHVVLQTRTGKLGLGTAYVHGLQFAKGNYIIIMDA1DFSHHPKFIPQM
NCU07965.1	DVAAQLVKLY-APHVQLQTRTGKLGLGTAYVHGLQFAKGNYIIIMDA~DFSHHPKFIPQM
FG04044.1 	EVAQQLVKAY-SPHVLLKPRAGKLGLGTAYVHGLKFVTGNFVIIMDA~DFSHHPKFIPQM
AN4947.1  	DVAKQLQNVWGADHIVLKPRAGKLGLGTAYVHGLQFTTGNFVIIMDA~DFSHHPKFIPEM
          	:** ** : :.  *: *:.*:*************:*..**::***** **********:*

MG05077.1 	IERQKSADYDIVTGTRYAPG----GGVHGWDLKRRMTSKGANILADTLLRPGVSDLTGSF
NCU07965.1	IAKQKAGNYDIVTGTRYAGD----GGVYGWDLKRKLTSKGANIFADTVLRPGVSDLTGSF
FG04044.1 	VALQEKGNYDIVTGTRYAGD----GGVFGWDLKRKFVSRGANLFADTVLRPGVSDLTGSF
AN4947.1  	VRIQKETDADIVTGTRYASRDGIRGGVYGWDLFRKFTSRTANLIADVMLMPGVSDLTGSF
          	:  *:  : *********  ..  ***.**** *::.*: **::**.:* **********

MG05077.1 	RLYKRNVLEKLFETTDVRGFSMQMALAVTAKAMGYSIAEVPITFVDRVYGDSKLGGEEIV
NCU07965.1	RLYKRDVLEKLFQSTDIRGFTMQMALAVTAKSQGFSIAEVPISFVDRVYGDSKLGGEEIV
FG04044.1 	RLYKRAALEKAIATTESKGYSFQMELMVRAKAMGCTVAEVPISFVDRLYGESKLGGDEIV
AN4947.1  	RLYRKSVLEKVISSTQSKGYSFQMEMMVRAKAMGYKVAECPITFVDRLYGESKLGGSEIV
          	***:: .*** : :*: :*:::** : * **: * .:** **:****:**:*****.***

MG05077.1 	EYAKGVFSLWARV
NCU07965.1	EYAKGVLQLWWST
FG04044.1 	QYAQGVFNLWLKV
AN4947.1  	EYLKGVFNLWLKV
          	:* :**:.**  .
```
