# Supplementary material for: Patterns of Intron Gain and Loss in Fungi
Source: PLoS Biol. 2004 Nov 30;2(12):e422. doi: 10.1371/journal.pbio.0020422 (PMC532390; doi:10.1371/journal.pbio.0020422)
Supplement: Table S1 — Also available at http://genes.mit.edu/NielsenEtAl/. (4.3 MB ZIP). [file pbio.0020422.st001.zip › NielsenEtAl/html/1025.html]

AN5727.1.NCU00972.1.MG09726.1.FG11048.1


```
 CLUSTAL W (1.82) Multiple Sequence Alignments - Introns Inserted


Sequence 1: MG09726.1	351 aa
Sequence 2: FG11048.1	350 aa
Sequence 3: NCU00972.1	350 aa
Sequence 4: AN5727.1	369 aa
Alignment Length: 375 aa
Number Identitical Residues: 135 aa
Alignment Score (without introns) 7905


MG09726.1 	MRFPAQTLGFLLSFLP--TVAVALTHKGVDWSSLLVSESAGRSFQDVNGQTRPLEQILAS
NCU00972.1	--MLLSALSAVLSLAG--SATAALTYKGVDWSSVPVEEKAGVSYKNVNGAAQSIEHIFRD
FG11048.1 	----MATLTRLLVFLSYLSVSLALQFRGVDWSSAAVEEQKGIKYTNSAGTAQPLEQILAA
AN5727.1  	-----MILSSLLPLSLVTLTSAALTYRGADISSLLIEEDSGVAYKNLNGETQAFELILAN
          	       *  :* :     .: ** .:*.* **  :.*. *  : :  * ::.:* *:  

MG09726.1 	NGVNTVRQRVWVG-AGSTYNLDYNLRLARRAKAAGMNVYLTLHFSDSWADPGQQ~TIPAG
NCU00972.1	SGVNTVRQRVWVNPSGGTYNLAYNINLAKRAKNAGLGVYIDFHFSDTWADPAHQ~AIPSG
FG11048.1 	NGVNSVRQRVWVNPSNGEYNLDYNIKLAKRAKAAGMSVYLTLHFSDTWADPGHQ~AIPRG
AN5727.1  	NGVNSIRQRIWVNPSDGSYNLEYNLELAKRVQDAGMSVYLDLHLSDTWADPGDQ0ATPSG
          	.***::***:**..:.. *** **:.**:*.: **:.**: :*:**:****..* : * *

MG09726.1 	WP-RDIDNLSWRLYNYTMEVGNAFAGAGVTPSIVSIGNEIRAGLLWPTGDYNNFYNMARL
NCU00972.1	WP-TAIDDLAWKLYNYTFDASNQFHDNGVQPAIISIGNEITGGLLWPTGGTSSWYNIARL
FG11048.1 	WP-TGIDDLAWRLYNYTLDVSNAFQAAGVPPALISIGNEITAGLLFPTGSTKSYYNIGRL
AN5727.1  	WSTTDIDTLAWQVYNYTLDVCNTFAENNVAVEIVSIGNEIRNGLLHPLGSTDHYDNIARL
          	*.:  ** *:*::****::. * *   .*   ::******  *** * *. . : *:.**

MG09726.1 	LHSASSGIRDSRLGRAPKIMVHLDNGW~NWDTQKWFYESLLKQGPFVPGDFDQMGVSFYP
NCU00972.1	LHSASAGIRDSRLNPKPKIMIHLDNGW2NWDTQNWWYTNVLKQGPLVSSDFDMMGVSFYP
FG11048.1 	LNSASYGIKDSRLSPKPKIMIHLDKGW~DWGTQEYFYTQVLNQKGIALDAFDAMGVSFYP
AN5727.1  	LHSGAWGVKDSSLSTTPKILFHLDNGW~DWDAQKYFYDTVLATGTLLSTDFDLIGVSYYP
          	*:*.: *::** *.  ***:.***:** :*.:*:::*  :*    :    ** :***:**

MG09726.1 	FY2GPDATFANLKTSLTNMANTWGKEIIVAETNWPTSCPSPQYQFPADVRSIPFSADGQT
NCU00972.1	FY~TPSATLSSLKSSLTNMANRWGKELVVAETDWPSSCPNPAYAFPSDAKNIPFNAAGQS
FG11048.1 	FY~GSGATFSALETSLTNMANKWGKQIFVSELDWPTSCPSPAQPFPSDMKNIPFSAAGQT
AN5727.1  	FY~NADATLSSLKTSLTNLKSNYGKNVLVVETDWPVQCSSPEYAFPSDLSSIPFSADGQE
          	**  ..**:: *::****: . :**::.* * :** .*..*   **:*  .***.* ** 

MG09726.1 	QFFRRVAKIVSGVRNGNGLFVWEPAWIDNQALGSSCQSNTMFAWPGKA~LSSLSVFKSM-
NCU00972.1	QWIKAVANVVASVPKGKGLFYWEPAWIHNANLGSSCASNSMFSNSGQA~LSSLSVFHNI-
FG11048.1 	QFIQKVASIVSKVRGGAGLFYWEPAWMNNQALGSSCPSNTLFAWPGKA~LSSLAVFKSI-
AN5727.1  	TFLGRLADTLEDVG-GVGIYYWEPGWVDNAGLGSSCEDNLMVDWRDRT0YHQNLYVRSFL
          	 ::  :*. :  *  * *:: ***.*:.*  ***** .* :.   .::   .   .:.: 

MG09726.1 	-------------------
NCU00972.1	-------------------
FG11048.1 	-------------------
AN5727.1  	HVRSKYYIRIEDRPVKTHH
          	   :.     .. . .:
```
