# Supplementary material for: Patterns of Intron Gain and Loss in Fungi
Source: PLoS Biol. 2004 Nov 30;2(12):e422. doi: 10.1371/journal.pbio.0020422 (PMC532390; doi:10.1371/journal.pbio.0020422)
Supplement: Table S1 — Also available at http://genes.mit.edu/NielsenEtAl/. (4.3 MB ZIP). [file pbio.0020422.st001.zip › NielsenEtAl/html/1026.html]

AN0075.1.NCU03739.1.MG08164.1.FG07180.1


```
 CLUSTAL W (1.82) Multiple Sequence Alignments - Introns Inserted


Sequence 1: NCU03739.1	369 aa
Sequence 2: MG08164.1	371 aa
Sequence 3: FG07180.1	380 aa
Sequence 4: AN0075.1	368 aa
Alignment Length: 384 aa
Number Identitical Residues: 165 aa
Alignment Score (without introns) 8514


MG08164.1 	MGNLRSLGLFFLAAFATTQVAAESDVLDLVPSNFDDVVLKSGTPTLVEFFAPWCGHCKQL
NCU03739.1	MVLLKSL----VVASLAAAVAAKSAVLDLIPSNFDDVVLKSGKPTLVEFFAPWCGHCKNL
FG07180.1 	MVLIKSF----VLGALAATVAAKSAVIELLPSNFDDIVLKSGKPTLVEFFAPWCGHCKKL
AN0075.1  	MVRASTL----LLSGLVTLATARSAVLDLIPKNFDKVVLNSGKPALVEFFAPWCGHCKNL
          	*    ::    : .  .: .:*.* *::*:*.***.:**:**.*:*************:*

MG08164.1 	APTYENLAQSFAASKGKVQIAKVDADAEKSLGKRFGVQGFPTLKWFDGKSDKPIDYEGGR
NCU03739.1	APVYEELATALEYAKDKVQIAKVDADAERALGKRFGVQGFPTLKFFDGKSEQPVDYKGGR
FG07180.1 	APVWEDLANTYESAKGKVQIAKVDADAHRELGKRFGIQGFPTLKFFDGKSAKPEEYKSGR
AN0075.1  	APVYEELGQAFAHAEDKVSIAKVDADANRDLGKRFGIQGFPTIKWFDGKSETPEDYKGGR
          	**.:*:*. :   ::.**.********.: ******:*****:*:*****  * :*:.**

MG08164.1 	DLDSLAGFITEKTGVKPKRKLAPPSNVVMLSDSTFSKTIGGDKNVLVAFTAPWCGH1CKS
NCU03739.1	DLDSLSNFIAEKTGVKARKKGSAPSLVNILNDATIKGAIGGDKNVLVAFTAPWCGH1CKN
FG07180.1 	DLESLTTFIAEKTGVKSKKKLEMPSEVTYLNDATFSKTVGSDKHILVAFTAPWCGH1CKT
AN0075.1  	DLESLTAFVTEKTGIKAKGAKKEPSNVEMLTDTTFKSVVGGDKDVFVAFTAPWCGH1CKK
          	**:**: *::****:*.:     ** *  *.*:*:. .:*.**.::********** **.

MG08164.1 	LAPIWEDLAQTFALEDDVIIAKVDAEAENSKATANDQGVQSYPTIKFWAKGQSKPEDYNG
NCU03739.1	LAPTWEKLAATFASDPEITIAKVDADAPTGKKSAAEYGVSGFPTIKFFPKGSTTPEDYNG
FG07180.1 	LAPTWEDLAATFANDKNVVIAKVDAEAPNSKATAEQQGVKSYPTIKWFPAGSKEAVAYES
AN0075.1  	LAPTWETLATDFALEPNVIIAKVDAEAESSKATARSQGVTGYPTIKFFPKGSTEGIVYQG
          	*** ** **  ** : :: ******:* ..* :* . ** .:****::. *..    *:.

MG08164.1 	GRSEADFVKFLNEKTGTQRAAGGGVDATSGTIAALDAIVVKYTGGTLLSDAAAEIKKEAE
NCU03739.1	GRSEADLVKFLNEKAGTHRTPGGGLDTVAGTIAALDEIVAKYTGGASLAEVAEEAKEAVK
FG07180.1 	GRTEQAFVDWINEKAGTHRVVGGGLDNVAGTVESLDTLVAKITGGATIAEIAAEVKKEVE
AN0075.1  	ARTEEAFVDFVNNNAGTHRAPGGTLNEKAGTILALDEIVAKYITSKNFGELVDEAKKVAK
          	.*:*  :*.::*:::**:*. ** ::  :**: :** :*.*   .  :.: . * *: .:

MG08164.1 	SLKDAAQVKYAQYYIRVFDKLSKNDDFASKELARLDGMLKKGGLAPAKLDELTRKTNVLR
NCU03739.1	SLKNSAELKYADYYLRVLDKLSKSEGYATKEFARLEGILKKGGLAPAKVDELTVKVNVLR
FG07180.1 	GLTDAAQKTYAEYYVRVFDKLSSNNDWVSKELGRLDGILAKGGLAPSKRDQIQQKTNVLR
AN0075.1  	TVGG----KYAEYYVKVAEKLAQNEEYAAKELERLKKVLSKGGSAPEKLDDMVSRSNVLR
          	 : .    .**:**::* :**:..: :.:**: **. :* *** ** * *::  : ****

MG08164.1 	KFVEKVT-------------GKDEL
NCU03739.1	KFVEKAAEE-----------AKEEL
FG07180.1 	KFVLKKADEKVEQVKEKVEEVKDEL
AN0075.1  	KFLEVEEKVED--------VVKDEL
          	**:    . .           *:**
```
