# Supplementary material for: Patterns of Intron Gain and Loss in Fungi
Source: PLoS Biol. 2004 Nov 30;2(12):e422. doi: 10.1371/journal.pbio.0020422 (PMC532390; doi:10.1371/journal.pbio.0020422)
Supplement: Table S1 — Also available at http://genes.mit.edu/NielsenEtAl/. (4.3 MB ZIP). [file pbio.0020422.st001.zip › NielsenEtAl/html/1027.html]

AN3607.1.NCU00902.1.MG04521.1.FG00710.1


```
 CLUSTAL W (1.82) Multiple Sequence Alignments - Introns Inserted


Sequence 1: NCU00902.1	538 aa
Sequence 2: FG00710.1	448 aa
Sequence 3: MG04521.1	556 aa
Sequence 4: AN3607.1	417 aa
Alignment Length: 617 aa
Number Identitical Residues: 101 aa
Alignment Score (without introns) 6328


MG04521.1 	MAHRHQQ~QPSGSLFGFVSPMDMQS-----------------------SQP~PVSQ0DNS
NCU00902.1	--MSHGQ~PPPGSSMYGFGAMGMGSGMGSGMGSGMGTGMGTGMGTGMSASQ0MTSD~PQD
FG00710.1 	--MTGGQ0------------MGAGG--------------------------~---Q~DND
AN3607.1  	-------~-------------------------------------------~---M~DPT
          	                                                            

MG04521.1 	AMSLVDPSMLSHFDTPMQMSLEGDSNVLMAAQQPFQRPIASNFPTSGSPSASISSMSQNA
NCU00902.1	MMSLLDTSVFPGFDG-MSMSLDVGD------------------------SMSNPFTPVSV
FG00710.1 	LMSMLDNNMLGSIDD-VPMGLESAD------------------------DSN--------
AN3607.1  	HLQLAQRSHQYGLDP---------------------------------------------
          	 :.: : .    :*                                              

MG04521.1 	PGLAARPQARPGQISNPLLQNHHQAADSGFSLPSPDEHASPMSGALTVASKQNYGGMPPP
NCU00902.1	PPPLPAGNAGPSHVG---VCGGHGAPDQLFSPDDLIATSMSSAGPMIATPTTTTSGPS--
FG00710.1 	----MNGSFGNGQTS---TEESVARAENQFN---TAANNVPGAGPLPAGAFAQMN-----
AN3607.1  	------------------------------------ELTSASYGISMGLPQPQHS-----
          	                                        .  *     .    .     

MG04521.1 	PQPSLQQQSGAHGPPGSIVTEYTKRRDWAARTAEELADLQQILDQDGRIKYVSPSVTALT
NCU00902.1	--------GGPSSGGGSTLTEFTKRRNWPAKVVEELQDWEHILDANGRIKHVSPSVEPLT
FG00710.1 	------------LAGASTLTEFTKRRNWPAKVVEELRDFLQILDANGRIKFASPSILQVT
AN3607.1  	----------LLQSHSSINSSAPGELPWPQRILSEVHDLLLLLSADGVVHFVSPSCKAIT
          	               .*  :. . .  *. :  .*: *   :*. :* ::..***   :*

MG04521.1 	GYTKDEILDVLLQDLIHPDDVGVYLSEMHDAAATGGSLRIYYRLKKKDGTYGVFESTGHA
NCU00902.1	GYKPPEIIDLFLRDLIHPDDVGVFTAELNEAIATGSQLRLFYRFRKKDGNWTIFETVGHA
FG00710.1 	GYSVDEIQDVFLKDLIHPDDQGVFVAELNESIASGNPLRLFYRFKKKDAEYAIFETVGHA
AN3607.1  	GFDKPHLEHDYITRFIHDEDKPVFARELHESVAMARPFHCHFRMYQADNSTCLLEAHGHP
          	*:   .: .  :  :** :*  *:  *:::: * .  :: .:*: : *    ::*: **.

MG04521.1 	HIATARFSTN-------------------PNNQSHFRQAVFLMSRPYPTKNAELLDSFLE
NCU00902.1	HIAAAKFAPN-------------------PQNQSPFCQAVFMMARPYPTKNAGLLDSFLE
FG00710.1 	HIAGAKFAPN-------------------PNNQSPFCQAVFMMARPYPTKNAGLLDSFLE
AN3607.1  	HFEQLNNDATGAANFNQSQHQQQQQQHQQQQHQQRLCQGVFLVCRPYHNESRHLLDSFLE
          	*:   .  ...::. ..:. ...... .. ::*. : *.**::.*** .:.  *******

MG04521.1 	HKIENERLRRRIAELRKEEYEDGGEDAQRNWP---DGRSEMTSEDATVSVSTGTTPRHAQ
NCU00902.1	HKIENERLKRRIAELRREEQEEQ-EESHRTWRMSQEGRSDVTPSDDTA-TQMGMTPFYIP
FG00710.1 	HKIENERLKRRIAELRREEEAEN-DEAQKQWMQSQEGRSDMTPSEGTG-VSS--STFYRP
AN3607.1  	HKLENVRLKERIAQLKREEERDLQTAAQQTRSLQVPSSHAQRPIPQTHKAFASNLDPSLL
          	**:** **:.***:*::**  :    :::    .  .     .   * ..  .       

MG04521.1 	QQVRHSSFGDVKGVGGVLPTDGKLGNAVSGILNSALTRENLEDSTARNRQDSIKDKMMRY
NCU00902.1	MNAQADVM---------MPPPSQP----ASSLNIALTRENLEG-IAGSRPDSIREKMLRY
FG00710.1 	SSERG------------MSEADR------MALNKALTRENLEG-SGGNRQDSLKDKMARY
AN3607.1  	SSGAADDNE-------------------SSDTLDNFNDMDVGFGQGQARAARGQKQGGEE
          	 .   .                      :      :.  ::  . .  *    :.:  . 

MG04521.1 	EGHSHAETIEMLTGLRYKEGERTHGFTSRDSNPTLTKGDAGIAIPIDRDPRVGADKKKKV
NCU00902.1	EGN-HADTIEMLTGLKYQEGERSHGITTGNASPTLIKGDAGIAIPLDRDPRTG-EKKKKI
FG00710.1 	EGASHTDTIEMLTGLRYIEGERSRGITTGNASPTLIKGDAGIAIPMERDPRTG-DKKKKL
AN3607.1  	TSVSHLNDVELLTGLHFTKGERAQGISTGTRDGRLYYSTTTNAKPS-REQRVPPENESRK
          	 . :* : :*:****:: :***::*:::   .  *  . :  * *  *: *. .:::.: 

MG04521.1 	KVAEEYVCTDC-~-------G1TLDSPEWRKGPSGPKTLCNACGL~RWAKKEKKRNPKTG
NCU00902.1	KVAEEYVCTDCV1ADKDAETG~TLDSPEWRKGPSGPKTLCNACGL~RWAKKEKKKNANNN
FG00710.1 	KTSEEYVCTDC-~-------G1TLDSPEWRKGPQGPKTLCNACGL~RWAKREKKRTTS--
AN3607.1  	RLKTEYKCADC-~-------G~TSDSPEWRKGPEGPKTLCNACGL1RWAKMGKKRQDSGL
          	:   ** *:**         * * *********.*********** ****  **:  .  

MG04521.1 	SGSG--------STPAAVRLVEQ
NCU00902.1	NNGGGIGGHNDIHTPMGDHMG--
FG00710.1 	------------HPPTVDQSVA-
AN3607.1  	-----------------------
          	
```
