# Supplementary material for: Patterns of Intron Gain and Loss in Fungi
Source: PLoS Biol. 2004 Nov 30;2(12):e422. doi: 10.1371/journal.pbio.0020422 (PMC532390; doi:10.1371/journal.pbio.0020422)
Supplement: Table S1 — Also available at http://genes.mit.edu/NielsenEtAl/. (4.3 MB ZIP). [file pbio.0020422.st001.zip › NielsenEtAl/html/1028.html]

AN3797.1.NCU04733.1.MG00630.1.FG08405.1


```
 CLUSTAL W (1.82) Multiple Sequence Alignments - Introns Inserted


Sequence 1: NCU04733.1	1105 aa
Sequence 2: MG00630.1	956 aa
Sequence 3: FG08405.1	1060 aa
Sequence 4: AN3797.1	949 aa
Alignment Length: 1152 aa
Number Identitical Residues: 264 aa
Alignment Score (without introns) 14839


MG00630.1 	MADASSTKQTIVQTLNDAQRRAVSSGATTVAILAGPGSGKTHTLASRVVWQVDVVGYRPQ
NCU04733.1	MSPSAPPKHSILDSLNNAQARAVTSDAATVAILAGPGSGKTHTLTSRVVWLVDHVGYQPQ
FG08405.1 	--MSSIVQDAILQSLNEAQRRAVTSTASTVAILAGPGSGKTHTLTSRVVWLIQRVGYRPS
AN3797.1  	----MDPLDPILDGLNSAQRTAVTSSAPILQVLAPPGSGKTKTLTSRVAYLLAHDGYRPQ
          	        ..*:: **.**  **:* *. : :** ******:**:***.: :   **:*.

MG00630.1 	DVVVATFTVKAAREMGERICKVLGPERGRKVVLGTFHSISRRYLAAYGKKIGLDQKFGIA
NCU04733.1	DVVVATFTVKAAREMKERIGKALGNGRENKIILGTFHSIARRYLAAYGRHIGLSEKFSIA
FG08405.1 	DVIVATFTVKAAREMKERIGKTLGEECEKKIVLGTFHSIARRYLSIYGNRIGLDSKFGIA
AN3797.1  	DVICCTFTIKASREMRERLAK---------------------------------------
          	**: .***:**:*** **: *                                       

MG00630.1 	DDSDSRAIIT~RICKRHQLGIDPAMARGWISKKKSRANDRVVSSQQEKAAKSGKQQPIHS
NCU04733.1	DDNDSRSIIT~RICKRLQLGLDPPMAKAWISKKKAKGMEPSPSSLPQPPKKQPVQR--GQ
FG08405.1 	DDGDSRAIIQ~RICKRLKLNIEPQQAKSWISKEKAKGPDAMAPPPTQKGRKE-------N
AN3797.1  	---------L0RIVKRLQLGIQPNTARARISHQKAHG----VSPDDVAAKQNKISKLLEH
          	           ** ** :*.::*  *:. **::*::.     ..      :.  .     

MG00630.1 	RELATCYEEYQAQLTRSNLLDYDDLLVKGVELLRAAPECVSNVQAVLIDEYQDTNGVQYD
NCU04733.1	RDLEVCYTEYQTHLERSNLLDYDDLLVRCVELLRKFPSCVSNIQTVLIDEYQDTNGVQYE
FG08405.1 	PELRTCYEEYQAQLTRSNLLDYDDLLVKCVELLRDHPACVSNVQTVLIDEYQDTNGIQYE
AN3797.1  	REFVQVYREYESELATSNLLDYDDLLLRCAELLRKHPKCVSNVQVVLVDEFQDTNQIQYD
          	 ::   * **::.*  **********:: .****  * ****:*.**:**:**** :**:

MG00630.1 	LMKLLAQRHGRITVVGDPDQ-----------------------N1KVALEENYRSSESIL
NCU04733.1	LMRLLAQKHQRITIVGDPDQSIYGWRSAEIKNLWRLLRDYPKTD~EISLEENYRSSQAIL
FG08405.1 	LMKLFAQAKQRITIVGDPDQSIYGWRSAEVKNLFRLLREYPNTD~EISLEENYRSSQSIL
AN3797.1  	LMNLLASWNRRITVVGDPDQSIYGFRSAEIENLKRMQRLYRNTE~VVLLEDNYRSSGSIL
          	**.*:*. : ***:******:  .  ::. ..         .::  : **:***** :**

MG00630.1 	ATSLKIIQQEEKRFDKVLKAVHTRGTKPVLRKLRNSAQEAEWIVAELRRTILLSGDMMNH
NCU04733.1	DLSLLVIQQDKKRYQKVLKPVHDRGSRPVLRKLKNASTEAEWIVAEIRRALLMSGSMLTH
FG08405.1 	DVSLTVIQQDTKRYKKVLLPVHTKGARPVLRSLKSSSAEGEWIVSEIKRAVMMFGDMLKF
AN3797.1  	KSAQDVIEQDLSRPAKKLQPTHTFGTLPVLRKLPSAEDEAQWLVLEIKRCIVLTGKLLKY
          	  :  :*:*: .*  * * ..*  *: ****.* .:  *.:*:* *::* ::: *.::..

MG00630.1 	DDVAILLRSAALSRHIESALGKAGIPYRMVG1GFKFYERAEIKLILDYLRVVYQPDNNDA
NCU04733.1	NDVAVLLRSASLSRHVESALGKAGIPYRMVG~GFKFYERSEIKTILDYLRVIHQPDNNDA
FG08405.1 	EDVAILLRSAALSRHIESALGKAGVAYRMIG~GHKFYERKEIKVLIDYLRVVSQPDNNDA
AN3797.1  	SDFAILLRSASLSRQIESAMGKHGVPYRMVG~GQRFFDRVEVKLLLDYLRVISHPENSEA
          	.*.*:*****:***::***:** *:.***:* * :*::* *:* ::*****: :*:*.:*

MG00630.1 	LARIINVPKRGAGDATIKALLEEAEKSQQSLWSLLRKHCTGGKQAKTTLRKQVEQKISGG
NCU04733.1	FGRIMNVPKRGIGDGTIKNLIEEAEKSSLSLYSLLVKHCRGDRTAKTKITKHAEQKISGE
FG08405.1 	LARIINVPRRGIGEATVKALIEEAEQSDMSLWTLILKHCRGNRKAKANIRPKMEQKLNTE
AN3797.1  	LLRIINVPSRKIGEETVKMLLNGAEKAGKPLWDYIKDVAQGRRSTEKAMSKPTNQGLS-S
          	: **:*** *  *: *:* *:: **::  .*:  : . . * : ::  :    :* :.  

MG00630.1 	LIRLILDIRKKMDESTAENPYSMLDVISQVLSRLDFEQHLKDTYNDEWEGRWANVQEFIN
NCU04733.1	LIRMLNGIRKKMEEAAESNPFGLVDLIEHLLTALNFQNYLQVTYPDDHEQRWANVQEFIS
FG08405.1 	LIRTITSLQKQASEISQSSPVTLVNLIEQLLTHLDFKKYLEEEYTEEHEQRWANVQEFVN
AN3797.1  	LVGLIEYSREKLRQCVDRS--APRRLLEIVMKRLSFREFITATYGSNDENRWANVEELMS
          	*:  :   :::  :    .      ::. ::. *.*.:.:   * .: * *****:*::.

MG00630.1 	LATDFMTGNEV--EEELPEIDGLQQT---PERDTLARFLANVSLATDVQNNDPGQ----N
NCU04733.1	LANDFVRDLHVSGEDALPEIEGLEQS---KEEEVLPQFLANVSLASDAQKGEEGQE---N
FG08405.1 	LVSDFMKDFGAPDEDALPEIDNLDQV---KVDDMLGRFLANVALASDAQNKGDTTE---Q
AN3797.1  	QADDATAADESEQDENLPEIAGLTQQESHPGEEALSRFLANVALSTEVQPKEETEGEDQA
          	 . *         :: **** .* * .:    : * :*****:*:::.*       ... 

MG00630.1 	RPMVTVSTIHAAKGLEWPVVFIPAAYNGSIPHIRSEDGDEERRLLYVAMTRAKALLYLSY
NCU04733.1	KPLVTISTIHAAKGLEWPIVFIPAVYNGSIPHMRSEDGDEERRLLYVAMTRAQSLLYLSY
FG08405.1 	KSLVTISTIHAAKGLEWPVVFVPSVYTGSIPHSRSEDTDEERRLLYVAMTRAKALLYLSC
AN3797.1  	QEKVTISTIHAAKGLEWPVVFIPAAYEGIIPHSRAEDSDEERRLLYVAMTRAQALLYLSY
          	:  **:************:**:*:.* * *** *:** **************::***** 

MG00630.1 	PVVGGYGNGEKVTLSSFLSGVEG-HFAVQGPTFNSAVLEELARILRRKAPSQKDVFGRLK
NCU04733.1	PVYHSPGSSERTEMSSFVSPVAN-SFAKKGPTFERPLMTHIAKVLQRELPSEKEIF-KGI
FG08405.1 	PLYGSQGLSSKVELSPFVAPFASKVFAKKGPSFDRAVVEGVAKILGRDAPSEKTVFGKLP
AN3797.1  	PLRQARENAETNVTSFLPQELITSRLRPVGPKLDEKVVYGIADILRRERPSAVAMLEGLD
          	*:  .   ..    * :   .  . :   **.::  ::  :* :* *. **   ::    

MG00630.1 	PFEVSMEDDIFPSDPEQDLGRSGNKTGLGRSSSGSGLSQGQPPTKRQRMHSTSGGLEQQD
NCU04733.1	PNMSHTEDNMYPIDPDDAFQESGQGFDSSRSK--------RPKLYNPVAISTGDGHEEPQ
FG08405.1 	P-MFSVEDDRFPVDPADHKNVERIDEESGRQYS-------RAPKRQRVSHPTTSYDESDE
AN3797.1  	S-LPSVYDDQWTEDGRESPG-MIIRWDGSRATD-------DEPSSKRRRYEREQYSTTTS
          	.      *: :. *  :           .*  .            .             .

MG00630.1 	QTWHQEYSTTMQQASGFTVSSLPGFVTAGAHHSAFPVPD--------------PPYSRDS
NCU04733.1	--WAKPHATTMDQASTFTIASLPGFVNAAVHQAAITAAANASAAAALEARANKASASSGI
FG08405.1 	LPWQREYATTMEKSSEFTVAALPGFTTAGAHRVALDAAAAAAPPP--------DSSKPGV
AN3797.1  	-IASMSSTNRMNGSSLMVPTTMSGFSTAREYITTT--------------------IQKQE
          	       :. *: :* :. :::.** .*  :  :                      .   

MG00630.1 	KTGQPNRGTGQRSLLGFVT---NSGQPRTGTGSQKQILQAPQNRGGTHHPRPPQSGRQQ-
NCU04733.1	KRGTNKRPADQKSILGFVK---TNFEPATGVGSSITSASTTTNRPSVPTPRLPQPALSRN
FG08405.1 	KKGSTKRGVGQKSMLGWLNQGPNAPKPPPEPQSQPQSRSLAQARYESALSRQNSRLLSLP
AN3797.1  	QSLQEERGAKSKPVVAFNR--PSALQKSIASFFGQSSAQSTQQRGLVQLLPNSSGALDHP
          	:    :* . .:.::.:    ..  :            . .  *         .   .  

MG00630.1 	-----------------GWGNDQMVG-----------RPAIAPELANHRLGGTRSTTLPR
NCU04733.1	PSLPLNHSHSPPAHTFTGAGSLQRAQSHPSKPSVPATTAGIAPELASHRLGSSRGLNLNV
FG08405.1 	-------------------GPQ---------------KPSIDPELAKHKLPTAR--PLAK
AN3797.1  	-------------------GRP--------------LHPRQSNAIPTQYIGHRPPTQHFR
          	                   *                  .     :..: :          

MG00630.1 	PGPPAKR------EHGAISTRNQYAHLSSSPTKAQPQ--------------EETNDPTSS
NCU04733.1	TRPPSLQPPKDASSTSSFHYKKQYVCFSSSPTRPEPDDGISAPPKSAGGGDEEEAEETEQ
FG08405.1 	PNVPKIS---------DGVPQKAYSCFSSSPPRPPSK-----------MEPEEEKKEDVG
AN3797.1  	PPRPALET----------SDPNRYTWLAAS-SRPTDK--------------ANSLKSQVH
          	.  *   .             : *  :::* .:.  .               :  .    

MG00630.1 	PPAPTRPVSTFHNTTCT--MPQGQ~G~GF--------------KRPAALTREE1LVF---
NCU04733.1	PPTPTRPAGSLHVTTCHNVLPTGL~G~GYPGKRAARGGAVGVVRKPVGLARLD~GTEGMG
FG08405.1 	EAMPNKPASTLHATTFMSVRPNGG0V2STKPQ-----SSLTPTPRNNMLPTLT~ARFSTS
AN3797.1  	TVIDTNGGS----KDSAASEAQGC~A~GS---------------RPGMVTVGT~GAG---
          	    ..  .    .      . *    .                :   :.          

MG00630.1 	------------------------------------------------------------
NCU04733.1	AIKPMERLMKPFKPLTMNRTAGGSGVGGGSGRGSGSGNGSTMGRPGLQRPGIGR------
FG08405.1 	AVQGARRYISTTSPKRNTVAGDTTSTSLMSKWNNLSPQNRRYSKAGLAVVAGVDVYMHYT
AN3797.1  	GMRPAATFHTTTMSMVQSQSGGPARRTLGIRR-SMNGWEERMKRVNK-------------
          	.        ..  .   . :.. :         . .         .              

MG00630.1 	------------------
NCU04733.1	------------------
FG08405.1 	YWPYIRSWFSSVEMEGKN
AN3797.1  	------------------
          	
```
