# Supplementary material for: Patterns of Intron Gain and Loss in Fungi
Source: PLoS Biol. 2004 Nov 30;2(12):e422. doi: 10.1371/journal.pbio.0020422 (PMC532390; doi:10.1371/journal.pbio.0020422)
Supplement: Table S1 — Also available at http://genes.mit.edu/NielsenEtAl/. (4.3 MB ZIP). [file pbio.0020422.st001.zip › NielsenEtAl/html/1029.html]

AN1151.1.NCU04071.1.MG01335.1.FG09485.1


```
 CLUSTAL W (1.82) Multiple Sequence Alignments - Introns Inserted


Sequence 1: NCU04071.1	366 aa
Sequence 2: MG01335.1	372 aa
Sequence 3: FG09485.1	364 aa
Sequence 4: AN1151.1	360 aa
Alignment Length: 373 aa
Number Identitical Residues: 200 aa
Alignment Score (without introns) 10417


MG01335.1 	MPLHHLMIGTWTPPGAIFTVQFDDEKLDIKLVKRTEIPKDEPISWMAFD0HARKNIYGSA
NCU04071.1	MPLHHLMIGTWTPPGAIFTVQFDDEKLTCKLIKRTEIPQDEPISWMTFD0HERKNIYGAA
FG09485.1 	MPVHHLMVGTWTPPGAIFTFAFDDEALTLKLVKRTEIPQDEPISWMTFS0HDKKAIYGAA
AN1151.1  	-MKHHLMVGTWTRPGRIYTVAFDDEALTLELVKKTDIPEAEPISWMTFS0HDKKTIYGAA
          	   ****:**** ** *:*. **** *  :*:*:*:**: ******:*. * :* ***:*

MG01335.1 	MKKWSSFAVKSPSEIVHEASHPMGGDP1KAASPDTNTRAIFLLPATKPPYAVYCNPFYKP
NCU04071.1	MKKWSSFAVKSPTEIVHEASHPIGGHP1RANDADTNTRAIFLLAAKQPPYAVYANPFYK-
FG09485.1 	MKKWSSFAVESPTSITHQVSHPMEHDP1NASLATTNTRAIFLLAANKPPYAVYCNPFYD-
AN1151.1  	MKKWNSFAVNSPTEIVHQVSHPVAGHP1MAANEDTNTRAIFVLAARKPPYNVYGNPFYK-
          	****.****:**:.*.*:.***:  .*  *    *******:*.* :*** ** ****. 

MG01335.1 	HAGYGNIFNVSDTG-ALATNVQDYPYQPDSGIHGMVFDPPTETYLYSADLSANRLWVHRR
NCU04071.1	FAGYGNVFSVSETG-KLEKNVQNYEYQENTGIHGMVFDP-TETYLYSADLTANKLWTHRK
FG09485.1 	HAGHGAVFTTDDTTKALKENVQNYPYQPNTGIHGMVFDP-EEEYLYSADLRANKIWTHRR
AN1151.1  	YAGFGNVFSVESDG-RLAKNIQNYEYEPNTGIHGMVFDP-TETYLYSADLQANKIWTHLK
          	.**.* :*....    *  *:*:* *: ::*********  * ******* **::*.* :

MG01335.1 	HLPGRNPAEVELVGSIEAPDPGDHPRWVAMHPTGKYLYALMEAGNRICEYVIDPQTHMPV
NCU04071.1	LASG----EVELVGSVDAPDPGDHPRWVAMHPTGNYLYALMEAGNRICEYVIDPATHMPV
FG09485.1 	RSKD--DPSLELVGSVDCADEKDHPRWVAMHPTGNYLYALMEKGNRICEYVIDPDTHLPV
AN1151.1  	DPKTG---ELSLVDCLEAPDPGDHPRWVEMHPSGKYLYALMEAGNRLAVYVIDERTHKPV
          	        .:.**..::..*  ****** ***:*:******* ***:. ****  ** **

MG01335.1 	YTHHSYPLVPPG~MPKKDRETGKGLYRSDVCAVSSSGRYLFASARSNSFDLTGYIAAFRL
NCU04071.1	YTHHSFPLIPPG~IPDRDPETGKGLYRADVCALTFSGKYMFASSRANKFELQGYIAGFKL
FG09485.1 	YTHKHYPLIPPG~IPDR-----WTQYRADVCVLSSSGKYLFASSRANSFDLTGYVAAFKL
AN1151.1  	FTHITYPLLPPG1LPPR------NKYRGDVTFTTRSGEYLFATTRSNHFDVTGYITAFKL
          	:**  :**:*** :* :        **.**   : **.*:**::*:* *:: **::.*:*

MG01335.1 	RDDGSIEEQLCLNPTPTSGGHSNAVSPCPWSDEWVAMTDDEQGWIEMFRWEGEFLARVAR
NCU04071.1	RDCGSIEKQLFLSPTPTSGGHSNAVSPCPWSDEWMAITDDQEGWLEIYRWKDEFLHRVAR
FG09485.1 	SDTGAIERQICLNPTPTSGGHSNAVAPCPWTDEWVAITDDQEGWLEIYRWQGEFLARVAR
AN1151.1  	GPNGNIERQLFIHPTSTSGGHSNAVSPCDWSDEWLALCDDQLGFVEIYRFRDENLSRVAR
          	   * **.*: : **.*********:** *:***:*: **: *::*::*:..* * ****

MG01335.1 	LRIPEPGFGMNAIWYD
NCU04071.1	VRIPEPGFGMNAIWYD
FG09485.1 	VRTPEPGFGMNAIWYD
AN1151.1  	VDIPEQGFGMNAIWYD
          	:  ** **********
```
