# Supplementary material for: Patterns of Intron Gain and Loss in Fungi
Source: PLoS Biol. 2004 Nov 30;2(12):e422. doi: 10.1371/journal.pbio.0020422 (PMC532390; doi:10.1371/journal.pbio.0020422)
Supplement: Table S1 — Also available at http://genes.mit.edu/NielsenEtAl/. (4.3 MB ZIP). [file pbio.0020422.st001.zip › NielsenEtAl/html/103.html]

AN2052.1.NCU04117.1.MG06878.1.FG01885.1


```
 CLUSTAL W (1.82) Multiple Sequence Alignments - Introns Inserted


Sequence 1: NCU04117.1	195 aa
Sequence 2: MG06878.1	213 aa
Sequence 3: FG01885.1	198 aa
Sequence 4: AN2052.1	209 aa
Alignment Length: 222 aa
Number Identitical Residues: 120 aa
Alignment Score (without introns) 4988


MG06878.1 	------MVAQEPVLFSGTVAENIAYGRPEASRAEIVAAAQKANCGFISDF~PEGLETQVG
NCU04117.1	------MVSQEPVLFSGTIAENIAYGRPRAPRTEIIAAAQKANCGFISDF~PEGLETQVG
FG01885.1 	------MVSQEPVLFSGSIAENIAYGRPQASRFDIISAARQANCNFISDL~PDGLETQVG
AN2052.1  	MRRKIGVVAQEPVLFSGTIAENISYGSPHSTRSEIVAAARKANCQFISDF0PDGLDTQVG
          	   . .:*:********::****:** *.:.* :*::**::*** ****: *:**:****

MG06878.1 	ARGAQLSGGQKQRIAIARALLKDPDILILDEATSALDAESETLVNSALAQLLKGRNTTIS
NCU04117.1	ARGAQLSGGQKQRIAIARALLKDPDILILDEATSALDAESETLVNSALAELLKGRSTTIS
FG01885.1 	ARGSQLSGGQKQRIAIARALLKDPDILILDEATSALDAESETLVNEALAGLLRGRNTTIS
AN2052.1  	PRGAQLSGGQKQRIAIARALIKDPDILILDEATSALDAESETLVNSALAALLRGNNTTIS
          	.**:****************:************************.*** **:*..****

MG06878.1 	IAHRLSTIKRSDKIIVLSSEGKVAEIGSYTELSANPDSAFSKLMEWQMSGGD~IPTQQRP
NCU04117.1	IAHRLSTIKRSDKIIVLSSEGTVAEIGSYTELSANKDSHFSKLMEWQMSGGD0E------
FG01885.1 	IAHRLSTIKRSDQIIVLNNEGKVAEIGSYRQLAADKESAFSKLMEWQMSGGE0E------
AN2052.1  	IAHRLSTIKRSDSIVVLGNDGTVAEQGTYEELSARPDGAFTKLMEWQLSGGD~SKP----
          	************.*:**..:*.*** *:* :*:*  :. *:******:***:   .    

MG06878.1 	PPIITEAEAISEELEGDEAAA---AEEEDVEGEHKDAKKTSEKQ
NCU04117.1	---------IEEEFA--EAEN---DVDDAVEKDVKSHKEPVRE-
FG01885.1 	---------IEDDLERGEEEE---EQFDEHDQENRDPKSEEKRP
AN2052.1  	--------PVSPSLDPETEEKPWVEQPEEYGDAEANDKAEQQR-
          	        .:. .:       .     :       . *   ..
```
