# Supplementary material for: Patterns of Intron Gain and Loss in Fungi
Source: PLoS Biol. 2004 Nov 30;2(12):e422. doi: 10.1371/journal.pbio.0020422 (PMC532390; doi:10.1371/journal.pbio.0020422)
Supplement: Table S1 — Also available at http://genes.mit.edu/NielsenEtAl/. (4.3 MB ZIP). [file pbio.0020422.st001.zip › NielsenEtAl/html/1030.html]

AN8215.1.NCU09545.1.MG08171.1.FG07127.1


```
 CLUSTAL W (1.82) Multiple Sequence Alignments - Introns Inserted


Sequence 1: MG08171.1	690 aa
Sequence 2: FG07127.1	672 aa
Sequence 3: NCU09545.1	681 aa
Sequence 4: AN8215.1	684 aa
Alignment Length: 718 aa
Number Identitical Residues: 332 aa
Alignment Score (without introns) 16752


MG08171.1 	MDKITDKIAALPPDASYFSLEFFPPKTAM~GFSNLRDRLDRMARALRPLFVNVTWGAGGS
NCU09545.1	MEKITDKVAALPPDSNYFSLEFFPPKTAM~GFANLRDRLERMARGLRPLFVNVTWGAGGS
FG07127.1 	MDKITDRIAALPADGTYFSLEFFPPKTAM~GFSNLRDRLHRMERALRPLFVNVTWGAGGS
AN8215.1  	MEKITHKIAALPPGANYFSLEFFPPKTQM0GFANLQARLERMAQALRPLFVTVTWGAGGS
          	*:***.::****....*********** * **:**: **.** :.******.********

MG08171.1 	TAQKSLELAEICQRELALTTCLHLTCTNMSRKLIDKALEDAKALGIRNILALRGDPPRPG
NCU09545.1	TAQKSLELAEICQRELGLTTCLHLTCTNMSRRLLDKTLEDAKVLGIRNILALRGDPPRAA
FG07127.1 	TSQKSLELAELCQREVGLTTCLHLTCTNMSKKLIDEALSDAKALGIRNILALRGDPPRRE
AN8215.1  	TAARSLELAEICQRQLQLTTCLHLTCTNMSRALVDQALEEAKVLGIRNILALRGDPPRSE
          	*: :******:***:: *************: *:*::*.:**.***************  

MG08171.1 	EYAIPDDSEGDVN-EFTWAIDLVRYIKLNHGDYFCVGVAAYPEGHAEESHPTNQSLEHDL
NCU09545.1	EYHDENDPEPTDDEEFSWAADLVRYIKKTHGDYFCIGVAAYPEGHAEEAHPREQSLEHDL
FG07127.1 	EYRDAEDSEDDGGQDFNWAVDLVRYIRKTHGDYFCIGVAAYPEGHAEESHPLGQSVEHDL
AN8215.1  	EYN--MHGEDDSNKDFTFAVDLVRYIRKKYGDYFCVGVAAYPEGHPADSFQDIQDPKVDL
          	**    . *   ..:*.:* ******: .:*****:*********. ::.   *. : **

MG08171.1 	PYLVEKTQAGADFIITQLFFDISAYEKFEKTLREHPSGAFKDIPIIPGLMPIQNYQMIKR
NCU09545.1	PYLVEKVQAGADFIMTQLFFDIDAYEHFENTLRDHPSGAFKDVIIIPGLMPIQSYQMIKR
FG07127.1 	PYLVQKVQAGADFIQTQLFFDIEAYEHFETTLREHPSGAFNGIPIIPGLMPIQSYQMIKR
AN8215.1  	PWLVEKTQAGADFIMTQLTYDIDAYTEFENLLRNHESGTFKTIPIIPGLMPIHSYKILTR
          	*:**:*.******* *** :**.** .**. **:* **:*: : ********:.*:::.*

MG08171.1 	TTKLSHAKIPDPLMARLDAVKKDDEQVKKVGVDIISELVDQVKEVKNRSSGPKGFHFYTL
NCU09545.1	TTKLSHAKIPPALMARLDAVKGDDEKVKMEGVAIVSEMIERIKEIKSRTPGPRGFHFYTL
FG07127.1 	TTKLSHAKIPDHLLERLDAVKGDDERVKMVGVDIVNELIDKIKDIKSKTPGPKGFHFYTL
AN8215.1  	VTKLSHVKIPPPILAKLDEVKHDDDAVKRLGVDILAELVDGMKKLP--TPGLRGFHFYTL
          	.*****.***  :: :** ** **: **  ** *: *::: :*.:   :.* :*******

MG08171.1 	NLEKAVSFIVERTNLIPATTPDDDEVAVVDDVALPSIHLNGATPAKDHLSVSSLHPPTHT
NCU09545.1	NLEKAVAFIAERTNLIPPGTPDEDESAILPD--IPQLRINGSSVPTRSI---------QD
FG07127.1 	NLEKAVSFILERANLIPDPPENEEAVVAVDELAIPALQINGNMRSSSHS--------RSR
AN8215.1  	NLEKTVSFILERCKLIPD--HDDDAEAVADG--VSLLTVDAEIAN---------------
          	****:*:** ** :***    :::  .      :. : ::.                   

MG08171.1 	NSRRPSTIGSDPRNRVIVSSGRPASHPD---YEATGFEASVPAQAINSRANTLAISEGEG
NCU09545.1	LSTRKGSIGSDPRDRVIVAN-QRPSHPD---YEASPSEAGLPAEPVNTRANTLAISEGEG
FG07127.1 	QSRRHSSVASDPHNYVIVGD-RPAVYPE---WEATGLEAGGRAEAVNTRANTLAISEGEG
AN8215.1  	RRRRASSISSLPHNRVIVDKVSNGSSKDSVTHEATAASAGLPAGPPD-RSTTLQISEGLG
          	   * .::.* *:: *** .       :: : **:  .*.  * . : *:.** **** *

MG08171.1 	VLGREATWDDYPNGRWGDARSPAYGEIDGYGVSLHMSGTQAVQLWGRPATVEDVSNIFMR
NCU09545.1	VLGREATWDDFPNGRWGDARSPAYGQIDGYGVSLHVTVPQALQLWGSPKTTEDINKIFIR
FG07127.1 	ALGREATWDDFPNGRFGDARSPAYGEIDGYGVSLHMSVTQAVKLWDYPKTRQDINDLFVK
AN8215.1  	ALGREATWDDFPNGRWGDARSPAFGEIDGYGPSLHVAPSVAHRIWGYPVSTDDISKLFRR
          	.*********:****:*******:*:***** ***:: . * ::*. * : :*:..:* :

MG08171.1 	HLRGALPAIPWSE-------EEFNAETETIRDTLLALNSR-GWWTVASQPAVNGLRSNDR
NCU09545.1	HLKGELSAIPWSE-------EGFNDETEAIRDNLIELNSR-GWWSLASQPAVNGLRSDDG
FG07127.1 	HIQGELSAIPWSE-------EELRAESSTIKPHLLQLNGK-GWWTVASQPAVNGLRSSDA
AN8215.1  	HVSGDLHMVPWSEGGAEENTSGLNAETETIRPELLALIDKKGWWTLASQPAVNGVRSDDP
          	*: * *  :****..:...:. :. *:.:*:  *: * .:.***::********:**.* 

MG08171.1 	TFGWGPQ-NGFVFQKAFVEMFLPSKDWKALREKLLS--EKDVVCFYASNAKGDFESSDGG
NCU09545.1	TFGWGPE-NGFVFQKAFVEFFIPSADWRVLEAKLKQPDMKDTVCFFATNAAGDFVSTDTI
FG07127.1 	TFGWGPP-NGFVFQKSFVEFFIPANEWEILKAKLVSSELQDYVCFYASNARGDYVSSDIG
AN8215.1  	IFGWGPPGEGFVFQKPFVEFFCPASDFKTTLKPLLQKHGHEKLAWFATNAAGDFESSLPA
          	 ***** .:******.***:* *: ::.     * .   :: :.::*:** **: *:   

MG08171.1 	GDAPDGAPTGNGFADEQPLPSGSSTNAVTWGVFPGKEIVTPTIIEEVSFRAWSEEAFGIW
NCU09545.1	NFRARMAAEAEGTAEADHRPHEPSTNAVTWGVFPGKEILTPTIIEEVSFRAWSEEAFGIW
FG07127.1 	G-HVDGSTEAS-----------PSTNAVTWGVFPGKEIITPTIIEEVSFRAWCEEAFGIW
AN8215.1  	ETSDTEPVEMN----------PNNVNAVTWGVFRGKEIVTPTIIEEVSFRAWGDEAYRIW
          	      .   .            ..******** ****:************* :**: **

MG08171.1 	AEWAKVYGKDSPSEKLLESLRADLWLVNIIHHDY------------IEKEALWDLLLK-
NCU09545.1	SEWAKIYGKGSDTQKLLEDLKEDLWLVNVIHHDY------------LEKDGLWKLLSQ-
FG07127.1 	GEWAKVYGRGSESEKLLNGIKDDYWLVNLIHHDY------------VNRDALWEVLSS-
AN8215.1  	DEWRRIYPKSSATEKFLEKTKNDVWLVCVVGQDFGAGTEVGSKEEDDEKKWMWRLLADC
          	 ** ::* :.* ::*:*:  : * *** :: :*:.:.:. .:.... ::. :* :* ..
```
