# Supplementary material for: Patterns of Intron Gain and Loss in Fungi
Source: PLoS Biol. 2004 Nov 30;2(12):e422. doi: 10.1371/journal.pbio.0020422 (PMC532390; doi:10.1371/journal.pbio.0020422)
Supplement: Table S1 — Also available at http://genes.mit.edu/NielsenEtAl/. (4.3 MB ZIP). [file pbio.0020422.st001.zip › NielsenEtAl/html/1031.html]

AN6853.1.NCU03596.1.MG00707.1.FG05890.1


```
 CLUSTAL W (1.82) Multiple Sequence Alignments - Introns Inserted


Sequence 1: NCU03596.1	409 aa
Sequence 2: MG00707.1	403 aa
Sequence 3: FG05890.1	350 aa
Sequence 4: AN6853.1	414 aa
Alignment Length: 503 aa
Number Identitical Residues: 121 aa
Alignment Score (without introns) 7033


MG00707.1 	------------------------------------------------------------
NCU03596.1	------------------------------------------------------------
FG05890.1 	------------------------------------------------------------
AN6853.1  	MSTTPSDAPTPTPATSQPAPPVDGNTVAPDVEKLHVSPEGESTPTPAPASESASAPPKES
          	 :::.:.:.:.:.:::..:.. ...: :.. ..   :....::.:.:.::.::::....:

MG00707.1 	--------------MTTATSNGSAQDKAAVGILKTPISAPLEGCTPPAKDPLTKEQEIKY
NCU03596.1	----------------------------MATVLKVPLASPTPDSQPKPQAALTADQEEKY
FG05890.1 	---------------------MASSAETTSGPRKTPIPTPGPNSQPTPRPELTEEQKTKY
AN6853.1  	LVQEEPAATPAPQQEIETIDSLNLPSSAADGLIQKPFVRPVNTAKPPPPAKLTPEQQAKY
          	  ....:::.:...   : ..     .:     : *:  *   . * .   ** :*: **

MG00707.1 	DWLLEQVKKWTEVPSTK-----GKGGPLTDAERMWLTRECLLRYLRATKWVEKDAEKRLR
NCU03596.1	NWLLEQVKNWKEVPATE-----GKAGPITDREKFWLTRECLLRFLRATKWNQKEAEKRIL
FG05890.1 	EALLEQVKGFTEIECEKQADKEDKSGPITDHERSWLTRECLLRYLRATKWTVDESAKRLK
AN6853.1  	ESVLKSVSGWTTVPTTAAKN--APTAPITDDERMFLTRECLLRYLRATKWNAPEAVARLQ
          	: :*:.*. :. :      .     .*:** *: :********:******   ::  *: 

MG00707.1 	ETLTWRRDFDVADLTWDHISPEQETGKQVILGFDKEGRVCHYLCPGRQNTQPSHRQVEHL
NCU03596.1	GTLTWRREYGVEELTADHISPENETGKQIILGYDKEGRVCHYLNPGRQNTEASPRQVQHL
FG05890.1 	ATLAWRREYGLEGFTPEYISPEQETGKQMIVGFDRQGRPCQYLNPARQNTDTTPRQLHHL
AN6853.1  	RTLTWRREYGIEKLTADYISIENETGKQVLLGYDIHGRPCLYLLPSNQNTEKSDRQVQHL
          	 **:***::.:  :* ::** *:*****:::*:* .** * ** *..***: : **:.**

MG00707.1 	VFMLERVLDLLPAQREKLVLLINFKQGKNRSYTAPGIGQAREVLNILQTHYPERLGRALI
NCU03596.1	VFMLERVIDLMPPQVETLSLLINFKSSKSRSNTAPGIGQAREVLNILQNHYPERLGRALI
FG05890.1 	FYMVERVTDLMPPGVEMLSLMINFKPSKERKNTSVPVSVAREVLHILQNHYPERLGKALI
AN6853.1  	VFMLERAIELMPADQETLALIVDYSQTKSGQNAS--IGQAKDTVHFLQNHYPERLGRALV
          	.:*:**. :*:*.  * * *::::.  *. . ::  :. *::.:::**.*******:**:

MG00707.1 	VNV1PWVVQGFFKLITPFIDPLTRDKLKFNEDMSNYVPKEQLWTEISGGALEFEYDHSTY
NCU03596.1	INV1PWIVNGFFKLITPFIDPNTREKLKFNEDMKKYVPAEQLWTEFNG-SLEFDYDHATY
FG05890.1 	INV1PWIVWGFFKIITPFIDPVTREKLKFNEDMKQYVPPEQLWSLDWGGDMDFEYDHETY
AN6853.1  	INM1PFIIMGFFKIITPFLDPVTREKLKFNENLTNHVPPSQLMKSVGG-DVEFKYDHAAY
          	:*: *::: ****:****:** **:******::.::** .** .   *  ::*.*** :*

MG00707.1 	WPALRKMCEERQVEKEKRWTAGGKQIGELEDYLGGKASQGITPPVAATTEVAA-TATATS
NCU03596.1	WPALQKMCVEKREAKYQRWVAGGQQIGELEDFITGHAQVGVAGPIAAATSAPEPTPAASA
FG05890.1 	WPALNEMCRQKREDKFRRWEAGGKEIGESEDYLAGGTDVSIKG-----------------
AN6853.1  	WPALNKLTELKQKEYRERWIKGGKRIGEYEHYLKTGASPSISQ-----------------
          	****.::   ::    .**  **:.*** *.::   :. .:                   

MG00707.1 	TAPVAAPA-------ATAAASEVAP------IAVAQESAAPE-KPAVEGAKKEESGAVSE
NCU03596.1	PAPAAEPETILAPEPALASETALAPPPVTASVPVATEAHDEAPAPAPEAAAIPEPTPAAV
FG05890.1 	---------------------------------VKFEG-------AKDGVKEVEEKLAAT
AN6853.1  	----------------------------------------------REAEANGSAGPAAD
          	                                               :.    .   .: 

MG00707.1 	KQGDKVEDLK---VDELKVAE---
NCU03596.1	PVEEKKEEEKPKVVASTEVGETTA
FG05890.1 	KLEEQP-------VAA--------
AN6853.1  	KAS---------------------
          	
```
