# Supplementary material for: Patterns of Intron Gain and Loss in Fungi
Source: PLoS Biol. 2004 Nov 30;2(12):e422. doi: 10.1371/journal.pbio.0020422 (PMC532390; doi:10.1371/journal.pbio.0020422)
Supplement: Table S1 — Also available at http://genes.mit.edu/NielsenEtAl/. (4.3 MB ZIP). [file pbio.0020422.st001.zip › NielsenEtAl/html/1032.html]

AN6549.1.NCU03809.1.MG04038.1.FG06100.1


```
 CLUSTAL W (1.82) Multiple Sequence Alignments - Introns Inserted


Sequence 1: NCU03809.1	360 aa
Sequence 2: MG04038.1	328 aa
Sequence 3: FG06100.1	317 aa
Sequence 4: AN6549.1	346 aa
Alignment Length: 394 aa
Number Identitical Residues: 82 aa
Alignment Score (without introns) 4412


MG04038.1 	MAAKPDTSLP---DEIQWNSMEHVMQFG-GIHDNTI~LYYFAASPFFDPTSNNAVVFSQA
NCU03809.1	MAFDPAANAGPPLDEIQWHTPP---QFEAGIHSNSI~LYYFAQSPFYDKTSNNEVVFQQG
FG06100.1 	MSNPNDPPLD----EIQWRSPMAIAQMG-GLHNNTI~LFYFAESPFFERTSNNAVVYNQA
AN6549.1  	MAGTQDPSSL---EEILWRSPSHVQMMGGYLHSNNI1LFYFAESPFFDATSNNASLAIQA
          	*:    .      .** *.:      : . :*.*.* *:*** ***:: ****  :  *.

MG04038.1 	VRNQSQLHIIATRQAFEARLREMSGLEYIVAQEPAETGPGMG-------TGVWVIRKQTR
NCU03809.1	LNNQAMSQYLATRELFESRLKEMSGLEFIVAQEPAETGPGMG-------TGVWVINKQTR
FG06100.1 	MNVPSMYPVIQTREAFETHLNTMSGLEFRVVEEPAETGPGAG-------TGVWVIRKQTR
AN6549.1  	NYNEAFRHFVETREAFEARLKTMQGLEFMVAYDPLQAAAGANAQFVHEPSNVWVIRKQTR
          	    :    : **: **::*. *.***: *. :* ::..* .:.   ..:.****.****

MG04038.1 	RKVKGSN-GFP---APDEIEVHSDYFVVGENIYMAPSLANILSSRI0ASIA---------
NCU03809.1	RKRPPTNPARPEDGPPDEIIVHSVYFVVGENIYMAPTLADVLSSRI0GAIA---------
FG06100.1 	RKSAYDD---------DEITVHASYFVVGENIYMAPTLSGILAARI0MTIS---------
AN6549.1  	RKRSGFE---------DEVVVLATFFVVGDCIYMAPSAASVIGNRI~VCLVALSVSRSAI
          	**    :         **: * : :****: *****: :.::. **   : : : : :: 

MG04038.1 	------------SSITKTFPIADSIKKWSPALGHGYKTPATNSLTS-RPRGTG--LESKE
NCU03809.1	------------TAITKTIPLVDEVSDWAPAVGRRYITPAQPSAGAGAASGTTNYTASRT
FG06100.1 	------------LSITNAVTAAESVRKWGPSRGNYYELPAAKTTTK------AKIQDSAA
AN6549.1  	VAELIAVKLSAVTSLTSLLKTASTLPKFTPSHGHTYLPPAPKSTDVSHPSVQS--QTSKE
          	 :.  : . ::  ::*. .  .. : .: *: *. *  **  :   . .        *  

MG04038.1 	ATPMP-ESQTSSAMTAAKNADR------SDP--DEKLAEESFAIHTHYGTEYMDENPITG
NCU03809.1	ATPLP-DGLPSTATTNKPGAKAGGGTTTNDPLLDSLLMEEALLTHERYGTEYMDENPITG
FG06100.1 	ATPMPPTDEPSKAPIASTPVTQ-----KDEEKELEKLAEESFMIHMKYGGEYIDENPITG
AN6549.1  	NTPMPDADATNKSQSFTGSQNS-----SGPAVYDMRSLAESFSLVARYGDEFMDESPLMG
          	 **:*  . ...:              ..          *::    :** *::**.*: *

MG04038.1 	KPGDFHFSSTGRKERLN-----------VPGQ-QQGK-AIGSGPDTTLPVLKTLPDNSPL
NCU03809.1	KPGDFHLTSTGRKTQTKSALTLKEAAAALPALNTKGLGAAGSNPLAKGAAAAAAANANAV
FG06100.1 	RPGEFHLTTTGRK---------------PPQLVTKDS--------PMRSITAPTINTKIE
AN6549.1  	EPGSFILSRPGDADR---------------GAAPKQS--------QPSSTNAGGRVGTPL
          	.**.* :: .*                       :             .        .  

MG04038.1 	S-KDAKSEKTGKTGGPPKPKRRKSKGG--PNTPS--
NCU03809.1	TGKETKSPKTPGGGGPPKPKRRKSKNV--ITTPGAA
FG06100.1 	DKKDSK-EKTPRSATAPKPKRKKSKMANSTSTPAAS
AN6549.1  	AKVDTPGKLSDKNSAAEEPKLRKKKSK-----PAS-
          	   :: .  :   . . :** :*.*       *.:
```
