# Supplementary material for: Patterns of Intron Gain and Loss in Fungi
Source: PLoS Biol. 2004 Nov 30;2(12):e422. doi: 10.1371/journal.pbio.0020422 (PMC532390; doi:10.1371/journal.pbio.0020422)
Supplement: Table S1 — Also available at http://genes.mit.edu/NielsenEtAl/. (4.3 MB ZIP). [file pbio.0020422.st001.zip › NielsenEtAl/html/1033.html]

AN5591.1.NCU03500.1.MG03940.1.FG05169.1


```
 CLUSTAL W (1.82) Multiple Sequence Alignments - Introns Inserted


Sequence 1: NCU03500.1	538 aa
Sequence 2: MG03940.1	460 aa
Sequence 3: FG05169.1	501 aa
Sequence 4: AN5591.1	481 aa
Alignment Length: 547 aa
Number Identitical Residues: 299 aa
Alignment Score (without introns) 13988


MG03940.1 	----------MFSNRKFSINRNTGVPKP---SRRFSLAEPSAN-----------------
NCU03500.1	MASHQHPPPGAFHNRKFSINRNTGIPRP---ARRFSIQEPSATGMSSSFDSVTNNMFARL
FG05169.1 	---------MSFSGRKFSINRHTGEPKPKMLARRFSTTEPSTN-----------------
AN5591.1  	---------MSFSGRRVSILRPSN--------RRFSVGKELSN-----------------
          	           * .*:.** * :.        ****  :  :.                 

MG03940.1 	----E1AGSKIHRQFRAAHEG~HLPHAGLDPTRASTGVVWCTERATEYGFMEEPEKWANL
NCU03500.1	IRNSE~ASSKIHRKFREAHEG2HLPHAGLDATRSSTGVIWCSEQAGEHGFFEEPEKWANL
FG05169.1 	----E1FQSKVHRQFRSAHEG~HMPHAGLDASRSSTGVIWCTERASEYGYLENPDSWANL
AN5591.1  	---NE1LQSETHRQFRTAHEG~HRPHAGLDASRASTGVVWCTERASEHGFLEDPSGWANL
          	   .*   *: **:** **** * ******.:*:****:**:*:* *:*::*:*. ****

MG03940.1 	GQ~GAPEVEDEIEGCFKRPTSVDVTLNSREYGPTAGIKPLREAVAHLYNEMHRKGQDSLY
NCU03500.1	GQ1GAPEVDDEIEGCFPRPQHLDISVNSREYGPTAGIKPLREAVANLYNEMHRQGKESKY
FG05169.1 	GQ~GAPEVEDDIEGCFPRPETINISMAGREYGPTAGIKPLREAVAKLYNEAHRKGKESQY
AN5591.1  	GQ~GAPEADDEIEGSFPRPETIPITSAAREYGPTAGIKPLRAAVARLYNEHYRQGKESQY
          	** ****.:*:***.* **  : ::  .************* ***.**** :*:*::* *

MG03940.1 	TWENVAIVPGGRAGLIRIAAVLNNSYLSFFIPDYTAYNEMLSLFKD~IAAIPVPLSEDDG
NCU03500.1	TWENVAIVPGGRAGLIRIAAVLNNAYVGFFIPDYTAYNEMLSLFKN~FAAIPVPLSEEDD
FG05169.1 	TWENVAIVPGGRAGLIRIAAVLGNSYLSFFLPDYTAYNEMLSLFKN0FAAIPVPLSEEDG
AN5591.1  	TWENVCIVPGGRAGLIRIAAILGNSYLSFPIPDYSAYSEMLSLFKN~IAPIPMPLAQEDH
          	*****.**************:*.*:*:.* :***:**.*******: :*.**:**:::* 

MG03940.1 	YHINPDKIAEEIARGTGVILTSNPRNPTGRVVSNPELAEIQDLCRDRATFISDEFY1SGY
NCU03500.1	YHIHPEKIAEEIARGTSVIITSNPRNPTGRVVANPELAEIQDICRERATLVSDEFY~SGY
FG05169.1 	YHIHPDKIAEEIARGTSVILTSNPRNPTGRVIQNPELAEIQDLCRERATFISDEFY~SGY
AN5591.1  	YHIHPDKIAEEIARGTSVILTSNPRNPTGHFISGDELAHIQDICRDRATLILDEFY~GGY
          	***:*:**********.**:*********:.: . ***.***:**:***:: **** .**

MG03940.1 	NYTSDCDGTTISAA0ENVLDVDDDD~VLIIDGLTKRFRLPGWRVAWILGPKE~FIKA2IG
NCU03500.1	NYTSNCDGTTISAA~ENVQDVDEDD~VLIIDGLTKRFRLPGWRVAWILGPKE~FIKA~IG
FG05169.1 	NYTSNCDGTTISAA~ENVDDVDEDD~VLIIDGLTKRFRLPGWRIAWILGPKE1YINA2IG
AN5591.1  	NYTTDCDGTTISGA~ANVVDVNKDD1VLLIDGLTKRFRLPGWRIAWVVGPKE~FIDA~LG
          	***::*******.*  ** **:.** **:**************:**::**** :*.* :*

MG03940.1 	SCGSYLDGGTNVAFQEAAIPMLEPSLVKAEMKALQSHFR~DKRDYVVKRLRDMGFTIKFV
NCU03500.1	SCGSYLDGGTNVPFQEAAIPMLDPTLVKAEMKALQRHFC~DKRDFVVGRLREIGFSIKLV
FG05169.1 	SCGSYLDGGASHPFQEAAIPMLEPSLVQNEMIHLQSHFR0DKRDYVVRRLREMGFIIKYV
AN5591.1  	SAGSYLDGGANVPFQEAAIPMLEPSLVHQEMKALQTHFR~EKRDFVLKRLREIGFRIQDV
          	*.*******:. .*********:*:**: **  ** **  :***:*: ***::** *: *

MG03940.1 	PDSTFYL~WLNLEGLP------KPIEDGLNFFQACLEEKVIVVPGIFFDLNPARRRDLFD
NCU03500.1	PDSTFYL~WLNLEWLP------GPISDGLNFFQACLEEKVIVVPGIFFDLNPSRRRDLFD
FG05169.1 	PDSTFYL2WLNLEGLP------ETIADGLNFFQACLEEKVIVVPGIFFDLNPSRRRDLFD
AN5591.1  	PQATFYI~WLDLTSLDPPLPKEANISDGLNFFNALLSEKVIVVPGIFFDLNPAKRRDLFD
          	*::***: **:*  * .. ...  * ******:* *.***************::******

MG03940.1 	SPCHHFVRFSYGPKMETLKLGCDGIERVVKK2----------------------------
NCU03500.1	SPCHHFVRLSYGPTLDILARGCDGIERVVRK~FQALEAEGAYGVPKAPSPSRTEGEKEPH
FG05169.1 	SPCHHFVRLSYGPRMDVLKMGMDAIERVVQN2---CITCGYFHQQIISYLLLSDSLMQLP
AN5591.1  	SPCHHFVRLSYGPKMEVLKMGLDGIERVIRR~-----ARGEHYEPTAMDEQAIED-----
          	********:**** :: *  * *.****::.      : .             ..     

MG03940.1 	-------~----~--------
NCU03500.1	TVPSAGG~GERE~KDEELHLL
FG05169.1 	IRDPFSA1IICS2K-------
AN5591.1  	-------~----~--------
          	
```
