# Supplementary material for: Patterns of Intron Gain and Loss in Fungi
Source: PLoS Biol. 2004 Nov 30;2(12):e422. doi: 10.1371/journal.pbio.0020422 (PMC532390; doi:10.1371/journal.pbio.0020422)
Supplement: Table S1 — Also available at http://genes.mit.edu/NielsenEtAl/. (4.3 MB ZIP). [file pbio.0020422.st001.zip › NielsenEtAl/html/1034.html]

AN3696.1.NCU04783.1.MG04616.1.FG08485.1


```
 CLUSTAL W (1.82) Multiple Sequence Alignments - Introns Inserted


Sequence 1: MG04616.1	587 aa
Sequence 2: FG08485.1	578 aa
Sequence 3: NCU04783.1	596 aa
Sequence 4: AN3696.1	561 aa
Alignment Length: 637 aa
Number Identitical Residues: 239 aa
Alignment Score (without introns) 11973


MG04616.1 	MSRVMRSVKNVTKGYSNVQVKVREA1TSNDPWGPTGTQMSEIAQLTFNS2STEFYEIMDM
NCU04783.1	MSKVIRSVKNVTKGYSAAQVKVRDA1TSNDPWGPTGTQMGQIAQMTYGT2STEFYEIMDM
FG08485.1 	MSKVMRSVKNVTKGYSSAQVKVREA1TSNDPWGPTGTQMSEIAQMTYNT2STEFYEIMDM
AN3696.1  	MSKVVRSVKNVTKGYSAVQVKVRNA1TSNDHWGPTGTEMAEIASLTFGS2PTDFYEIMDM
          	**:*:*********** .*****:* **** ******:*.:**.:*:.: .*:*******

MG04616.1 	IDKRLNDKGKNWRHVLKALKVLDYCLHEGSELVVTWGKQNIYIIRTLREFQYIDEDGRDV
NCU04783.1	LDKRLNDKGKNWRHVLKALKVMDYILHEGSEMVVTWAKQNIFIIKTLREFQYIDEEGKDV
FG08485.1 	IDKRLNDKGKNWRHVLKALKVLDYCLHEGSELVVTWARQSIYIIKTLREFQYVDEEGRDV
AN3696.1  	LDKRLNDKGKNWRHVLKSLKVLDYCLHEGSELVVTWARKNVYIIKTLREFQYIDEDSRDV
          	:****************:***:** ******:****.::.::**:*******:**:.:**

MG04616.1 	GQNV1RVAAKELTSLLND~EERLRAERSDRRSWKSRVTGLEEYAPHHHPDHHGGPSGRGG
NCU04783.1	GNNG~R-EHTPIGGLVFN1EERLREERTDRRAWKSRMQYGEEMPQHHAEPSRPRPR----
FG08485.1 	GQNV1RVAAKELTSLILD~EERLRAERSDRRSWKSRVTGLEEFAPQHAEPVQQANR----
AN3696.1  	GQDV1RVAAKELTALILD~EDRLRSERSDRKLWKSRVNGLDDYHGGHANGLPPPRR----
          	*::  *   . : .*: : *:*** **:**: ****:   ::    *             

MG04616.1 	HREPRRQMNDEDDTEYRLAIEASKHQEEEDRRKREGRQGH-ESDDDDLAKAIKLSKEEED
NCU04783.1	--QHRAQYADEDDAEYKLAIEASKHQEEEDRKRR-ERALA-EVDDDDLAKAIKLSKEEEE
FG08485.1 	RQQPRRQMNEEEDAEYRLALEASKYQEEEDRKKRESRP----DDDDDLAKAIKLSEEEEE
AN3696.1  	--ERRDRRRDDEDAEYRLAIEASKHEAEEERRRRAQAQSNGEEDDEDLAKAIKLSKEEEE
          	  : * :  :::*:**:**:****:: **:*::*      .. **:*********:***:

MG04616.1 	RRRRELEVAANNSSIFDEDLIQVNQ--QPQPTGLNQGYTQGNAVDFFANPIDQNQMQTQP
NCU04783.1	RRRRELE-ETNAAALFDDTPAQTTQ---PQFTGFNQGYQQGSAVDFFANPLDQNQLQAQQ
FG08485.1 	RRRRELE-SSNAASLFDDDPTPSQQTSQPQYTGFNQGYQQGNPVDFFANPIEQN--QPQP
AN3696.1  	LRRRELE-ESNAQSLFDDS-TPAAQ---PQPTGYNQGYQQQSAVDWFGNPINPQ--QPLT
          	 ******  :*  ::**:      *   ** ** **** * ..**:*.**:: :  *.  

MG04616.1 	TGFMNNAYT---GFQ-----PQQTGFPNGYSN--------QGFDPYGQ------QNMQQQ
NCU04783.1	TAYMNNAFT---GYGQQPMGYQQTGYQNGFQNGFQ-PQPTGIYDPYGQ------QQQQQQ
FG08485.1 	TGYMNNAYT---GYQQ----PQPTGFQPNYNTGFGGQQTGMGFDPFGQ------QQQQQP
AN3696.1  	TGYLNNQYANPTGFQG-----QATGMN-GYTNGFQAQPTGYTQNPYAQNNFLQPQATLQP
          	*.::** ::..:*:       * **   .: ..  .  .    :*:.*..  ..*   * 

MG04616.1 	NMQPFQAQ-PTGYNPYAQQQQQQQPPMPMQQS----EPALQAGSNNPWATNNK---PAQQ
NCU04783.1	P-QGFMAQ-PTGYNPYANQQQQQ-PQFDNSSS------TLQPGSNNPWASNNNNNSQQSA
FG08485.1 	QQQGFQPQ-PTGFNPYLQQQQQQQPQQQSFSSPASPGPTLQPGSNNPWATNNN---QQQS
AN3696.1  	QQTGFSTNNPWGTDMFNQQQQHQQPQQQES--------MLTPGSNNPWGNNQP-----ES
          	    * .:.* * : : :***:*.*              * .******..*:      . 

MG04616.1 	QVPMQTGSNNPFAAKPSPFKANSMSTMSTLGSLPEQKTLSSFNSTNQTSPF----QLQQQ
NCU04783.1	IKPMPTGSNNPFAQK-SPYKAP---SMPALSSLPEQKTLSSFPQISVTSSNSPFNTFGSQ
FG08485.1 	LQPTPTGSNNPFAQLGRPQSAR-PNPMSSLGALPEQKSLNSFGNQSQPQP-----QLQQQ
AN3696.1  	LKPMPTGSNNPFAAR-TQLQPQFQASRPATSGAPSLNTLSEERATKQFSN-----QFQSS
          	  *  ********      ..    . .: .. *. ::*..    .  .       : ..

MG04616.1 	NSFSQSS--SFPAS-PQQQTPQKELSEHEARLNQLLANGDGMDTFGNVGQTRIPAQHTAP
NCU04783.1	SQQQQQPQQQQPQFQQQPQQPQRELSEHEQKLNALLASGEGMDTFGNTGNLRIPAQHTAP
FG08485.1 	NSFPMQSQNSFPQSQPSFNAPQKEMSEHETRLNTLLASGDGMDTFGNTGNLRIPAQHTAP
AN3696.1  	TNPIANYQAPQSSQQNQSLAPPKPMDPHTARLNALLATSEGQDTFGNTGDLRIPAQHTAP
          	..   . .   .  . .   * : :. *  :** ***..:* *****.*: *********

MG04616.1 	GTFVNSAGIGAARLTADATGNNPFLRTQYTGMPNVTYGGQQQQQPQMPAATGPAGMGMGA
NCU04783.1	GTFVNSAGAGLNKITTEATGNNPFLKQQFTGLPTISYGG-----QQMPAATGPAGMGMNM
FG08485.1 	GTFINSAGSGAARINAEATGNNPFLRQQFTGMPTVNYG------AQMPAATGPAGMNG--
AN3696.1  	GTFVNSAGQGLDRLRAAHTGNNPFFGQQQQ--------------QFVPQQTG--------
          	***:**** *  :: :  ******:  *                  :*  **        

MG04616.1 	-----NGFGGMGQQSTNNPFAPR--PQQQN---GQGDLIQF
NCU04783.1	GYGGMNGGYQQQQMGSSNPFAAGGQQQQRQQQGGQGDLISF
FG08485.1 	--------------QTNNPFAHQQAPQQQQ---HNNDLIQF
AN3696.1  	-----------YLQQPNNPWGAHQQQPQQG-----GSLIDL
          	               ..**:.      *:      ..**.:
```
