# Supplementary material for: Patterns of Intron Gain and Loss in Fungi
Source: PLoS Biol. 2004 Nov 30;2(12):e422. doi: 10.1371/journal.pbio.0020422 (PMC532390; doi:10.1371/journal.pbio.0020422)
Supplement: Table S1 — Also available at http://genes.mit.edu/NielsenEtAl/. (4.3 MB ZIP). [file pbio.0020422.st001.zip › NielsenEtAl/html/1035.html]

AN2130.1.NCU06500.1.MG00371.1.FG05398.1


```
 CLUSTAL W (1.82) Multiple Sequence Alignments - Introns Inserted


Sequence 1: MG00371.1	1241 aa
Sequence 2: FG05398.1	1208 aa
Sequence 3: NCU06500.1	1218 aa
Sequence 4: AN2130.1	1241 aa
Alignment Length: 1284 aa
Number Identitical Residues: 551 aa
Alignment Score (without introns) 27118


MG00371.1 	MLMTQTVVSALGQAQNSASSIAPN------RHDPTTAAG---STPREDPYL--------A
NCU06500.1	MLMTQTPVSLLQGQNTTSSYNTQSQKTSYDRRDPAAADSSHATTLVDPNDS--------S
FG05398.1 	--MLKIQTAAVPPSIHSSRTIHDP------SGDSKSVSVRITSTPVQASEQ--------Q
AN2130.1  	MYGTRVLDPLAVDQGSAGMNKTKALVTAFEKFEYDYPEKPMANTLAPRSRSRGGSLSFSQ
          	    :   .       :.        :: .  :        :.*        ..: : : 

MG00371.1 	AYTSTSS---TLMNGIVGTAP--PGAMYVRALYDYEADDRTSLSFHEGDVIQVITQLESG
NCU06500.1	FFFATDP--VSSMNGVVGTAP--AGTMYVRALYDYEADDRTSLSFHEGDIIQVITQLESG
FG05398.1 	HSTTRADPAWGLMNGIVGTAP--MGAMYVRALYDYEADDRTSLSFHEGDVIQVITQLESG
AN2130.1  	RDVVRDHSPKRHIPPMKSPSPEPAPALFVRAMYDYDADDHTSLSFRQGDVIQVLNQLETG
          	       ..   :  : ..:*..  :::***:***:***:*****::**:***:.***:*

MG00371.1 	WWDGVING-VRGWFPSNYCEIIN---DVPEP--EPNGFIDSVEEDPEEDDYENGFDDEEE
NCU06500.1	WWDGVING-VRGWFPSNYCEVIASPDDAPGETEEKANPEQVVEEEAED---HDVYHESFD
FG05398.1 	WWDGVING-VRGWFPSNYCQIITSPDDIPDS--AHNGAFDVVEDDVED---PEIYDDQYD
AN2130.1  	WWDGVINGNVRGWFPSNYCAVITDLRELEEH-VSQVQVHDEADISAESGAGEEVEEEHDD
          	********.********** :* .  :            : .: . *..   :  .:  :

MG00371.1 	DDDD-QDLAG---GLPMEQPDSD-SKSGADFWIPQATSDGRLFYYNTMTGQTSVELPLES
NCU06500.1	EDDA-SDRDDDPDSLPIEGTDG--DRSRADFWIPQATPLGHLFYYNTMTGESSNELPLES
FG05398.1 	QDDD-SELDGPI-ALPLEGTDGG-DSSRADFWIPQATPDGRLFYYNMMTGDRSMELPLES
AN2130.1  	DADSPANARDSQPILPIEGMDPPSQQEEAAFWIPQATPDGRLFYFNTLTGYSTMELPFEN
          	: * . :  .    **:*  *  :. . * *******. *:***:* :**  : ***:*.

MG00371.1 	PTSMNESGPRDRMNVNLPDRTRPPPEMMARGLTQDEDEDSDANSASE~LDGESIMMASQT
NCU06500.1	PASVNETGPRDRMNVTIPDRTRPPPEMMARGLTQDEEDEDDITSASE0DNEGLTPIQPK-
FG05398.1 	PVSNNETGPRDRMNVNIPDKTRPPPEMMARGLTQDEDDES-LTSASE~AEGDSARLTTR-
AN2130.1  	P-SANDTGPYDRNNFFVPDQTRPPPELMARGFERDEDDYD--GSASE~AEGESLMLASHD
          	* * *::** ** *. :**:******:****: :**:: .   ****  :     : .: 

MG00371.1 	LG0SN~NRAPHLKDTLSPSISMDSMNGQHPITRTR-GDTFGATQAMNQTPMIAS--ATSF
NCU06500.1	--~--~--NRRLYDGVSPSTSMDSINGQPSGNRGRTETYFN---SNHHIVPMTS--TTAF
FG05398.1 	-R~AK0NRHRSSFGALSPSTSMDSINGASPVGRTRNGASSNGLLSAGQVPNIAS--ATSF
AN2130.1  	-S~MS~RRRQSFIDGVSPATSLDSLHPPSATKSMNEGKSPLGRSPHRPYGSTGAGSNTSI
          	    .        . :**: *:**::   .    .      .  .        :.: *::

MG00371.1 	T--TFNLPSAATIPRSFFDDGSTPPLTWSRLVNNMKRSIERYREAIQHGSRSEYVARAED
NCU06500.1	TSATFNLPTAATIPRSFFDDGSMQPLTWSRLVANMKKAIDRYREAIKNNHRSEYVARAED
FG05398.1 	TSTTYNLPTTATVPRSFFDDGSTPSLSWTLLVSNMRRAIDRYREAIMNNNRSEYVAKAED
AN2130.1  	SEQLHRPSISSRVSQHFFDDHTSTPITWPLLVDNMSQAVEAYRQTLLSGDRAEFVRKAED
          	:.  .. . :: :.: **** :  .::*. ** ** :::: **:::  . *:*:* :***

MG00371.1 	ISDHLRLLLAAGSGTTDNHSGQPSIISTNKALYPHFRDMMSKFSKLVISSHIAAADWPNQ
NCU06500.1	ISDHLRLLLAAGSGTTDNHSGQPSIISTNKALYPHFRDMMSKFSKLVISSHIAAADWPNA
FG05398.1 	ISDHLRLLLAAGSGTTDNHSGQPSIISTNKALYPHFRDMMSKFSKLVISSHIAAADWPNA
AN2130.1  	ISDHLRMLLAAGSDTTDNHSGNPSIISANKPLYQHFRDMMSKFSKLVLSSHMAAADWPGA
          	******:******.*******:*****:**.** *************:***:******. 

MG00371.1 	ESVQKCLSEADGVLLGVFSYVEVARQQRGEEIPRLFPGFVIGSTTGGSWQNNGLAGRDPI
NCU06500.1	ESVQKCLQEADGVLMGVYSYVEVARQQRGEDIPRLFPGFVIGSTSGGSWQTNGLGPHDPI
FG05398.1 	ESIQKCLQEADGVLMGVYSYVEVARQQRGEEIPRLFPGFVIGSTTGGSWQNNGLGPRDEI
AN2130.1  	DAVNKCLQEADGVMQGVYGYVEVARQQRGDYIRRIAPGFVMGSSSGGSWQNNGVSLNDSG
          	::::***.*****: **:.**********: * *: ****:**::*****.**:. .*  

MG00371.1 	TSNFLEDEEG--VVEPTAVLDGKLLERLDELKRLLVSSIRELDKSLILPDK-VISPYRHE
NCU06500.1	TSNFLDDEEG--VVEPTAILDSKLLERLDELKRMLVSGIRELDKSLVVTDK-VVTPFRHE
FG05398.1 	TANFLEDEEG--VVEPTAILDNKMLERLDEQKRILVSSIRELDKSLVMMEK-IVTPYRHE
AN2130.1  	PTSFLDQDGGDLRPEPTVPLEPNVLDHIDVLRRSFVGAIRRVEEQLTLNQKKIVTLAEHE
          	.:.**::: *.   ***. *: ::*:::*  :* :*..**.:::.* : :*.:::  .**

MG00371.1 	VIGNNVCLAGSRVLDTFKPWVAMIESIDLSSLG-NTFQTPQLADFSVNKQSLYDNISDLI
NCU06500.1	VISNNICAAGGKVVDMFKPWIATIESIDLSCLGSNGFQQPQLLDFATNKQSLYDNISDLV
FG05398.1 	VIGNNVCFAGGRVLDTFRQWIAMIESIDLSSLG-NSFQTPQLSDLGTNKQSLYDNISDLI
AN2130.1  	ELSELIAAAAVKVVEQFRPWVSAVESINLGPLG-TSFQNPQLIDFSSQKQRVYDAIADFV
          	 :.: :. *. :*:: *: *:: :***:*. ** . ** *** *:. :** :** *:*::

MG00371.1 	LGCQAVSGPLSDEWSEVRGESLENRLEYVRQCGRTLETNSSHVGFSLQLLSEQVQFNMQ-
NCU06500.1	LGCQAVAGPLADEWSEVRGHALEERLDYVRQCARALETNSSHIGFSLQLLSEQVQMVMQ-
FG05398.1 	LGCQAVAGPLADEWSEVRGEALENRLEYVRQCARALETNSSHIGFSLQLLSEQVQINMQS
AN2130.1  	VSCQAVSAPLGDEWAELRGDSLEDRLNAVRGVARQLENYVSQIGFSLSLLLEQVPETPT-
          	:.****:.**.***:*:**.:**:**: **  .* **.  *::****.** ***      

MG00371.1 	QQVEPRVREEPFVRQPLQRTETMPYQPSGHLRTESRGISAPQRPGYAQSVSVDGADLIAP
NCU06500.1	QQAEARAR-EPMQRTPLSRGDSLPYERH--LRTDSA----FLRPVLMSSASFSEGDPTAP
FG05398.1 	HQIEPRPRENSFVRSTLQRGETMPYDRP-HQRTESR--TAPVRPPLITSQSFTEGEPAPG
AN2130.1  	LRSESRIGGDSDGYMGLHS------------RSESR--STEVGVPSAHPLDGSPEKMRRN
          	 : *.*   :.     *              *::*   ::        . .    .    

MG00371.1 	NNKFRKIGGHSKLERIFGEDPSPQPPPVDETPEFLKLSYEDDIQWETKTQPPTVKGGTLL
NCU06500.1	T--FQRKGDPSKMKKFFGEDPTPIQP-VDDTPEFLRLDYEAELSWDQKVQPPVVKGGSLL
FG05398.1 	N---MRRGDYSKVKKIFGEDPSPQVPVTEDTPEFLLLDYEHEVSWDSKTSPPTVKGGSLL
AN2130.1  	MDKAQRFFGQAPPAAITREPIREIAREPEETPWFLKMDHEGEVFYDTKNDVPTLKCGTLA
          	 ..  :  . :    :  *         ::** ** :.:* :: :: * . *.:* *:* 

MG00371.1 	ALVEQLTRHDKLDSSFNNTFLLTYRSFTTARELFELLVKRFGIQPPEGLNQQDYDVWRDR
NCU06500.1	ALVEQLTRHDKLDSNFNNTFLLTYKSFTSARVLFELLVKRFGIQPPEGLTQTQYEQWRDS
FG05398.1 	ALVEQLTRHDKLDSNFNNTFLLTYRSFTTARELFEMLIKRFNIQPPEGLNPIDFEIWRDR
AN2130.1  	GLVEHLTRHDKPDMSFNSTFLLTYRSFTTASELFEMIMQRFNIQPPFGLNADEMQMWVDR
          	.***:****** * .**.******:***:*  ***::::**.**** **.  : : * * 

MG00371.1 	KQKLIRFRVVNILKSWFENFWMEDSSLEETKQLIRDVYTFARDTVKSTETPGSPQLMALL
NCU06500.1	KQKLIRFRVVNILKNWFDNFWMEDQS-EETKQLILDVYNFVNETVKSTETPGSKGLMAVL
FG05398.1 	KQKLIRFRVVNILKNWFDNFWMEDHN-DESKQLVRDVYSFAKDTVKSTETPGSAPLMAVL
AN2130.1  	KQKPIRFRVVNILKTWFENYWMEPND-ESHMQLLERAHSFTKDSIATTKTPGSTQLLAVI
          	*** **********.**:*:***  . :.  **:  .:.*..::: :*:****  *:*::

MG00371.1 	DQRLTGKDVSARRMVQNSNLPTPTPIMPKNMKKLKFLDIDVIEFARQLTIIESRLYAKIK
NCU06500.1	EQRRSGKEVNVRRMIQTVNQNTPAPIMPKNMKKLKFLDIDVTEFARQLTIIESRLYGKIK
FG05398.1 	DQRLSGKDAGARRMIQTVNQNTPTPIMPKNMKKLKFLDIDVIEFARQLTIVESRLYGKVK
AN2130.1  	EQRLRGQDTTVKRLVPTQATPAPTPIIPKNMKKLKFLDIDCTEFARQLTIIESRLYSKIK
          	:**  *::. .:*:: .    :*:**:*************  ********:*****.*:*

MG00371.1 	PTECLNKTWQKKQGEGEPEPAPNVKALILHSNQMTNWVAEMILAQSDVKKRVVVIRHFVA
NCU06500.1	STECLNKTWQKKVAEGEPEPAPNVKALILHSNQMTNWVAEMILAQTDVRKRVVVIKHFVA
FG05398.1 	ATECLNKTWQKKIPEGDPDLAPNVKALILHSNQMTNWVAEMILAQMEVKKRVVVIKHFVS
AN2130.1  	PTECLNKTWQKKVGPDEPEPAANVKALILHSNQLTNWVAEMILNQSDVKKRVVVIKHFVN
          	.***********   .:*: *.***********:********* * :*:******:*** 

MG00371.1 	VAD0RCRSLNNFSTLTSIISALGTAPIARLKRTWDIVPAKAQSTLESMRKLMASTKNFGE
NCU06500.1	VAD~KCRALNNFSTLTSIISALGTAPIARLKRTWDQIPQRVLATLETMRKLMASTKNFGE
FG05398.1 	VAD0RCRVLNNFSTLTSIISALGTAPIARLKRTWDQVPQRTHATLESMRRVMASTKNFGE
AN2130.1  	VAD0KCRALNNYSTLTSIISALGTAPIHRLSRTWAQVSGRTSAILEQMRRLMASTKNFGE
          	*** :** ***:*************** **.***  :. :. : ** **::*********

MG00371.1 	YREALHASNPPCIPFFG1VYLTDLTFIEDGIPSIIKKTNLINFAKRAKTAEVIRDIQQYQ
NCU06500.1	YREALHLSNPPCIPFFG1VYLTDLTFIEDGIPSVLKKTNQINFAKRAKTADVIGDIQQYQ
FG05398.1 	YREALHAANPPCIPFFG1VYLTDLTFIEDGIPSIIKKTNLINFAKRAKTAEVIRDIQQYQ
AN2130.1  	YREALHLANPPCIPFFG1VYLTDLTFIEDGIPSLTP-SELINFNKRTKTAEVIRDIQQYQ
          	****** :********* ***************:   :: *** **:***:** ******

MG00371.1 	AVVYSLQPVNELQDYILSNMSAAGDVHEMYDKSLQVEPREREDEKIVS2MCVTKGFSPSL
NCU06500.1	NVAYSLQPVPELQEYILSNMQAAGDVHEMYDKSLQIEPREREDEKIVS2TRYKLFVKLN-
FG05398.1 	NVGYSLQPVPELQDYILNNMQAAGDVHEMYDKSLQVEPREREDEKIVR2VLAESGFL---
AN2130.1  	NSPYLLTPVTELQEYILSNLQGAGDVHDMYDRSLEVEPREREDEKIAR~YATGGRDMSAL
          	   * * ** ***:***.*:..*****:***:**::**********.             

MG00371.1 	GSCERPDAICRALKRGDILRRQTTRLVVGW
NCU06500.1	----AAEQLWSPFKT---------------
FG05398.1 	------------------------------
AN2130.1  	TFGTLLMSSR--------------------
          	
```
