# Supplementary material for: Patterns of Intron Gain and Loss in Fungi
Source: PLoS Biol. 2004 Nov 30;2(12):e422. doi: 10.1371/journal.pbio.0020422 (PMC532390; doi:10.1371/journal.pbio.0020422)
Supplement: Table S1 — Also available at http://genes.mit.edu/NielsenEtAl/. (4.3 MB ZIP). [file pbio.0020422.st001.zip › NielsenEtAl/html/1036.html]

AN3641.1.NCU02702.1.MG04446.1.FG01018.1


```
 CLUSTAL W (1.82) Multiple Sequence Alignments - Introns Inserted


Sequence 1: MG04446.1	252 aa
Sequence 2: FG01018.1	232 aa
Sequence 3: NCU02702.1	238 aa
Sequence 4: AN3641.1	251 aa
Alignment Length: 272 aa
Number Identitical Residues: 84 aa
Alignment Score (without introns) 3966


MG04446.1 	MLSYENPTPAAPSASTAAEKLATSLSSSARASKVSGSVPDNSVSQSSMASTMKATGSSAS
NCU02702.1	MLSYN------TPISVNGQNAGPTAGMSARPPFKT----------VGNNGSANGVSSNDA
FG01018.1 	MLTYN-------STSTMPIKAPTKPVSIALSPTTS----------SPMT---AATPSLNS
AN3641.1  	MLSAS--------ERNPAVMDPSRTKVSALDLNHS-----------HSHTTTNQISSSSE
          	**: .                 .     *     :               :     *   

MG04446.1 	AASNSASDRSAAHIWLVTGPAGCGKSTVAQHLATSLDVPYIEGDE0YHPPANIEKMSNGI
NCU02702.1	VSGTGRHAPEEQHIWLVTGPAGCGKSTVARYLAESLHWPYIEGDE0FHPPANIEKMSAGI
FG01018.1 	AAINGHKTQGQQHIWLVTGPAGCGKTTVAEYLAKSLGMPYVEGDA0FHPTANIEKMANGI
AN3641.1  	QQASFMPSQCPQHIWVVTGPAGSGKSTVGRYLQQELGVPFLEGDD0FHPAANKAKMSAGT
          	   .        ***:******.**:**..:*  .*  *::***  :**.**  **: * 

MG04446.1 	PLTDMDRWDWLILLRDEALRRLGEGG-------------SDTKGVVVTCSALKRKYRDVI
NCU02702.1	PLTDADRWDWLTALREASIRALDQG----------------NSGVVLTCSALKRKYRDVI
FG01018.1 	PLTDEDRWDWLTALREESINRLNAG----------------SCGVVLTCSALKRKYRDVI
AN3641.1  	PLTDADRWDWLISLRSAATTLLSTPAPTSTNPTSTSTRAQAPTGVVVACSALKKKYRDVM
          	**** ******  **. :   *.  ..:::..::::: :.   ***::*****:*****:

MG04446.1 	RVARYFEPSVHVHFIYLAATEEALLERVARRQNHYMGANMVRSQFQDLEPPRPDETDIIS
NCU02702.1	RVAPYFTPNLHLHFIYLDASEEILLQRVLARQNHYMGANMVHSQFEALEPPTPAETDVIR
FG01018.1 	RVAGYYDRRIQIHFVFLDAPEELLLARVTQRQNHYMGANMVHSQFDILERPLADEKDVIT
AN3641.1  	RVAAYGSPNVRIHFVYLKLEPATLYARVSARQAHYMKQGMVESQLRDLEEPGQGEWDVIT
          	*** *    :::**::*      *  **  ** ***  .**.**:  ** *   * *:* 

MG04446.1 	IDVSG--TLDDVKTSALDKVREVMAADQ-----
NCU02702.1	IDVSR--PADMVMADALNQVLHTIDGIQKDQSQ
FG01018.1 	IDVSR--PIEVVEQEALSNVLETMAKSQDKL--
AN3641.1  	VPVQVGMGMGEVQREVMNAVEKVIRGYEGGL--
          	: *. .     *  ..:. * ..:   :
```
