# Supplementary material for: Patterns of Intron Gain and Loss in Fungi
Source: PLoS Biol. 2004 Nov 30;2(12):e422. doi: 10.1371/journal.pbio.0020422 (PMC532390; doi:10.1371/journal.pbio.0020422)
Supplement: Table S1 — Also available at http://genes.mit.edu/NielsenEtAl/. (4.3 MB ZIP). [file pbio.0020422.st001.zip › NielsenEtAl/html/1037.html]

AN5200.1.NCU06924.1.MG11020.1.FG01395.1


```
 CLUSTAL W (1.82) Multiple Sequence Alignments - Introns Inserted


Sequence 1: NCU06924.1	512 aa
Sequence 2: FG01395.1	485 aa
Sequence 3: AN5200.1	478 aa
Sequence 4: MG11020.1	505 aa
Alignment Length: 551 aa
Number Identitical Residues: 133 aa
Alignment Score (without introns) 8012


MG11020.1 	------MKIVICGAGPVGALAAIYAARRGHEVEIYELRGD1TALEETEVTRQLGKSINLT
NCU06924.1	-MEERKQKVVVVGAGPVGSLAALYAANRGHDVEIYELRGD1LR-DPSTTPLNFTRSINLA
FG01395.1 	--MEKPQKIVVVGAGPVGSLAALYAAQRGHEVEVYELRPD1LR-DPSTIPLNFTKSINLA
AN5200.1  	MASPAKQKVVIVGAGPVGCLAALYAAARGDDVELYELRGD1LR-VPGTIPLNFTKSINLS
          	       *:*: ******.***:*** **.:**:**** *         . :: :****:

MG11020.1 	MSDRGFESLRDSGDVDLVGDVVQGTLAVHMREVHRASSISCQKSTRMRYDANGK0HLNVV
NCU06924.1	LSERGLNAMRHANQPRLIDYVKGVTIPMRGRMIHGKRPDGKLYEEAQDYDIHGR0SILAI
FG01395.1 	ISERGINAMRHAGQPGLLDHVMSTTIPMRGRMIHGRSPTGALFEQSQDYDVKGR0AIHAI
AN5200.1  	LSHRGITALRHSGREHVINEILQEVVPIYG------------------------~-----
          	:*.**: ::*.:.   ::. :   .:.:                                

MG11020.1 	S~RGTLRDILLERVRKLPNTKIFFNSKLVHADLNARFAVFSD----STVKEAWGAEHQYK
NCU06924.1	D~RGDLNKRLLDMLEEMPNVTFFFNHKLTGADFKRNKAWFEN---KDESTSNPRDRAREI
FG01395.1 	D~RAGLNKRLLDVLDNMPNVKLFFNHKLTGADYRACKAWFEV---ADGKASK-ESRYKEI
AN5200.1  	H1RGMLNNVFLNELERIPNIKLFFNHKLTGADFQANKAWFERRLPGEAPLPGSSGRVPEI
          	  *. *.. :*: : .:** .:*** **. ** .   * *.   . .        .    

MG11020.1 	RVSFDLLIGADGAHSAVRHQMARYTHMNLTQTWLDTWWCEFHISPRPGVH-----MEPRL
NCU06924.1	EVDFDFMIGADGAHSAVRYHLMKFSRMDYQQEYIDTLWCEFQIAPSSSS----AKSKFRI
FG01395.1 	DISFNLMIGADGAHSAVRYHLMKFTRMNYHQEYIDTLWCEFHLKPVQTDNTADPMAKFRM
AN5200.1  	EVDFDFLIGADGAHSATRYHMMKFARVDYQQEYIDTLWCEFRIPPSPTN-------DFLI
          	 :.*:::*********.*::: ::::::  * ::** ****:: *           .  :

MG11020.1 	ASDALHIWPEADFMFLAMPNR~DGSFTCNLFAPRAVFDDLAVRAGGGGGKDHAAAEAALT
NCU06924.1	SPNHLHIWPGKEFMFIAIPSN~DGSFTCTLFAPAAIYEQLEEAGRTGD------TSSSIP
FG01395.1 	SPNHLHIWPGKDFMFIAIPSD0DGSFTCTLFMPSKDFSDLESN------------PASVP
AN5200.1  	SPSHLHIWPGKEFMFIALPSV~DKSFTCTLFAPASHYAQLERS------------TEDLL
          	:.. *****  :***:*:*.  * ****.** *   : :*                  : 

MG11020.1 	DFFQRHFPGIVPELMTAAELRVQFSRTTPSALHDMRVSKVNHGDRCVLVGDAAHAMVPFY
NCU06924.1	EFFDMHFPGVT-SLIAPADLIAQFQTNPHLPLISIKCKPYHFSSSVVIVGDAAHAMVPFY
FG01395.1 	AFFDSHFPGVT-DLIPGDELVESFNTNPHLPLVSVKCKPYHYGSSCIIVGDAAHAMVPFY
AN5200.1  	QFFDEHFPGVCPQLISPSDLTAQFRANPHLPLISIKCAPHHYSSSVVIVGDAAHAVLPFY
          	 **: ****: ..*:.  :*  .*  ..  .* .::    :...  ::*******::***

MG11020.1 	GQGLNVGLEDVRILFTEYLCCPSSPAPAATAWLAEKGGPNVGRGSPTTATTTKP---SMA
NCU06924.1	GQGMNAGLEDVRILF---------------DILDKHDRMTN-DDSSLEAS-------QRE
FG01395.1 	GQGMNAGMEDVRILF---------------SILDKHSHIDESNNPSSESSSASGPAFQRA
AN5200.1  	GQGLNAGLEDIQVLF---------------DALDKHGVYNANSDQAARAL-------ARQ
          	***:*.*:**:::**                 * ::.    . . .  :           

MG11020.1 	ESLSAYSARRQPDVAVMSTLALRNYGEMRHGGTAAKW-ARRAIEEALQVWVPSAGWRTLY
NCU06924.1	LALAEYSAVRVADAHAINDLALQNYIEMRSSVLSPVYRWRKALEEWLSVYVPSLGWQTKY
FG01395.1 	LALAEYSAVRPPDAHAINDLALQNYVEMRSSVLSKRYRLRKYLEEWMSVYFPRLGWQTKY
AN5200.1  	SAFAAYTASRTADAHAINDLSRQNYVEMRWGVKQPLYRLRKYIEEALYHYLPSLGWQTQY
          	 ::: *:* * .*. .:. *: :** *** .     :  *: :** :  :.*  **:* *

MG11020.1 	AR~VAFST-----------------------ESFVEIERKN----ARQGWILTAWAFLLL
NCU06924.1	SR~VSFGNERYSEVVKKSERQGQVLLRSLVGGVGLPMLAGGLFLWFRYKGALGRAAYGVF
FG01395.1 	SR0QCFAT--------------------VIGGQYPPCSK-------QSDNYPNSETLSDF
AN5200.1  	TR~VSFSNQRYSEIIAINRRQGRILG-AVFGSTLISVLAVTG-IYLWRQPTTRLLSLASF
          	:*  .*...  :.    .  ..     :  .                        :   :

MG11020.1 	LAVVAALVRFMLCHYC
NCU06924.1	YNCMGMVCRTIHGRRR
FG01395.1 	YSPS----YKLSSRCS
AN5200.1  	RGALQGALQGALTGTA
          	
```
