# Supplementary material for: Patterns of Intron Gain and Loss in Fungi
Source: PLoS Biol. 2004 Nov 30;2(12):e422. doi: 10.1371/journal.pbio.0020422 (PMC532390; doi:10.1371/journal.pbio.0020422)
Supplement: Table S1 — Also available at http://genes.mit.edu/NielsenEtAl/. (4.3 MB ZIP). [file pbio.0020422.st001.zip › NielsenEtAl/html/1038.html]

AN0943.1.NCU00644.1.MG01232.1.FG00656.1


```
 CLUSTAL W (1.82) Multiple Sequence Alignments - Introns Inserted


Sequence 1: NCU00644.1	212 aa
Sequence 2: MG01232.1	196 aa
Sequence 3: FG00656.1	186 aa
Sequence 4: AN0943.1	196 aa
Alignment Length: 225 aa
Number Identitical Residues: 62 aa
Alignment Score (without introns) 3424


MG01232.1 	MSFAIAMRRVALAPSTRTVAARRFESSA---ASKAQSTAKDTANKAAQGLTRVASAAGPA
NCU00644.1	MSFALVSRRSALTFGRR---AVRFESTT---SEKATEAAKKTAAKASEGLSRVSSAAGPV
FG00656.1 	--MSSLARPMLRSPALR-VAARRFESTT---AQKAAENAKQAATRAQEGLSRVTSTAGPA
AN0943.1  	-MPATATRAVLRQSQFLTRTAVRHSSSTSQATSKATETASSTASKAQQGLSRVSSSAGPA
          	   :   *         : :* *..*:::.::.** . *..:* :* :**:**:*:***.

MG01232.1 	ITNAAKGASDALGKVGGRTGKLIKFVER1QVPFVIYYTKVGVEVAKIVFRGQQMTPP2--
NCU00644.1	LAKYAKTLQSTLGRVGGRTGKLIAFAER1QTPFVIYYSKVAAELGRIVFRGQSMTPP~YV
FG00656.1 	IAGYAKGVASTLGKVGGRTGKIIGFVER1QVPFVVYYSKVGLELGKFVFHNQKMSPP2--
AN0943.1  	ISNAAQGLGNTLKKVGGRTGKVVSFIES1MIPPTIYYSRVGLELGKLVFRGQNMTPP2--
          	::  *:   .:* :*******:: * *    * .:**::*. *:.::**:.*.*:**   

MG01232.1 	-----------------------SMQTFQNYFQNVWKQVQNPQALMRQLSSKVPTQNPAE
NCU00644.1	PSSAPRLPMLGNLWVVDMPLHHSSVSTFKAYFQNFVKSVQSGSFFS---NAGASLQN---
FG00656.1 	-----------------------NMATFQTTYQNLIKSIQNRTIIQ---SSQNLVQQ---
AN0943.1  	-----------------------SSATFQSYFQPLINALRNPASLQ---NANFSPQNILA
          	                       .  **:  :* . : ::.   :    .:    *:   

MG01232.1 	SVKNISAAQWTAAGVLGAELLGFFTVGEIIGRMKLVGYHGEVEHHH-
NCU00644.1	-VRNISRTQVAAAGVLLAECLGFFTIGEMIGRMKLIGYHGETHAAAH
FG00656.1 	-VRNIGPAQLAAGGVVAAEVLGFFTVGEMIGRFKLVGYRGEVSSHH-
AN0943.1  	RVRNANKKEIALAGVTAAEVIGFFTVGEIIGRFNIVGYRGEAGHGHH
          	 *:* .  : : .**  ** :****:**:***::::**:**.
```
