# Supplementary material for: Patterns of Intron Gain and Loss in Fungi
Source: PLoS Biol. 2004 Nov 30;2(12):e422. doi: 10.1371/journal.pbio.0020422 (PMC532390; doi:10.1371/journal.pbio.0020422)
Supplement: Table S1 — Also available at http://genes.mit.edu/NielsenEtAl/. (4.3 MB ZIP). [file pbio.0020422.st001.zip › NielsenEtAl/html/1039.html]

AN1357.1.NCU03880.1.MG01003.1.FG10005.1


```
 CLUSTAL W (1.82) Multiple Sequence Alignments - Introns Inserted


Sequence 1: NCU03880.1	534 aa
Sequence 2: MG01003.1	455 aa
Sequence 3: FG10005.1	432 aa
Sequence 4: AN1357.1	472 aa
Alignment Length: 541 aa
Number Identitical Residues: 205 aa
Alignment Score (without introns) 9483


MG01003.1 	----MSSTTQAAAN-KRKRLTGGS--------TTSLPDEVAAAERAQ----ISESRDASA
NCU03880.1	MSPSLNSSTPQASG-KRKRSSAAADSIANSNINTTHLDPVDAAIMNQDQIIQTESRDASA
FG10005.1 	----MSSPSGSSQGGKRKR-------------NSLRVESTHDFAENG---IQTSSRDASG
AN1357.1  	----MSSP---LSNSKRKR------------ADSQHLSTADIAKSSTTDLLQPSSRDASG
          	    :.*.     ..****              :   . .        .   ..*****.

MG01003.1 	EEGD-TTAAETPATAP---------TSHPPIKRQRSSNN--AVGDAT---------VDLG
NCU03880.1	EEGD-TTAPESSRTAAGHRKTDSATSSHPPSKRQRPNNDQLSEGPATTDAIEQAQAIDPG
FG10005.1 	EEGD-TTAAESGRLHAR--------GSAPIPKRQRSSSN----RDNT---------IDPG
AN1357.1  	EEGDESTGPIISPVKAS--------NNPPPKRARKASVSEGQSGDAGKDTSISK--EDPG
          	****.:*..      .          . *  : ::.. ..       ..:  .    * *

MG01003.1 	EPSDTTEASVDIENRVGRKGRK-----SSNPEKEEES-----------MAPPPIGKLTHP
NCU03880.1	EPSDTTEASDHIAERVTRKSSRKATVGQNNGSEDGETGGYPIISKGPAMAPPPIGKLTHP
FG10005.1 	EPSDTTEASIDIAERVGRKGRQ----ALKELDEEEQR-------RIEAMPPPPIGNLQDP
AN1357.1  	EPSETTPASSDIETHTKTRPGL-----HLNTKPDEEL-----------MKPPVLGKLQDP
          	***:** ** .*  :.  :          : . : :            * ** :*:* .*

MG01003.1 	VG-YRTNDPPVGRPVRIYADGVFDLFHLG2HMRQLEQAKKAFPDVYLIVGVTGDAETHKR
NCU03880.1	VG-YKTNPPPVGRPVRVYADGVFDLFHLG2HMRQLEQAKKAFPEVYLLVGVTGDEDTHKR
FG10005.1 	AGGYKTNPPPVGRPVRVYADGVFDLFHLG2HMRQLEQAKKAFPDTTLVVGVTGDHETHKR
AN1357.1  	AGGYKTNPPPVGRPVRVYADGVFDLFHVG2HMRQLEQAKKAFPDVYLIVGVTGDKETHER
          	.*.*:** ********:**********:* *************:. *:****** :**:*

MG01003.1 	KGLTVLSGKERAETVRHCKWVDEVIENCPWIVTPEFLEEHKLDYVAHDDIPYGADEGDDI
NCU03880.1	KGLTVLSGKERAETVRHCKWVDEVIEDCPWIVTPEFLEEHKIDYVAHDDIPYGADEGDDI
FG10005.1 	KGLTVMSAAERAETLRHCKWVDEVIEDCPWVVTPEFLDENKLDYVAHDDLPYGADEGDDI
AN1357.1  	KGLTVLSGAERAESVRHCKWVDEVFPNCPWIVTPEFMEEHKIDYVAHDDLPYGAAEGDDI
          	*****:*. ****::*********: :***:*****::*:*:*******:**** *****

MG01003.1 	YGPIKAEGKFLVTQRTEGVSTTGIITK2IVRDYEKYIARQLKRGTSRQELNVSWLKKNEL
NCU03880.1	YAPIKAAGKFLVTQRTEGVSTTGIITK2IVRDYEKYIARQFKRGASRQELNVSWLKKNEL
FG10005.1 	YQPIKAAGKFLVTQRTEGVSTTGLITR2IVRDYEKYIARQFKRGTSRQELNVSWLKKNEL
AN1357.1  	YAPIKAQGKFLVTQRTEGVSTTGVITR2IVRDYDRYISRQFKRGASRQELNVSWLKKNEL
          	* **** ****************:**: *****::**:**:***:***************

MG01003.1 	DLKRHVQDLRENIRSNWTTTGQELSRELRQYWPASRPQSPAPS----------------R
NCU03880.1	DLKRHIQDLRDNIRSNWATTGQELSKELRQFWPSSRPQSPSPSGFPRLPPQLLAMAQNGG
FG10005.1 	DLKRHVQDLRENITNNWSTTGQELGRELKQFWPVSRPQSPARFN----------------
AN1357.1  	EIKRHVSELRDSIMTNWTNTGQELSRELRQLWN-SRPNSPAPST---------------R
          	::***:.:**:.* .**:.*****.:**:* *  ***:**:                   

MG01003.1 	PSLQVPNGDQ-----LTSPQQQSSLREAQLRSPTTPGG-ANVNSNDFVAGYAVGLIGGVR
NCU03880.1	SSADVSNATSPSPLGPASGSAQAGPSNLAPKSPTQAAAQAAGNVNDFITGYTLGLVGGVR
FG10005.1 	----------------SAGTAEG------LRSPTTPGT--SGTPKEFITGYALGLVGGVR
AN1357.1  	TSMDWGSSRG-----VVSPTAGGKSHVSRVEALGRTES-ITGREPDFATGYSLGLIGGVR
          	.: .  ..         :    .       .:   .         :* :**::**:****

MG01003.1 	SW0MTKSRRSLRDERDDSSSRPPSDDSSEESEGKEKTTRTRRVVPKGNNAAATGA-----
NCU03880.1	SW0MSRTRGPSQED----GSRAPSDDDSEEGDKKKSNEKRRSNVPAGSGAASASASTSLA
FG10005.1 	GW0MTKSRTNVADG-----SRPPSDDESEESDAHAKSPKESSNTPTAVAPSKL-------
AN1357.1  	AW0KAEASHQPVKKNTSLNWNAATVRDPPNLSGKLAVHRMCRTGEVTFMIFIFEAILGHI
          	.*  :.:     .   . . ...:  .. : . :    :               :  .  

MG01003.1 	----
NCU03880.1	QRRV
FG10005.1 	----
AN1357.1  	GT--
          	
```
