# Supplementary material for: Patterns of Intron Gain and Loss in Fungi
Source: PLoS Biol. 2004 Nov 30;2(12):e422. doi: 10.1371/journal.pbio.0020422 (PMC532390; doi:10.1371/journal.pbio.0020422)
Supplement: Table S1 — Also available at http://genes.mit.edu/NielsenEtAl/. (4.3 MB ZIP). [file pbio.0020422.st001.zip › NielsenEtAl/html/104.html]

AN7299.1.NCU00422.1.MG06316.1.FG06955.1


```
 CLUSTAL W (1.82) Multiple Sequence Alignments - Introns Inserted


Sequence 1: MG06316.1	417 aa
Sequence 2: FG06955.1	405 aa
Sequence 3: NCU00422.1	410 aa
Sequence 4: AN7299.1	403 aa
Alignment Length: 419 aa
Number Identitical Residues: 229 aa
Alignment Score (without introns) 10649


MG06316.1 	MASEDKEIA1FPPD~YTLNNPDTLTKYKTAAQISEKVLKDVSA~LCVAGAKIVEICEKGD
NCU00422.1	MASETTKQ-~-EID1YSLNNPDTLTKYKTAAQISEKVLAEVSK~LVAAGEKIVDICEKGD
FG06955.1 	MSSENKEV-~---D1YSLANPDTLTKYKTAAQISEKVLAEVSK~LVVPGAKIVDICQQGD
AN7299.1  	MAENQTQTP~-EVD1YTLNNPDTLTKYKTAAQISHKVLEAVSA1QCVEGAKIIELCQQGD
          	*:.: .: .    * *:* ***************.***  **    . * **:::*::**

MG06316.1 	QLIEEEVAKVYRGKKITKG1FSHPTTVSPSSYVTPYTPLKTDEAEAATELKAGEAVKIQL
NCU00422.1	KLIEEELAKVYRGKKITKG~FSHPTTVSPAAFVTPYTPLTSDEKEAAVEIQAGEPVKIQL
FG06955.1 	KLIEEEISKVYRGKKINKG1FSHPTTVSPSSYVTPYTPLTSDEAEAGAEIKDGEAIKIQL
AN7299.1  	KLLEEELAKVYKGKKIQKG1IGHPTTVSPNSHVTPYTPLVSETAEAETTLKAGEIAKIQL
          	:*:***::***:**** ** :.******* :.******* ::  ** . :: **  ****

MG06316.1 	GAQIDGFGSIVCDTILVPKEGEG--VVTGRDADLMLANYYANELLLRLMVPPGLLAQGTD
NCU00422.1	GAQIDGFGSIVCDTVVAPAKDQTDDVIEGRNADLMLANYYANELLLRLMVPPGLLATGTD
FG06955.1 	GAQIDGFGSIVCDTIIATPEDKAGDKITGRTADLILANYYINEVLLRLMIPPGTLAQGSD
AN7299.1  	GAQIDGFGTIVCDMVVVGA-----SEVTGREADLIHATYYANELLLRLMAPPGLLATGSD
          	********:**** ::.       . : ** ***: *.** **:***** *** ** *:*

MG06316.1 	EEKAKAAAAKPHTQSKITSLLEKVAKAYEVNLVESTTSWLFGRNEIEGTKKIVIAPGEGA
NCU00422.1	EEKAKAASQKPPSQAKITELLQKVVQAYDCNLVESTTSWLFDRNEIEGKKKIVIAPGDNT
FG06955.1 	EEKAKAAAQKAPTQAKITSLLEKVAKTYEVNIVESTTSWLFDHNEIEGSKKIVLSPAEGT
AN7299.1  	EEKKKAAAERPPTQARITQLIEKIAKTYDCTIVENTTSWLFERNEIEAEKKIILSPGSGV
          	*** ***: :. :*::**.*::*:.::*: .:**.****** :****. ***:::*....

MG06316.1 	KGEGVPEVGEVWGVEMGVSLGSGKVKQLDQRATLHRRTTTTYGLKRPTSRKILSEVQKKF
NCU00422.1	KGEGIPEVGEVWGVEMGVSLGSGKVKQFENRTTLHRRTTTTYALKRPSSRKLLSEVQKKF
FG06955.1 	KGEGVPEIGEVWGVEVGVSLGSGKVKGLDQRATLHRRTNQTYGLKRPTSRKILNEVQKKF
AN7299.1  	KGEGVPDVGEVWGVEVGLSLGSGKVKNLDLRPTLHRRTTTTYQLKRPSSRATLTEIVKKF
          	****:*::*******:*:******** :: *.******. ** ****:**  *.*: ***

MG06316.1 	GTFPFSLRQLEDERDAKSGVVECVRGNVFRQYELVGDKDGAAVARLLTTLA1ITKNGITK
NCU00422.1	GTFPFSLRQLEDERDAKSGVIECVRGNVFRAYEVVGDKDNSPVARLLTTVA1ITKNGLTK
FG06955.1 	GTFPFSLRQLEDERDAKSGVVECVRGNVFRQYELVGDKDNSPVARYLTTFA1ITKNGITK
AN7299.1  	GQFPFSLRQLDDEKAAKVGVIESVRNGVLRQYEPAGDADNAAVSRYLTTIA1ITKNGITK
          	* ********:**: ** **:*.**..*:* ** .** *.:.*:* ***.* *****:**

MG06316.1 	LGAAPPLDLSKYQTDKKIEDEEVLKILEQPLSRNTGNKKKKPKKKTKKPATKKEGEESEE
NCU00422.1	LGAAPALDLSKFKTDKKIEDEEILAILAQPLSRNTGSKNKNKKKKAAK----KEGEKADE
FG06955.1 	LGAPPPLDLEKYETDKRIEDEEILKILEQPIARNTGKKKSKPKKKTAK----KEGDEE--
AN7299.1  	LAAPATPDFEKIKSDKKIEDEEILKILELPLSKSTGSKNKNKKKKAKK----ADGADE--
          	*.*... *:.* ::**:*****:* **  *:::.**.*:.: ***: *     :* .   

MG06316.1 	ESDE
NCU00422.1	E---
FG06955.1 	----
AN7299.1  	----
          	
```
