# Supplementary material for: Patterns of Intron Gain and Loss in Fungi
Source: PLoS Biol. 2004 Nov 30;2(12):e422. doi: 10.1371/journal.pbio.0020422 (PMC532390; doi:10.1371/journal.pbio.0020422)
Supplement: Table S1 — Also available at http://genes.mit.edu/NielsenEtAl/. (4.3 MB ZIP). [file pbio.0020422.st001.zip › NielsenEtAl/html/1040.html]

AN3584.1.NCU03218.1.MG03638.1.FG09157.1


```
 CLUSTAL W (1.82) Multiple Sequence Alignments - Introns Inserted


Sequence 1: NCU03218.1	493 aa
Sequence 2: MG03638.1	495 aa
Sequence 3: FG09157.1	460 aa
Sequence 4: AN3584.1	487 aa
Alignment Length: 518 aa
Number Identitical Residues: 225 aa
Alignment Score (without introns) 10979


MG03638.1 	MGGIDQ--DNFSNISWQSDRLGAGSSAAHQHGGQESEQPTHNQAEPEPNYTSMIVHGMG-
NCU03218.1	MAVIDQ--DNFSNISWHSEQNAESAASTAQVHHESNSSPEYARSGPDDGRPGDNAAGMEH
FG09157.1 	MTATEQQQDDFSNVSWSEHVHDQQTRSVP-----DAEEPGHDMNAPGTGLERDAPS----
AN3584.1  	---MDH--DDFDSVSWRHGPDSDISRPTTS--GTDTAESPETRRDPNGKRRMSSASEIPQ
          	    ::  *:*..:**        : .. .    .  ..      *              

MG03638.1 	------------LGDEVLECNVSSPLKENDGTKDAFVSYLVTTH0TTFADFQKPDASVRR
NCU03218.1	D-------ELDHSGGEILDCTVSDPHKENDGTKDAYVSYLITTN0TTFPSFQKPKTTVRR
FG09157.1 	------------LGNEKLECTVDTPIKENDGTKDAFVSYLITTH0STFSSFQRSTTTVRR
AN3584.1  	AGPHADALDLAGIGDGVLECRVDTPIKENDGTKDAYISYLVTTH0TDFKSFQKADFTVRR
          	 .. :.: .    *.  *:* *. * *********::***:**: : * .**:.  :***

MG03638.1 	RFTDFVFLFKTLSREYPASAVPPLPDKQRMEYVRGDRFGNDFTSRRAYSLRRFLARCALH
NCU03218.1	RFTDFVFLYKVLCRDYQACAVPPLPDKQRMEYVRGDRFGTDFTARRAYSLQRFLARLALH
FG09157.1 	RFTDFVFLYKQLTRDYPAAAVPPLPDKQRMEYVRGDRFGSDFTTRRANSLQRFLSRLSLH
AN3584.1  	RFTDFVFLYKTLYREYPACAVPPLPDKHKMEYVRGDRFGAEFTTRRAWSLHRFLKRLTLH
          	********:* * *:* *.********::********** :**:*** **:*** * :**

MG03638.1 	PVLRRSAILHTFLESPDWNATMRSRASRSVSMSGTSSGESGNAHYGGGSAGGGSTGGGNS
NCU03218.1	PILRKADILHAFLESPDWNATMRS---RSVRGSLASPGGIGDSTLGGSAAAGG--GGG--
FG09157.1 	PTLRRAPILHTFLESPDWNATMRS---RGSRVSSASDPGSAG------------------
AN3584.1  	PVLRRAPLLAIFLESPDWNAHMRL---RGSRASTSGSDGGGTG-----------------
          	* **:: :*  ********* **    *.   * :.    . .                 

MG03638.1 	LANSVFDNFADTFINAFTKVHKPDRRFIEVREKSDKLDEDLAHVEKVVARVSRRETDMEA
NCU03218.1	----VFDTFADSFMNAFTKVHKPDRRFIEIKEKSDKLDEDLNHIEKVVARVARREADIES
FG09157.1 	----VFDNFADTFINAFTKLHRPDRRFLEVKEKSDKLDDDLGHIEKVIARVARREADLEV
AN3584.1  	----IFDNFTDTFVNAFTKVHKPDRRFIEVREKADKLDEDLTHVEKIVARVARREADLET
          	    :**.*:*:*:*****:*:*****:*::**:****:** *:**::***:***:*:* 

MG03638.1 	DHKDLAEQFQKLIVLEPGVEGPVRAFAASVEDTAQGLRGLREATEQDYLGSLRDLAAYSG
NCU03218.1	DLKDLAEQFQKLITLEPGVETAVRAFAASVEDTASGLKKLKDHTDQDYLGSLRDMVAYSG
FG09157.1 	DLRDLAEQFQKLIPLEPHVEPAVHGFSASIEDTASHLRKLKDMTDQDYLGSLRDMQAYSI
AN3584.1  	DYNDLATQFRKLVPLEPEVEVPLQVFAASVEETARGIKNLKDHTDQNYLGSLRDMEAYIL
          	* .*** **:**: *** ** .:: *:**:*:**  :: *:: *:*:*******: **  

MG03638.1 	ALKNLLKAREQKQLDFEQLTEYLNKSSAERDVLASG--GYSSGGALAG-AGGFIRSKIED
NCU03218.1	TLKNLLKAREQKQLDYEQLTEYLNKSRTDRDMLASGQ-SYGAGSALMSGAGGFIRSKIED
FG09157.1 	ALKNLLKAREQKQLDYEQLTEYLNKSTTERDTLQSGH-GGGSG------AGSFLRAKIED
AN3584.1  	SVKSLLKTREQKQLDFEALVDYRNKAVAERDSLAANPSSYYASNPLTSSPASFIRSKMED
          	::*.***:*******:* *.:* **: ::** * :. :.  :...  .....*:*:*:**

MG03638.1 	VRGVDHEQSRRERLRKLELRIEELTVEVERAKKTSELFDEEVIREVSDFERIKRIELKRQ
NCU03218.1	VRGVDHEQARRDRQRKLELRIEELTREVEVARNESESFAEQVSREVESFDWIKRVEFKRQ
FG09157.1 	VRGVDHEQARRERTRKLELRVEELTHEVESARKTSDMFDDEVVKEVADFERIKRIEMKAQ
AN3584.1  	MRGVDHEQSRRERMRKLELRIDELTREVESAKTTSEMFDEEVVREVADFERIKAIEFRDS
          	:*******:**:* ******::*** *** *:. *: * ::* :** .*: ** :*:: .

MG03638.1 	FGSLAQSHTDFYDATIDVWEKYVKEMEKEGAVAA-----
NCU03218.1	FSGLADAHIEFYGDVMSVWEQYVMEMEKEGVVLPA----
FG09157.1 	LGSLADSHIEFYGEVASIWEKYVEEMEKQGITSA-----
AN3584.1  	LGALAEQHIEFYQGVLNTWERFVAEMEEEQSTGDAHPNA
          	:..**: * :**  . . **::* ***::  .  : ..:
```
