# Supplementary material for: Patterns of Intron Gain and Loss in Fungi
Source: PLoS Biol. 2004 Nov 30;2(12):e422. doi: 10.1371/journal.pbio.0020422 (PMC532390; doi:10.1371/journal.pbio.0020422)
Supplement: Table S1 — Also available at http://genes.mit.edu/NielsenEtAl/. (4.3 MB ZIP). [file pbio.0020422.st001.zip › NielsenEtAl/html/1041.html]

AN1103.1.NCU00446.1.MG05196.1.FG04103.1


```
 CLUSTAL W (1.82) Multiple Sequence Alignments - Introns Inserted


Sequence 1: NCU00446.1	689 aa
Sequence 2: FG04103.1	696 aa
Sequence 3: MG05196.1	718 aa
Sequence 4: AN1103.1	690 aa
Alignment Length: 748 aa
Number Identitical Residues: 287 aa
Alignment Score (without introns) 15431


MG05196.1 	MEKTKRDTLDSTGDAVPAPQDMASHQAKRVKMGNGAPGAVELWRHPDPKSTPMWKFLEHV
NCU00446.1	-------------------MTVTN----------------ELWRHPDPQSTQMWKFLEHV
FG04103.1 	------MSIDKLAPSAAAPPSEDS----------------VLWQHPDPKSTHMWKFLEHV
AN1103.1  	-------------MSANGSPASPQ----------------ELWRHSAPESTRIYHFTKTI
          	              :  ..    .                 **:*. *:** :::* : :

MG05196.1 	NSTRSLSLASYEQLHKWSTADPAAFWGDVWHYAGVVASKPYDE~VLPEGG-MFPRLNFFS
NCU00446.1	NSKYGLQLNDYPSLYKWSVDNVAAFWEEAWHFTGIRHSKLFDE0VLPEHAPMLPRPDFFA
FG04103.1 	NKKHGLSLDGYPALYKWSIDDVSSFWEEVWHFTGVTASKSFDE0VLPQNAPMYPRPDFFS
AN1103.1  	AEKHGIPLRSYNDLWNWSISEPAKFWEEIWLYTSIIAHRPYDQ0VMGSETLLFPRPQFFE
          	 .. .: * .*  * :**  : : ** : * ::.:   : :*: *: .   : ** :** 

MG05196.1 	GALLNFAENLLFPPG-VDVDPSAVAVITATETDE--KETTWAELRDMVSRCAVALRAAGL
NCU00446.1	GARLNFAENLLFPGN-AQVNGSDVAVITATENDEHLTETTWDELREQVRQCSNALQAAGV
FG04103.1 	GSLLNFAENLLFPATSPQPEPSAPAVITVTELPNSTVTTSWAELRDAVRRCSNALRAAGL
AN1103.1  	GASLNFAENLLYPAC--SPDENAVAIIAATEADR--EYISWKELRDRVRRCANALKGAGL
          	*: ********:*    . : .  *:*:.**  .     :* ***: * :*: **:.**:

MG05196.1 	QRGDVVAGYISNHVEALVAALGAASIGALWTGISPDNGVSAVLDRLVQVRPKVLFADNGT
NCU00446.1	KENSVVAGFVANHVQALVALLSAASLGAIWTGISPDNGVSAVLDRLVQIKPKVLFSDNAT
FG04103.1 	KPNDVVAGFVSNHVEAIVAMLGAAAIGAIWTGISPDNGVSAVLDRLNQIAPKVLFADNGT
AN1103.1  	QKGDRVAGFLGNHANTVVAMLATTSIGAFWTGVSPDTGVHAVLERLTQIEPKILFADNAS
          	: .. ***::.**.:::** *.::::**:***:***.** ***:** *: **:**:**.:

MG05196.1 	TYNGKSWASTPKTLEIVGELKKNGLELVVVTRNLQAIDLSLDEIRALDVKADDFDAFLAA
NCU00446.1	LYNGKEWSGKAKTLEVVEALQKHGLELVVVVKGLQNFETGLDEIRVKGVKAEEYDEFLHS
FG04103.1 	VYNGKEWSSVSKTTEIVGALKDNGLERVVVINNISSGGLGLEELEKHGVVAVDYTKMLES
AN1103.1  	LYNGKVHSAAAKTSQIVCELPK--LEALVIFPTIGSVEIKLEEVSLRQGKAYTYADFLLT
          	 ****  :. .** ::*  * .  ** :*:   :      *:*:      *  :  :* :

MG05196.1 	AQVEQNVTGSQQQFEQLPSDHPLFILYSSGTTGVPK0AIVHSALGTLIQHKKEHLLHGSL
NCU00446.1	SPKEEPLV-----FAQLPPSHPLYVLYSSGTTGLPK~AIVHTAAGTLLQHKKELFIHSNL
FG04103.1 	AS-EEPLK-----FEQLPPSHPLYVLYSSGTTGLPK~AIVHTALGTLLQHKKEHLLHCSL
AN1103.1  	ASNPAAPL----EFASLPPEHPVYILYSSGTTGAPK~PIVHGSLGTLLQHKKEHVLHCDI
          	:           .* .**..**:::******** ** .*** : ***:***** .:* .:

MG05196.1 	SSKSRMLYFT1TTSWMMWHWSITALSVGTSLVLYSGSPFRPD------GYLSLPKLLSKL
NCU00446.1	SPRSRMLYYT~TTSWMMHHWSVSSLSCGASLVLYSGSPFRPH------GYLSLPRLLSSL
FG04103.1 	DSTSRMLYYT~TTSWMMWHWSIGALAVGSTVVVYSGSPFRPH------AHLSLPRLLSDL
AN1103.1  	YPGDRLFYFT~TTTWMMWHWLVSGLASGATIVLYDGSPFRPLDPEGGAGEMAMPRLIDEL
          	 . .*::*:* **:*** ** : .*: *:::*:*.****** .....:. :::*:*:..*

MG05196.1 	KVTHFGTSAAYLTALEANKIYPVGEPG--IDLSSLEGIYSTAAPLPPSTFAFVYEAFPSK
NCU00446.1	KVTHFGTSAAYLTTLEANSVRPIDPVHN-LDLSSQEAIYSTKKTLPPSTFAFVYTAFPST
FG04103.1 	EVTHFGTSAAYLTALEANNVYPVRDSS--IDLSRLQAIYSTASPLPPSTFKFVYEAFPKH
AN1103.1  	QITHFGTSAKYLSMLEQAALNPREYPHRPVSLQTLKAIFSTGSPLAPSTFEYVYSSIHPD
          	::******* **: **   : *      .:.*.  :.*:**  .*.**** :** ::   

MG05196.1 	IHLASITGGTDIISLFGAPCPLLPVRAGEVQCAGLGMAVAVVDSASDPDKPVLIT-DGEP
NCU00446.1	INLASITGGTDIISLFGAPCPLLPVRAGEVQCAGLGMAISVIDSASDASDPRPVDPVGSE
FG04103.1 	INLGSITGGTDIISLFGAPCPLLPVRVGEIQCAGLGMAIRAVDSATGES----IN-ADEP
AN1103.1  	IMLGSITGGTDILSLFCSGCPILPVYKGEIQCRSLAMAVSVYDYAGNDISS-----SGEP
          	* *.********:*** : **:***  **:** .*.**: . * * .  ..      .. 

MG05196.1 	GDLVCTVPFPCQPLTFFGDG--GDEKYRKAYFERFP-------GMWHHGDFVRQVDGPSG
NCU00446.1	GDLVCLKPFPCQPLTFFGPG--GDDKYRAAYFERFG------TAMWHHGDFVRMN-PSTG
FG04103.1 	GDLVCVKPFLCQPLTFFGPS--GEAKYKSAYFERFEDICGVDGAVWHHGDFIKIPDPSTG
AN1103.1  	GDLVCTRPFPAQPVMFWPPGPIGVEKYRKSYFDVFG------PSIWHHGDFVRLN-PETG
          	*****  ** .**: *:  .. *  **: :**: *        .:******::     :*

MG05196.1 	GLVMLGRSDGVLKPAGVRFGSAEIYNILTKFFPGDVEDALCVGRRRE-GDTDEAVCLFVV
NCU00446.1	ALVMLGRSDGVLKPAGVRFGSAEIYNVLIRFFASEVEDAVCVGRRRE-FDRDETVCLFVV
FG04103.1 	SLVMLGRSDGVLKPSGVRFGSAEIYNILTRFFAAEIEDAVCIGRRRE-TDSDETVCLFVV
AN1103.1  	GVVMLGRSDGVLKPAGVRFGSAEIYNILLKHFADEIEDSLCVGRRREGIDTDETVVLFVK
          	.:************:***********:* :.*. ::**::*:*****. * **:* *** 

MG05196.1 	PAA-GKTFDDELKGRIKTTIRSELSPRHVPAFIEEAQ-GGIPKTGNGKK2IEVAVKQIIS
NCU00446.1	MVP-GKSFTSELRDRIKNVIKRELSPRHVPGVVEECGPAGIPRTGNGKK~IEVAVKQILS
FG04103.1 	MVP-GHEFNDDLRLRIKSKIKAELSPRHVPGVVEECG-AGVPKTSNGKK2IEVAVKQILS
AN1103.1  	LASPSDSLPPDLASRIQATIRRELSPRHVPGIVDVCP--EIPVTSNGKK~VENAVKQILC
          	 ..... :  :*  **:  *: ********..:: .    :* *.**** :* *****:.

MG05196.1 	GMQVKTNASVANPESLDWFRAWHEQHP-----
NCU00446.1	GLKVKTNASVANPEALEWFRTWAKAAEEQLPK
FG04103.1 	GMKVKTNASVANPEALDWFKSWAETR------
AN1103.1  	GLNIKIGASVANASCLDWYRTWAAQHP-----
          	*:::* .*****...*:*:::*
```
