# Supplementary material for: Patterns of Intron Gain and Loss in Fungi
Source: PLoS Biol. 2004 Nov 30;2(12):e422. doi: 10.1371/journal.pbio.0020422 (PMC532390; doi:10.1371/journal.pbio.0020422)
Supplement: Table S1 — Also available at http://genes.mit.edu/NielsenEtAl/. (4.3 MB ZIP). [file pbio.0020422.st001.zip › NielsenEtAl/html/1042.html]

AN3011.1.NCU09299.1.MG03486.1.FG07851.1


```
 CLUSTAL W (1.82) Multiple Sequence Alignments - Introns Inserted


Sequence 1: MG03486.1	119 aa
Sequence 2: FG07851.1	119 aa
Sequence 3: AN3011.1	119 aa
Sequence 4: NCU09299.1	117 aa
Alignment Length: 121 aa
Number Identitical Residues: 58 aa
Alignment Score (without introns) 3055


MG03486.1 	MPQDMPPVGGYGPVQYK0RNLPSRGFRPAQLLFGMGLVMTYGWYKLGKGIR~EQN2ELAR
NCU09299.1	MPQDMPPAGGYDAVQYK0RNLPPSAFKPKTLLAFGGLIMVYGWYHLFHGIQ1---~-LAR
FG07851.1 	MAQDMPPKGGYEPVQYK0RNLPARGLRPGILLLGMGAVMGYGWYKLIGGMR~EMN2ELGR
AN3011.1  	MPQDMPPAGGYRQVQYK0RNIPARGFRPITYLVGMHLFMAYGYYKLFYGIR~EQN2ELAR
          	*.***** ***  **** **:*. .::*   *     .* **:*:*  *:: . . .*.*

MG03486.1 	EKMWARIHLIPALQAEEDRDAVRRHLADQAREKELMGSN-FPVYNSDR2YVRPTYAVTPT
NCU09299.1	EKMWSRIHLIPALQAEEDRDLVRRHLADVQREKELLGDKAVKAYHSDR~YVRPTFAITPG
FG07851.1 	EKMWARINLIPVLQAEEDRDQVRRYLADQKREKELLGDN-AKVYNSNR2FVRPTFAVTPP
AN3011.1  	EKIWGRLHILPLLQAEEDRDQVRRYYADKAREQQLLGSE-TKVYNSDR2FVRPTFAYTPA
          	**:*.*::::* ******** ***: **  **::*:*.:   .*:*:* :****:* ** 

MG03486.1 	QTKE-
NCU09299.1	KIIKD
FG07851.1 	PTTN-
AN3011.1  	KAIN-
          	   :
```
