# Supplementary material for: Patterns of Intron Gain and Loss in Fungi
Source: PLoS Biol. 2004 Nov 30;2(12):e422. doi: 10.1371/journal.pbio.0020422 (PMC532390; doi:10.1371/journal.pbio.0020422)
Supplement: Table S1 — Also available at http://genes.mit.edu/NielsenEtAl/. (4.3 MB ZIP). [file pbio.0020422.st001.zip › NielsenEtAl/html/1043.html]

AN0956.1.NCU00065.1.MG08825.1.FG07217.1


```
 CLUSTAL W (1.82) Multiple Sequence Alignments - Introns Inserted


Sequence 1: MG08825.1	614 aa
Sequence 2: FG07217.1	510 aa
Sequence 3: NCU00065.1	629 aa
Sequence 4: AN0956.1	563 aa
Alignment Length: 661 aa
Number Identitical Residues: 224 aa
Alignment Score (without introns) 11230


MG08825.1 	-----------MTTTAAAT-SMPGSDTNGVTSGVKPKLHGRAFYESIGSPKFVVAPMVDQ
NCU00065.1	MASHSATTASSLTSAAANLGATEANATTTPRAPKRTKLHGRAFYESIGSPKYIVAPMVDQ
FG07217.1 	-----------MTSKENSP-AVERP----------TKLEGRAFYESIGSPKFIVAPMVDQ
AN0956.1  	--------MAISETVPAISNGVAQIAPEKAEPVTRKKLHGRAFYESIGSPKYIVAPMVDR
          	         :   :     ..     .    .    **.************::******:

MG08825.1 	SEF0AWRMLTRSFMPESQRSTVLGYTPMFHARLFGENPSYRDSHFQAIR----KDAASPD
NCU00065.1	SEF0AWRMLTRSFLPADEQHKLLCYTPMFHARLFTEAPKYRDSHFQPIRGPLTADSPRPD
FG07217.1 	SEF0AWRMLTRSFISPTEQKSLLAYTPMLHARLFSQDDKYRKAHFQAVK----TDG----
AN0956.1  	SEF0AWRMLTRSFMTPEESKSVLAYSPMYHARLFEEHAGYRAKAFHPTR----AWGDTKC
          	*** *********:.  :  .:* *:** ***** :   **   *:. :      .    

MG08825.1 	A-QPTPWLDGNPAIDRPLFVQFCANDPSALLEAAKHVAPYCDAVDLNLGCPQGIARKGHY
NCU00065.1	SSDYVPFLDGNPEFDRSLFVQFCANDPAYLLSAAKLVAPYCDAVDLNLGCPQGIAKRGQY
FG07217.1 	---ETPWLDGNPSIDRPLFVQFCANDPDALLSAAKQVAPYCDAVDLNLGCPQGIARKGKY
AN0956.1  	KGDDSPYLDGNPAIDRPLFVQFCANDPDDFFNAARHVAPYCDAVDLNLGCPQGIARRGHY
          	 ..  *:***** :**.**********  ::.**: *******************::*:*

MG08825.1 	GAFLQEDQELIHKLIRQLHDGLSIPVTAKIRILDTPEATLAYARNVLDAGASILTVHGRR
NCU00065.1	GSFLQENQELIFELINTLHKELDIPVTAKIRILDTKEATLKYAQNVLRAGASILTVHGRR
FG07217.1 	GAFLQEDQDLIFRLINILHKELPVPVTAKIRILDTKEETLAYAQNVLKAGASILTVHGRK
AN0956.1  	GAFLQEDWDLIYKLINKLHMELEVPVTAKFRIQESKEKTLEYAKMILSAGASIITLHGRT
          	*:****: :**..**. **  * :*****:** :: * ** **: :* *****:*:*** 

MG08825.1 	REQKGHLTGVADWSAIRHLRENLPPETVIFANGNILQRGDLERCLEATGADAVMSAEGNL
NCU00065.1	REQKGHQTGLADWEYIRYLRENLPKETVIFANGNILQHADLEKCLAATGADGVMSAEGNL
FG07217.1 	REQKGHLTGLAEWQMIRFLRDSLPKETVIFANGNILQEGDIEKCLEATGADGVMSAEGNL
AN0956.1  	REQKGHNTGLADWSYIRYLRDNLPPDTVIFANGNNLNHDDLARCLEATGADGVMSAEGNL
          	****** **:*:*. **.**:.** :******** *:. *: :** *****.********

MG08825.1 	SDPTLFAPEPPVGEEGREYWRGKDG-LGGFRVDAVMRRYLDILHRYVEDKEPPVRRPLFV
NCU00065.1	SDPGLFARPPAVGEEGREYWRSKDGSRGGWRVDAVLRRYLDIIYKYVLEQQPPVRRPLFM
FG07217.1 	SDPAIFTKPPPVGEEGREYWRGKDG-KGGYRVDAVFRRYMDILHEHVFGNKPPARRPLFM
AN0956.1  	SDPSIFAKPPPVGSEGREYWRGRDG-RGGYRIDAVFRRYLDIIYKYVLEQPVPERKPLYI
          	*** :*:  *.**.*******.:**  **:*:***:***:**::.:*  :  * *:**::

MG08825.1 	VGEDEAWITQSLETQAATQAPEGGETEAPADAEEDEEGQPAHKKRRKTH-----KKKMRA
NCU00065.1	PGDDVAWLEEASSTSFETTQANDDSEGPARKKHKSANGSASTTTTTTAASSSSLNTTSNS
FG07217.1 	PGDDTEWMKE-------------------SEAVEEE---PPSKKRRK-------------
AN0956.1  	PSDPEEEALPQSNTTTNTTAPAAEVEAHGGEEKEEEEEGPPKKKQRKQK-----------
          	 .:         .:   :  .         .  :. .  .. ..  .             

MG08825.1 	AETGIGKKDKNPSPNYIAVKAHLFHMLRHFITKHTDVRDAVARSRLGEGHINYLERILDM
NCU00065.1	NNPHLDKKALQTSPNLVSMQPHCFHLLRHFVTHHTDVRDLLARARNGS-EIGKYEAILSQ
FG07217.1 	---DLGKKGEQ-GPNMAAMQPHLFHLLRHFVSKHTDVRDMLGKSRAGD--IEAYERVLSA
AN0956.1  	-VPHKFRHGNPNSPSTKFMQAHLFQLFRPLIATHTNVRDALARSSPSD--MSTFEHTLSI
          	  .   ::    .*.   ::.* *:::* ::: **:*** :.::  ..  :   *  *. 

MG08825.1 	VERKVAQGMIEYARTEGKSFEDEPLARITIKPQPKAETKDSGVPIAAAEAANGESEKSAV
NCU00065.1	VERKVALGLLEYERTGGSSFDVDVLETLY-----------------------QETE----
FG07217.1 	VERKVAEELLEYERTNGESIAETPLA---------------------------EGEE---
AN0956.1  	LEEEVRKGLKEYKQFP-ERFEH--------------------------------------
          	:*.:*   : ** :   . :                                        

MG08825.1 	DAVLDPEDDPESSFATVKKCRRPWWVVQPIVRPLPLEAAAKGAVTMKKP----KENKSNN
NCU00065.1	----DSKDDPESSAQARRENKRPWWVVQPIIRPLPKEALAKGALQLSKKELKAQAAKKAA
FG07217.1 	----DPPE-TESSKGTQRRCKRPVWVVQPIIRPLPNEALKKGALTMSKK----DKAKSQE
AN0956.1  	----KPDPSLTGSKATIAEYGRPFWVCQPHIRPTPEEAMEVGALQVKKG----EKQKKAE
          	    ..  .  .*  :  .  ** ** ** :** * **   **: :.*     .  *.  

MG08825.1 	GSNGG----------NGKQEDASRKRP----------HPDTESKVEALDKEISFPTSDLV
NCU00065.1	GGGGGGDAEATVASNLGSKEKEEKKTASEEALVPVQGQNGGETAVESLETTTNYPKSELV
FG07217.1 	---------------KKEEEKQEEKGK----------QEDIKAKDEALAG----------
AN0956.1  	VE--------------NEQDKANDRME----------AVSSVHTVEAADTPTTAAKDALL
          	                 .::. . :              .     *:     . ...   

MG08825.1 	SG
NCU00065.1	SG
FG07217.1 	--
AN0956.1  	SG
          	:.
```
