# Supplementary material for: Patterns of Intron Gain and Loss in Fungi
Source: PLoS Biol. 2004 Nov 30;2(12):e422. doi: 10.1371/journal.pbio.0020422 (PMC532390; doi:10.1371/journal.pbio.0020422)
Supplement: Table S1 — Also available at http://genes.mit.edu/NielsenEtAl/. (4.3 MB ZIP). [file pbio.0020422.st001.zip › NielsenEtAl/html/1044.html]

AN4577.1.NCU02361.1.MG03533.1.FG09042.1


```
 CLUSTAL W (1.82) Multiple Sequence Alignments - Introns Inserted


Sequence 1: AN4577.1	411 aa
Sequence 2: MG03533.1	457 aa
Sequence 3: NCU02361.1	502 aa
Sequence 4: FG09042.1	403 aa
Alignment Length: 504 aa
Number Identitical Residues: 244 aa
Alignment Score (without introns) 12222


MG03533.1 	-------------~----------------------------MGWQEASVSLAKIACI1S
NCU02361.1	MHLPYLIANCRPY2VSKRNYRNCGDIIWKPSTAPISPTRCGGLARKSSMTFGAIGDDT~W
FG09042.1 	-------------~--------------------------------------------~-
AN4577.1  	-------------~----------------------------MG--------------~-
          	                                           .                

MG03533.1 	~IQLYIMPEIRKVCKVSLDKPAAEQSALH0NRWHPDI~PFAGTIKNG~ETVKIECVDW2T
NCU02361.1	0TDNMGKTEIRNVCKVDFDKPASEQPQLH0NRWHPDI~PFAGFIKDG~ETVKIECVDW~T
FG09042.1 	~----MSRKIRTAQSVSFDIPAAEQPHLH~NRWHP--~-------EG1ETVKIECLDW~T
AN4577.1  	~-----TKAIRTAVKVDLNKPAREQKGLH0NRWHPDI1PSYGTIANN~EVVKIECLDW~T
          	         **.. .*.:: ** **  ** *****.  .  .   :. *.*****:** *

MG03533.1 	GGQIGNNDSADDIRNVDLTKIHYLSGPFEVETAEPGDLLLVEIMDVQPFEDQPWGFTGVF
NCU02361.1	GGQIGNNDSADDMKNVDLTKIHYLSGPFEVEGAQPGDLLLVEIMDVQPFQDRPWGFTGIF
FG09042.1 	GGQIGNNDSADDVRDVDLTKIHYLTGPFEIKGSEPGDLLVVNITDIQPFDHSPWGFTGVF
AN4577.1  	GGQIKNDDSADDIKNVDLTQIHYLSGPFDIETAEPGDVLLVEIQDVQPFEDQPWGFTGVF
          	**** *:*****:::****:****:***::: ::***:*:*:* *:***:. ******:*

MG03533.1 	DKSNG~GGFLDEIYPSA2AKAIWDFEGIYCTSRHIPHVKFAGLIHPGILGCAPSAEVLAE
NCU02361.1	DKANG0GGFLDEIYPSA~AKAIWDFEGIYATSRHIPGVKFAGLIHPGIIGCAPSAEVLAT
FG09042.1 	SKDNG~GGFLSEHYPDA~AKAIWDFDGVYCSSRHIPGVRFPGLIHPGILGCAPSAEILAE
AN4577.1  	ARENG~GGFLDEIYPEP2AKAIWDFEGIFCSSRHIPHVRFAGLIHPGILGCAPSAEVLAE
          	 : ** ****.* **.. *******:*::.:***** *:*.*******:*******:** 

MG03533.1 	WNKREGDLIA--ANKLE-RDVAKPPEPVNVHAGAADASLTARVGKEGARTIP~GRPEHGG
NCU02361.1	WNKREGELVA--ANKLADRDVAHLPQPINTHAGAASKEIAEKVAKEGARTVP~GRPEHGG
FG09042.1 	WNRREGELATSMAGSHPDKVFAQPPNEINAHGGLAKGDVMARIAKEGARTIP~GRPEHGG
AN4577.1  	WNRREGELIA--ANTLG-RDVAKPPEPSNVHAGSADGELAAKIGREGARTIP0GRPEHGG
          	**:***:* :  *..   : .*: *:  *.*.* *. .:  ::.:*****:* *******

MG03533.1 	NCD2IKNLSRGSKVYLPVHVSGAKFSVGDLHFSQ1GDGEISFCGAIEM~AGVITINFKVI
NCU02361.1	NCD~IKNLSRGSKVYLPVHVPGAKFSVGDLHFSQ~GDGEISFCGAIEM~AGVITINFKVI
FG09042.1 	NCD~INNISRGSKTYLPVHVPGAKFSVGDLHFSQ~GDGEISFCGAIEM0AGIITIKFDLI
AN4577.1  	NCD~IKNLSRGSKVYLPVHVPGAKFSVGDLHFSQ~GDGEISFCGAIEM~AGVITLKFTVI
          	*** *:*:*****.******.************* ************* **:**::* :*

MG03533.1 	KNGMADLGLKSPIYIPGPVEPHFGPGRHIYFEGFSVDQHGKQH~YMDVTVAYRQTCLR1V
NCU02361.1	KNGVEGMGLKSPIYIPGPVEPSFGPGRYIYFEGFSVDEHGKQH1YMDVTVAYRQTVLR~C
FG09042.1 	KGGVKSRGLKSPVYRPGDMGPTFSPSRYLTFEGFSVDEQGKQH~FMDATIAYRQSCLR~A
AN4577.1  	KDGMAKMAMKSPIFHPGPVEPQFGPGRYLTFEGFSVDEKGKQH~YLDATVAYRQTCLR~V
          	*.*:   .:***:: ** : * *.*.*:: *******::**** ::*.*:****: **  

MG03533.1 	IEYLRRFG~YSDYQIYLLMSCAPIQGHIAGIVDIPNACTTLGLPMDIFDFDISPS---AV
NCU02361.1	IEYLRRFG~YSDYQIYLLLSCAPIQGHVAGIVDVPNACTTLGLPMDIFDFDISPAAALKA
FG09042.1 	IEYLKQFG1YSGEQIYLLLSCAPIRGAIAGIVDIPNACTTLGIPMDIFDFDISIE----S
AN4577.1  	IEYLRRYG~YNDYQIYLLLSCAPVQGHIAGLVDIPNACTTLGVPMDIFDFDIRP----EA
          	****:::* *.. *****:****::* :**:**:********:*********        

MG03533.1 	PAKKLDMGTCAFETGVTEGKVTKGGENSEHSFGGGMTYKS
NCU02361.1	GETKRDLGTCAFETGVTEGKVTNGGKNSQISFGGGLTYKE
FG09042.1 	EPVVRNLGACPISR--------------------------
AN4577.1  	DAVKLDMGSCAFASK-------------------------
          	     ::*:*.:
```
