# Supplementary material for: Patterns of Intron Gain and Loss in Fungi
Source: PLoS Biol. 2004 Nov 30;2(12):e422. doi: 10.1371/journal.pbio.0020422 (PMC532390; doi:10.1371/journal.pbio.0020422)
Supplement: Table S1 — Also available at http://genes.mit.edu/NielsenEtAl/. (4.3 MB ZIP). [file pbio.0020422.st001.zip › NielsenEtAl/html/1045.html]

AN8216.1.NCU04202.1.MG08622.1.FG05972.1


```
 CLUSTAL W (1.82) Multiple Sequence Alignments - Introns Inserted


Sequence 1: NCU04202.1	152 aa
Sequence 2: FG05972.1	238 aa
Sequence 3: AN8216.1	161 aa
Sequence 4: MG08622.1	241 aa
Alignment Length: 242 aa
Number Identitical Residues: 98 aa
Alignment Score (without introns) 4494


MG08622.1 	MASPDSQPKPKPSSNGATTGDPAIKLGHQQRQKIFTPPNFALLLCLLLSLVNFYHLQKPG
NCU04202.1	------------------------------------------------------------
FG05972.1 	MVAEEPRQRKDPAVSGKENSKQQRRRTAENLAPPFFALLFALLAFYILFSPPSSSLSPPV
AN8216.1  	------------------------------------------------------------
          	                                                            

MG08622.1 	RASVIYVQEPSTPIPPTIYKNTAKMST-EQT2FIAIKPDGVQ0RGLVGPIISRFEQRG2Y
NCU04202.1	------------------------MSNQEQT2FIAVKPDGVQ~RGLVGNIISRFENRG~F
FG05972.1 	PVCHSTISSSVSSSQVIPDKNIAKMSSSEQT2FIAIKPDGVQ0RGLVGPIISRFENRG2F
AN8216.1  	----------------MPLLEPTKLTS-HRS~FIAIKPDGVQ0RGLVGPIISRFENRG2F
          	                    . :.::. .:: ***:****** ***** ******:** :

MG08622.1 	KLVAIKLVTPGKAHLEQHY1ADLKDKPFFAGLVEY1QPADMNSGPIAAMVWEGRDAVKTG
NCU04202.1	KLVAMKLTQPGQAHLEKHY~EDLNTKPFFAGLIKY~----MNSGPICAMVWEGKDAVKTG
FG05972.1 	KLAAIKLMTPGKEHLEKHY1ADLAGKPFFAGLIEY~----MNSGPICAMVWEGRDAVKTG
AN8216.1  	KLAAMKLTSPSRSLLEQHY~SDLKEKPFFPGLVTY1----MLSGPIVAMVWEGKDVVKTG
          	**.*:**  *.:  **:**  **  ****.**: *     * **** ******:*.****

MG08622.1 	RT1LLGATNPLASAPGTIRG2DYAI~DVGRNVCHGSDSVENAKKEIALWFKEGEVVSYQA
NCU04202.1	RT~ILGATNPLASAPGTIRG~DFAL0DMGRNVCHGSDSVENAKKEIALWFKPEELNQWNH
FG05972.1 	RS1ILGATNPLASSPGTIRG~DYAI~DVGRNVCHGSDSVENAQKEIALWFKEGEVVSWKS
AN8216.1  	RT~ILGATNPLASAPGTIRG~DFAI~DVGRNVCHGSDSVESAKKEIGLWFTPEEIQNYKL
          	*: :*********:****** *:*: *:************.*:***.***.  *: .:: 

MG08622.1 	SQASWIYEKP
NCU04202.1	HSAAWIFE--
FG05972.1 	AQFNWVYEKA
AN8216.1  	NAFGWIYEKE
          	    *::*.
```
