# Supplementary material for: Patterns of Intron Gain and Loss in Fungi
Source: PLoS Biol. 2004 Nov 30;2(12):e422. doi: 10.1371/journal.pbio.0020422 (PMC532390; doi:10.1371/journal.pbio.0020422)
Supplement: Table S1 — Also available at http://genes.mit.edu/NielsenEtAl/. (4.3 MB ZIP). [file pbio.0020422.st001.zip › NielsenEtAl/html/1046.html]

AN3370.1.NCU06057.1.MG09243.1.FG09385.1


```
 CLUSTAL W (1.82) Multiple Sequence Alignments - Introns Inserted


Sequence 1: MG09243.1	439 aa
Sequence 2: FG09385.1	422 aa
Sequence 3: NCU06057.1	487 aa
Sequence 4: AN3370.1	443 aa
Alignment Length: 505 aa
Number Identitical Residues: 205 aa
Alignment Score (without introns) 9978


MG09243.1 	MSSPKPPGT-SRSGPARTIDAFFNSPSAVYTAATLLRLGMLLYGLWQDAVSPVKYTDIDY
NCU06057.1	MATTSPAAPRPKSKSSSLLSTLFSRPLPLYVSAFLLRIVLLLYGLWQDANSPLKYTDIDY
FG09385.1 	MPSIAP----------------FLRTTPLFTISLLLRLGLLFYGIYQDAHSALKYTDIDY
AN3370.1  	MES------------------LFKRPFMVYGLAAGLRTVLLFYGAWQDAHSAVKYTDIDY
          	* :                   *  .  ::  :  **  :*:** :*** *.:*******

MG09243.1 	LVFTDAARFVADGGSPYDRETYRYTPLLAWMLVPT-----------------TTWFEFGK
NCU06057.1	LVFTDAARFVSRGESPYARETYRYTPILAWLLLPTTWTAGAQWGPWAAKVINVAWFSFGK
FG09385.1 	LVFTDASRFVADGQSPYARDTYRYTPLLAWILLPT-----------------VRFPAFGK
AN3370.1  	MVFTDASRYVSQGDSPYARDTYRYTPLLAWMLLPTT-------------WAIPGFFSFGK
          	:*****:*:*: * *** *:******:***:*:**:                  :  ***

MG09243.1 	VLFAAADLAAGHLLVSVLV------------------------RRGG-------MDGGTA
NCU06057.1	VLFAAADLVAGWLIEQVLVMGKDFPSSAAKGKEKDTEKTKEGGKKGPSVTASTGMDPSRA
FG09385.1 	LVFAAADLLAGWLILRVLR------------------------RRG--------MDEATA
AN3370.1  	ALFALSDVVAGWLVAKSLT-----------------------LTHG--------MSAERA
          	 :** :*: ** *:   *                          :*        *.   *

MG09243.1 	RRYAAVWLLNPMVAAISTRGSSEGLLGVLVAALLWAVLERRVVLAGVVLGLGVHFKIYPF
NCU06057.1	RAFAAIWLLNPMVATISTRGSSEGLLGVLVMALLWAVLSRRITLAGLLLGFSVHFKIYPF
FG09385.1 	GGFSALWLWNPMVATISTRGSSEGLLGVLTMGLLWAVDRRKFSLAAIILGLSVHFKIYPF
AN3370.1  	LKYASFWLLNPMVANISTRGSSEGLLGVLVVALLWAVLNRRIYLGGVLLGIGVHFKIYPF
          	  :::.** ***** **************. .*****  *:. *..::**:.********

MG09243.1 	IYAPAIVWWMDRERMQ-----RPGAASP---SFSSPASAAELITRFVTRERITLAGVSLA
NCU06057.1	IYAPAIVWWMDQERLSGVRAGGGGGGGG---QKKTSSSFRKTLTRFLTLPRLLLAFTSLA
FG09385.1 	IYAPAIVWWMDDARLG-----KETKAAP---QSSS---IKDAVANFFTPDRLKFGLLSLI
AN3370.1  	IYGMSILWWLDEKEFTTNKAQSESREVKPKFKDTPVGIFISQILSFITPCRIRLTLISLL
          	**. :*:**:*  .:    :        .. . .. .   . :  *.*  *: :   ** 

MG09243.1 	TFMGLNYAMYAL~YGTEFIVHTYLHHVSRIDHRHNFSPYNTLLYLNSASPP------SAS
NCU06057.1	TFLSLNFLMYRL~YGHPFLQETYLHHVTRIDHRHNFSPYNTQLYLSSASVSPSH--SAAE
FG09385.1 	TFMILNLVMFAI2YETPFLVHTYFHHVTRIDHRHNFSPYNVLLYLTSATPA------HAA
AN3370.1  	TFVALNAAMYLH~YGTPFLQHTYLHHLTRIDHRHNFSPYSTLLYLTAASSAGAVGHDAGG
          	**: **  *:   *   *: .**:**::***********.. ***.:*: . : . . . 

MG09243.1 	SPFRIESVAFLPQLLLSTVLIPLVLAKRHLPTSMLAQTFAFVTFNKVCTSQ0YFLWYMVL
NCU06057.1	PKFKIESLAFLPQLVLSTILIPLTLAKKDLPTSLLAQTFAFVTFNKVCTSQ~YFLWYLVL
FG09385.1 	PAFRIESFAFLPQLLLSCVLIPLALAKRDLATSMMAQTFAFVTFNKVCTSQ0YFLWYMIF
AN3370.1  	PSGSFESLAFIPQLLISVVVIPLVLGKKDLPGTMLAQTFAFVTFNKVCTSQ0YFLWYLIF
          	.   :**.**:***::* ::***.*.*:.*. :::**************** *****:::

MG09243.1 	LPLYLPHSSFLRSGRLGLAALLLWVAGQ~AAWLQQGYLLEFLGQSTFLPGLWVASLGFFL
NCU06057.1	LPLYLPRSSFWTSKRMGLVALGLWVLGQ~ALWLQQAYELEFLGRSTFLPGLWMASLGFFV
FG09385.1 	LPLYLPNSSFLRNGKLGIFALLLWIVSQ~AAWLQQGYELEFLGISTFYPGLWLASIAFFL
AN3370.1  	LPFYLPTSSLLRNPRLGIAVAALWILGQ0ALWLQQGYLLEFLGISSFLPGLFLASLGFFA
          	**:*** **:  . ::*: .  **: .* * ****.* ***** *:* ***::**:.** 

MG09243.1 	VNCWILGVIVSDVVNVPVQRASVKN---
NCU06057.1	VNCWILGVIVGDGGR-------------
FG09385.1 	VNCWILGVIISDGARQSTR-STVKFHVE
AN3370.1  	VNAWILGVIVADVGGLNLESGNEKRRVK
          	**.******:.*        .. .   .
```
