# Supplementary material for: Patterns of Intron Gain and Loss in Fungi
Source: PLoS Biol. 2004 Nov 30;2(12):e422. doi: 10.1371/journal.pbio.0020422 (PMC532390; doi:10.1371/journal.pbio.0020422)
Supplement: Table S1 — Also available at http://genes.mit.edu/NielsenEtAl/. (4.3 MB ZIP). [file pbio.0020422.st001.zip › NielsenEtAl/html/1047.html]

AN4463.1.NCU02510.1.MG07768.1.FG05619.1


```
 CLUSTAL W (1.82) Multiple Sequence Alignments - Introns Inserted


Sequence 1: NCU02510.1	1678 aa
Sequence 2: MG07768.1	1680 aa
Sequence 3: FG05619.1	1683 aa
Sequence 4: AN4463.1	1676 aa
Alignment Length: 1685 aa
Number Identitical Residues: 1087 aa
Alignment Score (without introns) 49927


MG07768.1 	-MAPLPIKFTELLQ~LSAVGVD~TSAIGFNSC0TLESDHYVCIREKKNEAASPEVVIIDL
NCU02510.1	MAQALPIKFQELLQ~LSALGIN~QTAITFNTC0TLESDNWVCIRDKKDEAASPEVIIVDL
FG05619.1 	-MAPLPIKFQELVQ~LANVGVD~TQSIGFNSC0TLESDSYVCVREKKSEAAQPEVVIIEL
AN4463.1  	-MAPLPIKFTELIN0LTNAEIA0PASIGFNTC0TLESDHFVCVRQKLDDEDKPQVIILNL
          	   .***** **:: *:   :    :* **:* ***** :**:*:* .:  .*:*:*::*

MG07768.1 	KNNNNVTRRPIKADSAIMHFTRQVIALRAQSRTLQIFDLEAKQKLKSTTMNEDVVFWKWI
NCU02510.1	KNGNNVIRRPIKADSAIMHWTRQVIALKAQARTLQIFDLENKAKLKSTNMSEDVLFWKWV
FG05619.1 	KNGNNVTRRPIKADSAVMHWNRQVIALKAQSRTLQIFDLEQKKKLKSCTMNEDVQFWKWI
AN4463.1  	KNNNEIIKRPINADSAIMHWSKNIIALRAQGRTIQIFDLSAKQKLKSAVMNDDVVYWKWF
          	**.*:: :***:****:**:.:::***:**.**:*****. * ****  *.:** :***.

MG07768.1 	SDTTIGLVTDAAVYHWDVFDANQATPVKQFARNDNLS0GNQIINYRANSEGKWMVVVGIA
NCU02510.1	SETTLGLITDGSVYHWDVFDPNQPAPVKVFDRNANLQ~GNQIINYRTSADGKWMVVVGIS
FG05619.1 	SENELGLVTTSSVYHWNVYDAGQDAPVKVFERNANLN0GCQIINYRVNSDGKWMVVVGIS
AN4463.1  	SERSLGLVTESSVYHWDVFDPTQAQPLKVFDRLPNLS0NCQIINYRVNDEEKWMVVVGIS
          	*:  :**:* .:****:*:*. *  *:* * *  **. . ******.. : ********:

MG07768.1 	QAQGRVVGNMQLYSKDRGISQSIEGHAAAFGTLRLEGAPQDTKVFTFAVRTATGAKLHIV
NCU02510.1	QQQGRVVGAMQLYSKDRGISQAIEGHAAAFGTLRLEGAPEDTKVFTFAVRTAVGAKLHIV
FG05619.1 	SQQGRVVGAMQLYSKDRGISQAIEGHAAAFGTLRLEGAPQDTKLFSFAVRTATGAKLHIV
AN4463.1  	SQQGRVVGSMQLYSKERGISQFIEGHAASFASIRVEGSPLEHKLFTFAVRTQTGAKLQIA
          	. ****** ******:***** ******:*.::*:**:* : *:*:***** .****:*.

MG07768.1 	EVDKPEANPAFAKKNVDVYFPAEAVNDFPVAVQVSQKYGVIYLVTKYGFIHLYDLENGTC
NCU02510.1	EVDHPETNPVFPKKAVDVFFPPEASNDFPVALQVSQKYGIIYLITKYGFIHLYDLETATC
FG05619.1 	EVDHPESNPVFQKKAVDMFFPPEATNDFPVALQVSQKYGVVYMVTKYGFIHLYDLETASC
AN4463.1  	EIDHQEPNPRFQKKAVEVYFPQEAVNDFPVAMQVSRKYDIVYLVTKYGFIHLYDLETGTC
          	*:*: *.** * ** *:::** ** ******:***:**.::*::************..:*

MG07768.1 	IFMNRISSETIFTTSPDGDSTGIVSINRKGQVLGVTIDDETMIPYLLQNPANTELAIKMA
NCU02510.1	IFMNRISSDTIFTACGDKDSTGVIGINRKGQVLFVSADENTIVPYVLEAHGN-ELAIKLA
FG05619.1 	IFMNRISSETIFTTCTDDGSSGIVGINRKGQVLFVTIDDSNVIQYLLQNPANTDMAIKMA
AN4463.1  	IFMNRISSETIFTTAPDSESAGLVGVNRKGQVLSVSVDENNIIQYLMENPAMSGLAVKLA
          	********:****:. *  *:*::.:******* *: *:..:: *:::  . : :*:*:*

MG07768.1 	SRAGLPGADQLYGQQFQQLFNGGNYMEAAKVAAGSPRGFLRTAETINKFKNLPQQPGQMS
NCU02510.1	SRAGLPGADNLYQQRFEQLFSNGNYQEAAKVAANSPRGFLRTPQTIERFKRLPQQPGQMS
FG05619.1 	SRAGLPGADNLYARQFEQLFNSGDYLAAAKVAANSPRGFLRSAETIEKFKRLPVQPGQMA
AN4463.1  	SKAGLPGADHLYQQQFDNLLAQGNYSEAAKIAANSPRGFLRTPETINKFKNAP-QTGQMS
          	*:*******:** ::*::*:  *:*  ***:**.*******:.:**::**. * *.***:

MG07768.1 	YILQYFGLLLDKGSLNHHETIELAQPVLAQNRKQLLEKWLNEGKLDCSEQFGDMVRPHDV
NCU02510.1	HILQYFGMLLDKGSLNEHETIELAQPVLAQNRKQLLQKWLSENKLECSERLGDMVRPHDI
FG05619.1 	FTLQYFGMLLDKGSLNKHETLELAQPVLQQNRKHLLEKWLKEGKLDCSEQLGDMVRPYDV
AN4463.1  	VILQYFGMLLDKGTLNKYESLELVRPVLQQNRKHLLEKWMRENKLESSEELGDIVRPYDM
          	  *****:*****:**.:*::**.:*** ****:**:**: *.**:.**.:**:***:*:

MG07768.1 	SMALKIYLKANVPQKVVAGLAETGQFDKILPYCAQTGYQPDWIQLLNHIVRINPEKGAEL
NCU02510.1	NMALAIYLKANAPHKVVAGFAETGQFEKILPYCGQANYQPDFIQLLHHIVRVNAEKGAEF
FG05619.1 	NMALTIYLKAEIPQKVVAGFAETGQFDKILPYSAQSGFQPDYIQLLQHITRVNPEKGAEF
AN4463.1  	NLALSIYLQANVPNKVIAGFAETGQFDKILAYSKQVGYQPDYTQLLQHIVRVNPEKGAEF
          	.:** ***:*: *:**:**:******:***.*. * .:***: ***:**.*:*.*****:

MG07768.1 	ATTLANHEGGSLVDIARVVDVFQAQGMVQQATAFLLDALKDNKPEHADLQTRLLEMNLMN
NCU02510.1	ATTLANHEGGPLVDFEKVVDIFQSQGMIQQATAFLLDALKDNKPEHAHLQTRLLEMNLVN
FG05619.1 	ASALANSEQGPLVDFERVCDIFQGQGMIQQATAFLLDALKENKPEHARLQTRLLEMNLMH
AN4463.1  	ATQLANEESGALIDLDRVVDVFLSQNMVQQATSFLLDALKDNKPEHGHLQTRLLEMNLVN
          	*: *** * *.*:*: :* *:* .*.*:****:*******:*****. **********::

MG07768.1 	APQVADAILGNEMFTYFDKGRIAALCEQAGLHQKALELYEDPAAVKRVVVNIAGMPNFNP
NCU02510.1	APQVADAILGNDMFSHFDKAHIANLCEQAGLLQKALELYEDPASIKRVIVNIPGMPNYNP
FG05619.1 	APQVAEAILGNEMFTHFDKTRIAQLCEQANLPQKALELYEDPEAIKRVVVNIPGQPNFNP
AN4463.1  	APQVADAILGNEIFTHYDRPRVSQLCENAGLIQRALENTDDPAVIKRNIVRT---DQLST
          	*****:*****::*:::*: ::: ***:*.* *:***  :**  :** :*.     : ..

MG07768.1 	EWLVNFFGKLSVEQSLDCLDAMMKTNIRQNLQSVVQVATKYSDLLGPTKLIDLFEKYKTA
NCU02510.1	EWLIEYFKALSVEQSLDCLDAMMKHNIRQNLQTVVQVATKYAELLGAQQLIDLFEKYKTA
FG05619.1 	EWLTTFFGKLSVEQSLDCLDAMMKANIRQNLQSVVTIATKYSELLGPVRLIDLFEKYKTA
AN4463.1  	EWLMNFIGRLSVEQTLDCMDTMLEVNIRNNLQAVVQICTKFSDLLGPSRLISLLEKYRTA
          	***  ::  *****:***:*:*:: ***:***:** :.**:::***. :**.*:***:**

MG07768.1 	EGLFYYLGSIVNLSEDPDVHFKYIEAATKMGQFSEVERICRDSNYYNPEKVKNFLKEAKL
NCU02510.1	EGLYYFLASVVNVTEDPEVVFKYIEAATKMGQIREVERICRDNSVYNPEKVKNFLKEAKL
FG05619.1 	EGLFYYLGSVVNLSEDPDVHFKYIEAATKMGQFNEVERLCRDSSVYNPEKVKNFLKEAKL
AN4463.1  	EGLYYYLGSIVNLSEDPEVHFKYIEAATAMNQISEVERICRESNYYNPEKVKNFLKEARL
          	***:*:*.*:**::***:* ******** *.*: ****:**:.. *************:*

MG07768.1 	TEQLPLIIVCDRFNFVHDLVLFLYQNQQFKSIEVYVQRVNPARAPAVIGGLLDVDCDEAI
NCU02510.1	SEMLPLMVVCDRFNFVHDLVLYLYQHQQFKSIEIYVQQVNPSRTPGVIGGLLDVDCDESI
FG05619.1 	PEQLPLIIVCDRFNFVHDLILYLYQSQQFAAIETYVQQVNPGRAPEVVGGLLDVDCDENV
AN4463.1  	TEQLPLITVCDRFNFVHDLVLYLYQNQQYKSIEVYVQRVNPSRTPAVVGGLLDVDCDESI
          	.* ***: ***********:*:*** **: :** ***:***.*:* *:********** :

MG07768.1 	IKNLLSTVDPASIPIDELVAEVETRNRLKMLLPFLEATLQAGNQQQAVFNALAKIYIDSN
NCU02510.1	IRNLLSTVNPASIPIDELVQEVETRNRLKLLLPFLEATLAAGNQQQAVYNALAKIYIDSN
FG05619.1 	IKQLLSSVNPQSINIDNLVSEVESRNRLKLLLPFLEATLQAGNQQQAVYNALAKIYIDSN
AN4463.1  	IKNLLTTVDPSVIPIDELVSEVETRNRLKLLLPFLEATLATGNQQQAVYNALAKIYIDSN
          	*::**::*:*  * **:** ***:*****:********* :*******:***********

MG07768.1 	NNPEKFLKENDQYDSLVVGKYCEKRDPNLAYIAYRKGGNDLELVNITNENSMYKAQARYL
NCU02510.1	NNPEKFLKENDQYDTLTVGKYCEKRDPNLAYIAYRKGQNDLELVNITNENQMYKAQARYL
FG05619.1 	NNPEKFLKENDQYDTLTVGKYCEKRDPNLAYIAYSKGQNDLELVNITNENSMYRAQARYL
AN4463.1  	NNPEKFLKENDLYDTLVVGKYCEKRDPNLAYIAYRKGQNDLELINITNENAMYRAQARYL
          	*********** **:*.***************** ** *****:****** **:******

MG07768.1 	LERADRELWMFVLSENNIHRRSVVDQVTSTAVPESTDPAKVSEAVAALLAADLPGELIEL
NCU02510.1	LERADRELWMFVLSENNVHRRSVVDQVISTAVPESTDPAKVSEAVTCFLNADLPGELIEL
FG05619.1 	LERSDAELWGFVLSENNIHRRSVVDQVTATAVPEANDPSKVSVAVSAFLENDLPLELIEL
AN4463.1  	VERADPEIWSFVLSENNMHRRSLIDQVVATAVPESTEPDKVSVAVKAFLEADLPGDLIEL
          	:**:* *:* *******:****::*** :*****:.:* *** ** .:*  *** :****

MG07768.1 	LEKIVLEPSPFSDNQNLQNLLMFTAAKADKSRVMDYIHKLDGFNPQEITAVCIDVGLYEE
NCU02510.1	LEKIVLEPSPFSDNQNLQNLLIFTAAKADKARVMDYIHKLDNFAADEIANVCIEVGLHEE
FG05619.1 	LEKIVLEPSPFSDNQNLQNLLMFTAAKADKARVMDYIHKLDNYNADEIATSCIEVGLFEE
AN4463.1  	LEKIILEPSPFSDNGSLQNLLMLTAAKADKGRLMDYIHQLNEFSPDEIAEMCISVGLYEE
          	****:********* .*****::*******.*:*****:*: : .:**:  **.***.**

MG07768.1 	AFEIFKKIDDKVSAVNVLVENVVSIDRAQAYAEDVDIPEVWSKVAKAQLDGLRVTDSIES
NCU02510.1	AFEVYKKIDNKEAAVNVLVEHVVSIDRAQAYAEEVDIPQVWSKVAKAQLDGLRVSDSIES
FG05619.1 	AFEIYKKADNKSAAVDVLIENVVSIDRAQAYAEEVDLPEVWSKVAKAQLDGLRVSDSIES
AN4463.1  	AFEIYKKVNNYISAVNVLVENIVSIDRAQEFAERVELPDVWSKVAKAQLDGLRVSDSIES
          	***::** ::  :**:**:*::******* :** *::*:***************:*****

MG07768.1 	YIKAEDPKNYLEVIEIATHAGKNEDLVKYLRMARKTLRETAIDTALAFCYARLDQLSELE
NCU02510.1	YIKAEDPKNYEEVIEVAVAAGKNEELIKYLRMARKTLREPVIDTALAFCYARLDQLPELE
FG05619.1 	YIKAEDPRNYLEVIEVATHAGKNEELVKYLRMARKTHREAAIDTALAFSYARLEQLSELE
AN4463.1  	YIHANDPSNYNEVIETATHAGKDEDLVKYLKMARKTLREPAIDTALAFCYARLDQLAELE
          	**:*:** ** **** *. ***:*:*:***:***** **..*******.****:**.***

MG07768.1 	DFLRGTNVTNIEESGDKAYGEGLFEAAKIFYTSISNWAKLATTLVHLGDYQAAVECARKA
NCU02510.1	EFLRATNVANVEESGDKAYAEGFFEAAKIFYTSISNWAKLATTLVHLSDYQAAVDCARKA
FG05619.1 	DFLRATNVANIEESGDKAYEEGLYEASKIFYTSISNWAKLATTLVHLGDYQAAVECARKA
AN4463.1  	DFLRSTNVADIEASGDKAYEEGYHQAAKIFYTSISNWAKLATTLVHLEDYQAAVECARKA
          	:***.***:::* ****** ** .:*:******************** ******:*****

MG07768.1 	NSIKVWKEVHEACVNKKEFRLAQICGLNLIVDAEQLQTLVKQYERNGFFDELISLLENGL
NCU02510.1	NNIKVWKEVHEACVNKKEFRLAQICGLNLIVDAEQLQALVKQYETNGYFDELISLLEQGL
FG05619.1 	NNIKVWKQVHEACVEKKEFRLAQICGLNLIVDAEQLQTLVKEYERNGYFDELISLLEQGL
AN4463.1  	NSVKVWKQVNQACVDKKEFRLAQICGLNLIVHAEELQDLVRQYERNGYFDELIAVLEAGL
          	*.:****:*::***:****************.**:** **::** **:*****::** **

MG07768.1 	GLERAHMGMFTELGIALSKYHPERLIEHLNLFWSRMNLPKMIRACEEANLWPELVFCYVH
NCU02510.1	GLERAHMGMFTELGIALSKYHPERLMEHLKLFWSRMNLPKMIRACEEANLWPELVFCYYH
FG05619.1 	GLERAHMGMFTELGIALSKYHPDRLMEHIKIFWSRMNLPKMIKACEEANLWPELVFCYYH
AN4463.1  	GLERAHMGMFTELGIALSKYHPDRVMEHLKLFWSRINIPKMIRACEEANLWPELVFLYCH
          	**********************:*::**:::****:*:****:************* * *

MG07768.1 	YDEFDNAALSVIERPENSWEHTQFRDIIVKVANLEIYFKAINFYLEQHPSLLTDLLQALT
NCU02510.1	YDEFDNAALAVMERPENSWEHQQFKEITVKVANLEIYYKAINFYLEQHPSLLTDLLQVLT
FG05619.1 	YDEFDNAALAVIERPENSWDHQQFKEIVVKVANLEIYYRAIKFYVEQHPSLLTDLLATLT
AN4463.1  	YDEWDNAALAMMERAADAWEHHSFKDIIVKVANLEIYYRALNFYLQEQPLLLTDLLQVLT
          	***:*****:::**. ::*:* .*::* *********::*::**::::* ****** .**

MG07768.1 	PRIDVNRVVRMFQKSDNLPLIKPFLLNVQTQNKRIVNDAINDLLIEEEDYKTLRDSVENY
NCU02510.1	PRIDVNRVVRMFQKSDNLPLIKPFLLSVQSQNKRTVNDAINDLLIEEEDYKTLRDSVNNY
FG05619.1 	PRIDVNRVVKIFQKNDDLPLIKPFLLNVQSQNKRVVNEAVNDLLIEEEDYKTLRDSVQNY
AN4463.1  	PRIDVNRVVRIFQASDNIPLIKPFLLNVQSQNKRAVNDAINDLLIEEEDYKLLRDSVDNH
          	*********::** .*::********.**:**** **:*:*********** *****:*:

MG07768.1 	DNYEPVELAGRLEKHDLVFFRQIAASIYRKNKRWEKSIALSKQDKLWKDAIETAAISVKS
NCU02510.1	DNYDAVDLAGRLEKHDLVFFRQIAASIYRKNKRWEKSINLSKQDKLWKDAIETAAISGKT
FG05619.1 	DNYDATELASRLEKHDLIFFRQIAASIYRKNKRWEKSIALSKQDKLYKDAIETSALSAKV
AN4463.1  	DNFDAVELAQRLEKHDLIFFRQIAANIYRNNKRWAKSIELSKQDKLYKDAIETAAISAKP
          	**::..:** *******:*******.***:**** *** *******:******:*:* * 

MG07768.1 	EVVEELLRY0FVDIGNRECYVGMLYACYDLIRPDLVLEMSWRHGLHDFTMPYMINLLSQQ
NCU02510.1	EVVEELLRY0FVDIGNRECYVGMLYACYDLIRPDLVLELSWRNGLNDFTMPYMINMLCQQ
FG05619.1 	DIVSDLLQY0FVDIGHRECYTGMLYACYDLIRPDLVLELSWRHGLMDFSMPYMINMLAQQ
AN4463.1  	DVVEELLRY0FVDIGSRECYVGMLYACYDLIRPDVILELSWRHGLQDFTMPFMINFLCEQ
          	::*.:**:* ***** ****.*************::**:***:** **:**:***:*.:*

MG07768.1 	TKELAVLKADNEARKAKEKEQEKKEDNAPILGGGRLMITAGPGTQ--PTSPAPFG-ANGF
NCU02510.1	TKELASLKADNEARKAKEQEKEKVEDNTPILG-NRLMITAGPG----QASPAPYGQTNGF
FG05619.1 	TKDLAALKADNEARKAKEQEKEKTDDNTPILGASRLMITAGPGGMGSAPSPAPYGQPNGF
AN4463.1  	TRTIEMLKKDNEERKAREVTQKKDEDNTPILGGSRLMLTQGPA----APAPPVYGQANGI
          	*: :  ** *** ***:*  ::* :**:****..***:* **.     .:*. :*..**:

MG07768.1 	APQPTGYGY-
NCU02510.1	VPQPTGFGF-
FG05619.1 	APQPTGYGF-
AN4463.1  	TPQATGFRPF
          	.**.**:
```
