# Supplementary material for: Patterns of Intron Gain and Loss in Fungi
Source: PLoS Biol. 2004 Nov 30;2(12):e422. doi: 10.1371/journal.pbio.0020422 (PMC532390; doi:10.1371/journal.pbio.0020422)
Supplement: Table S1 — Also available at http://genes.mit.edu/NielsenEtAl/. (4.3 MB ZIP). [file pbio.0020422.st001.zip › NielsenEtAl/html/1048.html]

AN6033.1.NCU08811.1.MG01472.1.FG09712.1


```
 CLUSTAL W (1.82) Multiple Sequence Alignments - Introns Inserted


Sequence 1: NCU08811.1	496 aa
Sequence 2: MG01472.1	490 aa
Sequence 3: FG09712.1	479 aa
Sequence 4: AN6033.1	506 aa
Alignment Length: 534 aa
Number Identitical Residues: 255 aa
Alignment Score (without introns) 11824


MG01472.1 	MAALASKQQSQKIFEKLKTKQANK0----------ICFDCGQKNPTWTSVPFGIYLCLDC
NCU08811.1	MSGLATKQQSLKLFEKLKAKPANK0----------ICFDCGQKNPTWTSVPFGIYLCLDC
FG09712.1 	MSSLATKQQSLKIFEKLKTKPANK0----------ICFDCGQKNPTWTSVPFGIYLCLDC
AN6033.1  	MS--ATKAESQKIFEKLKTKPANK0TDPVFAEITQICFDCGSKNPTWSSVPFGIYLCLDC
          	*:  *:* :* *:*****:* *** :..  :. :.******.*****:************

MG01472.1 	SSNHRNLGVHISFVRSTNLDQ1WQWDQLRVMKVGGNESATKFFQQNGGSAALNSKDPKTK
NCU08811.1	SANHRNLGVHISFVRSTNLDQ1WQWDQLRIMKVGGNESATKFFQQNGGSAALNSKDPKTK
FG09712.1 	SSNHRNLGVHISFVRSTNLDQ1WQWDQLRVMKVGGNESATKFFQQNGGTAALNSKDPKTK
AN6033.1  	SANHRNLGVHISFVRSTNLDQ1WQWEQLRIMKVGGNESATKYFQSNGGSAALASKDVKVK
          	*:******************* ***:***:***********:**.***:*** *** *.*

MG01472.1 	YHSAVATKYKEELKKRAARDAKE2YPEEVVITDGTD--AGDGTNTPAGEPDDDFFSSWDK
NCU08811.1	YQSAAATKYKEELKKRAARDARE2YPEEVVITDGDD--ATS-SNTPAGEPDDDFFSSWDK
FG09712.1 	YQSNAATKYKDELKRRAARDAQD2YPTEVIITDAID--DGS--ATPAGEPDDDFFSSWDK
AN6033.1  	YTSNAAVKYKEELKRRAALDAQE2YPEEVVITDVPAGATSNGSSTPAGD-DDDFFSSWDK
          	* * .*.***:***:*** **:: ** **:***   .:  ..: ****: **********

MG01472.1 	PAIKKPTPPVSRTATPPVVGRTPSPFLTAGGANGKDIARTPSPLAKSADSAVK--PAASR
NCU08811.1	PAIKKPTPPISRTSTPPVIGRTASPLLG----NGKDIQRTSSPLSKTDSDAPTPAPAASR
FG09712.1 	PAIKRPTPPVSRTGTPPVVGRTPSPFLNSG--NGKDIARTASPLSRTSTGENK---PASR
AN6033.1  	PSIKRPSNPPSRTGTPPVVSRTSSPFLN----AGANTARSKSPLSSDKESATAS-PAPTA
          	*:**:*: * ***.****:.**.**:*      * :  *: ***:    .   . ...: 

MG01472.1 	ITHSSALKKTTG-----AGPKKAN--VLGAKK-TTKLGVKKVN-AEVIDFDEAEKKAKEE
NCU08811.1	ITTSAALRKTTPGSSTTGGPRKVGGGILGAKKPAAKLGVKKIS-ADLIDFDEAEKKAKEE
FG09712.1 	ITTSAALRKTPAS----TGPRKAN--VLGAKK-TTKLGAKKVT-ADIIDFDEAERKAKEE
AN6033.1  	IRASAAARKTSGT----TTAKKGS--VLGTKK-APKLGAKKIGGADLIDFEEAERKAKEE
          	*  *:* :**.        .:* .  :**:** :.***.**: .*::***:***:*****

MG01472.1 	AERIEKLGYDPDDIDTTAAKKVGGVKAESSGAGILAPTPVSPARGGYGASGHSREKSASE
NCU08811.1	AERIEKLGYDPEAEEEKAAK-----KAETKTNNIITPAAAPVSSS--SSRSAAQEKSAAE
FG09712.1 	ADRIAKLGYDPDAEEDPATK------NSGSAAAIISPTPVAPSRG--SASSHTRQKSDAE
AN6033.1  	AERIEKLGYDPEAEEAEAAKT---KTSGTGATAIASPTPLSPNKVGFGATKTTHERNSSD
          	*:** ******:  :  *:*.    .       * :*:. .    . .:   ::::. ::

MG01472.1 	MERLGMGMGRLGFGQVG--------GNKPAAAAQPKKGGGFGSVGPIKATPEA1DEEKYA
NCU08811.1	VERLGMGVRKLGFGMVGKPGGAGAAAGAGGAAAAKKNAGGFGSVGPIKAS-EA1DEEQYA
FG09712.1 	VERLGMGMNRLGFGQVG-------GPKAAASSAPKRNAGGFGSVGPVRAQDVD1DSERYA
AN6033.1  	VERLGMGIGRLGFGQTV--------GSKPTAPAPKKLG--FGAVGAARSAEDE1EELQRT
          	:******: :**** .              :.*  : .  **:**. ::     :. : :

MG01472.1 	RSKFGAQKAISSDEFFGKGSYDPNAQAEAKTRLQGFEGASAISSNAYFGRPEEEE---VE
NCU08811.1	RNKFANQKAISSDEFFNKGNYDPSVKAETKARLQGFEGAQAISSNAYFGRPEDDAP--AE
FG09712.1 	REKFGTQKGISSDEFFGKGAFDPSQQSEAKTRLQGFEGATAISSNAYFGRPEDEP---EE
AN6033.1  	KNKFGAQKGISSDEFFGRDRFDPVAQSEAKERLRQFDGAQAISSNSYFGRPEDDYPPVDD
          	:.**. **.*******.:. :**  ::*:* **: *:** *****:******:: ..  :

MG01472.1 	EYGDLETAAKDFIRKFGLTRGDDLENLTAVLGDGATKLQG~-AIRSYLGS---------
NCU08811.1	DYGDLESAAKDFIRKFGITASDDLENLTQMVGEGAGRLQG~-AIRAYLGS---------
FG09712.1 	EYGDLESAAKDFVRKFGITAGDDLENLTQMAGEVSTRLQG~-AIRSYLGN---------
AN6033.1  	TYGDLEAAAKDFVRRFGITAGDDLENLTQLVGDGASKLQA1KRVQEFLFHNDILSLRTR
          	 *****:*****:*:**:* .******* : *: : :**. . :: :*  ..  :  :
```
