# Supplementary material for: Patterns of Intron Gain and Loss in Fungi
Source: PLoS Biol. 2004 Nov 30;2(12):e422. doi: 10.1371/journal.pbio.0020422 (PMC532390; doi:10.1371/journal.pbio.0020422)
Supplement: Table S1 — Also available at http://genes.mit.edu/NielsenEtAl/. (4.3 MB ZIP). [file pbio.0020422.st001.zip › NielsenEtAl/html/1049.html]

AN0692.1.NCU06659.1.MG04106.1.FG09924.1


```
 CLUSTAL W (1.82) Multiple Sequence Alignments - Introns Inserted


Sequence 1: NCU06659.1	419 aa
Sequence 2: MG04106.1	415 aa
Sequence 3: FG09924.1	412 aa
Sequence 4: AN0692.1	385 aa
Alignment Length: 419 aa
Number Identitical Residues: 260 aa
Alignment Score (without introns) 11988


MG04106.1 	MPRDPLIGL~VGKPSAGKSSTLNS~LTDATS~KVG~--R2FTTIDPQRAIGYLQID~CAC
NCU06659.1	MPRDPLIGL~VGKPSAGKSSTLNS~LTDASS~KVG1NFP2FTTIDPQRAIGYLQID~CAC
FG09924.1 	MPRDPLIGL0K---DPGQS--CRN~LALIAS~YIG~NFP2FTTIDPQRAIGYLQIE~CAC
AN0692.1  	MPRDPLIGL~VG-----------K0MLLRKS0ETS~---~---------------H1CAC
          	*********  .           . :    *   .                    . ***

MG04106.1 	ARHGLQDRCKPNHGSCVDGRRSVPIELLDVAGLVPGAHQGKGLGNKFLDDLRHADALIHV
NCU06659.1	ARYNVSERCKPNYGSCVNGKRSVPIELLDVAGLVPGAHEGKGLGNKFLDDLRHADALIHV
FG09924.1 	TRFNVSDRCRPNYGACVEGRRSVPIELLDVAGLVPGAHEGRGLGNKFLDDLRHADALIHV
AN0692.1  	KRYGVADKCKPNYGACTDGKRSVPIELLDVAGLVPGAHQGRGLGNKFLDDLRQADALIHV
          	 *..: ::*:**:*:*.:*:******************:*:***********:*******

MG04106.1 	VDASGTTDAEG~KNTRGYDPSVDIAWLRSEIVAWIRGNLMDKW~GSIRRRHVAAK~HTAV
NCU06659.1	VDASGTTDAEG~KVTRGYDPSVDIAWLRSEIVAWIKGNLWEKW~GSIKRRHIAVK~ATAV
FG09924.1 	VDVSGTVDAEG~KETRGYDPSVDIAWLRSEIVAWVLGNLMQKW~GSIRRRHQAIK~ATAT
AN0692.1  	VDVSGTTDAEG1KSTRGYDPSQDIEWLRSEIVRWVLGNLMQKW2GSIKRRHMAIK1ATAM
          	**.***.**** * ******* ** ******* *: *** :** ***:*** * *  ** 

MG04106.1 	ETLQAQFSGYGSTAKVVARTLDKLALKEGLEHWDD~ATIERVVEAFTDEKFPTVIALNKI
NCU06659.1	ETLQAQFSGYGSTAAVVARTLDKLGLKEPLEEWSE1ETVDRVVNAFTDEKFPTVIALNKI
FG09924.1 	ETLQGQFSGYGSTSTTVNRALDRCGLKEPLEDWSN~ETVEMVVNAFIDEKFPTVIALNKI
AN0692.1  	ETLQNQFSGYGSTPSTVARCLDRLALKEPLEEWSD~ETVEQVVQAFIDEKFPTVFALNKI
          	**** ********. .* * **: .*** **.*.:  *:: **:** *******:*****

MG04106.1 	DHPDSDK~NIAKIAKMQDPNKIVLCSAISEIFLRKMAKQGYIRYTEGSEFVDTREDLVAD
NCU06659.1	DHPDADK~NIAKIAKMQDPNSIVLCSAISEIFLRKMAKQGYIKYTEGSEFVDTREDLIAD
FG09924.1 	DHPDADK~NIAKIAKQQDPNTIVLCSAISEIFLRKMAKQGYVKYVEGSEFVDTKEDLIEQ
AN0692.1  	DHPDADK0NISKIAKMQDPQRIVLCSAISEVFLRRLAKQNYIKYTEGSEFLDTREDLIAD
          	****:** **:**** ***: *********:***::***.*::*.*****:**:***: :

MG04106.1 	GDPEGGGLKELDEKNKT~RIENLKDMVLYRFGSTGVNQVLSKAAEVLGLVPVYPVRNTTT
NCU06659.1	GDPDGGGLKELDEKNRN~RIENLKDMVLYRFGSTGVNQVLSKAAEILGLVPVFPVRNTTT
FG09924.1 	GDPTGGGLKDLDEKNRT~RIENLKDMVLYRFGSTGVVQVLSKAADLLGLVPIFPVRNTST
AN0692.1  	GDPDGGGLREMDEKLKT2RVENLKDMVLYRFGSTGVVQCLSRAAEVLGLVPVFPVRNLHT
          	*** ****:::*** :. *:**************** * **:**::*****::****  *

MG04106.1 	FGSGASES--KFVFRDCVLVKK~NTTVGEVARKVMGDAPVAYIEGVGAMRVSEDVVVTVG
NCU06659.1	FTSGANESANKAVFRDCVLVKK~NSTVADVARKIMGDAPIAYVEGAGGIRVAEDQIVTVG
FG09924.1 	FSSGASES--KFVFRDCVLVKK~GSTVGDVVRKVMGDAPIAFVEGAGNIRVSEDDTVAVG
AN0692.1  	FSSGTGTA----AFRDCVLVKK2NSTVGDVARKVMGDVPISYIEGVGGVRVSEDEIVAVG
          	* **:. :    .********* .:**.:*.**:***.*::::**.* :**:**  *:**

MG04106.1 	KND0ILSFKVGRG
NCU06659.1	KND0ILSFRVGRA
FG09924.1 	KND0VLSFKVGRA
AN0692.1  	KHD0VLSFKPGR-
          	*:* :***: **
```
