# Supplementary material for: Patterns of Intron Gain and Loss in Fungi
Source: PLoS Biol. 2004 Nov 30;2(12):e422. doi: 10.1371/journal.pbio.0020422 (PMC532390; doi:10.1371/journal.pbio.0020422)
Supplement: Table S1 — Also available at http://genes.mit.edu/NielsenEtAl/. (4.3 MB ZIP). [file pbio.0020422.st001.zip › NielsenEtAl/html/105.html]

AN0577.1.NCU06601.1.MG06118.1.FG05259.1


```
 CLUSTAL W (1.82) Multiple Sequence Alignments - Introns Inserted


Sequence 1: MG06118.1	726 aa
Sequence 2: FG05259.1	696 aa
Sequence 3: NCU06601.1	703 aa
Sequence 4: AN0577.1	642 aa
Alignment Length: 738 aa
Number Identitical Residues: 167 aa
Alignment Score (without introns) 10617


MG06118.1 	MPLRVKSKPAAQQANSRVRSPSPIGRHLDSKLPDIEDGDSFRELTKKLS~V~YIAAVIRL
NCU06601.1	MTVFGSSS--RNYPCEPTCPINTIGRVADREI---------------LF~R~YIAENIRL
FG05259.1 	MPIKVKSK---KLPAPRDGSPISIRHDYENDLPDIRTTDSFREVVKKLS~L2YFVAVIEL
AN0577.1  	MLHESMAS---LAPSINDRVEIPIIDDDEPLVD---------VVQKLHS2F~MISSISEV
          	*     :.     .        .*    :  :             .       :    .:

MG06118.1 	PYTFEQLRTTAAGEALIVLVQHLSTCCKNPAIVNSLL2ALKWHYGVSTEDK-GIAEARAN
NCU06601.1	PSTFEQLRTTAAGDCLRHLVDHLVLNCDHPAIVNALL2ALKWHYGAITEDK-GLNDARAS
FG05259.1 	PSTFEQLRTTAAGTSLRILVDHLTATCTNRGIVNALL2ALKWHYTTGSEHR-TLGESRAD
AN0577.1  	WYKFEELKLAPD-PRLRALVNSLAEDSHNPRIVPALM2ILKWRFDKFAEHDDGQNETRGY
          	  .**:*: :.    *  **: *   . :  ** :*:  ***::   :*. .   ::*. 

MG06118.1 	ACEIVAWRFLTHLSEREAVEYCLYEIPDPDDVDSNGEAENGGDEENGHRHIDENTALLPH
NCU06601.1	GCEIAAWRLLTHLSEREAVDYCLYEIP------DPALEANRQSDEEETSQVNEHSPLLAR
FG05259.1 	ACEIVAWRFLTRLSEREAVNYCLYEIP---DLDDDHQDEEAGNDHAGDEEAGEHSPLLSH
AN0577.1  	ACEYVAWQFLCHLNERELIEYLLEELK-------PPTQYTSSIDSAEAGIGGRATCMVEN
          	.** .**::* :*.*** ::* * *:                 :       .. : :: .

MG06118.1 	GWSGNGFIGSGRIIPERP-ASRKRYQLLQSLSSLTMSIK--QDDDDDDEQDPTAPFKTLN
NCU06601.1	AWTESVANPRRKHEFPNPTGDNRRNLLLQSLTRLTMSMTGEVDDDGEEEDDPTAPFINLN
FG05259.1 	IRT----IDNRRRGPGPAGSSMKRNQLLSSLSRLTMTS---DDSDDENEGDPTSSFKNLN
AN0577.1  	NLPGDEHTPLLSSSTTRSQLLERNRQHEDYFGSEHLETN---NYFPDGDGDGFSMFLGLN
          	  . .            .    :.    . :    :  .   :   : : *  : *  **

MG06118.1 	GLEIAAVADAKRFLSQTIVQKIITGIWEGDIVFWEDLTIHSVKRPKFYKPH~TADPFSRL
NCU06601.1	ALEIAAIADAKRFLSQNVVQKIITGIWNGDIIFWDNLSVHTEKKPRFYNPR~TADPFSRL
FG05259.1 	ALEIAAIADAKRFLSQHIVQKIITAIWSGDIVFWDSLSVHSTKKVRFYNPR~TADPFSRL
AN0577.1  	ALEIATIAHAKKFLSQKVVQKVVNDIWNGEIVFWDSLSVHSTKKPQRFNKR2TADPYSRL
          	.****::*.**:**** :***::. **.*:*:**:.*::*: *: : :: : ****:***

MG06118.1 	RVPKYLKSFEVLFFLSFLCLYYAVLNNRDDSSRITTLEAVLYVWFAAFFYDELSEWADAG
NCU06601.1	RVPKYLKSFEVVFFASFLFLYYAVLVERNPT-RITPLEIFLYLWLAAFSYDELSEWIDAG
FG05259.1 	RVPKYLKSWEVVFFCIFLALYYSVLIMRDES-RITIPEVVLFLWIAAFFYDELSEWLDAG
AN0577.1  	RVPVYRKAFEAGFFVSFLLLYYAVLLERNPA-RVGNFEAILYVWIAAFAYDELSGLVDSG
          	*** * *::*. **  ** ***:**  *: : *:   * .*::*:*** *****   *:*

MG06118.1 	SIFYATDVW~NLFDMVMLLIGIAFVSLR~VVGLYRGDQHLTETAFDILSLEALFMVPR~I
NCU06601.1	SIFYAADIW0NIFDMAIMAIGFTFMVLR1IIGLETQNAELIDLAFDVLALEALLMVPR~V
FG05259.1 	SIFYATDIW~NLFDMIMITIGFAFAILR1IIGIVQNKPEFSDLAFDILALEALFMIPR~I
AN0577.1  	MLFYQMTFW~NLWDLCIIGTGLVFVVTI1FCACSMDS---------LLKTLPRNYRLR2I
          	 :**   .* *::*: ::  *:.*     . .    .         :*   .     * :

MG06118.1 	CSVLSLSPYWGTL~IPCLKEMGKDFVKFMVLVVIVYLG~FLTTFSQLARHRFGLANMTMF
NCU06601.1	CSILSLSPYWGTL~IPCLKEMAKDFIKFMVLVVIIYLG~FLTTFSLVGRDSFPLRDMTWF
FG05259.1 	FSILSLSPAWGTL~IPCLKEMGKDFFKYMVLVVVVYCG~FLTTFSLVGRNVFAFKSMAWI
AN0577.1  	FSLVSLNPYFGSL0T-------KAFFRFVPVIIILYLG1FLTTFTMLARDRLSSKNMSWI
          	 *::**.* :*:*         * *.::: :::::* * *****: :.*. :   .*: :

MG06118.1 	LTKIFFG~SSYVGFDIMRDIDPVFGPPLMLTFI~TLSSIL~LMGSLT~GMLSNSFSR~VI
NCU06601.1	LTKIFFG~STFIGFDIMRQIDPVFGPPLMILFV~MLSSIL~LMGSLT~GMLSNSFSR~VI
FG05259.1 	LTKIFYG~SAPIGFEVMNQIDPFFGPPLMIMFI~TLSSFL~LMGSLT~GMLSNSFSR~VI
AN0577.1  	LVYVFFG2--------------------TFAMI2LRMRVD0ARFCLV1SLMSMSMEG0VM
          	*. :*:*                      : ::     .     .*. .::* *:.  *:

MG06118.1 	SHAREEYLYV~YSVYVLEASTSNRLTHFYPPF~NLIALVIFRPLRLFLPSDHKFRAARIA
NCU06601.1	THAREEYLFV~YSIYVLEASTSNRLTHFYPPF~NLLALVIFRPLRLFLPSDDKFRRARII
FG05259.1 	THAKEEYLYV~YSVYVLEASTSNRLTHFYPPF~NLLALVIFRPWRLIFTADEKFRAGRII
AN0577.1  	SHAREEYLFQ2LAIYVLESSTSKRLTYFMPPL0NLIPLLCIRPMRLFLP-AAEIRRVRII
          	:**:****:   ::****:***:***:* **: **:.*: :** **::.   ::*  ** 

MG06118.1 	LLKATHWPIVGVIQLYELIRGKFRPTDKFRGPVPDSLSAGASPRKMQKRPALQGQRSGSS
NCU06601.1	LLKATHLPIVFIIAMYEWIRGTSLRGATYSAVKAPPLGAPIASPAMMRKLAGPRREMRGR
FG05259.1 	LLKATHWPIVGIIKLYEMYRKPGALGDEFAGFKGPSHSSRRNGRQLITQ-----KRSTST
AN0577.1  	LLRATHLPCVALLWAYESSRRFLSRRNSLASARMTPKRLSRPTSTIQARSGPYSHHVMAL
          	**:*** * * ::  **  *          .    .         :  : .   :.  . 

MG06118.1 	LNDPSPWQRQAPRT----TSGADYSNRQQVLQSASQSRVDMVEDTGMAGPTDVEVRISEL
NCU06601.1	RSVTSLQSDQAAQS----RLGMSSMERRISAHHARQAEEAQEDSTYAERATDVEVRIADL
FG05259.1 	VGRP---ALASPRG----RANG---------QSTEQR-----EDD-MDAPTAVEVQLSEL
AN0577.1  	TSGLDVHSRASLRTRRHS---------------------------QHEQRPGQEEEVADM
          	 .  .     : :    :                                .  * .::::

MG06118.1 	SNKIDRLTDLVLAMQNQQALQT-QQTTQVSV---
NCU06601.1	AAKIDRLTELVISMQSQQSTTLGDVSLS------
FG05259.1 	NRKIDQLTAAVLVMQEKQAGGSGGESSNASTAAA
AN0577.1  	LDEMERLRAQMGRVAAALGVHHRRRQ--------
          	  ::::*   :  :    .
```
