# Supplementary material for: Patterns of Intron Gain and Loss in Fungi
Source: PLoS Biol. 2004 Nov 30;2(12):e422. doi: 10.1371/journal.pbio.0020422 (PMC532390; doi:10.1371/journal.pbio.0020422)
Supplement: Table S1 — Also available at http://genes.mit.edu/NielsenEtAl/. (4.3 MB ZIP). [file pbio.0020422.st001.zip › NielsenEtAl/html/1050.html]

AN6618.1.NCU03661.1.MG04067.1.FG05191.1


```
 CLUSTAL W (1.82) Multiple Sequence Alignments - Introns Inserted


Sequence 1: MG04067.1	833 aa
Sequence 2: FG05191.1	830 aa
Sequence 3: NCU03661.1	884 aa
Sequence 4: AN6618.1	831 aa
Alignment Length: 918 aa
Number Identitical Residues: 423 aa
Alignment Score (without introns) 21237


MG04067.1 	MTS-NSHPSDNNP--------------------------------------------AQR
NCU03661.1	MTQIKKMTSSITPPVTDISTSTSTSTSAGTTTTTTGSIATAAPATVLLTHPTPVPTSRPQ
FG05191.1 	MSQQNSRAADAPPQQ-----------------------------------------SAQR
AN6618.1  	MTSSNVQFTPPPS-----------------------------------------------
          	*:. :   :   .                                               

MG04067.1 	PASPAGSYYAMSDDEEGDYNTITHTESGRG~VKLLYSKSK0VYIHPTPSSKDNIPGFIAL
NCU03661.1	PSSPTGSYYAMSDDEEGDYNTITHTSMGRG0---------~VYIHPTPSAKDNIPGYIAL
FG05191.1 	SASPSNSFYALSDDEEGEYNTIRNEETGRG~VKLLFSKSK0VYVHPTPSSKDNIPGYVAL
AN6618.1  	PPSPTASFFDVSDEEEDEYNTIAHSTPKKG~VRLLFSKSK0VYVHPTPSAKDNIPGFIAL
          	..**: *:: :**:**.:**** :    :*      :.:. **:*****:******::**

MG04067.1 	LQQKPARNETRPNSSSS-AKS-NPASSDLLLAWLPESSLGDSASIYVKVDLCEGDSPPKQ
NCU03661.1	LQQRNQHRDDRPVSSSSRDPSNTPASSDLLLAWVPESQLGEAANLYVKVDLCDGDSPPKQ
FG05191.1 	LQQR-GHQEERPSSSSS-HDSQKIASSDLLLAWIPESSLGDSASIYVKVDLCDGDTPPKQ
AN6618.1  	VQQKPLPSTQKTTSSNS--NASRPDLSSFLLAWVPESALGDAYDTYVKVDLSEDDSPPRQ
          	:**:      :. **.*   :.    *.:****:*** **:: . ******.:.*:**:*

MG04067.1 	SYLVPPPPTVTTHSGSIGHYAFAVPVSAIYSLLVRPPSLGWWFGSVIINTRAGDSFPALF
NCU03661.1	SYLVPPPPTVTTHRGSVGPYAFAIPVSAIYSLLIRPPSVGWWWGSIIINSRGGDSFPALF
FG05191.1 	SYLVPPPPTVTSHVGSVGGYAFAIPVNAVYSLLVRPPSLGWWYGSVIINSRAGDSFPALF
AN6618.1  	RYLVPPLPETTTFKDPIGLYAFAVPLSQIYSLLVRPPSLGWWFGSLVINTRAGDSFPALF
          	 ***** * .*:. ..:* ****:*:. :****:****:***:**::**:*.********

MG04067.1 	FHDSECQSTMLRKKKITRDSFDPFGEGGQMFWGGDEVLRWLRRYVKVERSGAEPNIYLVE
NCU03661.1	FHDSECQSTILQKKKRTADTFDPFGEAGQMFWGGDEVLRWLRRYVQIERSGAEPNMYLVE
FG05191.1 	FHDNECQSTMLQKKKIARDTFDPFGESGQMFWGGDEVVKWLRRYVKIERSGAEPNIYLIE
AN6618.1  	FHDSECQSTILQKKKRARETFDPFDEDGSVFWGGDEVLRWLRKYVDVQRSTVDHTVYLIN
          	***.*****:*:*** : ::****.* *.:*******::***:**.::** .: .:**::

MG04067.1 	PSKEDSEAFGGNLTSSSPSQIGKRDSARGIGSFAAAGSSTSADAGMDPFVKFVKETGWNI
NCU03661.1	PSKEDSVAFGGKPTTNTATQAG---SSRGG---PGGANAKDQDAGMDPFVKLIKEAGWNL
FG05191.1 	PSKEDSEAFGHKLTSN-ASQIGNQDSSTGAQQRSAGGPS-SKDAEMDPFVKLIKETGWNL
AN6618.1  	PSEEDQLSFG-KPQLTEAAGSQDKPSPRKN-------ESAPHDAGMDPFMKAIKETRWRV
          	**:**. :** :   . .:   .  *.           :   ** ****:* :**: *.:

MG04067.1 	MEKFSKVTTMTRQAAQDLMENPSMPPQVRRLMRNPEVQTIQDEFDSARIYLARWAMGIAE
NCU03661.1	MEKFSKVTTFTRQAANDVLDNPRVPPQMRRLLRNPEVQTLQDEFDSARIYLARWAMGIAE
FG05191.1 	MEKFSKVTTFTRRAAQDIVENPNLPPQVRRLLRNPEVQTLQDEFDSARIYLARWAMGIQE
AN6618.1  	LEQLSKITTFTRRTANEIAENPRIPPQVRRLLKTPEIQTLQEEFDSARIYLARWAMSISE
          	:*::**:**:**::*::: :** :***:***::.**:**:*:**************.* *

MG04067.1 	QSERDRRQRIWTARDVMELEDTDVGEFELLEGATGAMSLEQQRKRVTMSEWKGFFDARTG
NCU03661.1	QSDRDRNRRIWTARDVMELEDTDVGEFELVDGAN-SLSLEERRRVLTLKEWNSFFDEESG
FG05191.1 	QSDRDRRQRIWSANDVMELEDTDVGEFELLEGAS-NLSLEERRKVVTMKEWNTFFDPTTG
AN6618.1  	QSERERNRRIWTARDTLEMENSAVGDFEILEAEMGNMALQERRKVVTLKEWQGFFDQQTG
          	**:*:*.:***:*.*.:*:*:: **:**:::.  . ::*:::*: :*:.**: ***  :*

MG04067.1 	RLTYTIDEVKERIFHGGLDPDDGVRKEAWLFLLGVHDWYSTADERKAQVASLRDGYVKLK
NCU03661.1	RLSVTVDEVKERIFHGGLDPEDGVRKEAWLFLLGVYDWYSTADERKAQAASLRDAYIKLK
FG05191.1 	RLSVTIDEVKERVFHGGLDPDDGVRKEAWLFLLGVYEWYSTADERKAQIASLRDHYYKLK
AN6618.1  	RLQVTVDEVKERIFHGGLDPNDGVRKEAWLFLLEVYPWDSDSEDRQALMNSRRDEYIRLK
          	**  *:******:*******:************ *: * * :::*:*   * ** * :**

MG04067.1 	GAWWERLVDLGGKGEAGEW~WREQRGRIE1KDVHRTDRTVPIFAGENIPHPDPDSPFASS
NCU03661.1	GAWWERQVDLGGEGE--EE1IPNTVVLTE~KDVHRTDRNVPIFAGEDIPHPDPDSPFAST
FG05191.1 	LSWWERLEGDGGEGETGEW~WREQKSRIE1KDVHRTDRHVPIFMGEDTPHPDPSSPFAEV
AN6618.1  	GAWWERMVEGDSTPKQQEW~WKEQRNRIE1KDVHRTDRTIPLFAGEDIPHPDPDSPFADV
          	 :****    ..  :  *    :     * ******** :*:* **: *****.****. 

MG04067.1 	GTNVHMEQLKDLLLTYNEYNQELGYVQGMSDLLAPIYAVVQDDAIAFWCFQRFMDRM~ER
NCU03661.1	GTNVHMEQLKDMLLTYNEYNKGLGYVQGMSDLLAPIYAVLQDDALAFWAFQHFMDRM~ER
FG05191.1 	GTNVHLEQMKEMLLTYNEYNKDLGYVQGMSDLLAPIYAVIQDDAVAFWGFQKFMERM~ER
AN6618.1  	GTNVHLEQMKDMLLTYNEYNPDLGYVQGMSDLLAPIYAVMQDDAVAFWAFANFMNRM0ER
          	*****:**:*::********  *****************:****:*** * .**:** **

MG04067.1 	NFLRDQSGMRAQLLALDHLVQFMDPKLYAHLQSADSTNFFFFFRMLLVWYKREFEWLDVL
NCU03661.1	NFLRDQSGMRAQLLALDNLVRFMDPKLYAHLESADSTNFFFFFRMLLVWYKREFEWADVL
FG05191.1 	NFLRDQSGMRNQLLTLDQLVQFMDPVLWNHLQKADSTNFFFFFRMILVWYKREFEWLDVL
AN6618.1  	NFLRDQSGMRAQLLTLDHLVQLMDPQLYLHLQSADSTNFFFFFRMLLVWYKREFEWVDVL
          	********** ***:**:**::*** *: **:.************:********** ***

MG04067.1 	HLWEVLWTDYLTSSFHLFFALAILEKHRDVIMTHLKHFDEVLKY-~-------------V
NCU03661.1	RLWEALWTDYLSSGFHLFIALAILERHRDVIMTHLKHFDEVLKY-~-------------V
FG05191.1 	RLWEGLWTDYMSANFHLFIALAILERHRDVIMEHLQHFDEVLKY-~-------------I
AN6618.1  	RLWETLWTDYLTSNFHLFIALAILEKHRDVIMDHLKQFDEVLKYS1DGLCSACANRLGIV
          	:*** *****:::.****:******:****** **::*******: .. .::.:.  . :

MG04067.1 	1NELSCTMDLESTLIRAEALFRRFQRLVEAIDKKKNFPAPRTTRSSSTATSSSTNGTVKA
NCU03661.1	1NELSTTIDLESTLIRAEALFQRFRRLVEAIDRKDHFPGPRRPLSTASSSSVSAALADAE
FG05191.1 	1NELSTTIDLEATLIRAESLFKRFQRLVDAVDKKQNFPAPRFNPKNSSGSSESAE-SGPS
AN6618.1  	~NELSNTMDLIPILTRAETLFHRFGRQIEAIDKKNNFPTP---PGQPQAQRPTPAQPQSS
          	 **** *:** . * ***:**:** * ::*:*:*.:** *      . .   :.  .   

MG04067.1 	GDEPST---------GPRSPS----------QNKDKAPDEQEDV----VKVITPELRGLL
NCU03661.1	GSKSSSPPLPPRPQSGPRTASSPSAATTKDNKGKTTATATAEEDKAIVEKIVTPALRKLL
FG05191.1 	KDGKTK---------DGKTPA----------KGKAKEP-EAPPQ----PKTITPELRKLL
AN6618.1  	KGKSPE---------RQLAAS----------TGVSSSTQAGPGSKPEAAKIIPQELRDLF
          	 .  .             :.:           .  . .      ..   * :.  ** *:

MG04067.1 	SREVAILPRNEVAQKGEGWATVTK-
NCU03661.1	SREVEIIR--APAREGAGYPTRERA
FG05191.1 	SKEVEVLPRTAVAQNGDGMPNK---
AN6618.1  	RKDVFWNG---NSQHSNSKP-----
          	 ::*        ::.. . .
```
