# Supplementary material for: Patterns of Intron Gain and Loss in Fungi
Source: PLoS Biol. 2004 Nov 30;2(12):e422. doi: 10.1371/journal.pbio.0020422 (PMC532390; doi:10.1371/journal.pbio.0020422)
Supplement: Table S1 — Also available at http://genes.mit.edu/NielsenEtAl/. (4.3 MB ZIP). [file pbio.0020422.st001.zip › NielsenEtAl/html/1051.html]

AN0498.1.NCU09444.1.MG04897.1.FG07509.1


```
 CLUSTAL W (1.82) Multiple Sequence Alignments - Introns Inserted


Sequence 1: MG04897.1	638 aa
Sequence 2: FG07509.1	599 aa
Sequence 3: NCU09444.1	664 aa
Sequence 4: AN0498.1	545 aa
Alignment Length: 691 aa
Number Identitical Residues: 186 aa
Alignment Score (without introns) 10798


MG04897.1 	MTSTGARENISTDENGAFDVETS----TANGSMKRDEPSQPPKTPAVSRPNEDTEILYSY
NCU09444.1	MADPTPGHTAPDTAPPSWRSEDNRSPVLAPHPATVPSTTLPPPSNNTQLLDANTEITHLY
FG07509.1 	MTEPGLRQTADTPPQDLGDLEIG----LEN---------TVPASTTQSKLGSQDPISYFY
AN0498.1  	MAPHSSASSSTQIEPDPQQAQYE-----------------HELETEKKPACLDQEIEYLY
          	*:      .           :                          .    :  * : *

MG04897.1 	LTFDTPLPVVNTRP------EREDQP---PPPPQPDLSRFSNPIYWPQHRKNVLLALSCI
NCU09444.1	LTFAILPPSANTTHQSSGSTTTTTTPQNDPPPPAPNLKPFTNPILWPTSVKITMLTLSCI
FG07509.1 	LTLDTPPPTVPSYHGS----TPSDTP---SLPQCPDLRPYASPFTMPRARKNIMLALSCI
AN0498.1  	LELDTPLPTPWITAPP----GPGQSP----APEAPNLEKYTSPFLWPKWRKSMMTWIACG
          	* :    *       .         *     *  *:*  ::.*:  *   *  :  ::* 

MG04897.1 	ATFLTAYTAGSYSPPARTIARDLDNGTASHLGVLAGISTFCLGFALAPMVLAPVSEINGR
NCU09444.1	ATFLTAYTAGAYSPPQKLLLQDLAH-AHSSTAILGGISTFCLGFALAPMFLAPFSEMNGR
FG07509.1 	ATMLTAYTAGTYSPPSRAMAKDIG---ASHTATLVGITTFCAGFAFAPMALAPISEIWGR
AN0498.1  	VTGLAGYSAGEITPASSQLTDDWD---ISAVVYNLGITLFCIGFALAPMVLAPFSEINGR
          	.* *:.*:**  :*.   :  *      *      **: ** ***:*** ***.**: **

MG04897.1 	YPVFIVANVVFTIFQAACGAVNSLAGMLLCRFGTGVGGSVFSSMVGGVIADLWDAEGRNT
NCU09444.1	QPVFVAAGIMFVVFQVVCGVVETLEGMLIARFFLGVGASVFSTMVGGVIADIWPTAERNT
FG07509.1 	RPVFILAGFVFVIFQAVCSVMPNLVGMLIARFFVGVGGSVFSSVVGGVIADLWEKEERNT
AN0498.1  	RPIFVASGVLFVACIVACGGTRQFGGFLVARFFQGVGASTFSTMVGGVISDIYHAHERNT
          	 *:*: :..:*.   ..*.    : *:*:.**  ***.*.**::*****:*::    ***

MG04897.1 	PMTLFSCAVLLGTGIGPMVGASSFFTM---------EGK-GMWRWVFYHQAIASAVLMIV
NCU09444.1	PMALFSGAVLCGTGLGPLVSAYMTERWGTDANGNIEEGNGAKWKWIFWHQVILGGALMVA
FG07509.1 	PMALFSGFVLFGTGLGPLVAAAFVNDL---------EDDTLAWKWSFWHQVILDGLLLIA
AN0498.1  	PMALFAAAALFGTGLAPLLTSVIVAHT--------------SWRWIYWSHAIVSGVFVLI
          	**:**:  .* ***:.*:: :                     *:* :: :.* .. ::: 

MG04897.1 	FAICFKESRGPVLLSRKAKALNKWYEEMEANGYYGAWVDDKFAQQSSFVPVGSSGSEAAT
NCU09444.1	LMVFFKESRGSVLLSRKAKALNRWYEQLEEKGFYGVWVDE--EEEE-----GVNGNADAD
FG07509.1 	IVALFKETRGSVLLSRKAKKLNEWYQKLEDAGVYGVWVTD--AQEN-----GSNSSSASS
AN0498.1  	IFFFFKETRGSVILSRKAGALNTYYEQLEAAGHVGVLMGS--------------------
          	:   ***:**.*:*****  ** :*:::*  *  *. : .                    

MG04897.1 	IADTPDQTPQDSDSYDEEKGGPSGRNSSTSPPTALRRIRWRVKSDEERTSMVKMMSVSVS
NCU09444.1	ADDEEKGPSS-------------------PGTKTLQRIRWKVKEDEERGSLTTMIGTSVY
FG07509.1 	VETLTEHDTK--VEYDER--------------SNLRRIRWLVEADEQRPPLLQMMATSVK
AN0498.1  	-------DPK------------------------PRRIRWKVKSDEQRQSLVQMISISLY
          	        ..                         :**** *: **:* .:  *:. *: 

MG04897.1 	RPFH~LLFTEPVVFWFALWVSFAWAVLYLTFGCIPLVFTTIYGWSMDASGRIFVA~MCVG
NCU09444.1	RPFH~LLFTEPVVFFFSLWAAFAWGVLYLTFGSIPLVFRRQYGWNVEQAGRIFVA~MIIG
FG07509.1 	RPFY~LLFTEPVVFSFSLWAAFSWAVLYLSFSVVPYLYSDNYNMSM----RIYVA~MMIS
AN0498.1  	RPFH1MLVTEPVVFFFSLWVSFSWAVLYLQFGSIPLVFKTNHEFNIEQTGAVFTS1MCVG
          	***: :*.****** *:**.:*:*.**** *. :* ::   :  .:. :. ::.: * :.

MG04897.1 	SIIATAVSIYQERLLYHPKWNADSG-------------------------VDPATLSR--
NCU09444.1	AILATAIGIWQEKMLHHPKWRAAATPVTSSSTEETSASDTEGPNALPYSSDEPKTPSTQK
FG07509.1 	AIVATTVAILQENLMKHPLWSGP---------------------------CHEKEVSR--
AN0498.1  	SLLITVISIYQEKIAN--------------------------------------------
          	::: *.:.* **.:                                              

MG04897.1 	---WWLFLRRRFPTNVPESRLYFTCITAALLPIGLFAFGLSSRADLHWIGPAASIAVATV
NCU09444.1	ENPIWPFLRTHFPTDSPEARLYFTCLTATFLPVGLFIFGFTARPYYHWIAPTIGIGIATM
FG07509.1 	---FWRFMRKHFPADSPEARLYFSCMTSLLLPAGLFVAFLSPASTSHYT-QAIGIGFANW
AN0498.1  	--------RFNLLPATPEARLYFVCFEAVLMPIGLFWFGWTSSPSIHWISPTIAIGCSTM
          	        * .: .  **:**** *: : ::* ***    :. .  *: . : .*. :. 

MG04897.1 	GILAVYLASFNYLADVYQQYASSAIAAQSCCRNLMGGAFPLVTGLLFRNLGPANASVLLG
NCU09444.1	GILSVYLAVFNYLADTYHRYASSALAAQSFCRNILGGVFPLVTAPLFTNLGEGRAGAILG
FG07509.1 	GIYSVYLATFNYLADTYHMYASSALAAQSFSRNVLGGIFPVLTGIMFDNLGLRTAGCVLG
AN0498.1  	GIFSVYLAVFNYLADTYHRYASSAIAAQSCCRNLLGGVFPLVTNAMFNNLGFPEASSLLG
          	** :**** ******.*: *****:**** .**::** **::*  :* ***   *. :**

MG04897.1 	CIAAALTLVPWILVAYGERIRARSRFAM~SLDKI
NCU09444.1	GVAVGLTAVPWVLVWCGSWVRGRSRFAL0QLEKS
FG07509.1 	GIASVLTLVPWVLMIFGSRIRARSKFAI0SLQKQ
AN0498.1  	AIGAALCLVPFVLAFYGQTIRAKSRMAS~ELAK-
          	 :.  *  **::*   *. :*.:*::*  .* *
```
