# Supplementary material for: Patterns of Intron Gain and Loss in Fungi
Source: PLoS Biol. 2004 Nov 30;2(12):e422. doi: 10.1371/journal.pbio.0020422 (PMC532390; doi:10.1371/journal.pbio.0020422)
Supplement: Table S1 — Also available at http://genes.mit.edu/NielsenEtAl/. (4.3 MB ZIP). [file pbio.0020422.st001.zip › NielsenEtAl/html/1052.html]

AN6267.1.NCU01674.1.MG01121.1.FG06277.1


```
 CLUSTAL W (1.82) Multiple Sequence Alignments - Introns Inserted


Sequence 1: AN6267.1	293 aa
Sequence 2: MG01121.1	292 aa
Sequence 3: FG06277.1	244 aa
Sequence 4: NCU01674.1	385 aa
Alignment Length: 386 aa
Number Identitical Residues: 95 aa
Alignment Score (without introns) 5992


MG01121.1 	------------------------------------------------------------
NCU01674.1	MPAYLTPGDNETPRAPATTNTTPSRPTPELLRARPIPNNWDTHQSASYSGRDSNLELCPS
FG06277.1 	------------------------------------------------------------
AN6267.1  	------------------------------------------------------------
          	                                                            

MG01121.1 	---------------------MAEDPRALL------------QK0ADKALASASGGFSFF
NCU01674.1	IYRLITCPSWLSIRAFSNKRFETCPSWHLLTLSRHLADAECEHQ~AEKTLASASKGWGLF
FG06277.1 	--------------------------------------------~---------------
AN6267.1  	---------------------MTADPRALL------------QK0ADKALQSASGGFSFF
          	                      :  .                 . :..:  ::: . .  

MG01121.1 	GGKEEKLQTAADYYIQAANAFKMQKSE1REAGQTFEKAAQIQNGKLNEPDDAANTYVDAF
NCU01674.1	GNKEDKYQNAADQYIQAANAFRLQKSN1TEAGKCFEEAAKIFTEKLKEPNDAANAMLDAF
FG06277.1 	----------------------MQQQN1LEAGKAFEQAAQVQTDKLKEPDDAANTLVDAF
AN6267.1  	GGRTEKYENAADLYTQAANAFRIQKLN1KEAGQAFEKAAAIQTQNLNEPGDAANTLQEAF
          	..  .. ..::.   .::.:  :*: :  ***: **:** : . :*:**.****:  :**

MG01121.1 	KAYRKSSPEDAARCVEMAIAQYCRKGNFRRAATFKENVGEMFEVEVGDLKKAMEAYEAAA
NCU01674.1	KVYRKDAPDNAVRCVEVAIKQYTMAGNFRRAASHKENQAEVYENELQNKPEAIKAYTTAA
FG06277.1 	KAYRKDDPQAAARCLNVAVDRYCAKGNFRRAASHKENLGELYEVDLGDAKSAIESYELAA
AN6267.1  	KVYRKSDPEDAVRVLSTAIQHYVLNGNLRRAASQQQYLAEVYEQELGDIKNALEAYEKAA
          	*.***. *: *.* :. *: :*   **:****: ::  .*::* :: :  .*:::*  **

MG01121.1 	GWYEGDGAAV2LSNKLWLKVADIAALDADYYKAIEAYEKVSQASINNNLMKYSVKDYFLK
NCU01674.1	EWYENDGAVA2LANKLWLKVADLSALAGDFFAAIEKFEKVAEASLGNNLMRYSVKEYFLK
FG06277.1 	TWYEGDNAAA2LANKLWLKVADVAALEGDYYKAIEKYEKVAEQSINNNLMKYSVKDYLLK
AN6267.1  	EWFDADNAEA2LANKHYLKAADLAALESDYYKAIEHYERIGRSSINNGLMKWSVKDYLLK
          	 *:: *.* . *:** :**.**::** .*:: *** :*::.. *:.*.**::***:*:**

MG01121.1 	GGLCHLATK~DMVSARRALEKYTEMDPTFPSTREYKLLADICESVEGNDREKFEDDLVAF
NCU01674.1	AGLCSLATK~DMVTAQRNITKYAEKDPSFTGQREYQLLVDLLEAASNNNLEMFQDKLAAY
FG06277.1 	AGICHLASG~DLVAAQRALEKYRDMDPSFGAQREHQLLCDLCEAIEAKSQEQFTDRLYQF
AN6267.1  	AGLCHLATQ0DMVSANRALESYRDIDPTFASTREHQLLVDLIQTIEAHDQEAFADKLFQF
          	.*:* **:  *:*:*.* : .* : **:* . **::** *: :: . :. * * * *  :

MG01121.1 	DRMTKLDKWKTTILLRIKEQIEEADN-EFA
NCU01674.1	DKMSRLDDWKAAVLLQIKNNFEEADN-EFS
FG06277.1 	DQISKLDKWKTTVLVRVKNQIEEADD-EFA
AN6267.1  	DQLSKLDKWKTTILLRVKNGIEGPEEDDFA
          	*::::**.**:::*:::*: :* .::.:*:
```
