# Supplementary material for: Patterns of Intron Gain and Loss in Fungi
Source: PLoS Biol. 2004 Nov 30;2(12):e422. doi: 10.1371/journal.pbio.0020422 (PMC532390; doi:10.1371/journal.pbio.0020422)
Supplement: Table S1 — Also available at http://genes.mit.edu/NielsenEtAl/. (4.3 MB ZIP). [file pbio.0020422.st001.zip › NielsenEtAl/html/1053.html]

AN2523.1.NCU03611.1.MG01802.1.FG10116.1


```
 CLUSTAL W (1.82) Multiple Sequence Alignments - Introns Inserted


Sequence 1: NCU03611.1	960 aa
Sequence 2: MG01802.1	840 aa
Sequence 3: FG10116.1	905 aa
Sequence 4: AN2523.1	916 aa
Alignment Length: 984 aa
Number Identitical Residues: 524 aa
Alignment Score (without introns) 23725


MG01802.1 	MAYRGAGGPGGG--RDYDGHNMQDLN-PHS~Q0-------QEDEAHRSLLTQGTT----L
NCU03611.1	MAYHGRGD-------GYDGHQLQDLPGGHN~Q0G-----DQHDDAQAPFLSENPM----P
FG10116.1 	MAYNGRDQ-------EYGGHALQDLPAGSS0Q~YHLPPQENDEEQGRGLLNSG-------
AN2523.1  	MAYHGSGPQSPGEHTYDDGHQLRDLSHSNT~S0Y-------EEEASHGLLSSQQSPFAGP
          	***.* .  . ..    .** ::**    . .         .::    :*..   . :. 

MG01802.1 	YDHDRLG---AHTPPVRPVSAYSLTESYAPNAPTTVPGSAVGASPSPFQNDYGVSSGYQG
NCU03611.1	YDNDRLG---TDTPPVRPVSAYSLTESYAPGAGTTRAGVAVNPTPPPHG-GYGGGGVSSG
FG10116.1 	YEQDRLG---ARTPPDRPVSAYSLTESYAPGASSAMPGQGP--------TGYGDTGGSFG
AN2523.1  	FDDPHQQRGLTASPVQRPTSGYSLTESYAPDAAYHDPYSAN------QSVYSGHSENPAA
          	::. :   . : :*  **.*.*********.*    .  .            *      .

MG01802.1 	AMGGHADDGFPIGGGDPQQGHPYDTEDSWVQRQNPNAAPQGGGLKRYATRKVKLVQGSVL
NCU03611.1	VDQGYNYGG-DYATDPAYRMSAIDEDDSWLRRQQPNAAPTGG-LKRYATRKVKLVQGSVL
FG10116.1 	QFG-----NLDANAPFPRPDSAFDPEDSWVERQQQPQMGGGGGLGRSKTRKIKLVQGSVL
AN2523.1  	AFG------VPGRVASPYARSETSSTEAWRQRQ-AGAAGGGNGLRRYATRKVKLVQGSVL
          	                .      .  ::* .**       *..* *  ***:********

MG01802.1 	SIDYNVPSAIRNAVQPKYR-EQEGTNEEFIKMRY~TAATCDPNDFTLKNGYDLRPRMYNR
NCU03611.1	SLDYPVPSAIRNAVQPKYR-DEEGNNEEFFKMRY~TAATCDPNDFTLKNGYDLRPRMYNR
FG10116.1 	SIDYPVPSAIKNAVQPQYR-DAESGTEEFHKMRY~TAATCDPNDFTLKNGYDLRPRMYNR
AN2523.1  	SVDYPVPSAIQNAIQAKYRNDLEGGSEEFTHMRY1TAATCDPNEFTLHNGYNLRPAMYNR
          	*:** *****:**:*.:**.: *. .*** :*** ********:***:***:*** ****

MG01802.1 	HTELLIAITYYNEDKVLLSRTLHGVMQNIRDIVNLKKSTFWNKGGPAWQKIVVCLVFDGI
NCU03611.1	HTELLIAITYYNEDKVLLSRTLHSVMTNIRDIVNLKKSSFWNRGGPAWQKIVVCLVFDGL
FG10116.1 	HTELLIAITYYNEDKVLLARTLHHTMQNIRDIVNLKKSTFWNKGGPAWQKIVVCLVFDGI
AN2523.1  	HTELLIAITYYNEDKTLTARTLHGVMQNIRDIVNLKKSEFWNKGGPAWQKIVVCLVFDGI
          	***************.* :**** .* *********** ***:****************:

MG01802.1 	EKTDKNVLDVLATIGIYQDGVVKKDVHGQETVAHI~FEYTTQLSVTPSQQLIRPQDDGPN
NCU03611.1	DKTDKNVLDVLATIGVYQDGVIKKDVDGKETVAHI~FEYTSQLSVTPNQALIRPVDDGPQ
FG10116.1 	DKADKNTLDVLATVGVYQDGVIKKDVDGKETVAHI~FEYTSQLSVTPNQQLIRPTNEGSQ
AN2523.1  	DPCDKDTLDVLATVGIYQDGVMKRDVDGKETVAHI0FEYTTQLSVTPNQQLIRPTDDGPS
          	:  **:.******:*:*****:*:**.*:****** ****:******.* **** ::*..

MG01802.1 	TLPPVQFIFCLKAKNSKKINSHRWLFNAFGRILNPEVCILLDAGTKPSSRSLLGLWEGFY
NCU03611.1	TLPPVQFIFCLKQKNTKKINSHRWLFNAFGRILNPEVCILLDAGTKPSPRSLLALWEGFY
FG10116.1 	NLPPVQMIFCLKQKNTKKINSHRWLFNAFGRILNPEVCILLDAGTKPSPRSLLALWEGFY
AN2523.1  	TLPPVQMMFCLKQKNSKKINSHRWLFNAFGRILNPEVCILLDAGTKPGPKSLLYLWEAFY
          	.*****::**** **:*******************************..:*** ***.**

MG01802.1 	NDKDLGGACGEIHAMLGKGGRKLLNPLVAVQNFEYKISNILDKPLESAFGYVSVLPGAFS
NCU03611.1	NDKDLGGACGEIHAMLGKGGKKLLNPLVAVQNFEYKISNILDKPLESAFGYVSVLPGAFS
FG10116.1 	NDKDLGGACGEIHAMLGKGGKKLFNPLVAVQNFEYKISNILDKPLESSFGYVSVLPGAFS
AN2523.1  	NDKDLGGACGEIHAMLGKGWKKLLNPLVAAQNFEYKISNILDKPLESSFGYVSVLPGAFS
          	******************* :**:*****.*****************:************

MG01802.1 	AYRFRAIMGRPLEQYFHGDHTLSKILGKKGIEGMNIFKKNMFLAEDRILCFELVAKAGQK
NCU03611.1	AYRFRAIMGRPLEQYFHGDHTLSKLLGKKGIEGMNIFKKNMFLAEDRILCFELVAKAGQK
FG10116.1 	AYRFRAIMGRPLEQYFHGDHTLSKMLGKKGIDGMNIFKKNMFLAEDRILCFELVAKAGQK
AN2523.1  	AYRFRAIMGRPLEQYFHGDHTLSKQLGKKGIEGMNIFKKNMFLAEDRILCFELVAKAGSK
          	************************ ******:**************************.*

MG01802.1 	WHLSYIKAAKGETDVPEGAAEFISQRRRWLNGSFAATLYSLMHFGRMYKSGHNIIRMFFF
NCU03611.1	WHLSYIKAAKGETDVPEGAPEFISQRRRWLNGSFAASLYSLMHFGRMYKSGHNIVRMFFF
FG10116.1 	WHLSYIKAAKGETDVPEGAAEFISQRRRWLNGSFAATLYSLMHFGRMYKSGHNIIRMFFL
AN2523.1  	WHLSYVKASKGETDVPEGAPEFISQRRRWLNGSFAAGIYSLMHFGRMYKSGHNIVRMFFL
          	*****:**:**********.**************** :****************:****:

MG01802.1 	HVQLIYNILNVIFTWFSLA~SYWLTTTVIMDLVGNPQVGQN---AREGWPFGNTVTPLFN
NCU03611.1	HVQLIYNIANVIFTWFSLA~SYWLTTTVIMDLVGTPVTASSSSAEHHGWPFGDTVTPFFN
FG10116.1 	HIQLIYTTLNTMFAWFSLG~SYWLTTSVIMDLVGKPNATSG----VHAWPFGDTGTPIVN
AN2523.1  	HLQMLYNWFSTFLTWFSLA1SYWLTTSVIMDLVGTPSSSNG----YTAFPFGKTATPIIN
          	*:*::*.  ..:::****. ******:*******.*   ..      .:***.* **:.*

MG01802.1 	AVLKYIYLAFVILQFILALGNRPKGSKYTYVTSFFVFSVIQAYILVLSGYLVVQAFQTPI
NCU03611.1	AVLKYIYLAFVILQFILALGNRPKGSKWTYITSFFVFSLIQSYILVLSGYLVARAFSVPL
FG10116.1 	ALLQYLYLAFVMLQFILALGNRPKGSKFTYIASFMVFGLIQGYILVLSAYLVVRAFDTPI
AN2523.1  	TLVKYIYLAFLLLQFILALGNRPKGSKLSYLASFVAFGIIQLYVVVDALYLVVRAFTGGA
          	::::*:****::*************** :*::**..*.:** *::* : ***.:**    

MG01802.1 	GEQIKTDT-AKDFMDSIFGKSGAAGVILLALIAIYGIYFIASFMYLDPWHMFHSFPYYML
NCU03611.1	DQQLQLDN-AKDAMASLFGGSGSAGVILVALVTIYGLYFLASFMYLDPWHMFHSFPYYML
FG10116.1 	GDQISFAS-TDAFLNSFFGGSSAGGVILVALITIYGLNFIASFMYLDPWHMFHSFPYYLV
AN2523.1  	PMDFNTDDGIGAFLSSFFGSSG-AGIIIIALAATFGLYFVASFMYLDPWHMFTSFPAYMA
          	  ::.   .    : *:** *. .*:*::** : :*: *:************ *** *: 

MG01802.1 	LMSTYINILMVYAFNNWHDVSWGTKGSDSNEALPSANITKGEKDEVVVEEIDKPQEDIDS
NCU03611.1	LMSTYINILMIYAFNNWHDVSWGTKGSDKAEALPSANVSKGEKDEAVVEEIEKPQEDIDQ
FG10116.1 	LMSTYINILMVYAFNNWHDVSWGTKGSDKAEALPSAHVTKGEKNEVVVEEVEKEQEDIDS
AN2523.1  	VQSSYINILNVYAFSNWHDVSWGTKGSDKADALPSAKTTGGKGEEAVIEEIDKPQADIDS
          	: *:***** :***.*************. :*****: : *: :*.*:**::* * ***.

MG01802.1 	QFEATVRRALAPFNDEEKPEPKDLEDS2--------------------------------
NCU03611.1	QFEATVRRALAPYKEDETPEPKDLEDS~YKSFRTMLVVSWLFSNCLLAVVITSDNFNTFG
FG10116.1 	QFEQTVRRALAPFKEEEEVEKADVEDG~YKSFRTGLVVCWLFGNILLIVCITSTNFDNLG
AN2523.1  	QFEATVKRALTPYVPPEEKEEKSLDDS~YKSFRTRLVTLWLFSNGLLAVCITSEGLDKFG
          	*** **:***:*:   *  *  .::*.  .:  :        ..       :: . .. .

MG01802.1 	--~---------------------------------------------------------
NCU03611.1	IG~VSIAVHLIVTLNHLLIYIDSKPHPRELRGSSSSFCSLLVRSLSSALLASVGSLAGPV
FG10116.1 	WG0EPATER--------------KAHYFQFLLYATAVLSLVR------FFGFLWFLGRTG
AN2523.1  	FT0NTSTER--------------TSRFFQALLWSNAVVALIR------FIGATWFLGKTG
          	    . :                ..   .    :.:  :           .     . . 

MG01802.1 	------~-------------------------
NCU03611.1	LCAALP0RKREEGREKGYCFLAAARRHGRVVP
FG10116.1 	IMCCFS~RN-----------------------
AN2523.1  	LLCCFA~RR-----------------------
          	  .. .
```
