# Supplementary material for: Patterns of Intron Gain and Loss in Fungi
Source: PLoS Biol. 2004 Nov 30;2(12):e422. doi: 10.1371/journal.pbio.0020422 (PMC532390; doi:10.1371/journal.pbio.0020422)
Supplement: Table S1 — Also available at http://genes.mit.edu/NielsenEtAl/. (4.3 MB ZIP). [file pbio.0020422.st001.zip › NielsenEtAl/html/1054.html]

AN2968.1.NCU00951.1.MG01598.1.FG00496.1


```
 CLUSTAL W (1.82) Multiple Sequence Alignments - Introns Inserted


Sequence 1: NCU00951.1	470 aa
Sequence 2: FG00496.1	290 aa
Sequence 3: MG01598.1	290 aa
Sequence 4: AN2968.1	301 aa
Alignment Length: 484 aa
Number Identitical Residues: 216 aa
Alignment Score (without introns) 9546


MG01598.1 	------------------------------~-----------------------------
NCU00951.1	MEVAPAYEPRSARTLEAALIEKKKEEAAAK0SLVRLQLLAKTRCRPLHFQPQSIRQHSTL
FG00496.1 	------------------------------~-----------------------------
AN2968.1  	------------------------------~-----------------------------
          	                                                            

MG01598.1 	------------------------------------------------------------
NCU00951.1	LPLPHPRPSSQPHIFHGYGHLPSVQNMPPQFGRPGQSQARTLASLGGPQSNSTHTPCPVG
FG00496.1 	------------------------------------------------------------
AN2968.1  	------------------------------------------------------------
          	                                                            

MG01598.1 	------------------------------------------------------------
NCU00951.1	GGAGTPPPPSAGNGSGGPAQSPNTKPLLGSSTLTRPNITTAISSARTQQIARHLSSSSVA
FG00496.1 	------------------------------------------------------------
AN2968.1  	------------------------------------------------------------
          	                                                            

MG01598.1 	-MASQFTVRKVAAPNTLEHRVYIEKDGVPVSPFHDIPLYANAEQTILNMVVEIPRWTNAK
NCU00951.1	TMASQYSVRKVGAPYTLEHRVYIEKDGVPVSPFHDIPLYANAEQTILNMVVEIPRWTNAK
FG00496.1 	-MASNYTVRKVAAPNTLEHRVYVEKDGQPVSPFHDIPLYANQEQTILNMVVEIPRWTNAK
AN2968.1  	---MSYTVRKIGQPYTLEHRVYIEKDGQPVSPFHDIPLYANAEKTVLNMIVEIPRWTNAK
          	    .::***:. * *******:**** ************* *:*:***:**********

MG01598.1 	LE0ISKDELLNPIKQDIKKGKLRYVRNCFPHKGYLWNYGAFPQ0TWEDPNAVHPETKAKG
NCU00951.1	QE0ISKEELLNPIKQDTKKGKLRFVRNCFPHKGYLWNYGAFPQ~TWEDPNSIHPETKAKG
FG00496.1 	LE0ISKEELLNPIKQDIKKGKLRYVRNCFPHKGYLWNYGAFPQ0TWEDPNTVHPETKAKG
AN2968.1  	QE0ISKEEFLNPIKQDTKKGKLRFVRNCFPHKGYLWNYGAFPQ0TWEDPNVVHPETKAKG
          	 * ***:*:******* ******:******************* ****** :********

MG01598.1 	DNDPLDVCEIGELVGYTGQVKQVKVLGVMALLDEEETDWKVIVIDVNDPLASKLNDVEDV
NCU00951.1	DNDPLDVCEIGELVGYTGQVKQVKVLGVMALLDEEETDWKVIVIDVNDPLAPKLNDVEDV
FG00496.1 	DNDPLDVCEIGELVGYPGQIKQVKVLGVMALLDEEETDWKVIVIDVNDPLASKLNDVEDV
AN2968.1  	DNDPLDVCEIGELVGYPGQVKQVKVLGVMALLDEEETDWKVIVIDVNDPLAPKLNDIEDV
          	****************.**:*******************************.****:***

MG01598.1 	ERHLPGLLRATNEWFRIYKIPDGKPENQFAFTGECKNKK2YAMDVIRECAEAWEKLITGK
NCU00951.1	ERHLPGLIRATNEWFRIYKIPDGKPENQFAFTGECKNKT2YAMDVVRECNEAWERLITGK
FG00496.1 	ERHLPGLLRATNEWFRIYKIPDGKPENQFAFTGECKNKD2YALDVVRECAEAWERLVTGK
AN2968.1  	ERHLPGLLRATNEWFRIYKIPDGKPENQFAFSGEAKNKK2YAEEVIHECADAWEKLVSGK
          	*******:***********************:**.***  ** :*::** :***:*::**

MG01598.1 	TQPGDVST~TNLSVSQSPSRVAAEQLPPLPPHEELAPAKIDASIDKWFFISGASA~----
NCU00951.1	TAPGGVST~TNVTVQHSSSRVAPDQLPPLPPNENLPPAPIDSSIDKWFFISGASA~----
FG00496.1 	TPAGGVST2TNVTVQHSPTRVSPDQLPPLPAHEEVPAEKIDASIDKWFFISGASA~----
AN2968.1  	SDRGDISL2ANSTLGNSDS-VDSSKLASIPRGENLPPAPIDGTIDKWFFISGAAV2ILHP
          	:  *.:*  :* :: :* : * ..:*..:*  *::..  **.:**********:.    .

MG01598.1 	--~--------
NCU00951.1	--~--------
FG00496.1 	--~--------
AN2968.1  	ST1CLNYSHAL
          	:: . . : :
```
