# Supplementary material for: Patterns of Intron Gain and Loss in Fungi
Source: PLoS Biol. 2004 Nov 30;2(12):e422. doi: 10.1371/journal.pbio.0020422 (PMC532390; doi:10.1371/journal.pbio.0020422)
Supplement: Table S1 — Also available at http://genes.mit.edu/NielsenEtAl/. (4.3 MB ZIP). [file pbio.0020422.st001.zip › NielsenEtAl/html/1055.html]

AN4468.1.NCU09539.1.MG09529.1.FG07438.1


```
 CLUSTAL W (1.82) Multiple Sequence Alignments - Introns Inserted


Sequence 1: NCU09539.1	119 aa
Sequence 2: MG09529.1	119 aa
Sequence 3: FG07438.1	119 aa
Sequence 4: AN4468.1	130 aa
Alignment Length: 132 aa
Number Identitical Residues: 51 aa
Alignment Score (without introns) 2718


MG09529.1 	M0VLILGVNFHEQKL~VR0KALESFWNLGPQKSARILAKYCIHPMAKLGTLPPKTITALT
NCU09539.1	M0VFILGVNFNEHKL~VQ0KALESFYGLGQQASARILAKYSIHPRAKMGTLPPKIVTALT
FG07438.1 	M0VFLLGVNFGEQKL~VK0KALESFYALGPTTSARIMAKYSIHKLAKVGSLAPRTVTSIT
AN4468.1  	M0VFILGVNFPEGQL0--~KSLQKFFGVGPQVSSRIMSRFHIHQTCKVGELANKQVLDLT
          	* *::***** * :*    *:*:.*: :*   *:**:::: **  .*:* *. : :  :*

MG09529.1 	AELSTMNIENEAKKVVLDNIRRLRDMGTYRGRRHAMHLPVRGQQTKNQ~TETARKLN-~-
NCU09539.1	AELSTMTIENDARRLVLDNIKRLRDMGTYRGRRHAMGLPVRGQQTRNQ~IANARKLN-~-
FG07438.1 	AELSQMTIETDARRLVQENIRRLKDMGSYRGRRHAMGLPVRGQRTRTQ~TASANRLN-~-
AN4468.1  	AVLSEMKIENDLRRQVLDDIKRLKETGTYRGRRHALGLPVRGQRTRNN0NKIAIRLNR1A
          	* ** *.**.: :: * ::*:**:: *:*******: ******:*:.:    * :**  :

MG09529.1 	----~-QIERRL------
NCU09539.1	----~-KIERHG------
FG07438.1 	----~-RVERRG------
AN4468.1  	WQGV0RSTECNGKMFISL
          	 ..     * . .   :
```
