# Supplementary material for: Patterns of Intron Gain and Loss in Fungi
Source: PLoS Biol. 2004 Nov 30;2(12):e422. doi: 10.1371/journal.pbio.0020422 (PMC532390; doi:10.1371/journal.pbio.0020422)
Supplement: Table S1 — Also available at http://genes.mit.edu/NielsenEtAl/. (4.3 MB ZIP). [file pbio.0020422.st001.zip › NielsenEtAl/html/1056.html]

AN4728.1.NCU00614.1.MG02867.1.FG00261.1


```
 CLUSTAL W (1.82) Multiple Sequence Alignments - Introns Inserted


Sequence 1: NCU00614.1	972 aa
Sequence 2: FG00261.1	966 aa
Sequence 3: MG02867.1	974 aa
Sequence 4: AN4728.1	889 aa
Alignment Length: 1001 aa
Number Identitical Residues: 270 aa
Alignment Score (without introns) 16822


MG02867.1 	-MRP--ILTLLLSALP-AALAVYKDEVGDIDFHHELVGLPQQDTTFFHRPRRDDRASLLY
NCU00614.1	MHLPTQLSALLLVALPSAVRAVFQDEVGHIDYHYELLGLPQRETTFFHKPRRDDKASLLY
FG00261.1 	MRLS--LQPLLLLALSSLGAAVFQDEVGQIDFHHALVGVPQVETTFFHRPRKSDKASLLY
AN4728.1  	MRLQ--ATLFLVASCVPSAFAIYADEVGHIDYHHALLGVPSSQSTFFHRPSSSSNAALLY
          	         :*: :  .   *:: ****.**:*: *:*:*. ::****:*  ...*:***

MG02867.1 	TLSDLGILGAINPSSGAALWRQQLP-----EDADGVRHLRAAEGEGWVASASGDTVQAWD
NCU00614.1	TLSDVGVLGAVNPSSGAVLWRQLLNG----TITDGGGFLRAGEGQNWLASAYGQSVHAWD
FG00261.1 	TLSDVGIIGAVNPSNGAVVWRQQIAD----DITNGGGFLRAAEGEHWVAAAYGSKVQAWD
AN4728.1  	TLSENSLLGAVNPKDGSLLWRQNLSRPAVTPDRDAQGLLRASGGKNAVVSALGDYVSAWS
          	***: .::**:**..*: :*** :  .: :   :.   ***. *:  :.:* *. * **.

MG02867.1 	AVSGRNVWSRGFEGEEVRDLEVMEMAVEGEG--HKDVLALYYKEDGGVTTLRRLRGTDGA
NCU00614.1	AVNGRNKFWMDFAG-EVKDLEVMEMTENN----RKDILALFDEN--ESTVLRRLSGNDGR
FG00261.1 	ALTGRNVWHNEFKG-EVKDLEILELTESS----RKDVLVLYDED--GTTVLRRIHGTLGQ
AN4728.1  	ALDGKLIWKSSSPGMPVVDLELLELEDASSAPLVTDAIALSGGH---SGSVRRLDGSTGE
          	*: *:  :     *  * ***::*:   ....  .* :.*   .      :**: *. * 

MG02867.1 	VVWEFREVT~KDLPLQVSTNLEKVFVVGLHGSLGS-YGLKVAVLDVPTGKRMDDISIATK
NCU00614.1	VVWEYKETS~GDVPLQVSTNVEKAFVISLRGSAGA-YNIKVTILDALSGKREDELVLGTK
FG00261.1 	VVWEFREVA~HNIPLQVSTDISKIYVVSLHGSPAS-YSLKVTALDTLTGGRLDDFAIGTK
AN4728.1  	VKWEYHDES2GDVPFQVSASSTAVFYVSLQPALLKGYKIRVTSLDPLTGRQASQQILNSD
          	* **::: :  ::*:***:.    : :.*: :   .* ::*: **  :* : .:  : :.

MG02867.1 	SDIQSSKDLILVGANSAMPIVAWTDNALTTLRVNVLGTKAKHDFPLA----AGTKSVEIH
NCU00614.1	ADVHDKDDVILVGANSAAPIIAWTDDTRQQLRVSVLGQKTRQEFALS----ADTISVEIH
FG00261.1 	GDVHGPKDVMFVGGNSAAPILAWTDSTLTKLKVNVLGSKTTQDLQLP----ADAVSVVIH
AN4728.1  	SDVSGPDSVLFVGANTAFPVIAWADKSHRTLKVNVIGTKQVTQVNIENTSGEDIRSIYIH
          	.*: . ..:::**.*:* *::**:*.:   *:*.*:* *   :. : .::. .  *: **

MG02867.1 	APHSIQSDPHFLVHMRTDSSNTAEVFHIDLKTAAIKKAYDLPLLAGKGAFSTSSDGANVY
NCU00614.1	APHIVQSQPHFLVHSKTSTGHTAEVYHVDLRTNVITKAYQLPFTSGPGAFSTSSNGANVY
FG00261.1 	APHLTQSQPHFLVHTRTKTGNKAEVYHTDLKNSKVSKAYELPHLSGPGAFSTSSDGANVY
AN4728.1  	APKALNALPHFLVQYETASGSWAEVYHVDLASSTVSKAYSLPFLQGWSVFSTGTKDANVY
          	**:  :: *****: .* :.  ***:* ** .  :.***.**   * ..***.:..****

MG02867.1 	FTRITQSEAILLASTSEKPLARWALKSGKVAEDNAINAVHAVSEVIKKS-ESSFAVRAAV
NCU00614.1	FTRITDEELVIFSSISDTVLGRWPLK----TTESRLVALHGVSEVVKKAGTDSYAVRSAA
FG00261.1 	FARVTEDETLVVSSESHAVLARWAFK----PAG-DIEAVHAVAEVIKKSGAEGFAIRAAA
AN4728.1  	FIRVTDSETTVVSSVSHGIIGRWTQH------PSLDGAVHAVSEVAMKG--DSVAVRSAI
          	* *:*:.*  :.:* *.  :.**. :       .   *:*.*:**  *.  .. *:*:* 

MG02867.1 	LTDADDWVMVQNGQVAWSKPEGLTGAVAGAWAEIPESEDLAKSLEAEAHSSPVAAYIHRV
NCU00614.1	VTDTDEWILIRNGDVVWSRPEGLTGAVAATFVEIPESENLAKTLEQEAHSNPLEAYIHRV
FG00261.1 	VTKSDDWVMVRNGEVDWKRPEGLSAAVAAVWADVPGVENLAKVLEEEAHTNPLQAYIHRV
AN4728.1  	VLESGDWQLIQNGAVGWTRPEALSGALAASWADVDSQQDLAHQLEVEGHESLLKAYTHRV
          	: .:.:* :::** * *.:**.*:.*:*. :.::   ::**: ** *.* . : ** ***

MG02867.1 	QRHINDLEHLPDYLNKLPQRFINSLLGTHVGGNGKKLERDGFGFNKIVIIATERGTVYGL
NCU00614.1	KRHLEDLQYLPTYLNNIPTRLMSSILGTEVSSHDVKLARDSFGFHKLVVLATRRGMIYVL
FG00261.1 	TRHMNDLQYLPDYLASLPERFISSISGGETVSKKEGLHRDTFGFNKLVVLVTRRGRMYGL
AN4728.1  	KRHLKDLENLPNWLKDLPKRVITSILADEVSN------LDSFGVSKPVIVATKNGWVYAL
          	 **::**: ** :* .:* *.:.*: . .. .       * **. * *::.*..* :* *

MG02867.1 	NAGDRGQTLWRHKVEDP---SNWDVQGMNVDNSQGTVTIRASNG--QTLVLKTDNGQVLD
NCU00614.1	DAGDHGKILASKRVFDLPKGQKWAIAGIKADDSTGIVTILGTNNDETIVKILPGTSFNIE
FG00261.1 	STEHKGQVVWSKSVLPQLSGETLDVKGMYAKD-EGVVTLRGAKG--EYVAIKSDTGDVVE
AN4728.1  	DSGNHGKVAWSVKAAEA---DTWNVKSIQTQP--GLATVQAADG--SSVTLNVTTGHITH
          	.: .:*:      .      ..  : .: ..   * .*: .:..    : :   ..   .

MG02867.1 	TTAAD--STLKLQSVALVDSASGPYLLSIPKDGQIGPLAADKAPKQTLVVRGGETSLRGV
NCU00614.1	VLSKGPEGSPATQSAALLNTVSGPRLFPVGSDGELQGLTSDILPNQVAVVRGSDRELKGV
FG00261.1 	VMPAG--SLPRVSSTVVVDSPAGNWLLPVGANGEVGPVPAGFTPSQTIVVRGEGETLKGL
AN4728.1  	TSAAT-------------DKPSS---------------EANTSPDVYTVTQEDGKILG--
          	. .               :. :.                :.  *.   *.:     *   

MG02867.1 	KFLTAEDSTEATEVTTWVFNAPKGEKIVEIATRPQHDPVASIGRILGDRRVLYK~YLNPN
NCU00614.1	AIADGKQTVS------WTFVLPKSQRIVDIAARPSHDAVASIGRVLGDRTVKYK~YLNPN
FG00261.1 	KFVESGNKVSAEEI--WQLQIFRGQKIVEIAKPDSHDPVASIGRVLADRRVSYK~YLNPN
AN4728.1  	-WSSKDSRIP-----VWTFQPAPGEKIIRATARPPHDPVASIGKVLGNRSVLYN2-----
          	      .         * :    .::*:  :    **.*****::*.:* * *:      

MG02867.1 	TIVVLTADPTA--STMTTYLLDTVSGETLSSSTYEGIDTTQPASCTMSENFFICTMFGDY
NCU00614.1	TLVVAAVDESTKIPSLVIYLLDTVSGQILASSKHEGVDPSKHIECAMAENWFTCTYFGQY
FG00261.1 	TIVVAAIDESS--SSLSVQLVDTISGQILASQSYAGVDSTKPISCTMAENWYACTFFGQY
AN4728.1  	--------------------------------THKGVDTAQPIASAMSENWFAYSFYAEG
          	                                .: *:*.::   .:*:**::  : :.: 

MG02867.1 	TLREDPNQSIKGHLLTVTDLYESESPDDRGPLGDAANFSSIAPLDDPTAPMPLPKVISQT
NCU00614.1	RLRDG-AQSLKGYQIVVSDLYETDKPNDRGVLGDAENFSSLGPIDAP-GDVPLPSVVSQT
FG00261.1 	TLEDGTKRSILGNQIVITDLYESSSPNDRGPLGDAETYSPLKPVDSP-AGPALPWAESQA
AN4728.1  	TDPSE----PKGYQLVISEMYESPIPNDRGPLGSASNYSSIADLPQP-------HVISQS
          	   .       *  :.::::**:  *:*** **.* .:*.:  :  *        . **:

MG02867.1 	WVLGAPISALAFSVTRQGISSRQLLAYLPHSRQIAGFHRGWLEPRRPVGRDPTPQELE-E
NCU00614.1	FILSAPISALEVTQTRQGITSRQVLAYLPENHGIVGIPRMVLEPRRPVGRDPTAAEVE-E
FG00261.1 	YVLSQPLNSLSVTQTRQGIANRQVLAYLPEAHSIAGLSRQVLDARRPVGRDSTPAEKEAE
AN4728.1  	FIIAEPISYMAVTQTRQGITTRQLLATLPASNAIIGIPRPVLDPRRPVGRDPTPTEAE-E
          	:::. *:. : .: *****:.**:** **  . * *: *  *:.*******.*. * * *

MG02867.1 	GLSRYAPAIEIDPKTVVSHELEILGVRSIVTSPAIVESTSLVLAYGVDVYVTRVAPSYVF
NCU00614.1	GLIKYHPAIEIDPKSVHTHERDVIGVQKIIAAPAVVESTSLVFAYGIDVFGSRVAPSFLF
FG00261.1 	GLIQYTPSIEIDPRSIISHQRNVLGVKNILATPVIVESTSLIVAYGVDVFGTRLAPSGMF
AN4728.1  	GLMKYAPFLDFDGRWYLSHARQVAGINTVLSAPTLLESTSLIFGFGNDIFATRATPSQAF
          	** :* * :::* :   :*  :: *:..::::*.::*****:..:* *:: :* :**  *

MG02867.1 	DILGKGFNKISLIGTVLALSAGVAALGPM0VRKKQMDMRWSAPA
NCU00614.1	DILGKGFNKVTLIGTVLAITAGVMILSPM0VRRKQINALWGAPM
FG00261.1 	DILGDGFNKSTLILTVVSLLGGVLFLSPM0VRRKQINRGWES--
AN4728.1  	DILGKGFSKLQLLLTIVALAIGVSMLAPM0ARKKKNDTLWKAR-
          	****.**.*  *: *::::  **  *.** .*:*: :  * :
```
