# Supplementary material for: Patterns of Intron Gain and Loss in Fungi
Source: PLoS Biol. 2004 Nov 30;2(12):e422. doi: 10.1371/journal.pbio.0020422 (PMC532390; doi:10.1371/journal.pbio.0020422)
Supplement: Table S1 — Also available at http://genes.mit.edu/NielsenEtAl/. (4.3 MB ZIP). [file pbio.0020422.st001.zip › NielsenEtAl/html/1057.html]

AN5753.1.NCU08137.1.MG09743.1.FG10755.1


```
 CLUSTAL W (1.82) Multiple Sequence Alignments - Introns Inserted


Sequence 1: NCU08137.1	130 aa
Sequence 2: FG10755.1	135 aa
Sequence 3: AN5753.1	120 aa
Sequence 4: MG09743.1	131 aa
Alignment Length: 143 aa
Number Identitical Residues: 51 aa
Alignment Score (without introns) 2574


MG09743.1 	-MFATKALRQAAAHAERQPLIKFIGKRNIPA1SVDHTPQPHPAAPGNILPGSGA------
NCU08137.1	MFAATRVLRQAAAHAERVPSIKFIGRRTIPT1SVDHTPKPHPASPTHSLPANWG------
FG10755.1 	MFSATRALRQAAVHTERTPLIKFLGPRTIPS1NLDHTPKPHPASGAEKLPESWSGYGNGD
AN5753.1  	-MHATTALR----NAVRTPLIRFVGKRSIPQ1SVDHTPRPHPASPTGVLPDS--------
          	 : ** .**    :: * * *:*:* *.**  .:****:****:    ** .        

MG09743.1 	-----TTFSKYREHAQQFGPLRKTIKSGDEGLVGGTSGHDLGPVQPSKSGVFFDRSELPS
NCU08137.1	---SSPSFSAYRQHAQQHGPLRKTIRPDVDGIGG-SPGAALGSVNP-PQGLYFDRNDLPA
FG10755.1 	AAASHKNFSSYRDHVQQHGPLQK------SGFGG-TSAASLGSVNA-PKGVAFDLSELPA
AN5753.1  	-------FAAYRAKAQQHGPLGR--ASFTQGSVGRTPGAALGPVQP-KQGEFFDRAELPR
          	       *: ** :.**.*** :   .  .*  * :..  **.*:.  .*  **  :** 

MG09743.1 	RFHRQPLSMAEIEAIETGGAALFA
NCU08137.1	RFRRQPLTEAEIEAIESG--CGFA
FG10755.1 	RFHRARLNAAEIEAIESGGAALFG
AN5753.1  	RFHRLPYTEAEIEAIETGGASLYA
          	**:*   . *******:*.:. :.
```
