# Supplementary material for: Patterns of Intron Gain and Loss in Fungi
Source: PLoS Biol. 2004 Nov 30;2(12):e422. doi: 10.1371/journal.pbio.0020422 (PMC532390; doi:10.1371/journal.pbio.0020422)
Supplement: Table S1 — Also available at http://genes.mit.edu/NielsenEtAl/. (4.3 MB ZIP). [file pbio.0020422.st001.zip › NielsenEtAl/html/1058.html]

AN2890.1.NCU03903.1.MG01029.1.FG09264.1


```
 CLUSTAL W (1.82) Multiple Sequence Alignments - Introns Inserted


Sequence 1: MG01029.1	503 aa
Sequence 2: FG09264.1	474 aa
Sequence 3: NCU03903.1	496 aa
Sequence 4: AN2890.1	500 aa
Alignment Length: 537 aa
Number Identitical Residues: 132 aa
Alignment Score (without introns) 8099


MG01029.1 	-----MFLS----AVSRLPLVVRVAILHMLHLSQQSKYLDLRTELSIAVMRSFVQDPKPR
NCU03903.1	----MDKTALFLQLVSKLPLMARVALLHMLQASPASKYQDLRTELTIAMLRSFMDVPKPR
FG09264.1 	-----MNLETIRIVVPLLPLILRQSFLHVLHLSDASKHLDLRSALIIACLRVILTPKTPR
AN2890.1  	MSTLNNPLALLWALLPRIPLILRTAVLHGIGQSAGSGKQDLRTEMTVAIIRSFLTGRR-V
          	 ::           :. :**: * :.** :  *  *   ***: : :* :* ::      

MG01029.1 	SISSTQKLLNKDPGVKGRIWVARYTSPAPPEVDVR---DALLAAVEGLMDECLSAMAAAG
NCU03903.1	SITFTQKMLSRVPPIRGTIWVSKYTLPIPTEEDARAIHDTLSKAIDGLQD----------
FG09264.1 	SISAVQELTLRDPGIKGRIWVSKYASPPPPETSIR---DALIVALQ--------------
AN2890.1  	PVGKQQKGAMHDPGIKGPLWVSKVRISQP-EMDVH---DAVIKAIEEL------------
          	.:   *:   : * ::* :**::   . * * . :   *::  *::              

MG01029.1 	PGGEGQQSAGKPVCRIPDLVPVEAEWTGYRASATSDSKLPSHLSERQRYDEMMKECTSPV
NCU03903.1	--RDQPKTARIQ---VPEVLPVEAEWTAHRPGVPKDAKLP-DIPEREKYDEMMKDVKAPT
FG09264.1 	--HTGDSTCRVP---VPDLVDVEAEWTGYRSGVSSGAPLP-DVSERERYHGMMRDCKRPT
AN2890.1  	--KVGEEKYDIP-----GVGPVEAEWTGYRRGVDKNAPEP-QLSEEQKYAELKKENDADM
          	      ..          :  ******.:* .. ..:  * .:.*.::*  : ::     

MG01029.1 	TVLYFHGGAHY~LMDPATHRHVTRKLAKLTGGRCYSVRYRLAPQNPFPAAVLDALVSYLT
NCU03903.1	TILYFHGGAYW~LMDPATHRPTCRELAKRTGGRCYSVRYRLAPQNPFPAAVMDALVSYLG
FG09264.1 	TVLYLHGGAYY~LCDPATHRTTTKKLAQLTGGRCYSVRYRLAPQHPFPAALLDAFVSYFT
AN2890.1  	VVLYFHGGAYY2LMDPCTHRLAVSQLSKRTKSPVLSVRYRLAPQNPFPAALVDALTAYLY
          	.:**:****:: * **.*** .  :*:: * .   *********:*****::**:.:*: 

MG01029.1 	LLYPPPGAFHKAVKPEHIVFAGDS2AGGNLCLALLQTILELNRQSREITWHGSTVSVPTP
NCU03903.1	LLYPPPEAFHEPVKPEHIVIAGDS~AGGNLSLALLQLIMQLQRSGTTVFWLGHERSIPLP
FG09264.1 	LLYPPPDAYHDPVQPEHIVIAGDS2AGGNLSLALLQLILELRRQDSPILWYGELRQVPLP
AN2890.1  	LIAPPPGSFHAPVPPNKIILAGDS~AGGNLSLVLLQTLLTLHRKSTTVTFHNTSVPITPP
          	*: *** ::* .* *::*::**** *****.*.*** :: *.*..  : : .    :. *

MG01029.1 	AGCAVNSPWMDMTLSSPSWKKNS--KWDYLP--SDDGLDD---------RRPSCAAWPAT
NCU03903.1	AGVAVNSPWIEITHSSPSCVTNG--AFDYLPGLEAQDKAEK--------LRAPCSAWPTN
FG09264.1 	AGLALNSPWLDVTQSSPTWEASTPTPFDYLP--KPENVDQL--------AIPPCKAWPAN
AN2890.1  	AGVAVSSPWCDISRSMPSIRKNA--PYDYLPAPSPFSSSEANGEPFRPPPVPADAIWPTN
          	** *:.*** ::: * *:   .    :****. .  .  : ....  ..  ..   **:.

MG01029.1 	PPRRSIYVDDAMILHPLASPLAAAN--WAGSPPIYVCTGWELLADEDRLLAARLEQAGVP
NCU03903.1	PPRMSMYVADDYIMHPLVSLLLAPS--WRGAPPTYICTGWEMLSDEDKFTAARFHAEGVS
FG09264.1 	PPRRNLYVADELAAHPLASLVMARS--WKGAPPIYLCTGWEILAYEDKYLARQLEADGVR
AN2890.1  	PPRVDYFVSASAILHPLVSPLAAPSDLWNNCPPVYISIGEEGLTDEGLVMARRMHKASVS
          	*** . :*      ***.* : * .. * ..** *:. * * *: *.   * ::.  .* 

MG01029.1 	VVFEEYEAMPHCFALVFNHLEASRRCFDAWAGFIGKVID---GAEGDSGVEGKGNSVESS
NCU03903.1	VTFEEYEGMPHCFAMVLKNLKEADRCMEGWSRFIARVTNSTASEGGLDGADDKRSAMRST
FG09264.1 	VVFEEYEGMPHCFAMMLRNAPATPRCYNGWASFISAAVE---NPGG----------IESS
AN2890.1  	VIAEQVEGMPHCFGLMMPGHRAAKAFYDSMGSFCVDAVAETLKERVDG---------KLR
          	*  *: *.*****.:::     :    :. . *   .  .:      .         .  

MG01029.1 	FTTVRAKTLEEVPIPTGELRKWSEEEVRERLVRRCQQLLP---AIKGDDSGTLA-TPKL
NCU03903.1	FTTIKSGTLEEKELDPMTLSPYTEEEIREKMRRRIEL--------KCVPASEGD-LAKL
FG09264.1 	AVMIKSKTCEEAPLRFDQLSDASEEEFRQRVLHKTG---------LADGDIEIP-MAKL
AN2890.1  	FLAFKPENNKEIPLS-EVASHLPDEEVDRLLAETKQWRVIGEKVLVDEWSAKIENRARL
          	   .:. . :*  :        .:**. . : .       ...           . .:*
```
