# Supplementary material for: Patterns of Intron Gain and Loss in Fungi
Source: PLoS Biol. 2004 Nov 30;2(12):e422. doi: 10.1371/journal.pbio.0020422 (PMC532390; doi:10.1371/journal.pbio.0020422)
Supplement: Table S1 — Also available at http://genes.mit.edu/NielsenEtAl/. (4.3 MB ZIP). [file pbio.0020422.st001.zip › NielsenEtAl/html/1059.html]

AN6040.1.NCU06966.1.MG01909.1.FG05666.1


```
 CLUSTAL W (1.82) Multiple Sequence Alignments - Introns Inserted


Sequence 1: AN6040.1	141 aa
Sequence 2: FG05666.1	156 aa
Sequence 3: NCU06966.1	188 aa
Sequence 4: MG01909.1	157 aa
Alignment Length: 198 aa
Number Identitical Residues: 59 aa
Alignment Score (without introns) 3073


MG01909.1 	--~--------MASIATLERIPAAKLRDLLLAEKAD-----------DPKVAVVDVRDDD
NCU06966.1	--~--------MTTIGSLQRLSATSLSKLILAEQASTAAAAAGTENSRSTLAIIDVRDDD
FG05666.1 	MI0SFFTQTRTMTTIASLKRLSAKSLSEKILQEVDA----------TDPTFAVIDVRDND
AN6040.1  	--~------MSSITIANLPRISRDALSALILSAS------------TPSKLAIIDVRDSD
          	          :  :*..* *:.   *   :*               : ...*::****.*

MG01909.1 	1YIGGHIKGALNVPSQQLEARMPTLIRQLQDKPTVIFHCALSQQRGPGAALRYIRERDEA
NCU06966.1	1YIGGHIKGSQNVPSHKLDAMLPTLVRQLQDKETVVFHCALSQQRGPSAALRYIRERDRL
FG05666.1 	1YIGGHIKGSTNVPAHTLDSMMPTLVRRLKDKKTVVFHCALSQQRGPSAALKYLRERDGL
AN6040.1  	1HVGGHIVSSTWVPSSTLDVRIPELVRTLKDKEKVVFHCALSQQRGPSAALKYARERERM
          	 ::**** .:  **:  *:  :* *:* *:** .*:***********.***:* ***:  

MG01909.1 	LKKANDKDAAAS--SAPGAS------------------QPVEQKVYVLDRGFVGWQEVYG
NCU06966.1	MPKEVETKLAASVEEAVGADGEGEVKKKQGEEEGEEKKKPVDQKVFVLDRGFVGWQEAFG
FG05666.1 	LCSMGEDPKGES-----------------------------GQDVFILDQGFSGWQEVYG
AN6040.1  	LGSE-ESHK---------------------------------QEVFVLEGGFVQWQEMYG
          	: .  :                                    *.*::*: **  *** :*

MG01909.1 	EDENLTEGYRKELWKDGYWC
NCU06966.1	LDERLTEGYSKELWRDGYWM
FG05666.1 	EDERLTEGYVKDLWEN-Y--
AN6040.1  	KDVRLTEAYVEDIWRE-Y--
          	 * .***.* :::*.: *
```
