# Supplementary material for: Patterns of Intron Gain and Loss in Fungi
Source: PLoS Biol. 2004 Nov 30;2(12):e422. doi: 10.1371/journal.pbio.0020422 (PMC532390; doi:10.1371/journal.pbio.0020422)
Supplement: Table S1 — Also available at http://genes.mit.edu/NielsenEtAl/. (4.3 MB ZIP). [file pbio.0020422.st001.zip › NielsenEtAl/html/106.html]

AN8696.1.NCU02014.1.MG03665.1.FG08527.1


```
 CLUSTAL W (1.82) Multiple Sequence Alignments - Introns Inserted


Sequence 1: NCU02014.1	426 aa
Sequence 2: FG08527.1	311 aa
Sequence 3: MG03665.1	311 aa
Sequence 4: AN8696.1	319 aa
Alignment Length: 430 aa
Number Identitical Residues: 95 aa
Alignment Score (without introns) 4504


MG03665.1 	MTITLNIPIPFLEKKAKIIAQLATPDSTYTDASPKGSVDVGIRELIAEINC-QAGLVTTS
NCU02014.1	--MLPPPTPSFLTRKSKILSQLSVPDVEYTDASPKGSVDVAIRELIDEINHGYEGLVTTS
FG08527.1 	MQEVASCPPAFVERKKKILDQLAIPDTEYTDASPKGSVDEGIRDLIDEINQ-QSGFVTTS
AN8696.1  	-----MIPPVFVSRKNKILAELSAPEEEYSDLSPKGSVDEGIRDLIEDINT-LPGLVTTS
          	       .  *: :* **: :*: *:  *:* ******* .**:** :**    *:****

MG03665.1 	SCAGRVSVFLEGKK------AQNRVQVGGGDAQ--------------------LAGTVGG
NCU02014.1	SCAGRVSVYLEGVKRKKDNKASGAGDVEGEGEGDGEGGEDGVRASTATSTAAAVAASSSG
FG08527.1 	SCAGRVSVFLEGRR---------VAEAEGEDER---------------------VAGVGG
AN8696.1  	SCAGRISVFLEGRK-----APSLSEDSPSAGDG--------------------RKFVPSG
          	*****:**:*** :      ..   :  . .                           .*

MG03665.1 	KGGGGRWLYVSHEPFG--DVGGR-----SWEEVLFGADGPGDDVAD------EGTGEAEE
NCU02014.1	GKGGGEWLFVSHDPLETVDAKTGREYDGDHWMEVFGLTGNGSEPGSGDDGDGDSQQEQQQ
FG08527.1 	KGAGGAWLFVSHDPIP--DKGDG----VTDWSSQFGLEDSTAAQN-------AAPTVKER
AN8696.1  	GKGAGRWLYVSHDPLEIKEN--------QSFLKLFGMVPGDGKPPG------ADKGGHAL
          	  ..* **:***:*:   :               **         .              

MG03665.1 	RLVHFKFEPM0ILHILTASSEHAQSVIRCGLEAGFRESGAINLLG---------------
NCU02014.1	RLIHFKFEPM0ILHILTTSPYHAHLAIQSGMTSGFRETGAVSILPRLTAPFFSSCHQCRT
FG08527.1 	RLVHFKFEAM0ILHVLTASPEHAQILLRCGLQAGFRESGALNIVP---------------
AN8696.1  	RLVRFHFEPM0ILHIMTATLHHAQPVLSAASSSGFRESGLQGLRC---------------
          	**::*:**.* ***::*::  **:  : ..  :****:*  .:                 

MG03665.1 	---------------PQQQQQAT-----------PMVAIRSMGLGLESIIGTLAAGG--L
NCU02014.1	HHNHLQQQPSSSSSSSSPSSSTTTPTPTESVTPNPIVAIRSMGLSFESLIGVQRGSQ--R
FG08527.1 	----------------SGKDATT-----------PMVAIRTMGLAFESLIGQQVDGQ--R
AN8696.1  	--------------LVEGDKGPS-----------PIVAVRSAGLALESVIGYYEDDSDVI
          	                . .. .:           *:**:*: **.:**:**    . .  

MG03665.1 	QCLVTPQYLAMLVRISNERFRQNAERIERFRLALQEEFGEKKQTGN--------------
NCU02014.1	QSLVSPEYLSLLVKIANERFEENKKRIARFQEALRLAFGEGATTAGGVEGQKKKKKSGDG
FG08527.1 	QRIVSPEYLQTLVDIANERFDENKKRIERFQNAFREAVSAPAPRRN-PEGQ---------
AN8696.1  	RSLVSEEYLQMLVTMSNERFSVNTERKKRFRIALLNNCSSENPNGANHKGKT--------
          	: :*: :**  ** ::****  * :*  **: *:    .       . ....        

MG03665.1 	----PGWEDAAARRERKKAEGLRRREEIAKEKEKSNSA----------NGNPDMSDEQPI
NCU02014.1	ESGNAEWEDAEARKQRKREEGLARREEVRRRKEEEEEEKRRKQEDIKEEILEENAGKVDL
FG08527.1 	-----EWEDAAARRERKRAEGLRKRAELKAKQEANTND---------ENELSDREGEKQV
AN8696.1  	---KPGWEDPQKRRERMRAEGLARKKLLERQDGATQAK----------DQDTLEADEAGI
          	   .. ***.  *::* : *** ::  :  ..                :      .:  :

MG03665.1 	SIP--------
NCU02014.1	AEVVLQVPDVL
FG08527.1 	GLLF-------
AN8696.1  	WGSLDT-----
          	
```
