# Supplementary material for: Patterns of Intron Gain and Loss in Fungi
Source: PLoS Biol. 2004 Nov 30;2(12):e422. doi: 10.1371/journal.pbio.0020422 (PMC532390; doi:10.1371/journal.pbio.0020422)
Supplement: Table S1 — Also available at http://genes.mit.edu/NielsenEtAl/. (4.3 MB ZIP). [file pbio.0020422.st001.zip › NielsenEtAl/html/1060.html]

AN6945.1.NCU02328.1.MG07992.1.FG10331.1


```
 CLUSTAL W (1.82) Multiple Sequence Alignments - Introns Inserted


Sequence 1: NCU02328.1	461 aa
Sequence 2: FG10331.1	418 aa
Sequence 3: MG07992.1	438 aa
Sequence 4: AN6945.1	434 aa
Alignment Length: 497 aa
Number Identitical Residues: 114 aa
Alignment Score (without introns) 6795


MG07992.1 	MLAAEKISTANDGTATGGTNASTAAGPTDGKHAVDAAIATQPADLASVPSLARAIAAGAD
NCU02328.1	-----------------------------MSTAVDLSLATRPSDADAVPGLIEDLTSLNG
FG10331.1 	--------------------MGSLGASKSTPDGIAVMDVEKPCNLEAVPDLIKEVVSVAD
AN6945.1  	-------MPHTENTTNACANGHGNGYNENGITVNVEELAVSPNLPDQVPGLLDKIAAFSK
          	        . ...::.. :.    .   .         .  *     **.*   :.:   

MG07992.1 	AVTGEDDAPRYELLLKARSLVQALETPRETMIKHCWAQ~TSATGALTFGVDAGLWELMAR
NCU02328.1	NWTEDDEETRHKMVIKARSLLQSLMTPREQMLQHTWAD0PGLDAALTTGVDVGLWKLMVK
FG10331.1 	ELASGSTDARQELLIKLRTLSLAIETPREIAMRHCWTM0TTSIGALAFGVDSGLWRAMVQ
AN6945.1  	QYLGAEPQARLKLLETARSLVYALETPREAIIRHCWAE0STSYAALETAVALNLFTALGT
          	     .  .* ::: . *:*  :: ****  ::* *:  .   .**  .*  .*:  :  

MG07992.1 	NG-DGPQAVADLAAVLGVDPPLL-----A~RVMRHVAAMGYLEEVGVDRYRPTAFVKALT
NCU02328.1	NGVDAVHKVDHLARSLGMDPELL-----G1RLLRHVCAMGHLVEVAQDEYKLTSFTKSMS
FG10331.1 	NG-DRGQSVSELADQTGVDPVLL-----A1RIMRHLAAMGYVDETAQDEYRPTVFSKSLS
AN6945.1  	HE---SKTVAELAEATGAEPALLSQLETG1RLMKHLAAMGVITETGCDEYRPTSFSKVLT
          	:     : * .**   * :* **:. .:. *:::*:.*** : *.. *.*: * * * ::

MG07992.1 	IPMIGAGYLVA~PSASGAAQLRFHDYARKHGFRNPDDPADTSCNNAYKTKS-NYFEYQAE
NCU02328.1	LDVIGDSYVCL2LGGIGRSPIDFYKFLRETNWQNPVDAAHTAFHVSYNSDVPNCMVYLGS
FG10331.1 	IPTVGNGLIGL2TCATGASPLKFHEFSRKSGWKNPTDTKNTPLMYAYNTDA-DMFSWIQS
AN6945.1  	VEKYSDAFPLM2TSRFTMGILALPAFLEKTKYRNPTSATDTAFQLGYNTDKGFFGLLQQE
          	:   . .           . : :  : .:  ::** .. .*.   .*::.         .

MG07992.1 	LGY1SMHFNHHM~AGYRQGRLPWMHPSFYPVQDRLVAGFDPS--TALLVDIGGSLGHDML
NCU02328.1	IGM~GPQMNHHM1GGYRQGRLPWHHPKIYPVEKELFPGTDASSDAPLVVDVAGGLGHDID
FG10331.1 	QGY~GSYFNDHM~MGYHP--TPWMATGRFPIQEQLIDGAHKSHDAPFWVDIGGCLGQDLL
AN6945.1  	PIT~AKRFNNHM~GVYAQGRARWMDPGFYPVRERLIDGVAINQEDVLLVDVGGSFGHDLL
          	    .  :*.**   *  .   *  .  :*:...*. *   . .  : **:.* :*:*: 

MG07992.1 	EFHRHHPMAPG~KLILQDLPAVISEIQPGDLPAAATAMSYDFTTEQPVRG~ARAYYLHSV
NCU02328.1	EFKRNYPNHPG0KLILQDRPTVIEDIK-DIDPT-IQRMPHDFLTEQPIKG~ARAYFMHSI
FG10331.1 	DLRRHYPSIPG~KLILQDLPPVIEQVK-KIQQTSFTAMEHDFFTEQPVKG1SRAYYLHSV
AN6945.1  	DFRRKWPDIPG~RLVLQDLPEVISAVK-DLHPS-IDITAHDFFTEQPVKA~ARAYYLHSV
          	:::*: *  ** :*:*** * **. ::     :      :** ****::. :***::**:

MG07992.1 	LHDWPDEAAGRILQRVRAAMTPGYSRLLVNENVVPDRGAWWETTALDFMMMTLFSAKERT
NCU02328.1	LHDWPDDVCQKILARLAEAMKPGYSKLLIFECVIPRTGAYWEATAGDMLMMTQLSACERT
FG10331.1 	LHDWPDSVCEKILGHITDAMERGYSKLLIHEHVVPLTNASWETTAKDILMMAMFSAGERS
AN6945.1  	LHDWPDDLCSKILANLAAAMKPRYSKLLVNENVIPDKGAYWETTSLDLIMMQLGSG-ERT
          	******. . :** .:  **   **:**: * *:*  .* **:*: *::**   *. **:

MG07992.1 	EADWRALLEGN--GFRIVGIWSGGKG-VESLIECELA~----------------------
NCU02328.1	EDQWHQLIEGSGLGLKIVKFWSSGLSAVENVIECELA2VDREATTSTCIMAVWEIGGQFV
FG10331.1 	EVQWRDLLEAK-AGLRITSIWQLDLP-DEYLIECELP~----------------------
AN6945.1  	ERHWRSLLESA--GLRIVGFWTAHRS-VESLIECELA~----------------------
          	* .*: *:*.   *::*. :*       * :*****.                       

MG07992.1 	---~----------------------
NCU02328.1	HTV1CRSRMLRRARRHGSGIASVGDE
FG10331.1 	---~----------------------
AN6945.1  	---~----------------------
          	
```
