# Supplementary material for: Patterns of Intron Gain and Loss in Fungi
Source: PLoS Biol. 2004 Nov 30;2(12):e422. doi: 10.1371/journal.pbio.0020422 (PMC532390; doi:10.1371/journal.pbio.0020422)
Supplement: Table S1 — Also available at http://genes.mit.edu/NielsenEtAl/. (4.3 MB ZIP). [file pbio.0020422.st001.zip › NielsenEtAl/html/1061.html]

AN2149.1.NCU04448.1.MG10358.1.FG05250.1


```
 CLUSTAL W (1.82) Multiple Sequence Alignments - Introns Inserted


Sequence 1: MG10358.1	566 aa
Sequence 2: FG05250.1	565 aa
Sequence 3: NCU04448.1	558 aa
Sequence 4: AN2149.1	577 aa
Alignment Length: 578 aa
Number Identitical Residues: 427 aa
Alignment Score (without introns) 17408


MG10358.1 	MATMFEQPRNGT----------LF~LGGQKISGADIRDQNV1LATQAIANVVKSSFGPSG
NCU04448.1	MASMFAQSGSGT----------LF~LGGQKIS--------V1IATQAIANVVKSSFGPSG
FG05250.1 	MASMFEQPRNGT----------LF~LGGQKISGSDIRDQNV1LATQAIANVVKSSFGPSG
AN2149.1  	MAGIFEAPRNADTLCESHPPAWQI1LGGQKITGADVRDQCV1LATQAIANVVKSSFGPSG
          	** :*  . .. : ..: ..:  : ******:.:.  .. * :*****************

MG10358.1 	LDKMMVDDIG0DVTVTNDGATILSLLDVEHPAGKILVDLAQQQDKE~VGDGTTSVVLIAA
NCU04448.1	LDKMMVDDIG0DVTVTNDGATILSLLDVEHPAGKILVDLAHQQDKE~VGDGTTSVVLIAA
FG05250.1 	LDKMMVDDIG0DVTVTNDGATILSLLDVEHPAGKILVDLAQQQDKE~VGDGTTSVVLIAA
AN2149.1  	LDKMMVDEIG0DVTVTNDGATILSLLDIEHPAGKILVDLAQQQDRE0VGDGTTSVVLIAA
          	*******:** ****************:************:***:* *************

MG10358.1 	ELLRRGNELMRNRIHPTTIITGYRLALREAVKYMNENVSIKVENLGRESLINIAKTSMSS
NCU04448.1	ELLKRGNDLMKNRIHPTTIITGYRLALREAVKYMKEHISIKVENLGRESLLSIAKTSMSS
FG05250.1 	ELLRRGNELMRNRIHPTTIITGYRLALREAIKYLNENVSIKVEDLGRESLINIAKTSMSS
AN2149.1  	ELLRRGNELMKNRIHPTTIINGYRLALREAVKYMNENIATKVDHLGKDSLVNIAKTSMSS
          	***:***:**:*********.*********:**::*::: **:.**::**:.********

MG10358.1 	KIIGSDSDFFANMVVDAMQAVKTTNTRNETKYPVKAVNILKAHGKGSLESVLVKGYALNC
NCU04448.1	KIIGADSDFFANMVVDAIQAVKTTNNKNETKYPVKAVNILKAHGKGVTESMLIKGYALNC
FG05250.1 	KIIGADSEFFSNMVVDAMQAVKSTNNRNETKYPVKAVNILKAHGKSTLESVLVKGYALNC
AN2149.1  	KIIGADADFFANLCVDAMLLVKTTNQKNEVKYPVKAVNLLKAHGKSGTESVLVNGYALNC
          	****:*::**:*: ***:  **:** :**.********:******.  **:*::******

MG10358.1 	TVASQAMKTHIKDAKIAVLDINFQKERMKLGVQITVDDPQQLEQIRAREAGMVIERIEMI
NCU04448.1	TVASQAMTTRVTDAKIACLDINLQKERMKLGVQITVDDPQQLEAIRARESGMIIERVEMI
FG05250.1 	TVASQAMPTRIQDAKIAVLDMNLQKERMKLGVQITVDDPQQLEQIRAREAGMVLDRVEMI
AN2149.1  	TVASQAMKTRITDAKIACLDMNLQKERMKLGVQITVDDPDQLEKIRERESGIVIERVEKI
          	******* *:: ***** **:*:****************:*** ** **:*::::*:* *

MG10358.1 	LKAGANVVLTTKGIDDLCLKYFVEKGAMAVRRCKKEDLRRIARATGATLLGTLSDLNGDE
NCU04448.1	LKAGANVILTTKGIDDMVLKLFVEKGAMAVRRCKKEDLRRIARATGATLLSTLSDLNGDE
FG05250.1 	LKAGANVILTTKGIDDLVLKTFVEKGAMGVRRCKKEDLRRIARATGATMLSTLSDLNGDE
AN2149.1  	LKSGANVILTTKGIDDMVLKLFVEKGAMAVRRCKKEDLRRIAKATGATLVSTLSDLNGDE
          	**:****:********: ** *******.*************:*****::.*********

MG10358.1 	RFDPSYLGYAEEVAQERISDDECILIKGTKAHSSASIILRGPNDFQLDEMERSVHDSLCA
NCU04448.1	KFEPSYLGHAEEVVQERISDDECILIKGTKVHSSASIILRGPNDFTLDEMERSVHDSLCA
FG05250.1 	KFDPSYLGYAEEVVQERISDDECILVKGTKAHSSASCILRGPNDFTLDEMERSVHDSLCA
AN2149.1  	KFEASYLGHADEVVQERISDDECILVKGTKVHTSASIILRGPNDFSLDEMERSVHDSLCA
          	:*:.****:*:**.***********:****.*:*** ******** **************

MG10358.1 	VKRTLESGSIVPGGGAVETALHIYLEEFAGTV~GSREQLAIGEFAQSLLVIPKTLAVNAA
NCU04448.1	VKRTLESGSIVPGGGAVETALHIYLEEYAGTV~GSREQLAIGEFAQSLLVIPKTLAVNAA
FG05250.1 	VKRTLESGSIVPGGGAVETALHIYLEEFAGTV~GSREQLAIGEFAQSLLVIPKTLAVNAA
AN2149.1  	VKRTLESGSIVPGGGAVETALHMYLEEFAVTV0GSREQLAIGEFAQSLLIVPKTLAVNAA
          	**********************:****:* ** ****************::*********

MG10358.1 	KDASDLVAQLRARHALSQRTQDGEGNEDEKTVARKKGYRNYGLDLARGKLVDQIKVGVLE
NCU04448.1	KDASELVAQLRSRHALSQRIQEGEANEDEKIVARKKAYKNYGLDLMKGKVVDEIKAGVME
FG05250.1 	KDAAELVAQLRSRHALSQRIQEGDGSEDEKTIARKKGYKNYGLDLAKGKVVDEIKIGVLE
AN2149.1  	KDSSELVAQLRKRHALSQRVQEGEANEKEKAVAKKKEYRNYGLDLTKGRVHDCLKAGVLE
          	**:::****** ******* *:*:..*.** :*:** *:****** :*:: * :* **:*

MG10358.1 	PSVSKVRQLKSAVEACISIMRIDTLIKLDPEQQAEDDGHGH--
NCU04448.1	PSMSKITQLKSAVEACISIMRIDTLIKLDPEQRAEDDGHDH--
FG05250.1 	PSMSKVRQLKSAVEACISIMRIDTLIKLDPEQ-REDDGHGH--
AN2149.1  	PSMGKLKQLKSAVEACIAIMRIDTMIKLDPER-KEDDGHGHDH
          	**:.*: **********:******:******:  *****.*.
```
