# Supplementary material for: Patterns of Intron Gain and Loss in Fungi
Source: PLoS Biol. 2004 Nov 30;2(12):e422. doi: 10.1371/journal.pbio.0020422 (PMC532390; doi:10.1371/journal.pbio.0020422)
Supplement: Table S1 — Also available at http://genes.mit.edu/NielsenEtAl/. (4.3 MB ZIP). [file pbio.0020422.st001.zip › NielsenEtAl/html/1062.html]

AN0595.1.NCU09741.1.MG07117.1.FG09786.1


```
 CLUSTAL W (1.82) Multiple Sequence Alignments - Introns Inserted


Sequence 1: NCU09741.1	692 aa
Sequence 2: MG07117.1	690 aa
Sequence 3: FG09786.1	689 aa
Sequence 4: AN0595.1	695 aa
Alignment Length: 696 aa
Number Identitical Residues: 407 aa
Alignment Score (without introns) 19896


MG07117.1 	MAELDTLDIIVLGLILCGTIAYFTKGKYWGVVKDPYAAS-FANTNGPKTGKTRNIVEKME
NCU09741.1	MAELDTLDLVVLAAILLGTVAYFTKGKLWGVTKDPYASS-LANASGAKAGKTRNIVEKME
FG09786.1 	MAELDTLDVIVLGVIFLGTVAYFTKGKLWGVTKDPYANG-FAAGGAAKPGRTRNIVEAME
AN0595.1  	MAQLDTLDVVVLAVLLAGSIAYFTKGTFWAVAKDPYASSGPAMNGVAKAGKSRNIIEKMD
          	**:*****::**. :: *::******. *.*.***** .. *  . .*.*::***:* *:

MG07117.1 	ETGKNCVIFYGSQTGTAEDYASRLAKEGKSRFGLETMVADLEDYDYENLDTVPSDKIVMF
NCU09741.1	EQGKNCIIFYGSQTGTAEDYASRLAKEGKSRFGLETMVADLEEYDFDNLDTIPDDKVVFF
FG09786.1 	ESGKNCVIFYGSQTGTAEDYASRLAKEGKSRFGLNTMIADIEDYDFDSLDTVPNDNVVMF
AN0595.1  	ETGKNCVIFYGSQTGTAEDYASRLAKEGSQRFGLKTMVADIEEYDYENLDQFPEDKVAFF
          	* ****:*********************..****:**:**:*:**::.** .*.*::.:*

MG07117.1 	VLATYGEGEPTDNAVDFYEFITGEDVSFSEGS-----TLDNLNYVAFGLGNNTYEHYNSM
NCU09741.1	VLATYGEGEPTDNAVDFYEFITNEDVNFTEGNEP---ALGNLNYVTFGLGNNTYEHYNSM
FG09786.1 	VLATYGEGEPTDNAVDFYEFITGEDATFNEGNDP---PLGNLNYVAFGLGNNTYEHYNAM
AN0595.1  	VLATYGEGEPTDNAVEFYQFITGDDVSFEGGGSAEDKPLSSLKYVAFGLGNNTYEHYNAM
          	***************:**:***.:*..*  *.......*..*:**:************:*

MG07117.1 	VRNVNKALEKLGAHRIGDAGEGDDGAGTMEEDFLAWKDPMWAALAEKMGLEEREAVYEPV
NCU09741.1	VRNVDKALQKLGAHRIGDAGEGDDGAGTMEEDFLAWKEPMWKALAERMGLEEREAVYEPV
FG09786.1 	VRKVDQALEKFGAHRIGEAGEGDDGAGTMEEDFLAWKDPMWESLAKKMGLEEREAVYEPI
AN0595.1  	VRQVDAALTKLGAQRIGSAGEGDDGAGTMEEDFLAWKEPMWAALSEAMNLQEREASYEPV
          	**:*: ** *:**:***.*******************:*** :*:: *.*:**** ***:

MG07117.1 	FSVTEREGLTVESPEVYLGEPNKMHLEGTAKGPFNAHNPYIAPIVKSYELFNVKDRNCLH
NCU09741.1	FSIIEREGLTKESPEVYLGEPNKMHLDGTAKGPFNAHNPYIAPIAQSYELFSVKDRNCLH
FG09786.1 	FAINERDDLSPESNEVYLGEPNKLHLEGTAKGPFNSHNPYIAPIAESYELFSAKDRNCLH
AN0595.1  	FCVTEDESLTPEDNSVYLGEPTKGHLEGQPNGPYSAHNPYIAPIVESRELFTVKDRNCLH
          	*.: * :.*: *. .******.* **:* .:**:.:********.:* ***..*******

MG07117.1 	IDVDVSGSNLTYQTGDHIAVWPTNPGEEVDCLLDVLGLTDKRDTVVSVRPLEPTAKVPFP
NCU09741.1	MDIDISASNLNYQTGDHIAVWPTNPGEEVDRFMNVLGLSEKRHTVVSVKALEPTAKVPFP
FG09786.1 	MEVDISGSNLKYETGDHIAIWPTNPGEEVNRFLDILDLSGKQHSVITVKALEPTAKVPFP
AN0595.1  	MEISIAGTNLTYQTGDHIAIWPTNAGAEVDRFLNVFGLEEKRHSVINIKGIDVTAKVPIP
          	:::.::.:**.*:******:****.* **: :::::.*  *:.:*:.:: :: *****:*

MG07117.1 	APTTYDAILRYHMEICAPVSRQFIATLAAFAPDEETKAEMTKLGGDKDYFSAKISKHYLN
NCU09741.1	TPTTYDAIVRYHMEICAPVSRQFLAQLAAFAPDAEARAEMTKLGAEKDYFYEKISLHHLN
FG09786.1 	NPTTYDAILRYHLEICAPVSRQFVSTLAAFAPNDSIKAEMNRLGSDKDYFHEKTGPHYYN
AN0595.1  	TPTTYDAAVRYYMEVCAPVSRQFVSTLAAFAPDEETKTEIVRLGSDKDYFHEKITNQCFN
          	 ****** :**::*:********:: ******: . ::*: :**.:****  *   :  *

MG07117.1 	IARVLFNVGKGKKWNNIPFSAFIEGLTKLQPRYYSISSSSLVQPKVITITAVVEKQEIPG
NCU09741.1	IARVLDIVGKGQKWTNIPFSAFIEGITKLQPRYYSISSSSLEQPKTISITAVVENTQLPG
FG09786.1 	IARFLSSVSKGEKWTTIPFSAFIEGLTKLQPRYYSISSSSLVQPKKISITAVVESQQIPG
AN0595.1  	IAQALQSITS-KPFSNVPFSLLIEGLNKIQPRYYSISSSSLVQKDKISITAVVESTRLPG
          	**: *  : . : :..:*** :***:.*:************ * . *:******. .:**

MG07117.1 	RDDPFRGVTTNYLLALKQKQNGEPHPEPFGRTFALAGPRDKYDGIKVPVHVRHSNFKLPS
NCU09741.1	RDDPFRGVATNYLLALKQKQNGEPEPCPFGLSYELTGPRNKYDGIHVPVHVRHSNFKLPS
FG09786.1 	RDDPFRGVATNYLFALKQKQNGDPSPAPFGQTYELTGPRNKYDGIHVPVHVRHSNFKLPS
AN0595.1  	ATHIVKGVTTNYLLALKQKQNGDPSPDPHGQTYAINGPRNKYDGIHVPVHVRHSNFKLPS
          	  . .:**:****:********:* * *.* :: : ***:*****:**************

MG07117.1 	DSTKPIILVGPGTGVAPMRAFVQERAKQAENGEEVGKTILFFGCRKSTEDFLYKDEWD0E
NCU09741.1	DPSKPIICIGPGTGVAPMRGFIRERVQQAKNGEKVGKTLLFFGCRKSTEDFMYKNEWE0E
FG09786.1 	DPGKPVIMIGPGTGVAPFRGFVQERAKLARDGVEVGKTLLFFGCRKPSEDFMYEKEWQ0E
AN0595.1  	DPSRPIIMIGPGTGVAPFRGFIQERAALAARGEKVGPTVLFFGCRKRDEDFLYKDEWK0V
          	*. :*:* :********:*.*::**.  *  * :** *:*******  ***:*:.**.  

MG07117.1 	YKKVLGDKFELVTAFSREGPKKVYVQHRLKERAQEINELLTKKAYIYVCGDAANMAREVN
NCU09741.1	AKEILGDNFELITAFSREGPKKVYVQHRLKERAEEINQLLEQKAYFYVCGDAANMAREVN
FG09786.1 	YKEALGDKFEMITAFSRESAKKVYVQHRLKERAQEVSDLLSQKAYFYVCGDASNMAREVN
AN0595.1  	FQDQLGDSLKIITAFSRESEKKVYVQHRLKEHAELVSDLLKQKATFYVCGDAANMAREVN
          	 :. ***.::::******. ***********:*: :.:** :** :******:*******

MG07117.1 	SVLGQIIAEQRGIPEAKAEEIVKNMRAANQYQ0EDVWS
NCU09741.1	TVLSQIISEQRGIPEAKAEEIVKNMRSSNQYQ0EDVWS
FG09786.1 	TVLAQIIAEGRGVSEAKGEEIVKNMRSANQYQ0WW---
AN0595.1  	LVLGQIIAAQRGLPAEKGEEMVKHMRSSGSYQ~EDVWS
          	 **.***:  **:.  *.**:**:**::..**     :
```
