# Supplementary material for: Patterns of Intron Gain and Loss in Fungi
Source: PLoS Biol. 2004 Nov 30;2(12):e422. doi: 10.1371/journal.pbio.0020422 (PMC532390; doi:10.1371/journal.pbio.0020422)
Supplement: Table S1 — Also available at http://genes.mit.edu/NielsenEtAl/. (4.3 MB ZIP). [file pbio.0020422.st001.zip › NielsenEtAl/html/1063.html]

AN2181.1.NCU09748.1.MG06107.1.FG05301.1


```
 CLUSTAL W (1.82) Multiple Sequence Alignments - Introns Inserted


Sequence 1: NCU09748.1	115 aa
Sequence 2: FG05301.1	76 aa
Sequence 3: AN2181.1	111 aa
Sequence 4: MG06107.1	116 aa
Alignment Length: 116 aa
Number Identitical Residues: 38 aa
Alignment Score (without introns) 2394


MG06107.1 	MATKKEETFYELYRRTS2LGICLTDALDDLITNDRINPQLAMKILANFDRVVAETLQEKV
NCU09748.1	MAANGNTNYYDLYRHGS2LGSTLTDALDDLIGAERIDPQLAMKVLMQFDRVITEALSEKV
FG05301.1 	-----------------~-----------------------MKILGNFDQAITEALQKNV
AN2181.1  	MSAQA---YYELYRGSS2LGLSLTDTLDDLINEGRIEPQLAMKILSTFDRVITEVLADKV
          	 ::.      .     :  .   :.: ..       ... :**:*  **:.::*.* .:*

MG06107.1 	KARLQFK0GALDNYRFCDDVWTFVIKNINFKLDGGNQTIQADKVKIVSCNAKRPGTDA
NCU09748.1	KARLTFK0GSLDTYRFCDEVWTFLIKNVTFKMDGGQSVVTADKVKIVSCSAKRSDEK-
FG05301.1 	KARLQFK0GSLDTYRFCDEVWTFLIKNVTFKMDSGSQSITANKVKIVSCNAKKPGEGQ
AN2181.1  	RTRLTFK0GHLDTYRFCDEVWTFLIKDVNFKLDN-QQTISADKVKIVSCNSKRPGEA-
          	::** ** * **.*****:****:**::.**:*. .. : *:*******.:*:..
```
