# Supplementary material for: Patterns of Intron Gain and Loss in Fungi
Source: PLoS Biol. 2004 Nov 30;2(12):e422. doi: 10.1371/journal.pbio.0020422 (PMC532390; doi:10.1371/journal.pbio.0020422)
Supplement: Table S1 — Also available at http://genes.mit.edu/NielsenEtAl/. (4.3 MB ZIP). [file pbio.0020422.st001.zip › NielsenEtAl/html/1064.html]

AN8363.1.NCU03255.1.MG00538.1.FG00530.1


```
 CLUSTAL W (1.82) Multiple Sequence Alignments - Introns Inserted


Sequence 1: MG00538.1	592 aa
Sequence 2: FG00530.1	595 aa
Sequence 3: NCU03255.1	610 aa
Sequence 4: AN8363.1	655 aa
Alignment Length: 796 aa
Number Identitical Residues: 124 aa
Alignment Score (without introns) 8881


MG00538.1 	MAVFSG-~---NNPRPAS--AAEYQRLGDSGDAEDDYDGPPGPKMLPVGPAPPRTSRKTL
NCU03255.1	MATLLRR0TTVKMRSPINPQAPGHNPVGLAEKEELVLRPLLAHDTQPSPGQRLKRKLSIL
FG00530.1 	MAAASG-~---LLKGKAGSGSAGYQPVYQDEGGDGHDEGHPTPHVQPIAPRGPMRTYRNM
AN8363.1  	-------~----------------------------------------------------
          	                                                            

MG00538.1 	TAAVVGFIAVCFLAWFATSKRLLFAT--------------------TSTGSRCPCEPDPD
NCU03255.1	LASCAVSLAVLFAFNILACNGTLFGIKPRPSELASPTPLQARDDQRSSGEKDCPCKPTST
FG00530.1 	VIVLSGLVVILMAMNLYLSLPLAFSS--------------------GSGSGSCPGDPHR-
AN8363.1  	------------------------------------------------------------
          	                                                            

MG00538.1 	VPQYFRTSPELWAGPTATGKAPFLQQTVTFDPTATYVPNAPLQTAIPIQGMGSGNKSIFQ
NCU03255.1	VPDYFNTSPGPWIGKTATGKAPFMAQTRTFDHAATYVPNAPLQTQVPIQGWHPGNLSIFG
FG00530.1 	VPQYFQTSPNLWPGVTETGKPAFMAQTHVFEPTATFVPNDPLQTSIPIEGMKEGNRSIFQ
AN8363.1  	---------------MRTIYAALLAGAGLATAIPHASPSVPASAPTGVS--YASGFDMTR
          	                 *  ..::  :      .   *. * .:   :.    .. .:  

MG00538.1 	MMGHLSPYSPSPGFGVREYPLPAGAEIVQVQ0MLSRHGSRYPT----SNSDVAALGKHIS
NCU03255.1	MMGFLTPYTPSTGFGVDEWPLPDGAEIIWLQ0MVSRHGSRYPT----GGSNVESFGARLA
FG00530.1 	MMGYLSPYSPSTGFGVDEYPIPPGAEIVQVQ~MLSRHGARYPT----PGANVATLGERIA
AN8363.1  	SWANLSPYKDAGSFGVPKG-VPKGCELSQVH~VLHRHAERYPTGYPLDGEGMEDFATKLA
          	  . *:**. : .*** :  :* *.*:  :: :: **. ****. .  . .:  :. :::

MG00538.1 	NAKKDKSFKA---SGPLSFLNDWEYQLGHEILVPRGRQELFDSG~RRIPVLIH-------
NCU03255.1	NAT--GKFNA---TGELEFLNNWKYQMGTEILVPRGRQELFDSG~VLHAYMYSSLYD---
FG00530.1 	NAS--ASLKT---SGALEFLNNWKYELGKAILVPRGRQELFDSG1ILHSYMYGSLYN---
AN8363.1  	NYTKTHSVKGPVATGPLSFLNDWEYLLGEDTLMVTGAATEATSG~AEFWIKYGRLLYRPD
          	* ..  ..: . ::* *.***:*:* :*   *:  *      **              ..

MG00538.1 	-------------------CG2KIIVRTT~TQDRMLKSAEYFMA~GFFGLEW2PRN----
NCU03255.1	------------------PNT~KIIARTT0TQDRMLRSAENFLA~GMFGLEW2PNN----
FG00530.1 	------------------PQS~KLIVRTT0TQDRMLKSAENWMA~GFFGLEW2TNN----
AN8363.1  	RDHVAAWDESLNVYPNGTARP~KPVFRTT~SQARILESARWWLS1GFFGNSG~ANSSYEQ
          	 .  :: ..: .  ...:.   * : *** :* *:*.**. ::: *:** .  ...: ..

MG00538.1 	ATIEVIIEEAGFNNSLAGYLNCPSGAGQHVG~DDARKIWVSNYLQNA1TARIQTMIEG-Y
NCU03255.1	VTLEVIIEGSNLNNSLAGYMNCPNEREDGLG~SAARDIWVGHYLQNA1TERFSKLVTG-Y
FG00530.1 	ATIEVIIEAPGFNNSLAGSLNCPN-ADKADY~VTPVAAWYEIYLKDA1TARFNNVTEG-F
AN8363.1  	YDLVVIPEESGSNNTLASYDSCPG--DMTEG2DDDAYVFIPRYTKNA~VARLSAYLPSDF
          	  : ** * .. **:**.  .**.              :   * ::* . *:.    ..:

MG00538.1 	HWTIEDTYAAQNMCPYET0VAYGFSRFCDLFNYEEWIGFGYSIDLWFAGISGFQSPIG0R
NCU03255.1	NWTLDDTYAAQTLCAYDT0VASGYSRFCSLFTYEEWIGFGYSHDLQFYGNNAFGSETG~R
FG00530.1 	TWTAADVWAAQNMCPYET~VAYGFSRFCDLFTYEEWEHFGYSIDLGFSSGAGFQSPIG0R
AN8363.1  	NLTAFDILAMQNLCAYEY~TSFGASAFCSLFTEQEWKDFAYNVDIQYYGDYAYGSPTG~R
          	  *  *  * *.:*.*:  .: * * **.**. :**  *.*. *: : .  .: *  * *

MG00538.1 	ALGIGYQQEVVARLKNHTLGYSGSQINVTLDNNTETFPLNQTLYFDFSHDTNIMAILTAF
NCU03255.1	AIGIGFQQEVLARLQNHTIPYSETQVNVTLDNNTVTFPLNQSLYLDFSHDTNIVSILAAF
FG00530.1 	ATGLGYQQEVMARLKNHTLGYSGSQINTTLDGMTETFPLNQSLYFDFSHDTNIISILTAF
AN8363.1  	AQGIGYVLELAARLQNQLITTSDTSINATLDDNTATFPLDQPFYMDMSHDDIILSVISAL
          	* *:*:  *: ***:*: :  * :.:*.***. * ****:*.:*:*:***  *:::::*:

MG00538.1 	GLRQFAHLMQ----PTTHPGEHNLTVANLTPFGARLDIEIIKTPKPVKPDRSG-------
NCU03255.1	GLTQFEEDLP----ADKYPGEHNFTVSHMTPFGARLDIEIIKTPKPLKADRSG-------
FG00530.1 	GFRQFAEDLP----TDKYPGDHEFVVSKITPFGARLDIEIIKAPQPISPNRED-------
AN8363.1  	GLQYFRFGPHGLPGNVDHAPNRTFSLSEMTPFGARMMSEVWTCPANTSFTSLDPVLYANP
          	*:  *     . ..   :. :: : ::.:******:  *: . *   .    ..   :..

MG00538.1 	--YMVNGGETKYVHFVLNQRTLPLGWSLPECDVSRVDGWCELGAFLSAQDKMPARARFDY
NCU03255.1	--YEDEGEETKYVHFVLNQRTVPLGWSHPECDAERVDGWCEFEAFLKVQEKMPGLARYEE
FG00530.1 	--YQE-GKETKYIHFVLNQRTVPLGKSFPECDVNRKDGWCELDTFIEVQDKMAEKAQFDY
AN8363.1  	LLKSAGAGTSKYIRFLLNGAPLPLKG--LVGCEHAVNGFCPLEGFLSGVPTLKERAQYQR
          	      .  :**::*:**  .:**            :*:* :  *:.   .:   *::: 

MG00538.1 	ACFGSYDPGPY~GSVMDGAPP------------------~--------------------
NCU03255.1	VCFADGGSP--~---------------------------~--------------------
FG00530.1 	ACFGDYTSPPY~GKVTDGVPPS-----------------~--------------------
AN8363.1  	ACFGEYPTGEQ0IIILTGGTFFTMSSTKSVNRGTALPRL0SSLSSWVMPGAAGSVQMYTS
          	.**..  .         . .  : :::.: . .:: .   :: ::   ..::.: .  ::

MG00538.1 	------------------------------------------------------------
NCU03255.1	------------------------------------------------------------
FG00530.1 	------------------------------------------------------------
AN8363.1  	AGSAPLQVTTRPPKPSSIKNFGLLKDLQRGKAAADGSTEANQCPQGESLPSRRIIALKAR
          	:.::. . :: ....:: .. .  .. . ..:::..::.:.......: .:    : .: 

MG00538.1 	-----------------------------
NCU03255.1	-----------------------------
FG00530.1 	-----------------------------
AN8363.1  	HKMQGNPIHSNVRTIERYPHMKTSLDGRI
          	 . ....  :.  : .  .  .:: ..
```
