# Supplementary material for: Patterns of Intron Gain and Loss in Fungi
Source: PLoS Biol. 2004 Nov 30;2(12):e422. doi: 10.1371/journal.pbio.0020422 (PMC532390; doi:10.1371/journal.pbio.0020422)
Supplement: Table S1 — Also available at http://genes.mit.edu/NielsenEtAl/. (4.3 MB ZIP). [file pbio.0020422.st001.zip › NielsenEtAl/html/1065.html]

AN1017.1.NCU07024.1.MG01822.1.FG09612.1


```
 CLUSTAL W (1.82) Multiple Sequence Alignments - Introns Inserted


Sequence 1: MG01822.1	357 aa
Sequence 2: FG09612.1	370 aa
Sequence 3: NCU07024.1	355 aa
Sequence 4: AN1017.1	379 aa
Alignment Length: 414 aa
Number Identitical Residues: 286 aa
Alignment Score (without introns) 11779


MG01822.1 	MAEFVRAQIFGTTFEITS-~------------R2YSDLQPVGMGAFGLVC2SARDQLTNQ
NCU07024.1	MAEFIRAQIFGTTFEITS-~------------R2YSDLQPVGMGAFGLVC2SAKDQLTNQ
FG09612.1 	MAEFVRAQIFGTTFEITSS2PPFIAILTRLARR~YSDLQPVGMGAFGLVC2SARDQLTNQ
AN1017.1  	MAEFVRAQIFGTTFEITS-~------------R2YTDLQPVGMGAFGLVC2SARDQLTAQ
          	****:*************              * *:************** **:**** *

MG01822.1 	NVAIKKIMKPFSTPVLAKRTYRELKLLKHLKHEN0V----------------------IS
NCU07024.1	NVAIKKIMKPFSTPVLAKRTYRELKLLKHLRHEN~VGLPDSIAQAIGNHATNMLAQQVIS
FG09612.1 	NVAVKKIMKPFSTPVLAKRTYRELKLLKHLKHEN0V----------------------IS
AN1017.1  	PVAVKKIMKPFSTPVLSKRTYRELKLLKHLRHEN0I----------------------IS
          	 **:************:*************:*** :                      **

MG01822.1 	LSDIFISPLEDI2YFVTELLGTDLHRLLTSRPLEKQFIQYFLYQIM0RGLKYVHSAGVVH
NCU07024.1	LSDIFISPLEDI~YFVTELLGTDLHRLLTSRPLEKQFIQYFLYQIM0RGLKYVHSAGVVH
FG09612.1 	LSDIFISPLEDI2YFVTELLGTDLHRLLTSRPLEKQFIQYFLYQIM0RGLKYVHSAGVVH
AN1017.1  	LSDIFISPLEDI2YFVTELLGTDLHRLISSRPLEKQFIQYFLYQIM0RGLKYVHSAGVVH
          	************ **************::***************** *************

MG01822.1 	RDLKPSNILVNENCDLKICDFGLARIQDPQMTGYVSTRYYRAPEIMLTWQKYDVEVDIWS
NCU07024.1	RDLKPSNILVNENCDLKICDFGLARIQDPQMTGYVSTRYYRAPEIMLTWQKYDVEVDIWS
FG09612.1 	RDLKPSNILVNENCDLKICDFGLARIQDPQMTGYVSTRYYRAPEIMLTWQKYDVEVDIWS
AN1017.1  	RDLKPSNILINENCDLKICDFGLARIQDPQMTGYVSTRYYRAPEIMLTWQKYDAKVDVWS
          	*********:*******************************************.:**:**

MG01822.1 	AGCIFAEMLEGKPLFPGKDHVNQFSIITELLGTPPDDVINTIASEN0TLRFVKSLPKRER
NCU07024.1	AGCIFAEMLEGKPLFPGKDHVNQFSIITELLGTPPDDVINTIASEN0TLRFVKSLPKRER
FG09612.1 	AGCIFAEMLEGKPLFPGKDHVNQFSIITELLGTPPDDVINTIASEN0TLRFVKSLPKRER
AN1017.1  	AACIFAEMLLGAPLFPGKDHVNQFSIITELLGTPPDDVIQTICSEN0TLRFVKSLPKREP
          	*.******* * ***************************:**.*** ************ 

MG01822.1 	QPLK-----------NKFKNADP---SA1IDLLERMLVFDPKKRITATEALAHEYLTPYH
NCU07024.1	QPLK-----------NKFKNADS---SA1VDLLERMLVFDPKKRITATEALSHEYLAPYH
FG09612.1 	QPLR-----------NKFKNADD---SA1IDLLERMLVFDPKKRITATEALAHDYLSPYH
AN1017.1  	QDLAKLPKFLALVHPDKKPEEDEDYKNT1INLLKAMLVYNPKDRISAEAALAAPYLAPYH
          	* * . ..  :   .:*  : * . ..: ::**: ***::**.**:*  **:  **:***

MG01822.1 	DPTDEPIAEEKFDWSFNDADLPVDTWKIMM2YSEILDYHNAEAGMQQMDDQFTGQ-----
NCU07024.1	DPTDEPVAEEKFDWSFNDADLPVDTWKIMM~-----------------------------
FG09612.1 	DPTDEPVAEEKFDWSFNDADLPVDTWKIMM2YSEILDYHNVEAGVTNMEEQFNGQ-----
AN1017.1  	DETDEPVAEEKFDWSFNDADLPVDTWKIMM2YSEILDFHNIDQGGDINPALVEGAGLNQQ
          	* ****:***********************  :.  .  . . .         . . ...

MG01822.1 	---
NCU07024.1	---
FG09612.1 	---
AN1017.1  	GFQ
          	. .
```
