# Supplementary material for: Patterns of Intron Gain and Loss in Fungi
Source: PLoS Biol. 2004 Nov 30;2(12):e422. doi: 10.1371/journal.pbio.0020422 (PMC532390; doi:10.1371/journal.pbio.0020422)
Supplement: Table S1 — Also available at http://genes.mit.edu/NielsenEtAl/. (4.3 MB ZIP). [file pbio.0020422.st001.zip › NielsenEtAl/html/1066.html]

AN0260.1.NCU04646.1.MG06763.1.FG01839.1


```
 CLUSTAL W (1.82) Multiple Sequence Alignments - Introns Inserted


Sequence 1: NCU04646.1	513 aa
Sequence 2: FG01839.1	500 aa
Sequence 3: MG06763.1	506 aa
Sequence 4: AN0260.1	500 aa
Alignment Length: 523 aa
Number Identitical Residues: 269 aa
Alignment Score (without introns) 13714


MG06763.1 	MLRQDFSRPDAKRRNVTDHRKKQFADPAYKATDYPHRMNFYAIPPTADITLEQFEQWAID
NCU04646.1	MLRSNVSRIDPKRRNVLDHRKKQFAEAAYKDTDYPHRLNFYSTPPTADITIEQFEQWAID
FG01839.1 	MLRQDFNRIDPKRRNVVDHRKKQFASPTYKDLDYPYRLNFYTDPPTADITLEQFEQWAID
AN0260.1  	MIRQEFNRIDPKRRANLNYKKTQFATPTFKQQDYPYRLNFYDTPPTAEITLEQFEQWAID
          	*:*.:..* *.***   :::*.*** .::*  ***:*:***  ****:**:*********

MG06763.1 	RLRI~LAELEACSFRNKTSQETALHMKPLLDKYLPLEANSSAST-----QLHAQRQKDHY
NCU04646.1	RLRI1LAELEACSFRNKTPAETASHMKPLLDKYLPLDTNSSSSS-----QLFAQRQKDHY
FG01839.1 	RLRV1LAELEACAFRNKTPAETATHMKPILKQHLNLEANSSSSK-----KLFEQRQKDHY
AN0260.1  	RLKI1LAEIEACSYRNKTAAETTAHITPLLQKFLPLSANTSSPKGAADPRIKNERQKDHY
          	**:: ***:***::****. **: *:.*:*.:.* *.:*:*:...::..::  :******

MG06763.1 	GHFILRLAFCSTEDLRRRFVRVETMLFRMRLAADDSRERAAFIASLDGLEW-EPVPEDER
NCU04646.1	SHFILRLAFASTEDLRRRFTRVETMLFRMRLNADDGRERAAFINSLN-LDW-ETVSDEEK
FG01839.1 	SHFILRLAFSSTEDLRRRFTRVETMLFRLRLNEDDLSERSAFVKTLG-LDWCEDVTEEDR
AN0260.1  	SHFILRLAFSATEDLRRRFARAETMLFRFRFQADDSRERRAFIDSLN-LDW-ESVGEDER
          	.********.:********.*.******:*:  **  ** **: :*. *:* * * ::::

MG06763.1 	RSLSAELAAVAGWKKESAG---------DDEMWCKVDWERVPDLVEGRRVLLKAGKAYVP
NCU04646.1	RELAAELAATASFGGYKKGQQQQQQYEEDQQTWCKVSWLRVPELVEQRRVFLRQGYAYVP
FG01839.1 	REYAAELAAFTSN---RKG-------ENDDDTWFKVDWERVPDLIESRRVFLKAGKAFVP
AN0260.1  	RELSEYLVAATPG--LRRS---------DEDTWYKVDWERVPELVERRSVFLSKGKAYVP
          	*. :  *.* :       .         *:: * **.* ***:*:* * *:*  * *:**

MG06763.1 	AKEQTSMVVTEFTSRLEKALE0LTARALPRLDEDDRLTPILNHLSKNFITPDASYGSGGD
NCU04646.1	AREQQAMVVSEFSSRLERQLE~LTARALPRLDEDDRLTPILAHLSKNFITPDASYVG--T
FG01839.1 	GREQTGMVISEFTSRLERQLE0LTARALPRLDEDDRLTPILNHLSKNFITPDASYTS--S
AN0260.1  	EREQLSMIIAEFTARLERALE0LTSRALPRLDEDDRLSPILNHLSKNFGSAESVYTE--G
          	 :** .*:::**::***: ** **:************:*** ****** :.:: *     

MG06763.1 	DQAAPGSELTAANVDKLSS-EHFPLCMQHLHRSLRRDSHLKHFGRLQYSLFLKGIGLSLE
NCU04646.1	SSAISSADISARNIDTLVNNHHFPACMSHLHRTLRRDAHLKHYGRLQYTLFLKGIGLNLE
FG01839.1 	TAAVPGAEISAANIDNLS--QHFPACMSHLHRSLRRDGHLKHFGRLQYSLFLKGIGLNLE
AN0260.1  	EGFVDGAPITAASIDPLS--QHFPLCMRSLHMSLRKNNHLKHFGRLQYTLFLKGIGLSLE
          	     .: ::* .:* *   .*** **  ** :**:: ****:*****:********.**

MG06763.1 	ECLVFWRSSFNKITDDTFNKEYRYNVRHVYGDVGGDANRRGRGYSPFSCQKILTEHPPGP
NCU04646.1	ECLLFWRQSFNKITDDTFNKEYRYNVRHTYGDVGGDSNRRGGGYSPYSCQKILTEHPPGP
FG01839.1 	ECLVFWRKSFNNMTDDKFNKEYRYNIRHVYGDVGGDSNRRGGGYSPFSCQKILTEHPPGP
AN0260.1  	ECILFWRQSFKGFTDEEFNSRYKYNVRHAYGDVGGDINRRGRGYPPYSCQKILSDTNPGA
          	**::***.**: :**: **..*:**:**.******* **** **.*:******::  **.

MG06763.1 	GEAHGCPYRHFNLENLTALLQQ-VGINDRSVLNGVREDKEKQKFHLACNR2VFEYVHKNE
NCU04646.1	GEAHGCPYRHFNMENLQTLLQQGMGVTDRGVLNGVKEDKEKQKFHMACNR2VFEHLHKEE
FG01839.1 	GEAHGCPYRHFNLENLSALVQA-MGVNDRSVLQGVKEDKDKQKFHMACNR2VFEHLHKQE
AN0260.1  	GQTHGCPYRHFSVDNLIGLLQS-TGVNDKDLLRGVREDVEKTRYHIACNR~VFEYTHKAE
          	*::********.::**  *:*   *:.*:.:*.**:** :* ::*:**** ***: ** *

MG06763.1 	IRRAKDEGIMTVAQLETIVHPNEYFKRSYLLKHLDSSKDGDVKMDG
NCU04646.1	LKKAKDEGIMTAAQLETIVHPNEYFKRSYLLKNMG-KMQGDVKMEG
FG01839.1 	IRKAKDEGVMTQSQLETIVHPNEYFKRSFLLKNLG-KET-DVRMEG
AN0260.1  	IKRAKEDGSAGEIELDTIVHPNTYFKRSYLLKQLG-KTPRTA----
          	:::**::*     :*:****** *****:***::. .    .
```
