# Supplementary material for: Patterns of Intron Gain and Loss in Fungi
Source: PLoS Biol. 2004 Nov 30;2(12):e422. doi: 10.1371/journal.pbio.0020422 (PMC532390; doi:10.1371/journal.pbio.0020422)
Supplement: Table S1 — Also available at http://genes.mit.edu/NielsenEtAl/. (4.3 MB ZIP). [file pbio.0020422.st001.zip › NielsenEtAl/html/1067.html]

AN6904.1.NCU07549.1.MG08906.1.FG06703.1


```
 CLUSTAL W (1.82) Multiple Sequence Alignments - Introns Inserted


Sequence 1: NCU07549.1	138 aa
Sequence 2: FG06703.1	128 aa
Sequence 3: MG08906.1	137 aa
Sequence 4: AN6904.1	137 aa
Alignment Length: 138 aa
Number Identitical Residues: 59 aa
Alignment Score (without introns) 3416


MG08906.1 	MTIRPLQKVSVG-Q0NGVGAFILQ~CKRLEFHYCDWAGSSKGMN2QFIRQHLPTFAAKNP
NCU07549.1	MTVKALTQISSAGR0NGVGAFVLQ~CKKLDIHYSDWAGSSRGMN2GFIKSLLPKFAAANP
FG06703.1 	MTVKALRQIAKG--~--------Q0CKKLDFYYCDWAGSSKGMN2GFIKSLLPKFAAANP
AN6904.1  	MPVQGVRAVSTA-R0NGVGAFILQ~CKRLDFHYCDWAGSSRGMN2AFLKHALPAFAKENP
          	*.:: :  :: .   .. .:   * **:*:::*.******:***  *::  ** **  **

MG08906.1 	QIEITVSPRPQKHPVVVGHFINGNNRPVCVRNLDANQILKKVELLREMNGEVNKKFSKPV
NCU07549.1	QIEFVVSPRPAKHPILMGHYINGRTKAICVRNMEPLEILKKAELLRDASGEKPQKFKKPV
FG06703.1 	QVEFAISPRPGKHPVIIGHYINGLHKPICVRNLSPYEILKKAELLRDASGEKLKKHNQAV
AN6904.1  	QIEIRVSPRPHKHPIIKGHYINGREKAICVRNLEPEQITQKANLLKQASGEKLKRTKKPV
          	*:*: :**** ***:: **:***  :.:****:.. :* :*.:**:: .**  :: .:.*

MG08906.1 	RSINESVRGVWSPYHGNGMPV
NCU07549.1	TSTNPSVRGVWSPYHGQGMAV
FG06703.1 	TSTRPSVRGVWSPYHGKGTPV
AN6904.1  	TSINESVRGIWSPYHGGLKSV
          	 * . ****:******   .*
```
