# Supplementary material for: Patterns of Intron Gain and Loss in Fungi
Source: PLoS Biol. 2004 Nov 30;2(12):e422. doi: 10.1371/journal.pbio.0020422 (PMC532390; doi:10.1371/journal.pbio.0020422)
Supplement: Table S1 — Also available at http://genes.mit.edu/NielsenEtAl/. (4.3 MB ZIP). [file pbio.0020422.st001.zip › NielsenEtAl/html/1068.html]

AN3673.1.NCU02970.1.MG05621.1.FG01377.1


```
 CLUSTAL W (1.82) Multiple Sequence Alignments - Introns Inserted


Sequence 1: NCU02970.1	121 aa
Sequence 2: MG05621.1	137 aa
Sequence 3: FG01377.1	118 aa
Sequence 4: AN3673.1	120 aa
Alignment Length: 138 aa
Number Identitical Residues: 45 aa
Alignment Score (without introns) 2363


MG05621.1 	MSISNEALSK0LIQEIELKSIQAQQQISMVRSQQASKQREMRLAELTRSEISSLPTETPV
NCU02970.1	MSISNEALQK0LVREIEAQSIAAQQQIGLVRTQQASKQREMRLAQLTRNELATLPETTAV
FG01377.1 	MSIPNEALQK0LVREIESQALVAQQQIGLARTQMTSKQREQRLVKLTMNEMSTLPSDAVV
AN3673.1  	MSIPNEALQK0LLQEIESRVITSQQQIGITKAQMTSKNRDIRMLELTSKELSSLPADTNV
          	***.****.* *::*** : : :****.:.::* :**:*: *: :** .*:::**  : *

MG05621.1 	YEGLGKI2INDKVIAVNVLIHGFKR~FVLTPASDLNNKLGKQVKELGTDIENLGKRLHYL
NCU02970.1	YEGVG--~---------------KM2FVSVPVPALKDRLSTEMKEMETEVENLGKRLHYL
FG01377.1 	YEGVG--~---------------KM2FVSLPVDSLRQKLEGQTQTLEGEVDKLSQRLLYL
AN3673.1  	YEGVG--~---------------KM2FVAVPRATVDKRISTETGELKGEIENLEKRMNYL
          	***:*                  *  **  *   : .::  :   :  ::::* :*: **

MG05621.1 	ETTAKNSQSHIDQILKRGDA-
NCU02970.1	ETTAKNSQEHIEKMLKGGGQA
FG01377.1 	ETTHKNSREHIEQMLRTK---
AN3673.1  	EMTFKNSRENLEAILKSGRA-
          	* * ***:.::: :*:
```
