# Supplementary material for: Patterns of Intron Gain and Loss in Fungi
Source: PLoS Biol. 2004 Nov 30;2(12):e422. doi: 10.1371/journal.pbio.0020422 (PMC532390; doi:10.1371/journal.pbio.0020422)
Supplement: Table S1 — Also available at http://genes.mit.edu/NielsenEtAl/. (4.3 MB ZIP). [file pbio.0020422.st001.zip › NielsenEtAl/html/1069.html]

AN8825.1.NCU00269.1.MG01661.1.FG05558.1


```
 CLUSTAL W (1.82) Multiple Sequence Alignments - Introns Inserted


Sequence 1: NCU00269.1	954 aa
Sequence 2: FG05558.1	1051 aa
Sequence 3: AN8825.1	980 aa
Sequence 4: MG01661.1	946 aa
Alignment Length: 1130 aa
Number Identitical Residues: 288 aa
Alignment Score (without introns) 15211


MG01661.1 	----------------~----------~--------MDDDDKSKVQ--------------
NCU00269.1	----------------~----------~--------MEDGHHSPD---------------
FG05558.1 	MNLVEKATSLRVNKHK2HVGTSKKFIQ2PSVGPIVSEDRGHKIPTNATICSSFDQVGESV
AN8825.1  	-----------MSTHD~NADRQSEFVA~DAVTAMKLEQSENNTDAPILNGG---------
          	            .. .   . ...     :  .    :  .:        .         

MG01661.1 	------------~------------------------------------------LVDKE
NCU00269.1	------------~-------------------------------------------STKE
FG05558.1 	LDSNWLRPSHRI0AYARSTTFNTPLIPLAVFARIIPRVCIDSSTRLFASRQYLGQTSTPR
AN8825.1  	------------~----------------------------------------GAAMKPD
          	                                                     .      

MG01661.1 	GRA--------------EPEVNGNGIVKMETQNSATDSAQRSRSPSSMLR--------DG
NCU00269.1	SKS-------------EEHSKMNGSKLKRDGASNAGNAATPNGSQTGIS-------RPPS
FG05558.1 	SKLGLWAIASDVLMEDDEYTTSKMEEIKLEEGANDAQVKQETNTPMSITNGDHESSRSPT
AN8825.1  	SKAAS---PEPLIKDERASSTFMKSRSSSRTPSSRTPLKKEHSDSEDIQE-----KRGDD
          	.: .    ..    ..           .     .            .:       .    

MG01661.1 	ETQTVDDGDKISPRNASPDAKS---TRKNPQ-QLPMRTSKLFSHLADVTEEASRGFAILS
NCU00269.1	MSPDEHKAASESTATPSENVPTQKPSRKASQKNMK-REPVLFNHLPDVREEACTHFQVIH
FG05558.1 	ASQDGLKSRSESADTPSSNRPS-KLSRKASQKLAASREPVLFDHLPDMTTESCKFFQRIP
AN8825.1  	ASGTEKVGGGISVKMEPGQPPK--LARSSSQKVVP-RPPQLFLDLPDSTEEAQKTFEVIE
          	 :     .   *    . :  .   :*. .*.    * . ** .*.*   *:   *  : 

MG01661.1 	DCVYASKALGNSGQETLDCDCEEDWR1-----------------~--DGLNHACAEDSDC
NCU00269.1	DCLYGSKNMGASEHDALDCDCAEEWR1-----------------~--GDMNHACGEDSDC
FG05558.1 	DCLYGSKHLGSTDNDALDCECRDEWH1-----------------~--DGKNLACGEDSDC
AN8825.1  	TCQYANKYMGYTEH-AMECDCAEEWV1LVVVLAPSPSFRVPSQN1PASSTNRACGEDSDC
          	 * *..* :* : : :::*:* ::*       :.:.:   .:.. .:.. * **.*****

MG01661.1 	INRVTKIECVS--GNCGDGCQNQRFQRKQYANVSVIKTENKGYGLRADANLEPNDFVFEY
NCU00269.1	INRATKMECVDGDCNCGSGCQNQRFQRKQYADVSVIKTEKKGFGLRANTDLQVNDFIFEY
FG05558.1 	INRATKMECSAEGGNCAGGCQNQRFQRKQYANVSVIKTEKKGFGLRADSDLQPNDFVFEY
AN8825.1  	INRATKIECMG-DCGCGPDCQNQRFQRREYANVAVIKTEKKGYGLRAEEDLRPHQFIFEY
          	***.**:**   . .*. .********::**:*:*****:**:****: :*. ::*:***

MG01661.1 	IGEVIGEELFRSRLMKYDTQRLEHFYFMSLTRTEYVDATKKGNLGRFCNHSCNPNCYVDK
NCU00269.1	IGEVINEPTFRSRMVKYDKEGIKHFYFMSLTKSEFVDATKKGNLGRFCNHSCDPNCYVDK
FG05558.1 	IGEVINEPTFRRRMIQYDEEGIKHFYFMSLNKSEFVDATKKGNYGRFCNHSCNPNCYVDK
AN8825.1  	VGEVINEGPFHRRMRQYDAEGIKHFYFMSLSKGEFVDATKKGNLGRFCNHSCNPNCYVDK
          	:****.*  *: *: :** : ::*******.: *:******** ********:*******

MG01661.1 	WVVGDKLRMGIFAMRAIKAGEELCFNYNVDRYGANPQRCYCGESNCSGILGGKTQTERTT
NCU00269.1	WVVGDKLRMGIFAGRAIKAGEELVFNYNVDRYGADPQPCYCGEPNCTGFIGGKTQTERAT
FG05558.1 	WVVGDKLRMGIFTSRKIQSGEELVFNYNVDRYGADPQPCYCGEPNCVGFIGGKTQTERAT
AN8825.1  	WVVGEKLRMGIFAERHIQAGEELVFNYNVDRYGADPQPCYCGEPNCTGFIGGKTQTERAT
          	****:*******: * *::**** **********:** *****.** *::********:*

MG01661.1 	KLPLAMIEALGIDDGDHWESSVRK----PRKKKAGESEEEYVGSIQPRKLEDGEVGVVMS
NCU00269.1	KLPPATIEALGIEDGDSWDTAVAATVKKPRKKKATEDDEEYINRFEPRGLDEEGVTKVMA
FG05558.1 	KLPAATVEALGIDGGDGWDTSVAK---KPRKKKPDEDDEEYVNSIRPRSLSEDDARKVMA
AN8825.1  	KLSNATIEALGIEDADGWDTAVAK---RPRKKKMGEEDEEYVDSVQPKSLDESGVTKVMA
          	**. * :*****:..* *:::*      *****  *.:***:. ..*: *.:  .  **:

MG01661.1 	TLRSCKEKWVATKLLHRILAVDEERVLNRVVKFHGYEYLKTTLNTFKDDNEVVFQVLSIL
NCU00269.1	TLMQCKEKWIAVKLLGRLQNADDDHVRNRVVKMHGYQILKTTLNTFKEDTNVVLQILDIL
FG05558.1 	ALMQCKEKWIAVKLLDRIMQCDEERVIHCVMRMHAYQILKTTLNTFIDDHNVVLQVLDIL
AN8825.1  	ALMQCKEKWIAVKLLGRIQRCDDERVRNRVVKMHGYQILNSQLAMWKDDFNVVLQILDIL
          	:* .*****:*.*** *:   *:::* : *:::*.*: *:: *  : :* :**:*:*.**

MG01661.1 	YKLPRVTRNKIDDSNIEPLITELSNSKHEEIAAESKKLLEVWKTLQVGYRIPRAKADRSA
NCU00269.1	YQLPRITKNKITDSNIEAAVEPLTHSDHEDVASQSKRLLQEWSKLETAYRIPRKKLDPSA
FG05558.1 	DKFPRLTRNKVQDSKIEATIEGLTQSEHEDVASKSKHLLNEWSKLEVAYRIRRRKFDPNA
AN8825.1  	DKFPRLTRNKIIDSKIESTIQPLTSCGDERVEQKATVLLQLWSTLEIGYRIPRMKRDPNA
          	 ::**:*:**: **:**. :  *: . .* :  ::. **: *..*: .*** * * * .*

MG01661.1 	RDNAGSFFDDRRQQAREAASAPLPRAPSPVR---NAPTGPRSSMP--QRNPHYVPPRRKY
NCU00269.1	PVTTNSFEDDRRNVNHE-EHPSRPVNPFEN---MVVPTGPRSNIP--QRNMNYFNNQ-RP
FG05558.1 	PAAN-SFEE-RRGAGRE-EETVQSTSKTASPTPIDAPKGPRNSMP--QRNNAFFQNGGRS
AN8825.1  	ATPTVSQFHRRDDISDERQQRPRSRSRSRS---IEAPRGPAAQKRGGQGPRNQHHQGPRT
          	     *  . *     *      .           .* **  .  ..*          : 

MG01661.1 	PQQPPRDAPALPSGWRSAIDQRTGRQYYWETANPDKKSWVRPTSEMAK-------ANAAL
NCU00269.1	RKLPTN----LPAGWFVTTD-STGKYYFYDKSG--HTQWQRPTTPAVD-VPKPS-AKVEQ
FG05558.1 	RRPPFNAS--LPQGWFTAKD-AAGNTYFYTKQG--ATTWQRPTQPATEPAAKAP-SKAMK
AN8825.1  	FRRRFDP---LPQGWFAAE--SNGRTYYYSARG--DTTWTRPTKPAPQPPPPPKESRDKA
          	 :        ** **  :     *. *::   .   . * ***    .. . . .:.   

MG01661.1 	QAQKIQDIINQCAQPTPKPSSATHTPQPVG-TPVAEPKRETWRSWPADKQRRLYENT0IF
NCU00269.1	NQKALQDIIDSLTKEPTPRHSANQTPKSNTPVPD-NGKKEKWRSLPVEKQMKIYENT0LF
FG05558.1 	EQLAIQSIINQVTEKGTPKHTSVSTPKAAETPPK-EVKEEKWRSLPVDKRMKIYENT0LF
AN8825.1  	LQSIIDGIMNAKEQTPKEKSGTPTTPQPSKPTPEGKDRQEKWRSYSEEKQKKLYENT0LY
          	    ::.*::   :       :  **:.  . * .: :.*.*** . :*: ::**** ::

MG01661.1 	PHIKYVADKYYKRLPKEDLKKFVKD~VNKTLAASDYKHGRVNDP-SKVEEKQQSKIRKYT
NCU00269.1	PHVKYVMDKFHRRLPKEDLKRFGRE~INKKLVASDYKNHRVDDP-TTISSNQARKIKKFV
FG05558.1 	PHIKHVLDKFHHKLPKEELKRFGKD~IAKKLVASDFKNNRVEDPGAPLSDKQVKKIKQYV
AN8825.1  	PHIKYVVDKFKHKLPKDDLKRYAKD0VAKKLVNSDFKNNRVTDP-TKIDDKQQKKVKKFC
          	**:*:* **: ::***::**:: :: : *.*. **:*: ** ** : :..:*  *:::: 

MG01661.1 	RDFLDKAVKKHELRQAEKASANNKSLEDGAAEPSGSTLSGAAEGSKKSPPGTDARVQSTN
NCU00269.1	KDFFDRAVVKHRENEQRAAQKAGPSSSGAPSPTNGGSAKSPLGRNGASNATVKTQQPDAD
FG05558.1 	KDFLDRAVKKYGEHKRKADEDADTQMKDDQGPSAAGSGAGSVVDGSDGTALAKVDG-TSM
AN8825.1  	KEFFDKAVAKHQAHEKRKAEKLAKEGSSD------NKLATPVGGQSEGDGTPDVKMSDDE
          	::*:*:** *:  .: .  .    . ..       ..   .      .    ..      

MG01661.1 	DSAPRSADSPDSSATDLKRKRGESHDVENVEGAAADLTPGYTPLAKRVKETDVEEPSPPP
NCU00269.1	GDIVLTDVEDEGENTPATSSSDRKRKR-AEEQEVPAPASEAIPSPKRAKEDSTTEDSIPS
FG05558.1 	GEVDVTAVSDR-EGTGSLGSPDRKRKRDLDTSGSPYVTSTDGPNMKRLREDELEAPSPPP
AN8825.1  	GSTGR-------EGTGSLK---RKRDE-LSVGNTNTPDDTPTSSTKRQR------SSTPP
          	..          . *       ..:.                .  ** :       * *.

MG01661.1 	PPPTPPPLDELDDAVMIEEATEGRRLREHEDELRLENEEAHRLQQDAVEQKRLREHEEAL
NCU00269.1	PPP-PPPPP-TDTPLTEEERS----MREQEEALMRENEEAQRLEDEE------AERRVSV
FG05558.1 	PPP-PPPQSDMDEVVTAEQEA----LREQEEALMRENEEAQRLEDEA------SHTKGLE
AN8825.1  	PPP-PPAMNTNDNNNDNDDMS----VRSDEPDADADADEVVLVGNPTPPPPPPPPPQEDM
          	*** **.    *     :: :    :*..*     : :*.  : :           .   

MG01661.1 	ERENQETLLALQTDQRGPE-----------------PDGDTPMANNVNGTKMGTVMV
NCU00269.1	TVQGAAVATSKVNGVNGTKAHHTSEASPAVSDENGMDAGRDEKSHEQMAQQEPVSR-
FG05558.1 	DVLDASNEISRLN----------------------------KEARKPGSQKMPA---
AN8825.1  	RIPDADAETNGHN-----------------------FEGYGEMNRSHQAQIGIEGNV
          	   .        .                         .     ..  .
```
