# Supplementary material for: Patterns of Intron Gain and Loss in Fungi
Source: PLoS Biol. 2004 Nov 30;2(12):e422. doi: 10.1371/journal.pbio.0020422 (PMC532390; doi:10.1371/journal.pbio.0020422)
Supplement: Table S1 — Also available at http://genes.mit.edu/NielsenEtAl/. (4.3 MB ZIP). [file pbio.0020422.st001.zip › NielsenEtAl/html/107.html]

AN0290.1.NCU07471.1.MG09902.1.FG01226.1


```
 CLUSTAL W (1.82) Multiple Sequence Alignments - Introns Inserted


Sequence 1: NCU07471.1	388 aa
Sequence 2: FG01226.1	282 aa
Sequence 3: MG09902.1	288 aa
Sequence 4: AN0290.1	266 aa
Alignment Length: 391 aa
Number Identitical Residues: 173 aa
Alignment Score (without introns) 7890


MG09902.1 	-MAADPFDSAL2DLLRRLDPKHTTRHLNGLMTIVPDLTEDLLSSVDQPLTVRRCKQTGRE
NCU07471.1	MATADPFDSAL2DLLRRLNPKHTAEHLNNLITLAPDLTEDLLSSVDQPLTVKRCKQTGRD
FG01226.1 	-MAVDPFDSAL2DLLRRLNPKQTTDHLNAIISIAPDLTEDLLSSVDQPLTVRRCKQTGRD
AN0290.1  	-MADAQFDSAL2DLLRRLNPRDTKQNLQAITSIVPDLTEDLLSSVDQPLEIRRCPKTKRD
          	  :   ***** ******:*:.*  :*: : ::.*************** ::** :* *:

MG09902.1 	YLLCDYNRDGDSYRSPWSNEFDPPLDDG-PGGLGGVGPQGGNEG-AGELGVPGERVRKME
NCU07471.1	YLLCDYNRDGDSYRSPWSNQFDPPLEGGNQGGSGGDGEGDGGEGGAAGSIMPGERVRKME
FG01226.1 	YLLCDYNRDGDSYRSPWSNQFDPPLDEAGSGGVGAGGNEGAGEG-----AIPSERVRKME
AN0290.1  	YLLCDYNRDGDSYRSPWSNEFDPPLDDG---------------------TVPSERVRRLE
          	*******************:*****: .                      :*.****::*

MG09902.1 	VKANEAFDVYRDLYYEGGVSSVYLWNLDDGFAGVVLLKKA1APQGGNNEGVWDSIHVFEA
NCU07471.1	IKANEAFDVYRELYYEGGVSSVYFWNLDDGFAGVVLLKKS1SPTNPSSSGVWDSIHVFEA
FG01226.1 	VKANEAFDVYRDLYYEGGVSSVYFWNLDDGFAGVVLLKKS1SPQGGNSEGVWDSIHVFEA
AN0290.1  	VAANEAFDVYRELYYEGGVGSVYFWDLDDGFAGVILLKKG1VSPGGKHSGEWDSIHVFEA
          	: *********:*******.***:*:********:****.  . . . .* *********

MG09902.1 	SERGRSTTYRLTSTVILTLSAGGGDSALGDMNLSGNMTRQLEQDMRTAEGDESHIANLGR
NCU07471.1	SERGRTSNYRLTSTVILSLATKGN--ALGEVDLSGNMTRQVEQDLP-VENDESHIANIGR
FG01226.1 	IERGRSTHYKLTSTVILTLSTSGG--NLGEMDLSGNMTRQVEQDLP-VENDDSHIANVGR
AN0290.1  	TDRGRMAHYKLTSTVILHLSNENE--ALGEMDLSGNMTRQIEVDMN-VDSDASHVANVGK
          	 :*** : *:******* *:  .    **:::********:* *:  .:.* **:**:*:

MG09902.1 	LVEDMELKMRNLLQ~EVYFGKAKDVVGDLRS~LGSLSEGQRDRDAQREIIGSMQR-~---
NCU07471.1	LVEDMELKMRNLLQ~EVYFGKAKDVVGDLRS~VGSLSEGQRDRDAQMEIIGSMRKA2CLN
FG01226.1 	LVEDMELKMRNLLQ~EVYFGKAKDVVGDLRS~IGSLSEGARDREAQRELIGSMRK-~---
AN0290.1  	LVEDMELKMRNLLQ1EVYFGKAKDVVGELRS1IGPLSETNRDRATHQEMIRGLQR-~---
          	************** ************:*** :*.***  *** :: *:* .:::     

MG09902.1 	------------------------------------------------------------
NCU07471.1	YGLGLTDGTRPDQFDQPPGPSILLNRSETRHPKNIPGNAHTQRHVSQKRDFAYRLPSDPA
FG01226.1 	------------------------------------------------------------
AN0290.1  	------------------------------------------------------------
          	                                                            

MG09902.1 	------------------------------------
NCU07471.1	DPDLFPPRHFFFWRIPGCFLMGQITDFVGFGGETLV
FG01226.1 	------------------------------------
AN0290.1  	------------------------------------
          	
```
