# Supplementary material for: Patterns of Intron Gain and Loss in Fungi
Source: PLoS Biol. 2004 Nov 30;2(12):e422. doi: 10.1371/journal.pbio.0020422 (PMC532390; doi:10.1371/journal.pbio.0020422)
Supplement: Table S1 — Also available at http://genes.mit.edu/NielsenEtAl/. (4.3 MB ZIP). [file pbio.0020422.st001.zip › NielsenEtAl/html/1070.html]

AN0117.1.NCU04337.1.MG06072.1.FG05072.1


```
 CLUSTAL W (1.82) Multiple Sequence Alignments - Introns Inserted


Sequence 1: NCU04337.1	381 aa
Sequence 2: MG06072.1	389 aa
Sequence 3: FG05072.1	377 aa
Sequence 4: AN0117.1	546 aa
Alignment Length: 547 aa
Number Identitical Residues: 158 aa
Alignment Score (without introns) 8976


MG06072.1 	MFNWAKQQ2LANVAGTQEPIYGPTAIKSVA-QETSNISHTELTRDDLKWRAMTSTCVETQ
NCU04337.1	MFKWAQAA2LANVAGTKEPIYGPEAIRSVA-EEAKTTPYTETTKDDLKWQAMESTCVETQ
FG05072.1 	MFNWAKQQ2LANVAGTQEPIYGPSAIKSVA-IEAEKTPYTEVTRDGLKWKAMDSTSVETE
AN0117.1  	-MNWLKST2LSAVAGTQEPIYGPEAIQPVSQQQTEDAPYTELTKHDLRWRAYQYTNVETQ
          	 ::* :   *: ****:****** **:.*:. ::.  .:** *:..*:*:*   * ***:

MG06072.1 	TFYMTTDSGHLAFVQVIYSNVA2GIRTTCQFNTKVFNLKDDGKPHLWCTTPLSNHSFSDD
NCU04337.1	CFYFMTDSGQLAFAQVIYSNVA2GIRTTCQFNCKVFSL-DGSKPHLWCSTPLNNHEFSED
FG05072.1 	SFYITADNGYIALAQVIYSNVA2GIRTTCQFNCKVFDK-DPTKPHLWASTPLNNQDFNED
AN0117.1  	TYYAMADNGTLVMVQIIYSNIA2GIHTTAQFNCKIFNTSGDGTPHIWFSDPLYNHMFDES
          	 :*  :*.* :.:.*:****:* **:**.*** *:*. ..  .**:* : ** *: *.:.

MG06072.1 	MTAFYADDCAVELSEDGTSYTIKSMTDERAIVNIKVTRTTPGFHAGKTGTTLFGTDLSDP
NCU04337.1	KTSFYATDCAVELSEDGNSYTIKSLNDERSIVNVTIKRTAPGFKIGTSGTTLFGTDLANP
FG05072.1 	KTSFYADDCAVELSEDGTYYTIKSMNSQDAIVNLKITRSTPGFQAGTTGTTLYGTDHNNP
AN0117.1  	MSSFAADNISLSLNEEGNAYTLKSAVNEGCLVDLTFNRAAPGFAIGKDGTTYFGTDPQNP
          	 ::* * : ::.*.*:*. **:**  .: .:*::...*::***  *. *** :***  :*

MG06072.1 	WGTMRHAFWPRCQTEGTITTPDGPVDVKGRCLFVHALQGMKPHHA-~-AASWNFINFQGP
NCU04337.1	WGSMRHVFWPRCVAEGTIATPDGPVDCKGRAMFVHALQGMKPHHA-~-AAKWNFCNFQGP
FG05072.1 	WGSIRHAFWPRCVSEGTITTKEGPIDLKGKALFVYALQGMKPHHA-~-AGRWNFVNFQGP
AN0117.1  	WGSMRHMFWPRCNVTGTITTKEKVHDMTGRGMFSQALQGMKPHHAG1TASRWNFINFQTP
          	**::** *****   ***:* :   * .*: :*  **********. :*. *** *** *

MG06072.1 	THSAVLMEYITPPSYGSTTVSVGAIAKDGEIVVAGCSNHVEHVEIRKDSENDWKEPTKVK
NCU04337.1	NYSAVLMQYTTPPSYGSTVVNVGGIVKDNEIIFAGAEGAVTHVAIKGDTENDWPEPTAIK
FG05072.1 	TYSAIMMEFTSTPSYGSTLVNVGGIVKDGEIIHAGAMSTATHTQVKKDSENEWPEPSEVK
AN0117.1  	SFSAIMMEFTTPPSYGSTVVNVGGIAKDGEIIYAGTTNSATHTEASQDETSDWPEPKSIK
          	..**::*:: :.****** *.**.*.**.**: **  . . *.    *  .:* **. :*

MG06072.1 	LTWSGATKDGKKVDAGIVSEYETRLDRIDVMAEVPGFVKKFAAATAGTKPYIYQ0-----
NCU04337.1	FEWKGTTKDGKQADAVLEGELEDKLDRIDVMAEVPGFVKQIVAGAVGTKPYIYQ0-----
FG05072.1 	YTWSGATKDAKPVEASIEGPLGERVDRVDVMAEVPGFVKTIVAAAAGTKPYIYQ0-----
AN0117.1  	WVWEGKTKDGKTVTAEVDGPLGPKLDRIDVMAEVPGFIKTIAGSVAGARPYIFQ0RNLVP
          	  *.* ***.* . * : .    ::**:*********:* :.....*::***:*  .  .

MG06072.1 	------------------------------------------------------------
NCU04337.1	------------------------------------------------------------
FG05072.1 	------------------------------------------------------------
AN0117.1  	ATLSTYISPQPSLASAPTTPSSLLNIDTGCYIHILPLLRLQVAIFNAFPYFCTPVHRSPW
          	:: ::  :...: :::.::.::  . .:..     .    . :  .: .  .:.   :. 

MG06072.1 	----------~--YMPRKASPTLKLKIGDEPEITETGAMFCEATFITDSTTGEQ------
NCU04337.1	----------~--YAPQKKKLTLKLKLGEE-EISEEGYLFSEATFISA------------
FG05072.1 	----------~--YHP---KLSLKLKIGDE-EIVEEGVMFTEATFIS-------------
AN0117.1  	PRYVTISSPQ0LELLTKLSPVTFKRNAEAPAEQPANTSLFTQHAQRSGMTPALGSEVDPN
          	.   : ::..  .  .     ::* :    .*      :* : :  :  :..  :. ...

MG06072.1 	-----------------------------------~----------------~------~
NCU04337.1	-----------------------------------~----------------~------~
FG05072.1 	-----------------------------------~----------------~------~
AN0117.1  	ARDVTSRLARRGGVYRIKNEIYDEIRIVLKERLAE0VCLVMESGTIPSSERK0LVTTRD0
          	: . ::  :  ..    ...  ..     ..  :.  .   .:.: .::. .   :: . 

MG06072.1 	-------~--------
NCU04337.1	-------~--------
FG05072.1 	-------~--------
AN0117.1  	VVYALKR0MLTTHPFG
          	   : .    :: . .
```
