# Supplementary material for: Patterns of Intron Gain and Loss in Fungi
Source: PLoS Biol. 2004 Nov 30;2(12):e422. doi: 10.1371/journal.pbio.0020422 (PMC532390; doi:10.1371/journal.pbio.0020422)
Supplement: Table S1 — Also available at http://genes.mit.edu/NielsenEtAl/. (4.3 MB ZIP). [file pbio.0020422.st001.zip › NielsenEtAl/html/1071.html]

AN3972.1.NCU04569.1.MG00771.1.FG10203.1


```
 CLUSTAL W (1.82) Multiple Sequence Alignments - Introns Inserted


Sequence 1: MG00771.1	1376 aa
Sequence 2: FG10203.1	1360 aa
Sequence 3: NCU04569.1	1375 aa
Sequence 4: AN3972.1	1274 aa
Alignment Length: 1389 aa
Number Identitical Residues: 669 aa
Alignment Score (without introns) 34627


MG00771.1 	MGSTQEQKKGIRIAIDRGGTFTDCVGNLNGE-DVVIKLLSEDPANYKDAPLEGIRRIMSH
NCU04569.1	MGSSQ-KDRGVRIAIDRGGTFTDCVGNYNGE-DIVIKLLSVDPANYDDAPLEGIRRIMSH
FG10203.1 	MAAAS-QSRGIRIAIDRGGTFTDCVGEHNGK-ETIIKLLSEDPANYKDAPLEGIRRIMSH
AN3972.1  	----MAENCKITISIDRGGTFTDVHAVVPGRPDIILKLLSVDPAHYQDAPTEGVRQILEL
          	      :.  : *:*********  .   *..: ::**** ***:*.*** **:*:*:. 

MG00771.1 	FLKRDIPRGEQLDTSEIESIRMGTTVATNALLERKGEKISLIVTRGFKDCLTIGNQSRPK
NCU04569.1	FLKKEIPRGQPLDTAKIDSIRMGTTVATNALLERKGEKIAMVVTKGFKDCLVIGNQSRPK
FG10203.1 	FLGRDIPRGEALDTSKIDSIRMGTTVATNALLERKGEKIAMVVTKGFKDCLTIGNQSRPK
AN3972.1  	VTGKPHPRGQPLELGPIESLRMGTTVATNALLERKGARSALLTTKGFRDLLRIGDQSRPN
          	.  :  ***: *: . *:*:**************** : :::.*:**:* * **:****:

MG00771.1 	IFDLAIRKPDVLYESVVEIDERVTLEDYAEDPYRTATKAEVKAGTPEARTADLVMGLSGE
NCU04569.1	IFDLAIRKPEVLYSTVVEVEERVTLEDYAEDPERHLTKVDVKAGTTEAKNADLVMGLSGE
FG10203.1 	IFDLAIRKPDVLYEKVVEIDERVTLEDYAEDPERTQTEAEAQVGTKEAEGKTLVRGLSGE
AN3972.1  	IFDLTMARPGMLPEAVVEIDERIVPVHPASDKD-------------CFSGARLVEGVTGE
          	****:: :* :* . ***::**:.  . *.*                     ** *::**

MG00771.1 	TVRILERPDEGKIRKQLQEVYDSGIRCIAVCLMHAYTFPDHETTIGRLAREIGFQHVSLS
NCU04569.1	AVRILQRPEKETIRAKLQEIYDSGIRSIAVCLMHAYTFPDHEALVGEVAREIGFTHISLS
FG10203.1 	TVRILKRAEEDDIRSKLKDVYDQGIRSIAVCLMHGYTYPDHEALIGRVAKDIGFQHISLS
AN3972.1  	KFRVVKELDIEKVRPELERLKEKGYQSLSVALVHSYVYPEHERLIGELAEQMGFS-VTLS
          	 .*:::. :   :* :*: : :.* :.::*.*:*.*.:*:**  :*.:*.::**  ::**

MG00771.1 	SELMPMIKLVSRATSVCADAYLTPAIKKYIAGFQEGFAGGLGSRSVQKTEGSRGARCEFM
NCU04569.1	HELMPMIKLVSRATSVCADAYLTPAIKKYISGFQKGFVGGLGTKGVKQSEGAVGARCEFM
FG10203.1 	HELMPMIKLVSRATSVCADAYLTPAIRKYIDGFQAGFEGGLGTRSVKEETGAKGARCEFM
AN3972.1  	SKLQPMIKIVPRGMSAAADAYLTPVIKTYIDSISSSFEGGLAN--------QHSCRFEFM
          	 :* ****:*.*. *..*******.*:.** .:. .* ***..          ..* ***

MG00771.1 	QSDGGLVDVEKFTGLKAILSGPAGGVVGYAITSYDDNTRIPVIGFDM~GGTSTDVSRYGE
NCU04569.1	QSDGGLVDVDKFTGLKAILSGPAGGVVGYAITSYDENTKIPVIGFDM~GGTSTDVSRYGE
FG10203.1 	QSDGGLVDVEKFTGLKAILSGPAGGVVGYAITSYDEETKTPVIGFDM0GGTSTDVSRYGE
AN3972.1  	QSDGGLVDFRKFSGLKAILSGPAAGVVGFAATSWDAEEKTPVIGFDM~GGTSTDVSRF-D
          	********. **:**********.****:* **:* : : ******* *********: :

MG00771.1 	GRYDHTFETTTAGVTIQSPQLDINTVAAGGGSRLFFKNGLFVVGPESAGAHPGPACYRKG
NCU04569.1	GRYDHTFETTTAGVTIQSPQLDINTVAAGGGSILFFRNGLFVVGPESASAHPGPACYRKG
FG10203.1 	GRYEHVFETTTAGVTIQSPQLDINTVAAGGGSRLFFRNGLFVVGPESAGAHPGPACYRKG
AN3972.1  	GHLEHVFGSKLAGVQIQSPQLDINTVAAGGGSILNWRNGLFYVGPESASAHPGPACYRKG
          	*: :*.* :. *** ***************** * ::**** ******.***********

MG00771.1 	GPATVTDANLFLGRLLPEFFPKIFGKNEDEGLDVEASRKVLQELTDEVNKTS----EKQM
NCU04569.1	GPATVTDANLFLGRLLPEFFPKIFGKNEDEGLDPEASRIKIQELADQIKAET----GKEM
FG10203.1 	GPATVTDANLVLGRLLPEFFPKIFGENEDEGLDVEASRKVLQELADQVNRES----DKNL
AN3972.1  	GPLTVTDANLFLGRLLPEFFPHIFGENEDQPLDLEVTTKKFKELTDTVNAERRQKGESEY
          	** *******.**********:***:***: ** *.:   ::**:* ::    ... .: 

MG00771.1 	TIDEVAYGFLTVANESMTRPIRSITEAKGHDSSKHRLASFGGAGGQHAVAIAEALGIKQV
NCU04569.1	DLDEVAYGFLTVANEAMTRPIRSITEAKGHDTSKHRLATFGGAGGQHAVAIAESLGIKQI
FG10203.1 	TADEVAYGFLTVANETMARPIRSITEAKGHDSSKHRLATFGGAGGQHAVAIAESLGIQQI
AN3972.1  	TPEEVALGFLKVADESMARPIRNLTEARGFETATHHLASFGGAGGQHACPVAASLGISRI
          	  :*** ***.**:*:*:****.:***:*.:::.*:**:********* .:* :***.::

MG00771.1 	LVHRYSSVLSAYGMALADVVDERQEPESAVWADDGE-VVNGLKTKMEKLKDQSRQALRDQ
NCU04569.1	LIHRYSSVLSAYGMALADVVDERQEPDSSVWKADDQSVIQGLKDKMEALKEKSRQALRDQ
FG10203.1 	LVHRYSSVLSAYGMALADVVDERQEPDSLVWKDDDK-TVSELKKKMEKLKDQSQKSLNDQ
AN3972.1  	IIHKFSSVLSAYGLALAEVVKESQEPLSTQYESSKP----ELKKKLAEMTEAAVEDMKEQ
          	::*::********:***:**.* *** *  :  .       ** *:  :.: : : :.:*

MG00771.1 	GFEDKEIVFEEYLNMRYRGTESALMIVKPSEEEVAKTYNGKDWAFGEAFVRQHRYEFGFT
NCU04569.1	GFEDDQIVFEEYLNMRYRGTESTLMIIKPTAEEAEKHYNGNEWDFASAFVRHHRYEFGFT
FG10203.1 	GFQESEIAFEEYLNMRYRGTESALMIVRPTAEEAKEHFDGKEWDFGQAFVKQHRYEFGFT
AN3972.1  	GFSSDQVRHERYLNLRYDGSDTSLMILEPE--------DGS--DFIEQFRERHRREFGFN
          	**...:: .*.***:** *::::***:.*         :*.   * . * .:** ****.

MG00771.1 	LDDRDIIIDDVRVRGIGKSFSYDEKTVDEQLKTVQRNEVSTDSQKKHSDAKVYFDG--GR
NCU04569.1	LEERDIVIDDVRVRGIGKSFRYEEKSVDEQLKTIQKKDVDV--KNTHSTAKVYFEN--GR
FG10203.1 	LDERDIIIDDVRVRGIGKSFRHQDDTVDKQLKDLKQQEVSD--KKKLNSQQVYFEG--GR
AN3972.1  	SD-RPVLVDDIRVRTIAASKVRDEKSPLVQLREAKIRDITS---SPDLITKTFFDGQKGR
          	 : * :::**:*** *. *   ::.:   **:  : .::     .     :.:*:...**

MG00771.1 	LDTPIFKLEDMAVGDKIPGPAMLADGTQTIVVAPKTAALILQTHVVIDIDEDQRKTD~AD
NCU04569.1	MDTPIYKLGDLSVGTVIKGPAMLADGTQTIVVTPKSTALVLETHVVVDIEETDKNKD~-Q
FG10203.1 	KETPVFKLEDLQVGDSIPGPAMLADGTQTIVVTPKATAIILKTHVVINLEKQGSKIE2SS
AN3972.1  	VDTPVFKLDNIEKNSRIHGPAIIIDNTQTIVVVPNAVANVLETCILIDLKETRSTEN~KP
          	 :**::** ::  .  * ***:: *.******.*::.* :*:* :::::.:   . :   

MG00771.1 	VGEGQREVDPIMLSVFGHRFMAIAEQMGRALQKTSVSTNVKERLDFSCAIFDSTGGLVAN
NCU04569.1	KGDGEREVDPIMLSIFGHRFMAIAEQMGMALQKTSVSTNVKERLDFSCAIFDANGGLVAN
FG10203.1 	KASGDREVDPIMLSIFGHRFMAIAEQMGRALQKTSVSTNVKERLDFSCAIFDATGGLVAN
AN3972.1  	TSG----IDTIKLSIFGHRFMSIAEQMGRTLQKTAVSTNIKERLDFSCALFSPDGGLVAN
          	 .     :*.* **:******:****** :****:****:*********:*.. ******

MG00771.1 	APHLPVHLG~SMSTCVKRQAEIWKGKLKKGDVIATNHPSYGGTHLPDVT~LIMPAFNAKG
NCU04569.1	APHLPVHLG1SMSTCVRRQAEIWKGKLRKGDVLMTNHPSYGGTHLPDVT~LIMPAFNEAG
FG10203.1 	APHLPVHLG~SMSTCVRRQAEIWKGKLEKGDVIISNHPSYGGTHLPDVT~LLMPAFDEKG
AN3972.1  	APHVPVHLG~SMQFAVRYQHKKWLGNLKDGDVLVANHPSCGGTHLPDIT0VITPVFDKPG
          	***:***** **. .*: * : * *:*..***: :**** *******:* :: *.*:  *

MG00771.1 	-DKILFYAASRAHHADIGGITAGSMPPHSRELYQEGAAIKSEKFVSEGKFDEERVVELFY
NCU04569.1	-DKILFYAASRAHHADIGGITAGSMPPHSRELYQEGAAVKSEKLVSEGKFNEDRVIELFH
FG10203.1 	-ENILFYAASRAHHADIGGISAGSMPPHSRELYQEGASIRSEKLVSGGKFNEKRVVELFY
AN3972.1  	GSEIMFYVASRGHHADIGGILPGSMPPKSTELWQEGAAIEGDKVVSNGKFDEERMVELLV
          	..:*:**.***.******** .*****:* **:****::..:*.** ***:*.*::**: 

MG00771.1 	REPAKQPGCSGTRCLADNINDLRAQVSANQKGISLIETLIDEYGEQTVDFYMVQIQNNAE
NCU04569.1	KEPAQYPGCSGTRCLADNMSDLRAQVSANQKGISLIETLIAEYGEDTVQFYMVAIQNNAE
FG10203.1 	EEPAKYPGCSGTRCLADNINDLRAQVSANQKGISLIEALIAEYGEETVQFYMVHIQNNAE
AN3972.1  	KKPAQYPGCSGARCITDNISDLKAQIAANTRGITLIQALFAEYGVQTVQKYMYAIQETAE
          	.:**: *****:**::**:.**:**::** :**:**::*: *** :**: **  **:.**

MG00771.1 	QCVRRLLKEVSARFEGKDLSSEDFMDDGSPIRLKIKIDAEKGEADFDFEGTGPEVYGNIN
NCU04569.1	QQVRNLLRTVHKRFQGRDLSAIDYMDDGSPIQLKVTIDPEAGEAVFDFAGTGPEVYANIN
FG10203.1 	QCVRRLLKGVYKRFEGKDLSAVDFMDDGSPIRLKIRIDAEKGEAEFDFSGTGPEVYGNIN
AN3972.1  	TAVRNLLKDLYHRFEGRPLEAVDYMDDGTPIKLKVTINGDDGSAVFDFEGTGPQVYGAWN
          	  **.**: :  **:*: *.: *:****:**:**: *: : *.* *** ****:**.  *

MG00771.1 	APEAVTYS2AIIYCLRCLISQDIPLNQGCLKPIHVKIPPKSLLSPSDHAAVVGGNVLTSQ
NCU04569.1	APEAISYS~AIIYTLRCMISEDIPLNQGCLKPVTVKIPPKSLLSPSDNAAVVGGNVLTSQ
FG10203.1 	APQAITFS~AIIYCLRCLISDDIPLNQGCLKPIHVKIPPKSILSPSPGAAVVGGNVLTSQ
AN3972.1  	APIAITHS~AIIYCLRCMINADMPLNQGCLAPIDIKVPPSCLLSPTKNAAVVGGNVVTSQ
          	** *::.* **** ***:*. *:******* *: :*:**..:***:  ********:***

MG00771.1 	RVTDVIFRAFEACAASQGCCNNLTFGFGSNQAGQD--EVKGFGYYETISGGSGAGPTWEG
NCU04569.1	RITDVIFKAFQACAASQGCCNNLTFGFGGNVAGEK--EVKGFGYYETIAGGSGAGPTWEG
FG10203.1 	RITDVIFKAFQACAASQGCCNNLTFGFGGNQDGAE--AVKGFGYYETIAGGSGAGSDWEG
AN3972.1  	RVTDVVFKAFRACAASQGCCNNLTFGKNAKKDPENGNEIPGFGYYETIAGGSGAGPTWDG
          	*:***:*:**.*************** ..:    ... : ********:******. *:*

MG00771.1 	TSGVHVHMTNTRITDSEIFERRYPVLLREFSIRPDSGGAGQHRGGDGVIRDIEFRIPLQV
NCU04569.1	TDGVHVHMTNTRITDSEIFERRYPVLLREFSIRKGSGGKGKHRGGDGVVRDIEFRLPLQV
FG10203.1 	TSGVHCHMTNTRITDSEIFERRYPVLLREFSIRSGSGGQGQHRGGDGVIRDIEFRIPLQV
AN3972.1  	ESGIHVHMTNTRITDPEILEKRYPTLLRQFTLRSGSGGKGQHPGGEGVIREIEFLTPMDC
          	 .*:* *********.**:*:***.***:*::* .*** *:* **:**:*:***  *:: 

MG00771.1 	SILSERRVYKPYGMAGGESAACGLNLWVRKVAKASWETSLKQIQNGGSGREQEEEEKQKQ
NCU04569.1	SILSERRVYRPYGLAGGEDAQCGLNLWVRKVKKARWEDTLRKIQNGEQDQQQEEEEKKKK
FG10203.1 	SILSERRVYRPYGLNGGGDGECGLNLWVRKVEKANWEASLKQFHT-------------KD
AN3972.1  	SILSERRVHRPYGLEGGENAEPGMNLWITK----------------------------DK
          	********::***: ** ..  *:***: *                            ..

MG00771.1 	QGE--QIEYEERYINLGAKNSAPMKAGDRIIINTPGGGGWGPVGTEKVSNTERDPEENWK
NCU04569.1	EVGGEAEGYEERVINMGAKNSAPMKAGDRIIICTPGGGGWGKSGEERELDETRDPMGAWK
FG10203.1 	DAG--EVEYEERHVNMGAKNTAAMKAGDRIIICTPGGGAWGAEGAESVAKKKVDHTEAWR
AN3972.1  	DTG------EDHTVNIGGKNTIHVETHDRIVIMTPGGGGWGK------------------
          	:        *:: :*:*.**:  ::: ***:* *****.**                   

MG00771.1 	KGSHAAREEMALQA
NCU04569.1	MGSHAARMDMALQA
FG10203.1 	KGSGSARDETALQA
AN3972.1  	--------------
          	
```
