# Supplementary material for: Patterns of Intron Gain and Loss in Fungi
Source: PLoS Biol. 2004 Nov 30;2(12):e422. doi: 10.1371/journal.pbio.0020422 (PMC532390; doi:10.1371/journal.pbio.0020422)
Supplement: Table S1 — Also available at http://genes.mit.edu/NielsenEtAl/. (4.3 MB ZIP). [file pbio.0020422.st001.zip › NielsenEtAl/html/1072.html]

AN2914.1.NCU08162.1.MG09712.1.FG03694.1


```
 CLUSTAL W (1.82) Multiple Sequence Alignments - Introns Inserted


Sequence 1: NCU08162.1	466 aa
Sequence 2: MG09712.1	468 aa
Sequence 3: FG03694.1	454 aa
Sequence 4: AN2914.1	491 aa
Alignment Length: 498 aa
Number Identitical Residues: 253 aa
Alignment Score (without introns) 12436


MG09712.1 	MAEL---PSHEKKANMLWGGRFTG~GLDPLMVAYNESIYFDKNLYRQDIAGSIAFARANS
NCU08162.1	MSG-----QAKPTENMLWGGRFTG1GLDPLMVQYNESIHFDRALFAQDITGSIAFARANA
FG03694.1 	MAANNTNNAGAPAEGMLWGGRFTG1GIDPLMHQYNASISYDKHLYKEDILGSIAFARANA
AN2914.1  	MSAP------KVAENMLWGGRFTQ1GLDPLMVQYNESLPYDRILWKQDIAGSIAFARANT
          	*:            .********  *:****  ** *: :*: *: :** *********:

MG09712.1 	KAGVITQAEFEQIEKGLLEVQKEWEAGTFKIVPGADE2D~IHTANERRLGEIIGKDVAGK
NCU08162.1	KAGIITQDEFNKLEQGLLAVKKEWEDGTFKIVPGVDE~D~IHTANERRLGEIIGKDVAGK
FG03694.1 	KSGIISNDEFTEIERGLREVMKEWEAGTFTIMP-NDE~D0IHTANERRLSEIIGKEIGGK
AN2914.1  	KSGILSAHEFSEIERGFKQIAEEWSTNTFVVKP-NDE~D~IHTANERRLSEIIGKEIGGK
          	*:*:::  ** ::*:*:  : :**. .** : *  ** * *********.*****::.**

MG09712.1 	LHTGRSRNEQVVTDMRMWLRDELRKIESYLVSFLTVTAARAEREI~DLVMPGYTHLQRAQ
NCU08162.1	LHTGRSRNEQVATDMRMWLRDELRKIEKHLQDFLNVTAARAEQEI~DVIMPGYTHLQRAQ
FG03694.1 	LHTGRSRNEQVVTDMRMWLRERIREIESYLVAFLQVIAGRAEADI2DHIMPGYTHLQLAM
AN2914.1  	LHTGRSRNEQIATDLRLWLRDELRKLDAFLSDLIKVSIARAESEI~DYIMPGYTHLQKAQ
          	**********:.**:*:***:.:*::: .*  :: *  .*** :* * :******** * 

MG09712.1 	PIRWSHWMLSYGLVFASDLERLREVIKRVNRS~PLGCGALAGNPFGIDREMMAKELGFDG
NCU08162.1	PVRWSHWMLSYGFAFASDLERLREVIKRVNRS~ALGCGALAGNPFGIDREMMSKELGFDG
FG03694.1 	PVKFSQWLLSYGFSFASDLERLREVLKRVNRS2PLGAGALAGNAFGIDRQMMAKELGFEG
AN2914.1  	PVRWSHWLLSHATAFADELKRLREVTKRVNRS~PLGTGALAGNPFHIDREAMAKELGFEG
          	*:::*:*:**:.  **.:*:***** ****** .** ******.* ***: *:*****:*

MG09712.1 	LMWNSMGAVADRDFVAETLQWGAMLMQHISRWAEDLIIYSTAEFGF~VRLADAYSTGSSL
NCU08162.1	LLWNSMGAVADRDFVAETLQWGSMLMQHISRWSEDLIIYSTSEFGF~VRLADAYSTGSSL
FG03694.1 	IMWNSMGAVADRDFVTEFLSWGTMFMGHISRFSEDLIIYSTAEFGF0K------------
AN2914.1  	LLYNSMNAVGDRDFAMETMQWGSSFMLKISRWAEDLIIYSSLEFGF~VRLSDAYSTGSSL
          	:::***.**.****. * :.**: :* :***::*******: ****    :.: ::.:: 

MG09712.1 	MPQKKNPD~SLELLRGKSGRAFGHMAGFMMVQKGLPSTYNKDLQESIEPMLDHVRTVSDS
NCU08162.1	MPQKKNPD~SLELLRGKAGRAFGHMAGFMMTQKGIPSTYNKDLQESWEPMLDHVKTVSDS
FG03694.1 	----KNPD~GLELLRGKAGRAFGHFAGVYVATKGIPSTYNKDLQESWEPMLDHVKTVSDS
AN2914.1  	MPQKKNAD1SLELLRGKAGRAFGHMAGLMCTIKGLPTTYNKDLQESVEPLLDHIKTVGDS
          	 ...**.* .*******:******:**.  . **:*:********* **:***::**.**

MG09712.1 	IQIAEGVLATLEARGDKMQAALDPFMLATDVADYLVRKGVPFRETHHISGRCVALSEQTG
NCU08162.1	IQIANGVLATLSIQPEKMKESLDPFMLATDLADYLVRKGVPFRETHHISGRCVALSEQTG
FG03694.1 	VQIANGVLSTLKLRPDRMRASLNPFLLATDVADALVRLGVPFRETHHISGRVVAKSEELG
AN2914.1  	IQIATGVLSTLTTIPEKMAAALAPEMLATEFADYLVRKGVPFREGHHISGRVVQLAEKHG
          	:*** ***:**    ::*  :* * :***:.** *** ****** ****** *  :*: *

MG09712.1 	VPMDKLTYEQLREIDERFEPDIAESFDYDKSVEMRSAKGGTCKEAVMEQVKVLRKML~E-
NCU08162.1	IPMDKLSYEQLKGIDARFEEDISKVFDYEKSVEMRSAKGGTSKACVQEQIQVLKSMI~A-
FG03694.1 	IPMDQLSVEQLQAIDSRFPDNIKDVFNYEASVESRNAQGGTSRAGVLEQIEVLKGML~N-
AN2914.1  	VPMDQLSLEQLKSVDDRFGDDIQECLDYERAVELKDAIGGCSKRAVLEQTSVLKSIL2KL
          	:***:*: ***: :* **  :* . ::*: :** :.* ** .:  * ** .**: ::   

MG09712.1 	--------------------------
NCU08162.1	--------------------------
FG03694.1 	--------------------------
AN2914.1  	RSSQVQEAFALDHEQVCNENHAVKGI
          	 ::. ..: : . .. .... : ..
```
