# Supplementary material for: Patterns of Intron Gain and Loss in Fungi
Source: PLoS Biol. 2004 Nov 30;2(12):e422. doi: 10.1371/journal.pbio.0020422 (PMC532390; doi:10.1371/journal.pbio.0020422)
Supplement: Table S1 — Also available at http://genes.mit.edu/NielsenEtAl/. (4.3 MB ZIP). [file pbio.0020422.st001.zip › NielsenEtAl/html/1073.html]

AN5287.1.NCU01181.1.MG08661.1.FG05140.1


```
 CLUSTAL W (1.82) Multiple Sequence Alignments - Introns Inserted


Sequence 1: NCU01181.1	451 aa
Sequence 2: MG08661.1	452 aa
Sequence 3: FG05140.1	452 aa
Sequence 4: AN5287.1	326 aa
Alignment Length: 452 aa
Number Identitical Residues: 132 aa
Alignment Score (without introns) 9603


MG08661.1 	MADKIPAI0CRDRVSPRAKQTLDIVTKFVKEECLP~ADPVLEAQVGQGSARWDAHPPIVE
NCU01181.1	MSTQIPIA0VRNRVSERAKKTLDVVARFVEEDCIP2ADTVYEAQIGVGDARWEAHPQILE
FG05140.1 	MSARIPAI0AANRVSDAAKKQLDLVAKFVEEECIP2ADPVVEALAGEGDARWEGHPSIIE
AN5287.1  	--------~--------------------------~------------------------
          	                                                            

MG08661.1 	DLKRKARALGLWNMFLPAGHYKESPGFTNLEYGLMAEQLGRSRVASEAVNCAAPDTGNME
NCU01181.1	DLKAKAKSLGLWNMFLPKGHYKESPGFTNLEYGLMAEWLGKSRVASEAVNCSAPDTGNME
FG05140.1 	DLKEKARKLGLWNMFLPKGHYKESPGWTNLEYGLMAEWLGRSHVASEACNCAAPDTGNME
AN5287.1  	--------------------------------------MGKVYWAGQTMNCHAPETGNIE
          	                                      :*:   *.:: ** **:***:*

MG08661.1 	VLAKYGNEEQKARWLRPLMEGEIRSAFLMTEPQIASSDAKNIEMDIRREGNEYVLNGQ0K
NCU01181.1	VLAKYGNEEQKAKWLKPLMDGKIRSAFLMTEPQVASSDARNIEMKITKDGDHYVLNGQ~K
FG05140.1 	VLAKYGNDAQKAQWLKPLMDGKIRSAFLMTEPQVASSDATNIELSIRREGNEYVLNGQ~K
AN5287.1  	LLAKYCNEQQKEQWLKPLLRGEFASAYSMTEPDVASSDATKIAISIRREGDEYMINGR~K
          	:**** *: ** :**:**: *:: **: ****::***** :* :.* ::*:.*::**: *

MG08661.1 	WWSSGAGDPRCAIYIVMGKSDRNNKDPYRQQSVVLVPADTPGITIHRMLSVYGYDDAPHG
NCU01181.1	WWSSGAGDPRCKVYIVMGKSDPNNKDPYRQQSVIIVPSDTKGITIERMLSVYGYDDAPHG
FG05140.1 	WWSSGAGDPRCKIYIVMGKTDPDNKDPYRQQSVILVPAETPGITINRMLKVYGFDDAPHG
AN5287.1  	LFAT-KWNPEVKLYILMGCTDPNNPNPRRRHSMLLIPSDTPGFRMRQNLSIMGHDCAPES
          	 :::   :*.  :**:** :* :* :* *::*::::*::* *: :.: *.: *.* **..

MG08661.1 	HGHITFSNVRVPVANMVLGEGRGFEIIQGRLGPGRIHHCMRAIGA0AERALDWMLMRIND
NCU01181.1	HGHITFYNVRVPAANLVLGEGRGFEIIQGRLGPGRIHHAMRTIGA~AERALEWMLMRIND
FG05140.1 	HGHLTFNNVRVPASNLVLGEGRGFEIIQGRLGPGRIHHAMRSIGA0AERALDWMLLRVND
AN5287.1  	HGEYVYDNVRIPVSNVVLGEGGAFEIAQGRLGPGRIHHCMRLIGQ~AELAFDYALVRATD
          	**. .: ***:*.:*:***** .*** ***********.** **  ** *::: *:* .*

MG08661.1 	PRKTTFGKQLREHGVILEWVAKSRLEIDSARLVVLNAAIKMDDLGPKAALAEIAQAKVLV
NCU01181.1	PKKTPFGKQLREHGVILEWVAKSRIDIDAARLVVLNAAIKMDEQGPKAALTEIAQAKVLV
FG05140.1 	ESKKPFGKLLREHGVIIEWIAKSRIEIDAARLIVLNAAIKMDDLGPKKALKEIAEAKVLI
AN5287.1  	ERKKPRGKLIWQFDSNIERIAQMRLKLDALRLVVFNAADTMDALGNKSGRYVIAQSKILV
          	  *.. ** : :..  :* :*: *:.:*: **:*:*** .**  * * .   **::*:*:

MG08661.1 	PSMALTVIDRAVQAFGGA~GVSQDTPLANMWAQIRTLRLADGPDEVHLQQMGRNENRRGK
NCU01181.1	PQTALTVIDRAVQSFGGA~GVSQDTPLANMWAQIRTLRLADGPDEVHLQQMGRNENKRGK
FG05140.1 	PQTALTVIDRAIQAYGGA~GVSQDTPLAYMWAGIRTLRLADGPDEVHLQQMGRNENKRSA
AN5287.1  	PIAVAKIVDECMQLYGGR0-VGRDQ-----------LKTAGSVVARHQQYY---------
          	*  . .::*..:* :**   *.:*            *: *..    * *           

MG08661.1 	EAAEEIERQRKRTEELLKQWKVERSEPGTGITRKSKL
NCU01181.1	AVTDKINAQKAKAEELRKKYGAKRTEIGSNIK-HSKL
FG05140.1 	EATATIKMQRAKTEELLKAYGVERLQPGARIQHKAKL
AN5287.1  	KVALQLKKQFGLDEELGDDCTWEYIEP--------KL
          	 .:  :: *    *** .    :  :         **
```
