# Supplementary material for: Patterns of Intron Gain and Loss in Fungi
Source: PLoS Biol. 2004 Nov 30;2(12):e422. doi: 10.1371/journal.pbio.0020422 (PMC532390; doi:10.1371/journal.pbio.0020422)
Supplement: Table S1 — Also available at http://genes.mit.edu/NielsenEtAl/. (4.3 MB ZIP). [file pbio.0020422.st001.zip › NielsenEtAl/html/1074.html]

AN0347.1.NCU01523.1.MG01079.1.FG04327.1


```
 CLUSTAL W (1.82) Multiple Sequence Alignments - Introns Inserted


Sequence 1: MG01079.1	211 aa
Sequence 2: FG04327.1	199 aa
Sequence 3: AN0347.1	237 aa
Sequence 4: NCU01523.1	214 aa
Alignment Length: 242 aa
Number Identitical Residues: 155 aa
Alignment Score (without introns) 5805


MG01079.1 	MA-DLGG~FLPN---~---AVVLIGDSGVGKSNLLSRFTRNEFNLDSKSTIGVEFATRSI
NCU01523.1	MANDEYD0IPLP---~----VVLIGDSGVGKSNLLSRFTRNEFNLDSKSTIGVEFATRSI
FG04327.1 	-------~-------~------MIGDSGVGKSNLLSRFTRNEFNLDSKSTIGVEFATRSI
AN0347.1  	MANDEYD~VSPRPLT0FLFKVVLIGDSGTGKSNLLSRFTRNEFNLDSKSTIGVEFATRSI
          	 :..  .     . :       :*****.*******************************

MG01079.1 	QVDSKTIKAQIWDTAGQERYRAITSAYYRGAVGALLVYDISKHQTYENVTRWLKELRDHA
NCU01523.1	QVDSKTIKAQIWDTAGQERYRAITSAYYRGAVGALLVYDISKGVTFENVNRWLKELRDHA
FG04327.1 	QVDSKTIKAQIWDTAGQERYRAITSAYYRGAVGALLVYDISKHQTYENVTRWLKELRDHA
AN0347.1  	QVDSKTIKAQIWDTAGQERYRAITSAYYRGAVGALLVYDISKHQTYDNVNRWLKELRDHA
          	******************************************  *::**.**********

MG01079.1 	DANIVIMLVGNKSDLRHLRAVPTEEAKAFAS1ENHLSFIETSAFDASNVELAFQNILT--
NCU01523.1	DQNIVIMLVGNKSDLRHLRAVPTEDAKKFAE1ENHLSFIETSALDATNVELAFQNILT--
FG04327.1 	DANIVIMLVGNKSDLRHLRAVPTEEAKSFAS1ENHLSFIETSALDASNVELAFQNILT--
AN0347.1  	DSNIVIMLVGNKSDLRHLRAVPTEEAKQFAS1ENNLSFIETSALDASNVELAFQNILTGI
          	* **********************:** **. **:********:**:***********. 

MG01079.1 	------------------E1IYRIVSSKALDSGDTPS---MPQ-TGTNIALSNPTEDSAA
NCU01523.1	------------------E1IYKIVSTKNFDNGPSGSNEGHQNLSGQSISLSQTANDPQA
FG04327.1 	------------------E1IYRIVSSKALDSGDSAQ---ATIGAGTNISLSKPADDDAA
AN0347.1  	APVGISKRRSLTAILFFVE~IYRIVSSKALE-GESGG---ASVGERRQIIDIEKTQDTEN
          	:. . :.  : ::     * **:***:* :: * :            .*   : ::*   

MG01079.1 	KNGKCC
NCU01523.1	KSG-CC
FG04327.1 	KGGKCC
AN0347.1  	KGG-CC
          	*.* **
```
