# Supplementary material for: Patterns of Intron Gain and Loss in Fungi
Source: PLoS Biol. 2004 Nov 30;2(12):e422. doi: 10.1371/journal.pbio.0020422 (PMC532390; doi:10.1371/journal.pbio.0020422)
Supplement: Table S1 — Also available at http://genes.mit.edu/NielsenEtAl/. (4.3 MB ZIP). [file pbio.0020422.st001.zip › NielsenEtAl/html/1075.html]

AN8664.1.NCU02124.1.MG02570.1.FG08780.1


```
 CLUSTAL W (1.82) Multiple Sequence Alignments - Introns Inserted


Sequence 1: NCU02124.1	255 aa
Sequence 2: MG02570.1	258 aa
Sequence 3: FG08780.1	255 aa
Sequence 4: AN8664.1	261 aa
Alignment Length: 271 aa
Number Identitical Residues: 102 aa
Alignment Score (without introns) 6015


MG02570.1 	MSTMEATAGHSKAC~CN~VPPVVESGYEKKGTYEEVGGYKTY1VTGPQDATKGIIAIY-~
NCU02124.1	MSTMQACHGHNEAC2CN~IPPVVTSGYSPKGSFTEYDGLKTY~VTGPDDATKGIVVIY-~
FG08780.1 	MSTMPASHGHSEAC~CN~IPPVVTKGYEAKGTYKDIGGYKTY1VTGPVDAKKAIVVIY-~
AN8664.1  	MS------GVSKAC~CS2IPPIVAKGYQPKGEYKTINGLKTY1VTGPSDATKAILVVYG1
          	**      * .:** *. :**:* .**. ** :   .* *** **** **.*.*:.:*. 

MG02570.1 	--------DIFGYFDQTLQGMDILATSDASQKYRVFMPDWFKGNPCPIEW~YPPNTEEKQ
NCU02124.1	--------DIFGYFDQTIQGADILATSDDT-KYKVFMPDWFKGEPCPIEW~YPPDTDEKK
FG08780.1 	--------DIFGYFEQTLQGADILAFSDAHQKYKVFIPDWFKGGPCPIEI2YPPDNDDKK
AN8664.1  	PLNLLTLPDIFGFFPQTLQGADILATSSKQ-KYRIFMPDFFEGEPADITW~FPPSNEDQK
          	. .  : .****:* **:** **** *.   **::*:**:*:* *. *   :**..::::

MG02570.1 	QKVGNWFK-DWNPAETAAKVPDYVKAVREKNPG---IKSWGIIG0FCWGGKIVCLTTSSD
NCU02124.1	KNLGAFFS-KNPPHGVAEKLPAFVKTLSAKHPN---IKSWAIIG0YCWGGKVVSLITKSE
FG08780.1 	KQLGEFFE-TYPPPKVAGQVPDYVKAVKEQDSS---IEKFGILG0YCWGGKVVALSVKAD
AN8664.1  	NKLGNFFQTKAAPPKTLSKIPGVVSEANSYAPSGGAFESWSILG~YCWGGKITVLSSGSE
          	:::* :*.:   *  .  ::*  *.      ....:::.:.*:* :*****:. *   ::

MG02570.1 	NNPFAAGASIHPAMVDAADAKNIKVPLIVLASKDEAAKDVSAFEESLPSSVPKHIETFGD
NCU02124.1	NNPFSIGAECHPAMVDPEEAKGIKVPLILLASKEEPEDKVKEFEQNL--SVPKHVETFKD
FG08780.1 	SNPFSIAAQIHPAMVDASDAEGLSVPTMLLASMEEPEEEVKKFEDNL--KVAKHVETFKD
AN8664.1  	NKTFKAAVQCHPAMLDPNDAKGVNIPMALLASKDEKPDDVSQFGANL--KVDHYVETFPT
          	.:.*  ... ****:*. :*:.:.:*  :*** :*  ..*. *  .*  .* :::***  

MG02570.1 	QVHGWMAARADLKDARVKEEYTRGYKTVIEFFGKNWN
NCU02124.1	QVHGWMAARGDLKDERVKSEYVRGYKTVLEFFGKNWK
FG08780.1 	QIHGWMAARADLNDSRVKEEYERGYKTVVEFFGKNF-
AN8664.1  	QIHGWMAARSQLEDEQVRKEYERGYQTALDFLAKHA-
          	*:*******.:*:* :*:.** ***:*.::*:.*:
```
