# Supplementary material for: Patterns of Intron Gain and Loss in Fungi
Source: PLoS Biol. 2004 Nov 30;2(12):e422. doi: 10.1371/journal.pbio.0020422 (PMC532390; doi:10.1371/journal.pbio.0020422)
Supplement: Table S1 — Also available at http://genes.mit.edu/NielsenEtAl/. (4.3 MB ZIP). [file pbio.0020422.st001.zip › NielsenEtAl/html/1076.html]

AN5670.1.NCU02280.1.MG00878.1.FG06337.1


```
 CLUSTAL W (1.82) Multiple Sequence Alignments - Introns Inserted


Sequence 1: AN5670.1	197 aa
Sequence 2: FG06337.1	207 aa
Sequence 3: NCU02280.1	200 aa
Sequence 4: MG00878.1	204 aa
Alignment Length: 214 aa
Number Identitical Residues: 51 aa
Alignment Score (without introns) 3046


MG00878.1 	--------MSPQPHEGEDYRPQDAIKTGLRGTFIYGAFGLFGAATLAAVQRKRVGVLAPI
NCU02280.1	--------MAPQGDD-TVFQPKDAIKSGVSGALFSGGAGLLMASLRTSMKKNNVGSMHVF
FG06337.1 	MEHPSSANLPAHKKPDTYFKPHDVLDDTAKAAVVGGLSGLFLSSVKNAMAKHNVGILSVF
AN5670.1  	--------MGATG-EDHHYHPQDTIARTMKTTGLTGSVGLFASAVQNTLARQNVGPWGVF
          	        : .    .  ::*:*.:      : . *  **: ::   :: ::.**    :

MG00878.1 	TKYGALVSATA1FGGGMYDFTRTATSNLRQKNDHVSEAVGGLLAGAIFGLAV~PG1PTRL
NCU02280.1	THGGGTIISFT1LAGGIYRFAQQASANLREKEDGWNHAIGAFLGGSVMGLRS1--~-LRF
FG06337.1 	TRGSAMIGIGA1AAPAAYVFASRTSMNLREKDDSFSAALGGFALGAVLGLPT1--~-KRM
AN5670.1  	TRSGATVGILA1AMGGTYEFVKTSSANLREKEDHWNVALGGFFSGAILGLRA1--~-RTF
          	*: .. :   :    . * *.  :: ***:*:*  . *:*.:  *:::**         :

MG00878.1 	PVVVGFAAGSAVTLGAYAYTGKSLRGWGKKDDGKDDFERKEELRKNRRRPIEETIAELGE
NCU02280.1	PVILGFGAMAGSVVGAFAFSG-GLTGWGR-DPNVDEFERKEAMRLNRRRPVEETLAEVGE
FG06337.1 	PIVMGLGGGLAAFQGMFHYLGGRYDSFKR-EG--DEFERKEIVRRSTRLPIEQTISEIGE
AN5670.1  	PALLGYGAALATAMGGFEYTGGSLFGRKR-DPNVDEFERREKLRTQWRTPGEQTLAELGE
          	* ::* ..  .   * : : *    .  : : . *:***:* :* . * * *:*::*:**

MG00878.1 	GRG1IHPPGYEERRRERLKEKYGVEINPVSADPNAA--
NCU02280.1	GRG1IYPPGYQERRRQRLLEKYGVEVKPVSADPNVASA
FG06337.1 	GRG1IRPPGYDERRAERLSQKYGVEINPIKATVEGSQ-
AN5670.1  	GRG1IYGPGYAERRRERIKEAYGIDV-PVSPPAAS---
          	*** *  *** *** :*: : **::: *:..
```
