# Supplementary material for: Patterns of Intron Gain and Loss in Fungi
Source: PLoS Biol. 2004 Nov 30;2(12):e422. doi: 10.1371/journal.pbio.0020422 (PMC532390; doi:10.1371/journal.pbio.0020422)
Supplement: Table S1 — Also available at http://genes.mit.edu/NielsenEtAl/. (4.3 MB ZIP). [file pbio.0020422.st001.zip › NielsenEtAl/html/1077.html]

AN6689.1.NCU06968.1.MG08006.1.FG04983.1


```
 CLUSTAL W (1.82) Multiple Sequence Alignments - Introns Inserted


Sequence 1: MG08006.1	280 aa
Sequence 2: FG04983.1	284 aa
Sequence 3: NCU06968.1	286 aa
Sequence 4: AN6689.1	280 aa
Alignment Length: 291 aa
Number Identitical Residues: 109 aa
Alignment Score (without introns) 5911


MG08006.1 	MAPPPPASLPLPQRLAALAQTLQ~F1AWFVG2HFVLIAATVKYSLSWIAMKGYR--SNIS
NCU06968.1	MAPPPPADLPLAQRIQKLAQTLQ~F1AWFAG2HATLLLCVTRYAFSWLRMNYYGRMAQFC
FG04983.1 	MAPPPNPNLPLQERLMALAQTLQ~F1GWFVG2HLTLILATIRYGFSWLRMNYYTGMAKFS
AN6689.1  	MAPPPPSNLPLAERLKALAQTLQ2F~AWFIG2HVTLLGSVFRYLLSYATFNYYSGAAQVS
          	***** ..*** :*:  ****** * .** * * .*: .. :* :*:  :: *   ::..

MG08006.1 	YRFIFLSAAVTYGIVVYKTLRARAK--QRAPQISPLALAADENVQYLV1LAL--IWLFSP
NCU06968.1	YRTTFLSAALTYGIVVYKTWRARQK--TGAKPANVVSYLTDENVQYLL1LAL--VWLFMP
FG04983.1 	YRTAFIAAAVTYGIVVYKTMRARAKSGQRAAPT-PLAMLADENIQYLA1IAMSLVWLFCP
AN6689.1  	YRLAFISAAVTYGIVVYKGHVARGR-LQGSLPSILLKLAGDENVQYLG1MAL--VWLYSR
          	**  *::**:********   ** :    :     :    ***:***  :*:  :**:  

MG08006.1 	RYILATLPYGIYSFFHVATYMRANLIPVVFPP-KPA-AADSVD---GKPVAHPIADMIGR
NCU06968.1	QYPIAMLPFAIYSVFHVATYTRANLIPTIMPPTKVAPAAGASPSAKPQYTQHPMSDAIGS
FG04983.1 	QYPLALIPYTIYSVFHVATYTRANLIPAVVAP-RPAPEADGATPSRRTSVDHPIANKIGA
AN6689.1  	QVPLALLPFSVYSVFHVATYTRAHLIPTLQPPSTPAGSPGRAN-----AKQSPLAETIGR
          	:  :* :*: :**.****** **:***.: .*:  *  ..            *::: ** 

MG08006.1 	FVKEYYDASMSVVASLEILLWGSVFLSALLFQRRSWILLPLYTAFLRARYSQSIHVQNSF
NCU06968.1	FVKQYYDSSMSVVANIELVLWIRILLSAIIFQRRSWILLVIYTVFLRTRFAQSSHVQNSF
FG04983.1 	FVKEYYDASMAIVASLEIALWGRIFLSAILFQRRSWILIVLYTAFLRARYTQSTHVQHSF
AN6689.1  	FVKQYYDASMDLVAGLEIALLFRLLLAILTFSKGSFILLFIYLTFFRARYSQSSFVQQAV
          	***:***:** :**.:*: *   ::*: : *.: *:**: :* .*:*:*::** .**::.

MG08006.1 	AKLEANVENLLGNQGTPPAARQAWQAIKNGGRQFYLATDINRYIGGAAAPPKKTS
NCU06968.1	STFEARIDNLIGAQGTPPAARQAWETVKGLARQFHQATDVNKYISGAAAP-KKTS
FG04983.1 	SQLSARVDSFVSAQGTPPAARSIWQSVKEGARQFHDATAL-----GNAGPAKKSS
AN6689.1  	RHFTARVDASMSHQSTPPAVRQGWEGFKDAARQAYQATDVSR---FTAGAGKKPQ
          	  : *.::  :. *.****.*. *: .*  .** : ** :.      *.. **..
```
