# Supplementary material for: Patterns of Intron Gain and Loss in Fungi
Source: PLoS Biol. 2004 Nov 30;2(12):e422. doi: 10.1371/journal.pbio.0020422 (PMC532390; doi:10.1371/journal.pbio.0020422)
Supplement: Table S1 — Also available at http://genes.mit.edu/NielsenEtAl/. (4.3 MB ZIP). [file pbio.0020422.st001.zip › NielsenEtAl/html/1078.html]

AN1731.1.NCU02936.1.MG04244.1.FG04886.1


```
 CLUSTAL W (1.82) Multiple Sequence Alignments - Introns Inserted


Sequence 1: MG04244.1	530 aa
Sequence 2: FG04886.1	475 aa
Sequence 3: NCU02936.1	550 aa
Sequence 4: AN1731.1	478 aa
Alignment Length: 659 aa
Number Identitical Residues: 148 aa
Alignment Score (without introns) 8029


MG04244.1 	------------------------------------------------------------
NCU02936.1	MFPTSTSPASRQLVCNVGHARARTIASALGPAAASISRHHVSHSILMVGRINGVGRTYAT
FG04886.1 	----------------MSSALRRGTAQPLRWATASSSAITINTLRPCLA---------AR
AN1731.1  	----------------MKAATPRPSVRALSSGRSYRTARFVSR----------------T
          	                   :       .   . :  :    .                  

MG04244.1 	---------------------MATVVVK-----------NSSCLSVLPLKTVLRSLAVTT
NCU02936.1	RLIHSSTNRPKPTDHLTINNASSLPATGGSQSSEMPKTPGPSPLAVLPLTNVLRSWMTTT
FG04886.1 	RHIHSSDRR------------TNTIVEQRQIRDPLPSVPSKAPLSVLPLIMILRSLATMT
AN1731.1  	SNARSSLAAD-----------TNSLLQQAP---PSPKKQLASPLAKLPLSSVLRSLLILS
          	    ::                             ..    : *: ***  :***    :

MG04244.1 	VSSSPLLLNPSLAIMSGLAHSKSPLLNPDRNPILRWALKKTFYAQFCAGENPLEVRQTIA
NCU02936.1	VSSSPFLLPPSLAVMSVLAHTTNPVLNPDRNPLLRAFLKKTFYAQFCAGENPAEVRQTID
FG04886.1 	VSSSPLLLPPSLHVMGILANTSNPILNPDKNPLLRFFLKKTFYAQFCAGEKSPEIKKTID
AN1731.1  	VSSSSILLKPCIYTLSALAHPKTALLDVAKNPLLNLLVKHTIYKQFNAGENKLEVQRSIN
          	****.:** *.:  :. **:....:*:  :**:*.  :*:*:* ** ***:  *::::* 

MG04244.1 	GLKKIGFTGVLLGYAKEVVLSDKETKELHSCT--EGKASEECTQKEVIPWAEGTMETVRL
NCU02936.1	SLKQIGFSGVILGYAREVVLTDSETKDLASCAAEGGAAAEECIRTEINPWAEGTMETVRL
FG04886.1 	GLKNIGFNGVILNYAKEVVLTKDEGADLKN----AAVETESAIQNEILPWARGTLETVRL
AN1731.1  	AIKELGYRGVLLGYAREVLVGESKTD----------PRDEQASRQEIQTWLDGTLQTVDM
          	.:*::*: **:*.**:**:: ..:               *.. : *: .*  **::** :

MG04244.1 	VSPGDFVSVK2FTGAGRQALFSLSEKLPPPPALAKAIDDICKLAAARGVRLLFDAEQQAL
NCU02936.1	ASPGDFVALK~FTGAGRQALYSLSKQLPPSEALGSAIDGICNLAAERGVRLLFDAEQNAL
FG04886.1 	AEPGDFVALK~FTGAGSIALHQLKERMPPSPAMYKAIDSICQLAHERGVRLLFDAEQDML
AN1731.1  	AQEGDFVALK2FTGMGIQALEYLQNQAPPSPFMDEAIKQVCDLAISRNVRLLVDAEEQAV
          	.. ****::* *** *  **  *.:: **.  : .**. :*.**  *.****.***:: :

MG04244.1 	QPGIDDWTMHYMRKHN--APGRCVVYNTYQAYLKATPSVLASHLRAAAQSAAEGKPFAVG
NCU02936.1	QPGIDDWTLDYMRRYN---KSNAVVYGTYQAYLKSTPATLSKHLAIAAS---EG--FTLG
FG04886.1 	QDGIDDWTLEFIRKYNK-GLGEAVIFGTYQAYKKKCPEVLSAHLKLAKE---EG--FALG
AN1731.1  	QPGIEEWATMYQKYCNSRTPGRAIFYNTYQAYLCSTPATLARHLEISRK---EG--YTLG
          	* **::*:  : :  *.   ...:.:.*****    * .*: **  : .   **  :::*

MG04244.1 	AKLVRGAYLGSDPRHLIHDTKADTDACYDGVAAALLRQRWEGPLAGKD--------EGSE
NCU02936.1	VKLVRGAYLGADPRYLICDTKADTDGQYDGIAEALLRKTWSGPLQPPVPQSGSAEKETTN
FG04886.1 	VKLVRGAYLNSDPRELFHDTKEDTDACFDSLSASVLTREWNADVKG-----------SGP
AN1731.1  	VKLVRGAYLKTEPRHLIWAKKEQTDECYDGIVEALLTRRYNHMLKPAS------AEHTTE
          	.******** ::** *:  .* :**  :*.:  ::* : :.  :           .    

MG04244.1 	FPEVHMVLASHNSESVCKGRE--IIKRGEARTT--EVAFAQLQGMADEVSCDLIASACEK
NCU02936.1	FPDVGVVLATHNRESVLKGKA--LLDSGVAKLGYEQVAFAQLQGMADEVSCELVAGPHET
FG04886.1 	YPAASLVVASHNAESVRLSRA--IMEAGRAKS---DIAFAQLQGMADEVSCELVEANQQD
AN1731.1  	LPPVSVIVATHNRDSVRKAHALRLEQASRGEKSDVELSYAQLQGMADEISCELLQGFQTA
          	 * . :::*:** :**  .:   : . . ..    ::::*********:**:*: .    

MG04244.1 	-DAAAVKS--------------------YKYLVWGTTGECMKYLLRRAHENRDAVQRTRS
NCU02936.1	PKEMEMAAEKESEKGSEKGSEMVNKPQVYKYLVWGSTGECMKYLLRRAYENRDAVQRTRS
FG04886.1 	-KTMNLPA--------------------YKYLVWGTTGECMKYLLRRAHENKDAVQRTKG
AN1731.1  	GPENTKVAES---------------PNVYKLLTWGSVKECMGFLLRRAVENTEAVGRTKQ
          	       :..               .. ** *.**:. *** :***** ** :** **: 

MG04244.1 	GRDAMAAEVWRR2IAGTSWKSVHDSIQIVLFSH0MGGVLVAGTGQADMVESNMTHLIEYR
NCU02936.1	GREAMGREVRRR~VKG-----------------~-----LFGVSS---------------
FG04886.1 	SRDALWHELVRR~CKS-----------------~-----VVGLA----------------
AN1731.1  	SQEAMFSELRRR~ARR-----------------~-----AFGLRY---------------
          	.::*:  *: **                             *                  

MG04244.1 	TDGS2NAKVASSTLV0TPFESVEKCCGVGGGFLWSLLGCRCCVNRGVVWVAGVLELLFRV
NCU02936.1	----~----------~--------------------------------------------
FG04886.1 	----~----------~--------------------------------------------
AN1731.1  	----~----------~--------------------------------------------
          	                                                            

MG04244.1 	VSAI
NCU02936.1	----
FG04886.1 	----
AN1731.1  	----
          	
```
