# Supplementary material for: Patterns of Intron Gain and Loss in Fungi
Source: PLoS Biol. 2004 Nov 30;2(12):e422. doi: 10.1371/journal.pbio.0020422 (PMC532390; doi:10.1371/journal.pbio.0020422)
Supplement: Table S1 — Also available at http://genes.mit.edu/NielsenEtAl/. (4.3 MB ZIP). [file pbio.0020422.st001.zip › NielsenEtAl/html/1079.html]

AN2049.1.NCU04116.1.MG06877.1.FG01886.1


```
 CLUSTAL W (1.82) Multiple Sequence Alignments - Introns Inserted


Sequence 1: MG06877.1	398 aa
Sequence 2: FG01886.1	394 aa
Sequence 3: NCU04116.1	392 aa
Sequence 4: AN2049.1	414 aa
Alignment Length: 428 aa
Number Identitical Residues: 106 aa
Alignment Score (without introns) 6420


MG06877.1 	MSYTDDAVLAKLSALNESHDSIATAAQWIMFHR2RFADRTVQLWLSRLKESPSSKRLNLV
NCU04116.1	MAYNEDAVLSKLSALTETHESIATTAQWIMFHR2RHAAQTVHLWLTKLKDLPSPKRLNMI
FG01886.1 	MAYNDDSVLARLSSLNESHDSIATAAQWIMFHR2RHAERTVQLWLQRLKDSSSTKRLSLI
AN2049.1  	MAYTDDSVKAKLSALNETQEGIVTVAQWVMFHR2RHAERTAQLWLQKLRDSPAAKRLNLI
          	*:*.:*:* ::**:*.*:::.*.*.***:**** *.* :*.:*** :*:: .:.***.::

MG06877.1 	YLG--~-------DKE2DDFVNAFSP~IIAEAAAVAYKGASSDVQNKLRRVIDVWKDRNI
NCU04116.1	YLANE1VTQQSKARNK~DDFLQAFSP~FIADATALSYKGASSDIQNKLRRVVDVWRERKI
FG01886.1 	YLANE1VAQQSKIRHK~DDFIIAFAP~VIAEAASVAYKGAPAELQAKLKRVIDVWRDRSI
AN2049.1  	YLANE1VAQQSRARRK~EDFLIAFSP0IIAEAVAIAYKGASNDIQQKLRRVVEVWRQRNI
          	**...  :..:   .: :**: **:* .**:*.:::****. ::* **:**::**::*.*

MG06877.1 	FEPAIQAAVEARLED1LDKAKGVPKS----SSFGAPGLSG-PGGSVPSELSALVAPQQNV
NCU04116.1	FPIEVQDAIDSRLRE1LDSAR--SGG----TMFGSTSLSS-PAAAVPPELAPLVTSQQAV
FG01886.1 	FEAPIQAAIDARIGE1LDKARGMAKPGFSGSAFASGGAAASAGAAIPSEFAPLVSAHQSV
AN2049.1  	FEPPIQDAVEARVDE1IDKSRSTGKK----PLLGGSLFSG-PSGSTPSELQPIVPLQVAL
          	*   :* *:::*: : :*.::.        . :..   :. ...: *.*: .:*. :  :

MG06877.1 	SKQQLPMKSAISAANQDYERLTNPANPVPTAPVYAARLNGLLKTLATAEGAVAECVKARK
NCU04116.1	SKSAQALKTSLTTANSDYSKLMDPAHAPPQAPVYAARLNGLLKNLANAEGAVTECIKTRE
FG01886.1 	TKLSPPLKATVASASQEYEKQTDPSTPVPSAPVYAARLNGLLKTLANAENAVAECVKARE
AN2049.1  	SKAAVASGTSGTTANVEYEKLNDPNTPLPTPPVHAARLSSLLKTLANAESSVSEVIKSRR
          	:*   .  :: ::*. :*.:  :*  . * .**:****..***.**.**.:*:* :*:*.

MG06877.1 	ELIGALEKMLDSNREALRADEAQLVELSSRRRAIDAKKSDVEMAIMSGLSMQENSVSHGG
NCU04116.1	ELISALEKMLSSNRQALEAEQSQLRDLGTRKTAVEEKKQAIELSIIGGLPHNTQEPATGE
FG01886.1 	GLVSGLETLLNANRAALEQEKSDHAQLVSRKAEIEEKKQQVEIGIMRALGPADSNGNPGD
AN2049.1  	ALIDGLEKILETNRTALSQEEALAVQLQERRAETEAKKREVEDAIMRGLSAENTPAADSG
          	 *:..**.:*.:** **  :::   :*  *:   : **  :* .*: .*         . 

MG06877.1 	--APDGPPAPDPAQPQVEALTPPSAGADDDMYDNSASFAPPPPEPVPATEQPTEVQP---
NCU04116.1	ERAPSSSDGHGIARPQVEALTPP------HVQDHDDFYDHRPSEQQQPQNGHTHPTG---
FG01886.1 	--AESLIAPAEPDRPEMEALTPP-------AFD---PFDAPTPEALTPEGEPAVAPA---
AN2049.1  	---SATGSGEAVPRPAIEGLTPPP----VEAITPIGSPSQQPQDTTSSAENGAQTLGGFS
          	             :* :*.****.                 . :   .    :    . :

MG06877.1 	------MASGIEMLSNIAS----QYHSVPTNGN-TNKRRRVESGEEFPDLGKDDGIDPDV
NCU04116.1	------SAPGIEMLSALAS----QYESVPTREA---KKRKIDETADIPDVG----IDADV
FG01886.1 	------PATEEETAS---------YQSLPISINGSNKRRRIDT-EEFPDLGGDDGIDADV
AN2049.1  	LGVDQPPVAPIPGLSGMGQPSYGELQQESMNGFQAKKRKVTHSEEDYAKFAGG-DLDADV
          	 . ... ..     *  ...: .. .. .     :.*::  .   : .... . .:*.**

MG06877.1 	KEMLSNDVKTEAA
NCU04116.1	AEMIQKESTI---
FG01886.1 	AQMLKEESQS---
AN2049.1  	AELLNQEGHSQS-
          	 :::.::   .:
```
