# Supplementary material for: Patterns of Intron Gain and Loss in Fungi
Source: PLoS Biol. 2004 Nov 30;2(12):e422. doi: 10.1371/journal.pbio.0020422 (PMC532390; doi:10.1371/journal.pbio.0020422)
Supplement: Table S1 — Also available at http://genes.mit.edu/NielsenEtAl/. (4.3 MB ZIP). [file pbio.0020422.st001.zip › NielsenEtAl/html/108.html]

AN9496.1.NCU03752.1.MG00712.1.FG07151.1


```
 CLUSTAL W (1.82) Multiple Sequence Alignments - Introns Inserted


Sequence 1: NCU03752.1	278 aa
Sequence 2: MG00712.1	248 aa
Sequence 3: FG07151.1	263 aa
Sequence 4: AN9496.1	270 aa
Alignment Length: 334 aa
Number Identitical Residues: 86 aa
Alignment Score (without introns) 4597


MG00712.1 	MESVTS-------AGGLPPDLFDATTLQSLASVLVIVSIAYGTSLKALSPTTPGS-LRFL
NCU03752.1	MASTTAPLSLESFLSTLPPDLFDQTTLISLASTVAILLAAYSLSRVALDPKTTTSRYRLL
FG07151.1 	MAAETA--------SSLPPDLFDQTTIISLLSTLAIVFTAYAASLKFLPSSSSGT-LRFL
AN9496.1  	MSTVTP---------PASSFTIDTPTILCILFALSFMPIAYVLGNNLIPSSQTRN--RIL
          	* : *.           ..  :* .*: .:  .: ::  **  .   : .. . .  *:*

MG00712.1 	FIWHMFDALIHFALEGSFLYHCFFSWLPVSDLTVKQLAKFHPTPEHFLGTSGRIYGAQAG
NCU03752.1	FIWHAFDALIHFCLEGSFLYHCFFSSAPLGHLDPK---EVFVDPYNYLGRIDKVHGAQAV
FG07151.1 	FIWHLADALCHFLLEGSFLYHCFFSHLPLLN-DVKT--ELFPTPAGFLGYSDRVYGAQSG
AN9496.1  	FYWHAYDALTHLFIEGSFLYECFTSYATLPA-------GFAAPEPAFLGIKDRVYGAAHG
          	* **  *** *: :******.** *  .:          .      :**  .:::**   

MG00712.1 	DGFWAN--------------------------LWMVYAKADKRWAGADL0TVISLELLTV
NCU03752.1	AGRFVDGVVGGPAKAGDVGGGLLEKVVRGTAELWMVYAKADKRWAGVDL0SVVSLELLTV
FG07151.1 	GDNPFA-------------------------MLWMVYAKADKRWAGVDL~GVVSLELLTV
AN9496.1  	SAPSAR--------------------------LWQEYAKADKRWATADA~TVISLELLTV
          	                                **  ********* .*   *:*******

MG00712.1 	FVVGPMAVWVCYDIAKK----------------------------------NPRYNITMI
NCU03752.1	LVVGPLACWVCYDIAKK----------------------------------NSRVNIVMI
FG07151.1 	FFDGPLAVYVCYCLARK----------------------------------DPKVSIWMI
AN9496.1  	FLGGPAAIYVCYLVWQSSCTQPAPKPTSSKSSSPKSTSKSSAAKLESQGASKAKLWLVAT
          	:. ** * :*** : :.:.:..:...:::.:::..:::.::::. .:..::..:  :   

MG00712.1 	MLATAEIYGG1FMTFCPEWLTANVNLDTSNFIY1LWFYLVFFNM2LWVFIPIYSIYVGSK
NCU03752.1	MIATAEIYGG1WMTFCPEWLVGSVNLDTSNWMY~LWLYLAFFNG~LWVVIPAYVIYVASG
FG07151.1 	ILATCELYGG1FMTFCPEWLVGNPNLDTSNFMY~LWVYLIFFNT~LWVWIPLWVIWYSVK
AN9496.1  	ALATAELYGG~FMTFVPEWLTGSTQLDTSNAVY~LWFYLFFFNT~LWVWIPLWVLWEAAK
          	 :**.*:*** :*** ****... :***** :* **.** ***  *** ** : :: .  

MG00712.1 	DIFDAFSVR------~-AAAG~QIKKKQK-----------
NCU03752.1	EIMGAFKVR------~-DAAA~KAKKSL------------
FG07151.1 	DISNALSVRQGKKNL2YDLSS0KYRNTFTSQLEWNWFGVS
AN9496.1  	EVKRAFVLA------~--EGV~EGKKVK------------
          	::  *: :           .  : ::
```
