# Supplementary material for: Patterns of Intron Gain and Loss in Fungi
Source: PLoS Biol. 2004 Nov 30;2(12):e422. doi: 10.1371/journal.pbio.0020422 (PMC532390; doi:10.1371/journal.pbio.0020422)
Supplement: Table S1 — Also available at http://genes.mit.edu/NielsenEtAl/. (4.3 MB ZIP). [file pbio.0020422.st001.zip › NielsenEtAl/html/1080.html]

AN7679.1.NCU04293.1.MG00949.1.FG10388.1


```
 CLUSTAL W (1.82) Multiple Sequence Alignments - Introns Inserted


Sequence 1: MG00949.1	376 aa
Sequence 2: FG10388.1	376 aa
Sequence 3: NCU04293.1	379 aa
Sequence 4: AN7679.1	394 aa
Alignment Length: 404 aa
Number Identitical Residues: 145 aa
Alignment Score (without introns) 8262


MG00949.1 	MMNGY-EKNNHDEDPFAP-SGSVV--SAFDAFP1KSKPQYVTRTSGGGKWTVAMLLVSAI
NCU04293.1	-MNGYDEKRQLDEDAFGA-KGSIV--SAFDAFP1KSKPQYVTRTTAGGKWTVFVGLISFI
FG10388.1 	MMNGT-EKPDYDEDKFGPKEGSLV--AAFDAFP1KSKPQYIQRTSGGGKWTVAVSIISLI
AN7679.1  	-MNGF-AAHGLDEDAFAEKSGLTGGLRTFDAFP1KTKPSYTTPSRRGGQWTVLILIICTI
          	 ***       *** *. ..*   .  :***** *:**.*   :  **:*** : ::. *

MG00949.1 	LTWSELARWWRGVETHTFAVEKGVGQSMQINMDTVVHMRCQDIHVNVQDAAGDRIMAAAR
NCU04293.1	LFWSEASRWWRGSESHTFAVEKGVSHALDINLDIVVKMKCQDIHINVQDAAGDRILAASR
FG10388.1 	LIWGELGRWWRGAESHNFEVEAGVSREMQINLDIVVKMNCDDIHVNVQDASGDRIMAAKR
AN7679.1  	FSITEFRTWLKGHETHHFTVEKGVSHDLQLNFDAVIHMPCDALHINIQDAAGDRVLASEM
          	:   *   * :* *:* * ** **.: :::*:* *::* *: :*:*:***:***::*:  

MG00949.1 	LKMDDTTWAQWVDGSGVHRLGHDQHGKVVTGEGH------EEGFGEEHIHDIVALGKKRA
NCU04293.1	LHRDPTVWQHWVDNKGIHKLGRDAQGKVVTGEGYMQGQGHDEGFGEEHVHDIVSLGRRKA
FG10388.1 	LHTDKTLWGQWADNKGVHKLGRDDQGRVNTGQGYNDPKYEDEGFGEEHVHDIVALGKKRA
AN7679.1  	LKKEPTSWKLWMDKRNYHSSEYQTLSDSRGDEER--VAAMEEDVHAGHVLNELRRNGKR-
          	*: : * *  * *  . *    :  .    .:        :*..   *: : :  . :: 

MG00949.1 	RWSKTPRL-WGATPDSCRIFGSLDLNKVQGDFHITARGHGYIEFGDHLDHSA1FNFSHIV
NCU04293.1	KWARTPRL-WGATPDSCRVFGSLELNKVQGDFHITAKGHGYMEFGQHLDHSA1FNFSHII
FG10388.1 	KWAKTPRF-RGN-ADSCRIYGSLDLNKVQGDFHITARGHGYMGHGEHLDHSK1FNFSHII
AN7679.1  	KFAKGPKLRRGDVVDSCRIYGSLEGNKVQGDFHITARGHGYRDGREHLDHSA1FNFSHII
          	:::: *::  *   ****::***: ***********:****    :*****  ******:

MG00949.1 	NEFSFGDFYPSLVNPLDKTVNTCEKNFHKFQYFLSVVPTLYSVK----------SSTGAF
NCU04293.1	SELSFGPFLPSLVNPLDQTVNIASANFHKFQYFISVVPTVYSSS----------GKS---
FG10388.1 	SELSYGPFYPSLENPLDGTVNTADGNFHKFQYYLSVVPTVYSVN----------SRS---
AN7679.1  	TELSFGPHYPSLHNPLDKTIATTEFHYYKYQYFLSIVPTIYSRNQNLRLDALPSSSSARS
          	.*:*:* . *** **** *:   . :::*:**::*:***:** ...   .: .:. :.  

MG00949.1 	GYSTIFTNQYAVTEQSSEISEM--NVPGIFFKYDIEPILLDIEESRDTILVFLIKVINIL
NCU04293.1	----IVTNQYAVTEQSQEVTER--IIPGIFVKYDIEPILLHIDEERDSFLVFIIKVVNVI
FG10388.1 	----ILTNQYAVTEQSKAVDDR--YIPGIFFKYDIEPILLTVHESRDGIISLFVKIINII
AN7679.1  	NKNLIFTNQYAATSQSDAIPESPYVIPGIFFKYNIEPIMLLISEERTGFLNLLIRIVNTV
          	. . *.*****.*.**. : : .  :****.**:****:* : *.*  :: ::::::* :

MG00949.1 	SGAMVAGHWGFTMSEWIKEVLGKRRRASS---NGVLGNKAGYDE--
NCU04293.1	SGALVAGHWGYRISDWCQEVWGKRRRRAGGPSEGMLGGK-GHERDD
FG10388.1 	SGVLVAGHWGFTISDWIHDVIGRRRRSNGG--VGVLGSKEGFDQ--
AN7679.1  	SGVLVTGGWVYQIMTWLGELR--RRRRGGEKSEGYLHGKLEEE---
          	**.:*:* * : :  *  ::   ***  .  : * * .*   :
```
