# Supplementary material for: Patterns of Intron Gain and Loss in Fungi
Source: PLoS Biol. 2004 Nov 30;2(12):e422. doi: 10.1371/journal.pbio.0020422 (PMC532390; doi:10.1371/journal.pbio.0020422)
Supplement: Table S1 — Also available at http://genes.mit.edu/NielsenEtAl/. (4.3 MB ZIP). [file pbio.0020422.st001.zip › NielsenEtAl/html/1081.html]

AN2896.1.NCU09058.1.MG03335.1.FG06084.1


```
 CLUSTAL W (1.82) Multiple Sequence Alignments - Introns Inserted


Sequence 1: NCU09058.1	343 aa
Sequence 2: FG06084.1	319 aa
Sequence 3: MG03335.1	349 aa
Sequence 4: AN2896.1	305 aa
Alignment Length: 356 aa
Number Identitical Residues: 146 aa
Alignment Score (without introns) 7121


MG03335.1 	MPPVSRILSYAPRVAIRPSSQLARPARAFAVGTVRYYSAEQPEEPLIRVTDLPAPNSGHI
NCU09058.1	---------MAVRIALPRNTTSSFRRFVRVAG--RFYSTEAPAGPIIRVTNLPAPNSGHI
FG06084.1 	---------MRPRLSQPVTT-------LRVLR--RCYS-SATAEPLIRVTNLPAPNTGHI
AN2896.1  	------MPPRLTRLALPIRASPTVSLRVKVAR----YS-TSPDDAVIQTQYVPAPGSGNI
          	        .   *::    :  :      .      **   .  .:*:.  :***.:*:*

MG03335.1 	RVLELNRPKARNAISRALLASLREEVHSIARQYDAQTGEEIPTPSWNKRFGGIAGDSEKG
NCU09058.1	RILELNRPAARNAISRALLSSLRDEIDDVHSQYDAATGEEKPVASWQKRFGGVAGEDEKG
FG06084.1 	RILELNRPSARNAISKALLANLRAEIDELHSQYGPN-GEELPLP---QRFGGAAGVDEKG
AN2896.1  	RVLLLNRPNARNALSKNLLTSLAQHVNSIS------------------------AEGGNG
          	*:* **** ****:*: **:.*  .:..:                         . . :*

MG03335.1 	PTRALVLASAVESSFCAGADLKERRGFTQEE2TNEFLANLRSTFAALDALPIPTISAISS
NCU09058.1	PTRALIISSAVDTSFCAGADLKERKGFSQEE2TAAFLTSLRTALTSLQNLPIPTISAISS
FG06084.1 	PTRAVVLASAVDTSFCAGADLKERKGFTPGE2TAEFLTNLRNTFTSLSNLPIPTISAISS
AN2896.1  	PTRALVIGSNADSAFCAGADLKERLHMTKDE2TNAFLAKLRGTFRDLAALPVPTISAVSS
          	****:::.* .:::**********  ::  * *  **:.** ::  *  **:*****:**

MG03335.1 	RALGGGLELALCTHFRVLTSNAIVSLPETRLGIIPGAGGTHRLPRLIGLGRARDMIVTGR
NCU09058.1	IALGGGLELALATHFRVLTSNAVVGLPETRLGIIPGAGGTYRLPQLIGIPRARDLILTGR
FG06084.1 	VALGGGLELALSTHFRVLSSNATVGLPETRLGIIPGAGGTHRLPALIGLSRARDLILTGR
AN2896.1  	LALGGGLELALCTHLRVFGSNSTVALPETKLAIIPGAGGTYRLPSLIGVNRARDLILTGR
          	 **********.**:**: **: *.****:*.********:*** ***: ****:*:***

MG03335.1 	AVSGAEAYFLGLADRLVEVLPPDEQEAAD-----TTDKDAALLSAAREAALTEAVRLASQ
NCU09058.1	RVSAPEAYFLGLADRLVEVAPESEEQAKEWAAMEQEPRDKMILSLARRTALSEAVRLAME
FG06084.1 	RVGAPEAYFLGIADRLVEVVPKDERDG------------SAILSEARKAALSEAVRLAQE
AN2896.1  	RVTGPEAYFIGLCDRLVEILPEEEQKE----------------GAAREKVLRESIKLALD
          	 * ..****:*:.*****: * .*..                 . **. .* *:::** :

MG03335.1 	ICEGGPIGIRAALQAVQ--APSQETENKMYERVIGTEDRNEALKAFAEKRKPVFKGR
NCU09058.1	ICEGGPVAIRAALKAVQ--EPSEMVENDMYLRVVRTEDRDEALKAFAEKRKPVFKGR
FG06084.1 	ICEGGPIGVRAGLQAVA--WAREEVENKMYERVVNSEDRNEALKAFGEKRKPIFTGR
AN2896.1  	ICDGGPIAIKQALKAVNGYEQGEAAENEAYDGVVETEDRREALIAFAEKRKPAFRGR
          	**:***:.:: .*:** .    : .**. *  *: :*** *** **.***** * **
```
