# Supplementary material for: Patterns of Intron Gain and Loss in Fungi
Source: PLoS Biol. 2004 Nov 30;2(12):e422. doi: 10.1371/journal.pbio.0020422 (PMC532390; doi:10.1371/journal.pbio.0020422)
Supplement: Table S1 — Also available at http://genes.mit.edu/NielsenEtAl/. (4.3 MB ZIP). [file pbio.0020422.st001.zip › NielsenEtAl/html/1082.html]

AN6636.1.NCU07442.1.MG02766.1.FG08596.1


```
 CLUSTAL W (1.82) Multiple Sequence Alignments - Introns Inserted


Sequence 1: NCU07442.1	616 aa
Sequence 2: MG02766.1	632 aa
Sequence 3: FG08596.1	613 aa
Sequence 4: AN6636.1	642 aa
Alignment Length: 681 aa
Number Identitical Residues: 252 aa
Alignment Score (without introns) 13150


MG02766.1 	MSSEAVQSWARQVQNLAQEKLCGSAPAQASN~VT~GFTDCVALLRPFDISDVPQYVWAVV
NCU07442.1	MAAEIPRSLLVQLR----------TALEDTP2VV~G-------------SYIPESSWALV
FG08596.1 	MEDQEPLLLAVWRR--------CSEMAAEHN~IS2QSTLS-------AAGTVAAIIAATL
AN6636.1  	MECLAPYIPPALLS---------LVERAQEQ~VQ~N---------------QTHTLSIAV
          	*                               :                   .      :

MG02766.1 	AAVLGGLVFLKAFSGENDRPVPYTVPSP-------------KTP---EMVEILQKPSIK0
NCU07442.1	WSALAALAVWYMASRE-DQPIRYTIPPA-------------NFP---KEENILENPSIK~
FG08596.1 	ILRITNAIAAASISAATSRPRKYTVPSP-------------KVP---EPHTTVDITSVK~
AN6636.1  	LSLSAVLLGYLFVAGSRESPVSFTVPNPPEINPHWEGSKWEDLPQGSEERNVIEGQIRG0
          	             :   . *  :*:* ... ..  ..:. .. *..::    ::      

MG02766.1 	VSGSTAIQCYNPATGQFLG-FVNPATPAAIDRAIEQAATAQKKWALTSFRERRKVLRSML
NCU07442.1	ASGTSAIQCYAPATGQFLG-FVNPSTPEGIDRAIDQAHAAQVEWAKTTFRQRRAVLRSLL
FG08596.1 	VSGSSAVQCYAPATGQFLG-NVNPSTPAAIDRAVSAAATAQKTWAETTFGQRRAVLSSLL
AN6636.1  	QWNENLIMSYCPADGRVLGSGIKPATADDVDRAIQAASRAQEQWATTTFAERRRVLKTLL
          	  . . : .* ** *:.**: ::*:*.  :***:. *  **  ** *:* :** ** ::*

MG02766.1 	Q~YILDNQEEICRVAGMDSGKTMVDAQLGEILVTVEKLQWTIAHGEKALRPERRPTNLLM
NCU07442.1	Q~YVLDNQEEICRVACLDSGKTMVDAQLGEILVTAEKLQWTIKHGEKALRPESRPTNLLM
FG08596.1 	Q~HVLDNAEEIVKIACLDSGKTMVDAQLGEILVTAEKLKWTLSHGEQALRPSRRPTNFLM
AN6636.1  	K2YVLEHQDEIVIACCLDSGKTKVDATFGETLVTAEKLKWTIDHGERALSPESRPTNFLM
          	: ::*:: :**   . :***** *** :** ***.***:**: ***:** *. ****:**

MG02766.1 	MYKRNSVHYEPLGVIAALVSWNYPFHNLLGPIISAIFAGNGILVKVSENTAWSSSYFASI
NCU07442.1	AYKRNTVHYEPLGVVAALVSWNYPFHNLIGPVISALFAGNGIVVKVSEQTAWSSQWFTSV
FG08596.1 	MYKRNTVHYEPLGVVAALVSWNYPFHNFIGPVISALFSGNGILVKVSEQTAWSSQYFTNI
AN6636.1  	MYKKNQVIYEPLGVVSACVSWNYPFHNFISPVISAIFAGNGIVVKPSEQTAWSSVYFLNI
          	 **:* * ******::* *********::.*:***:*:****:** **:***** :* .:

MG02766.1 	ARGALFAHGYDPSLIQ~TVACWPQVAGHITSHKGISHITFIGSQAVAHKVAESAAKVLTP
NCU07442.1	IRGALVAHGHNPALVQ1TVVCWPQTANHITSHPKISHITFIGSQPVCKKVAESASKALIP
FG08596.1 	ARGALIAHGHDPQLVQ~TIVCWPQAAGHLTSHPSISHITFIGSQSVAHHVAASAAKSLTP
AN6636.1  	IRGALENCGHPRDLVQ~SVVCLPKVADHLTSHPGIAQITFIGSRPVAHKVCESAAKALTP
          	 ****   *:   *:* ::.* *:.*.*:***  *::******:.*.::*. **:* * *

MG02766.1 	VCAELGGKDAFIVLDSAAK--DLNRIVEMMLRGTFQSAGQNCIGIERIIATPAVYDRIVL
NCU07442.1	VLAELGGKDASIILASAPKS-DLPRIVNTLMRGTFQASGQNCIGIERIVVAPQHYDTLLS
FG08596.1 	VVAELGGKDPFIVLDSASG--DLKRIAEVILRGTFQAAGQNCIGIERVIAPSAIHDKLVE
AN6636.1  	VTVELGGKDPSVILDDSRTISEVTSVASVLMRGVFQSAGQNCIGVERVIALPGVYDKLLD
          	* .******. ::* .:   :::  :.. ::**.**::******:**::. .  :* :: 

MG02766.1 	ALAERVRGLRVGPGPL------------DADVGAMVSDASFDRLEHLVADAVRCGARLLA
NCU07442.1	MLTPRVRALRLGP---------------TADVGAMISDNAFARLEGLVADAVKQGARLLA
FG08596.1 	MLAPRVNALRLGP---------------DADVGAMISDASFDRLEELIAEAVSQGARLLA
AN6636.1  	TVTSRIKALRLGSVLLDTKPNNPNNKSGAPDVGAMISPASFSRLEFLIQRAVSQGARLVA
          	 :: *:..**:*.   .:........:. .*****:*  :* *** *:  **  ****:*

MG02766.1 	GGKRLIHPQHPSGHYFTPTLVVDVTPDMALAREECFGPIMTLMRAPANTAKAVLDVANAP
NCU07442.1	GGKRYAHPEYPSGHYFVPTLLVDVTPDMAIAQEECFGPIMVVMRAASSSAEDILAVANAP
FG08596.1 	GGKRYDHPEYPSGHYFQPTFLADVTPEMRIAQNECFAPVLTLLRAKSSSPEDILSIANAP
AN6636.1  	GGKQFEHPTYPLGHYFTPTLLADVTPSMEIAQTELFAPVFLMMRASSVS--DAITIANST
          	***:  ** :* **** **::.****.* :*: * *.*:: ::** : :    : :**:.

MG02766.1 	DFGLGGSVFGRDSDPVLKEVVRGLRTGMVAVNDFATYYAVQLPFGGVGGSGYGRFAGEEG
NCU07442.1	DFGLGSSVFGSEWDSTLHEVVRGLKAGMVAVNDFGATYAVQLPFGGVAGSGYGRFAGEEG
FG08596.1 	NFGLGASVHGSERDPNVQPIVKGLRAGMVAVNDFAVYYAVQLPFGGVGGSGYGRFAGEEG
AN6636.1  	QYALGASVFGYN-TRDVNACVSGIKAGMVSVNDFGSYYTVQLPFGGVKGSGYGRFAGEEG
          	::.**.**.* :    ::  * *:::***:****.  *:******** ************

MG02766.1 	LRGISNAKSICEDRAGWLGVRTAIPPPMRYPVRD-------QDRSWRFAKGVVELGYGLT
NCU07442.1	LRGLCNIKAVCEDRFGWLGVRTAIPRPMQYPVPD-------QERSWRFARGVVEVGYGMG
FG08596.1 	LRGLCNAKAVCEDRFGWLGVRTSIPPPVQYPIKS-------QSDSWKFTQGVVELGYGAP
AN6636.1  	LRGVSNIKAICVDRFPRL-MATRIPPRVDYPIMKGEAEKENGDGAFEMCKGVVETGYQIT
          	***:.* *::* **   * : * **  : **: ...:.... . ::.: :**** **   

MG02766.1 	LGAK~VRGLVGLAKNS-----------
NCU07442.1	LGRK0T-GVVPLRTTDNGSTARYMHIP
FG08596.1 	I-RK~LKGLGKILQNM-----------
AN6636.1  	LAGR~VRGILRLIGNM-----------
          	:. :   *:  :  .
```
