# Supplementary material for: Patterns of Intron Gain and Loss in Fungi
Source: PLoS Biol. 2004 Nov 30;2(12):e422. doi: 10.1371/journal.pbio.0020422 (PMC532390; doi:10.1371/journal.pbio.0020422)
Supplement: Table S1 — Also available at http://genes.mit.edu/NielsenEtAl/. (4.3 MB ZIP). [file pbio.0020422.st001.zip › NielsenEtAl/html/1083.html]

AN5341.1.NCU04379.1.MG01550.1.FG09591.1


```
 CLUSTAL W (1.82) Multiple Sequence Alignments - Introns Inserted


Sequence 1: MG01550.1	190 aa
Sequence 2: FG09591.1	190 aa
Sequence 3: NCU04379.1	190 aa
Sequence 4: AN5341.1	189 aa
Alignment Length: 190 aa
Number Identitical Residues: 163 aa
Alignment Score (without introns) 6439


MG01550.1 	MGKS2QSKLSQEQLAELQKSTHFDKKELQQWYKG1FLKDCPSGTLTKEEFQKIYRQFFPF
NCU04379.1	MGKS2QSKLERDKLEELEKATHFDKKELQQWYKG1FLKDCPSGMLTKAEFQKIYAQFFPF
FG09591.1 	MGKS2QSKLSQDQLVELQKSTHFDKKELQQWYKG1FLKDCPSGTLTKEEFQKIYRQFFPF
AN5341.1  	MRR-~QSKLSPTQLEELQKATHFDKKELQQWYKG1FLKDCPSGQLTKEEFQDIYRKFFPF
          	* :  ****.  :* **:*:************** ******** *** ***.** :****

MG01550.1 	GDPSSFADYVFNVFDSDKSGTIDFKEFIVALSVTSRGKMEDKLDWAFQLYDIDGDGKISY
NCU04379.1	GDPSSFADYVFNVFDTDKSGTIDFKEFICALSVTSRGKMEDKLDWAFQLYDIDGDGKITY
FG09591.1 	GDPSSFADYVFNVFDSDKSGTIDFKEFICALSVTSRGKMEDKLDWAFQLYDIDGDGKISY
AN5341.1  	GDPSSFANYVFRVFDSDNSGMIDFKEFICALSVTSRGRMEDKLDWAFQLYDIDGDGKITY
          	*******:***.***:*:** ******* ********:********************:*

MG01550.1 	DEMLQIVEAIYKM0VGSMVKLPEDEDTPEKRVRKIFRMMDKDENGSLDMQEFKEGSKRDE
NCU04379.1	DEMLKIVEAIYKM0VGSMVKLPEDEDTPEKRVSKIFRMMDKDENGSLDMKEFKEGSQRDE
FG09591.1 	DEMLKIVEAIYKM0VGSMVKLPEDEDTPEKRVRKIFRMMDKDENGSLDMEEFKEGSKRDE
AN5341.1  	DEMLAIVEAIYKM0VGSMVKLPEDEDTPEKRVRKIFRMMDKDENGSLDMEEFKEGSKRDE
          	**** ******** ****************** ****************:******:***

MG01550.1 	TIVSALSLYDGLV
NCU04379.1	TIVSALSLYDGLV
FG09591.1 	TIVSALSLYDGLV
AN5341.1  	TIVSALSLYDGLV
          	*************
```
