# Supplementary material for: Patterns of Intron Gain and Loss in Fungi
Source: PLoS Biol. 2004 Nov 30;2(12):e422. doi: 10.1371/journal.pbio.0020422 (PMC532390; doi:10.1371/journal.pbio.0020422)
Supplement: Table S1 — Also available at http://genes.mit.edu/NielsenEtAl/. (4.3 MB ZIP). [file pbio.0020422.st001.zip › NielsenEtAl/html/1084.html]

AN5167.1.NCU07565.1.MG00134.1.FG06762.1


```
 CLUSTAL W (1.82) Multiple Sequence Alignments - Introns Inserted


Sequence 1: NCU07565.1	1161 aa
Sequence 2: FG06762.1	1158 aa
Sequence 3: MG00134.1	1161 aa
Sequence 4: AN5167.1	1166 aa
Alignment Length: 1234 aa
Number Identitical Residues: 548 aa
Alignment Score (without introns) 25483


MG00134.1 	MFARSKSKFHGFGADKRQ-----------QESGFL~G----SDSRS-----RTTSERPYT
NCU07565.1	MFSRLHSKHKGHSKEQQNRRPPDHQASTLHDLGML~GPSSTSILSA-----SGGSRRPDF
FG06762.1 	MFRRLRRNQDHDTASVKTRDEDRALAVNLREFGLS~-----SSRAS-----KSSSRRPQT
AN5167.1  	MWKHRPPSRSDSKSGTVDSGERGFFGWSKKKTPLS1FARPESPARSPLRPFRSLSRADRA
          	*: :   .                 . . :.  :   . . *   :.  .    *.    

MG00134.1 	ATSFNAPSLDAPSYAP-----SFPEKN1SSISSGTIKPPQSHRSSASVSSNHSVDSYGRS
NCU07565.1	DRSNHSYHNHSGVPARQYPATSYSERD1SPIPNGHLGAQSHYSLVTPSSPSQSREST---
FG06762.1 	GSDSRPFTETQSINS-----KSLRGQD~SVSSTG----SVHSGLSNNSRRVGGIDNN---
AN5167.1  	DSSFSSHTAASRARAPSALTADSYYSD~APRSAAQFSAYSNRSVSRTSSHETFNTLPGRP
          	  .  .        : .  : .    : :  . .   .                   . .

MG00134.1 	IAPNMSSERARTRRERTFVGSECAVCEEPLEHTLRGERILQFSCGHVSHEACFYEYIKEI
NCU07565.1	-MMNSNA--SRGRRERTFVGSECAVCEEPLEHTLQGERILQFSCTHVSHEACFYEFIREF
FG06762.1 	-VMSSNADRSRSRRERTFVGSECAVCEEPLEHTLRGERILQFSCTHVSHEACFYEFIREF
AN5167.1  	MAGLMDVDRSRSRRERTFVGSECAVCEEPLEHTLRGERVLQFSCAHVAHEACFYEYLREI
          	     . . :* **********************:***:***** **:*******:::*:

MG00134.1 	ESQYCPSCNAPLHLDTSRGGNVLDI-----------------E1KISNLVRSVNS--DSR
NCU07565.1	ESQFCPTCNAPLHLDTSRGGNVLDI-----------------E1KISSMVRSVSVN-DTR
FG06762.1 	ESQYCPTCDAPLHLDTSRGGNVIDIGRPAILGQLDSTLTTSQE~KISNMVRSVGT--DSR
AN5167.1  	EGQYCPTCDAPLGLDSTRGGNVLDIGKQLSDPRADG-ADGAIE~KLSNIVRSVNSDAMTQ
          	*.*:**:*:*** **::*****:**.        ..    : * *:*.:****. .: ::

MG00134.1 	SIGTPTPTPQQWDDHGRPQSRGSNARSGSGVG-LSHPSSHNGRDNAMPR-SNRDTRDSGH
NCU07565.1	SPPAPTPAPAPAPAPSQQPWDDQLARAPSRASSARQDGGLAP--HTMARVSQRDSRDAPS
FG06762.1 	SAMTPTPTPWESHTARAPSVDSNQRRPSQQQQQQQQQQQHAGGRDSVPRGSMRDSREAPP
AN5167.1  	RSGLTTPTPWDSVTSRQNPPSEVGGRPYPTSDVGSRP---YPASDAGSRPYNRDSRDTYS
          	    .**:*                *.        :        .: .*   **:*::  

MG00134.1 	SN-PHSERYMRHGRSDSEAT----GVASSTGYPETTQSGGPRRAHDYDLQAMETSLASP-
NCU07565.1	SSDHRYTPNSRHARSDSETTGATGGMASSGGYPETMQSG-PPRRHDYDVQAMETSISSPP
FG06762.1 	SD--RYGP-SRHARSDSEAT----GVASSGGYPETTQSG-PARRHDYDVQAMETTPTSP-
AN5167.1  	NRRDSKDTGMQRERIERLAS-VSRHHSRNGSAAGSSGEYHEGRRHDYDLQAMESDLSPRT
          	.         :: * :  ::  :   : . . . :  .    * ****:****:  :. .

MG00134.1 	RSIARNPIPLPTVTVRSEFPTINRSRQQQTLTCLITIEVPDCKWRPDPEDLPMGNIHSPQ
NCU07565.1	RAITRNPIPAPTVTIRSEFPTISKSRQQQTLTCLVTVEVPDNKWRPDPEDLQNG------
FG06762.1 	RPITRNPIPAPIVTVRSEFPTLSRSRQQQTLTCLVTVEVPDNKWRPDPEDLGAP------
AN5167.1  	APTTKNPIPAPIVTIRSEFPTISRSRQQQSLTCLITVEVPEGNWRPDTDDLRTG------
          	 . ::**** * **:******:.:*****:****:*:***: :****.:**         

MG00134.1 	QMHHQAPQQVPSRMDARVAEDPYG-PTSPTQSVPRFFPYESREVLEEMTENLRNRVDNWH
NCU07565.1	---------QPMLPSS-RAEEPYPRAPSPARSVPRFYPYESPEVLAEMTENLRSRVDNWH
FG06762.1 	---------APQPPVPNRAEEAYGRPPSPARSAPRFYPYESTDALEEMTDSLRNRVDNWH
AN5167.1  	------------STHSLPKDEPYPSRFPSVPEKP--APFEPQENLDEIAEELRAKVDNWH
          	               .   ::.*    ... . *   *:*. : * *:::.** :*****

MG00134.1 	GLDFSR~FGKLRLYGTLRVGKDKISWQELECYLFAEMLICVKEKKTPAGAQQQWDENGLP
NCU07565.1	GLDFSR2FGKLRLYGTLRVGKDKVSWQELECFLFAEMLICVKEKKNVPSAPGQWEDEAAR
FG06762.1 	GLDFSR~FGKLRLYGTLRVGKDKVSWQELECFLFAEMLICVKEKK-IPQSQQWDENGQPR
AN5167.1  	GLEFQR2FGKLRLHGHMRVGKDRESWQDLECYLFHEMLICVKERR---VPEHHYDPQMVK
          	**:*.* ******:* :*****: ***:***:** ********::    .    :     

MG00134.1 	RKPRCTLKGSILIKKHLNEVSETG-------------SIDENVLTLNLSVAELSQFHLRF
NCU07565.1	KTGRCTLKGSILIKKHLNGVSETG-------------SVDENILTLSLSVNELPQFHLRF
FG06762.1 	KTTRCTLKGSILIKKHLNEVSETG-------------NIDENILTLSLSVVELPQFHLRF
AN5167.1  	PRPRCTLKGSILIRKHLKTIEDVAGMGTSSVIKHELTSLDEPVLTLHLSVSELPCFYLRF
          	   **********:***: :.:... .:::  . . :.:** :*** *** **. *:***

MG00134.1 	QNRNQLKLWQQALMDLNAVETSPVRSPDYERGEFSEAEED-EWQRSQQQGQRTSSLASST
NCU07565.1	ENRNQLKLWQQALLDLNAVETSPMRSPEYDR-EFSEADED-EWNRSAGSKQRVSSQASS-
FG06762.1 	ENRNQLKLWQQALLDLNAVETSPVRSPEYDR-GESETDEELDWQRSR--PQRVSSVASS-
AN5167.1  	PNRSQLDIWRRALLDN---TAESLRSPELDFDRHSGVEED-DYRNGN--MKRQASLNSS-
          	 **.**.:*::**:*     :..:***: : .  * .:*: ::...    :* :*  ** 

MG00134.1 	WNGPKSTTTAPTEYTSVAKSP-LFPPVHVPIDVVVVVPISSSMQGVKINLVRDALRFMVH
NCU07565.1	WGGARSATTAPTEYTNV-RSPGLLPSIHVPVDVVVVVPISASMQGVKINLVRDALRFMVS
FG06762.1 	WNGARSTTTAPTDYTNFPRSPAMSKSVHVPIDVVVVVPISSSMQGVKINLVRDALKFMVN
AN5167.1  	HGAARSNNTAITDYTNMGVESVLSPSIHIPVDIVVVIPVSSSMQGLKITLLRDSLKFLVQ
          	 ...:* .** *:**..  .. :  .:*:*:*:***:*:*:****:**.*:**:*:*:* 

MG00134.1 	QLGDRDRMGLVTFGSGGGGVPIVGMTTKSWHGWGGVLNSIKPVGQKSHRADVVEGANVAM
NCU07565.1	SLGDRDRMGLVTFGSSGGGVPVVGMTTKAWPGWSNVLASVKPVGQKSHRADVVEGANVAM
FG06762.1 	TLGERDRMGLVTFGSGGGGVPIVGMTTKAWPGWSNILTSIKPVGQKSHRADVVEGANVAM
AN5167.1  	NLGPRDRMGLVTFGSSGGGVPLVGMTTKSWGGWSKILSSIRPVGQKSLRADVVEGANVAM
          	 ** ***********.*****:******:* **. :* *::****** ************

MG00134.1 	DLLMQRKYNNPIATIMLISDASTSDADSVDFVVSRAEAAK2ISIHSFGLGMTHKPDTMIE
NCU07565.1	DLLMGRKYNNPIATIMLISDASTSDADSVDFVVSRAEAAK2ITIHSFGLGTTHKPDTMIE
FG06762.1 	DLLMQRKYNNPIASIMLISDASTSDADSVDFVVSRAEAAK~ITIHSFGLGMTHKPDTMIE
AN5167.1  	DLLMQRKSSNPVSSILLISDSSTSDPDSVDFVVSRAEAAK2VGIHSFGLGLTHKPDTMIE
          	**** ** .**:::*:****:****.************** : ******* *********

MG00134.1 	LSTRTKASYTYVKDWMMLRECLAGCLGSAQTLSHQNVKLKLKLPEGSPAKFHKISGALQI
NCU07565.1	LSTRTKASYTYVKDWMMLRECLAGCLGAMQSLSHQNVKLKLKLPEGSPAKLGKISGALQI
FG06762.1 	LSTRTKASYTYVKDWMMLRECLAGCLGSMQTLSHQNVKLKLRLPEGSPAKFHKISGALQI
AN5167.1  	LSTRTKGSYLYVKDWMMLRECVAGCLGAIQTTSHQNVKLKLRLPEGSPAKFVKISGALHT
          	******.** ***********:*****: *: *********:********: ******: 

MG00134.1 	TKRATGRDAEASLGDLRFGDKRDILVQLVILPDTASEDQLPQDPWETIVSGLEALGGPMD
NCU07565.1	TKRATGRDAEANLGDLRFGDKRDVLVQLTIMPDTSSEEPQSHAYWDTVVSGLEAIGGPMD
FG06762.1 	TKRATGRDAEASLGDLRFGDKRDVLVQLVILPDNTSQEQLPQDPWDNIVSGLEALGGPMD
AN5167.1  	TKRATGRDAEAALGDLRFGDKRDVLVQLVIPPDNATHETPPQDPWESLVSGLEALGGGLD
          	*********** ***********:****.* **.::.:  .:  *:.:******:** :*

MG00134.1 	PEEQRTVSVEEVPLIQADLNWGDILRDGTLMHMPRPSLLAITMLPATNHQKSKSWQNTPP
NCU07565.1	DEDQRAMSVEEVPLIQADLSWGDILREGATQHT-RPSLLAITMLPATPGSK-KHWTASPA
FG06762.1 	NDNERTLSVEEVPLIQADLSWGDILREGTVTHMPRPSLLAITMLPVSGSSK-KPWQNSPP
AN5167.1  	GDDQRVLSVEEVPLIQADLTYGDLLREGHLTHSPRPSLLAITMLPPSPRHK-GGRPSTPP
          	 :::*.:************.:**:**:*   * .*********** :   *      :*.

MG00134.1 	IPPHPHIVQRRMELLTSDMLTRALTLVSRGQHDRAHTLLNETRSILKGLGKGGLPPVPPA
NCU07565.1	IPPHPYIVQRRMEILTSDMLTRALTLVSRGQHDRAHTLLSETRSILKGLGKGGLPPVPPA
FG06762.1 	IPPHPSIVQRRMELLTSDMLTRALTLVSRGQHDRAHTLLNETRSILKGLGKGGLPPIPPT
AN5167.1  	IPPHPSIVQRRMELLTSDMLTRALTLVSRSQHDRAQHLLNETRSILKGLGKGSLPPLPPP
          	***** *******:***************.*****: **.************.***:**.

MG00134.1 	----ASRSQPSTPHPNHEGSP-TSLTPERKHSPSPTASATSHYPPHMTQAMMRRPSTDAL
NCU07565.1	PGGPPTKSLPSTPLRTHSPIP---ATPDRKNTPSPTMANHPMGLGG-GFTISSRRSNDAL
FG06762.1 	--GKPNPSAPSTPHPGNDASPGLSPTPDRRRSPSPPASAISGTNGPPSSQLGRSRSTDGL
AN5167.1  	----AAKGLAEPESRGETPTS---------DSPKSSFASHS------------SAASDTA
          	    .  . ...    .   .          :*... :  .              :.*  

MG00134.1 	SALHGGPGSSGIDVQTVSALDSELMSALEWIGHPAVFGRDSRKAVLQAIGVISSQRAFTF
NCU07565.1	GAMN--TGASAIDPTTVSALDAELESSLEWINHPAIFGRDSRKAVLQAIGVISSQRAFTF
FG06762.1 	GLS------AGIDANTVAALDAELESSLEWINHPAVFSRDSRKAVLQAIGVISSQRAFTY
AN5167.1  	TITP----VAAVDTQTMMALDGDLQAALEWINHPAVFGRDSRKAVLQSIGVISSQRAYTF
          	         :.:*  *: ***.:* ::****.***:*.*********:*********:*:

MG00134.1 	RTPIEALWAGRISGVKKLTEHSREWREEGGGEGGIMEES
NCU07565.1	RTPIESLWAGRVSGIKKLTEKSQEWTVEGGGEGGIMEEA
FG06762.1 	RTPIEGLWACRVSGVKNLTEKSREWREDGGGEGGITEES
AN5167.1  	RSPSEEHWAQRISGVRRLIERSKEWRETG--DDALTEE-
          	*:* *  ** *:**::.* *:*:**   *  :..: **
```
