# Supplementary material for: Patterns of Intron Gain and Loss in Fungi
Source: PLoS Biol. 2004 Nov 30;2(12):e422. doi: 10.1371/journal.pbio.0020422 (PMC532390; doi:10.1371/journal.pbio.0020422)
Supplement: Table S1 — Also available at http://genes.mit.edu/NielsenEtAl/. (4.3 MB ZIP). [file pbio.0020422.st001.zip › NielsenEtAl/html/1085.html]

AN4793.1.NCU00554.1.MG03051.1.FG08498.1


```
 CLUSTAL W (1.82) Multiple Sequence Alignments - Introns Inserted


Sequence 1: NCU00554.1	362 aa
Sequence 2: MG03051.1	450 aa
Sequence 3: FG08498.1	364 aa
Sequence 4: AN4793.1	363 aa
Alignment Length: 453 aa
Number Identitical Residues: 230 aa
Alignment Score (without introns) 10428


MG03051.1 	MTSQFPTKKCG1VLGATGSVGQRFILLLQQHPSFTLVAVGASSRSAGKAYKDAVRWKQSS
NCU00554.1	-MSQFPTRNVG1VLGCTGSVGQRFILLLQNHPVLKLVAVGASSRSAGKKYRDAVRWKQST
FG08498.1 	MASQFPTRKCG1VLGATGSVGQRFILLLAQHPYLTLHAIGASSRSAGKKYKDAVRWKQAS
AN4793.1  	-MASFPKKKCG1VLGATGSVGQRFILLLAEHPFLELHAIGASERSAGKKYKDAVRWKQST
          	  :.**.:: * ***.************ :** : * *:***.***** *:*******::

MG03051.1 	PMVQAVADLVVRECRPSEFADCDVVFSGLDSDVAGDVE~MAFLKADLAVFSNAKNYRRDP
NCU00554.1	PISAEFGDLVVRDCKASEFADCDIVFSGLDSDVAGDVE~KEFQHANIAVFSNAKNYRRDP
FG08498.1 	PMG-EIADMVVRECKAEEFQDCDVVFSGLDSDVAGDIE1MAFIKADIPVFSNAKNYRRDP
AN4793.1  	PMSEKLSDLVLRNCKAENFTDCDLVFSGLNSDVAGDLE1MEFIKADIPVFSNAKNYRKHP
          	*:   ..*:*:*:*:..:* ***:*****:******:*   * :*::.*********:.*

MG03051.1 	LVPLVVPTVNLPHLSMIPHQRKHHGLNKGFLVCNSNCAVIGLVIPFAALQARFGPIETVS
NCU00554.1	LVPLVVPTVNLNHLDLIPHQRKTLGLEKGFLVCNSNCAVIGLVAPFAALQARFGKIDTVS
FG08498.1 	LVPLVVPTVNLPHLDLIPHQRSVHKLNKGFLVCNSNCAVIGLVGPFAALQAAFGPISTVS
AN4793.1  	VVPLVVPTVNPNHLDLIPHQRKHFGLKKGFLVCNSNCAVIGIVIPFAALQAKFGPVEEVE
          	:*********  **.:*****.   *:**************:* ******* ** :. *.

MG03051.1 	VVTMQAVSGAGYPGVSSMDIIDNVVPYIAGEEDKLESEARKILGGINAEATAFEEQTSMR
NCU00554.1	VVTMQAVSGAGYPGVSSMDIIDNVVPFISGEEDKLETEAQKILGSLNADATAFEDQKQLR
FG08498.1 	IVTLQAVSGAGYPGVSSMDVIDNIVPFISGEEDKLETEARKILGRLDDNGTAFIDQEGLR
AN4793.1  	VFTEQAVSGAGYPGVPSMDIMDNVIPFISGEEDKLENEAQKILGSLTADATAFDEQQGLR
          	:.* ***********.***::**::*:*:*******.**:**** :  :.*** :*  :*

MG03051.1 	VSASCNRVPVLDGHTACVSLRFANRPPPPAEAVKAAMATYVSEAESLGCPSAPSPSIMVF
NCU00554.1	VSAACNRVPVLDGHTACVSLRFAQRPPPSAEEVKEAMRSYVSDAQKLGCPSAPEPPIKVF
FG08498.1 	VSATCNRVPVMDGHTACVSLSFERKPSPSAEEVRKALRDYKCEAQALGCPSAPEPAIKVF
AN4793.1  	IGATCTRVGVTDGHMAFVSLRFKNRPAPSAEQVVQAMREYQSEAQKLGCPSAPAEAIKVF
          	:.*:*.** * *** * *** * .:*.*.** *  *:  * .:*: *******  .* **

MG03051.1 	--EEADRPQPRLDRDLSRGYTVSVGRVRE-DESGIFDIKFVALSHNT1VIGAAGSSILNA
NCU00554.1	--DEADRPQPRLDRDLCKGYTVSVGRVRE-DESGIFDIKFVALSHNT1VIGAAGSSILNA
FG08498.1 	GDDEPDRPQPRLDRDLGRGYTVSVGRVRE-DEAGIFDIKFTALSHNT1VIGAAGSSILNA
AN4793.1  	--DEPDRPQPRLDRDINRGYTVSVGRVREGNQGGYFDIRFAALSHNT~VIGAAGSSILNA
          	  :*.**********: :***********.::.* ***:*.****** ************

MG03051.1 	EAAVLKGYL2LKWYRRGNIFDLHTRLHAHMSQVSSKLMSNQKTSYKFAPLTITAQRSRYR
NCU00554.1	EAAIFKGLI~--------------------------------------------------
FG08498.1 	EAAILKGYI~--------------------------------------------------
AN4793.1  	EVAVIKGYI~--------------------------------------------------
          	*.*::** :                                                   

MG03051.1 	SKRDSFFGVRLGNTIHRCYPGPEWALRRDEPKFQCDG
NCU00554.1	-------------------------------------
FG08498.1 	-------------------------------------
AN4793.1  	-------------------------------------
          	
```
