# Supplementary material for: Patterns of Intron Gain and Loss in Fungi
Source: PLoS Biol. 2004 Nov 30;2(12):e422. doi: 10.1371/journal.pbio.0020422 (PMC532390; doi:10.1371/journal.pbio.0020422)
Supplement: Table S1 — Also available at http://genes.mit.edu/NielsenEtAl/. (4.3 MB ZIP). [file pbio.0020422.st001.zip › NielsenEtAl/html/1086.html]

AN9094.1.NCU02109.1.MG01320.1.FG00872.1


```
 CLUSTAL W (1.82) Multiple Sequence Alignments - Introns Inserted


Sequence 1: MG01320.1	504 aa
Sequence 2: FG00872.1	508 aa
Sequence 3: AN9094.1	505 aa
Sequence 4: NCU02109.1	487 aa
Alignment Length: 518 aa
Number Identitical Residues: 284 aa
Alignment Score (without introns) 13004


MG01320.1 	-MDTVKKVLGLSGSSATP----PSDEQLTQLREKYTKAGQEQVFTFYDSLSAGEQAALFQ
NCU02109.1	--MAVDP--------------VPTPEQVSELKDKYTNAGQGQVFTFYDSLSSEEQAQLYK
FG00872.1 	-MEAIKQALHLGKGSDAPAE--PTPEALNELKEKYTKAGQEQVFTFYDSLSSAERGTLYQ
AN9094.1  	MAAAIKSLVDKFQGHSEPALRQPSAEEVRELKQKYEQAGQSQVFAFYDQLSQKEQAQLFH
          	   ::.       .   .:   *: * : :*::** :*** ***:***.**  *:. *::

MG01320.1 	QLSQIDPDHINKITDRALNPPKTDNDGKAPTLEPLPESATASILDSDPKDVEGWYNSGLD
NCU02109.1	QLAGFDPLYINKIAAKALTPQSSESE--KPTLEPLPDSARASTLDSDKQTQDEWWNRGLQ
FG00872.1 	QLSGFDPSHINDITQRALNPAKTSDE--PDRLEPLPESATASILDSGADDIAKWYDSGLD
AN9094.1  	QLSAFDPARINELADRALNPPKSETG--PISLEPLPEVATASILDSDPKDIQSYYNEGIK
          	**: :**  **.:: :**.* .:.       *****: * ** ***. .    ::: *:.

MG01320.1 	IIAKGKVGVVLMAGGQGTRLGSSAPKGCFDIGLPSSKSLFQIQAERILKVQQLAAKKAGA
NCU02109.1	LIADNKVAVVLMAGGQGTRLGSSAPKGCFDIGLPSHKSLFQIQAERIARLQVLASQRREQ
FG00872.1 	LISKGQVAVVLMAGGQGTRLGSSAPKGCYDIGLPSHKSLFQLQGERIAKVQELAAKKG--
AN9094.1  	LVADNQVAVVLLAGGQGTRLGSSQPKGCFDIGLPSHKSLFQLQAERIGKLQLLAKKTSG-
          	:::..:*.***:*********** ****:****** *****:*.*** ::* ** :    

MG01320.1 	DKPAVVPWYVMTSGPTRKPTEEFFEKHSFFGLDKANVQFFEQGVLPCISNDGKILLESKG
NCU02109.1	AGSPVVPWYVMTSGPTRKATEDFFKTNNYFGLSPDQVIIFEQGVLPCISNDGKILLESKS
FG00872.1 	-SNAVVPWYVMTSGPTRGPTEKFFQKNNYFGLSQENVKIFEQGVLPCISNDGKILLETKG
AN9094.1  	-KDAVIPWYVMTSGPTRKPTEEFFQQHNYFGLDKSNVFIFEQGVLPCISNEGKIMLESKS
          	   .*:*********** .**.**: :.:***.  :* :***********:***:**:*.

MG01320.1 	K~LAVAPDGNGGIYQALVVSGVLDDMRKRGVEHIHAYCVDNCLVKVADPVFIGFSAAKDV
NCU02109.1	R~VAVAPDGNGGIYNALVDAKVLDDMARRGIEHVHAYCVDNCLVKVADPVFIGYCASQNV
FG00872.1 	K~VAVAPDGNGGLYNALVLSGVVDDMRKRGIQHIHAYCVDNCLVKVADPVFIGFSAALDV
AN9094.1  	K0VAVAPDGNGGIYQALLAAGVREDMRKRGIKHIHAYCVDNCLVKVADPVFIGFAASKKV
          	: :*********:*:**: : * :** :**::*:*******************:.*: .*

MG01320.1 	DIATKVVRKRNATESVGLILLKNGKPDVVEYSEIDKETAEATDAKLGEGVLKFRAANIVN
NCU02109.1	DIGTKVVRKRNATEPVGLILLKNGKPDVVEYSEIDDAVAAEEDPAQPG-VLRFRAANIVN
FG00872.1 	DIATKVVRKRNATESVGLILSKNGKPDVVEYSEIDQATAEETDPKQPD-LLRFRAANIVN
AN9094.1  	DLATKVVRKRNATESVGLILQKNGKPDVVEYSEIDKETAEAKDSKNPD-LLKFRAANIVN
          	*:.***********.***** **************. .*   *.     :*:********

MG01320.1 	HYYSFRFLESIPLWAHKLPHHVARKKIPHADLESGETVKPEKPNGIKLEQFVFDVFPMLE
NCU02109.1	HYYSFRFLKSIPEWASNLPHHIARKKIPYADLESGETVKPEKPNGIKLEQFVFDVFPLIE
FG00872.1 	HYYSFRFLDSIPQWAHKLPHHIARKKIPSADLESGETVKPEKPNGIKLEQFVFDVFPFLT
AN9094.1  	HYYSFDFFNSIETWVHKLPHHIARKKIPSVNIESGEVVKPEKPNGIKLEQFIFDVFPMLP
          	***** *:.**  *. :****:****** .::****.**************:*****:: 

MG01320.1 	LSKFACMEVRREDEFSPLKNARGTGEDDPDTSKHDIMDQGKRWVAAAGAVVTGEKADTGV
NCU02109.1	LSKFACMEVKREDEFSPLKNARGTGEDDPDTSKHDIMAQGRRWLEAAGAKFA-EGAEDGV
FG00872.1 	LEKFASLEVKREDEFSPLKNAPGTGEDDPDTSKADIMTQGKRWVEAAGAIVVGDKADVGV
AN9094.1  	LEKFASIEVRREDEFSPLKNARGTGEDDPDTSKRDIMNQGQRWIESAGGVVVTEGDAVGV
          	*.***.:**:*********** *********** *** **:**: :**. .. :    **

MG01320.1 	EVSP-----~---LVSY0GGEGLEAFKGKEIVAPAVLEKE-
NCU02109.1	EVSP-----~---LVSY0CGEGLQSYADRKVVAVDRIE---
FG00872.1 	EVSASDQLQ1SCANNSQ~GGEGLEKLNGNEITPPAVLERE-
AN9094.1  	EVSP-----~---LISY0GGEGLEFLKGRELKAPAVIEKEE
          	***.           *   ****:   ..:: .   :* ..
```
