# Supplementary material for: Patterns of Intron Gain and Loss in Fungi
Source: PLoS Biol. 2004 Nov 30;2(12):e422. doi: 10.1371/journal.pbio.0020422 (PMC532390; doi:10.1371/journal.pbio.0020422)
Supplement: Table S1 — Also available at http://genes.mit.edu/NielsenEtAl/. (4.3 MB ZIP). [file pbio.0020422.st001.zip › NielsenEtAl/html/1087.html]

AN5607.1.NCU05620.1.MG04342.1.FG06417.1


```
 CLUSTAL W (1.82) Multiple Sequence Alignments - Introns Inserted


Sequence 1: NCU05620.1	1956 aa
Sequence 2: FG06417.1	1958 aa
Sequence 3: MG04342.1	1874 aa
Sequence 4: AN5607.1	1968 aa
Alignment Length: 1986 aa
Number Identitical Residues: 809 aa
Alignment Score (without introns) 46706


MG04342.1 	------------------------------------------------------------
NCU05620.1	MDESLEAHPGAAGHFAAEGAHSAFLGGLLASADTISRATSPGLPCSN-GSDQDDQKRYRP
FG06417.1 	MDENIGPRPSGHASMPPT-ANSSLLANFLP-YEPISRATSPGIPYPKTGTDEDDKKRYRA
AN5607.1  	--------------MDNPNSKTILTPDNFHNVHTISRATSPGGGPDGVNGDGEPKARVRP
          	                  .: :    .   .  . : :::..      . . . .    .

MG04342.1 	----------------------------------MAEDFSPGALHWTRELQGWLNLKFEM
NCU05620.1	RTFSYFRLLPFEVEEESHRDAALNGILKNLYVSIMAEDFSPGAVHWTRELQGWLNLKFEM
FG06417.1 	RTFAYFSQLPFEVEEEAQRDAALQGILKQLYIAVRAEDFSPGALHWTKELQGWLTLKFEM
AN5607.1  	RTYPYFKYLPYQTEDEAQRARYLRDILTQLYIAVESGDFSPGAVHWTRELRAWLSLKFDP
          	 : .     . . ...:       .  ..   :  : ******:***:**:.**.***: 

MG04342.1 	TRELRSKLVQVYYHLALAPGLDTNTADRFTKMLVTLTR2RKHYLKPVEDLVLDWRPLWKE
NCU05620.1	SRELRARLAHLYFHLALAPGLDPNTADRFARMVVTLTR~KRHYLKPGQDLTLDWRPLWKE
FG06417.1 	TREQRAKLAKLYYSLALAPGLDATASDRFLRMVLSLTR2KNHYLKPGEDLVLEWRPLWNE
AN5607.1  	TRSDRIKLVKLYYELSLAPGIDPNVAERFSSMFMLLTK2RKHYLRPIKDLTLDWRPLYRE
          	:*. * :*.::*: *:****:*...::**  *.: **: :.***:* :**.*:****:.*

MG04342.1 	IKALVLPSEC-PSHQSRRRRSQRHLLRLCLHSQIYFDPSSRREMLDEFLPFFSTSDLTNA
NCU05620.1	LKGLVFPSET-APHQGSRKRAQKHIMKLCLHGSVYFDPKERRAILDELLPYFSTSDMSNA
FG06417.1 	IKAWVLPSEV-PAHQSNRKRSAKQLLKLCTHAHTYFDPSERRAMLEEFLPFFSVNELPNA
AN5607.1  	LKAFVLPTESGLVHSSNLKRNVKTLTKLCAFIQLYVDPCELPAMLEEFLPHYSTSFSEGA
          	:*. *:*:* .  *..  :*  : : :** .   *.** .   :*:*:**.:*..   .*

MG04342.1 	YIVLALVTTMLPSTPSASTDELSQPSEYFPTLFHIWSLVNRSKIFDQCFIDVFSRMSRDY
NCU05620.1	YIVAGAINTLLPTGPAPPTEPQSQPSDFVPTFFHLWSLMTRSKAFDVGFIDIFSRMARDY
FG06417.1 	FIVAGVLNALLPSHPAPINEPQSQPADIFPTLFHLWSIINRSKAFDIFFIDLLSRIARDH
AN5607.1  	FVVVGLINLLAPTTPPPESREDLLPQHYMPTYFHLWSLVSRSKTFDQTFLDFFSRLARDS
          	::* . :. : *: *.. .     * . .** **:**::.*** **  *:*.:**::** 

MG04342.1 	LGCSDVPFSEHGIFTKDQSDYIFTAILRLTEIPVGQANSPYTT-LDYSSGLAVYLEKDKK
NCU05620.1	LSCTHVPFTEHGIFTREQSDLIFTAILRLTEIPVGQANSPYAS-LDYSAGLGIYLEKDKK
FG06417.1 	IACSYVPFGSHGIFSKDQSDLIFTAILRLTQIPVGQANSPYTP-LDYLSGAGIYVEKDKK
AN5607.1  	LPAGHIPFSEYGLFTKEQSSLIFTAILRLLEIPVGQSSSPYSALVDISSGLGIMLDRDSR
          	: .  :** .:*:*:::**. ******** :*****:.***:. :*  :* .: :::*.:

MG04342.1 	KYPVPYMIARWVVHSLSPHCLGKDSSILLSLEGLMESIDTFFHPSNHGSWAQFLGQLTYY
NCU05620.1	KYPVPYMVARWIVHSISPLCLDQESSVMSNLEGLMESIDTFFHPSNQGSWSMFLAQLTFF
FG06417.1 	KYPVAYMISRLIVSSLSPACLKNEDSIISHLEGFMESIDTFFHPSNQGSWTTMLGQLTLY
AN5607.1  	KHPVAHHIARWIVMSLSPACLDKEESILSQLEGLIQAVETFFHPSNSGSWTKTLAQLVYY
          	*:**.: ::* :* *:** ** ::.*::  ***::::::******* ***:  *.**. :

MG04342.1 	LTDIFVSRWNREQSGELNLPADRKINDELKKRFVLSLREVTFMGLFAKSSKVANYYYSTL
NCU05620.1	LTDIFVSRWNREQSGELDIPADRRINDDLKKRFVLSLREVTFMGLFSKSSKVVNYYYTAL
FG06417.1 	LTEAFVSRWNREQSGELDLPEERKINDELKRRFVMALKEVTFMGLFSKSSRVSYYYYSAL
AN5607.1  	LTDFFVMRWNREQSGEMEVPQERRLTEPLKRRFVLCLRDVIFMGIYSKSATAMSFSLSTL
          	**: ** *********:::* :*::.: **:***:.*::* ***:::**: .  :  ::*

MG04342.1 	QGLAYLQPDLILPGALQRFYPSLQGLVEVHRTTASLCGLQMITNIMARQKGFRCHLTALM
NCU05620.1	QGLAYLEPDLMLPGALQRFYPSLQGLVEVHRTTSSLCSLQMIANIMSKHKGYRCHITALL
FG06417.1 	QGLAYLEPDLVLPGALQRFYPSLQGLVEVHRTTSSLNGLQMIANVMSKNKGYRCHITALL
AN5607.1  	QGLAFLEPHLILPGALQRIYPSLQGLVEVHRTTSSLRALQILARIIARTKGYRCHLTTLL
          	****:*:*.*:*******:**************:** .**:::.:::: **:***:*:*:

MG04342.1 	ALALPGIDANDLGKTQYTLNFFQSVAYSIPLVSLIKEHDDVHDTSLAMQFVQGEMDRMEQ
NCU05620.1	ALALPGIDANDLNKTQYTLNFIQSVAYSIPFVPLVDENNDIHDTSLAMNWVQGEMERMER
FG06417.1 	ALALPGIDANDLNKTQYTLNFIQSVAYSIPMVPLVKEGSHIHDTALAMEWVQGQMDRMER
AN5607.1  	GMALPGIDANDLEKSLHTLTFIQAACYNIPMTDLTKGRDEINCDMLAMQWIPGEMERMEQ
          	.:********** *: :**.*:*:..*.**:. * .  ..::   ***::: *:*:***:

MG04342.1 	EGQDVKINYDEELTDEMEEAIVRSSTAGFGEFIIALLGKVFTLLENLPDSSHLRTGSPED
NCU05620.1	EGQDVKINYNEELSDEDEANILRSSTAGLGEFVMALLGKVFALLENLPDASHLRTGSPED
FG06417.1 	EGQNVKFDYKNELSDEDEANILRSSTTGFGEFILTLLGKVFTLLENLPDANQVRGGTPED
AN5607.1  	EGVEVQLNYDTELSDETEEMILRSSTCGFGDFIISFLGRVFTLLENLPDVSRVRNGSPEE
          	** :*:::*. **:** *  *:**** *:*:*::::**:**:******* .::* *:**:

MG04342.1 	NVINTLPAALTPLFASLSPELFEIALEKLSNFVSTHVVHQARDAMAWICNALCKVNPEKT
NCU05620.1	NVINTLPAALTPLFASLSPQLFDSALEKLANFVSTHVVHQARDAMAWICNALCKVNPEKT
FG06417.1 	NVINALPAALSPIFASLSPELFDMALDKVATFVSSHVVHQARDAMAWILNALCKVNPEKT
AN5607.1  	NIVNTLPATFMPLLSSLSPEYYEIALTKVVDFVSNHVIHQARDAMAFICNAVCKVNPEKA
          	*::*:***:: *:::****: :: ** *:  ***.**:********:* **:*******:

MG04342.1 	LKVFIPMLIANIRSEIDDNGAASDRSSGTDVLPRDRALVWHVSMLSMCVVHVGNEVLRYR
NCU05620.1	LKVFIPMLIVNIRNEIDYNGAASDRSSGTEVLPRDRALVWHVSMLSMCVVHVGSEVLKYK
FG06417.1 	LKVFIPMLVVNIRNEIDYNNAASDRSSGTDYLPRDRALVWYVSMLAMAVVHVGSEVLKYR
AN5607.1  	LKRFIPVLIQAIRTEIDDNGAGSTRTTGTDVLPRDRGLVWNVSMLSMCVVHVGDAVLAYK
          	** ***:*:  **.*** *.*.* *::**: *****.*** ****:*.*****. ** *:

MG04342.1 	AELLGIAQYMQEKCRGLPTIHISNFVHHLLLNLTHTYTLDNALFEPDVIAKGLGVEHWGR
NCU05620.1	DELFGIAKYMQEKCRGLPTIHISNYVHHLLLNLTHTYPIDNALYEPDVIARGIDVSDWGR
FG06417.1 	DELLGIADYMQEKCRGLPTILISNYIHHLLLNLTHTYPIDHALYEPEVIQRGLDVDDWGK
AN5607.1  	KELFDIAVYMQQKCRGIPTVHVSNFIHHLLLNLTGTYTSDYSLYEPDVVAKGIQPEHWCY
          	 **:.** ***:****:**: :**::******** **. * :*:**:*: :*:  ..*  

MG04342.1 	VTAPADLTIHWHRPSREEIEFAVELFELQAHAASKRLETLMSGESPIARSAKNKEWSDEV
NCU05620.1	TTEPSELTIKWHRPSPAEVAFAVELFESEARGATERLMQLMSDDPPVSRKGKNKEWSDEL
FG06417.1 	TTAPADLSIRWHQPSPAEIDFAVELFASQTKSAKDQLELLMSDNPPVSRTGKNKEWSDEV
AN5607.1  	RPDPQNLTVKWHVPKRQEIEFAAELFQNQAESALKQLAALTDENSNIRRDGIGKEWSDEV
          	 . * :*:::** *.  *: **.***  ::..* .:*  * . :. : * . .******:

MG04342.1 	SRVLSRIRLVISGVSALFDPERVVNHKANGHVAEQDDSMDTDGDT------AMDEDDDPL
NCU05620.1	SRSLTALRLLISGVSTLFDPVRASGEATG-SSGNQD--TDAAGDT------IMEEDNDPL
FG06417.1 	SRLMQQIRLVTSGMATMFDPERAAGIMTGNTEDDHD--VAREDDD------EMMIDEDPL
AN5607.1  	IRNLVLLRLIISGVSVLFDAKAASKTKGSRTNGVSDKAEDVEMTDGVIGTGPEDEEADSS
          	 * :  :**: **::.:**.  .     .      *.:       .  .:.    : *. 

MG04342.1 	AEAADDDELKRKYIYPAGYLLDRSSPLYEHIHQLREDIGRTLSRTHRFLIAEQEDDVPCF
NCU05620.1	AEIADDDELKRTFRYPAGYVLTTDDPLYHRVHDLREEVGRTLSQTHSFLNANQQDDVACF
FG06417.1 	AEVAEDEELRPQFRYKAGYALKFSDPAYSRIHDLREELGHLLTKTHSFLNENQEDDVNSF
AN5607.1  	LDTSEEDTVRETYTYPTGYPLEENDPIYVSIHDIRERAGWTLHKVHRYLCEKQEDDVPCF
          	 : :::: ::  : * :** *  ..* *  :*::**  *  * :.* :*  :*:*** .*

MG04342.1 	TALYAAYRTWITDVGIERSAHPLERLLRLYKADVGPFKVSGLRKPYPRPLLVKRADAYQL
NCU05620.1	TALYSAYRTWITDVGIERSAHPLERLTRLYKADISPFKISGLRKVYPRPLLIKRADAYQL
FG06417.1 	TALYAAYRTWITDVGIERSAHPLERHVRLYKSDIAAFKIKGLRKVYPRPLLIKRAEAYQL
AN5607.1  	SALYSAYKCWFVDVGIERSAHVLDRVTRLLLADIHPYKMSGIRKDYPRPLLIRRANMYHL
          	:***:**: *:.********* *:*  **  :*: .:*:.*:** ******::**: *:*

MG04342.1 	QRVKYNASYRQKGELDKQLLLDLAESCTSVYADVRRVAQGSQDSSLKVLIGGRPLVIPML
NCU05620.1	QRVKYNSAYRTSSELDRRLLLDLAESCTSSYADVRRVAQSAQDSSLKVIIGGRPLVIPVI
FG06417.1 	LRRKHNASSRQKSELDKRLLLDLAESSLSIYADVRRVAQSAQDSSLKSLIGSKPLVIPVI
AN5607.1  	QRLRHNAAPRCRSRLDEILLLDIAESCVSAYTETRRNAQTAGESALKVVWGSRLLVIPPL
          	 * ::*:: *  ..**. ****:***. * *::.** ** : :*:** : *.: **** :

MG04342.1 	LKKLRIALDEVNHDRIKGAMYTLLFTSLLKTLVKDWRFASDFMRMYLETANVDKASIQAL
NCU05620.1	LNALRKALDDMDHDRIKGAMYTLFFTTLLKTCMKDWRFAPELMRLYIETASVDKTSIQNL
FG06417.1 	LERLRKALDANDHDRIKGGMYTLLFTSLLRTLVKDWRFAPEAMRLYIETAGIDKPSIQNL
AN5607.1  	LRALQNGIKENDYARIKGSLFSLLLSSVARTVGRHWKYAPTLVRAFIDASAVDRPSVQRI
          	*. *: .:.  :: ****.:::*::::: :*  :.*::*.  :* ::::: :*:.*:* :

MG04342.1 	GSTALYSLMDFGRPLERLILKDDSIIDGIKPA-DDCSARIKKRHKFILERRAKVEAKKSA
NCU05620.1	GATALYPLIDFGKPFERMILFDRSVADTIRPP-QDFSASIQRRHEFIKERRAKVEEKKAA
FG06417.1 	GSSALYTLIDFGKPFERMIIVNDELVDTIKPT-ADVSAAIESRHQFILQRRTRVEKSKAS
AN5607.1  	CSSVVYQIMDYGRPMERMAILDRDLVESIAPTSQNVDEQIQQKRNSLNNKRALIEKKKAA
          	 ::.:* ::*:*:*:**: : : .: : * *.: : .  *: ::: : ::*: :* .*::

MG04342.1 	LGLELTEMARVSHWKVATRCILFATNLSSRFDTIAPPEYIELVVKGTNDPHPGLRSGYQS
NCU05620.1	LGLELINKAKASHWKVATRCAMFALNMSLRFETLAPPEFIELVVTGTNDPHPGLRSTYLA
FG06417.1 	LGLELTQRAKGAHWKIATRCAVFATNLCLRFHTVAPPEFIDLVAQGTNDPHPSLRGYYLS
AN5607.1  	LAEELVDLAREAHWKVASRAATIVISMGLRFDYIASERLVELVTMGSIDDHPGLRGMYSQ
          	*. ** : *: :***:*:*.  :. .:  **. :*. . ::**. *: * **.**. *  

MG04342.1 	AFSTLFTTIDMRAVYDHDYRKFLAE-EVKDYQTVEVDVPQDDPQWTDRFLKGFEDYDNTP
NCU05620.1	AFSGLCTSIDMRAVYQHSYEKYLLEQAVGD-NKFEVPVPEDDPEWTQKFLAQFENTD-EI
FG06417.1 	AFTSVFTAVDMRAVYGHDYRNYLLEKEVGDRNRVQVAVEKGDTEFTYNFLEAFKQPE-GA
AN5607.1  	GLTALFTMIDVRAICNHDYKSYILGNQNFP-AKIKVATKRYEKGWTEEFLASFANPE--T
          	.:: : * :*:**:  *.*..::  .       .:* . . :  :* .**  * : :   

MG04342.1 	EYYVDTDHPGWLVWGKKFLGMRSRPLPFEDYDDVEKAAREQIGKLLDKEWFKQCFEYMKQ
NCU05620.1	QYFVDSDYPGWLVWGKKFIASSANPEPFLGYDEIETAARQQIGKLLTRDWFKQCFEYLKQ
FG06417.1 	SYMVDADHPGWLVWGKKFTAYRAKPLPFNAYDEVESALRDQMGQILNRDWLSECFSYLKQ
AN5607.1  	EYYIDHDFPGWLVWADHMPGYKPNVERDIEYDQIEWQIRSRMGKLFDRAWFKKFFMYLKQ
          	.* :* *.******..:: .  ..      **::*   *.::*::: : *:.: * *:**

MG04342.1 	EPRDSVNDRFRMQNAILLIHLFDLMN-YNVTAVKLEDIIDLVKDVYGDGSDKHQHRATSE
NCU05620.1	EPRDAGADRFRMSNVVLLMQVFDLMN-YGQTAATFDDIVELTKEIYGDGGDKHQHRATAE
FG06417.1 	EPRDTTTDRFRMSNVYLLMHVFDLMH-YGKTAITLDDVKELVKEVFGDGNDKHQHRATSE
AN5607.1  	EPRDPSADKFRMSCAMLLLYAFELMLRDGLTAATFEDIKEEIEAVYEDGSDKHQHRATAE
          	****.  *:***. . **:  *:**   . ** .::*: :  : :: **.********:*

MG04342.1 	ILGALLSGSAEDPIEMRAKIWKFAAPWMMKILAEDLTPDNLSYWMTCLHVIADSKDPRKF
NCU05620.1	IVGAMLSGSSDDPREFRDKVWAFAAPLMLKVIADDLTPDNLQYWMTCLHLTVDSKDPRRS
FG06417.1 	IIGALLAGSSDDPPEIRNLVWEYAAPFMLKIFADDLTPDNLQYWLTCLHLVLDSKDPRRS
AN5607.1  	ILGALVSSVTDTSVEKRTLVWEYAFPIVQKIFIEGLTPENSGYWTTFLHMILQCRDPRRV
          	*:**:::. :: . * *  :* :* * : *:: :.***:*  ** * **:  :.:***: 

MG04342.1 	PEFMKSIGAFRLDMSSNAAFKESSQLQLLELLINNSGWHFRHEKPILEDFLAHIDHPYKS
NCU05620.1	KELIDPLANFRLDMNSNAAFKESAKIQLLEFIINDAGWHFRRDKPILEDFLAHIDHPYKS
FG06417.1 	HEIVDTLRAFRLDMTSNAAFKESSKVQLLEFIVADGGWHFRHDQPILDDFLAHIDHPYKA
AN5607.1  	WPLVDWLASFRLDMTTNAAFKESSKINLLHQSIIDAGWHFRLEKPIVQDYLAHLDHPYKG
          	  ::. :  *****.:*******::::**.  : :.***** ::**::*:***:*****.

MG04342.1 	VRESIGRVIATIYRSRYHESFPNVDTLLRENKAASSIGIRPYEPNDEFKATIIEVFERLE
NCU05620.1	VRESIGRVIATIYRTRYHESFPNVWSLLEQNKSASSIGIRPYEATEDFTATIREVFDRIE
FG06417.1 	VREAIGRVLSVIYKTRYHESFENVSKLLEQNKAASTTGIRPYQPTEEFAATIKDVFSRLE
AN5607.1  	VREAMGQTLATIFRTRYHESYPDVKSLLADQEASSSVGSYPYSPDTDFRQMINDIFTRIE
          	***::*:.::.*:::*****: :* .** :::::*: *  **..  :*   * ::* *:*

MG04342.1 	KWRHEREPGQQTPSSYTSGSKTVLAWLDTMLSSHECTQLVQFFPDPFINQLLHMMDVKED
NCU05620.1	KWRHERTPGQQTPSSYTSGSKTVLVWLDSMLASQECIQLVPFFPEPFIDQLLHMMDVKED
FG06417.1 	KWRHERTPGQQTPSSYTSGSKTVLMWLDCTLSSHECIQLVPFFPTPFMEELLHMMDVKED
AN5607.1  	EWRQERTPGQQTPSSYTSGSKTVLLWLDSTLSSHECTQLAPFFAEVFTGQLLHMMDVKED
          	:**:** ***************** ***  *:*:** **. **.  *  :**********

MG04342.1 	PELMRLAYHVYRHLPNIPLREGEDDAFIKALIKVGRSASSWHQRLRALVNMQVVYFRRLF
NCU05620.1	PELMRLAYHVYRHMPNIPFRSGEDDAFIAALIRVGKTAASWHQRLRALVNMQVIYFRRLF
FG06417.1 	PELMRLAYHVYRHLPNIPFRDGEDAEFIDALIRIGKTSTSWHQRLRALVNMQVIYFRRIF
AN5607.1  	PELQSLAYHVFRHLPNVPYPAEENSDFIKTLIRIGQTSPSWHQRLRVMINIQIIYFRRLF
          	***  *****:**:**:*    *:  ** :**::*:::.*******.::*:*::****:*

MG04342.1 	LTAAPQRALLFDAVSNMLADAQLEVRDGACATLAGMIRCSPHVYREPIIHNLKARFEEQL
NCU05620.1	LTQPAQRQMLFDAVGDMLSDVQLEVRDGASATLAGMIRCSPERIRNPIIEQLKARFQLQL
FG06417.1 	LTRSAQREALFTAVSDMLGDPQLEVRSCASTTLAGMIRCSPRRIRDPTIAHLKARFEDEL
AN5607.1  	LLSPSDRDKLFECVASMLEDPQHEVRAGASATLSGMVRCSPEFLRKEIVDRFKKRFTQIL
          	*  ..:*  ** .*..** * * ***  *.:**:**:****.  *.  : .:* **   *

MG04342.1 	SQNPMPKSKRALQASG-----ASSGTETP-VDVQAQIRARHAAVLGLGALIEAFPYLTPP
NCU05620.1	EMNPMPKRK-------------LPGTDTP-VDVQKQIIRRHAAVLGLGALIEAFPYATPP
FG06417.1 	ERNPMPKRNR-----------HLAGTDTP-VDIHKQITRRHAAILGLGALIEAFPYATPP
AN5607.1  	VENPLPKRPKMPRMASGLSSPGSSGANTPNPEHTRLVISRHGAVLGLGALIQAFPYSSPP
          	  **:**       :.. ::.  .*::**. :    :  **.*:*******:**** :**

MG04342.1 	PKWMPEVLALLARNAASDPGIVGKATKTILAEFKKTRQDSWGVDQK0YFTSEQLEDLEG~
NCU05620.1	PKWMPEVLAYLATHAASDPGVVGKATKGILAEFKKTRQDSWTVDQK0YFTSEQLEDLEV1
FG06417.1 	PEWMPEVLAMLARKAAADPGVVGKATKTILSEFKKTRQDSWTVDQK0YFTSEQLEDLEG~
AN5607.1  	PTWIPEALTTLSVRAASDPGIVGSSVKSIISEFKKTRQDTWHIDAK0AFTSDQLEDLSG~
          	* *:**.*: *: .**:***:**.:.* *::********:* :* *  ***:*****.  

MG04342.1 	VLWKSYFA-
NCU05620.1	MGNHGQMKR
FG06417.1 	VLWKSYFA-
AN5607.1  	VLWKSYFA-
          	:  :. :
```
