# Supplementary material for: Patterns of Intron Gain and Loss in Fungi
Source: PLoS Biol. 2004 Nov 30;2(12):e422. doi: 10.1371/journal.pbio.0020422 (PMC532390; doi:10.1371/journal.pbio.0020422)
Supplement: Table S1 — Also available at http://genes.mit.edu/NielsenEtAl/. (4.3 MB ZIP). [file pbio.0020422.st001.zip › NielsenEtAl/html/1088.html]

AN4376.1.NCU01195.1.MG08074.1.FG07174.1


```
 CLUSTAL W (1.82) Multiple Sequence Alignments - Introns Inserted


Sequence 1: AN4376.1	459 aa
Sequence 2: FG07174.1	451 aa
Sequence 3: NCU01195.1	454 aa
Sequence 4: MG08074.1	435 aa
Alignment Length: 469 aa
Number Identitical Residues: 252 aa
Alignment Score (without introns) 12024


MG08074.1 	MTVLPLEPEFSQAYNE1LAGTLEDSTLFEKYPELRTALSVVSIPERVIQFRVVWEDDSGA
NCU01195.1	MSNLPSEPEFEQAYKE1LAYTLENSSLFQKHPEYRTALTVASIPERVIQFRVVWEDDNGN
FG07174.1 	MSHLPHEPEFEQAYGE1LASALENSSLFNEHPEYRTALAVAAIPERVIQFRIVWEDDKGN
AN4376.1  	MSNLPVEPEFEQAYKE1LASTLENSTLFEQHPEYRRALQVVSVPERVIQFRVVWENDKGE
          	*: ** ****.*** * ** :**:*:**:::** * ** *.::********:***:*.* 

MG08074.1 	LRVNRGYRVQFSSALGPYKGGLRLHPS0----------------------~------GGA
NCU01195.1	VQVNRGYRVQFNSALGPYKGGLRLHPS~VNLSILKFLGFEQIFKNALTGL1SMGGGKGGA
FG07174.1 	LQVNRGYRVQFNSALGPYKGGLRFHPS~VNLSILKFLGFEQIFKNALTGL1SMGGGKGGA
AN4376.1  	VQINRGYRVQFNSALGPYKGGLRFHPS~VNLSILKFLGFEQIFKNALTGL1NMGGGKGGS
          	:::********.***********:***  . :  .  . ..  ..: :.  . ....**:

MG08074.1 	DFDPKGKSDAEIRRFCVAFMTELSKHIGADTDVPAGDIGVGGREIGYMFGAYRKLRNRFE
NCU01195.1	DFDPKGKSDAEIRRFCCAFMAELHKHIGADTDVPAGDIGVGGREIGYMFGAYRKAANRFE
FG07174.1 	DFDPKGKSDAEIRRFCQSFMTELSKHIGAETDVPAGDIGVGGREIGYLFGAYRKLRNRWE
AN4376.1  	DFDPKGKSDSEIRRFCTAFMTELCKHIGADTDVPAGDIGVTGREVGFLFGQYRRIRNQWE
          	*********:****** :**:** *****:********** ***:*::** **:  *::*

MG08074.1 	GVLTGKGLDWGGSLIRPE1ATGYGLVYYVGHMLEYAGVG--GWAGKRVAISGSGNVAQYA
NCU01195.1	GVLTGKGLSWGGSLIRPE~ATGYGLVYYVGHMLEYSGAG--SYAGKRVALSGSGNVAQYA
FG07174.1 	GVLTGKGLSWGGSLIRPE~ATGYGLVYYVDYMLKHANRG--SFEGKRVALSGSGNVAQYA
AN4376.1  	GVLTGKGGSWGGSLIRPE~ATGYGVVYYVEHMIKHVTGGKESFAGKRVAISGSGNVAQYA
          	******* .********* *****:**** :*:::   *...: *****:**********

MG08074.1 	ALKIMELGGTVVSLSDSRSALVAVEGGRTITEADVKRVMELKDRRRPLSDLLPESEEGGS
NCU01195.1	ALKLIELGATVVSLSDSKGALVATGESG-ITVEDINAVMAIKEARQSLTSFQH-------
FG07174.1 	ALKIIELGGSVVSLSDSKGALVAK-EGSSFTPEQIHNIAALKIKHQSLTAFEH-------
AN4376.1  	ALKVIELGGSVVSLSDSKGSLIVKDESASFTPEEIALIADLKVARKQLSELATSS-----
          	***::***.:*******:.:*:.   . ::*  ::  :  :*  :: *: :  .:     

MG08074.1 	TSPGGLKHLPGARPWTHV-GKVDVALPCATENEVSKEEAEALVAAGCRFVAEGSNMGCEQ
NCU01195.1	--AGHLKWIEGARPWLHV-GKVDIALPCATQNEVSKEEAEGLLAAGCKFVAEGSNMGCTL
FG07174.1 	--QDKFTWIEGARPWVHV-GKVDIALPSATQNEVSKEEAQALLEAGAFIVAEGSNMGCTA
AN4376.1  	AFAGKFTYIPDARPWTNIPGKFEVALPSATQNEVSGEEAEHLIKSGVRYIAEGSNMGCTQ
          	:  . :. : .**** ::.**.::***.**:**** ***: *: :*   :********  

MG08074.1 	GAIDVFEANRRSKGKGDAVWYAPGKAANCGGVAVSGLEMGQNSS0RLKWSREKVDKELAN
NCU01195.1	EAIEVFENNRKEKK-GEAVWYAPGKAANCGGVAVSGLEMAQNSQ~RLNWTQAEVDEKLKD
FG07174.1 	DAIDVFEAHRKENG-AQALWYAPGKASNCGGVAVSGLEMAQNSQ~RIQWSEKEVDDRLKA
AN4376.1  	AAIDIFEAHRNANP-GDAIWYAPGKAANAGGVAVSGLEMAQNSA~RLSWTSEEVDARLKG
          	 **::** :*. :  .:*:*******:*.**********.***  *:.*:  :** .*  

MG08074.1 	IMETAFRTGLDTAKEYVNRASDDELPSLVAGSNIAGFVKVARAMQAQGDWW---
NCU01195.1	IMKNAFFNGLNTAKTYVEAA-EGELPSLVAGSNIAGFVKVAQAMHDQGDWWSKN
FG07174.1 	IMKDAFVAGLETAQKYVEAK-EGELPSLVAGSNIAGFIKVAEAMHNQGDWY---
AN4376.1  	IMEDCFKNGLETAQKFATPA-KGVLPSLVTGSNIAGFTKVAEAMKDQGDWW---
          	**: .*  **:**: :.    .. *****:******* ***.**: ****:
```
