# Supplementary material for: Patterns of Intron Gain and Loss in Fungi
Source: PLoS Biol. 2004 Nov 30;2(12):e422. doi: 10.1371/journal.pbio.0020422 (PMC532390; doi:10.1371/journal.pbio.0020422)
Supplement: Table S1 — Also available at http://genes.mit.edu/NielsenEtAl/. (4.3 MB ZIP). [file pbio.0020422.st001.zip › NielsenEtAl/html/1089.html]

AN2858.1.NCU09533.1.MG07463.1.FG05130.1


```
 CLUSTAL W (1.82) Multiple Sequence Alignments - Introns Inserted


Sequence 1: NCU09533.1	417 aa
Sequence 2: MG07463.1	408 aa
Sequence 3: FG05130.1	409 aa
Sequence 4: AN2858.1	403 aa
Alignment Length: 417 aa
Number Identitical Residues: 259 aa
Alignment Score (without introns) 11884


MG07463.1 	--------M0VGTGEYTTGFVGGGASGSDKKVGVVGLSMFDLRRRGKVGKLGMVGVNGKK
NCU09533.1	MASPLNVLM0VGTGEYTTGFVGGGASGSDKKVGVVGLSMFDLRRRGKVGKLGMVGVNGTK
FG05130.1 	--------M0IGTGEYTTGFVGGGASGSDKKVGVVGLSLFDLRRREKVNKLGMVGVNGKK
AN2858.1  	-MAPPSVLM~VGTGEYTTGYVGGGASGSDKKVGVVGLTLFDLRRRGKVGDLSMVGVSGRK
          	  :. .  * :********:*****************::****** **..*.****.* *

MG07463.1 	FGAIR1EHLHKNIQQVYNNLDTSFDSYPANDAVDPDAYKAAIDALSPGDAITIFTPDTTH
NCU09533.1	FPAIR1EHLNKNITQVYNNLDTSFESFPADNAKDPESYKAAIDALQPGDAITIFTPDTTH
FG05130.1 	FPAIR1EHLNKNIQQVYNDLDTSFDSFPANDKVDPDAYKSAIDALSPGDAITIFTPDPTH
AN2858.1  	FPGIR1EHLHKNITQVYNNLDTSFTSYPADDATDPDAYKAAIDALPKGSAITIFTPDPTH
          	* .** ***:*** ****:***** *:**::  **::**:*****  *.********.**

MG07463.1 	YPIAMYAVERGIHVMLTKPAVKLLSHHQELLEAARKKGVYVYVEHHKRYDPAYADARFKS
NCU09533.1	YPIALYAIERKIHVMITKPAVKELAHHIALLEAAEKHGVYVYIEHHKRFDPAYADAKHRA
FG05130.1 	FPIAKYAIERGIHVMITKPAVKALEEHQQLVELAQKKGVYVYVEHHKRYDPAYADARAKA
AN2858.1  	FSIALYAIERGIHVLVTKPAVKTLPEHIALLEAAQKHNVFVFVEHHKRFDPAYSDARAKA
          	:.** **:** ***::****** * .*  *:* *.*:.*:*::*****:****:**: ::

MG07463.1 	QKLGAFNYFYSYMSQPKSQLETFKAWAGVDSDISYYLNSHHIDICDSMASPLGYVPVKVS
NCU09533.1	KKLGDFNYFYSYMSQPKSQLQTFAAWAGIDSDISYYLNSHHVDICDSMVSQLGYVPVKVS
FG05130.1 	QKLGDFNYFYSYMSQPKSQLETFKAWAGKESDISYYLNSHHIDINDSMVQERGYVPVSVN
AN2858.1  	KNLGDFNYFYSYMSQPKFQLETFKAWAGKESDISYYLNSHHVDICDSMVGN-DYTPVRVN
          	::** ************ **:** **** :***********:** ***.   .*.** *.

MG07463.1 	ASASKGVATSLGCNEATEDTITVLVHWENKE-TKGHATGVYTASWTAPQRAGVHSNQYFH
NCU09533.1	ASASTGVAVSLGCHEKTEDTISLLVHWQKEDDPTKHATGVYTASWTAPQKAGVHSNQYFH
FG05130.1 	ASSSKGVAVELGCDPCTEDTISLLVTWNKNGEPTKRAVGVYTASWTAPQKAGVHSNQYFH
AN2858.1  	ASASKGTAVELGCVPETEDTITLLVDWKHKTQPGKVATGVYTASWTAPQKAGVHSNQYFH
          	**:*.*.*..***   *****::** *::: ..   *.***********:**********

MG07463.1 	Y~LAKDGEIRIDQAKRGYDVADDNVGQLMWYNP~FYMKYAPDEDGNFAGQSGYGYISMEK
NCU09533.1	Y~LAQGGEIRIDQAKRGYDVAEDAAGQLMWYNP~FYMKYAPDEDGNFAGQSGYGYVSIEK
FG05130.1 	Y~LAANGEIRVDQAKRGYDVADDSVGQMMWYNP~FYMKYAPDEDGNFAGQTGYGYISIEK
AN2858.1  	Y1MGATGEIRVNQAKRGYDVTGDDSG-LAWINP2FYMRYAPDEEGNFGGQTGYGYISFEK
          	* :.  ****::********: *  * : * ** ***:*****:***.**:****:*:**

MG07463.1 	FVDGCRAINAGSLKPEDLDAKGLPTLKNTIATTAILEAGR~RAIDENREVMIEIKDGVWS
NCU09533.1	FVDGCRAVNAGTLTPRDLDAKGLPTLRNTIATTAILEAGR~RSIDENREVKIERKDGQWR
FG05130.1 	FVDGCRAVNSGKLKPEDLDAKPLPTLKNTIATTAILEAGR~RSIDENREVKIVIENGKWR
AN2858.1  	FIDAITALNEGRVTLKELDNRPLPTLKNTIATTAILHAGR0N--DPQRPLTVF-------
          	*:*.  *:* * :. .:** : ****:*********.*** .  * :* : :        

MG07463.1 	LV
NCU09533.1	LV
FG05130.1 	LE
AN2858.1  	--
          	
```
