# Supplementary material for: Patterns of Intron Gain and Loss in Fungi
Source: PLoS Biol. 2004 Nov 30;2(12):e422. doi: 10.1371/journal.pbio.0020422 (PMC532390; doi:10.1371/journal.pbio.0020422)
Supplement: Table S1 — Also available at http://genes.mit.edu/NielsenEtAl/. (4.3 MB ZIP). [file pbio.0020422.st001.zip › NielsenEtAl/html/109.html]

AN6546.1.NCU06713.1.MG04054.1.FG05194.1


```
 CLUSTAL W (1.82) Multiple Sequence Alignments - Introns Inserted


Sequence 1: MG04054.1	129 aa
Sequence 2: FG05194.1	128 aa
Sequence 3: NCU06713.1	128 aa
Sequence 4: AN6546.1	128 aa
Alignment Length: 129 aa
Number Identitical Residues: 85 aa
Alignment Score (without introns) 3757


MG04054.1 	MPNAGLKTIIALSF~VLAVGFLLVILSCALWKVYYPLLVVATYVLAPVPNWIARSCSNPD
NCU06713.1	MA-AGLKTIIALSF~VLAVGFLLVILSCALWKAYYPLLVVATYVLAPIPNWICSHCANPD
FG05194.1 	MS-AGLKTIIALSF~VLAVGFLLVILSCALWKVYYPLLVVATYVIAPIPNWICGHCANPD
AN6546.1  	MT-AGLKTIIALSF0VLAIGFLLVILSSALWHNFLPLTVVATYVIAPLPNWICSRCANPD
          	*. *********** ***:********.***: : ** ******:**:****.  *:***

MG04054.1 	DFVESSGGAALDLGRFCTGFLVVMGIA1LPIVLAHAHMINAGALVMSVAGGLLIYGTVIS
NCU06713.1	DFVESSGAAVLDLGRFCTGFLVMMGLA1LPVVLANSAIITVPAMIMSVIGGLLIYGTIIS
FG05194.1 	DFVESSGAAVLDLGRFFTGFFVVMGIA1LPVVLAHSGLIEVQAMVMSIIGGLLIYGTIVS
AN6546.1  	DFMDSSGNAVADFGRFLTGFLVLMGVA1LPAVLAHSGAIQVPAMIMSILGGLLIYGTIIS
          	**::*** *. *:*** ***:*:**:* ** ***::  * . *::**: ********::*

MG04054.1 	FGMFFTEEQEF
NCU06713.1	FAMFFQEEQDF
FG05194.1 	FGMFFHEEQDF
AN6546.1  	FSMFFREQEEF
          	*.*** *:::*
```
