# Supplementary material for: Patterns of Intron Gain and Loss in Fungi
Source: PLoS Biol. 2004 Nov 30;2(12):e422. doi: 10.1371/journal.pbio.0020422 (PMC532390; doi:10.1371/journal.pbio.0020422)
Supplement: Table S1 — Also available at http://genes.mit.edu/NielsenEtAl/. (4.3 MB ZIP). [file pbio.0020422.st001.zip › NielsenEtAl/html/1090.html]

AN5566.1.NCU02325.1.MG00919.1.FG10358.1


```
 CLUSTAL W (1.82) Multiple Sequence Alignments - Introns Inserted


Sequence 1: AN5566.1	533 aa
Sequence 2: NCU02325.1	529 aa
Sequence 3: MG00919.1	543 aa
Sequence 4: FG10358.1	522 aa
Alignment Length: 547 aa
Number Identitical Residues: 252 aa
Alignment Score (without introns) 13025


MG00919.1 	MSSTTEQAAAPHQTYD0TILTLDFG~SQY~THLITRRLRECNVYSEMLPCTTKLSDLTWK
NCU02325.1	-MAAATLGEVPTKAFD~TILTLDFG2---~---ITRRMRELNILSEMLPCTTKIADLDYK
FG10358.1 	MSDAFETAAPPHATYD0LIL-----~---~-----RRLRSLGVYSEMLPCTQKIKDLGWK
AN5566.1  	-MADT----IPHNTFD~TILVLDFG~SQY2THLITRRLREINVYSEMLPCTQKLADLGWK
          	          *  ::*  **  . . :.  :   :**:*. .: ******* *: ** :*

MG00919.1 	PKGIILSGGPYSVYDKDAPHADPAFFDLGVPVL~GICY~GLQELA~WRLGKDNVVAGTER
NCU02325.1	PKGVILSGGPYSVYEDGAPHVDPAVFEL-----~---D1GMQEIA~WRASPENVIAGVHR
FG10358.1 	PVGVILSGGPSSVYADDAPGVDPLVFELGVPVL2GVCY~GNQLIA1WRANPKSVARGVNR
AN5566.1  	PKGIILSGGPYSVYEEGAPHADPAFFELGVPIL~GICY~GLQELA~HRLHADNVVAGTAR
          	* *:****** *** ..** .** .*:*. .   . .  * * :*  *   ..*  *. *

MG00919.1 	EYGHADLTPQKNS--QAEKLFKGMGDDSLQV~WMSHGDKLSQLPTGFQTIATTANSPFAA
NCU02325.1	EYGHSNLKALKGDDAHVDRLFAGLED-SMRV~WMSHGDKLGALPEGFHTVAVSDNSEYAA
FG10358.1 	EYGETQMAIHKIG-THGDRLFEGLGD-SLIV1VMSDFDKVVQLPDGFQTIATTKNSEFAG
AN5566.1  	EYGHADLKATKFG-GHVDKLFENIEG-DMTV~WMSHGDKLRNLPEGFHTIGTTQNSEYAA
          	***.:::   * .  : ::** .: . .: *  **. **:  ** **:*:..: ** :*.

MG00919.1 	IAHESQNIFG~VQFHPEVTH~TRRGLDLLRNFAVGICGAQQNWNMHNFIEQEISRIRNLI
NCU02325.1	IAHKTKPIYG~LQFHPEVTH~SQNGTQLLKNFAVDICGCAQNWTMARFLDQEIARIRDLV
FG10358.1 	IAHETQPIFG1IQFHPEISH~TEKGTDIIANFATKICGARPDWKMDDFSAREIKRIRELV
AN5566.1  	IAHKSDPIYG~IQFHPEVTH1TPQGGQLLKNFAVGICGAEQKWTMAEFIGQEIQRIRSLV
          	***::. *:* :*****::* : .* ::: ***. ***.  .*.*  *  :** ***.*:

MG00919.1 	GDKAQV~IGAVSGGVDSTVAAKLM1KEAIGDRFHA~VLVDQGVMRHNECAEVKKALQD~H
NCU02325.1	GPEGQV~LGAVSGGVDSTVAAKLM~KEAIGDRFWA~VLVNNGVMRLDECEQVERDLKQ~H
FG10358.1 	GDKAQV0IGAVSGGVDSTVAAKLM~KEAIGDRFHA1ILVDQGLMRLN----------D2T
AN5566.1  	GPDGQV~LGAVSGGVDSTVAAKLM~TEAIGDRFHA~VLVDNGCMRLNECEKVQEVLQE~Q
          	* ..** :**************** .******* * :**::* ** :.. . .   .:  

MG00919.1 	LGINLTVADASERFLSGLKGVEDPEQKRKFIGNTF~IDIFEEEAIK~IEKAAENTPNAGK
NCU02325.1	LGINLTVIDASKDFLEGLKGLHDPEQKRKFIGGKF~IDVFEAEAQK~IEEAAAKSGKGTK
FG10358.1 	WALTSLVVDGSELFLGRLAGVTEPEAKRKIIGGTF1IDLFEIEALR2IEKEAENTDRAGK
AN5566.1  	LGINLTVVDAGEQFLAGLKGVHDPEQKRKFIGGKF~IDVFEDEARK~IEAKSNG-----K
          	 .:.  * *..: **  * *: :** ***:**..* **:** ** : **  :       *

MG00919.1 	VEWFLQGTLYP~D~VIESISFKGPSA-TIKT~HHNVGGLPKRMMEG~QG-LRLIEPLREL
NCU02325.1	IGFFLQGTLYP~D~VIESLSFKGPSA-TIKT~HHNVGGLPERMTNG~QG-LQLIEPLRSL
FG10358.1 	VEWFLQGTLYA1D~IVESLSFKGAASSTIKS1HHNAGGLPARMQNG0EAQLKLLEPLREL
AN5566.1  	VEWFLQGTLYP~D1VIESISFKGPSQ-TIKT~HHNVGGIAERLMRG~HG-LKLIEPLREL
          	: :*******. * ::**:****.:  ***: ***.**:. *: .* .. *:*:****.*

MG00919.1 	~FKDEVRAMGRELKINEELVMRHPFPG~PGIAIRILGEVTRERVEIARKADHVFISMIKE
NCU02325.1	~YKDEVRELGRTLGIHEELVMRHPFPG~PGIAVRILGEVTEEKVRIARQADHIFISEIRK
FG10358.1 	2FKDEVRAFGRQLGIHEELIGRHPFPG~PGLGIRIIGEVTPERVEIVRKADHIFISMIRE
AN5566.1  	~FKDEVRELGRQLGISPELVGRHPFPG1PGIAIRVLGEVTREKVEMARQADHIFISMIRE
          	 :***** :** * *  **: ****** **:.:*::**** *:*.:.*:***:*** *::

MG00919.1 	AGIYNEISQAYAALDTSRA~VGVM0GDKREYGYIVILRAVVTTDFMTATVYHMKPELLDR
NCU02325.1	AGLYDQISQAYAAVDPSRA~VGVM~GDKRVYGYIIILRAVTTTDFMTAEAFNFPWDFLQR
FG10358.1 	AGIYDEVTQAYAALDSSRA1VGVQ~GDARVYGYICILRAVTSLDMMSAEPYEFTWSLMKA
AN5566.1  	AGIYDEIGQAYAALDPSRA~VGVM~GDKRVYANIILLRAISTKDFMTATPYPFSYEFLSK
          	**:*::: *****:*.*** ***  ** * *. * :***: : *:*:*  : :  .::. 

MG00919.1 	ISTRIVNEVDGVTCVTYNITSKPPGTIEMQ
NCU02325.1	VMNRIVNEVNGVCRVTYDITSKPPGTIELE
FG10358.1 	ISRRIVNEVDGIARVVYDTTSKPPGTIELE
AN5566.1  	VSTRIVNEVAGVCRVCYDYTSKPPGTIEME
          	:  ****** *:  * *: *********::
```
