# Supplementary material for: Patterns of Intron Gain and Loss in Fungi
Source: PLoS Biol. 2004 Nov 30;2(12):e422. doi: 10.1371/journal.pbio.0020422 (PMC532390; doi:10.1371/journal.pbio.0020422)
Supplement: Table S1 — Also available at http://genes.mit.edu/NielsenEtAl/. (4.3 MB ZIP). [file pbio.0020422.st001.zip › NielsenEtAl/html/1091.html]

AN1037.1.NCU02209.1.MG01985.1.FG05784.1


```
 CLUSTAL W (1.82) Multiple Sequence Alignments - Introns Inserted


Sequence 1: MG01985.1	551 aa
Sequence 2: FG05784.1	475 aa
Sequence 3: AN1037.1	426 aa
Sequence 4: NCU02209.1	481 aa
Alignment Length: 552 aa
Number Identitical Residues: 234 aa
Alignment Score (without introns) 12526


MG01985.1 	MPSTRSTTSGIAQEKTPMRRTTTSATVESDVSAPGTAVQSPMDSP-RHSASSTSLSSLSS
NCU02209.1	--MASVSSALPEGNKPALRRTQTEATSDSYPGTAD---ASPFDSPLERSASNTSLSSQAS
FG05784.1 	----MASTSALPKQNPALRRTVTSTTATDTESAA----VSPSDSP-RHSASSTSLSSLSE
AN1037.1  	---------------------------------------------------------MAS
          	                                                          :.

MG01985.1 	VDAAAEKKSNESVGKLVDTYGNTFEIPDFTIKDIHDAIPKHCFERSAIRSLSYVARDMVL
NCU02209.1	DNVKTDK---AEFGKLLDTYGNEFEVPDFTIKDIRDAIPAHCFERSALHSLAHVVRDIIY
FG05784.1 	IDIAKPK---AEYGVMLDTYGNKFEVPDFTIKEIYNAIPKHCFQRSALKGYGYILRDIVL
AN1037.1  	D---AGK---GDLGKMLDTYGNEFKIPDYTIKDIRDAIPSHCYNRSAIRSLSYVFRDLAV
          	      *    . * ::***** *::**:***:* :*** **::***::. .:: **:  

MG01985.1 	LATTFYVFHNYVTPEYIPSKPARAGLWAIYTVLQGLFGTGIWVLAHECGHQAFSPSKTIN
NCU02209.1	LTVTFYVWNKYVTPEYIPMKAARVVLWGLYTFMQGLFGTGLWVLAHECGHQAFSPSRLIN
FG05784.1 	LATTFSIWYNYVTPEYIPSTPARAGLWAVYTVLQGLFGTGLWVIAHECGHGAFSDSRLIN
AN1037.1  	LASVFYVFHKYVTPETVPSYPARVALWTLYTVVQGLFGTGIWVLAHECGHQAFSTSKVLN
          	*: .* :: :***** :*  .**. ** :**.:*******:**:****** *** *: :*

MG01985.1 	NTVGWILHSSLLVPYFSWQMSHSKHHKATGHIERDMVFVPRTREEHASRIGRMVHELSEL
NCU02209.1	DTVGWVLHSALLVPYFSWKFSHSKHHKATGNIERDMVFVPRTREQFASRIGRFVHEISEL
FG05784.1 	DITGWVLHSSLLVPYFSWQISHRKHHKATGNMERDMVFVPRTREQQATRLGKMTHELAHL
AN1037.1  	DTVGWILHSALLVPYFSWKISHGKHHKATGNLARDMVFVPKTREVYASRIKKTIYDLNEV
          	: .**:***:********::** *******:: *******:***  *:*: :  ::: .:

MG01985.1 	TEETPIATLIHLVGQQLIGWPLYIITNKTGHNYHERQREGRGKGKKNGLFTGVNHFNPSS
NCU02209.1	TEETPIYTLIHLIGQQLIGWPNYLMTNVTGHNFHERQREGRGKGKKNGWFTGVNHFNPSS
FG05784.1 	TEETPVFTLIMLVLQQLVGWPNYLMTNVTGHNYHERQKEGRGKGKHNGLGGGVNHFDPRS
AN1037.1  	MEETPLATATHSILQQLFGWPLYLLTNVTGHDNHERQPEGRGKGKRNGYFTGVNHFNPNS
          	 ****: *    : ***.*** *::** ***: **** *******:**   *****:* *

MG01985.1 	PLYENKDAGKVLLSDLGVGLVIAGLVYLCQTFGTQNMLVWYFIPYLWVNHWLV1AITFLQ
NCU02209.1	PLYEEREAPWIIVSDIGIAIAATALIYLGNTFGWSNMFVWYFLPYLWVNHWLV~AITYLQ
FG05784.1 	PLYEHSDAKLIVLSDIGIGLMGTALYFLVQKFGFYNMAIWYFVPYLWVNHWLV1AITFLQ
AN1037.1  	PLFEAKDAKLIILSDIGLAITASILYLIGSKFGWMNLLVWYGIPYLWVNHWLV~AITYLQ
          	**:*  :*  :::**:*:.:  : *  : ..**  *: :** :********** ***:**

MG01985.1 	HTDPSLPHYTAEEWNFVRGAAATIDREFGFVGRHLLHGIIETHVLHHYVSTIPFYNADEA
NCU02209.1	HTDPSLPHYTPDQWNFVRGAAATIDREFGFIGRHLLHGIIETHVLHHYVSTIPFYHADEA
FG05784.1 	HTDPTLPHYTNDEWNFVRGAAATIDREMGFIGRHLLHGIIETHVLHHYVSSIPFYNADEA
AN1037.1  	HTDPTLPHYQPESWTFARGAAATIDREFGFIGRHILHGIIETHVLHHYVSTIPFYHADEA
          	****:****  :.*.*.**********:**:***:***************:****:****

MG01985.1 	TDAIKKVMGKHYRSDTAGGPAGFLKSLWTSSRMCQWVEPSAEAEGSGKGVLFFRNHNKIG
NCU02209.1	SEAIKKVMGRHYRADVQDGPIGFIKAMWKAARWCQWVEPTEGAEGKGKGVLFYRNQNGLG
FG05784.1 	TEAIKPVMGKHYRADVQDGPRGFIRAMYRSARMCQWVEPSAEAEGAGKGVLFFRNRNKVG
AN1037.1  	SEAIKKVMGSHYRSEAHTGPLGFLKALWTSARVCHWVEPTEGTKGENAGVLFFRNTNGIG
          	::*** *** ***::.  ** **::::: ::* *:****:  ::* . ****:** * :*

MG01985.1 	TPPIKMSAQ2KIRLCNDLLGMHK0GKNQMNGSRERRGGQSSLKRVRNQRSTNMNESHMTV
NCU02209.1	VKPAKLPKT~N------------~------------------------------------
FG05784.1 	TAPAVLKA-~-------------~------------------------------------
AN1037.1  	VPPIKLTKP~N------------~------------------------------------
          	. *  :    .                                                 

MG01985.1 	FRAFRTWSSCTRAST
NCU02209.1	---------------
FG05784.1 	---------------
AN1037.1  	---------------
          	
```
