# Supplementary material for: Patterns of Intron Gain and Loss in Fungi
Source: PLoS Biol. 2004 Nov 30;2(12):e422. doi: 10.1371/journal.pbio.0020422 (PMC532390; doi:10.1371/journal.pbio.0020422)
Supplement: Table S1 — Also available at http://genes.mit.edu/NielsenEtAl/. (4.3 MB ZIP). [file pbio.0020422.st001.zip › NielsenEtAl/html/1092.html]

AN7658.1.NCU01161.1.MG07332.1.FG10361.1


```
 CLUSTAL W (1.82) Multiple Sequence Alignments - Introns Inserted


Sequence 1: NCU01161.1	724 aa
Sequence 2: MG07332.1	759 aa
Sequence 3: FG10361.1	736 aa
Sequence 4: AN7658.1	659 aa
Alignment Length: 774 aa
Number Identitical Residues: 291 aa
Alignment Score (without introns) 16029


MG07332.1 	MADTEVAPTFGAELK0DGFKPANAWVGHGIAWIDDLQQFYRERAAIEKDYSAKLAALAKK
NCU01161.1	-------------MQ~DGFKPANVYVANNIAFFDDVQAFFRERSAAEKRYSAELSAIAKK
FG10361.1 	MAEVDVMPTFGSELK0DGFKPANVWLGHGIAWLDEIQQFYRERAAIEKEYSAKLMALSKK
AN7658.1  	MAAAEAAPHFGAELK0---------VSNGIAWMEEVQQFYRERSAIEKEYAAKLTALCKK
          	 :  .  .  .:.::          :.:.**:::::* *:***:* ** *:*:* *:.**

MG07332.1 	YYEKKSKKSAVLSVGDTPAMTPGSLES2ASITTWTTQLNTVERRAEEHSRYANELINRVA
NCU01161.1	YYDKKAKKSTSLSVGDTPTMTPGSLES2ASMTTWSTILTKLELVASEHDRLANEFVTKIA
FG10361.1 	YFEKKNKRTAQLSVGETPAMTPGSLES2ASLTTWATQLTTLESRAGEHDKYANNLVSQVA
AN7658.1  	YYDRKAKKISPLSVGDNPTMTPGSLES~ASLTTWSTQLAAVEAHAAERDKFATDLVAQVA
          	*:::* *: : ****:.*:******** **:***:* *  :*  * *:.: *.::: ::*

MG07332.1 	TPMQAFQTKFDELRKRHAEFADKLEKERDSSYSELRKVKGKYDNVCQEVESKRKKTESAF
NCU01161.1	EPIKEVAIKLDAQRKRQVEYAEKLEKEKEAQYAALQKVKTKYDSVCQDVESKRKKSESAF
FG10361.1 	EPLKFFGGRFEELRKRHSDYATKLEQERDSQYAALAKTKGKYDNVCQEVEAKRKKTESHF
AN7658.1  	EPLKQSAVQYEELRKCHVDFHGKLEKERESSFSDLKKAKGKYDGACQEVESRRKKMESAF
          	 *::    : :  ** : ::  ***:*:::.:: * *.* ***..**:**::*** ** *

MG07332.1 	D--KAKAQSNYQQQIHEMNNAK~NTYIIAINVTNKQKEKYYHEYVPEVMD~GLQDLSEFK
NCU01161.1	D--KAKAQSSFQHQLQEMNNMK~NTYLIAINVTNKQKEKHYHEFLPEVMD~NLQDLAEFR
FG10361.1 	D--KAKTQNAYQQQILEMNNAK~NTYIIAINVTNKQKEKYYHEYVPEVMD~SLQDLSEFK
AN7658.1  	DHGKTKAQTVYQQQIMEMNNVK0------------LKEKFFHEYVPELLD0---------
          	* .*:*:*. :*:*: **** *              ***.:**::**::*          

MG07332.1 	TIKINELWTLASTLESGLLKSSGEMIDHQTEEIARNLPHLDSMMYMRHNIGAWQEPSDKM
NCU01161.1	TMKLNGFWSEATQLEFSMLQNCSGEVDHMAKEIVRNEPQLDSMMYMQHNTGAFQEPPDKV
FG10361.1 	TTKLNSFWTIATNIESEMLQQSNGMVQHQGNEILRNLPHLDSMMYIQHNMGAFNEPADKH
AN7658.1  	------------------------------------------------------EPANVG
          	                                                      **.:  

MG07332.1 	FEPSPVWHDDDSMVVDEPAKVYLRNVLNKSKSQLGDLRREVDKKRREVESMKRVKQNVRN
NCU01161.1	FEPSPVWHDDDSMVTDEPAKIYLRNVLNKSKGQLGELRREVDKKRREVETTKRAKQRVLE
FG10361.1 	FEASPVWHDDGTMVVDETAKVFLRNVLGKSKSQLGELRREVDKKRREVEGVKQLKQRVRE
AN7658.1  	FEPSPVWHDDEALITDETAKVFLRNLLSKSKTQVRELRVESDQKRREVENAKRVRQSIQE
          	**.******* :::.**.**::***:*.*** *: :** * *:******  *: :* : :

MG07332.1 	GTDK-KDEVAVVNSIFAMQEDLHQVERKRITAEVETSTITSAVGDVTLGAKNHNFKSQTF
NCU01161.1	GTEQTQDEFLVLTQLFLQQEDLHQVDRKRLTAEVETSTITSAVGDVTLGAKSHNFKSQTF
FG10361.1 	GKEK-KDEVEVVRALFQMQENLHAIDRQRLTAEVETSTITSVVGDVTLGAKNHNFKGQTF
AN7658.1  	GRDN-RNEVDVVRSIFFMQESLHEIERKRLTAEVETSTIISVVGDLSLGAKNHNFKSQTF
          	* :: ::*. *:  :*  **.** ::*:*:********* *.***::****.****.***

MG07332.1 	KIPTNCDLCGERIWGLSAKGFDCRDCGYTCHSKCEMKVPAECPGEQSKEDRKKLKAERQE
NCU01161.1	KIPTNCDLCGERIWGLSAKGFDCRDCGYTCHSKCEMKVPAECPGELNKEERKKYKQERQE
FG10361.1 	KIPTNCDLCGERIWGLSAKGFDCRDCGYTCHSKCEMKVPPDCPGEQTKDERKKLKAERQD
AN7658.1  	KIPTNCDLCGERIWGLSAKGYDCRDCGYTCHSKCEMKVPAECPGEQTKEEKKRLKAERQE
          	********************:******************.:**** .*:::*: * ***:

MG07332.1 	AAN-TLLKPSASTTHKKTPSSTEGMAELPALSRSDTMNSLSSGYAASAHR-SISGARSPA
NCU01161.1	LAN-TLLKPSTSSSHVN---------EMPDLTRSNTVSSVRSGYAASAQR-SISGPISPG
FG10361.1 	AANNKLLKPSATMTSVHSNNS-----DAPELTRSNTMTSLSSHSARPSISGSISAQLTPT
AN7658.1  	QASAAPAVDLAPTSASS---------TAPSLSRRDTMNSLSSGYAVSANR-SVSNVGTHE
          	 *.       :. :              * *:* :*:.*: *  * .:   *:*   :  

MG07332.1 	--EEMPPGE-----APAAPKPANTLRKNRVIAPPPAAYISELPGSSPANGSSGIGAGKTP
NCU01161.1	--DDAPPDVPNTRPPAASPEPSSVPRKNRILAPPPPAFMSEVPG-SASNG------GGHQ
FG10361.1 	--EETPPEVAR--PSVSSTATSGTAPKRRIMAPPPTAYIKDSGA-NETNG------GAKE
AN7658.1  	SVAEAPESIPTSTAPAAPTATKPAVKRNRILAPPPAQYISSPPSAEAPTSN-----SSQK
          	:  : *   . : .. :.. .  .  :.*::****. ::..  .:. ....     .   

MG07332.1 	EQKGKMLYGFDANGEGELTVPAGRDLVILEPDT1GSGWIKVRAG-YKEGLVPATYVELAP
NCU01161.1	EQKAKMLYTFEAGGEGELSVLEGRELVVLEPDT1GSGWTRVRAG-YKEGNVPTSYVEILP
FG10361.1 	EKRGKMIYPFEATGEGELTVQDGRDVVLLEPDD1GSGWVKVRAG-YKEGLVPTSYVEFTT
AN7658.1  	EPRGKMLYPYQATGADEVTVQEGEEIFVLEPDD1GSGWMRVRSESSAEGLVPASYVEVLA
          	* :.**:* ::* * .*::*  *.::.:****  **** :**: :  ** **::***. .

MG07332.1 	AGAPSATVIAPPPVMAPQHTGASGRPGSIYSNSGSSIG--GTAPAVKKKGPAVAPRRGAK
NCU01161.1	P--------TTPTALVPQHTGQSGRPPSTYSNSGSSI-----AASTKKKGPAVAPRRGAK
FG10361.1 	V-------------TIPSAPAPSARPSSTYSTSTTSS----LTQSNKKKGPAVAPKRGAK
AN7658.1  	AP--------------ASSSSPAGRPGSTYSSSSASLAGSTAAAAGKKVGPAVAPRRGAK
          	                .. .. :.** * **.* :* ..:  : : ** ******:****

MG07332.1 	KAVRHVDVMYDYTAQSDAEHSIVEGERLVLIKEDPGDGWAEVEKGGLTKSVPASYLQLV
NCU01161.1	K-LQYVEALYDYQAGSDTEHSMTVGERFVLIKEDQGDGWAEVEKGGVTKSVPANYVQTV
FG10361.1 	K-LRYVEALYEYAAQAETEHSMAEGERFVLVQEDPGDGWVEVEKAGVTGSVPASYVQAV
AN7658.1  	K-LQYVEALYDYEARSDMEWSMVEGDRFVLVNRDSGDGWADVERGGVTKSVPANYIQEV
          	* :::*:.:*:* * :: * *:. *:*:**::.* ****.:**:.*:* ****.*:* *
```
