# Supplementary material for: Patterns of Intron Gain and Loss in Fungi
Source: PLoS Biol. 2004 Nov 30;2(12):e422. doi: 10.1371/journal.pbio.0020422 (PMC532390; doi:10.1371/journal.pbio.0020422)
Supplement: Table S1 — Also available at http://genes.mit.edu/NielsenEtAl/. (4.3 MB ZIP). [file pbio.0020422.st001.zip › NielsenEtAl/html/1093.html]

AN5601.1.NCU03748.1.MG08564.1.FG00346.1


```
 CLUSTAL W (1.82) Multiple Sequence Alignments - Introns Inserted


Sequence 1: NCU03748.1	524 aa
Sequence 2: MG08564.1	450 aa
Sequence 3: AN5601.1	450 aa
Sequence 4: FG00346.1	450 aa
Alignment Length: 528 aa
Number Identitical Residues: 224 aa
Alignment Score (without introns) 11270


MG08564.1 	-----~---------------------------~--------------------------
NCU03748.1	MVPIT1AKSDWRPARLYLYCKVRQASWCCYPAT1CRYHKSQRWLPNSNAIPLTPPTYISP
FG00346.1 	-----~---------------------------~--------------------------
AN5601.1  	-----~---------------------------~--------------------------
          	                                                            

MG08564.1 	-------------------MATKSVLML2GSGFV~TRPTLDVLTDSGIKVTVA1CRTLES
NCU03748.1	AILPSPRATARAGDADLKFHVFSKVLML~GAGFV~TRPTLDVLSEAGIPVTVA1CRTLAS
FG00346.1 	--------------------MPQSALLL~GSGFV~ATPAVEVLSKAGVHVTVA~CRTLAS
AN5601.1  	---------------MAKQIAGSKVLLL~GSGFV1TKPTVEVLSKADVEVTVA1CRTLES
          	                 .    ...*:* *:*** : *:::**:.:.: **** **** *

MG08564.1 	AKKLSAGVQHSTPISLDVNDDAALDAEVAKHDLVISLIPYTFHATVIKSAIRQKKHVVTT
NCU03748.1	AQKLSEGVKNATPISLDVTNDEALDAEVAKHDLVISLIPYTFHATVIKSAIRQKKHVVTT
FG00346.1 	AKNLAGTFDNTKAVSLDVNDSAALEQAVSEHDITISLIPYTFHAAVIKAAIKAKKNVVTT
AN5601.1  	AKKLCEGFKNTKAISLDVNDDKALDEAMSKVDLAISLIPYTFHAQVIKSAIRTKKHVVTT
          	*::*.  ..::..:****.:. **:  ::: *:.********** ***:**: **:****

MG08564.1 	SYVSPAMMELDQAAKDAGITVMNEIG~LDP~G~IDHLYAIKTIEE~VHAAGGKIKTFLSY
NCU03748.1	SYVSPAMMELDAEAKAAGITVMNEIG0LDP~G~IDHLYAIKTIDE~VHQAGGKILSFLSY
FG00346.1 	SYVSPAMEELHEEAKAAGITVLNEIG0VDP~G2VDHLYAVDFIDR~IQQEGGKIKSFKSY
AN5601.1  	SYVSPAMMELDQQCKDAGITVMNEIG~LDP0G~IDHLYAVKTISE0VHAEGGKITSFLSY
          	******* **.  .* *****:**** :** * :*****:. *.. ::  **** :* **

MG08564.1 	CGGLPAPES~SDNPLG~YKFSWSSRGVLLALRNAASFYKDGKVTNVAGPELMATAKPYFI
NCU03748.1	CGGLPAPED~SDNPLG~YKFSWSSRGVLLALRNAGKWWQDGKIVEVEGKDLMKMAKPYFI
FG00346.1 	CGGLPAPEN1SNNPLG1YKFSWSSRGVLLALKNNAKYYEDNKLVDISGVDLMSTAQPYHS
AN5601.1  	CGGLPAPEC~SNNPLG~YKFSWSSRGVLLALRNAAKFYQDGQEKSIAGPELMAAAKPYYI
          	********  *:**** **************:* ..:::*.:  .: * :**  *:**. 

MG08564.1 	-YPGFAFVAYPNRDSTPYKER~YQIPEADNIVRG0TLRYQGFPQFIKVLVDIGFLSDEEQ
NCU03748.1	-YPGYAFVAYPNRDSTIYKER~YNIPEAQTVIRG~TLRYQGFPQFIKTLVDIGFLDDTAR
FG00346.1 	GYLGFNFVAYGNRDSTGYRER2YRIPDAETVVRG~TMRYNGFPQFVKALVDIGFLSTDEQ
AN5601.1  	-YPGFAFVAYPNRDSTPFRER~YNIPEAQTLVRG~TLRYQGFPEFIKVLVDMGFLSDEPQ
          	 * *: **** ***** ::** *.**:*:.::** *:**:***:*:*.***:***.   :

MG08564.1 	PFLKEAIPWKEATQKIVKASSASEQDIVSTIVSNATFESTEEQKRIVAGLKWL1GIFSDK
NCU03748.1	ESLSKQTPWKEATKEIVGAASSSQADLEAAILSKATFESAEDQKRILSGLRWI~GLFSDE
FG00346.1 	DFFKQSIPWKDALQKFIGANSSSEEDLTKAILSKTSFKDESVKNQVLAGLKWI1GVFSDV
AN5601.1  	TYLSTPIAWKEATQKILGATSSDEKDLEWAISSKTTFPNNDERDRIISGLRWI~GIFSDE
          	  :.   .**:* :::: * *:.: *:  :* *:::* . . :.::::**:*: *:*** 

MG08564.1 	KITPRGNALDTLCATLEEKMQFEEGERDLV0MLQHKFEIENKDGSRETRTSSLCEYGAPI
NCU03748.1	TITPRGNPLDTLCATLEQKMQFEEGERDLV~MLQHKFEIEHADGSRETRTSTLVEYGDPK
FG00346.1 	KTTPRGTALDTLCASLEQKMAYEKGERDIV~FLQHTFEVINKDGSQNTWTSTLVEYGAPE
AN5601.1  	KITPRGNPLDTLCATLEQKMQYGPEERDLV~MLQHKFGIEHKDGSKEVRTSTLCEYGVPG
          	. ****..******:**:** :   ***:* :***.* : : ***::. **:* *** * 

MG08564.1 	GSGGYSAMAKLVGVPCAVA~VKFVLDGTISDRGVLAPMNSKINDPLMKELKEKYG2IECK
NCU03748.1	G---YSAMAKTVGVPCAVA~VKQVLSGQISGKGVLAPMSTDITEPLMKELHEKYG~ITMI
FG00346.1 	GSGGFSAMSRLVGVPCGVA1TKMVLDGTITDKGVVAPVYPSLARTLMNELKNNYG2IECK
AN5601.1  	G---YSAMAKLVGIPCGVA~VKLVLDGTINQTGVLAPMTWDICEPIQKTLKEEYG~IEMI
          	*   :***:: **:**.** .* **.* *.  **:**:  .:  .: : *:::** *   

MG08564.1 	EKVVA
NCU03748.1	EKTIS
FG00346.1 	EKIIA
AN5601.1  	EKTL-
          	** :
```
