# Supplementary material for: Patterns of Intron Gain and Loss in Fungi
Source: PLoS Biol. 2004 Nov 30;2(12):e422. doi: 10.1371/journal.pbio.0020422 (PMC532390; doi:10.1371/journal.pbio.0020422)
Supplement: Table S1 — Also available at http://genes.mit.edu/NielsenEtAl/. (4.3 MB ZIP). [file pbio.0020422.st001.zip › NielsenEtAl/html/1094.html]

AN1013.1.NCU04331.1.MG07048.1.FG10010.1


```
 CLUSTAL W (1.82) Multiple Sequence Alignments - Introns Inserted


Sequence 1: NCU04331.1	301 aa
Sequence 2: MG07048.1	302 aa
Sequence 3: FG10010.1	301 aa
Sequence 4: AN1013.1	301 aa
Alignment Length: 303 aa
Number Identitical Residues: 205 aa
Alignment Score (without introns) 8947


MG07048.1 	-~M0PFHKLVKNSAYYS2RFQTKYKRRQQGKTDYYARKRLITQAKNKYNAPKYRLVVRFT
NCU04331.1	-~M0AFHKLVKNSAYYS2RFQTKYKRRREGKTDYYARKRLITQAKNKYNAPKYRLVVRFT
FG10010.1 	-~M0PF-KLVKNSAYYS2RYQTKYKRRQQGKTDYYARKRLITQAKNKYNAPKYRLVVRFT
AN1013.1  	M0A~PYSKTVKSSAYYS2RYQTKYRRRREGKTDYYARKRLITQAKNKYNAPKYRLVVRFT
          	    .: * **.***** *:****:**::*******************************

MG07048.1 	NRDIIMQIVTSEISGDKVFASAYSHELKAYGITHGLTNWAAAYATGLLIARRVLKKLGLD
NCU04331.1	NRDIILQIVSSEITGDKVFASAYSHELKAYGIEHGLTNWAAAYATGLLLARRVLKKLGLD
FG10010.1 	NKDIICQIVTSEISGDKVFISAYAHELKAYGIEHGLTNWAAAYATGLLVARRALKKLGLD
AN1013.1  	NRDIVTQIVYSEITGDKVFASAYSHELKRYGITNGLTNWAAAYATGLLLARRTLKKLGLD
          	*:**: *** ***:***** ***:**** *** :**************:***.*******

MG07048.1 	EDFTGVEEPEGEFTLTEAAETDEGTRRPFKAYLDVGLARTSTGARVFGAMKGASDGGILI
NCU04331.1	ETFKGVEEADGEYKLTEAAETDDGERRPFKAFLDVGLARTSTGARVFGAMKGASDGGIFI
FG10010.1 	EDFAGVEEADGEYKLTEAAETDDGERRPFKVFLDVGLKRTSTGARVFGAMKGASDGGILV
AN1013.1  	EDFTGVEEPDGEFSLTEAAETEEGTRRPFKAFLDVGLARTSTGARVFGAMKGASDGGIFI
          	* * ****.:**:.*******::* *****.:***** ********************::

MG07048.1 	PHSENRFPGFDIESKELDSETLKKYIFGGHVAEYMETLADDDEERYKSQFQQYIDEDIDA
NCU04331.1	PHSENRFPGYDMESEELDAETLKKYIFGGHVAEYMETLADDDEERYKSQFNRYIEDDLEA
FG10010.1 	PHSEKRFPGYDMETKELDADTLRNYIFGGHVAEYMETLADDDEERFRSQFQKYVDDDVEA
AN1013.1  	PHSESRFPGYDIEAEELDAETLRSYIFGGHVAEYMEGLADDDEERFRGQFHKYTENEIDA
          	****.****:*:*::***::**:.************ ********::.**::* :::::*

MG07048.1 	DGLEDIYTEAHAAIREDPFKKPEGAEAKKSKEEYKKESLKFKAKKLTLAERKARVQERIA
NCU04331.1	DGLEDLYAEAHAAIREDPFKKAESEAPKKTKEEWKAESLKYKKSKLTREQRAAGVQERIA
FG10010.1 	EGLEDLYTEAHAAIREDPFKKVESDAPKKTKEEWKEISQKYKSKKLTKEEKEKRVQERIQ
AN1013.1  	GDIEELYAEAHKAIRADPFKKDESEGPKKTKEEWKAESKKYRKTKLSHEEKKARVEAKIR
          	 .:*::*:*** *** ***** *.  .**:***:*  * *:: .**:  ::   *: :* 

MG07048.1 	ELRDAE
NCU04331.1	ALRSE-
FG10010.1 	EIMQRD
AN1013.1  	ELAA--
          	 :
```
