# Supplementary material for: Patterns of Intron Gain and Loss in Fungi
Source: PLoS Biol. 2004 Nov 30;2(12):e422. doi: 10.1371/journal.pbio.0020422 (PMC532390; doi:10.1371/journal.pbio.0020422)
Supplement: Table S1 — Also available at http://genes.mit.edu/NielsenEtAl/. (4.3 MB ZIP). [file pbio.0020422.st001.zip › NielsenEtAl/html/1095.html]

AN2208.1.NCU08882.1.MG05346.1.FG02075.1


```
 CLUSTAL W (1.82) Multiple Sequence Alignments - Introns Inserted


Sequence 1: NCU08882.1	370 aa
Sequence 2: MG05346.1	370 aa
Sequence 3: FG02075.1	362 aa
Sequence 4: AN2208.1	360 aa
Alignment Length: 374 aa
Number Identitical Residues: 118 aa
Alignment Score (without introns) 6914


MG05346.1 	MSDQKNFNVGVVGYG2MSAKVFHIPFIKLTEGLTLHSIVQRSPKPNDSAPHDYPDIHHHT
NCU08882.1	MAPSNKFNVGIIGYG2LSAKVFQIPFVALTESLVLHSIVQRSPSPGNSAPEDHPSAKHFT
FG02075.1 	MSG-KTFNVGIVGYG2MSAKIFHIPFLTQTPQLKLHAIVQRSPKEGNSAPADYPDIKHYT
AN2208.1  	MAS-KTWNVGIVGYG~FSAKIFHIPFVQENPQFKLYAVVQRTPKPDDDAEKDHPGIKSYR
          	*:  :.:***::*** :***:*:***:  .  : *:::***:*. .:.*  *:*. : . 

MG05346.1 	SVDTLVADPALDLVVISTTPDTHYAFVKQALEAGKHV~LVEKPFVPTSAQAVELADLART
NCU08882.1	SIEPMLADPDVHVVIISTPPNTHFETARDALRNGKHV~LVEKPFVPTSAQAEELAALAAE
FG02075.1 	DYKQLFADSDVDLIIISTPPNNHFELTKAALKAGKHV~LTEKPFVPTSAEADKLIEIAKQ
AN2208.1  	SAEDLVQDAGVDVVVITTAPDSHHYLAKLALENGKHV1ICEKPFTPTYKEAAELVDISKK
          	. . :. *. :.:::*:*.*:.*.  .: **. **** : ****.**  :* :*  ::  

MG05346.1 	KGKLLCVYQN~RRWDGDFVTLRRLLEVGELGRVWEFETHFDRHRAAH--PTNWKASMTMS
NCU08882.1	KKRVLCVYQN~RRWDSDFLTVQKLIREGTLGRIVEFETHFDRFRLEK--PTTWKGQLSMD
FG02075.1 	NGKLLIVYQN~RRWDSDFVTLKKLISEGTLGRIFQFDNHFDRYRMVP--SNNWKLDLPLS
AN2208.1  	QNKFLAVYQN1RRWDADFVTLSKLVKTGQLGRVVEFETHFDRHRPEEPAPTVSKWKNKVV
          	: :.* **** ****.**:*: :*:  * ***: :*:.****.*   .:..  * .  : 

MG05346.1 	QGGGVVYDLGTHLVDQVYVLFGKPSTVSAKFVRQREGRLVSGVNGIEDEPDSVNAVLSYA
NCU08882.1	QAGGVLYDLGTHLLDQVFVLFGMPTSVSAKFLDQREGRIVTGGSDESQQPDSIAAVLTYA
FG02075.1 	QGGSALFDLGTHLIDQAYVLFGKPQSVHGRLLSQRSGKFDFE------NPDGVSAELTYP
AN2208.1  	PGGSAIHDLGSHLLDQALYLLGKPERVTGFVGSQREVNTSG-------FQDSFTVLLHYK
          	 .*..:.***:**:**.  *:* *  * . .  **. .            *.. . * * 

MG05346.1 	DSGLLVHVRIGVLSVEAEQPRFWVRGSEGTYRK~TGLDTQEDQLKAGMTGTEKEFGRDEK
NCU08882.1	DTGLLVHVRISVVSAETKQPRFRIRGTKGSYQK~AFLDPQEDQLRGGMAATDARFGKEDE
FG02075.1 	D-GLIVNIRISVLSAELEQPRFWVRGTKGSFRK~LGLDTQEDALKAGTKATDEGFGKEDP
AN2208.1  	S-GTLVTAKAGVVSPEEEQLRFWVRGDKGSFKK0FHLDCQEDQLKAGMRPGDSGYGREPS
          	. * :*  : .*:* * :* ** :** :*:::*   ** *** *:.*    :  :*::  

MG05346.1 	P--G~RLAKLSAEGKAQEVAYPNIDPPATYLQLYRGLAAALRSGKEEDCPVPAAQAAEVL
NCU08882.1	SRYG~RLCYVTEDGKIEEKVYPTTEP-ETYIKIFEGFAKALETGNEDDIPVPASQAAKVL
FG02075.1 	AR-Y~KLIVVDENEKAKEQSLSSIEV-PTYKAFYAQLAKAVETGKEEDVPVKASEARDVL
AN2208.1  	ERYG1TLTTIK-DGKPVREVTPTVEP-PTYSEYYRKIARALAG--EGELPASGEEAAEVI
          	      *  :  : *  .   .. :   **   :  :* *:    * : *. . :* .*:

MG05346.1 	KIIEAMRESAKTGKDVVPA
NCU08882.1	RIIEALRESAKTGRDVAP-
FG02075.1 	QIIEGVFESAKTGKDVTFA
AN2208.1  	RLIELAQESSKQGKTLDF-
          	::**   **:* *: :
```
