# Supplementary material for: Patterns of Intron Gain and Loss in Fungi
Source: PLoS Biol. 2004 Nov 30;2(12):e422. doi: 10.1371/journal.pbio.0020422 (PMC532390; doi:10.1371/journal.pbio.0020422)
Supplement: Table S1 — Also available at http://genes.mit.edu/NielsenEtAl/. (4.3 MB ZIP). [file pbio.0020422.st001.zip › NielsenEtAl/html/1096.html]

AN2904.1.NCU02260.1.MG00908.1.FG10769.1


```
 CLUSTAL W (1.82) Multiple Sequence Alignments - Introns Inserted


Sequence 1: NCU02260.1	421 aa
Sequence 2: MG00908.1	421 aa
Sequence 3: FG10769.1	421 aa
Sequence 4: AN2904.1	430 aa
Alignment Length: 430 aa
Number Identitical Residues: 363 aa
Alignment Score (without introns) 13833


MG00908.1 	MGDVLVENSSNYTPPHKKAAPS-TIPSIENFEGLPTEGGDDYATLKKLQRQLE~YIQLQE
NCU02260.1	MGDVLVETPASVTQPQKKSAPS-AIPNIETFEGVSTEGGDDYITLKKLQRQLE~YIKLQE
FG10769.1 	MGDVAVENPANQVAPHKRAAHS-AIPTIDNFEGVSTDGGDDYANLKKLQRQLE2YIQLQE
AN2904.1  	MGDIAVENPASVLSSYTKAAPLDTIPNIDSLEGTGADDGDEYATLKKLQRHLE2YIKLQE
          	***: **..:.   . .::*  .:**.*:.:**  ::.**:* .******:** **:***

MG00908.1 	EYIKDEQR2SLKRELVRAQEEIKRIQSVPLVIGQFMEAIDQN2TGIVQSSTGSNYVVRIL
NCU02260.1	EYIKDEQR2SLKRELVRAQEEIKRIQSVPLVIGQFMEAIDQN2TGIVQSSTGSNYVVRIL
FG10769.1 	EYIKDEQR2SLKRELVRAQEEIKRIQSVPLVIGQFMEAIDQN2TGIVQSSTGSNYVVRIL
AN2904.1  	EYIKDEQR2SLKRELVRAQEEIKRIQSVPLVIGQFMEAIDQN2TGIVQSSTGSNYVVRIL
          	******** ********************************* *****************

MG00908.1 	STLDRELLKPSSSVALHRHSNALVDILPPEADSSIAMLGADEKPDVTYADVGGLDMQKQE
NCU02260.1	STLDRELLKPSSSVALHRHSNAVVDILPPEADSSIAMLGADEKPDVTYADVGGLDMQKQE
FG10769.1 	STLDREKLKASSSVALHRHSNALVDILPPEADSSIAMLGTDEKPDVTYADVGGLDMQKQE
AN2904.1  	STLDREKLKPSSSVALHRHSNALVDILPPEADSSIAMLGENEKPDVTYADVGGLDMQKQE
          	****** **.************:**************** :*******************

MG00908.1 	IREAVELPLTHFDLYKQI-~-------GIDPPRGVLLYGPPGTGKTMLVKAVANSTTANF
NCU02260.1	IREAVELPLTHFDLYKQI-~-------GIDPPRGVLLYGPPGTGKTMLVKAVANSTTANF
FG10769.1 	IREAVELPLTHFDLYKQI-~-------GIDPPRGVLLYGPPGTGKTMLVKAVANSTTANF
AN2904.1  	IREAVELPLTHFDLYKQIV1LIVFLRLGIDPPRGVLLYGPPGTGKTMLVKAVANSTTASF
          	******************         *******************************.*

MG00908.1 	IRVVGSEFVQKYLGEGPRMVRDVFRMARENAPAIIFIDEIDAIATKRFDAQTGADREVQR
NCU02260.1	IRVVGSEFVQKYLGEGPRMVRDVFRMARENAPAIIFIDEIDAIATKRFDAQTGADREVQR
FG10769.1 	IRVVGSEFVQKYLGEGPRMVRDVFRMARENSPAIIFIDEIDAIATKRFDAQTGADREVQR
AN2904.1  	IRVNGSEFVQKYLGEGPRMVRDVFRMARENSPAIIFIDEIDAIATKRFDAQTGADREVQR
          	*** **************************:*****************************

MG00908.1 	ILLELLNQMDGFDQTANVKVIMATNRADTLDPALLRPGRLDRKIEFPSLRDRRERRLIFG
NCU02260.1	ILLELLNQMDGFDQTANVKVIMATNRADTLDPALLRPGRLDRKIEFPNLRDRRERRLIFT
FG10769.1 	ILLELLNQMDGFDQTANVKVIMATNRADTLDPALLRPGRLDRKIEFPSLRDRRERRLIFS
AN2904.1  	ILLELLNQMDGFEQTSNVKVIMATNRADTLDPALLRPGRLDRKIEFPSLRDRRERRLIFS
          	************:**:*******************************.*********** 

MG00908.1 	TIASKMSLAPEVDLDSLIVRNDPLSGAVIAAIMQEAGLRAVRKNRYNIIQSDLEDAYSSQ
NCU02260.1	TIAGKMSLAPEVDLDSLIVRNDPLSGAVIAAIMQEAGLRAVRKNRYNIIQADLEDAYSSQ
FG10769.1 	TIASKMSLAPEVDLDSLIVRNDPLSGAVIAAIMQEAGLRAVRKNRYNIIQTDLEDAYSSQ
AN2904.1  	TIASKMSLSPEVDLDSLIVRNEPLSGAVIAAIMQEAGLRAVRKNRYNIIQSDLEDAYSAQ
          	***.****:************:****************************:*******:*

MG00908.1 	VKGTSEDNK~FDFYK
NCU02260.1	VKGTSDENK~FDFYK
FG10769.1 	VKGTGEENK~FDFYK
AN2904.1  	VKTGQEADR2LEFYR
          	**   : :: ::**:
```
