# Supplementary material for: Patterns of Intron Gain and Loss in Fungi
Source: PLoS Biol. 2004 Nov 30;2(12):e422. doi: 10.1371/journal.pbio.0020422 (PMC532390; doi:10.1371/journal.pbio.0020422)
Supplement: Table S1 — Also available at http://genes.mit.edu/NielsenEtAl/. (4.3 MB ZIP). [file pbio.0020422.st001.zip › NielsenEtAl/html/1097.html]

AN2090.1.NCU09227.1.MG10605.1.FG07094.1


```
 CLUSTAL W (1.82) Multiple Sequence Alignments - Introns Inserted


Sequence 1: NCU09227.1	713 aa
Sequence 2: MG10605.1	696 aa
Sequence 3: FG07094.1	688 aa
Sequence 4: AN2090.1	677 aa
Alignment Length: 720 aa
Number Identitical Residues: 322 aa
Alignment Score (without introns) 16793


MG10605.1 	MAKDKK-AKGD-KKAKL~AEKKAKQEKKAEKKAKAKSAKVDDSDAEDVDLDAVLAEYQRQ
NCU09227.1	MAKDKK-GKSESKKAKL~AEKKQKQEKKAEKKAKVKSAKIEGSDAEDVDLDAVLEEYKKQ
FG07094.1 	MAKDKKKNNADTKKAKK0AEKAAKQANKGEKKAKNKAAKIEGSDAEDVDLDEVLEEYRRQ
AN2090.1  	MGKKNK--KSAEHKERV~AAKQSKKADKKEKKHKSK-GKDADSDAEDADLDAILAQYAEE
          	*.*.:*  :.  :* :  * *  *: .* *** * * .*  .*****.*** :* :* .:

MG10605.1 	QEQFHKVTETVSSEAPRPRAASCFLASPSNTNQLLLFGGEYYNGALATFFNDLHVYHIDR
NCU09227.1	QEQFLKVTENVVDEPPRARAASTLMASPSNSNQLLLFGGEYFNGALATFFNDLMVYYIDR
FG07094.1 	QEQFLKVTETVIEAPPRARAASTLMASPHDSNTLLLFGGEYFNGSLAQFYNDLNIYNINR
AN2090.1  	QARFLKVTEVVSG-PPSPRSSATVLASPSNRNELLIFGGEYFDGTLATFYNNLFVYLIDR
          	* :* **** *   .* .*::: .:*** : * **:*****::*:** *:*:* :* *:*

MG10605.1 	DEWRTVTSPNAPLPRSGHAWCRGGNQANSVFLFGGEFSSPKQGTFYHYNDFWRLDAQSKE
NCU09227.1	DEWRCVTSPNAPLPRSGHAWTRGGNDSTGVYLFGGEFSSPKQGTFYHYNDFWRLDPSTRE
FG07094.1 	DEWRCVTSPNAPLPRSGHAWTRAGN-PNHVYLFGGEFSSPKQGTFHHYSDFWRLEPVTRE
AN2090.1  	GEWREVTSPNSPLPRSGHAWCRGGN-TGGIYLFGGEFSSPKQGTFYHYNDFWHLDPSTRE
          	.*** *****:********* *.** .  ::**************:**.***:*:. ::*

MG10605.1 	WEKVEAKG--KTPPARSGHRMTYFK~QYIILFGGFQDTANQTRYLADLWLYDTQHFVWFN
NCU09227.1	WARIETKG--KTPPARSGHRMTYYK~NYIILFGGFQDTANQTKYLQDLWLYDTQNFVWHS
FG07094.1 	WTKIEFKGKDKSPSARSGHRMTYWK~QYIILFGGFQDTSNQTKYLSDLWIFDTVNFVWHS
AN2090.1  	WSRIETKG--KGPPARSGHRMTYFK0NYIILFGGFQDTSQQTKYLQDLWIYDCSKYTWYN
          	* ::* **  * *.*********:* :***********::**:** ***::*  ::.*..

MG10605.1 	PTLPPAQLKPDARSSFTFLPHEQGAVLYGGYSRVKATVAANKGAKPG----SQGQKNILK
NCU09227.1	ITPPAAQLKPDARSSFTFLPHDQGAVLYGGYSRVKATVAAGKQTKQGGGGGAGGSKNILK
FG07094.1 	PQLPPAQLKPDPRSSFTLLPHEQGAVLYGGYSRVKSTVNVKQKGNKGP---SQAQRNVLI
AN2090.1  	PVLSTASQKPDPRSSSSFLPHESGAVLYGGYSRVKVTAGAGGKSAKG-----GPQRMTMK
          	   ..*. ***.*** ::***:.************ *. .      *       .:  : 

MG10605.1 	PMVHQDCFFLRITPPGPEAAAGAAPTVRWEKRKKPANTPTPTRAGTTMAYHHRGKRGILF
NCU09227.1	PMVHQDCFFLRITQPPADAPPNTGPTVRWEKRKKPANTPNPTRAGATMAYHKG--RGIMF
FG07094.1 	PKVHEDCFFLRISQPATDASPNTPPAVRWEKRKKPANAPNPTRAGATMAWHKG--RGILF
AN2090.1  	PMVHQDTWFLRITPPAPEAPPSTPPTVRWERRKKPANSPNPARAGATMAYHKG--RGIMF
          	* **:* :****: * .:*...: *:****:******:*.*:***:***:*:   ***:*

MG10605.1 	GGVHDVEESEEGMESEFFNGLFAWNIERNRFFPLALRKVRQGGGKKG---GEQQQRVGRR
NCU09227.1	GGVHDVEESEEGMESEFFNQLFAWNIERNRFFPMALKKARAPGKKNGAGNGEGRERVGRR
FG07094.1 	GGVHDVEASEEGMDSEFFNQLFAWNIERNRFMPLSLRKARQ--QKKA----AAEPRGGRR
AN2090.1  	GGVHDVELSEEGIESEFFNTLFAWNTDRNRFFPLTLRRPKNTGKSQQ-----GNQAAKSR
          	******* ****::***** ***** :****:*::*:: :  . .:       .     *

MG10605.1 	GRAQANQEELLKQLAALETGAS-LDNLDDLELK---------QDESKEEPKEPMREMPVS
NCU09227.1	DRARQNEEELLRQLAALQAGAKGVDGADEMEIDALLKKQRGEEEEEEKDEKRKVRDMPVS
FG07094.1 	GRAQANEDELLRQLAALESGAS-LDDADDIELA---------KKEEEQDDEKPAREMPVT
AN2090.1  	NRGKATEEELLQNLKALEAKKGIRVDENDDDNE--------FLPKEDEEPVQPEKPAIVR
          	.*.: .::***::* **::      . :: :             :..::  .  :   * 

MG10605.1 	MEFPHPRFNAQLAIQDDVLYIYGGTFEKGDREFTFDDLYAVDLGKMDGCKEIFSRQTEDW
NCU09227.1	MEMPHPRFNAQLAVQDDVLYIYGGTFEKGDREFTLDDMYAIDLGKMDGCKEIFKREGDDW
FG07094.1 	MEPPHVRFNAQLAIQDDVLYIYGGTFEKGDREFTFDDLYAIDLGKLDGCKEVFSRPVEDW
AN2090.1  	FEMPHMRFNAQLAVQEDTLFIFGGTYEKGDREFTFNDMYSIDLGKMDGVKEIFYNEPGNW
          	:* ** *******:*:*.*:*:***:********::*:*::****:** **:* .   :*

MG10605.1 	V0QSEDEDDDDEDDDDEDEDEEDEEEEEEEDSDKKSKKFTPSKRGKKPADQDSNADTDSA
NCU09227.1	I0ESEDEDDDEEDDEDEDSDEEADDEEDAMEVDDKKAKYTPSDRKKKKAKAG--EETPVA
FG07094.1 	I0ESDDEDDDDEDEEDEEDEEEDEEADEEASQQLR----TPSKRKKKQDEIS--EVSSEV
AN2090.1  	H~LLNEADSDEEMDEDDEEGEEEEEAEEEDAMSLD----TASAAPTETTDVT----VPSV
          	    :: *.*:* ::*::. ** :: ::    .      *.*   .:  .         .

MG10605.1 	LGSSIGTEEDETEDFTEARVDDGLPHPR0PFESRREFFVRTSAEWQEILMTNLRWKNIQP
NCU09227.1	ENAPIAAAPEEEEESEETRVDDGLPHPR0PFESRRDFFQRTSAEWQEILMTNLRWKGIQP
FG07094.1 	SSEPSTPSEEDDTETSATSVDDGLPHPR0PFETRREFFVRTSNEWQEILMTSLRWKNIQP
AN2090.1  	TRDLEQLDIEEQD--AEPSIQDSRPLPR~PFETLREFFSRTSEEWQQILLETLNERGVAV
          	         ::      . ::*. * ** ***: *:** *** ***:**: .*. :.:  

MG10605.1 	ETLAVKEIKAKAFELSEEKWWDCREEITALEDEQEAAGIGEVVSLADRGETGG--AAGGL
NCU09227.1	ESLAVKEIKTKAFELSEEKWWDCREEITALEDEQEAAGIGEVVSLADKAGAGGGGGSGGA
FG07094.1 	ETMPIKEIKAKAFELSEEKWWDCREEIVALEEEQEAAGIQEVVSLADRGDASA---AGGA
AN2090.1  	EKN-IKELRKDAFNLAEEKWWDSREEIMALEDEQEAAGIGEVISIADRAENAG----GAG
          	*.  :**:: .**:*:******.**** ***:******* **:*:**:.  ..    *. 

MG10605.1 	RRR-
NCU09227.1	GRRR
FG07094.1 	RRR-
AN2090.1  	RRR-
          	 **
```
