# Supplementary material for: Patterns of Intron Gain and Loss in Fungi
Source: PLoS Biol. 2004 Nov 30;2(12):e422. doi: 10.1371/journal.pbio.0020422 (PMC532390; doi:10.1371/journal.pbio.0020422)
Supplement: Table S1 — Also available at http://genes.mit.edu/NielsenEtAl/. (4.3 MB ZIP). [file pbio.0020422.st001.zip › NielsenEtAl/html/1098.html]

AN9064.1.NCU00891.1.MG01176.1.FG04922.1


```
 CLUSTAL W (1.82) Multiple Sequence Alignments - Introns Inserted


Sequence 1: NCU00891.1	383 aa
Sequence 2: FG04922.1	353 aa
Sequence 3: MG01176.1	353 aa
Sequence 4: AN9064.1	359 aa
Alignment Length: 387 aa
Number Identitical Residues: 178 aa
Alignment Score (without introns) 9401


MG01176.1 	MAS------~--ATEGKS0PHDVCFEERPMPKLASEHDVLVAVNYTGICGSD~VHYWHHG
NCU00891.1	MATDG--KS0NLSFVLNK~PLDVCFQDKPVPKINSPHDVLVAVNYTGICGSD~VHYWLHG
FG04922.1 	MS------S0NLSFVLNK~PGDVSFEERPKPSLASPHDVLVAVNYTGICGSD~VHYWVHG
AN9064.1  	MSSQTPTAQ0NLSFVLEG~IHRVKFEDRPIPKLKSPHDVIVNVKYTGICGSD0VHYWDHG
          	*::. .: . . :   :     * *:::* *.: * ***:* *:******** **** **

MG01176.1 	SIGDFVVKDPMVLGHESAGTVVEVGSAVKTLQVGDRVALEPGYPCRRCRDCLAGRYNLCP
NCU00891.1	AIGHFVVKDPMVLGHESAGTIVAVGDAVKTLSVGDRVALEPGYPCRRCVHCLSGHYNLCP
FG04922.1 	SIGKFVVEDPMVLGHESAGTVVEVGDKVKTLKAGDRVALEPGYPCRRCQNCLAGKYNLCP
AN9064.1  	AIGQFVVKEPMVLGHESSGIVTQIGSAVTSLKVGDHVAMEPGIPCRRCEPCKAGKYNLCE
          	:**.***::********:* :. :*. *.:*..**:**:*** *****  * :*:**** 

MG01176.1 	EMRFAATPPYDGTLAGFWTAPADFCYKLPESVSLQEGAMIEPLAVGVHIVRQAKVSPGQS
NCU00891.1	EMRFAATPPYDGTLTGFWTAPADFCYKLPETVSLQEGALIEPLAVAVHITKQAKIQPGQT
FG04922.1 	DMVFAATPPYHGTLTGFWSAPADFCFKLPDNVSLQEGALIEPLAVAVHIVKQARVKPGDS
AN9064.1  	KMAFAATPPYDGTLAKYYTLPEDFCYKLPESISLPEGALMEPLGVAVHIVRQANVTPGQT
          	.* *******.***: ::: * ***:***:.:** ***::***.*.***.:**.: **::

MG01176.1 	VVVMGAGPVGLLCAAVARAFGATTVVSVDIVESKLEVAKQIAATHTYLSQRISPQDNAKA
NCU00891.1	VVVMGAGPVGLLCAAVAKAYGASKVVSVDIVPSKLEFAKSFAATHTYLSQRVSPEENARN
FG04922.1 	VVVMGAGPVGLLCAAVAKAYGASKIVSVDIVQSKLDFAKDFASTHVYASQRIAPEENAKN
AN9064.1  	VVVFGAGPVGLLCCAVAKAFGAIRIIAVDIQKPRLDFAKKFAATATFEPSKAPATENATR
          	***:*********.***:*:**  :::***  .:*:.**.:*:* .: ..: .. :**  

MG01176.1 	LIAAAGLEDNGGADVVIDATGAEPSIQTSIHAVRVGGSYVQGGMGKPDITFPILAFCCKE
NCU00891.1	IIAAADLGE--GADAVIDASGAEPSIQAALHVVRQGGHYVQGGMGKDNITFPIMALCIKE
FG04922.1 	ICDLAGLPD--GADVVIDASGAEPSIQASIHVLKNGGSYVQGGMGKADITFPIMAFCIKE
AN9064.1  	MIAENDLGR--GADVAIDASGVEPSVHTGIHVLRPGGTYVQGGMGRSEMNFPIMAACTKE
          	:    .*    ***..***:*.***:::.:*.:: ** *******: ::.***:* * **

MG01176.1 	VTASGSFRYSAGDYRLAIDLVANGKVNLKALITETVPFDKAQEAFTKVSEGQVIKVLIAG
NCU00891.1	VTASGSFRYGSGDYRLAIQLVEQGKVDVKKLVNGVVPFKNAEEAFKKVKEGEVIKILIAG
FG04922.1 	ATASGSFRYGAGDYPLAVELVATGKVDVKKLITGIVDFKQAEEAFKKVKEGEAIKVLIKG
AN9064.1  	LNIKGSFRYGSGDYKLAVQLVASGQINVKELITGIVKFEDAEQAFKDVKTGKGIKTLIAG
          	 . .*****.:*** **::**  *::::* *:.  * *..*::**..*. *: ** ** *

MG01176.1 	PNEK--------------------------
NCU00891.1	PNEDVEGSLDTTVDEKKLNEAKACGGSGCC
FG04922.1 	PNEQ--------------------------
AN9064.1  	PGAA--------------------------
          	*.
```
