# Supplementary material for: Patterns of Intron Gain and Loss in Fungi
Source: PLoS Biol. 2004 Nov 30;2(12):e422. doi: 10.1371/journal.pbio.0020422 (PMC532390; doi:10.1371/journal.pbio.0020422)
Supplement: Table S1 — Also available at http://genes.mit.edu/NielsenEtAl/. (4.3 MB ZIP). [file pbio.0020422.st001.zip › NielsenEtAl/html/1099.html]

AN6874.1.NCU03503.1.MG02384.1.FG01640.1


```
 CLUSTAL W (1.82) Multiple Sequence Alignments - Introns Inserted


Sequence 1: NCU03503.1	471 aa
Sequence 2: MG02384.1	649 aa
Sequence 3: FG01640.1	473 aa
Sequence 4: AN6874.1	478 aa
Alignment Length: 659 aa
Number Identitical Residues: 167 aa
Alignment Score (without introns) 8656


MG02384.1 	MEMNSRLEASVSIYYIKLYFEAGLSHAKKFKICLMTCGTCDWSLFIDVVPSNFNSNILDN
NCU03503.1	------------------------------------------------------------
FG01640.1 	------------------------------------------------------------
AN6874.1  	------------------------------------------------------------
          	                                                            

MG02384.1 	WQGFWCNYCHFCMHA0ACDNWMEVVELYHIVPISFAT0LASIHVTFELKQIWLNLHHPPL
NCU03503.1	------------MAA~GVD------------------~----------------------
FG01640.1 	------------MAP~DAK------------------~----------------------
AN6874.1  	------------MAE~KLLP-----------------~----------------------
          	            *                                               

MG02384.1 	FDARLSSKLPSSNMAKEKRKKPPTGSEGKTIVFLHPDLGIGGAERLVVDAAVGLQKRGHR
NCU03503.1	----------------EKDKK--------TIVFLHPDLGIGGAERLVVDAAVGLQNRGHK
FG01640.1 	----------------DKG----------SIVFFHPDLGIGGAERLVVDAAVGLQERGHR
AN6874.1  	-----------------KP---------ASIIIIHPDLGIGGAERLIIDVALALQNSGHK
          	                 *           :*:::************::*.*:.**: **:

MG02384.1 	VVIFTSHCDPSHCFDEARDG~TLDVRVRGNTIVPPSILSRFSILCAILRQIHLILQIAIF
NCU03503.1	VVIFTSHCDPRHCFDEARDG1TLDVRVRGNSIIPPSLLGRFSILCAILRQLHLILQITLL
FG01640.1 	VVIFTNHCDPKHCFDECRDG1TLDVRVRGHWLIPMSILSRLTILCAILRHVHLLIHIALT
AN6874.1  	VTVYTSHRDKSHCFEEARDG~TLEVRVRGDSFFPPSIRGRFVVLLAILRQLCLTFELLRE
          	*.::*.* *  ***:*.*** **:*****. :.* *: .*: :* ****:: * :.:   

MG02384.1 	SSELADLQP-DGFFVDQLSAGLPLLAGLSPQLNSPP--PHILFYCHFPDLLLAQ-----G
NCU03503.1	TSELRTLSP-SAFFVDQLSAGLPLLKLLVP--TSP-----IFFYCHFPDLLLVQ-----G
FG01640.1 	G-ELQDLNP-RAFIVDQLSAGLPLMRYIAP--DSP-----ILFYCHFPDLLLAQ-----G
AN6874.1  	VAQTENENADQVFIVDQVPACVPLLKMLGPRWFASKGKQRILFYCHFPDQLLARRDGGSA
          	 ::    ...  *:***:.* :**:  : *   :. ..  *:******* **.: ...:.

MG02384.1 	RQKWWKRLYRVPFDRLEEWSMGFADAIAVNSGFTKGVVSRTWPALAAKTDLQVVYPCVDV
NCU03503.1	RQTWYKRLYRLPFDTWEEWSMGFADSIAVNSSFTKGIVSHTWPSLASKRSLEVVHPCIDV
FG01640.1 	RESALKRLYRRPFDWLEEWTMGFASAVAVNSGFTKGVVNQTWPNLKKRTDTKVVYPCVDT
AN6874.1  	LLQLLKGLYRYPFDWFEGWAMSASDRVVANSTFTKSVVRGVFGAEKLG-DVRVVYPCVDT
          	     * *** ***  * *:*. :. :..** ***.:*  .:       . .**:**:*.

MG02384.1 	RVPGEPPKEGQSAAEGNKEDVTSELDWKESRILLSINRFERKKNVALAIKAYAGLPKEKR
NCU03503.1	RSTSD-------SSQNPNDDDKDVLPWTKTGIILSINRFERKKDIALAIKAFASLSPEQR
FG01640.1 	GVKEK-------EDAGNDGD----IPFKGEKIILSINRFERKKDIGLAIKAFAAIPEAER
AN6874.1  	AAKEK--------SEKDVGT-----IWEGKKILLSVNRFEKKKDLALAIRAYHGLG-EKR
          	    .                     :    *:**:****:**::.***:*: .:   :*

MG02384.1 	AGVKLVIAG1GYDNRVPENVSYHTELTNLAD--SLDLRHGTTKTVVTALGLARDPSIDVL
NCU03503.1	GKAKLIIAG~GYDNRVHENVSYHMDLVDLAEGAPYHLKTATAKTVVSALNTS--PDVEVL
FG01640.1 	KGCRLILAG1GYDPRVSENVQYHAELEALAS--SHGLEHLTTKTLITALSAP--TSVPVL
AN6874.1  	KGVRLVIAG1GYDPRITENVQYHKELDALAT--SLGLQTATSKTVPSALSIP--SSIDVL
          	   :*::** *** *: ***.** :*  **   .  *.  *:**: :**. .  ..: **

MG02384.1 	FLQSVPSALKTALLRSAKLLLYTPAEEHFGIVPLEAMLAGLPVLACDSGGPTETVLEGVT
NCU03503.1	FLLSVPNTLKEILLRSAKLLVYTPSNEHFGIVPLEAMLRGVPVLAANNGGPTETVVEGET
FG01640.1 	FLLSIPNSLKATLLRSARVLLYTPKNEHFGIVPLEAMLARTPVLAANSGGPVETIVDGET
AN6874.1  	FLPSVSSAFRDSLLAKSSLLLYTPVNEHFGIVPIEAMRAGIPVLASNTGGPLETIVEGKT
          	** *:..:::  ** .: :*:*** :*******:***    ****.:.*** **:::* *

MG02384.1 	GWLRSPEKPEEWTKVVDMVLNELSDEQKDDMRAAGPKRVKENFAEEQMAERLDRIFDDME
NCU03503.1	GWLRDPNDVGEWAKVMDKVLNGMGEEELKRMGKKGVERVKGRFADTQMAERLEEIIERMP
FG01640.1 	GWLRSPDDVDEWAKVVRLAL-ELGDDQIKSMGDRSEARVKDMFGRAQMASRFNEILDDIV
AN6874.1  	GWLRDVDDVPAWTGVIEKVLYQLGADELRQMSVAAKERVEAEFSLHAMGDRLEGEIGKML
          	****. :.   *: *:  .*  :. ::   *   .  **:  *.   *..*::  :  : 

MG02384.1 	QSAS0GYNWAGDKWDFSSTLSVLIPCVKSQEIPYHDICISDDLIWSRCVMTPIRVPKVLE
NCU03503.1	K---~-----GD-----AAQSGMILLVVGAAVAAVAGVIS-AVYWKLW------------
FG01640.1 	E---~------------KKASSSVRTVVNVAAVFGLGLLG--LVASALIARMGKQAEPSE
AN6874.1  	S---~---------------TERRQFNGAQQALLLLGMLG--VVFAVLVGLVLAWVGFV-
          	.                   :                 :.  :                 

MG02384.1 	MNAG
NCU03503.1	----
FG01640.1 	FSV-
AN6874.1  	----
          	
```
