# Supplementary material for: Patterns of Intron Gain and Loss in Fungi
Source: PLoS Biol. 2004 Nov 30;2(12):e422. doi: 10.1371/journal.pbio.0020422 (PMC532390; doi:10.1371/journal.pbio.0020422)
Supplement: Table S1 — Also available at http://genes.mit.edu/NielsenEtAl/. (4.3 MB ZIP). [file pbio.0020422.st001.zip › NielsenEtAl/html/11.html]

AN6181.1.NCU00706.1.MG00546.1.FG10181.1


```
 CLUSTAL W (1.82) Multiple Sequence Alignments - Introns Inserted


Sequence 1: AN6181.1	106 aa
Sequence 2: FG10181.1	116 aa
Sequence 3: MG00546.1	119 aa
Sequence 4: NCU00706.1	117 aa
Alignment Length: 136 aa
Number Identitical Residues: 78 aa
Alignment Score (without introns) 3299


MG00546.1 	----------M~VNIPKTRRTYCAGKECKKHTNHRVTQYKAGK~ASSFAQGKRRYDRKQS
NCU00706.1	----------M~VNVPKTRKTYCAGRSCGKHTLHKVTQYKAGK~ASAFAQGKRRYDRKQS
FG10181.1 	MTRWLTLWIQQ~VNIPKTRNTYCKGKECRKHTQHKVTQYKAGK~ASLFAQGKRRYDRKQS
AN6181.1  	----------M0VNVPKTRKTYCKGKECHKHTQHKVTQYKAGK0ASLFAQGKRRYDRKQS
          	            **:****.*** *:.* *** *:******** ** *************

MG00546.1 	GYGGQTKPVFHKKAKTTKKIVLRL~ECTVCKTKCQLPIKRCKHFELG2TQLLTRTFSWLC
NCU00706.1	GYGGQTKPVFHKKAKTTKKVVLRL0ECSVCKQKKQLPLKRCKHFELG2LQFP--------
FG10181.1 	GYGGQTKPVFHKKAKTTKKVVLRL~ECVKCKTKLQLALKRCKHFELG2------------
AN6181.1  	GYGGQTKPVFHKKAKTTKKVVLRL~ECTQCKTKKQLALKRCKHFELG2------------
          	*******************:**** **  ** * **.:*********             

MG00546.1 	SGDKKTKGAALVF-------
NCU00706.1	-AGRETETPRWRLPTELDAT
FG10181.1 	-GDKKTKGAALVF-------
AN6181.1  	-GDKKTKGAALVF-------
          	 ..::*: .   :
```
