# Supplementary material for: Patterns of Intron Gain and Loss in Fungi
Source: PLoS Biol. 2004 Nov 30;2(12):e422. doi: 10.1371/journal.pbio.0020422 (PMC532390; doi:10.1371/journal.pbio.0020422)
Supplement: Table S1 — Also available at http://genes.mit.edu/NielsenEtAl/. (4.3 MB ZIP). [file pbio.0020422.st001.zip › NielsenEtAl/html/110.html]

AN0297.1.NCU02948.1.MG01569.1.FG01403.1


```
 CLUSTAL W (1.82) Multiple Sequence Alignments - Introns Inserted


Sequence 1: MG01569.1	204 aa
Sequence 2: FG01403.1	204 aa
Sequence 3: NCU02948.1	244 aa
Sequence 4: AN0297.1	204 aa
Alignment Length: 244 aa
Number Identitical Residues: 111 aa
Alignment Score (without introns) 5632


MG01569.1 	MAPKIAIVY0YSMYGHIKQLAEAEKAGIEKAGGTADLFQ2IEETLPEEVLSKMGAPAKPT
NCU02948.1	MAPKIAIVY0YSMYGHIRQLAEAAKAGIEKAGGTADLYQ2VPETLSDEVLAKMYAPPKPT
FG01403.1 	MAPKIAIVY0YSMYGHIKQLAEAEKAGIEKAGGTADLFQ~VPETLPEEVLAKMHAPPKAT
AN0297.1  	MAPKIAIVF0YSMYGHVEKLAQAELKGIQAAGGNADLYQ~IQETLPEEVLAKMHAPPK-S
          	********: ******:.:**:*   **: ***.***:* : ***.:***:** **.* :

MG01569.1 	DVKVLSD0PSTLEAYDAFLFGIPTRYGNFPGQ2WKAFIDKTGKQWAAGGFYGK~YAGIFI
NCU02948.1	DIPVIED~PAILKEYDGFLFGIPTRYGNFPAQ~WRAFWDKTGGLWATGGLYGK~AAGLFI
FG01403.1 	DVPTLND~PSILESYDAFLLGIPTRYGNFPAQ~WKAFWDQTGKQWASGGFWGK~MAGIFV
AN0297.1  	SVPTLEK~PEQLLEYDAVLFGIPTRYGNFPAQ~WKAFWDRTGGIWATGGFFGK2YAGLFV
          	.: .:.. *  *  **..*:**********.* *:** *:**  **:**::**  **:*:

MG01569.1 	STASQGGGQESTAIAAMSTFAHHGIIYVPLGYTKCFGIQTDLSEARGGSPWGAGTFA~GG
NCU02948.1	STAGLGGGQESTAIAAMSTLAHHGIIYVPLGYAKVFGELSDLSAVHGGSPWGSGTLS~GG
FG01403.1 	STASQGGGQETTAQNAISTLTHHGIIYVPFGYAKAFGTLTDLSEVRGGSAWGAGTFA~GA
AN0297.1  	STGTLGGGQESTAIAAMSTLTHHGFLYVPLGYKTAFPLLSNLEEIHGGSAWGAGTFA0GA
          	**.  *****:**  *:**::***::***:** . *   ::*.  :***.**:**:: *.

MG01569.1 	DGSRQPSEKELELARIQGEEFYNHVSKATN~-----------------------------
NCU02948.1	DGSRQPSESELKVAGIQGEEFYNTLSKLTA1SQPKPAPQQKKEPVKQEQQPTKQKETKEG
FG01403.1 	DGSRQPSAKELELAQVQGEHFYQTVAKFTG~-----------------------------
AN0297.1  	DGSRQPTKLELEIAETQGKSFYEHVSRVNF~A----------------------------
          	******:  **::*  **: **: ::: .  :                            

MG01569.1 	-----------
NCU02948.1	PCGLPSKCVIL
FG01403.1 	-----------
AN0297.1  	-----------
          	
```
