# Supplementary material for: Patterns of Intron Gain and Loss in Fungi
Source: PLoS Biol. 2004 Nov 30;2(12):e422. doi: 10.1371/journal.pbio.0020422 (PMC532390; doi:10.1371/journal.pbio.0020422)
Supplement: Table S1 — Also available at http://genes.mit.edu/NielsenEtAl/. (4.3 MB ZIP). [file pbio.0020422.st001.zip › NielsenEtAl/html/1100.html]

AN3660.1.NCU03924.1.MG01032.1.FG08400.1


```
 CLUSTAL W (1.82) Multiple Sequence Alignments - Introns Inserted


Sequence 1: MG01032.1	840 aa
Sequence 2: FG08400.1	847 aa
Sequence 3: NCU03924.1	838 aa
Sequence 4: AN3660.1	1261 aa
Alignment Length: 1287 aa
Number Identitical Residues: 223 aa
Alignment Score (without introns) 14584


MG01032.1 	MAEPEST---LFSGCRIAFVPSSTLKPSTIAE0ATKIVLRNGGEVLEPESNGKLA-VHEA
NCU03924.1	MAGENEKPG-LLDGCSIYFVQSKSLSASLISE0FSKLVHKHGAQLLEPTKDGKIP-LEHA
FG08400.1 	MAG-------VFAECAIAFVTSTELAPKLIGE0LSTILEDNGATICEPRRDGSLP-IEKV
AN3660.1  	MAGHQDDNLRLFDQLKVCIVCSKDLSPDAAHQ0LASTLETHGGEPVVYEPPADFPDIAAF
          	**  ..    ::    : :* *. * ..   :  :. :  :*.        ..:..:   

MG01032.1 	THIVSDTIDFEEYTAAGECLVPVVTCNWITNSLMKRKLAALRPHSPDPRLIFHAVNVTCA
NCU03924.1	THIVSNTIDFEQYHDALQYMIPVVNSNWIKITLARNKVAQVRPYSPDPRMIFSNVILTCA
FG08400.1 	THIISNTIDFPQFTEAQAIMIPVVTTQWITHSIARRKQSQIRPFSPDPRMIFSEVVVTCA
AN3660.1  	SHIMSTTIDFPQFDAAKDALIPVIKPQWMHASLAKRRLANVRQYSPDPRLFLNDVVVTCG
          	:**:* **** ::  *   ::**:. :*:  :: :.: : :* .*****:::  * :**.

MG01032.1 	DLPPTDKESIIGATIALGGTESKDLTKLTTHICALSYDHPKVALAVSKGLKCKVVLPHW2
NCU03924.1	DIPESDKETIIGATMALGGMESKDLTRQTTHICALSMDHEKCQEAQKKNPKCKIVLPHW~
FG08400.1 	DLPETDKECIAGAVMALGGQESKDATRMTTHICALSMDHPKLQTALQKGWKGKVVLPHW2
AN3660.1  	DIPEGDKDAIIGGVLAKGGLYNPRLTGMCTHLVDLTIDSDKAKQVRARNLNVKIVLPHW~
          	*:*  **: * *..:* **  .   *   **:  *: *  *   .  :. : *:***** 

MG01032.1 	FDDCFKLGKRIDEGPYQLPDPEILKAGESTKDIAVPCSQHLEGATTTMPTTAPENTKS--
NCU03924.1	FDDCFRLGRRISEAPYMLPNPEILRT-GPDEDITIPPSEAVEGATSVIPSAAMPGRER--
FG08400.1 	FDDCFKLGKRIDEGPYVLPDPEILKK-SPEDDVKIPTNENLLGATSHAPSYLPLPPDSDV
AN3660.1  	FDDCLKLGRRIDERPYKLPDPEILRA-APDAPIRSAESRDIIGASTPEPSKLPTPLTSP-
          	****::**:**.* ** **:****:   .   :  . .. : **::  *:          

MG01032.1 	--RPASVFNHRTIKLSVDLPLNERFLAIIRAHITKGGGKITDDVEECDIYICHYREGDDY
NCU03924.1	--EKIVVFAQKKVMISQDLPINARLRNILNNLIEQSDGEVVNDVDACDMYVCQYRDGDEY
FG08400.1 	ARPPVTIFQDRRVMLSKDLSLTERLSKVIQEIIINGGGKVVDEVEDCDTFICQYRDGPQY
AN3660.1  	VKPKLSVFHGKVIMLSSDLEIGSHLRDTISAIVEENGGKVTTDVSEATTFICRFRDGFNY
          	      :*  : : :* ** :  ::   :   : :..*::. :*. .  ::*::*:* :*

MG01032.1 	IYAAQKKKHVGNIAWLLYLVTHDEWTSPLRRLLHYPVPRGGIPGFEGCKITVSNYGGEAR
NCU03924.1	IRAAQQGKDVGNLAWLYYLIVHNEWTRPTRRLLHYPIPRDGIPGFTGMKITLSNYGGDAR
FG08400.1 	VRAAQSCKEVGNLAWLFWLIVHNDWTSPLRRLLHYPIPRDGIPGFKELRITVSNYGGEAR
AN3660.1  	RVASRLNKDVGNLSWLYHLMTYDSWTSPYRRLLHYPIPKTPIPGFEKFKISLSNYVGEAR
          	  *::  *.***::**  *:.::.** * *******:*:  ****   :*::*** *:**

MG01032.1 	TYLENLIRACGAEFTKTMKQDNTHLITARNSSEKCEAALDWNVTMVNHLWIEESYAKCEM
NCU03924.1	VYLENLITAAGATYTKTMKADNTHLITARMNGEKCEAAKEWNIEIINHLWIEDSYAKCEV
FG08400.1 	IYLENLIRACGAEFTKTMKSENTHLITARDTSEKCKAAPEWGIHVVNHLWIEESYAKCEI
AN3660.1  	SYLEHLITATGAECTKTLRQENTHLVTAHDNSEKCSAAREWNIHVVNHLWLEECYAQWRL
          	 ***:** * **  ***:: :****:**: ..***.** :*.: ::****:*:.**: .:

MG01032.1 	QHPSNNKKYTTFPQRTNLSEVIGQTSLDETVLREVYYPGGEDVVSEAGSDT----ASEQE
NCU03924.1	Q-TFSNPRYQHFPPRTNLGEIIGSTFFDEQRLREVYFPGGEEVLSTAAKKRRKANDAANK
FG08400.1 	T-PVSTKKYTHFPPRTNLGEIIGQTFFDESRLRDKYYPGGEEKMSPAAKRKRKILEAAEQ
AN3660.1  	L-PESNNRYTHFPRRTNLGEVVGQTRLDRSALESMFFAS--EEASSEPSRK-----AMQK
          	  . .. :*  ** ****.*::*.* :*.  *.. ::..  :  *   .       : ::

MG01032.1 	NEVEAGEEIAEVAVPVRAKPAAKGKKSPLSKADNSNKAALATPARST--RSGGSGKENVT
NCU03924.1	NVYASNLAETDAGTANKSSPLG-GKTAIKPPRGAQNKQNIQTPAKTT--RTTR-GKENDT
FG08400.1 	NAYPRGPAEGVVIGQADSEDVEMEDVEEESEKPTKKKAATKASSVATPIRSRHAGKENDT
AN3660.1  	REQNTAPGKPLGSTDIDDNMVS-NTTNATPAGRSKKNTNLHTPSHLY---LASDGKENDT
          	.                 .          .    .::    :.:          **** *

MG01032.1 	PASRPSTSRSAKSTALNKLHVLAPDIALYEKEKKRMSNGP---FGGKRAASELEKQQAEA
NCU03924.1	PSVMSSGSRSAKDKALSKLHQLAPDIALYEKEKKRTAKDGP--WGGKRAADQIDRDR-AA
FG08400.1 	PSVISTGGRSAKAKAQAALLGLSDDIALYEKERKRNAKGGSAIWGGKRAADQAEK---DI
AN3660.1  	PS--STSSRKSKEAATARLHEIAPDIALYEKEKKR---VGGVIYGGRRKTDEGRVVL--N
          	*:  .: .*.:*  *   *  :: ********:**        :**:* :.:        

MG01032.1 	AEKEKEKKDAAQDDEDGERPAKRQRSSRPPVQYRICLTGWKRWLAPDVKSVSQEDKER0R
NCU03924.1	KESSPAEDGGEEDETEQKRPAKKARVS-SSPEMRICLTGYKRWVNDKMR----EEADR0-
FG08400.1 	TKTKSATPEAEEDATTAKRPAKKAKPTLPGVTMRVILTAFNRWVGDKAK----EDRDR0K
AN3660.1  	SKKRSSMDAQTDSEAEDTTEAKRQKKSKPPITMHLLITGYQRWVGNMKK----EDAEK0R
          	 :.        :.       **: : : .    :: :*.::**:    :    *: ::  

MG01032.1 	KLRQMGINIVQEGQPCDYLAAPRVVRTQKFLTTLARGPELISDQFLVDALENGELPNVED
NCU03924.1	--------IVQDNVPCDYLAAPRMVRTMKFLRCLARGPDIISSDYVTACVEAGKVLPPKD
FG08400.1 	KLREMGIQIVGEGQPCDYLAAPNVVRTVKFLCALSRGPSVISSDFIEQALDTGNLPDVEG
AN3660.1  	QLRELGIMVVQDARKCSHLAAPSVLRTPKFVNAIAYSPVIVQVEFITQCLKKNKLLDPED
          	.  . .  :* :   *.:**** ::** **:  :: .* ::. :::  .:. .::   :.

MG01032.1 	YPLEDANYP-----DIQKSIARARQNKGKLLRSVPIYCTAEVPHGPNAFKAIAEANGAIF
NCU03924.1	YLLVDKESEDRFGVTLQTAISRARANRGRLLWGVPVFCTEEIKNGVQSYQTIAEANGAIF
FG08400.1 	FILKDKDAEKKYKIDLEKSVARARANRGKLLLGVPIYCTEKIRNGPDSYRAIAEANGAIF
AN3660.1  	FLLDDKEAK-KFGFSLEQARVNARANKNKLLRGHHIYCVETIRGGFDAFKSIVDANGGEC
          	: * * :        :: :  .** *:.:** .  ::*.  :  * :::::*.:***.  

MG01032.1 	KIYRARSGVTIRPTTAEEDGGAPPDPVYLLSGDTAAEKLLWPKFEHMAREGHMEPRIVDP
NCU03924.1	RVFRGRGGSVIKPTTPEEDGGAPPDPVYLLTSPSPAEKALWPKFEEMAKKGNMEPRIVVS
FG08400.1 	KIYRARSGTTIRPTTAEEEGNAPPEPVYLLSSDARDEKALWDRFREMARGGNMEPRVVVP
AN3660.1  	NLFRGRVSYHTARKDSDEEKQSSRKDIYLLSSAAPEHQKLWPRFRQLVHDMGKTPRIVRV
          	.::*.* .     . .:*:  :. . :***:. :  .: ** :*..:.:     **:*  

MG01032.1 	DWLLDVAMRQRLDFDEKFMVRLRDQ-----~-----------------~-----------
NCU03924.1	DWLLDVAMKQELTFDPRYLAVRYFAENPPF~-----------------~-----------
FG08400.1 	DWLLDVAMAQQVRFDEDFLVEKYYELE---~-----------------~-----------
AN3660.1  	DWLLDMAMSQELRVAEDYELSEGMIEQSEE2CGGADTTIESGMTTSGV0EAEMRCGEDQP
          	*****:** *.: .   :        ..   ...:.:: .:. :::.  .:.  ......

MG01032.1 	------------------------------------------------------------
NCU03924.1	------------------------------------------------------------
FG08400.1 	------------------------------------------------------------
AN3660.1  	APLRQSDVRSRSEENSAFTLNKYSNPQRQRTEVHNPYIMGATAKKQKREVYRNKVAAVTS
          	:.  .:.  : :...:: : .. :... . :.  ..   .:::.... .   .. :: ::

MG01032.1 	----------------------------------~-------------------------
NCU03924.1	----------------------------------~-------------------------
FG08400.1 	----------------------------------~-------------------------
AN3660.1  	AESGEAALAALKLPQKKYYRQRAHANPFSDHLLN2PLSPAHMDWATHFPAFVNPDPSQTN
          	:.:..:: :: . ....   . : :.. :.   . . :.:  . ::  .:  ....:.:.

MG01032.1 	--------------------------------------~---------------------
NCU03924.1	--------------------------------------~---------------------
FG08400.1 	--------------------------------------~---------------------
AN3660.1  	LAGTRKLIKDVEVVDIGCGFGGLLVGLAPVLPDTLMVG1MEIRVQVTEYLTNRIKALRHQ
          	 :.: .  .. .  . ... ..   . :.  ..:   .  .   . :.  :.  .:   .

MG01032.1 	------------------------------------------------------------
NCU03924.1	------------------------------------------------------------
FG08400.1 	------------------------------------------------------------
AN3660.1  	QALKLQLQQSSAASTPAAAPSPSPAPVPETPTANDPSVDDSEIFPSTLIPGGYQNITAIR
          	.: . . ..::::::.:::.:.:.:. ..:.::...: ..:.  .::  ... .. ::  

MG01032.1 	------------------------------------------------------------
NCU03924.1	------------------------------------------------------------
FG08400.1 	------------------------------------------------------------
AN3660.1  	ANTMKFLPNFFARGQLSKIFICFPDPHFKARKHKARIVSETLNAEYAYALRPGGLLYTIT
          	:.: .  ..  : .. :.   . ...  .: . .:   :.: .:. : :  ...   : :

MG01032.1 	------------------------------------------------------------
NCU03924.1	------------------------------------------------------------
FG08400.1 	------------------------------------------------------------
AN3660.1  	DVEEYHYWILRHFGYDVEAEQAQKQGEGQEEVQESVGKREGSAELFERVSEEDIEKDECV
          	. ..         . . .:..:......... ..: .. ..::.  .  :... ..... 

MG01032.1 	----------------------------------
NCU03924.1	----------------------------------
FG08400.1 	----------------------------------
AN3660.1  	RVMKEATEEGKKVARNGGNKYVAVFRRKSNPEWV
          	   ..::..... : .....  :    .:...
```
