# Supplementary material for: Patterns of Intron Gain and Loss in Fungi
Source: PLoS Biol. 2004 Nov 30;2(12):e422. doi: 10.1371/journal.pbio.0020422 (PMC532390; doi:10.1371/journal.pbio.0020422)
Supplement: Table S1 — Also available at http://genes.mit.edu/NielsenEtAl/. (4.3 MB ZIP). [file pbio.0020422.st001.zip › NielsenEtAl/html/1101.html]

AN7161.1.NCU01849.1.MG08431.1.FG01982.1


```
 CLUSTAL W (1.82) Multiple Sequence Alignments - Introns Inserted


Sequence 1: AN7161.1	377 aa
Sequence 2: FG01982.1	378 aa
Sequence 3: NCU01849.1	385 aa
Sequence 4: MG08431.1	347 aa
Alignment Length: 402 aa
Number Identitical Residues: 99 aa
Alignment Score (without introns) 5901


MG08431.1 	MLDRLAINLLLLASSAAVLAHPGADVRTEFLERQEHLNNPARRTLSECHARLEARGHYAA
NCU01849.1	-MHLSSLLLG-AIAATAVTAHPGHDIRAEHAERQAILDLIGRSDYGRCADKLKARGVEAR
FG01982.1 	MPSIKNVLASFALAATIASAHPGHDIAHEAAERRDFLNSVKRSSLAHCATKLKARGVEAR
AN7161.1  	MVYLSTLLTGAVGLATVVFAHPGHDVKAEAAERAAALKSVRARGLSQCATQLQARGVEAA
          	      :       :: . **** *:  *  **   *.       ..*  :*:***  * 

MG08431.1 	ELERRMTKINGLRSKRGLQ------~------------AAPGAVQKRDGGARAG----CV
NCU01849.1	AVARREELAKKMMKKRNLE-----A1RSIYARGTHH--SNATFTLQTPASEIFASTDFCF
FG01982.1 	NVARRSAQVNKARAKRALK----KR~EEESALNKSHNQTEQGFTECTDAAALFASINSCV
AN7161.1  	SVARRDASLQKIRKARGLSGPLLKA~RDTTPLTTSH-ESNLTVDLSTDPSVLFASGGSCV
          	 : **    :     * *...  .   .  .  .   .:          .   .: . *.

MG08431.1 	LDPEVTEGPFW1VQGEVIRPDIREDSNGVVLHLDINVIDVTTCE~PIPDAYVELWG~CNS
NCU01849.1	LSPEVTEGPYY~VAGEYIREDITEDQAGVDLALDLQVYDVETCE~PVPNVYLEIWH1CNS
FG01982.1 	LTPEVTQGPYY1VAGEYVRENVIEEQDGLNIVLDYQVIDVETCD~PVPNVYLEMWH~CNS
AN7161.1  	LAEDVTQGPYY1VSGELIRQNLVEDQPGVPLYLDIQLLDSETCE1PVPDVYLDFWH~CNA
          	*  :**:**:: * ** :* :: *:. *: : ** :: *  **: *:*:.*:::*  **:

MG08431.1 	T0GVYTGVIAKGNGVGLDAPEEINNSALRGVQPTDADGTASFVTIVPGHYVGRTNHLHT1
NCU01849.1	T~GVYSGVSANGNG-NGDAAN-LNATFLRGLQQTDADGIAQFETLFPGHYTGRATHIHV~
FG01982.1 	T~GVYSGVIANGNGDNSDETN-IDKTWLRGIQKTNSDGVAQFESIFPGHYTSRATHIHV~
AN7161.1  	T~GVYSGVVASGNGDSSDETN-LDATFLRGIQQTDDSGVAEFETIFPGHYTGRTTHIHV~
          	* ***:** *.*** . * .: :: : ***:* *: .* *.* ::.****..*:.*:*. 

MG08431.1 	IVH-HGATRLSNDTIQG---GTVSHVGQFYFDDVSIEAVELESPYSTNTQAWTRNADDFL
NCU01849.1	MVH-VDPTVFPNNTIR---STTASHVGQIYFDQDLITEVEAEPVYNTNTQKLTLNSDDML
FG01982.1 	MVH-TNATLLANHTLG--SDNYASHVGQAFFDQDLISQVETLEPYASNTQEITLNEDDGI
AN7161.1  	LSHPADTTANANDTLEGLYTTTSSHVGQIFFDQDLISLVEATDVYSSNTQELTLNADDSI
          	: *. ..*  .*.*: .      ***** :**:  *  **    * :***  * * ** :

MG08431.1 	LRQGQA-GGDNPFIELTLLGGSMADGLHGVIDVGVDPSAKQEPKPVNFWTADGGKPVEGS
NCU01849.1	LQQGSQ--VGDPIVNYVYLGSTVSEGLLGWIGFGINTTLSKDVSAAATFYESGGVANSNG
FG01982.1 	LSEEISTDGVDPFMEYTLLGDSVSDGLFAWLAFGINTTVSNSVTPAAYYYKEGGVANGNS
AN7161.1  	LLQELN-DDIDPFVEYVLLGEDVTDGIFAWINVVIDSSQSTSVTPAAYLTEDGGVENENS
          	* :    .  :*::: . **  :::*: . : . ::.: . . ...     .**    ..

MG08431.1 	------------PWAGYPWTVRLMALLRRWGL----------------
NCU01849.1	GFPGGPGGGAGGPPSGFPSGGFPTGVRPTNASSSAAATAAPACNAKKN
FG01982.1 	--GGAPGGGAGGPPSGMPSGSRPGSTPSATPDAE--------------
AN7161.1  	--GGF-GGGSDGPGGSAPGGSAPTGAPPS-------------------
          	   .  ...:..* .. *      .
```
