# Supplementary material for: Patterns of Intron Gain and Loss in Fungi
Source: PLoS Biol. 2004 Nov 30;2(12):e422. doi: 10.1371/journal.pbio.0020422 (PMC532390; doi:10.1371/journal.pbio.0020422)
Supplement: Table S1 — Also available at http://genes.mit.edu/NielsenEtAl/. (4.3 MB ZIP). [file pbio.0020422.st001.zip › NielsenEtAl/html/1102.html]

AN0753.1.NCU05304.1.MG06082.1.FG09747.1


```
 CLUSTAL W (1.82) Multiple Sequence Alignments - Introns Inserted


Sequence 1: MG06082.1	456 aa
Sequence 2: FG09747.1	472 aa
Sequence 3: NCU05304.1	485 aa
Sequence 4: AN0753.1	484 aa
Alignment Length: 498 aa
Number Identitical Residues: 146 aa
Alignment Score (without introns) 9183


MG06082.1 	MAEAATNTAAPA------VRPTKPDEAAYKEKLAQAEKEHSDVMTKY0NAIKAKIDIAQP
NCU05304.1	MAESQSPAAAPAAT----TKPHKPDQDAFNENLAKAEKEYQEALKKY0NEIKAKVELAAP
FG09747.1 	MAETATSPAPAAENKQAHVRPTKPDEEVFKKELAKAEKEHKASMDRL0AAVKAKIDIAMP
AN0753.1  	MAAAVKSEGASEPK----VRPTKPDEETFKANLAQAEKEHAAVQEKL0NQIKAKIEAAKP
          	** : .  ...  .    .:* ***: .:: :**:****:     :    :***:: * *

MG06082.1 	NKNKDAPNPTQKRRQELIAQANEIRQKQAGGKNARQTKQDQIKRLDEQLRSRIAEQKNAR
NCU05304.1	SKNKDQPSPTQKKRQELISQLNEIRQQQAGGKNARTSKLDQIKRLDEQLRSRIAEQKTAR
FG09747.1 	NKNKDQPNPTQQRRQELIAQANEIRQKQAGGKNARTGKLDQIKRLDEQVRSRIAEQKTAK
AN0753.1  	N-NQD--SPAAKRQQELRAELSSIRQKQQGFKASRTSTQEKINALDATLKARIAEQNNSR
          	. *:*  .*: :::*** :: ..***:* * * :*  . ::*: **  :::*****:.::

MG06082.1 	GKVNFKSVEEVDREISRLEKQIEGGTMR----------------------------I0EI
NCU05304.1	SKVNFKSTEELDREIERLEKEVNGGMMKLVDEKKALAEISNLRKQRKSFAGFDDAQK~QI
FG09747.1 	AKVPYKSLEDVDRQIATLDKQVNSGTMKLVDEKKALSDISSLRKMRKNFGQFDDSQK~QI
AN0753.1  	TRMSFKNVEELDREIARLEKQVDSGTLRLVDEKKILADISSLRKQRKNFASLDDAQK~VI
          	 :: :*. *::**:*  *:*:::.* ::  ....  :. :.  .  .. .  ..:.   *

MG06082.1 	DDLRNKIKEVKDSMDNPEQKALSEQYNKIQAEIDVIKAEQDEAYKGLSSLRDERTKLQNE
NCU05304.1	DDLKAKIKEIKDSLEDPEAKALSEKYNKLQAELDAIKAEQDEAYKNLSSLRDERTKLQQL
FG09747.1 	DDLRAKIKEIKDSMDDPEQRALSDQYNKIQAEIDTIKAEQDEAYKGISSLRDERTKLQAE
AN0753.1  	NDLKTQITTLKKTLDNPEAKALSDKYTEIQKELDAIKAEQDSAFKNLNALRDERTKLHAE
          	:**: :*. :*.::::** :***::*.::* *:*.******.*:*.:.:********:  

MG06082.1 	QREKFEAIRKIKDEYYAQKKAYSNFEWEAKQKARERRKAEDAKRAQEFKMERAKKMLAEA
NCU05304.1	QSEKYQAIKKLKDEYYGAKKEFAKWEREQREKARERQQAERERIAKERRMERAQKMLAEA
FG09747.1 	QQEKYTAIRKLKDDYYGQKKAFQAYDREAREARREKQRAEQERYHQERKKAEAERRLGDA
AN0753.1  	QQAKWTAIREVKDNYYKARKAYKEYEDEAWRIRREKQKAQREAFEREKKRKIADKKLEEA
          	*  *: **:::**:**  :* :  :: *  .  **:::*:     :* :   *.: * :*

MG06082.1 	SDPAYLEEIRRANSLIRFFDPSHQGETRAPLLAS---SGLSAEATRKVDDSGLKGTRLMS
NCU05304.1	SDPAYLEEIRRANSLLKYFDPSHEVAEKAPLLAD---KGLGAQALRKVDDSGLKGMKLVR
FG09747.1 	SDPAYLDEIRRANSLLQFLDPNHKVE-KGPLMAD---TGLGAQAQRSVDESGLKGTKLLR
AN0753.1  	SRPAYTDEILVAQGLIRHFNPSYDFAALGLSDKKDQASGFRAEVGRTVDDSGMKGMKVLK
          	* *** :**  *:.*::.::*.:.    .    ...:.*: *:. *.**:**:** ::: 

MG06082.1 	KKDRDDEYAPAVKKGKKGKKT----NAAPDAAASGKFSCPPSVMEDCAFMGIEPPMSAAD
NCU05304.1	KEERDDDYLPAVKKGKKSKKP----TGAAPVATGKTFSLPPSVIEDCSFVGVEPPMAATD
FG09747.1 	KEDREEEYAPAVKKGKKGKKA----GAGP---SSNKFSVPPAVVEDCAAMGIDPPMSAAD
AN0753.1  	KEEDDYFVGTGGKKSKKGKKGSANGSPAPSNLAETKFNMNVGIIEDFAKVKIDPPMNQSD
          	*:: :    .. **.**.** ::..  ..   :  .*.   .::** : : ::***  :*

MG06082.1 	VPAVLEKAKAKLEHWKSDQAAQTQK0NIDKAKKEIEKIEAEEAAGHDANGDSKEDKAVAG
NCU05304.1	IPAAVEKIKAKLEQWKADQPEQTRK0NIEKAKKEIERLEAEEAG--EASGSATPKKAVEE
FG09747.1 	IPTVAEKVRAKLDFWKNDQEAQTQR0NIEKAKKEIAELEAAEAN-----GDK-----IEQ
AN0753.1  	VPAVVEKLAAKITEWKKDQAAKTQE0NINKAKEEIARLDAEESTATETNGKASEKDATAE
          	:*:. **  **:  ** **  :*:. **:***:** .::* *: . .:.*. . ..:   

MG06082.1 	VTSDLKETAIEDKKDEETKA-
NCU05304.1	VTEGVKNATIEEKTEAVEASA
FG09747.1 	ATSDLKETSIADKQES-----
AN0753.1  	VADDLNKASLEEKA-------
          	.:..:::::: :*
```
