# Supplementary material for: Patterns of Intron Gain and Loss in Fungi
Source: PLoS Biol. 2004 Nov 30;2(12):e422. doi: 10.1371/journal.pbio.0020422 (PMC532390; doi:10.1371/journal.pbio.0020422)
Supplement: Table S1 — Also available at http://genes.mit.edu/NielsenEtAl/. (4.3 MB ZIP). [file pbio.0020422.st001.zip › NielsenEtAl/html/1103.html]

AN6610.1.NCU03718.1.MG00727.1.FG07154.1


```
 CLUSTAL W (1.82) Multiple Sequence Alignments - Introns Inserted


Sequence 1: MG00727.1	294 aa
Sequence 2: FG07154.1	236 aa
Sequence 3: NCU03718.1	282 aa
Sequence 4: AN6610.1	238 aa
Alignment Length: 303 aa
Number Identitical Residues: 102 aa
Alignment Score (without introns) 5273


MG00727.1 	MADDTAPLASLSLTHVFY0DPTDPVSYVCAWLALAPQALCVVYVTLIWSSREAEVALLFA
NCU03718.1	MADNVTPLASLSLTHVYY0NPDDPISLLCAWLALVPQALCVVYATLIWSTREAEVILMFA
FG07154.1 	MADDSAPLASLSVTHVYY0DPEDHLSLVCAYLALLPQALCVVYATLVLFTREVEVGLMFL
AN6610.1  	MEEDEQPLASLSLTHVHY0NPDDPLSLVSAWLALVPQALCVVYVTLVWASREVEVGLMFA
          	* ::  ******:***.* :* * :* :.*:*** ********.**:  :**.** *:* 

MG00727.1 	GQLACEAINFALKRLIKEERPRR~IH---GKGYGMPSSHAQFLAFWALSLALFLLVRHRP
NCU03718.1	GQLACEAANFALKRLIKEERPAR~IHSTGGKGYGMPSSHAQFVSFWAVALGLFLLARHTP
FG07154.1 	GQLACEALNFALKRLIKEERPRR~IH---GKGYGMPSSHAQFVAFWSVSLALFLLVRHKP
AN6610.1  	GQLVCEALNFALKRIIKEERPKQ1MF---GKGYGMPSSHAQFVAFFAVYLTLFLIFRHAP
          	***.*** ******:****** : :.   *************::*::: * ***: ** *

MG00727.1 	KAHKIKKQGAGGGGGSDKKET------VKATAAPAWDLWQLQELYGVDRYPHRPWSMFER
NCU03718.1	REQQQQQQQKQKQRERKKQVTNVKTTTTNGSGNGSLFKTLTDSATDLERYAHEPWSFAHR
FG07154.1 	--PRVLKSRADSG-------------------------------------VHRPWSVIER
AN6610.1  	--------------------------------------------------NSANQSILFR
          	                                                       *.  *

MG00727.1 	AAVSAMGFALAGVVAWSRVYLGYHTPKQVFVGLSAGCVSAIGWFVATEVARQTGLLGWVL
NCU03718.1	FVASLGALVLAGAVAWSRTYLGYHTEKQVLVGCGAGTLCAVAWFVVTHVVRQSGLLGQIL
FG07154.1 	MAVSMAGMAIAAATAWSRVYLNYHTPKQVVVGCAAGAVSAIGWFIIVAIVRQTGLLGWAL
AN6610.1  	MVASLGITLGASAVAVSRIYLTYHTVRQVLAGCAVGAVFALFWFTFTGLLRSYGWIDWAL
          	 ..*      *...* ** ** *** :**..* ..* : *: **  . : *. * :.  *

MG00727.1 	SLPAARWFRLRDLV~I~EEDICQAGWEKWEQRRVALAVAAGKSDTADT~SAT0SWTLNNL
NCU03718.1	DFPVVRWFRVRDLV~V~EEDLPQAGWEKWEEQRVARREVEERKKAL--~---~-------
FG07154.1 	ETPLVRAFRIRDLV~V~EEDMCQAGWEKWEDRRVASRTTKNR------~---~-------
AN6610.1  	EHSIVRLLRIRDLV0Q0GLHKLR-GYKYTYVLDMTRCCVFDKNNSELQ2CST~RHHAQ--
          	. . .* :*:****     .  : *::      ::   .  :..:    .::     .  

MG00727.1 	DKTLASGSA
NCU03718.1	---------
FG07154.1 	---------
AN6610.1  	---------
          	
```
