# Supplementary material for: Patterns of Intron Gain and Loss in Fungi
Source: PLoS Biol. 2004 Nov 30;2(12):e422. doi: 10.1371/journal.pbio.0020422 (PMC532390; doi:10.1371/journal.pbio.0020422)
Supplement: Table S1 — Also available at http://genes.mit.edu/NielsenEtAl/. (4.3 MB ZIP). [file pbio.0020422.st001.zip › NielsenEtAl/html/1104.html]

AN4923.1.NCU03922.1.MG01026.1.FG09266.1


```
 CLUSTAL W (1.82) Multiple Sequence Alignments - Introns Inserted


Sequence 1: NCU03922.1	454 aa
Sequence 2: MG01026.1	456 aa
Sequence 3: FG09266.1	456 aa
Sequence 4: AN4923.1	459 aa
Alignment Length: 460 aa
Number Identitical Residues: 280 aa
Alignment Score (without introns) 12900


MG01026.1 	MASRPQNIGIKAIEIYFPSQ0YVEQSELEKFDGVSSGKYTIGLGQTKMAFCDDRED1IYS
NCU03922.1	MATRPQNIGIKAIEIYFPSQ0YVEQSELEKFDGVSTGKYTIGLGQTKMAFCDDRED1IYS
FG09266.1 	MSSRPQNIGIKAIELYFPSQ0YVDQVELEKFDGVSAGKYTIGLGQTKMSFCDDRED1IYS
AN4923.1  	-MSRPQNIGIKAIEVYFPSQ~CLDQTELEKHDGVSEGKYTIGLGQSKMSFCDDRED1IYS
          	  :***********:*****  ::* ****.**** *********:**:******* ***

MG01026.1 	LALTATSNLLKKYAIDTNSVGRLEVGTETLLDKSKSVKSVLMQLFG--DNTNIEGVDTVN
NCU03922.1	LALTAVSRLLKNYEIDTNTIGRLEVGTETLLDKSKSVKSVLMQLFG--ENTNIEGVDTIN
FG09266.1 	FALTATSKLLKNYNIDPNSIGFLEVGTETLLDKSKSVKSVLMQLFG--DNTNIEGVDTIN
AN4923.1  	IALTTLSSLMKKYNIDPNSVGRLEVGTETLLDKSKSVKSVLMQLFAPHGNTNIEGVDTVN
          	:***: * *:*:* **.*::* ***********************..  *********:*

MG01026.1 	ACYGGTNALINSVNWIEGSGWDGRDAIVVAGDIALYAKGNARPTGGAGCVAMLVGPDAPI
NCU03922.1	ACYGGTNAFFNSVNWIESSAWDGRDAIVVAGDIALYAKGNARPTGGAGCVAMLVGPNAPI
FG09266.1 	ACYGGTNAVFNAINWVESSAWDGRDAIVVAGDIALYAKGNARPTGGAGAVALLIGPNAPI
AN4923.1  	ACYGGTNALFNSINWVESSAWDGRDAVVVCGDIALYAKGAARPTGGAGCVAMLIGPDAPI
          	********.:*::**:*.*.******:**.********* ********.**:*:**:***

MG01026.1 	VVEPGLRGSFMQHAYDFYKPDLASEYPYVDGHFSLTCYTKALDGAYRAYNKREAQLQNGH
NCU03922.1	AVEPGLRGSYMAHAYDFYKPDLTSEYPYVDGHYSVNCYTEALDGAYRAYNQREKLLTNG-
FG09266.1 	VAEPGLRGTYMQHAYDFYKPDLTSEYPYVDGHYSVNCYSKALDAAYRAYCKREAKQANGT
AN4923.1  	IFEPGLRASYLTHAYDFYKPDLTSEYPVVDGHFSLRCYTEAVDACYKAYGAREKTLKEKT
          	  *****.::: **********:**** ****:*: **::*:*..*:**  **    :  

MG01026.1 	ANGNGAADS--SKTPLDRFDYIAFHSPTCKLVQKSYARLLYHDYLANPDHPAFAEVPGEL
NCU03922.1	VNG-HSEDS--TKTPLDRFDYLAFHAPTCKLVQKSYARLLYHDYLANPESPVFADVPPEV
FG09266.1 	NGVTNGDAS--TKTGLDRFDYMAFHSPTCKLVQKSYARLLYHDYLANADSPVFAEVAPEL
AN4923.1  	QNGTNGVTHDESKTALDRFDYVLFHAPTCKLVQKSYARMLYNDYLANPTHPSFAEVAPEL
          	 . . .   ..:** ******: **:************:**:*****.  * **:*. *:

MG01026.1 	RDMDYEKSLTDKGVEKAFMALTKKRFQERVNPGIQVATLCGNMYCASVWGGLSSLIGHVD
NCU03922.1	RDMDYKKSLTDKVVEKTFMTLTKKRFQERVNPAIQVPTLCGNMYCGSVWGGLASIIGHVD
FG09266.1 	RDMDYEKSLTDKVVEKTFMTLTKKRFQERVNPAIQVATNCGNMYCGSVWSGLASLISVVD
AN4923.1  	RDLDYETSLTDKNVEKTFMGLTKKRFAERVRPGLDVATLCGNMYTATVYAGLCSLLSNVT
          	**:**:.***** ***:** ****** ***.*.::*.* ***** .:*:.**.*::. * 

MG01026.1 	SATLQGKRIGLFSYGSGLAASFLSFRVNGSTETISKALDIPNRLEARRAVQPQTYDD0MC
NCU03922.1	SAQLEGKRIGLFSYGSGLAASFCSFRVTGSTEKLAKTLNLPARLAARRAVPPESYDA0MC
FG09266.1 	NKDLEGKRIGLFSYGSGLAASFLSFRINGSVDKISDVLNIPSRLESRRAVPPETYDQ0MC
AN4923.1  	FDPSQPKRLGFFSYGSGLASSLFSAKIVGDVSYIVEKLNLHKRLESRTVLSPTDYEA~MC
          	    : **:*:********:*: * :: *... : . *::  ** :* .: *  *:  **

MG01026.1 	EMRKKAHLQKNFTPTGDVSTIVNGTYYLENVDDMFKRTYSVKA
NCU03922.1	DLRKQAHLQKNYTPKGEVSTLEPGTYYLENVDDMFKRTYSIKA
FG09266.1 	DLRKQAHLQKDYTPKGDPSTILPGTYYLTKVDDMFKREYAIKE
AN4923.1  	ELREHAHLAKDFKPSGNPDTLFPGTYYLTEIDGMFRRKYETKA
          	::*::*** *::.*.*: .*:  ***** ::*.**:* *  *
```
