# Supplementary material for: Patterns of Intron Gain and Loss in Fungi
Source: PLoS Biol. 2004 Nov 30;2(12):e422. doi: 10.1371/journal.pbio.0020422 (PMC532390; doi:10.1371/journal.pbio.0020422)
Supplement: Table S1 — Also available at http://genes.mit.edu/NielsenEtAl/. (4.3 MB ZIP). [file pbio.0020422.st001.zip › NielsenEtAl/html/1105.html]

AN1638.1.NCU09228.1.MG10606.1.FG08340.1


```
 CLUSTAL W (1.82) Multiple Sequence Alignments - Introns Inserted


Sequence 1: NCU09228.1	904 aa
Sequence 2: MG10606.1	883 aa
Sequence 3: AN1638.1	883 aa
Sequence 4: FG08340.1	1284 aa
Alignment Length: 1311 aa
Number Identitical Residues: 425 aa
Alignment Score (without introns) 22441


MG10606.1 	------------------------------------------------------------
NCU09228.1	------------------------------------------------------------
FG08340.1 	MPSAAIETLPGHILGGDAQSRPRLDQPLTYTGSLDNYSQLDVTPVIGREFNGLQIRDLLK
AN1638.1  	------------------------------------------------------------
          	                                                            

MG10606.1 	------------------------------------------------------------
NCU09228.1	------------------------------------------------------------
FG08340.1 	WDDVHIRDLAVTISQRGVVFLKDQDVTPHEMKDFMLRLTDLAGCPTSSGLHVHPLTEEGS
AN1638.1  	------------------------------------------------------------
          	                                                            

MG10606.1 	---------------------------------------~--------------------
NCU09228.1	---------------------------------------~--------------------
FG08340.1 	ELGDQISVISSEKQKKGGGLTHQLSDVSRFASAGWHSDI2TFEKVPSDYAMLKIHTLPAT
AN1638.1  	---------------------------------------~--------------------
          	                                                            

MG10606.1 	----~-------------------------------------------------------
NCU09228.1	----~-------------------------------------------------------
FG08340.1 	GGDT0LWASGYEVYDRLSDPMKKFLEGLTATHDATFFHDEARRLGNPIRKGIRGSPLNQG
AN1638.1  	----~-------------------------------------------------------
          	                                                            

MG10606.1 	------------------------------------------------------------
NCU09228.1	------------------------------------------------------------
FG08340.1 	EQLTSVHPLIRTNPVTGWKSVFVNKGFTKRINGLSKDESDTLLAYLFNLVTQNHDAQVRY
AN1638.1  	------------------------------------------------------------
          	                                                            

MG10606.1 	---------------------------------------------~--------------
NCU09228.1	---------------------------------------------~--------------
FG08340.1 	RWSKNDCAIWDNRSTFHCATYDYEAARAGDRVCSLGEAPYLDTNT1STTTSTRPLHTSTS
AN1638.1  	---------------------------------------------~--------------
          	                                                            

MG10606.1 	---------------------------------------MCKLHDIEMGGGVGANNAT--
NCU09228.1	---------------------------------------MCRTQ--AQADVASGVNIQ--
FG08340.1 	PYLLQSNLATTSTRSIRSRPSVLEPKTTPLISRHQQRNCSCRRAMLRNGNDVTSASLDIR
AN1638.1  	---------------------------------------MCGTR---RAEAAGSTNVP--
          	                                        *       .  . . .    

MG10606.1 	-GRELLPTNVVPKHYDLTLEPDLEKFTFNGSVVVH~LDVAEDSKSISLHTLEIDVKNAKV
NCU09228.1	-GRELLPTNVIPKHYHITLEPDFQKLTFDGTVVID~LDVEEDSKSISLHTLEIDIHNAKI
FG08340.1 	RGRQVLPKNVKPLHYDLTLEPNFETFKYEGTVVID~FDVVEDSTSIALNTVDLEIHDTLV
AN1638.1  	-GREVLPTNVKPTHYDLTLEPNFETFKYDGTVIID2LQVAEDTTSISLNSTEIDIHTATV
          	 **::**.** * **.:****:::.:.::*:*::. ::* **:.**:*:: ::::: : :

MG10606.1 	TSGGQTIT~S-PKISYNEDTQVTKIDFDETISKGSKAELTIDFTGTLNDKMAGFYRAVYK
NCU09228.1	TSGGQTVS2SSPKVSYNETTQVSTFDFDNAVNKGAKAQLEIQFTGQLNDKMAGFYRSTYI
FG08340.1 	EANGATIS~SSPTLDYDKDSQTTTITFDKTIPAGQKARLTQRFTGTLNDDMAGFYRSSYK
AN1638.1  	SAQGSVVS~SSPEILLNKDKQEATIKFSETISAGSSAQLKLTFTGTLNDNMAGFYRSSYK
          	 : * .:: *:* :  :: .* :.: *.:::  * .*.*   *** ***.******: * 

MG10606.1 	RDDGSEGVLAVSQMEPTDARRAFPCFDEPSLKATFAVTLIADKKLTCLSNMDVASESEVQ
NCU09228.1	NPDGTQGLLAVSQMEPTDARRAFPCFDEPSLKAEFTVTLIADKKLTCLSNMDVASESEVK
FG08340.1 	DEQGNTKYIATTQFEATDARRAFPCLDEPALKATFTVTLIADKDLVCLGNMDVASEKEVD
AN1638.1  	TPQGETKYIASTQMEPTDARRAFPCFDEPALKAKFTVSLIADKSMTCLGNMDVASEQELE
          	  :*    :* :*:*.*********:***:*** *:*:*****.:.**.*******.*:.

MG10606.1 	SALTGTTKKAVKFHNSPLMSTYLLAFIVGELNYIETKDFRVPVRVYAPPGLNIEHGRFSL
NCU09228.1	-----DGKKAVTFNKSPLMSTYLVAFVVGELNYIETNDFRVPVRVYAPPGQNIEHGRFSL
FG08340.1 	SKVTGKKSKVITYNKTPIMSTYLLAFIIGDLKHYETNNFRVPIRVWCTPDQNLDHAVFSA
AN1638.1  	-----GGKKIVKFNTSPVMSTYLVAFIVGHLNYIETKNFRVPIRVYATPDQDIEHGRFSL
          	       .* :.::.:*:*****:**::*.*:: **::****:**:..*. :::*. ** 

MG10606.1 	NLAAKTLAFYEKVFGIDFPLPKMDQVAIPDFAQGAMENWGLVTYRVVDLLLDEKVSGAAT
NCU09228.1	DLAAKTLAFYEKVFGIEFPLPKMDQIAIPDFAQGAMENWGLVTYRVVDLLLDEKVSGAAT
FG08340.1 	ELGARTLEFYEEQFGSKYPLPKMDMVAVPDFAAGAMENWGLITYRVVDLLLDEKTSSAVT
AN1638.1  	ELAAKTLAFYEKAFDSEFPLPKMDMVAVPDFSAGAMENWGLITYRIVDVLLDEKTSSASR
          	:*.*:** ***: *. .:****** :*:***: ********:***:**:*****.*.*  

MG10606.1 	KERVAEVVQHELAHQWFGNLVTMDWWDGLWLNEGFATWASWYSCNVFFPEWKVWESYVTD
NCU09228.1	KERVAEVVQHELAHQWFGNLVTMDWWDGLWLNEGFATWASWYSCNIFYPEWKVWETYVTD
FG08340.1 	KKRVAEVVQHELAHQWFGNLVTMDFWDGLWLKEGFATWMSWYSSNAFYPEWRIWEGYVTE
AN1638.1  	KERIAETVQHELAHQWFGNLVTMDFWDGLWLNEGFATWMSWYSCNSFYPEWKVWQTYVID
          	*:*:**.*****************:******:****** ****.* *:***::*: ** :

MG10606.1 	TLQSALSLDSLRSSHPIEVPVKRADEINQIFDSISYAKGSCVLRMISTYLGEDVFLEGVR
NCU09228.1	NLQRALALDSLRSSHPIEVPVKRADEINQIFDAISYSKGSCVLRMISTYLGEDVFLEGVR
FG08340.1 	DLRSALGLDSLRSSHPIEVPVKRADEVNQIFDAISYEKGSCVLRMISKYLGEDVFLKGIR
AN1638.1  	NLQSALSLDSLRSSHPIEVPVKRADEINQIFDAISYSKGSSVLRMISKYLGEDIFLQGVR
          	 *: **.*******************:*****:*** ***.******.*****:**:*:*

MG10606.1 	QYLKKHAYGNTQTDDLWDSLAKASGKPVHEVMTAWTKNVGYPVITVTENEKDSSIHLKQN
NCU09228.1	RYLKKHAYGNTQTGDLWAALGDASGKSVEEVMDVWTKHVGYPVVTVTEKD-EKTIHVKQN
FG08340.1 	IYLDRHAYANTETTDLWAALSEASGKDVERVADIWTKKVGYPVVAITEDESKGTIHVKQN
AN1638.1  	NYIKKHAYGNTQTGDLWSALANASGKPVEEVMDIWTKNVGFPVVTVSENPTSSSIKVKQN
          	 *:.:***.**:* *** :*..**** *..*   ***:**:**::::*. .. :*::***

MG10606.1 	RFLRTGDTKPEEDQVLYPVLLGLRTKDGIDESRTLTARENDFKLPDVDFFKLNANHTSLF
NCU09228.1	RFLRTGDVKPEEDKVIFPVFLGLRSKDGIDESLTLDKREDSFEVPSTEFFKLNANHTGLY
FG08340.1 	RFLRTADVKPEEDEVLYPVFLNLRTKEGIQEDLALNVREADFKVPDFDFYKVNSGHSGIY
AN1638.1  	RFLRTGDVRPEEDTTIFPVMLGLRTKQGVDEDTLLSEREGEFKLPDLDFYKLNADHSAIY
          	*****.*.:**** .::**:*.**:*:*::*.  *  ** .*::*. :*:*:*:.*:.::

MG10606.1 	RTAYSPERLEKLGNAARNGLLSVEDRAGMLADAGALAVSGYQKTSGVLNLLKGYDSESQF
NCU09228.1	RTSYTPERLEKLGEAARQGLLSVEDRAGMIADAGALASSGYQKTSGVLTLLKRFDSEKEF
FG08340.1 	RTSYTSERLQKLGQNVKAGLLGVEDRAGMIADAGALAAAGYQKTSGLLSLLQGFDSEDEF
AN1638.1  	RTSYTPERLTKLGEAAKAGLLTVEDRAGMIADAGALAASGYQSTSGLLSLLAGFDSEPEF
          	**:*:.*** ***: .: *** *******:******* :***.***:*.**  :*** :*

MG10606.1 	VVWTEIIGRLAAVHSAWIFEDKAIKDSLEAFQRDLISSRAHKMGWAFSESDGHIEQQFKA
NCU09228.1	IVWSEIISRVAAVQAAWIFEDKAVRDGLEAFQRELVSPRAHEMGWEFSESDGHIEQQFKA
FG08340.1 	IVWDEITLRVASLRDAWIFEEDDVNKALKAFQRDLVSKKANEIGWNISSSDDFTAQRFKA
AN1638.1  	VVWNEILTRVGALRAAWVFEDAQTKDALEGFQRALVSDKAHTLGWQFSENDGHIIQQFKA
          	:** **  *:.::: **:**:   ...*:.*** *:* :*: :** :*..*..  *:***

MG10606.1 	LLFGSAGIAGDKDIVAAAKDMFKKFMDGDKSAIHPNIRGSVFAMALKYGGDDE0YNRILD
NCU09228.1	MLFGNAGLCGDEKIIAAAKEMFKKFIAGDKSAIHPNIRGSVFSIALKYGGTEE0YDAVLN
FG08340.1 	LMFGKAAIVEDEAAKKAAFELFEKFINGDREAVQPNLRSSVFGVVLTYGGEAE~YNAVLK
AN1638.1  	LLFSAAGNAGDKTVVQAAQDMFQRFAAGDISAIHPNIRGSVFSIVLKNGGKKE~YDVVYD
          	::*. *.   *:    ** ::*::*  ** .*::**:*.***.:.*. **  * *: : .

MG10606.1 	FYRTSTNSDERNTALRSLGRSNKPEHIKQTLDLMFSGEVKDQDIYMPAAGLRSHSEGIEA
NCU09228.1	FYRTSTNSDERNTALRCLGRARSPELIKRTLDLLFSGEIKDQDVYMPTAGLRSHPEGIEA
FG08340.1 	EYETAKQSSERNTALRSLGFAKDPALMKRTFAYTLSDNVKTQDIYLPLAGLRAHKEGIVA
AN1638.1  	RFRNAPTSDEKTTALRCLGAAEDPELIQRTLGLALGDEVKNQDIYMPLGGLRNHAAGIDA
          	 :..:  *.*:.****.** :..*  :::*:   :..::* **:*:* .*** *  ** *

MG10606.1 	LSKWIMDNWDALYIKLPPALSMLGSMVAICTSSLTKPEQLKQVEEFFANKDNK~GYEMSL
NCU09228.1	LFNWMTENWDELVKRFPPQLSMLGTLVTIFTSSFTKREQLAKVEKFFEGKNTN~GFEMSL
FG08340.1 	LWGWVKENWDVLTKRLPPGMSLLGDMVAISTSSFTHADQIDDVKSFFEQKGSK~GFELEL
AN1638.1  	RWAWMKDNWDTLYQRLPPGLGMLGTVVQICTASFCTEEQLKGVQNFFANKDTK0GYDRAI
          	   *: :*** *  ::** :.:** :* * *:*:   :*:  *:.**  *..: *::  :

MG10606.1 	AQSLDAIRSKIAWLERDRSDVAAWVKEQGY-~-----------~----------------
NCU09228.1	AQSLDAIRSKVAWVERDGEDVAKWVKDNKYS1SSENGTPSATR2SDILQMEVADAASSRI
FG08340.1 	AQSLDSMKARQNWLARDKEDVKQWLIQNKYL~-----------~----------------
AN1638.1  	EQSLDAIRAKISWVQRDRADVGSWLKSKGYL~PGNGKL-----~----------------
          	 ****:::::  *: **  **  *: .: *  ....                        

NCU09228.1	
MG10606.1 	
AN1638.1  	
FG08340.1 	
          	
```
