# Supplementary material for: Patterns of Intron Gain and Loss in Fungi
Source: PLoS Biol. 2004 Nov 30;2(12):e422. doi: 10.1371/journal.pbio.0020422 (PMC532390; doi:10.1371/journal.pbio.0020422)
Supplement: Table S1 — Also available at http://genes.mit.edu/NielsenEtAl/. (4.3 MB ZIP). [file pbio.0020422.st001.zip › NielsenEtAl/html/1106.html]

AN0554.1.NCU03415.1.MG03900.1.FG00979.1


```
 CLUSTAL W (1.82) Multiple Sequence Alignments - Introns Inserted


Sequence 1: MG03900.1	496 aa
Sequence 2: FG00979.1	492 aa
Sequence 3: NCU03415.1	494 aa
Sequence 4: AN0554.1	497 aa
Alignment Length: 499 aa
Number Identitical Residues: 292 aa
Alignment Score (without introns) 14240


MG03900.1 	--MSLVTELKTPVTGAYQQPTGV2FINNEWVEGVDKKTFETINPSTEEVICSVSEATEKD
NCU03415.1	MSSNVFVELKTPVTGTYKQPTGL2FINNEFVEGVDKKTFEVINPATEEVICSVHEATEKD
FG00979.1 	--MALTVELSTPVTGTYQQPIGL2FIDGKWVEGVDKGKFEVINPSTEEVITSVCEGTEKD
AN0554.1  	-MSDLFTTIETPVI-KYEQPLGL2FINNEFVKGVEGKTFQVINPSNEKVITSVHEATEKD
          	    : . :.***   *:** *: **:.::*:**:  .*:.***:.*:** ** *.****

MG03900.1 	VDIAVKAARKAFEGEWKQTAPGQRSKLLTNLAELVEKNLDLLAAVESLDNGKSLAMAKGD
NCU03415.1	VDIAVAAARKAFEGVWRDVTPQQRGIYLLKLADLLEKNLDLLAAVESLDNGKSITMARGD
FG00979.1 	IDLAVAAARKAFDGEWKNTAPQTRGNLLLKLADLAEKNLDLLAAVESLDNGKSITNARGD
AN0554.1  	VDVAVAAARAAFEGPWRQVTPSERGILINKLADLMERDIDTLAAIESLDNGKAFTMAKVD
          	:*:** *** **:* *::.:*  *.  : :**:* *:::* ***:*******::: *: *

MG03900.1 	VGAVAGCLRYYGGWADKIEGKTIDIAPDMFHYTRSEP0IGVCGQIIPWNFPLLMLAWKLG
NCU03415.1	VGAVVGTIRYYGGWADKIEGKTIDISPDSFHYTRQEP~LGVCGQIIPWNFPLLMLAWKVG
FG00979.1 	VGAVVGCLRYYGGWADKIEGKTIDIAPDMFHYTRSEP0IGVCGQIIPWNFPLLMLAWKIG
AN0554.1  	LANSIGCLRYYAGWADKIHGQTIDTNPETLTYTRHEP~VGVCGQIIPWNFPLLMWSWKIG
          	:.   * :***.******.*:***  *: : *** ** :*************** :**:*

MG03900.1 	PALATGNTIVLKTAEQTPLSALVFANLIKEAGFPAGVVNIISGFGKVAGAAISAHMDIDK
NCU03415.1	PALATGNTIVMKTAEQTPLSALVFAQFVKEAGFPPGVLNIISGFGRIAGAAMASHMDIDK
FG00979.1 	PALATGNTVVMKTAEQTPLSALVFTQFIEQAGFPAGVFNLVSGYGKTAGAALSSHMDVDK
AN0554.1  	PAVAAGNTVVLKTAEQTPLSALYAAKLIKEAGFPAGVINVISGFGRTAGAAISSHMDIDK
          	**:*:***:*:***********  ::::::****.**.*::**:*: ****:::***:**

MG03900.1 	VAFTGSTVVGRTIMKAAASSNLKKVTLELGGKSPNIIFNDADIEAAVSWVNFGIYYNHGQ
NCU03415.1	VAFTGSTMVGRQIMKAAAESNLKKVTLELGGKSPNIIFNDADIDQAIDWVNFGIYFNHGQ
FG00979.1 	IAFTGSTVIGRQIMKAAASSNLKKVTLELGGKSPNIVFEDADIEEAINWVNFGIYYNHGQ
AN0554.1  	VAFTGSTLVGRTILQAAAKSNLKKVTLELGGKSPNIVFDDADIDNAISWANFGIFFNHGQ
          	:******::** *::***.*****************:*:****: *:.*.****::****

MG03900.1 	CCCAGSRIYVQEGVYDKFVAAFKERAEKNKVGDPFKEDTFQGPQVSELQFNRIMEYIKSG
NCU03415.1	TCCAGSRVYVQEGIYDKFVAAFKQRAQQNKVGDPFHDETFQGPQVSQLQYDRIMGYIKAG
FG00979.1 	CCCAGTRIFVQESIYDKFLAAFKKRAEENKVGDPFNEETFQGPQVSQLQYDRIMGYIKAG
AN0554.1  	CCCAGSRILVQEGIYDKFVARFKERAQKNKVGNPFEQDTFQGPQVSQLQFDRIMEYINHG
          	 ****:*: ***.:****:* **:**::****:**.::********:**::*** **: *

MG03900.1 	KEEGATVETGGERHGDKGYFIQPTIFSNVRPEMKIMKEEIFGPVVAMAKFKTEEEVIALA
NCU03415.1	KEEGATVETGGERHGDKGYFIQPTIFTNVRHDMKIMKEEIFGPVCAVAKFSTEEEVIKLG
FG00979.1 	KDEGATVEIGGERLGDKGYFIKPTIFSNVRPDMKIMQEEIFGPVCAISKFKDEAEVIDLA
AN0554.1  	KKAGATVATGGDRHGNEGYFIQPTVFTDVTSDMKIAQEEIFGPVVTIQKFKDEAEAIKIG
          	*. ****  **:* *::****:**:*::*  :*** :******* :: **. * *.* :.

MG03900.1 	NDTNYG~LAAAVHTKDLNTSIRVSNALKAGTVWVNCY~NMLHHQLPFG0GFKESGIGREL
NCU03415.1	NDSNYG~LAAAVHTKDLNTAIRVSNHLRAGTVWVNTY1NALHHQLPFG~GYKESGIGREL
FG00979.1 	HDTAYG~LAAAVHTKNLNTALRVSNALKAGTVWVNCY~NMLHHQLPFG~GYKESGIGREL
AN0554.1  	NSTDYG1LAAAVHTKNVNTAIRVSNALKAGTVWINNY~NMISYQAPFG~GFKQSGLGREL
          	:.: ** ********::**::**** *:*****:* * * : :* *** *:*:**:****

MG03900.1 	GEAALANYTQNKS0VAIRLGGPIF-
NCU03415.1	GEAALANYTQCKS~VAIKLN-----
FG00979.1 	GEAALANYTQNKS~VAIKLY-----
AN0554.1  	GSYALENYTQIKT~VHYRLGDALFA
          	*. ** **** *: *  :* ..  :
```
