# Supplementary material for: Patterns of Intron Gain and Loss in Fungi
Source: PLoS Biol. 2004 Nov 30;2(12):e422. doi: 10.1371/journal.pbio.0020422 (PMC532390; doi:10.1371/journal.pbio.0020422)
Supplement: Table S1 — Also available at http://genes.mit.edu/NielsenEtAl/. (4.3 MB ZIP). [file pbio.0020422.st001.zip › NielsenEtAl/html/1107.html]

AN0727.1.NCU01637.1.MG01157.1.FG04292.1


```
 CLUSTAL W (1.82) Multiple Sequence Alignments - Introns Inserted


Sequence 1: NCU01637.1	489 aa
Sequence 2: MG01157.1	487 aa
Sequence 3: FG04292.1	456 aa
Sequence 4: AN0727.1	591 aa
Alignment Length: 593 aa
Number Identitical Residues: 205 aa
Alignment Score (without introns) 10356


MG01157.1 	------------------------------------------------------------
NCU01637.1	------------------------------------------------------------
FG04292.1 	------------------------------------------------------------
AN0727.1  	MADMSGNMLKRPHPDDEDNNAQKRPRSNNGSPHPGQGAPAAGNIDIEKIVAEARAKAQAV
          	 :. :..  . . .......:.. . :...:. ....:.::.. . ..  :.: :.:.: 

MG01157.1 	-----MDRGHSPGLGPRPDSASRVQKPETAADKMA---ALKARVAAAIGSS---------
NCU01637.1	-----MDRGQPSGLGPRHDATSRVQKPESAADRMA---ALKARVAAAVGGS---------
FG04292.1 	---------------------------------MA---ALKARVAAAIGTS---------
AN0727.1  	RDRLMAEKRVSASPSPAPAASSPSPAPPAASSTMSRIEQMKARVAAATGRSQAAAQQPSA
          	 .    .   ... ..   :::    . :::. *:  . :******* * *.:::...::

MG01157.1 	-------------------KAKGGLNVGLHPMLEELNKPKSSVKASDSRSSSLSSNRATG
NCU01637.1	-------------------KAKGGLNTPLHPALADLGAPIKPGDSLAAAAAGRRAAQKVA
FG04292.1 	-------------------KAKGGLNVGLHPALEDLGSHKPSNKSKESTPAPSGAR----
AN0727.1  	PTPPPLPRAPEDDEDDSLSRARGGLDVGLHPALLSDTLDFRGSKGRQVQSRNRRTESPGV
          	.:... . :.......: ::*:***:. *** * .        ..    .    :     

MG01157.1 	TKYGHRNSESGAGAGLGDRQSNPYLDTVATG-GQSTKQREPRHLVFNQKGKYIAQGNALR
NCU01637.1	ESFNRKEHRTPSATTTGQPRANPYLDTSSHG-PQG-KPKEPRQLIFNQKGKYIAQANALR
FG04292.1 	SDKPRSQDALRSASVNRESGENPYFDQSSSAQPGGGKARQSRSLVFNQKGKYIAQANALR
AN0727.1  	SGKQERAGLDLSGPSLEEIKNNPYYDPNLGPKATISKPRQSRQLLFNQKGKYIQQAAALR
          	    .      :..   :   *** *     .    * ::.* *:******** *. ***

MG01157.1 	RQAALEAMKKRIAEQTRKTGIDEDLDVPVTFIEE-PPDIEWWDEGLVDGQNYDRIEDEST
NCU01637.1	RQAALEEMKRRIAEQARKAGLDEDRDIEKAFVVEAPPDVEWWDQGLIDGNDYSNIPD--S
FG04292.1 	RQAALEAMKKRIAEQTRKAGIDDDLDVERKFVVEAPPEIEWWDEGLVEGGSYDVLDDPSK
AN0727.1  	RQAQLEEMKKRIAERARQAGIDEDLDVEKAFMVPAPPAIEWWDEHLVNEPDYAAIDDENN
          	*** ** **:****::*::*:*:* *:   *:  :** :****: *::  .*  : * ..

MG01157.1 	LRITSDDSIITELIQHPVALEPPQDKNVQAPKPMFLTAKEQKKLRRQRRAADLKEKQAKV
NCU01637.1	IKVNTPDSVVTIYIQHPVAIEPPQDKLAAEPKPMYLTPKEQQKLRRQRRMMELKEKQAKI
FG04292.1 	LKFTTPDTIITEYIQHPVALEPPQDGHVPAAKPMFLVKKERQKLRRQRRMAELKETQAKI
AN0727.1  	LKIDSADSIITRYIQHPVLLEPPQEKLKPEQKPMYLTPKEQAKIRRQRRMADLKEQQAKI
          	::. : *:::*  ***** :****:      ***:*. **: *:*****  :*** ***:

MG01157.1 	RLGLLPPEPPKITRNNMMVVLADQ~AVKDPTAVEAQVNREVAKRHEGHLQANEERKLTKE
NCU01637.1	RLGLEPAPPPKVKKSNLMRVLGEE~AVKDPTAVEARVNREIQERFDKHMQSNEERKLTKE
FG04292.1 	RLGLVPAPPPKVKKGNLMRVLGDV1AVKDPTAVEARVNREIAERHQKHVESNEERKLTKD
AN0727.1  	RLGLEPAPPPKVKKSNLMRVLGEQ~AVKDPTAVEARVNREIAERREKHEATNEERKLTKE
          	**** *. ***:.:.*:* **.:  **********:****: :* : *  :********:

MG01157.1 	QRHEKLAANQEKDASKGLHLLVFKINSLANGQHRYKIAVNAEQHALTGICIMHPRFSIVI
NCU01637.1	QRHEKLAQNQQKDVQKGVHTLVFKIGSLANGKHRFKIGQNAQQHALTGACIMHPKFCLVI
FG04292.1 	EKHEKLAANQQKDAEKGIHMLVFKIGSLANGQHRYKIGINADQLALTGTCIMHPKFNLVI
AN0727.1  	QRHEKLARQQAQDAEKGLIMTVYRIDSLANGRHRFKISKNAEQNALTGVCVMHPKFNLVI
          	::***** :* :*..**:   *::*.*****:**:**. **:* **** *:***:* :**

MG01157.1 	VEGGAWSIAKYKKLMLNRINWTENT-PSRDRD-SAKAGVLRDWLKPEDDKGGLKDMSANQ
NCU01637.1	VEGGEHSVNAYKKLMMRRIDWTESV-PSLDREPSASGAAVREWLKAEDEQGMLKDLSNNK
FG04292.1 	VEGGEWGIKKFKKLMLNRIDWTENS-PSRDRD--GKQGATRDWLLAEKDTGELKDMSTNE
AN0727.1  	VEGGAHSSNNYRKLMMNRIDWTENAGPSAVRE--GNREAQASWLAAEDEKGELKDLSSNT
          	****  .   ::***:.**:***. .**  *:  ..  .  .** .*.: * ***:* * 

MG01157.1 	CKLVFEGEVKTRAFRKWG-SKVCETDAEARDVLARVKMENFWAQARTVT-----
NCU01637.1	CQLIFEGESKTQSFKKWS-SKVCETDQEAREFLSQMKMENFWTQAKNTPSHV--
FG04292.1 	CKLIFEGEEKARAFRKWG-SKVCETDSEARDALARTKMDNFWQLAKGFA-----
AN0727.1  	CTLLWEGQVKARAFRKWLGARVCETDSQAKDVLARAKLESFWTLAKSAKQQGEF
          	* *::**: *:::*:** .::***** :*:: *:: *::.**  *:   .  .
```
