# Supplementary material for: Patterns of Intron Gain and Loss in Fungi
Source: PLoS Biol. 2004 Nov 30;2(12):e422. doi: 10.1371/journal.pbio.0020422 (PMC532390; doi:10.1371/journal.pbio.0020422)
Supplement: Table S1 — Also available at http://genes.mit.edu/NielsenEtAl/. (4.3 MB ZIP). [file pbio.0020422.st001.zip › NielsenEtAl/html/1108.html]

AN3648.1.NCU02758.1.MG05646.1.FG01291.1


```
 CLUSTAL W (1.82) Multiple Sequence Alignments - Introns Inserted


Sequence 1: NCU02758.1	515 aa
Sequence 2: FG01291.1	473 aa
Sequence 3: MG05646.1	494 aa
Sequence 4: AN3648.1	478 aa
Alignment Length: 522 aa
Number Identitical Residues: 201 aa
Alignment Score (without introns) 9737


MG05646.1 	MPP0A~RTARGQLVSNENDENAGSTRMTRTKAKAAALNVDELALPTKQLQTKKGVAGKPA
NCU02758.1	MPN~L0RTRNPRSLANENDENSTATTTRMTRAKAATLHVDELSMPAKTMQTKKTTISTTT
FG01291.1 	MPT0V~RATRTR---NENDENS--GTTRLTRAQAAALKVDELSMPAKAALQTKKSTANGT
AN3648.1  	---~-~--------MNENDENG--PSTRLTRAKAAALTTDAPAANGALKKPLQTKKAATG
          	               ******.       *:*:**:* .*  :         :   .   

MG05646.1 	PTTRTT----RNALGDVSNVTKVD----AGRKAVGGKAGAVAKAAAPAG-VQKSSARTAP
NCU02758.1	AATAARTVGKRNALGDVSNVTKVEV---AGTKKAIAKPGLVSKAAQPSG-IQKKTTTATS
FG01291.1 	AAANTR---KRAALGDVSNVGKADG---VAGKKAK---GLVSKAAQPTG-IEKKTARPT-
AN3648.1  	ANGTQR---KRAALGDVSNVGKADNGETKDAKKATSKTGLTSKATMQSGGVQKLSRSNLS
          	.        .* ******** *.: ..:   * . ...* .:**:  :*.::* :    .

MG05646.1 	-----TRSALSSKPANVTNGNAEKQSGRGTLS-KRKAPAQAAANN-IKEESTLEGEPARK
NCU02758.1	RTATTARRALSNKEPNKTVG---PSAGAGTIPAKRKPPPSTSKLAPIKESAPVENEPARK
FG01291.1 	------RPALASQTANSKP----AQSGSGTINNKRKVLTDTKPKAPVKKTEPTSKEPE--
AN3648.1  	------RTAVGAKDNNVKKP-ATEAKRPGSGSGMGSAMKRTSSQKSLQEKTIQQEEPPRK
          	      * *:. :  * .   :      *:     .    :    .:::    . **  .

MG05646.1 	QARVAATA---AVDTKRAAPAKELKCVEPELKPTREFIRDPRLLAGEVPPGVIDLSMDDY
NCU02758.1	KIHVEEPEKKKVSRTEAKENDAPSKAVKPMAEPPAPVVRDVVPVQSVYPPGVKDLDTEDL
FG01291.1 	--LTEENE-----RSETPEEAEVEKPEVSVEKP------EVQDAPFKYPPGVNNLDEEDL
AN3648.1  	KVDIEKVVEKQAEAVSVKGDVKAGAQTEELEKP---------------QDFVADLDTEDL
          	.       ...    .               :*                  * :*. :* 

MG05646.1 	DDPLMVAEYAEEIFSYMLNLELSSMPNPNYMDHQDDVEWKTRGILIDWLIEVHTRFHLVP
NCU02758.1	EDPLMVAEYATEIFEYLRDLECKSVPNPQYMSHQDDLEWKTRGILIDWLIEVHTRFHLLP
FG01291.1 	EDPLMVAEYANEIFEYLRDLECKSIPNPQYMSHQDDLEWKTRGILVDWLIEVHTRFHLLP
AN3648.1  	DDPLMAAEYVVEIFDYLRELEMETLPNPDYIDHQPDLEWKMRGILVDWLIEVHTRFRLLP
          	:****.***. ***.*: :** .::***:*:.** *:*** ****:**********:*:*

MG05646.1 	ETLFLAVNIVDRFLSEKVVQLDRLQLVGITAMFIASKYEEVMSPHVTNFRHVTDDGFSES
NCU02758.1	ETLFLAVNIIDRFLSEKVVQLDRLQLVGITAMFVASKYEEVLSPHIANFRHVADDGFTEA
FG01291.1 	ETLFLAINVIDRFLSEKVVQLDRFQLVGITAMFIASKYEEVLSPHVENFKRIADDGFSEA
AN3648.1  	ETLFLAVNIIDRFLSAEVVALDRLQLVGVAAMFIASKYEEVLSPHVANFSHVADETFSDK
          	******:*::***** :** ***:****::***:*******:***: ** :::*: *:: 

MG05646.1 	EILSAERFILSTLNYDLSYPNPMNFLRRVSKADNYDTPCRTIGKYLMEISLLDHRFLQYR
NCU02758.1	EILSAERFILSTLNYDLSYPNPMNFLRRISKADNYDIQSRTLGKYLMEISLLDHRFMPYR
FG01291.1 	EILSAERFVLSTLNYDLSYPNPMNFLRRVSKADNYDIQSRTIGKYLMEISLLDHRFMAYR
AN3648.1  	EILDAERHILATLEYNMSYPNPMNFLRRISKADNYDIQTRTLGKYLMEISLLDHRFLGYP
          	***.***.:*:**:*::***********:*******   **:**************: * 

MG05646.1 	PSLVAASAMALSRIILDRGEW0DKTISYYSGYNEDDVEPVVNLMVDYLSRPVIHEAFFKK
NCU02758.1	PSHVAAAAMYLARLILGRGEW0DKTIAYYAGYTEEEIEPVFHLMVDYLARPVIHEAFFKK
FG01291.1 	PSHVAAGAMYLARLMLDRGEW0DATLSYYAGYTEDEVEPVVHLMVDYLARPVVHEAFDKK
AN3648.1  	QSQIGAAAMYLARLILDRGPW0DATLAHYAGYTEEEIDEVFRLMVDYLHRPVCHEAFFKK
          	 * :.*.** *:*::*.** * * *:::*:**.*:::: *..****** *** **** **

MG05646.1 	YASKKFFKA1SILSRNWVEENGYLFGIDQTDVAIDQL---------
NCU02758.1	YGSKKFLKA1SILTRQWAKKNAVLYGVVDAEIPLDKLKDRPVDQLP
FG01291.1 	YAAKKFLRA1SLLARQWAKKNAVLFGITDIELGLDQIS--------
AN3648.1  	YASKKFLKA1SIMTRQWAKKYHHLYIDSALTEPYNSIKDNE-----
          	*.:***::* *:::*:*.::   *:         :.:..
```
