# Supplementary material for: Patterns of Intron Gain and Loss in Fungi
Source: PLoS Biol. 2004 Nov 30;2(12):e422. doi: 10.1371/journal.pbio.0020422 (PMC532390; doi:10.1371/journal.pbio.0020422)
Supplement: Table S1 — Also available at http://genes.mit.edu/NielsenEtAl/. (4.3 MB ZIP). [file pbio.0020422.st001.zip › NielsenEtAl/html/1109.html]

AN2745.1.NCU01276.1.MG07515.1.FG01906.1


```
 CLUSTAL W (1.82) Multiple Sequence Alignments - Introns Inserted


Sequence 1: NCU01276.1	190 aa
Sequence 2: MG07515.1	190 aa
Sequence 3: FG01906.1	190 aa
Sequence 4: AN2745.1	173 aa
Alignment Length: 190 aa
Number Identitical Residues: 84 aa
Alignment Score (without introns) 4905


MG07515.1 	MANFRQFVPSDLSKLSKCNLDPFTETYDLSFYLQYHAKWPSLFQVAEDQHGNIIGYI1MG
NCU01276.1	MANFRQFRPDDLNKFSKCNLDPFTETYELGFYLQYHAKWPSLFQVAEDQHGNIIGYI1MG
FG01906.1 	MATFRRFRPDDVNKFSKCNLDPFTETYELNFYLQYHAKWPSLFQVCEDMDGNIVGYI1MG
AN2745.1  	MTSIRRMSPTDLFSLNLTNLDPLTENYDLGFYLNYLMRWPSLFSSVKDRREGIAGYI1MG
          	*:.:*:: * *: .:.  ****:**.*:*.***:*  :*****.  :*   .* *** **

MG07515.1 	KLESSPDIYKYSEHYLPWHAHITAVTVAPEARRLGIGKLLSQQLEAAADAGDAWFVDL~F
NCU01276.1	KLESSPDYYKFSEHYLPWHAHITAVTVAPEARRLGIGKMLTEQLEAAADANDAWFVDL~F
FG01906.1 	KVESSPDAYKFSEHYLPWHAHITALTVAPEARRSGIAKILTDQLEVAADAENAWFVDL~F
AN2745.1  	KLEEQHPSLKASEHYTPWHGHITVLTVAPAWRRLGHARRLTERLERGSDINNAWFVDL2-
          	*:*..    * **** ***.***.:****  ** * .: *:::** .:*  :******  

MG07515.1 	VRKSNHKAIRFYKSMGYSVFRVVKDYYGDHSSDPTLDSEDAYDMRKPCARDVKRKHIRDD
NCU01276.1	VRSTNHKAIQFYKSMGYSIFRTVKDYYGDHSSDPTKSSEDAYDMRKPMKRDVKKEHIREN
FG01906.1 	VRSSNHRAITLYKNLGYSVFRVVKDYYGDHATDPSKSSEDAFDMRKPMKRDKDHQHIRDD
AN2745.1  	----------------YSVFRRVVNYYSDDPTGMSEKGEDAFDMRKPCSRDKKLEHIREN
          	                **:** * :**.*..:. : ..***:*****  ** . :***::

MG07515.1 	GENHEVNPEDVW
NCU01276.1	GEKFEVDPSDVW
FG01906.1 	GENHLVNPEDVW
AN2745.1  	GENFPVSPEHVS
          	**:. *.*..*
```
