# Supplementary material for: Patterns of Intron Gain and Loss in Fungi
Source: PLoS Biol. 2004 Nov 30;2(12):e422. doi: 10.1371/journal.pbio.0020422 (PMC532390; doi:10.1371/journal.pbio.0020422)
Supplement: Table S1 — Also available at http://genes.mit.edu/NielsenEtAl/. (4.3 MB ZIP). [file pbio.0020422.st001.zip › NielsenEtAl/html/111.html]

AN3785.1.NCU08357.1.MG03737.1.FG00667.1


```
 CLUSTAL W (1.82) Multiple Sequence Alignments - Introns Inserted


Sequence 1: NCU08357.1	732 aa
Sequence 2: MG03737.1	649 aa
Sequence 3: FG00667.1	710 aa
Sequence 4: AN3785.1	684 aa
Alignment Length: 754 aa
Number Identitical Residues: 297 aa
Alignment Score (without introns) 15794


MG03737.1 	-------------------------------------~---MS~A2CRIPD---------
NCU08357.1	MKATPLIINWHHENNPYPIYSAHFEPGGKGRLATAAG~DNNVR0L~WRIEEDGENRRVEY
FG00667.1 	MKAAPLIINWHDQNAP--VYSAHFEPTGKGRLATAGG~DNHIR~I~WKVQVDGEDRKVEY
AN3785.1  	MKANPLLIAWHNDNAP--IYSVHFDPNGKGRLATAGN2DNNVR0L~WKVEPVGQERKVTY
          	 .: .       .. .    :   .. ...  :::.. .. :     ::   ...     

MG03737.1 	-----------------MEG1DLLASAGDDGNIILWIPAENHLPASFGSEGLEDKETWRT
NCU08357.1	LATLSKHTQAVNVVRWAPKG1ELLASAGDDGNVILWVPSDTHH-ATFGNEGLEDKETWRT
FG00667.1 	LSTLSKHNQAVNVVRWAPKG1ELLASAGDDGNVILWVPSETPQ-TAFGSDAPEDKESWRA
AN3785.1  	LSTLVKHTQAVNVVRFSPKG1EMLASAGDDGNVLLWVPSELQTQPGFGEDRADDKETWRV
          	 ::  . ..: .    : :* ::*********::**:*::    . **.:  :***:**.

MG03737.1 	KNMCRSSGAEIYDLAWSPDSQYFIIGSMDNVARIYSAATG~ALVRQIAEHSHYVQGVAWD
NCU08357.1	KHMCRSLGTEIYDLAWSPDAAFFIIGSMDNVARIYNASTG~TLVRQIAEHSHYVQGVTWD
FG00667.1 	KHMCRSSGAEIYDLAWSPDGVHFMIGSMDNIARIYNAQTG1SLVRQIAEHSHYVQGVTWD
AN3785.1  	KHMCRSSGAEIYDLAWSPDGVFIITGSMDNIARIYNAQTG1QMVRQIAEHSHYVQGVAWD
          	*:**** *:**********. .:: *****:****.* **  :**************:**

MG03737.1 	PLDEFIATQSSDRSVHIYALRTKDGKYTLSG-HEDKPQKIAGHMKTDLPPRRISSHSPAP
NCU08357.1	PLNEYIATQSSDRSVHIYSLRTKDGQYTLANNHDDKPPKIASHAKTDLPPRRISSHSPAP
FG00667.1 	PLNEYIATQSSDRSVHIYSLKTKDGQYTLNV--DDKPPKLASHIKADLPPRRISSSSPAP
AN3785.1  	PLNEFVATQSSDRSVHIYSLKTKDGQFTLTS--------HGKFLKMDLPARRISRSSPAP
          	**:*::************:*:****::**           . . * ***.****  ****

MG03737.1 	PEFGHRSQVSTLD-SVAASSPAPSAPGTPTSMALPMNPPSVISHSRRSSFSS----RRSV
NCU08357.1	PEFGNRPAFTSLDPSAVAGSPNPSIPGTPTSMALPMNPPSVISHSRRSSFSS----RRSV
FG00667.1 	PDFGHRSSLSVLDPPPSIGSPVPSAPGTPTSFALPMNPPSVVSHSRRSSFSS----RRSV
AN3785.1  	LDLSVRSQPATSN-SVAVASPAPSTPGTPMTSHLPMDPP-PVSHSRRSSFSSSPNIRRSA
          	 ::. *.  :  : .   .** ** **** :  ***:**  :**********:.. ***.

MG03737.1 	SPAPSMPLPAVMPMEPSPKPHPSAS-LGMKNASLYHNETLTSFFRRLTFTPDGSLLITPA
NCU08357.1	SPAPSMPLPAVMPIEASPKPHSSSYGLGMKNASLYANETLTSFFRRLTFTPDGSLLLTPA
FG00667.1 	SPAPSMPLPAVMPMDPSPKPSAMSSGMGMKSANLYANETLTSFFRRLTFTPDGSLLLTPS
AN3785.1  	SPAPSLPLPAVRPLEVTS---PGLGGLGVRNANIYANETFTSFFRRLTFTPDGSLLLTPA
          	*****:***** *:: :.   .   .:*::.*.:* ***:****************:**:

MG03737.1 	GQYQTQHQVE--GSKPTYEVTNTVFIYSRGGINKPPICHLPGHKKPSVVVRCSPIIYTLR
NCU08357.1	GQYQTQHQVD--GGKPLYEVINTVYIYTRGGINKPPIAHLPGHKKPSVAVRCSPIIYTLR
FG00667.1 	GQYQNQHQAER-DAKPTYEVINTVYIYTRGGINKPPIAHLPGHKKPSVVVKCSPIFYTLR
AN3785.1  	GQYKTSQVSSTDPGKTVDEVINTVYIYTRAGFNRPPISHLPGHKKPSVAVKCSPILYTLR
          	***:..:  . . .*.  ** ***:**:*.*:*:***.**********.*:****:****

MG03737.1 	QSLPATRHITIDTSSAEEPIPSLPEPVSKPSPATSVMDPPPPPGGEVIGTTGKPPNPEAP
NCU08357.1	QSPPVTKHITIDTSSAEEPIPSLPEPLAKPSTAPSVMEPPPPPLPTESSVSG---STEAP
FG00667.1 	QSPPVTRNITIDTSSSEEPIPALPEPLSKPSPAPSIMEPPPPPANTSETKPS---GVDAA
AN3785.1  	EAPEPSKHITLGTTSAEDASATSTGEESK----NNITAKP----------------FESP
          	::   :::**:.*:*:*:. .: .   :*     .:   *                 ::.

MG03737.1 	SASPGPKPAFALPYRMVYAVATQDSVLLYDTQQMTPICIVSNLHCATFTDLAW2SKDGHT
NCU08357.1	VQSPGPKSAFALPYRMVYAVATQDSVLLYDTQQHTPICVVSNLHCATFTDLTW2STDGLT
FG00667.1 	STTPGPKPAFSLPYRMVYAVATQDSVLLYDTQQKTPICVVSNLHCATFTDLAW2SSDGLT
AN3785.1  	TANQLPPPVFSLPYRVVYAVATQDAVLVYDTQQQAPLCVVSNLHFATFTDLAW2SNDGLT
          	  .  * ..*:****:********:**:***** :*:*:***** ******:* *.** *

MG03737.1 	LLISSSDGFCSTLSFSPSDLGQVYTGELPLRQSVTPTTTVLSSQNTPAATPTTVPAPPSP
NCU08357.1	LLISSSDGFCSTLSFLPGELGIPYTGEIGPRHSLS-SGGATSNQNTSLPNPSGVFAPPSP
FG00667.1 	LMISSSDGFCSALSFAAGELGDIYKGEVGPPKPQT---AASSNQSTPLPTPTNAFAPPSP
AN3785.1  	LIMSSSDGFCSTLSFAPGELGQPYT-------------APLSAPQTTGTGPTSALHTP--
          	*::********:*** ..:**  *.                *  .*. . *: .  .*  

MG03737.1 	FHASQSH-HRTASSSFAAPSPP-AFATAGQRPSSPARSNSTCSVVTQGS--------GIL
NCU08357.1	FHNGTNHRHRDSASSFTAPSPPTAASFVNPPPPSPARSNSTSSAITQASTS----QGGVM
FG00667.1 	FPNGSHHQHRNSASSFTAPSPPQSASVASQRPSSPARSNSTSSIATITTQASTVPAAGVV
AN3785.1  	----------------TDPTKPAHLQTAPPPPASPARSNSVSSIATQRASQQ---AETVV
          	                : *: *     .   *.*******..*  *  :  .      ::

MG03737.1 	NAPTLIGGTVPGIAATNSGKVTG-VPITTPPETPRSSVANPSTVTASGVKRDISESDKE-
NCU08357.1	SNPPLISGQVPSIAATNSGKVTG-LPVVTPPETPQSSSGGAGSTNLKRESSDSREANNA-
FG00667.1 	TNPTLISGNVPGIAATNSGKVTG-VPLTTPPETPRS-----ANATVVGSKREASESEK--
AN3785.1  	NNPTPTLGSVPLVTATHSAQPPATLPLTTPPETPLSQSEASNAGSTVLGKRSESEKEESQ
          	. *.   * ** ::**:*.: ..::*:.****** *.   .   .    . .  * :: .

MG03737.1 	-----EASQEPKKRRIAPTPVG--------DSHNS-----
NCU08357.1	-----ENSQQPKRRRIAPTLVSGGASSTTDGSSNSDVAKA
FG00667.1 	-----EDVKEPKKRRIAPTLVE---------PKN------
AN3785.1  	GLTPTEQAQPPKKRRVAPTLISAGSGSSNEGPPDTNVGG-
          	. :.:*  : **:**:*** : ..:.::.... ::. .
```
