# Supplementary material for: Patterns of Intron Gain and Loss in Fungi
Source: PLoS Biol. 2004 Nov 30;2(12):e422. doi: 10.1371/journal.pbio.0020422 (PMC532390; doi:10.1371/journal.pbio.0020422)
Supplement: Table S1 — Also available at http://genes.mit.edu/NielsenEtAl/. (4.3 MB ZIP). [file pbio.0020422.st001.zip › NielsenEtAl/html/1110.html]

AN1918.1.NCU09873.1.MG00450.1.FG08601.1


```
 CLUSTAL W (1.82) Multiple Sequence Alignments - Introns Inserted


Sequence 1: NCU09873.1	561 aa
Sequence 2: MG00450.1	548 aa
Sequence 3: FG08601.1	579 aa
Sequence 4: AN1918.1	556 aa
Alignment Length: 582 aa
Number Identitical Residues: 425 aa
Alignment Score (without introns) 18245


MG00450.1 	---------------------------------MHPSGVQ2AHIPHEHTELE1EELHEKA
NCU09873.1	------------------MSRLVFNHDHVNKTNLHPGGVK2PHV--EHTELE~EELHQKA
FG08601.1 	MIEPLHPISTIPPVQKNNTDLVAVLPNNVNKTSLHPTGVT2PHQ--EHTELE~QELHDKA
AN1918.1  	-----------------------MVATSVNRTALHPGGVQ2PGK--GHTELE~EELHEHA
          	                             . : :** **  .     ***** :***::*

MG00450.1 	HIDYDRVAI0IPNPSVPALYEDALVYEPGSAISSTGALTAYSGNKTGRSPLDKRVVKEPT
NCU09873.1	HIDYDRVAI0IANPSVASLYEDALVYETGTAITSSGALTAYSGKKTGRSPSDKRIVKEPS
FG08601.1 	HIDYDRVAI0IPNPSVAALYEDALVYETGTAITSSGALTAYSGAKTGRSPLDKRIVEEAS
AN1918.1  	HIDYDRVAI0IANPSVAALYEDALVYETGTAITSSGALTAYSGAKTGRSPSDKRIVKEES
          	********* *.****.:*********.*:**:*:******** ****** ***:*:* :

MG00450.1 	SENDIW2WGPVNKPMSPD0VWKINRERAIDYLNTRSRIYVVDGYAGWDEKYRIRVRVVCA
NCU09873.1	SENDIW2WGPVNKPMSPE0VWKINRERAVDYLNTRNRIYVVDGYAGWDEKYRIRVRVVCA
FG08601.1 	SKDNIW2WGPVNKPMTPE0VWKINRERAVDYLNTRSRIYVIDGFAGWDEKYRIKVRVICA
AN1918.1  	SEKEVW~WGPVNKPMTPD0VWRINRERAVDYLNTRNRIYVIDGFAGWDERYRISVRVVCA
          	*:.::* ********:*: **:******:******.****:**:*****:*** ***:**

MG00450.1 	RAYHALFMRNMLIRPPREELEHFQPDYTIYNAGTFPANRYTEGMTSGTSVAINFAEKEMV
NCU09873.1	RAYHALFMRNMLIRPPREELEHFHPDYTIYNAGSFPANRYTEGMSSSTSVAINFAEKEMV
FG08601.1 	RAYHALFMRNMLIRPTREELNDFHPDYTIYNAGKFPANRYTEGMTSGTSVAINFEQKEMV
AN1918.1  	RAYHALFMRNMLIRPSAEELKHFHPDYVIYNAGSFPANRFTEGMTSATSVAINFAEKEMV
          	***************. ***:.*:***.*****.*****:****:*.******* :****

MG00450.1 	ILGTEYAG~EMKKGIFTVLFYEMPIKHNVLTLHSSANEGKNGDVTLFFGLSGTGKTTLSA
NCU09873.1	ILGTEYAG~EMKKGIFTVMFYEGPVKHNILTLHSSANEGKDGDVTLFFGLSGTGKTTLSA
FG08601.1 	ILGTEYAG1EMKKGVFTVLFYEMPIKHNVLTLHSSANEGKNGDVTLFFGLSGTGKTTLSA
AN1918.1  	ILGTEYAG~EMKKGVFTILFYEMPVKHNVLTLHSSANEGQNGDVTVFFGLSGTGKTTLSA
          	******** *****:**::*** *:***:**********::****:**************

MG00450.1 	DPNRMLIGDDEHCWSDSGVFNIEGG0CYAKCIGLSAEKEPDIFGAIQFGSVLENVVFDPN
NCU09873.1	DPNRRLIGDDEHCWSDRGVFNIEGG~CYAKTIGLSAEKEPDIFNAIRYGSVLENVVFNPE
FG08601.1 	DPNRALIGDDEHCWSDNGVFNIEGG~CYAKTIGLSAEKEPDIYGAIRYGSVLENVVFDPL
AN1918.1  	DPKRALIGDDEHCWTDRGVFNIEGG~CYAKCIGLSAEKEPDIFNAIRFGSVLENVVFDPI
          	**:* *********:* ******** **** ***********:.**::*********:* 

MG00450.1 	TRVVDYDDCTLTENTRC2AYPIEYISNAKIPCLSNNHPSNIILLTCDARGVLPPISKLNS
NCU09873.1	TREVDYGDATLTENTRC~AYPIEYIPNAKIPCLSPNHPKNIILLTCDARGVLPPISKLDS
FG08601.1 	TREVDYDDATLTENTRC~AYPIEYISNAKIPCLSPNSPSNIILLTCDARGVLPPISKLDS
AN1918.1  	SRVVDYDDSTLTENTRC~AYPIEYIENAKVPCLSDSHPSNIILLTCDARGVLPPISKLTT
          	:* ***.*.******** ******* ***:**** . *.******************* :

MG00450.1 	AQTMFHFISGYTSKMAGTEDGVTEPQATFSSCFAQPFLALHPMRYAKMLADKIEHHNANA
NCU09873.1	AQTMFHFISGYTSKMAGTEDGILEPQATFSSCFAQPFLALHPMRYAKMLAEKIENHNANA
FG08601.1 	AQTMFHFISGYTSKMAGTEDGVTEPQATFSSCFAQPFLALHPMKYAKMLADKIETHKANA
AN1918.1  	EQTMFHFISGYTSKMAGTEDGVTEPQATFSSCFAQPFLALHPMRYARMLADKISQHKANA
          	 ********************: ********************:**:***:**. *:***

MG00450.1 	WLLNTGWVGAGAAQGGKRCPLKYTRAILDAIHSGELAKQEYETYEVFNLQVPKSCPGVPS
NCU09873.1	WLLNTGWVGAGFAQGGKRCPLKYTRAILDAIHSGELANVEYENYEVFNLQVPKSCPGVPS
FG08601.1 	WLLNTGWVGAGFAQGGKRCPLKYTRAILDAIHSGDLANVEYENYGVFNLQVPKSCPNVPS
AN1918.1  	WLLNTGWVGAGATTGGKRCPLKYTRAILDAIHSGELAKAEYETYDVFNLHVPKSCPGVPD
          	*********** : ********************:**: ***.* ****:******.**.

MG00450.1 	ELLNPKAAWTAGNDSFDTEVKKLGGLFLENFKKYESEATEDVIKAGPVV-
NCU09873.1	ELLNPKTAWTAGANSFDTEVKKLGGLFLENFKKYESEATEDVIKAGPVV-
FG08601.1 	ELLNPSKAWTAGEDSFNTEVVKLGKLFRENFAKYESEATEDVVKAGPVV-
AN1918.1  	ELLNPKNSWTATT-SFSDEVNKLAKLFNENFQKYADQATKEVIAAGPVVQ
          	*****. :***   **. ** **. ** *** ** .:**::*: *****.
```
