# Supplementary material for: Patterns of Intron Gain and Loss in Fungi
Source: PLoS Biol. 2004 Nov 30;2(12):e422. doi: 10.1371/journal.pbio.0020422 (PMC532390; doi:10.1371/journal.pbio.0020422)
Supplement: Table S1 — Also available at http://genes.mit.edu/NielsenEtAl/. (4.3 MB ZIP). [file pbio.0020422.st001.zip › NielsenEtAl/html/1111.html]

AN6743.1.NCU01000.1.MG08785.1.FG04970.1


```
 CLUSTAL W (1.82) Multiple Sequence Alignments - Introns Inserted


Sequence 1: MG08785.1	591 aa
Sequence 2: FG04970.1	592 aa
Sequence 3: AN6743.1	793 aa
Sequence 4: NCU01000.1	595 aa
Alignment Length: 829 aa
Number Identitical Residues: 293 aa
Alignment Score (without introns) 15216


MG08785.1 	-----MPNPTK-DLHTVD-ETSFP---YIFEQNATVQLKTCDGLIRCNVYRPK-------
NCU01000.1	MGDASQPRTIHGPPRLLDIQTTVPDPRILFMKNVSIPLKSSPFPIRANIYLPKPCASSPG
FG04970.1 	-----MPNQIKEDLHTVD-EKSFP---YIFEQNATVALKAGDGLVRLNVYRPK-------
AN6743.1  	-----MPNPIRADITTID-STSFP---YIFEQNVTITLKDNSGLVRCNVYRPK-------
          	      *.  :     :* ..:.*    :* :*.:: **     :* *:* **       

MG08785.1 	----------NSGPAPVLVTYGPYGKDIHYHD2FHPKSYAEVNPNHKSDHSAWETPDPKF
NCU01000.1	LDVNEPQPEVQSKKYPVIVTYGPYGKDIPYSS~FHPGSFAEVNAEQRSEFSAWETPDPVY
FG04970.1 	----------GVDKVPVLVTYGPYGKDISYED2FHPKSFSEVNPQHKSEHSAWETPDPAF
AN6743.1  	----------SPEKVPVLVTYGPYGKDIPYAD~FHPKSFSEVNPEHRSQHSAWETPDPAF
          	               **:********** * . *** *::***.:::*:.******** :

MG08785.1 	WTRHGYVVVRADERGLGQSPGVLDTMS~RGTSDAFCELIEWAADQPWSSGKVGLLGISYY
NCU01000.1	WCKQGYAVVRADERGTGQSPGLLDTMS1--------------------------------
FG04970.1 	WTKNGYAIVRADERGLGQSSGKLDTMS~RGTSEAFFDVVEWAAEQPWSSGKVGLLGISYY
AN6743.1  	WTKKGYAVVRADERGTGQSRGKLDTMS~RETSEAFFDVVEWAAEQEWSSGKVGLLGISYY
          	* ::**.:******* *** * *****   ::.:  .  . ::..  ::.. .  . :  

MG08785.1 	AGSQWRVAARKPKGLAAMIPWEGMSDYYRDRCRHGGILSNAFIKFWWNRQVITNQYGRPG
NCU01000.1	--SQWRVAARRPKGLAAIIPWEGMSDYYRDRCRHGGILSNNFIDIWWNRQVLVNQYGLPG
FG04970.1 	AGSQWRVAARKPKGLSAIVPWEGMSDYYRDRCRHGGILSNAFIKFWWNRQVITNQYGRPG
AN6743.1  	AGSQWRVAARQPKGLACIIPWEGMSDYYRDRCRHGGILSNSFISFWWNRQVLSNQYGRPG
          	:.********:****:.::********************* **.:******: **** **

MG08785.1 	RSASNWGP---------DTIEGDLPADELERNRNDQTIDNQKHRFRDEEYYASKEYDMGD
NCU01000.1	RSELKFPPDGPSARGQEDTIEGDLSEEQLVSNRNDQTKDNEAHRFRDEDYYASKEYRLED
FG04970.1 	RSARNWGP---------DTIEGDLPDDELEANRQDQTIDNQVNRFRDDTYYASKEYDMGD
AN6743.1  	RAARNWGV---------DTIEGDLVEEELEANRQDQTVDNRINRFRDDLYYASKEYTMSD
          	*:  ::           *******  ::*  **:*** **. :****: ******* : *

MG08785.1 	IE~VPLLSVGNWGGILLHLRGNIEGYVQANSEFKYLRMITGRHDLPFYYDEEVEIQRSFL
NCU01000.1	IE~VPVLSVANWGGITLHLRGNVLGYTYAGSRFKYLRFITGRHDLPFYYKEHVELQKSFL
FG04970.1 	IE~VPLLSVGNWGGILLHLRGNIEGYIHAGSKFKYLRLITGRHDLPFYYEEEVEIQRSFL
AN6743.1  	IQ0VPLLSVANWGGILLHLRGNVEGYTQAGSQLKYLRFITGRHDLPFYYKEEVDIQLSFL
          	*: **:***.***** ******: **  *.*.:****:***********.*.*::* ***

MG08785.1 	DAFLKGEDRVGWATKGKLPSVDLVMRKGNVGFNDAQAERAYTRRAESQWPPASTVYTTYH
NCU01000.1	DAFLKGEDKVGWSIPDKVSPIEVTLRKGNVGFNDAEKEKAYKRRNEGQWPLYSTEYTDFY
FG04970.1 	DAFLKGEDRVGWSEEGKVSPVSLVLRKGNVGFNDAEKEKVYPRREESEWPIARTQYKKLF
AN6743.1  	DAFLKGDDRAGWST-GTAPKVDLVLRKGDVGFNNAEAEKTYARRVENEWPIARTQYTRFY
          	******:*:.**:  .. . :.:.:***:****:*: *:.* ** *.:**   * *.  .

MG08785.1 	CHPDGVLSTAAPSLPRRTKLSYRALGTLANPSLVSFSTPKFETETEITGHPRARLSVSVT
NCU01000.1	LGSDHTLSRNRPDPTMPAQIGYKALESLDKPELVQFVTAPFEKETEITGHIVAHLNVSVT
FG04970.1 	LTPEQGLSWGEPKTDRK-KISYKALGTLEKPEVLQFSTPAFEAETEITGHVVAHLNVSVS
AN6743.1  	LTPNRELLTTPPEQKGYSKLSYAALGNIENPSFIQFTTPPFEHETEITGHIVAHLNVSMS
          	  .:  *    *.    :::.* ** .: :*..:.* *. ** *******  *:*.**::

MG08785.1 	PDASGPTPSEIDVFLTLRYIGPDEKEVFYTGTAGDPVPLCKGWLRTSLRKVNESHRKHRA
NCU01000.1	PEN-TQNEADIDLFLTIRHIDRSGNEVFYTGTAGDPVPVCKGWLRVSNRKVHEENPRHKP
FG04970.1 	PDPSGPTPSDIDLFVTLRHIDPSGQEVYYTGTAGDPVPVTKGWLRVSLRKVDQEHPKNRE
AN6743.1  	PNPGAPTPTDIDLFLTLRYISPEGKEVYYTGTAGDPVPLCKGWLRVSLRKVNDKHARHRE
          	*: .  . ::**:*:*:*:*. . :**:**********: *****.* ***.:.: ::: 

MG08785.1 	YLPHRDYTSADVLPVIPGEVYTIDIEVWPTNVVVEKGGRIVFEVASGDTQGSGIFLHDDP
NCU01000.1	WLPFREYFSTDVLPVKAGEVYGVDVELWPTNVVVGVGGGILLEVSSGDTQGAGIFQHNSE
FG04970.1 	WLPHRNYTSKDVLPVIQGEVYAVDVEVWPTNVVVDKGGKLVFEVASGDTQGSGIFQHNDA
AN6743.1  	YLPHRDYFSTDVQPVIPGEVYPVDVEIWPTNVVVEKGGQIVLEVASGDTQGSGIFLHNEP
          	:**.*:* * ** **  **** :*:*:*******  ** :::**:******:*** *:. 

MG08785.1 	VD-~------------------------------~---------------------R~AV
NCU01000.1	TD-~------------------------------~---------------------R2PA
FG04970.1 	VD-~------------------------------~---------------------R2SP
AN6743.1  	TDS2GVSLHILDVFHAPDVDAVVPNTARPWLAQP0ASPKMSASNESSAPKNLRPLSR~YI
          	.*: . :    .   :.. .:  ..:: .  :.. ::.. :::..:::...  . :*   

MG08785.1 	EKLQGVNNINFGEGLENYLVLPVIP-----------------------------------
NCU01000.1	SKFAGQNHIHFGEGFDNYVTLPIIPEHI--------------------------------
FG04970.1 	EIFQGHNHIHFGPRQQNYITLPVIPN----------------------------------
AN6743.1  	TTHNAAGKAIFSESIAPTMPVTPIPDGADFSLAYTSPTIPASFANETDIAAYASYLEPGN
          	    . .:  *.      : :. **.  . : : ::.: .:: :..:. :: ::  ....

MG08785.1 	------------------------------------------------------------
NCU01000.1	------------------------------------------------------------
FG04970.1 	------------------------------------------------------------
AN6743.1  	SPGLVISTGSVCRIVDMPPNALSSMHRTVSLDYGVVLEGEVQLELDSGETRLLKRGDVAV
          	:..   ::.: .   . ...: ::   : : . .   ... . . .:..:   . .. : 

MG08785.1 	-------------------------------------------------------
NCU01000.1	-------------------------------------------------------
FG04970.1 	-------------------------------------------------------
AN6743.1  	QRGTNHAWRNVTPPGEDGQAQWARMLYVLLPAKTVEIDGKALGEELGHIGVRSST
          	. .:. :  . :.......:. :       .:.: . ...: ... .  .  :::
```
