# Supplementary material for: Patterns of Intron Gain and Loss in Fungi
Source: PLoS Biol. 2004 Nov 30;2(12):e422. doi: 10.1371/journal.pbio.0020422 (PMC532390; doi:10.1371/journal.pbio.0020422)
Supplement: Table S1 — Also available at http://genes.mit.edu/NielsenEtAl/. (4.3 MB ZIP). [file pbio.0020422.st001.zip › NielsenEtAl/html/1112.html]

AN1319.1.NCU01331.1.MG02467.1.FG10001.1


```
 CLUSTAL W (1.82) Multiple Sequence Alignments - Introns Inserted


Sequence 1: MG02467.1	126 aa
Sequence 2: FG10001.1	124 aa
Sequence 3: NCU01331.1	128 aa
Sequence 4: AN1319.1	126 aa
Alignment Length: 128 aa
Number Identitical Residues: 76 aa
Alignment Score (without introns) 3330


MG02467.1 	MSA-NDSAA1WPLADA-ALTQ0EILDLVQSAAH~YRQLKKGA~NE~ATKT~LSRGVSELV
NCU01331.1	MSGQNESAA~WPKAEDGALVQ~ELLDCVQQASH~YRQLKKGA~NE~ATKS2VSRGTSELV
FG10001.1 	MS---ESAA1WPLADQ-KLEQ~ELLDLVQSSQH1ARQLKKGA0NE~ATKT~LNRGVSELV
AN1319.1  	MAD-DTNAA1WPIADE-ALSQ~QLLDLVQSATH~YRQLKKGA~NE2TTKT~LNRGTSELV
          	*:  . .** ** *:   * * ::** **.: *  ******* ** :**: :.**.****

MG02467.1 	ILAADCEPLAILLHIPLL~AEDKNV~PYVFV0PSKIALGRACGVSRAVIAASITSNEASD
NCU01331.1	ILAADTQPLSIVLHIPLL1CEDKNV~PYVYV~PSKTALGRACGVSRSVIAVSLTSNEASD
FG10001.1 	VLAADTQPLAILLHLPLL~CEDKNV1PYVYV~SSKMHLGRACGVSRAVIAASITSNDASE
AN1319.1  	ILAADTTPLPIILHLPLL~CEDKNV~PYVYV~PSKLALGRATGVSRPVIAASITTNEASD
          	:****  **.*:**:*** .***** ***:* .**  **** ****.***.*:*:*:**:

MG02467.1 	LQGQIRTLRDKVERLAI
NCU01331.1	LNSKIRALRDKVERLAM
FG10001.1 	LAGQIRAMRDKVERLAI
AN1319.1  	LMPQIRAIKVQVERLMI
          	*  :**::: :**** :
```
