# Supplementary material for: Patterns of Intron Gain and Loss in Fungi
Source: PLoS Biol. 2004 Nov 30;2(12):e422. doi: 10.1371/journal.pbio.0020422 (PMC532390; doi:10.1371/journal.pbio.0020422)
Supplement: Table S1 — Also available at http://genes.mit.edu/NielsenEtAl/. (4.3 MB ZIP). [file pbio.0020422.st001.zip › NielsenEtAl/html/1113.html]

AN4215.1.NCU01219.1.MG05447.1.FG02097.1


```
 CLUSTAL W (1.82) Multiple Sequence Alignments - Introns Inserted


Sequence 1: AN4215.1	102 aa
Sequence 2: MG05447.1	102 aa
Sequence 3: FG02097.1	106 aa
Sequence 4: NCU01219.1	109 aa
Alignment Length: 111 aa
Number Identitical Residues: 45 aa
Alignment Score (without introns) 2185


MG05447.1 	MSEAAKQKAQKLIDENAVM~VFSKSYCPYCTATKSKLKDIGAKYNVLELDQID1DGSAIQ
NCU01219.1	MSDAATQKAKQLINDNAVV1VFSKSYCPYCSNTKQILDGLNAKYATYELNQES~DGSDVQ
FG02097.1 	MS-QASTKVQQLIDNNSVV1VFSKSYCPYCKQTKKTLDDLNTEYELLELDEVA1DGSALQ
AN4215.1  	MS-SAKVKAQQIIDENGVV1VFSKSYCPYCKASKSLLSELGAKYYALELDTID1DGADLQ
          	**  *. *.:::*::*.*: **********. :*. *. :.::*   **:    **: :*

MG05447.1 	DALQEITGQRSVPNIFIGQKHIGGNSDFQALG------NSE-SLIKAAGAL--
NCU01219.1	DALLKLTGQRTVPNIFIGKQHIGGNSDLEAVVKNGKNGKKIQELLQEAGAL--
FG02097.1 	DALEKISGQRTVPNVYIKQQHIGGNSDLQSLNS----GGKLKNLLKEANALKA
AN4215.1  	NALEEISGQRTVPNIYIAKKHIGGNSDLQGIK------KDLPALLKDAGAL--
          	:** :::***:***::* ::*******::.:        .   *:: *.**
```
