# Supplementary material for: Patterns of Intron Gain and Loss in Fungi
Source: PLoS Biol. 2004 Nov 30;2(12):e422. doi: 10.1371/journal.pbio.0020422 (PMC532390; doi:10.1371/journal.pbio.0020422)
Supplement: Table S1 — Also available at http://genes.mit.edu/NielsenEtAl/. (4.3 MB ZIP). [file pbio.0020422.st001.zip › NielsenEtAl/html/1114.html]

AN5678.1.NCU05830.1.MG11062.1.FG00613.1


```
 CLUSTAL W (1.82) Multiple Sequence Alignments - Introns Inserted


Sequence 1: NCU05830.1	659 aa
Sequence 2: FG00613.1	595 aa
Sequence 3: MG11062.1	620 aa
Sequence 4: AN5678.1	585 aa
Alignment Length: 675 aa
Number Identitical Residues: 249 aa
Alignment Score (without introns) 13105


MG11062.1 	MPS-EGDIEMHSLAAKED---------IMLAQQGSSSNHSMR------REPIYDDNTRGP
NCU05830.1	MAGFDQDIELSAYSHSHGLGISGNGRSGRQRSIAEASSFTLGGASPSPRTKSFADVSPGG
FG00613.1 	MA--PHDVERGSISKMK------------EESLAPGSSFSM-------REPEYADILPPS
AN5678.1  	-------MDKDFLKGNP------------ERSTSSDS------------TPVYGESTSRR
          	       ::                      . .  *               : :     

MG11062.1 	SALR--------------------RWADSFRRDPGIRITPKEVPLYLDRDQVHHHHIHHE
NCU05830.1	SHGRSKSIAAASDGAGGGFHGTMSRFVDSFRRGDGT--INKNLDLDDDNNTSSHGYS---
FG00613.1 	YWG---------------------RFVDGFRR-------DQRSSLFTNDPLGQQ------
AN5678.1  	ASLG-------------------QRVLGSFQRDP-----NAHVTAFAS------------
          	                       .*  ..*:*.        .     .            

MG11062.1 	QYHHPPPPLPPQVPMREHDGERYYDLRS--ATLATVNTGLSRELKGRHLQMIAIGGSIG~
NCU05830.1	----------SHSISRGHGGTRYYDLRPGTGKINTPSSLLARELKGRHLQMIAIGGSIG~
FG00613.1 	-----------EGLGRVHDGAHYYDLQS--AMLETANSGLARELKGRHLQMIAIGGSIG1
AN5678.1  	-----------------FSDGKTYDIEN--ATEKAANSPLHRSLRGRHLQMIAIGGSIG1
          	                 ... : **:.   .   : .: * *.*:************** 

MG11062.1 	TGLFVASGKALATGGPASVLLAYCIIGVMLYCTVHALGELAVVFPVAGSFSAFSTRFLDP
NCU05830.1	TGLFVASGKTLSDGGPASVLIAYLIIGVMLFCTIQALGELAVIFPVAGSFSAFSTRFLDP
FG00613.1 	--------RALADGGPASILLAFTIVGAMLFCTCQALGELAVIFPIAGSFSSWATRFIDP
AN5678.1  	TGLFVSSGRVLATGGPASLLIAYALIGCMLYCAVHALGEMAVIFPVAGSFAHYSTRFIDP
          	:.   ::.:.*: *****:*:*: ::* **:*: :****:**:**:****: ::***:**

MG11062.1 	SWGFAMGWN2-SLHDYTILGSQREKSRVCGNILDGHIG--------------LNLFGVKG
NCU05830.1	SWGFAMGWN2YALQWIGVLPLEVLAGAMTIGYWNKEINKAIFVTIFLVIIFVINLFGAKG
FG00613.1 	SWGFAMGWN2YAMQWLIVLPLEIIAASLTLSYWDESLTRAIFVSAFLVLIIVINMFGVKG
AN5678.1  	AWGFAMGWN~YALQWLAILPLEIVAAAITVDYWESNISSAVWVALFWAAIVSINLFGVKG
          	:********  :::   :*  :   . :  .  :  :  :   :        :*:**.**

MG11062.1 	YGEAEFIFAIVKVTAVIGFI2LLGIVINVGGTTTEGYIGTKYWHNPGAFNNGFKGLCSVF
NCU05830.1	YGEAEFIFAIIKVTAVVGFI2LLGIVINIGGTPTEGYIGGKYWEDPGAFHNGFKGLCSVF
FG00613.1 	YGEAEFIFSIIKVIAVIGFI2LLGIVLNCGGTPDSGYIGGRFWHEPGAFNNGFKGMCNVF
AN5678.1  	YGEAEFVFSSIKVIAVIAFI2ILGVILNCAGGQNGSYIGVKYWHDPGAFHHGFKGLCDVF
          	******:*: :** **:.** :**:::* .*    .*** ::*.:****::****:*.**

MG11062.1 	VQAAFAFAGTELVGLAAAETANPRKSLPTAIKQVFWRITLFYIVALTIVGFLVPYDSKRL
NCU05830.1	VTAAFAFAGTELVGLAAAETANPRKSLPTAIKQVFWRISLFYIVSMTLVGLLVPYNEPRL
FG00613.1 	VTAAFSFAGTELIGLAAAETANPRKSLPTALKQVFWRITLFYIVALTLVGLLVPYNDPRL
AN5678.1  	VNAGFAFAGTELVGLAAAETANPRKSLPTAIKQVFWRITLFYIVSLTLVGLLIPYTEPRL
          	* *.*:******:*****************:*******:*****::*:**:*:** . **

MG11062.1 	L-ARNIADAAASPFVIAIESAGIEVLPSIMNSVILIAVLSVGNSAVFGSSRTLAALADQG
NCU05830.1	LNAQNIADASASPFVIAIESAGATVLPSVMNGVILISVISVGNSAVYGSSRTLAALAELG
FG00613.1 	TKGTSDADAAASPFVIAIEEAGIQVLPSVMNAVILCAVLSVGNSAVFGSSRTLAALANLR
AN5678.1  	INGTSTVDTKASPFVIAIKNAGIEVLDSVMNVVIMIAVLSVGNSAVYGSSRTLAALAEQQ
          	 .. . .*: ********:.**  ** *:** **: :*:*******:**********:  

MG11062.1 	QAPRILAYVDRRGRPLVAILLSMSIGLLAFVADSDIQQELLDWLLAISALSSIFTWGSIC
NCU05830.1	QAPAILAYVDRRGRPIVAILVTMAVGLLAYLGDVPSQKNIFDWLLAISGLSSILTWASTC
FG00613.1 	QAPKILGYVDRKGRPLVAIAIASAFGLIAFLADLPQQGAVLDWLMSISGLSTIITWGSIC
AN5678.1  	QAPRFLGYIDRKGRPLWAICIASAMGLLGFLAATDKQEVAFEWMIAISGLSSIFTWGSIC
          	*** :*.*:**:***: ** :: :.**:.::.    *   ::*:::**.**:*:**.* *

MG11062.1 	LSHIRFRRAWSKKGRSLGDLAFRAQPGLAGSWIGFLLNAIFLVAQFWVGGWPLPPVTGEE
NCU05830.1	LAHIRLRKAWAYNHRSVQDIAFKAQGGVIGSWIGFVLNVLVLVTQIWVSIDPLH-QGDTP
FG00613.1 	VCHIRFRRAWAARGRSVSELPFQSQVGVVGSWIGITLNVLVLIAQFWVGAFPIG-WQD--
AN5678.1  	LAHIRFRRAWKVQGNSLHDLAFRSQPGIIGSWIGFAFNCLVLVAQFWVGFAPID---YET
          	:.***:*:**  . .*: ::.*::* *: *****: :* :.*::*:**.  *:       

MG11062.1 	MTPLSIAGEFFLQCMAIPIVLIMWLGHKLYYRTSLVRTDDMDIDTGRRDFN-LPILMAQE
NCU05830.1	LTTKERVRSFFVSCLAIPVVLICTIVHKWYYKTKWVRIEDMDVDTGRRDFGRLGIIKAQE
FG00613.1 	LTSSQVAQNFFHKWVGAPCVLAFYIFHKLYFRTTFVRTRDMDVDTGRRDFN-VPILVAQE
AN5678.1  	MSASMLARNFFSKYLAAPVVLLFYIPYKLRFKTKILRAKDMDLRTGRRELN-IQWLMKEE
          	::.   . .** . :. * **   : :*  ::*. :*  ***: ****::. :  :  :*

MG11062.1 	YEEKLNWPRWKRLYRFFC
NCU05830.1	KEERMGWPRWKRVYRFMC
FG00613.1 	KQERARWPRWKRYYKFMC
AN5678.1  	RAQQAAWPAWKKIYKFFC
          	  ::  ** **: *:*:*
```
