# Supplementary material for: Patterns of Intron Gain and Loss in Fungi
Source: PLoS Biol. 2004 Nov 30;2(12):e422. doi: 10.1371/journal.pbio.0020422 (PMC532390; doi:10.1371/journal.pbio.0020422)
Supplement: Table S1 — Also available at http://genes.mit.edu/NielsenEtAl/. (4.3 MB ZIP). [file pbio.0020422.st001.zip › NielsenEtAl/html/1115.html]

AN2562.1.NCU05886.1.MG05553.1.FG11567.1


```
 CLUSTAL W (1.82) Multiple Sequence Alignments - Introns Inserted


Sequence 1: NCU05886.1	484 aa
Sequence 2: MG05553.1	481 aa
Sequence 3: FG11567.1	464 aa
Sequence 4: AN2562.1	451 aa
Alignment Length: 508 aa
Number Identitical Residues: 179 aa
Alignment Score (without introns) 10536


MG05553.1 	---------------MSH---HESDQEPPKDQQYPV1KWYRSTFTNVTILGLCNLAAPGI
NCU05886.1	MHVVSSGPPDEFPSGLPHRDTHTSEVEPPKDQQHPV1KWYRSSMFNVTIVGVCSFACPGI
FG11567.1 	---------------MSN---ENSSGHGPR--QYPV1KWYRSTFYNMTVLGLCNLAAPGI
AN2562.1  	---------------MTDEEVATSDSITTP-RKYPV1KWYRSTYFNALILGLCNFFAPGI
          	               :.. .   *.   .   ::** *****:  *  ::*:*.: .***

MG05553.1 	WGAMNSLGAGGAARPELINAANALTFCLMVVSCLFSS~AIVRFIGIKGALIFGT2LGYAP
NCU05886.1	WSAMNSLGAGGAASPNLINAANAITFSLMVLSSYLSS1THH------------S~IGYAP
FG11567.1 	WGAMNSLGAGGAQKPYLVNTANALTFCLMVLSCWGGS~VLVHYIGIKGALIFGT2FGYAP
AN2562.1  	WGAMNSLGGGGASKPYLVNTANALTFCLMVLSCFFGS~VIVKFIGIKWTLIVGT~MGYAP
          	*.******.***  * *:*:***:**.***:*.  .* .     . . :   .: :****

MG05553.1 	YAAGLYTNNRYGNEWFVLLGAALCGISAGVFWMAEAAIAIAYPEPWNRGRAIGYWLTYRL
NCU05886.1	FAAGLYTNNRYGNEWFVLLGAALCGISAGVFWAAEASIAIAYPEPWNRGKALGYWLSFKL
FG11567.1 	YAAGLYTNNRFGNEWLVILGAALCGISAGVFWMAEAAIAIAYPEPWNKGKALGYWLTYRL
AN2562.1  	YAAGIYTQVRYDSDWLTLFGAALCGISAGLFWMAESAIALSYPEPQNQGRFLGFWLSFRV
          	:***:**: *:..:*:.::**********:** **::**::**** *:*: :*:**::::

MG05553.1 	SGQILGGAINLGLNADRNGAGQVSYTVFLVFIAIQSAGPLIAFLLSPPAKVQRKDGKRVQ
NCU05886.1	FAQMLGGAINFGLNANNDRAGQVSYTVFLVFIAIQASGPFIGFLLNSPDNVERKDGKKVD
FG11567.1 	SGQIIGGAINLGLNVDRDEAGQVSYTVFLIFIAIQASGPLFALFLSPPNKVERTDGKKVD
AN2562.1  	GGQIVGGAINLGVNVHRNTAGSVSYVVYYIFIALQAFGPFVGLLLTSPGKVERTDGVPVK
          	 .*::*****:*:*...: **.***.*: :***:*: **:..::*..* :*:*.**  *.

MG05553.1 	LAITQNWWVEIKTTAKLFFTPKFLLLVLWIGQAVFAEAVFFTYIAL1WFSVRSRALASFL
NCU05886.1	LTITQNPWREIKATTKLFFTKKFLLLLLWIGNAVFSESVFFTYLAM~WFSVRSRALGSLM
FG11567.1 	LSIVNNPWLETKKITRLFFTTKFLLIVLYIGAVVFSEAVFFTYLSL1WFTVRSRALGSFV
AN2562.1  	LRIANNVWFEIKAMTKLLLSKKFVLIIPLIWQATFGEAVMFTYNSL~WFSVRARALGSFV
          	* *.:* * * *  ::*::: **:*::  *  ..*.*:*:*** :: **:**:***.*::

MG05553.1 	SGILAVTAGNGLG0YWLDSTRFSLKVRSRASFWVIAIFQGAWWTWATVNVTRFHRTRPTY
NCU05886.1	SGIVPVIAGNILG~YWLDRGSISLKVRSRVAFWTLVIFQGAWWTWATVLVTRFKETKPTY
FG11567.1 	SGIIAVIGGNILG0AWLDRTSVPLKRRTRGAFWVLVVLQGGWWTWATILVTRFNISQPTY
AN2562.1  	SGIMAIVSGNLLG~AFLDS-KISLKLRSRVGFIIVLGLQGAWWLWGTIVVTDFHKTNPVF
          	***:.: .** **  :**   ..** *:* .*  :  :**.** *.*: ** *: :.*.:

MG05553.1 	DWVDDDFG~QGFAVFLLLTIGFQLNYMFF2LDPKLTLHSNPRYFFIHNLSQDEPDIIRYA
NCU05886.1	DWATPGFG~AAFGVFIFLAVSFQLNYLFS2------------YFIVHNIAESDEEIIRYA
FG11567.1 	DWSSPGFG~AAFAIFVFLTFGFQLNYLFL2------------YFVVQNLASDEEEIVRYA
AN2562.1  	DWSDS--G2DSYG---HRDVELRGN----~-----------RYFIVGNLAKDEEEVVRIA
          	**     *  .:.      . :: *                 **.: *::..: :::* *

MG05553.1 	ALLRGTESAWQALS~YGLSSLTIFGEVGGVYMNFGMWAVSIYPAWLVLRHFGVHKS---T
NCU05886.1	ALLRGTESAWQALS~YGLESLTLFTATGGVYMSFVIWAVAIYPAWHLLRHFGVHTSRPLA
FG11567.1 	ALLRGTESAWQAIS~YGTSSIPIMARVGGVYFNLALWAVSILPAWTVIKHFGSVPY----
AN2562.1  	SLLRGMESASQAVS0YGLSSISIMASVGSIYLNFGLWALALFPAWLIIREIGVSLG----
          	:**** *** **:* ** .*:.::  .*.:*:.: :**::: *** :::.:*        

MG05553.1 	GVEAISHSPAGDK------VVDSSVEDHTIKALGGL~
NCU05886.1	GDDSYSASIERVKEKGAISSATSSGDEAGNESTSKA~
FG11567.1 	GSDIDATSTPSSE--------DVKLKGSAKVTSSNE2
AN2562.1  	-DKKVERETRTAR--------EVSGAGAH-------~
          	  .    .    .          .
```
