# Supplementary material for: Patterns of Intron Gain and Loss in Fungi
Source: PLoS Biol. 2004 Nov 30;2(12):e422. doi: 10.1371/journal.pbio.0020422 (PMC532390; doi:10.1371/journal.pbio.0020422)
Supplement: Table S1 — Also available at http://genes.mit.edu/NielsenEtAl/. (4.3 MB ZIP). [file pbio.0020422.st001.zip › NielsenEtAl/html/1116.html]

AN6089.1.NCU01589.1.MG03165.1.FG06246.1


```
 CLUSTAL W (1.82) Multiple Sequence Alignments - Introns Inserted


Sequence 1: NCU01589.1	574 aa
Sequence 2: FG06246.1	587 aa
Sequence 3: MG03165.1	602 aa
Sequence 4: AN6089.1	588 aa
Alignment Length: 604 aa
Number Identitical Residues: 451 aa
Alignment Score (without introns) 17568


MG03165.1 	MQRALSSRTRTSALRSSAAAAKFRPGAGLSQQLRFAHK~VSCASFGGSQRAT0ELKFGVE
NCU01589.1	MQRALTR----ASVGK--AATRLPA-----QQLRFAHK0-------------~ELKFGVE
FG06246.1 	MQRALNTRARASALSS--AATKYRAGS-LSQQVRFAHK0-------------~ELKFGVE
AN6089.1  	MQRALSSRTSVLSAASKRAAFTKPAGLN-LQQQRFAHK0-------------~ELKFGVE
          	*****.  :   :  .. **    .. .  ** *****               *******

MG03165.1 	GRAALLAGVDTLAKAVSTTLGPKGRNVLIESSYGSPKITKD1GVTVAKAITLKDKFENLG
NCU01589.1	GRAALLAGVETLAKAVATTLGPKGRNVLIESSFGSPKITKD1GVTVAKSISLKDKFENLG
FG06246.1 	GRAALLAGVDTLAKAVATTLGPKGRNVLIESSFGSPKITKD1GVTVARAVSLKDKFENLG
AN6089.1  	ARAQLLKGVDTLAKAVTSTLGPKGRNVLIESPYGSPKITKD1GVTVAKAVQLQDKFENLG
          	.** ** **:******::*************.:******** *****::: *:*******

MG03165.1 	AKLLQDVASKTNEVAGDGTTSATVLARAIFSETVKNVAAGCNPMDLRRGIQAAVDAVIEF
NCU01589.1	ARLIQEVAGKTNEVAGDGTTSATVLARAIFSETVKNVAAGCNPMDLRRGIQAAVEAVVEY
FG06246.1 	AKLLQDVASKTNEVAGDGTTTATVLARAIFSETVKNVAAGCNPMDLRRGIQAAVEAVVQF
AN6089.1  	ARLLQDVASKTNELAGDGTTTATVLARAIFSETVKNVAAGCNPMDLRRGIQAAVEAAVDY
          	*:*:*:**.****:******:*********************************:*.:::

MG03165.1 	LHKQKRDITSAEEVAQVATISANGDVHVGKMIANAMEKVGKEGVITVKEGKTLVDELEVT
NCU01589.1	LQANKRDVTTSEEVAQVATISANGDKHIGELIASAMEKVGKEGVITCKEGKTLYDELEVT
FG06246.1 	LQKNKRDITTSAEIAQVATISANGDVHIGQMIANAMEKVGKEGVITCKEGKTVADELEVT
AN6089.1  	LQQNKRDITTGEEIAQVATISANGDTHVGKLISTAMERVGKEGVITVKEGKTLEDELEVT
          	*: :***:*:. *:*********** *:*::*:.***:******** *****: ******

MG03165.1 	EGMRFDRGFVSPYFITDAKAQKVEFEKPLILLSEKKISAVQDIIPALEISTQTRRPLVII
NCU01589.1	EGMRFDRGYVSPYFITDPKSQKVEFEKPLILLSEKKISQASDIIPALEISSQTRRPLVII
FG06246.1 	EGMRFDRGFVSPYFITDTKSQKVEFENPLILLSEKKISAVQDIIPALEVSTQQRRPLVII
AN6089.1  	EGMRFDRGYTSPYFITDAKAQKVEFEKPLILLSEKKISAVQDIIPALEASTTLRRPLVII
          	********:.*******.*:******:*********** ..******* *:  *******

MG03165.1 	AEDIEGEALAVCILNKLRGQLQVAAVKAPGFGDNRKSILGDIAVLTNGTVFTDELDVKLE
NCU01589.1	AEDIDGEALAVCILNKLRGQLQVAAVKAPGFGDNRKSILGDIAVLTNGTVFTDELDVKLE
FG06246.1 	AEDIEGEALAVCILNKLRGQLQVAAVKAPGFGDNRKSILGDLAILTDGTVFTDELDIKLD
AN6089.1  	AEDIEGEALAVCILNKLRGQLQVAAVKAPGFGDNRKSILGDLGVLTNGTVFTDELDIKLE
          	****:************************************:.:**:*********:**:

MG03165.1 	KATIDMLGSTGSITITKEDTIVLNGEGSKDMITQRCEQIRGVMADPTTSEYEKEKLQERL
NCU01589.1	KATPDMLGSTGSITITKDDTIILNGEGSKDAIAQRCEQIRGVMADPSTSEYEKEKLQERL
FG06246.1 	KATPDMLGSTGSITITKEDTIVLNGGGSKDAIAQRCEQIRGVIADPTTSEYEKEKLQERL
AN6089.1  	KLTPDMLGSTGSITITKEDTIILNGEGSKDAIAQRCEQIRGVMADPTTSEYEKEKLQERL
          	* * *************:***:*** **** *:*********:***:*************

MG03165.1 	AKLSGGVAVIKVGGSSEVEVGEKKDRFVDALNATRAAVEEGILPGGGTALLKAASQALGD
NCU01589.1	AKLSGGVAVIKVGGASEVEVGEKKDRFVDALNATRAAVEEGILPGGGTALIKASVHALKN
FG06246.1 	AKLSGGVAVIKVGGSSEVEVGEKKDRFVDALNATRAAVEEGILPGGGTALIKASAQALNE
AN6089.1  	AKLSGGVAVIKVGGASEVEVGEKKDRVVDALNATRAAVEEGILPGGGTALLKAAANGLEN
          	**************:***********.***********************:**: :.* :

MG03165.1 	VKSANFDQQLGVSIIKNAITRPARTIVENAGLEGSVIVGKLMDEFGSDFRKGYDAAKGEY
NCU01589.1	VKPANFDQQLGVTIVRNAITRPAKTIIENAGLEGSVVVGKLTDEFANDFNKGFDSAKAEY
FG06246.1 	VPTANFDQQLGVSIVKNAITRPARTIIENAGLESSVVVGKLTDEHAADFNKGFDSAKGEY
AN6089.1  	VKPANFDQQLGVSIVKSAITRPARTIVENAGLEGSVIVGKLTDEFSKDFNRGFDSAKGEY
          	* .*********:*::.******:**:******.**:**** **.. **.:*:*:**.**

MG03165.1 	VDMIDAGIVDPLKVVRTGLLDASGVASLLGTTEVAIVEAPEEKGP-AGGMGGMGGMGGMG
NCU01589.1	VDMIQAGILDPLKVVRTGLVDASGVASLLGTTEVAIVEAPEEKGP--APMG---GMGGMG
FG06246.1 	VDMINAGILDPFKVVRTGLIDASGVASLLGTTEVAIVDAPEEKGAGGPPMGGMGGMGGMG
AN6089.1  	VDMIAAGIVDPLKVVRTALVDASGVSSLLGTTEVAIVEAPEEKGP--AAPGGMGGMGGMG
          	**** ***:**:*****.*:*****:***********:******.     *. .******

MG03165.1 	GMGGMM-
NCU01589.1	GMGGMM-
FG06246.1 	GMGGMM-
AN6089.1  	GMGGGMF
          	**** *
```
