# Supplementary material for: Patterns of Intron Gain and Loss in Fungi
Source: PLoS Biol. 2004 Nov 30;2(12):e422. doi: 10.1371/journal.pbio.0020422 (PMC532390; doi:10.1371/journal.pbio.0020422)
Supplement: Table S1 — Also available at http://genes.mit.edu/NielsenEtAl/. (4.3 MB ZIP). [file pbio.0020422.st001.zip › NielsenEtAl/html/1117.html]

AN3934.1.NCU08618.1.MG01574.1.FG05625.1


```
 CLUSTAL W (1.82) Multiple Sequence Alignments - Introns Inserted


Sequence 1: MG01574.1	183 aa
Sequence 2: FG05625.1	184 aa
Sequence 3: NCU08618.1	183 aa
Sequence 4: AN3934.1	162 aa
Alignment Length: 184 aa
Number Identitical Residues: 103 aa
Alignment Score (without introns) 5130


MG01574.1 	MAAIIKGLYDWLLRTFW2ATEMDVTMIGLQNAGKTSLLRVLS0GGEFTID2TIPTVGFNM
NCU08618.1	MAGLFKKVYDWLLRTFW2ATEMDVTMIGLQNAGKTSLLRVIS0GGEFTLD2SIPTVGFNL
FG05625.1 	MAGIFQRVYNWLMRMFW2AMEMEVTMVGLQNAGKTSLLRVLA0GGEFTLD2SIPTVGFNM
AN3934.1  	MAGIFRTIYDWLLRMFW2ATEMDVTMIGLQNAGKSSLLRVLA0GGEFTID2---------
          	**.::: :*:**:* ** * **:***:*******:*****:: *****:*          

MG01574.1 	KKVQRGHVTMKCWDMGGQERFRGMWERYCRGVTAIL~FIVDVADFDQIPTAKQHLHSLMG
NCU08618.1	KRVQRGHVTLKCWDLGGQPRFRQMWERYCRGVNAIV~FIVDIADPRLLPQAKDELHSLMR
FG05625.1 	KKVQRGHVTLKCWDIGGQPRFRTMWERYCRGVSAIV~FIVDIADTPLIPQAKEELHDLMS
AN3934.1  	------------WDLGGQPRFRPMWERYCRGVNAIV2YIVDAADRAALPVATEELHELMN
          	            **:*** *** *********.**: :*** **   :* *.:.**.** 

MG01574.1 	CESLVGIPLLVLGNKSDLPDKLSVDELIDAMDLKSINTREVSCYGISAKEETNLDAVIQW
NCU08618.1	NETLQGIPLLVLGNKSDLPERLSVDELIDAMDLKSIAGREVSCYGISAKEEMNLDAVLQW
FG05625.1 	RKSLEGIPLLILGNKSDLPDKLSVDELIDELDLKNIRGREVCCYGISAKEETNLDAVVEF
AN3934.1  	KPTLDGIPLLVLGNKSDLPNKLSVDDLIEQMNLKSITRREVSCYGISAKEETNLDAVLHW
          	  :* *****:********::****:**: ::**.*  ***.********* *****:.:

MG01574.1 	LMKYSSK-
NCU08618.1	LMRFAGK-
FG05625.1 	LMKYATRP
AN3934.1  	LIARASR-
          	*:  : :
```
