# Supplementary material for: Patterns of Intron Gain and Loss in Fungi
Source: PLoS Biol. 2004 Nov 30;2(12):e422. doi: 10.1371/journal.pbio.0020422 (PMC532390; doi:10.1371/journal.pbio.0020422)
Supplement: Table S1 — Also available at http://genes.mit.edu/NielsenEtAl/. (4.3 MB ZIP). [file pbio.0020422.st001.zip › NielsenEtAl/html/1118.html]

AN0090.1.NCU06267.1.MG05255.1.FG02566.1


```
 CLUSTAL W (1.82) Multiple Sequence Alignments - Introns Inserted


Sequence 1: NCU06267.1	232 aa
Sequence 2: MG05255.1	204 aa
Sequence 3: FG02566.1	198 aa
Sequence 4: AN0090.1	202 aa
Alignment Length: 235 aa
Number Identitical Residues: 41 aa
Alignment Score (without introns) 2780


MG05255.1 	MSSLPDWRDEYLASIKEAEKNSPVNRDLVEAC1SQLQDRVAALEAERDALK---------
NCU06267.1	---MPGWRDEYLSSLMDADQRNPVNRELVDTC1QQLFDRISVLEAEKAALQQQVETYLSS
FG02566.1 	---MPNWRDQYLSGIKDAELNNPVNMELVQTC1SQMADRISALEAEKNGLE---------
AN0090.1  	---MAHWREEYAAALAARDRREKANVAIYNAY1SQLADRTASSMIAVSDLQSDAQRSALS
          	   :. **::* :.:   : .. .*  : ::  .*: ** :        *:.. .    :

MG05255.1 	ASGASAGQS--------------ASDPASQSGDAAQSAVVARLRLDLAEALGTQERLQSR
NCU06267.1	SSSSSSGAANKPRDRDTTKTDAVASDSTTTAPTAADSALLARLRVDLAEALRAKGDFQRR
FG02566.1 	TLVTTNGKT-----------------TARPTEPSTNDPAVAQLKQDLAEALRSKGVAEKR
AN0090.1  	TPVADPRQQ-----------------QPSPASGPSPQDIILAIRADLAEAQRSRSELEEQ
          	:  :                       .  :  .: .  :  :: *****  ::   : :

MG05255.1 	LGLAESELERLRAKTAEDAKTIRTLNTQCVSLSTKVKDRNEELQGKSKLVE0NVQDELIA
NCU06267.1	LNVVEEELVRLRTKTTTDNKTLQTLTAERKTLTIKLRDREEELRAKSKMLA~DVQDELQV
FG02566.1 	LRSSEEELLQLRSKHKTNTRSIRDLTADKNSLTTRLKDREYELREKRKFIE0QVQDEMIA
AN0090.1  	LARVTTELEKLRRRNIQNGKRISSMESEITHLQLRLKDRDEELREKAKLLE0GFQDEIAT
          	*     ** :** :   : : :  : ::   *  :::**: **: * *::   .***: .

MG05255.1 	LTLQLNVMEQQKAKIQAENDQLVERWMKRMGQEAEAMNLANEPK----FAKRG----
NCU06267.1	LNTHLDLVEKRRSEMEAENKQLVARFMKRVGQEAEAMNMANDSTSGSGHRSSGSRRR
FG02566.1 	LNLQMSMAEKERDKVKKENKELVDRWMKRMAQEAEAMNLANEPI----FKKGR----
AN0090.1  	FELQLNMAEERSNRLQKENQELIDRWMARMGKEADAMNDAYQFS-------------
          	:  ::.: *:.  .:: **.:*: *:* *:.:**:*** * :
```
