# Supplementary material for: Patterns of Intron Gain and Loss in Fungi
Source: PLoS Biol. 2004 Nov 30;2(12):e422. doi: 10.1371/journal.pbio.0020422 (PMC532390; doi:10.1371/journal.pbio.0020422)
Supplement: Table S1 — Also available at http://genes.mit.edu/NielsenEtAl/. (4.3 MB ZIP). [file pbio.0020422.st001.zip › NielsenEtAl/html/1119.html]

AN8801.1.NCU04803.1.MG09511.1.FG08435.1


```
 CLUSTAL W (1.82) Multiple Sequence Alignments - Introns Inserted


Sequence 1: MG09511.1	315 aa
Sequence 2: FG08435.1	330 aa
Sequence 3: NCU04803.1	330 aa
Sequence 4: AN8801.1	347 aa
Alignment Length: 347 aa
Number Identitical Residues: 192 aa
Alignment Score (without introns) 9046


MG09511.1 	MPFNTELTRKLGIK---------------I1PVVQGGMM~HVG1YASLASAVSNAGGLGI
NCU04803.1	MPLQTELTRRLGIT---------------V1PVIQGGMQ0HVG~TAELASAVSNAGGLGI
FG08435.1 	MPFATELTKRLGIR---------------V1PVVQGGMM~HVG~TADLASAVSNAGGLGL
AN8801.1  	MGFNTALTRALGIKSKATLSSYQQILTSLV~PVVQGGMQ~WVG~YAELAAAVSNAGGLGI
          	* : * **: *** :.:: :: ..  :: : **:****   **  *.**:*********:

MG09511.1 	~ITALIFPTPEGLREEIRKCRTMTDKPFGVNLTLLPSMVPPDYPAYVRVVVEEGIKVVET
NCU04803.1	~ITALIFPEPEGLRQEIRKCKKLTTRPFAVNITLLPALVPPDYEAYAQVVIDEGIKIVET
FG08435.1 	~ITALIFPTPEELRKEIQRCRTLTKNPFGVNITLLPSMVPPNYAAFAQTIIDEGIKVVET
AN8801.1  	0LTALTQPTPEDLRKEIRKCRSMTKNPFGVNLTLLPALVPPDYGAYAQVIIDEGIKIVET
          	 :***  * ** **:**::*:.:* .**.**:****::***:* *:.:.:::****:***

MG09511.1 	AGNSPGPVIAALKKAGIIVLHKCTSIRHAESAVKMGVDFLSIDGFECAG2----------
NCU04803.1	AGNSPGPVISKLKKAGVTILHKCTTIRHAQSAVKLGVDFLSIDGFECAG2HVGESDITNF
FG08435.1 	AGNSPGPVITQLKKAGIIVLHKCTTIRHAQSAVKLGVDFLSIDGFECAG~HVGESDITNF
AN8801.1  	AGNNPGPVIRQLKAANITILHKCTTIRHAKSAVKLGVDFLSIDGFECAG~HVGEHDITNF
          	***.*****  ** *.: :*****:****:****:**************   .. . :. 

MG09511.1 	----RARQKLKVPFIASGGFADGQGLAAALCLGASGINMGTRFMCTVEAPIHNNIKETIV
NCU04803.1	ILLSKARQTLNVPFIASGGFADGQGLAAALMLGACGVNMGTRFLCTVEAPIHHNIKEAIV
FG08435.1 	ILLSKARQTLGVPFIASGGFADGYGLAAALCLGACGINMGTRFMCTVEAPVHIKVKEEIV
AN8801.1  	ILLNRARQDLGVPFIASGGFADGYGLAAALALGAEGINMGTRFMCTVEAPIHQKVKQAIV
          	   .:*** * ************ ****** *** *:******:******:* ::*: **

MG09511.1 	GASEHDTALVLRSFRNTTRLYRNKVTEEAIKAEKGAN-GDFSQLAPLVSGQRGKQVFING
NCU04803.1	KAQETDTALVLRRWRNTTRLFKNKVVRDALKVEKESKTGEFSEIAPYVSGKRGKEVFING
FG08435.1 	RAQETDTTLLLRRWTNTTRLYKNKVAMDALEIEKKSESGEFAEVAPYMSGKRGKEVFITG
AN8801.1  	DAEETDTALVMRRWKNTTRLFSNEVTKQALKVEKESKTGEFAEIAPFVSGKRGREVFLNG
          	 *.* **:*::* : *****: *:*. :*:: ** :::*:*:::** :**:**::**:.*

MG09511.1 	DPDYG0VWTAGQVIGLIHDIPTCDVLLKRIEKEAEDSLKEKLSMTNLD--SKL
NCU04803.1	DPEYG~VWTAGQVMGLINDIPTCKDLIARIEKEAEETIKEKVKLFAAP--SKL
FG08435.1 	DVDFG0VWTTGQVMGLINDIPTCDVLVSRIEKEAETALKERLALLVPE--SKL
AN8801.1  	DVNFG0VWTAGQVIGLIHDIPTCAELLSRIEKEADEALNRSRSLYTATPQSKL
          	* ::* ***:***:***:*****  *: ******: :::.   :    ..***
```
