# Supplementary material for: Patterns of Intron Gain and Loss in Fungi
Source: PLoS Biol. 2004 Nov 30;2(12):e422. doi: 10.1371/journal.pbio.0020422 (PMC532390; doi:10.1371/journal.pbio.0020422)
Supplement: Table S1 — Also available at http://genes.mit.edu/NielsenEtAl/. (4.3 MB ZIP). [file pbio.0020422.st001.zip › NielsenEtAl/html/112.html]

AN3592.1.NCU09265.1.MG01607.1.FG00491.1


```
 CLUSTAL W (1.82) Multiple Sequence Alignments - Introns Inserted


Sequence 1: NCU09265.1	571 aa
Sequence 2: MG01607.1	577 aa
Sequence 3: FG00491.1	562 aa
Sequence 4: AN3592.1	565 aa
Alignment Length: 589 aa
Number Identitical Residues: 296 aa
Alignment Score (without introns) 15115


MG01607.1 	MQYRF-IAAALSTASLFVGTYAAEDAQKVIKDDATAASSAAPSSTSSAAPELSTFK0---
NCU09265.1	MRFN--VAAAAASAALLAGGVSADDAQKVLKDES-SSSTVAEAATSVSPAEIPTFT0---
FG00491.1 	MKLNA-VAAAVS-AAMLTGNVHAEDVK---------------EASPSVPDKLPTFT0---
AN3592.1  	MRLNTALTSALVSSASLMGYAHAEDDS---------------TADATSVVERPTFT~VSP
          	*: . ::::*  ::: : *   *:* .                : .    : .**.  :.

MG01607.1 	-~PTKLKAPFLEQFTEDWETRWKPSHAKKNMKGSGKDEEEWAFVGEWAVEEPTVYKGMEG
NCU09265.1	-~PTKLKAPFLEQFTDGWDARWKPSHAKKETG--PDTEEEWAYVGEWSVEEPVVFNGMEG
FG00491.1 	-~PTNLKADFLEQFTDDWDQRWQPSHAKKDTTG---SEEEWAYVGEWAVEEPVKYKGIDG
AN3592.1  	I0PTSLEAPFLEQFTDDWESRWTPSHAKKEDSK---SEEDWAYVGEWSVEEPTVYKGIDG
          	  **.*:* ******:.*: ** ******:       **:**:****:****. ::*::*

MG01607.1 	DKGLVVKNPAAHHAISAKFPKKIDAKGKPLVVQYEVKLQK1GLECGGAYMKLLRDNKALH
NCU09265.1	DKGLVVKNAAAHHAISAKFPKAIDPKAR-------TSLSN1GLECGGAYLKLLRENKALH
FG00491.1 	DKGLVVKNPAAHHAISAKFPKKIDNKGKTLVVQYEVKLQN1GLECGGAYMKLLRDNKALH
AN3592.1  	DKGLVVKNVAAHHAISAKFPKKIDNKGKTLVVQYEVKPQN1SLVCGGAYMKLLQDNKKAL
          	******** ************ ** *.:.   . ... .: .* *****:***::**   

MG01607.1 	QDEFSNTTPYVIMFGPDKCGHTNK~VHFIFNHKNPKTGEYEEKHLNSPPTARIVKTTELY
NCU09265.1	QDEFSNTTPYVIMFGPDKCGHTNK~VHFIFNHKNPKTGEYEEKHLSAPPTAKIVKTTELY
FG00491.1 	QEEFANTTPYVIMFGPDKCGHTNK~VHFIFNHKNPKTGEYEEKHLESPPSAKITKTTELY
AN3592.1  	ADDFSNTTPYVIMFGPDKCGATNK0VHFIFRHKNPKTGEYEEKHLKAPPAARTSKLSSLY
          	 ::*:*************** *** *****.**************.:**:*:  * :.**

MG01607.1 	TLIVHPNNTFIIQQNGEQVKQGSLLEDFTPAVNPPAEIDDAKDKKPEDWVDEARIADPEA
NCU09265.1	TLIVHPNNTFLIQQDGETVKEGSLLEDFVPSVNPEKEIDDPNDTKPEDWVDQARIPDPDA
FG00491.1 	TLIVHPNNTYAIKQNNEEVKTGSLLEDFSPAVNPPAEIDDADDKKPEDWVDQARIPDPEA
AN3592.1  	TLIVRPDQSFQILIDGAAVKNGTLLEDFNPPVNPEKEIDDPKDKKPDDWVDEAKIPDPDA
          	****:*:::: *  :.  ** *:***** *.***  ****..*.**:****:*:*.**:*

MG01607.1 	KKPEDWDEEAPYEIVDEEATKPEDWLEEEPLSIPDPESKKPEDWDDEEDGDWIAPTVPNP
NCU09265.1	KKPDDWDEEAPYEIVDEEATMPEDWLVDEPQTIPDPEAQKPEDWDDEEDGDWIAPTVPNP
FG00491.1 	KKPEDWDEEAPFEVVDEEATKPEDWLEEEAVTIPDPEAEKPDDWDDEEDGDWIAPTVPNP
AN3592.1  	TKPDDWDEDAPYEIVDESAEKPDDWLEDEPNSIPDPEAEKPEDWDDEEDGDWIPPTVPNP
          	.**:****:**:*:***.*  *:*** :*. :*****::**:***********.******

MG01607.1 	KCGEVSGCGPWTKPMIKNPAYKGKWTAPLIDNPAYKGEWAPRKIKNPDYFEDKTPANFEP
NCU09265.1	KCFEVSGCGPWTKPMKKNPDYKGIWSAPLIDNPAYKGPWAPRKIANPDYYEDKTPANFEP
FG00491.1 	KCADASGCGPWTKPMKRNPDYKGKWTAPYVENPAYKGTWAPRKIKNPNYFEDKTPANFEP
AN3592.1  	KCSEVSGCGPWSPPLIKNPAYKGKWTAPLIDNPAYKGPWAPRKIANPDYFEDKTPSNFEP
          	** :.******: *: :** *** *:** ::****** ****** **:*:*****:****

MG01607.1 	MGA0IGFEIWTMQNDILFDNIYIGHSVEEARKFAEETFFEKHPIEQLLELAEKPK-EEET
NCU09265.1	MGA~IGFEIWTMQNNILFDNIYIGHSVEDAKALADETFFKKHPVEEKLEEAEKPKPEENK
FG00491.1 	MGA~IGFEIWTMQNDILFDNIYIGHSIEDANKLAEETFGVKHPVEKALAEADKPK-QDDK
AN3592.1  	MGA0IGFEIWTMQNDILFDNIYIGHSVEDAEKLRKETFDIKRPIEEAEEEASKPK--KET
          	*** **********:***********:*:*. : .***  *:*:*:    *.***  .:.

MG01607.1 	PKPTGEKTLLEDPVAYAKERLDLFLSIAKDSVPDAIRLVPQVPAAAGAILVTLIALLSAV
NCU09265.1	PSSPSDLKFTEDPVTYIKEKVDLFVTIAKKDPVEAIKFVPEVAGGAAALLVTFIAILASL
FG00491.1 	PRSPSDLNFMEDPVHYITEKLDLFKAIAAQDPIQAIKFVPEVAGGFAAIILAAAGLIAVL
AN3592.1  	KAAGTSVSFKEDPVTFVREKVDHFVGLAKEDPINAVKQVPEVAGGLGALLVTMILIIVGA
          	  .  . .: **** :  *::* *  :* ..  :*:: **:*... .*::::   ::   

MG01607.1 	LGGGS-AAPAAKKVASDVKDKAKDVK---ASAVATGAEVKEGAT-KRATRSQQ-
NCU09265.1	VSGGSSPAPAVKKAAKDVKEKAKDVKDKAAEAVATGTEKAKEAT-KRNTRNQS-
FG00491.1 	FNLGK-SSPAVQKTAEKASNKAKQVKDKAAEASATGAEKVKGEVNKRTTRSQS-
AN3592.1  	IGASS-PAPAPVKKG---KEAAKASKEKASEAVSSAAETAKGGATKRTTRSSAE
          	.. .. .:**  * .   .: **  *..::.* ::.:*  :  ..** **.. .
```
