# Supplementary material for: Patterns of Intron Gain and Loss in Fungi
Source: PLoS Biol. 2004 Nov 30;2(12):e422. doi: 10.1371/journal.pbio.0020422 (PMC532390; doi:10.1371/journal.pbio.0020422)
Supplement: Table S1 — Also available at http://genes.mit.edu/NielsenEtAl/. (4.3 MB ZIP). [file pbio.0020422.st001.zip › NielsenEtAl/html/1120.html]

AN5932.1.NCU07870.1.MG06397.1.FG05421.1


```
 CLUSTAL W (1.82) Multiple Sequence Alignments - Introns Inserted


Sequence 1: NCU07870.1	1473 aa
Sequence 2: FG05421.1	1494 aa
Sequence 3: MG06397.1	1482 aa
Sequence 4: AN5932.1	2019 aa
Alignment Length: 2073 aa
Number Identitical Residues: 709 aa
Alignment Score (without introns) 34812


MG06397.1 	MSSKANKRSKLAELRALRQAGKKTFDTYEVEQEGSLYEEVDEDSYKKIVRERLNQDDFVV
NCU07870.1	MSRRVAKRDKYAELRELRQSGKK--KTYDVGEIDELYEEVDENQYKKIVRDRLNEDDFVV
FG05421.1 	----MSHRARLAELKALRASGKKAFDNYKVADVDDLYDEVDEDGYKKVVRERLNQDDFVV
AN5932.1  	---MASARAKLAELRALRAAGKKRLSTYEVEEQGDIYEEVDDDGYKKIIRNRLDEDDFVV
          	       * : ***: ** :***  ..*.* : ..:*:***:: ***::*:**::*****

MG06397.1 	DDNGEGYADDGREDWDRVP-VYDESDSEDGAPARKKTSKAA1KRARDEEQAKRDANDRDI
NCU07870.1	DDNGEGYADDGREEWDRLP-QYH-SDSEEELGLPGSKPRKS1KKQKEHEDAKRDANDRDI
FG05421.1 	DDNGEGYADDGREDWDRVQ-AYE-SDSEEEAGVRGRPSKAA1KKSRQEEQTKRDASDRDI
AN5932.1  	DDNGEGYADDGREVWNEQTGQYSDESDDDDLPARG---KAA~KRKREEEKQRKEKINNGI
          	************* *:.  . * ....::         : : *: ::.*. :::  :..*

MG06397.1 	SEYFSKGASKTQPKQK0AIKTEDDDRFLADLLGEVDSNVRVPVYRAPKKTDKTLERRKAR
NCU07870.1	TEYFTKGAVKAPPKQK~VIKTEEDEKFLDDLIDQVTAQIPVPVTRVSKKRDRSVERRKVR
FG05421.1 	SEYF-KGANRTQPKPK0AVKTKADDDFLSDLLGEVDSNIPEPVRHISKVERSGGGRRKAR
AN5932.1  	SKYFSSGAAAPTPKPK0PVATAEDDAFLADLLGEVDNNVVSNTVPKHN-VVKSETRRKVR
          	::**:.**  . ** *  : *  *: ** **:.:*  ::   .    :       ***.*

MG06397.1 	ALSPGPDTRLPLHKKVKIVDDRPAAPS---PHEDEDDGFMNMDDEPLPLTAEDVPMSDP-
NCU07870.1	VLSPVREPRPPMAKRAKTVDDRASSPAGDDDLLPDDDYLPAMDDE--PAPVSDYLMSDAI
FG05421.1 	ALSPAPEP---ISKKKKIIDTRMSSPP--PALDDEDNFFPPADDD--LLETADVPMSDP-
AN5932.1  	ILSPPLSEKTRTSKPAIKDENSGPVPP-----VAEEPVLDMDNGDGPLDTNDDIPMSDT-
          	 ***  .      *     :   . *.       ::  :   :.:       *  ***. 

MG06397.1 	APSSPAAKVAERKAQADKKAP---EDVDEDEDMMEVSHAG---AIKASSVNLAASRPIKK
NCU07870.1	MPSSPAAKVATRRTFGRPEPKKNEDDEDEDEDMMEVAHTG---AVTAASVNISASRQIKK
FG05421.1 	APSSPAAKVAQRKAQIKQEPK----DDDDDDDMMEVAHTG---AIATTSVNLTSKRPIKK
AN5932.1  	MPSSPVTKAVERKSAVLIKEE----PEDDDDNMMEVVQATGHDEAKAPSVNISGSRPPPK
          	 ****.:*.. *::    :        *:*::**** :: . .   :.***::..*   *

MG06397.1 	IIKPEPVPEPIKSSPVKAEVSDDVDASSWNSITQELNVVS--SQTETRTVGKMDFKNAVE
NCU07870.1	ILKT--EPVPASSSPARAPAP-EVDAASWNEINQKLNVVSM-SQSEGRSVGKIDYKDAIE
FG05421.1 	IIKADIDPTPASSSPVKPNVA-SVNATSWNALTERLNVVSS-SPAEVKSIGKIDHKDAIE
AN5932.1  	IKKQ-PYATPASSSPVKSAAD--VNAS-WNDVRNKLNVLNSPASTETRAFGKLRPQDVAE
          	* *    . * .***.:. .   *:*: ** : :.***:. .: :* ::.**:  ::. *

MG06397.1 	ADGSLRFFWTDYTEVNGSLCLFGKVLDKKTKAYVSCFIKVDNILRKLYFLPRENRHRDGE
NCU07870.1	EDGSLNMFWTDYTEVNGSLCLFGKVLNKKTKTYVSCFVKVDNILRKLFFLPRKYRVRGGE
FG05421.1 	EDGSLNFFWTDYTELNGSLFLFGKVLNKKTKSYVSCFVKVDNILRKLYFLPREHRMQDGE
AN5932.1  	QDGSLRFFWLDYTEVNGSLCLFGKVKNKQNGSYASAFVKVDSILRKLYFLPREYRHKGGR
          	 ****.:** ****:**** ***** :*:. :*.*.*:***.*****:****: * :.*.

MG06397.1 	DCPDEPVQMMDVYSEVDDIMTKMKVDMHKIKACTRKYAFELPDVPKEGKYLKLLYPYTK~
NCU07870.1	ETT-EEVEMMDVYNEVDEIMTKMNVGMHKIKACTRKYAFELPDVPKESQYLKLLYPYTK~
FG05421.1 	ETG-EEVEMMQVYDEIDTMMTKMNVGMYKIKACTRKYAFELRDVPKEAQYMKLLYPYNK1
AN5932.1  	ETD-EEVDMEDVYHEVDQMMSRLRVGMHKIKACTRKYAFEMPGIPKEAEYLKLLYPYDK~
          	:   * *:* :** *:* :*:::.*.*:************: .:***.:*:****** * 

MG06397.1 	PQLDLARSGETFSRVFGTNTGLFEQFVLLKNIMGPCWLKIGDADFGAIRNASHCRLEVLV
NCU07870.1	PAMEPTHKGETFSHVFGTNTALFEQFVLWKNIMGPCWLKIEDADFTSLRNASHCKLEVLV
FG05421.1 	PEIDPNRPGETYSHVFGSNTSLFEQFVLWKNIMGPCWLKIEDADFDKLKNASHCKLEVVA
AN5932.1  	PALPMDTKGETFSHVFGTNTSLFEQFVLWKNIMGPCWLKIEEADFSAVNNASWCKFECQT
          	* :     ***:*:***:**.******* *********** :***  :.*** *::*  .

MG06397.1 	EHPNQISVMSETTDLPDTPPLTIMSLALRTTFNAKDNKQEILAISARVYENVSLDETTPA
NCU07870.1	EHPNMVSVPDNAESI-EPPPLTLLSLAMRTAFNPKDNKQEILAVSGRVYLDVSLSDTTPP
FG05421.1 	DHPNMVSVLSESDNL-DAPPLTLMSVSLRTAFNEKDNKQEILSISARIYEKVSLADTTPA
AN5932.1  	SKPGLISPVPDSENL-DPPTLTLISLAFRTQLNVKENKQEILVASARVYENISLTETTPP
          	.:*. :*   :: .: :.*.**::*:::** :* *:******  *.*:* .:** :***.

MG06397.1 	EKLPCRTFTLIRPNGAAFPIGFEALTKDKRRIKGLVKTLKQESEMLSFFLAQVDVADPDV
NCU07870.1	DKLASRSFTAVRPYGSAFPIGFETLAKE--RNRGVLKLFKQEHEILNFLLAQIDVVDPDV
FG05421.1 	EKLPCRTFTVIRPHGQSFPLGFDQLAKK--RNRGLIVLKKQEADILAFFLAQVDVADPDV
AN5932.1  	EKLPCKTFTVMRPAGSSYPVNFEAEIRK---QRGTYMLEKSEQMLLSKFLALFERMDPDV
          	:**..::** :** * ::*:.*:   :.    :*     *.*  :*  :** .:  ****

MG06397.1 	IVGHQLEGVDYSILLNRLHERKIHQWSRLGRLRRSEWPSSMGKMGGNVFAERQIMSGRLL
NCU07870.1	ILGHQLEGVDYSILLNRLHEKKTHQWSRLGRLRRSQWPSSIVKMGGNVFAERQIMSGRLL
FG05421.1 	ILGHQLEGVDYSVLLNRLHEKKIPGWSRLGRLRRTQWPASIGKTGGNVFAERQILSGRLM
AN5932.1  	LMGHQLQEVDLSILLSRLKEKKTPGWHRLGRLKRGEWPKNFNKGGG-FFTERHLVAGRLV
          	::****: ** *:**.**:*:*   * *****:* :** .: * ** .*:**::::***:

MG06397.1 	CDLANDAGK~SVMTKCQSWSLTEMCNLYLGGGGANAASRRRDVDNEAALKTWAG-TKDGL
NCU07870.1	CDLANDAGK~SVMTKCQTWSLTEMCSLYLGG-----ESRRRDIDNEVALKTWANADKHGL
FG05421.1 	CDLANDAGK~SVMLKCQSWSLTEMCSLYLSG-----DNRRREFDNEVALKTWAK-EKQGL
AN5932.1  	CDVANDMGK0SLMMKCQSWSLTEMCDLYLGS-----GNGRQDLDIDAALKTWAT-SKDGL
          	**:*** ** *:* ***:*******.***..      . *::.* :.******   *.**

MG06397.1 	MDYLTHIEADTYFIAALALRTQVLPLTKVLTNLAGNSWARTLTGTRAERNEYILLHEFHR
NCU07870.1	MDYISHAETDTYFIAALALRTQILPLTKVLTNLAGNSWARTLTGTRAERNEYILLHEFHR
FG05421.1 	LDYITHMEADTHYIAALALGVQMLPLTKVLTNLAGNSWARTLTGTRAERNEYILLHEFYR
AN5932.1  	VNFVTHCDTDTYFIAALVLKLQMLSLTKVLTNIAGNSWARTLSGTRAERNEYILLHEFYK
          	:::::* ::**::****.*  *:*.*******:*********:***************::

MG06397.1 	NKYICPDKRAFKGRQRAAEEDE-EGQAGETGKKKDKYKGGLVFEPEKGLYDKFVLVMDFN
NCU07870.1	NKYVVPDKQTFKGRQRIEEENA-EEEGGEGGKKKDKYKGGLVFEPEKGLYDKFVLVMDFN
FG05421.1 	NKYICPDKQTFRSRQRAEEAQR-EGETAEG-KKKDKYKGGLVFEPEKGLYDKFVLVMDFN
AN5932.1  	NKYICPDKYSSKLQKAEEIAQEGDEDDATDKKKKDKYKGGLVFEPERGLYDRYVLVMDFN
          	***: *** : : ::     : .: : .   ***************:****::*******

MG06397.1 	SLYPSIIQEYNICFTT~VDRQSR0I~DDEEDA-VPEVPPAELDQGILPRLIATLVSRRRQ
NCU07870.1	SLYPSIIQEYNICFTT0S-----~-~-EDDDA-VPEVP-KEQAQGILPKLIATLVSRRRQ
FG05421.1 	SLYPSIIQEFNICFTT~VDRPD-~T0KEGDDE-VPEVP-TNQDQGILPRLIATLVSRRRQ
AN5932.1  	SLYPSIIQEYNICFTT~VDRTSS0A~ENENDEKVPEVPASDAEQGILPRLIATLVGRRRE
          	*********:******  .  .    .: :* .*****. :  *****:******.***:

MG06397.1 	VKSLMKDKSATPEQLATWDIKQLALKLTANSMYGCLGYTKSRFYARPLAILTTYKGREIL
NCU07870.1	VKSLMKDKNATPEELATWDIKQLALKLTANSMYGCLGYTKSRFYARPLAVLTTYKGREIL
FG05421.1 	VKSLMKDKKATPEELATWDIKQLALKLTANSMYGCLGYTKSRFYARPLAVLTTFKGREIL
AN5932.1  	VKKLMKDKRATPEQLALWDTKQLAFKLTANSMYGCLGYTQSRFYARPLAMLTTFKGREIL
          	**.***** ****:** ** ****:**************:*********:***:******

MG06397.1 	RSTKELAENNSLQVIYGDTDSVMINANVDNVAAAFRVGQDFKRAVNERYRLLEIDIDNVF
NCU07870.1	RSTKELAESNSLQVIYGDTDSVMINANKDNVAEALRVGHEFKKAVNERYRLLEIDIDNVF
FG05421.1 	RSTKELAESNSLQVIYGDTDSVMINANVDNVADAFKVGNEFKKAVNEQYKLLEIDIDNVF
AN5932.1  	RSTKELVESKQLRVIYGDTDSVMINTNMDTISDALKVGEELKTAVNERYKLLEIDIDNVF
          	******.*.:.*:************:* *.:: *::**.::* ****:*:**********

MG06397.1 	RRILLQAKKKYAAINMVEVDGKFVEKMEVKGLDMRRREYCALSKEISARILDEILSGDET
NCU07870.1	RRILLQAKKKYAAINMVEVNGKWVEKMEVKGLDMKRREYCGLSKEISNRILTEILSGDDT
FG05421.1 	RRILLQAKKKYAAINLVEKDGKFIEKMEIKGLDMKRREYCALSKEISQHLLNEILSGDDT
AN5932.1  	RRLLLHAKKKYAAINMTEVDGKYVDKLEVKGLDMKRREYCALSKEASQRLLNEILSGDDQ
          	**:**:*********:.* :**:::*:*:*****:*****.**** * ::* ******: 

MG06397.1 	EVSVTRIHEYLRDIAAKMRAQEIPPQKYIILT~QLGKGPKEYPNGDSMPQVQVALREIAR
NCU07870.1	EVSVQRIHEYLRDISAKMREGAVPVQKYIIST~QLGKAPKDYPNADSMPQVQVALRELAK
FG05421.1 	EVSIARIHEYLNEIAGKMREQSIPVQKYIIHT~QLGKAPQDYPDSNSMPQVQVALREMAK
AN5932.1  	ELVLNRVHDYLRELAGNMREYTIPVQKYVIYT0KLSKRPEEYPNKETMPPAQVALRELAR
          	*: : *:*:**.:::.:**   :* ***:* * :*.* *::**: ::** .******:*:

MG06397.1 	GKTIRKGDVISYIIT-TGS----EPAATRAYTPPDVLKPDSGLSPDVDWYMAKQIFPPVE
NCU07870.1	GKTIRKGDVISYIIT-GDSKTTSEAVAKRAYTPQDVLKAESGLSPDVEWYIGKQIFPPVE
FG05421.1 	GKTVRKGDVIAYVIT-GDS-NSSEPAPKRAYTPGD-LKADSSLLPDVEWYIGKQIFPPVE
AN5932.1  	GKTVRPNDVISYIVTSGDSETSSLPPAKRSYTLQDVIKPDSRLKPDIEFYLLKQIFPPIE
          	***:* .***:*::*: .*..:: . ..*:**  * :*.:* * **:::*: ******:*

MG06397.1 	RLCANIIGTSTAQLAENLGLDPRRYASVSGQNKGFGNGDADAGLEIHPLESQMPDDVRFR
NCU07870.1	RLCANIVGTSTSQLAENLGLDVRRYSSNTNNNS-----SGPQDLEIHPLESQIPDSLRFQ
FG05421.1 	RLCANIVGTSTSQLAEQLGLDIKRYSSFQTQQN-----SSSNDLEIHPLDSQIPDEVRFG
AN5932.1  	RLCAPIPGTDAVRLAECLGLDVRKYQINTTTTN------STQNTEIFPLESQIPDSVRFE
          	**** * **.: :*** **** ::*       .      .  . **.**:**:**.:** 

MG06397.1 	NCSRLELRCRACKATSVFEGLQAVDGEEKQSHVTPSGVACG--SCGATIPPVSVIAQVER
NCU07870.1	SCARLSLRCRKCKTSHPFEGLASPSSV---NNVTPSGIQCP--SCQYVLPMLSVVAQVEA
FG05421.1 	DCTRLSLRCRKCKALSTFEGLAATP-----ERVSQSGILCS--SCGTLVSTLSVVAQMEH
AN5932.1  	SAARLTLTCRRCKERSVFEGLAASI-----HMCKPTGLFCPNSSCGNPISTLTIIAQLES
          	..:** * ** **    **** :          . :*: * .:**   :. ::::**:* 

MG06397.1 	AVRLTTARYYEGWLVCDDTSCGARTTQMSVYGSRCLGPKGLAI-DCMGRMRYEFGERDLY
NCU07870.1	AVRAQTARYYEGWLVCDDSSCGNRTRQISVYGTRCLGSKGLARGECGGRMRYEYTERDIY
FG05421.1 	AIRTQTSRYYEGWLICDDTQCGNRTRQMSVYGSRCLGPKGLAR-DCLGRMRYEYTEKAIY
AN5932.1  	QIRAQTSKYYEGWLVCDESTCGNRTRQISVYGHRCLGPRGQAE-GCLGRMAYEYSEKQMY
          	 :*  *::******:**:: ** ** *:**** ****.:* *   * *** **: *: :*

MG06397.1 	NQLVYFASLFDVERARAGGVKD-----------EAARERLLALAEQNR~VR---------
NCU07870.1	NQLVYFASLWDVDKAKSKAKENNDEDVDAAKLSAEEKERVAIMAEHNR~VR---------
FG05421.1 	TQLLYFASLWDVDKAKTKAAIT--------EMSRPDRENILALAEHNR~VR---------
AN5932.1  	NQLLYFASLWDVDKARAAAEKE---------SNEEKKDSIAALVEFNR0MDDIRLAGNAR
          	.**:*****:**::*:: .             .   :: :  :.* ** : .   :..: 

MG06397.1 	---------FGTVKA-------~---------------------------~---------
NCU07870.1	---------FGTVKG-------~---------------------------~---------
FG05421.1 	---------FGNIKG-------~---------------------------~---------
AN5932.1  	PGIRTSFKAFGKEIAGHLLGIW2GYYVYVRPFITGKVVSAKRALANLEYL0LSPFWRVST
          	..  :: .:**.  ..   .   .      .  :..  ::. : :. .    :.    ::

MG06397.1 	------------------TVDRYLDKC~GRQWVAMDNL----------------------
NCU07870.1	------------------VVEKYLDKC~GRRWVAMDTL----------------------
FG05421.1 	------------------VVDKYLDKC~GRQWVAMDTL----------------------
AN5932.1  	GCNSSSACPHELDGESDNLSGKCFDLA1TTGLTAQNTLGVQDIFVVPADFVEKQIRAGLD
          	...::::.. . ...:..   : :* .     .* :.*. ..    .:.  ...  :. .

MG06397.1 	-----FTKLG~FAAV~A---~-------------------~-------------------
NCU07870.1	-----FAKLG~FVPQ~----~-------------------~-------------------
FG05421.1 	-----FTKLG~FKPL0SDQV1FHAPSIPDDKQQKHRKDPS~SKLDQALIHQIPSGRDAN-
AN5932.1  	DVGADVVKLG1MLSS~ATTI~DVVADALTSYQIPSVVLDP0VMISTSGSQLLPEAAVQGL
          	. .:...*** : .  :       ..   . .       .    . :     ...   . 

MG06397.1 	------------------------------------------------------------
NCU07870.1	------------------------------------------------------------
FG05421.1 	------------------------------------------------------------
AN5932.1  	RTKLLPLTTILTPNIPEAQLLLKDAGQEPTNPEDLQGLIDLAKRVAALGPRAVLLKGGHL
          	 :.  . ::  :.. ..:.   ..:....:.... ..  . :.  :: .. :   ...  

MG06397.1 	------------------------------------------------------------
NCU07870.1	------------------------------------------------------------
FG05421.1 	------------------------------------------------------------
AN5932.1  	PLTKDYTAAKGSDDASRVIDILYAGEEVTQFETEYLVSKNTHGTGCSLASAIAANLALGK
          	. :.. :::..:..::   .   :... :. .:.   :..: .:..: ::: ::. : ..

MG06397.1 	-------------------------------------------~----------------
NCU07870.1	-------------------------------------------~----------------
FG05421.1 	-------------------------------------------~----------------
AN5932.1  	DLKRAVRNGVRFVEAGIKTSYDIGKGSGPINHFHSVYSLPFAP2GRFLEYVLDRPDVQSV
          	. . :  ..    .:. .:: . ...:.. .   :  : . :. .   .   . .. .: 

MG06397.1 	----------------------------------~----------------------~--
NCU07870.1	----------------------------------~----------------------~--
FG05421.1 	----------------------------------~----------------------~--
AN5932.1  	WKRFTEHEFVLGLGSGTLPVERFKEYLVQDYLYL0VQFARSNALASYKAKDMESIAA0SA
          	 .  :. .   . .:.: . .  ..   ..      . : :.: :: .:.. .: :: ::

MG06397.1 	-----------------------------------~------------------------
NCU07870.1	-----------------------------------~------------------------
FG05421.1 	-----------------------------------~------------------------
AN5932.1  	KIVLHIQQETALHIDYCASFGLSKEEMEKVPETTA1CTAYSRYILDVGQSEDWLALQVAL
          	.     ...::   . .:: . :... .. ..::: .:: :    . ..:..  : . : 

MG06397.1 	------------------------------------------------------------
NCU07870.1	------------------------------------------------------------
FG05421.1 	------------------------------------------------------------
AN5932.1  	APCLIGYGAIAQRLHAEEKTLREGNRYWKWIENYVAEDYTEAVRLGSGKKHSAPHSRMRE
          	:..  . .: :.   :...:  ...   .  ..  :.. :.:   .:... ::. :   .

MG06397.1 	-----------------------------------------------------
NCU07870.1	-----------------------------------------------------
FG05421.1 	-----------------------------------------------------
AN5932.1  	HLFQLATDGFPELLETHMRKVSLSRMEELVKIFIKATELEIMFWDMGLGAGHS
          	   . ::.. ..  .:   . : :  ..  .   .::. .    . . .:. :
```
