# Supplementary material for: Patterns of Intron Gain and Loss in Fungi
Source: PLoS Biol. 2004 Nov 30;2(12):e422. doi: 10.1371/journal.pbio.0020422 (PMC532390; doi:10.1371/journal.pbio.0020422)
Supplement: Table S1 — Also available at http://genes.mit.edu/NielsenEtAl/. (4.3 MB ZIP). [file pbio.0020422.st001.zip › NielsenEtAl/html/1121.html]

AN3469.1.NCU02435.1.MG03578.1.FG11626.1


```
 CLUSTAL W (1.82) Multiple Sequence Alignments - Introns Inserted


Sequence 1: NCU02435.1	137 aa
Sequence 2: FG11626.1	137 aa
Sequence 3: MG03578.1	137 aa
Sequence 4: AN3469.1	140 aa
Alignment Length: 140 aa
Number Identitical Residues: 120 aa
Alignment Score (without introns) 4260


MG03578.1 	MPPKAADKKPASKAPATASKAP-EKKDAGKKT--AASGDKKKRTKTRKETYSSYIYKV1L
NCU02435.1	MPPKPADKKPASKAPATASKAP-EKKDAGKKT--AASGDKKKRTKARKETYSSYIYKV1L
FG11626.1 	MAPKAADKKPASKAPATASKAP-EKKDAGKKT--AASGDKKKRSKSRKETYSSYIYKV1L
AN3469.1  	MPPKAAEKKPSTGGKAPAGKAPAEKKEAGKKTAAAASGEKKKRGKTRKETYSSYIYKV1L
          	*.**.*:***:: . *.*.***:***:*****::****:**** *:************ *

MG03578.1 	KQVHPDTGISNRAMSILNSFVND1IFERVATEASKLAAYNKKSTISSREIQTS~VRLILP
NCU02435.1	KQVHPDTGISNRAMSILNSFVND1IFERVATEASKLAAYNKKSTISSREIQTS2VRLILP
FG11626.1 	KQVHPDTGISNRAMSILNSFVND1IFERVASEASKLAAYNKKSTISSREIQTS~VRLILP
AN3469.1  	KQVHPDTGISTRAMSILNSFVND1IFERVATEASKLAAYNKKSTISSREIQTS2VRLILP
          	**********.************ ******:********************** ******

MG03578.1 	GELAKHAVSEGTKAVTKYSSSTK
NCU02435.1	GELAKHAVSEGTKAVTKYSSSTK
FG11626.1 	GELAKHAVSEGTKAVTKYSSSTK
AN3469.1  	GELAKHAVSEGTKAVTKYSSSAK
          	*********************:*
```
