# Supplementary material for: Patterns of Intron Gain and Loss in Fungi
Source: PLoS Biol. 2004 Nov 30;2(12):e422. doi: 10.1371/journal.pbio.0020422 (PMC532390; doi:10.1371/journal.pbio.0020422)
Supplement: Table S1 — Also available at http://genes.mit.edu/NielsenEtAl/. (4.3 MB ZIP). [file pbio.0020422.st001.zip › NielsenEtAl/html/1122.html]

AN3867.1.NCU03632.1.MG06947.1.FG05911.1


```
 CLUSTAL W (1.82) Multiple Sequence Alignments - Introns Inserted


Sequence 1: NCU03632.1	547 aa
Sequence 2: MG06947.1	509 aa
Sequence 3: FG05911.1	521 aa
Sequence 4: AN3867.1	323 aa
Alignment Length: 575 aa
Number Identitical Residues: 108 aa
Alignment Score (without introns) 7563


MG06947.1 	--------------MPPKAKAAPKAKEFEK1P~KEAPPPPRTLNEQSHQRYELAHPLHEA
NCU03632.1	--------------MPPKGKAATKPKATET1S~Q--PSEPTTVLERSQQRYEKTRPFEAA
FG05911.1 	MYQVTNPTIHHNLELSPTPDNATQDQSRPQ~A1AP-ESPPDTIAQRSEQRFFQTNPAEKR
AN3867.1  	------------------------------~-~---------------------------
          	                                                            

MG06947.1 	FQKVGISGLSAPELQLWTSAQYCRATAASSGTAHKKQQQQLGNKAHRETWRTFNEASLPL
NCU03632.1	RQQKGLSKLPKEDQKGWICDQLLHLTNRLPAASR---SSLLGPKASKEIWWTVNGTNSIV
FG05911.1 	RQQVGLSSLTPAEKKTYAHVHLIHPAVNR--------RVPFSNKTEREFWKFVTKEGLPI
AN3867.1  	------------------------------------------------------------
          	                                                            

MG06947.1 	R----VPKKPTCWGKDFAGRDVGELSLDQFRARFEKRARLTCLLAA----RETSSPQ---
NCU03632.1	RGLKHKPKPHTTWGTDRNGHDVGSYTIERFDERFRKRIALTALQVSSRVFRENVEREKRG
FG05911.1 	R----RLPRNYAWGTDRTGRDIGTYSPDELEQRSLKHAKLTSLQIQHRHFLSKREKQ---
AN3867.1  	------------------------------------------------------------
          	                                                            

MG06947.1 	------------SEPELIKEKRRRLEMATLRSELYGELVGSLARDPAWDDVVPIPLNEPD
NCU03632.1	FVDARSGREIIVTEREIDEEKVRRSKMAALKKDLYGAITGKLAESVEWEDVVPIPHEEPE
FG05911.1 	---------HEISAEDITAEKTRRKAMAALKRDLYGEITGTLAKDPEWDDVIPIPQNEPE
AN3867.1  	--------------------------------------MGKYASDSEWASIDPIPLNDGS
          	                                       *. * .  * .: *** :: .

MG06947.1 	N----ALAAIAYPDDYAEA1ISYLRAVMASKEYSPRCLKLTEHIISMNPAHYTVW~LYRF
NCU03632.1	G----ALAAIIYPAEYAEA1MSYLRAVMTTKEYSPRCLRLTEHIIAMNPAHYTVW~LYRA
FG05911.1 	G----ALAQIAYPDDYAEA1VSYLRAVMAADECSPRTLRLTEHVISMNPAHYTVW~LFRF
AN3867.1  	ESGAMPLATIAYSEEYLEA~TSYLRAVMAANEMSDRALKLTEDIISMNPAHYTVW2IYRA
          	 :.: .** * *. :* **  *******::.* * * *:***.:*:********* ::* 

MG06947.1 	SIIKALGLAIPDEIQWLNSVALQHLKNYQIW~HHRHLLIDNYYPKIADDKEQVARLATSE
NCU03632.1	ANIFALGISIPDEIEWLNEVALANLKNYQIW~HHRHLLVEHYYPTISSDPDALAQFAKQE
FG05911.1 	KIISVLKLSIPDEINWLNEVALSNLKNYQIW~NHRQLLMDYYYPIIEEDDQTIRKLARSE
AN3867.1  	KIVFALNKDLLEELEWLNGVSLRYLKNYQIW2HHRQVIMS--------SREHFPSLPPKE
          	  : .*   : :*::*** *:*  ******* :**::::.        . : .  :. .*

MG06947.1 	RDFITTMLAEDTKNYHVWSYRQFLVRRLQAWRDPEERRAVEGLIDDDVRNNSAWSHRFFL
NCU03632.1	RGFLIAILSEDTKNYHVWSYRSWLVGKLGMWEDEEELKSIEKMIDEDVRNNSAWSHRFVL
FG05911.1 	TQFITRMLEEDAKNYHVWSYRQYLVSKLFMWTMG-ELLSTQNHIEEDVRNNSAWSHRFYI
AN3867.1  	MDFLMEMFAQDSKNYHVWTYRHWLVRHFELWDSPRELADVNSLLNSDVRNNSAWNH----
          	  *:  :: :*:******:** :** ::  *    *    :  ::.********.*    

MG06947.1 	AFTDPEQTTAGSHATEADLAVPAA~VIDEELAYAKAKIDLAPQNQSPWNYLRGVLVKGGR
NCU03632.1	VFSNPKYATPGKAATEKDEKVPQE~LVEREVKYAQNKVYLAPQNQSPWNYMRGVLVKGGQ
FG05911.1 	VFSDPTASTPGSGPTDADPRVPAE~TLDREINYCKEKISLAPQNQSPWNYLFAVLAKGAR
AN3867.1  	-----------------------H1LVDEELRYAQDQILRAPENRSPWSYARGILRAASR
          	                          ::.*: *.: ::  **:*:***.*  .:*  ..:

MG06947.1 	KLASVEEFASGFVADLGDAEKEE----VRSTHALDLLAEIYAEKGDKDKAALC--LDRLG
NCU03632.1	PLASVQEFVEEFVNKLGEGEEEEE---VKSTHALDLLAEIYAEKKENEKADLC--LRRLA
FG05911.1 	PLSSLKEFAESFVSALG--EDAEE---VRSSHALDFLAKLYDDEGKREKAELC--LQRLG
AN3867.1  	PLSEWTEFAQKFVVDKRDDQGQIVDVSVKSSHAVEWLADVYADAEENGRAEAVRMLNLLK
          	 *:.  **.. **    . :    . :*:*:**:: **.:* :  .. :*     *  * 

MG06947.1 	DEWDRIRKGYWDWRKKTLTTAV-----~--------------
NCU03632.1	EKWDKIRGGYWEWRRKCLTQSETEKAP~GEQVEKAEEGIAAA
FG05911.1 	EKWDPVREGYWKYRVQLLKSGAKE---~--------------
AN3867.1  	DKYDPIRKNYWNYRIRTIEAEEVPASA2PKSNLPYLLNIMF-
          	:::* :* .**.:* : :       :.  ..      .
```
