# Supplementary material for: Patterns of Intron Gain and Loss in Fungi
Source: PLoS Biol. 2004 Nov 30;2(12):e422. doi: 10.1371/journal.pbio.0020422 (PMC532390; doi:10.1371/journal.pbio.0020422)
Supplement: Table S1 — Also available at http://genes.mit.edu/NielsenEtAl/. (4.3 MB ZIP). [file pbio.0020422.st001.zip › NielsenEtAl/html/1123.html]

AN2154.1.NCU08045.1.MG07110.1.FG05066.1


```
 CLUSTAL W (1.82) Multiple Sequence Alignments - Introns Inserted


Sequence 1: NCU08045.1	965 aa
Sequence 2: MG07110.1	963 aa
Sequence 3: FG05066.1	969 aa
Sequence 4: AN2154.1	921 aa
Alignment Length: 991 aa
Number Identitical Residues: 405 aa
Alignment Score (without introns) 22227


MG07110.1 	MSATTKGGQEPVAPTNIRKRRGVQAEGDTESSTAQDGIGHAPTAQYEQSEDQEQ-AKLRQ
NCU08045.1	MSSSAADPFAARLNSDVRQR------HPTASATSKNVEGTSQQKQQQQQQQSEANAAASR
FG05066.1 	MSTAAD---VPDTSPGLRQR---------QSGVGSTAQQDTQAQLSHDLEPSETINSSEK
AN2154.1  	MDRGLSTG-TNQSGAGLRER-----------AVTSQADLN---VITENPTVTNASNGKDK
          	*.            ..:*:*           .. .           .:    :      :

MG07110.1 	ANKSYGRTPDGTV~FTVPTTHDMVSQLLDPRQPKNLSDVIVLAILGMHILAAYALPSHLK
NCU08045.1	VKKTYGKTPDGTV1FVVPTTHDMVTQLLDPREPKNLSDVAVLAIIALHFLAAYYLPWGVK
FG05066.1 	SSKTYGRTPDGTV1FIVPTTHDMVSQLLDPRQPKNLSDAIVLAILGLHILAAYFLPSGSK
AN2154.1  	AGKTFGRTPDGTV1FTVPQTHDMVSQLLSPSEPKNLSDLVVLTILAGHIFLLWILPSGAK
          	  *::*:****** * ** *****:***.* :******  **:*:. *::  : **   *

MG07110.1 	RPVFAAIFIFWRASYNIGIGILLRVQSQHKLLVVKAKKWGIFEDPASGKNPRPWLYHLLK
NCU08045.1	RPLFAAIFMFWRLAYNVGIGYLLTIQSKYKLLVTWAKRWKLFENPATGKNPRPWLYNLLK
FG05066.1 	RTVFAVVFLFWRACYNIGIGVLLQIQSNHRRLVTWAKRWKLFEHPSTGKNPRPWLYKLLK
AN2154.1  	IPVFAVIYLFWRSCYNAGIGWLLHNQSHHKTLVRWAEKSQIFVNPATGKNPYPQLYHLIK
          	 .:**.:::*** .** *** **  **::: **  *::  :* .*::**** * **:*:*

MG07110.1 	RELEAKIPEDYKLEEAPIEYNTWLLFRRVVDLILMCDFVSYCLFAAVCGSTPDGEAPWAT
NCU08045.1	KELETKIPQDYKFEEAPIEYNTWLTFRRVVDLILMCDFISYCLFAIVCAHKPDGEGLFMC
FG05066.1 	TELETKITEDYEFEKAPLEYNTWLVFRRVVDLILMCDFVSYCLFAMVCGHTPEGENPLIG
AN2154.1  	RELEIKISKDYSFEEAPLEYNTWLVFRRLVDLILMCDFASYCLFAIACSRHPANESVLMT
          	 *** **.:**.:*:**:****** ***:********* ****** .*.  * .*     

MG07110.1 	VARWVMGWVLVGLNLWIKLDAHRVVKDFAWYWGD~FFYLIDQELTFDGVFELAPHPMYSI
NCU08045.1	FARWAAGITLVGFNLWVKLDAHRVVKDYAWYWGD1FFYLIEQELTFDGVFELAPHPMYSI
FG05066.1 	FSRWAVGISLIGFNLWVKLDAHRVVKDFAWYWGD~FFYLIDQDLTFDGVFEMAPHPMYSI
AN2154.1  	VIRWTSGIALVLFNLWVKLDAHRVVKDYAWYWGD~FFYLIDQELTFDGVFEMAPHPMYSV
          	. **. *  *: :***:**********:****** *****:*:********:*******:

MG07110.1 	GYIGYYGISMMAASYDVLFISIIAHLAQLIFLVVVENPHIEKTYNPPPPRS-------AG
NCU08045.1	GYAGYYGISMMAASYDVLFISIIAHAAQFAFLVIVENPHIEKTYNPPQPRVRCE--SEAG
FG05066.1 	GYAGYYGISMMAASYEVLFISILAHLAQFAFLVIVENPHIEKTYNPPPPRKR----TVSG
AN2154.1  	GYAGYYGISLMAASYKVLFISIIAHAAQFAFLVLVENPHIDKTYNPPPPRKRTITEHDAA
          	** ******:*****.******:** **: ***:******:****** **    :.  :.

MG07110.1 	-QMQPL-------DGAKDADSATR---DSPATVHNMLGFKNIDLFRVPDYTTILLCIYLG
NCU08045.1	SQLQEF-------ASEYSVPSTTGRHDNTPLPVHNLIGLKNLDFFRITDVAIVLLCAYLA
FG05066.1 	SQIDTIPADTRSIEGAFDQQTLKPSQKDEPGQVHNLVGLSNLDLFRVPDFAVIVMPFYVA
AN2154.1  	SQRSQSPDTPNAPSVSEENVPNATTFSSPPPAVHNLLGFHNLDLHRITDTSSILVQFLMF
          	:* .  .  . :     .  .     .. *  ***::*: *:*:.*:.* : :::   : 

MG07110.1 	VLSLATPKTEFWQAAFIVHAVLCRLWFSLGLGWILTFQSKEKRFTRHFVKYGESAGEAWR
NCU08045.1	VVTMVTPNTRFYQALFVLHALAWRLWYSAGLGVILTMQSEEKMFTRHFLKYGESVGEAWR
FG05066.1 	ILTLATPSTAAWQAAFVFHALVWRVWYHLGLGLILNQQSKNKMWTRHFLKFGESAGEAWR
AN2154.1  	SLTVLTPSTPWYQFLFVANAAIWRLWYSVGIGYLLNRQSNCKSWTRHFVKYGETPHEAWN
          	 ::: **.*  :*  *: :*   *:*:  *:* :*. **: * :****:*:**:  ***.

MG07110.1 	QWKGLYHVSTVMCHGSFIAACWKMYTVPADWTQEWSILKHVTGTGLVALQIWTAVSIYDE
NCU08045.1	QWKGIYHLSNCLCHASFIAASYKMYEFPADWTYGWALLKHVVGLSLIALQVWTATSIYES
FG05066.1 	QWKGLHHISMIMCNTAFVAACWKMYSPPEDWAYGLVMLKHVLGASLVALQLWTAFSVYDS
AN2154.1  	QWKGTYHLSMVMCYASFISAVWKMYTLPSNWGYGLAILRHVLGAGLISLQIWTSVSIYES
          	**** :*:*  :*  :*::* :***  * :*     :*:** * .*::**:**: *:*:.

MG07110.1 	LGEFGWFFGDFFFNNRTKLTYRSIYRFLNNPERVMGSLGLYGAALITWSRAIFVLALISH
NCU08045.1	LGEFGWFYGDFFFDSKRQLTYTSIYRFLNNPERVFGTAGLWGAALITWSRAIFLMALAGH
FG05066.1 	LGEFGWFCGDFFFDHQAKLTYKSIYRFLNNPDRFFGTAGVWGAALITWSRSIFLMALVTQ
AN2154.1  	LGEFGWFYGDFFFDESPKLTYNGIYRFLNNPERVLGLAGVWGAVLITASGTVAFLAFLSH
          	******* *****:   :*** .********:*.:*  *::**.*** * :: .:*:  :

MG07110.1 	LLTLAFISYVEKPHMQKIYGQSIREEAGLTKFVKRSLPPPVKEWSDSVDKVLDEAKAFVE
NCU08045.1	FLTLAFLAYVEKPHMQKVYGRNLRDDAGVTKFIKRSLPPPVTEWQQSIDKVLDETKHFID
FG05066.1 	ILTVFYISYIERPHMQKIYGRGLRQEAGLTKFIKKSLPPHVKGWQESVDKVLDDTSQFVE
AN2154.1  	ILSLGFIQFVERPHMQKLYGRSLRQDAGLVKSLKRSLPPSLRQLHGSVDKIFDESYEFIE
          	:*:: :: ::*:*****:**:.:*::**:.* :*:**** :     *:**::*::  *::

MG07110.1 	DFVESARPKLAAGVSTIVRDTTALFNTYPARLTISQLNPSLAGLDPKQYSLSLEGTVAQK
NCU08045.1	EFVDAARSRLATGSSTIVKDTSALFNKYPARLTLSKISPDLAGYDPKHYGLSLAGTRVVG
FG05066.1 	DFLDTARPKFASGVKTIVRDTSALFNMAPARLTITRITPDLEGYDPKLYSLSVNGMQTTH
AN2154.1  	EIIDTARPKLAAGVNTFVRDTTALFQKYPARVTISRIDADLAGYDLRDYSLTVEASQLPL
          	:::::**.::*:* .*:*:**:***:  ***:*:::: ..* * * : *.*:: .     

MG07110.1 	AAS-GRSTGKESIHGRFPKDVKTLVLEYGAPIRVKW~TAPANHSKNDWVGLYMVIDNRSR
NCU08045.1	TN--EKATGKESPNARVLKDVKTQAFEYGAPIRVKW~TAPANHSKKDWVGLYMVTDNRSR
FG05066.1 	APIVEKYTGKESLTGRFPKPVRTMAFEYGAPLRVKW~RAPANHSKKDWIGLYMVTDNRSR
AN2154.1  	DEG---DLSKEGDNARTPLDRRGDLENLGFPIRHTR1-----------------------
          	        .**.  .*     :    : * *:* .                         

MG07110.1 	EVTEVPSQGRWVPTCPGVYD-RATDGGIITADKPLPSQP-------DMVQGEMVFEGDRL
NCU08045.1	EVTEVPSLGRWVPTNPGEYD-TTTDQGILVWDQPVEKKSE----DTDLVEGEMVFEGDKL
FG05066.1 	ETTEVSSLGRWAPTNAGSYDSLTADVSILVDEHPVSATNAT---DADLVEGEVIFEGDKL
AN2154.1  	---------RWVAVNEGFYDNLTCERGILISDVVVSTSQGDNGEKHDIATGEVVFSGDKL
          	         **...  * **. : : .*:  :  :      .... *:. **::*.**:*

MG07110.1 	WWTQGTFELRYHHDGSHNVMSVSEPFEIKIGRFDEDLDEEVLATGGGVYERAVESELLPL
NCU08045.1	WWTQGVFEFRYHHGGGHHVMSISEPFEIQIPKFDD--EHMGVDISGEVGERAVEAALLPV
FG05066.1 	WWTQGVFEFRYHHNGHHHAMAISEPFEIRISKFDE--EDVDLGAKG-LYEQAVEAALLPV
AN2154.1  	FWTQGVFEFRYHHNGKHNVMAISRPFEVRIPRFEE-------EDHFDMSQTAVETSLLPV
          	:****.**:****.* *:.*::*.***::* :*::            : : ***: ***:

MG07110.1 	VRNCLDRDPEIAPSTVDEPFGSPVERDSKYAKRVVYAIHHM~FGIEFAPAVVPADGNVRK
NCU08045.1	IRNCLDRDPDIAPSNAEERFGGHVERDGKYARRVVYAIRHM~FGIDFAPAVVLADGNVRR
FG05066.1 	VQNCLDRDPDIAPNQPEEPFGGHVERDTKYAKRIVYAIREM~FGIEFAPPVVAADGSVRK
AN2154.1  	IQNCFDRDPEIAPETPEEQYGSLVERDGKFAKRVVFAVHQM2FGVEFAPEVVRSDGNVRN
          	::**:****:***.  :* :*. **** *:*:*:*:*::.* **::*** ** :**.**.

MG07110.1 	LAWRICNAKQVL0APYSMSKSRGTTTPLGEKFLETL
NCU08045.1	LAWRICHAKEVL0APFSMSHTNGRTTPVDSKFSE--
FG05066.1 	LAWRVCNAKEVL0LLGTLQHVSIERNNYTCASRLSF
AN2154.1  	LAWRICNAKRVL0YVSRWRYDTN-------------
          	****:*:**.**
```
