# Supplementary material for: Patterns of Intron Gain and Loss in Fungi
Source: PLoS Biol. 2004 Nov 30;2(12):e422. doi: 10.1371/journal.pbio.0020422 (PMC532390; doi:10.1371/journal.pbio.0020422)
Supplement: Table S1 — Also available at http://genes.mit.edu/NielsenEtAl/. (4.3 MB ZIP). [file pbio.0020422.st001.zip › NielsenEtAl/html/1124.html]

AN1491.1.NCU00157.1.MG05682.1.FG02532.1


```
 CLUSTAL W (1.82) Multiple Sequence Alignments - Introns Inserted


Sequence 1: NCU00157.1	425 aa
Sequence 2: FG02532.1	450 aa
Sequence 3: MG05682.1	443 aa
Sequence 4: AN1491.1	508 aa
Alignment Length: 513 aa
Number Identitical Residues: 125 aa
Alignment Score (without introns) 7239


MG05682.1 	----------------------------------------------MA~TPHEKLLEYFT
NCU00157.1	-----------------------------------------------M~DDR--KLAFFT
FG02532.1 	-----------------------------------------------M~SESNALLAFFS
AN1491.1  	MEPMLPEAGEAALLSSYAPQASGSMGVDPDPITGLVSTSAAPLVRVEE1APKFELESYIA
          	 ..  ..:..::  :: :..::.: . .... :.  :::::.               :::

MG05682.1 	AMDA~AGAVIV--KG1RTRYDRLMLIGQCSVVLCVDALKVAVAEAKRGKDVARYREAVDL
NCU00157.1	AIED~QGGVIV--KG1RTRFERLFHIGRSSVPLCVDALKAAVQEAKAGSDILRYQMAVNS
FG02532.1 	QQSN~TGGIIV--RG1RTRIDRLIQIGKSSVPLCIDALKLAIVEAKAGSDLTQYAEAWNC
AN1491.1  	NYTA1NSWLIPSQKG~RTRFNRLYLIGTCSSYLAVDALKAAIAEAKSGKDVARYLRAVQA
          	      . :* :.:* *** :**  ** .*  *.:**** *: *** *.*: :*  * : 

MG05682.1 	LRQASPNEPEASLDRKWIDVTEKSNREETKRLEQELKGYKNNLVRESIR0MGNEDLGKHL
NCU00157.1	LFQAAPNEPEALLDKAWMESKEKENRDTTAHLQAELQGYKNNLIKESIR0MGNEDLGKHF
FG02532.1 	LRVAAPQEPEAQLETEWVDRVERENKAETTRLESQLKQYRHNLIKESIR0MGNEDLGQHF
AN1491.1  	LADVAPNEPEATIDSDWVERSQKVVKAETDRLEHELRGYKNNLIKESIR0MGNEELGQHY
          	*  .:*:**** ::  *::  ::  :  * :*: :*: *::**::**** ****:**:* 

MG05682.1 	EEIGNLADAAEAYSRMRPDVSTSKQVADVGKHIIRVALQKRDWTAVNSSLNRIAGLQSQL
NCU00157.1	EAIGDVEAAMDSFWKMRTEVSSTEQLVDLGKLLVRVAIERRDWKSIGNHLKPLNSVNDSD
FG02532.1 	EKTGNLEAAAEAYNRMRQDVTTTKHIIDCGIHLVNVYIAKRDWTMVLNNLGKIVGVQSGD
AN1491.1  	HRIGDLTSAFKAYSRMRDFCTTPSHIASMLFKIINVAIERGDWLNVQSNVHRLRSQGGKP
          	.  *::  * .:: :**   ::..:: .    ::.* : : **  : . :  : .  .  

MG05682.1 	SEDLGFPTYIRVMDGLANLGQERYKEAAAAFLKADHSAPASSYADAMSPNDVAIYGSLLS
NCU00157.1	PKAKALKTYSKIANGIAALGQERYKEAAFCFVEASSGVPPEIYNQIASPNDVAIYGGLLA
FG02532.1 	-EERMYQPYTKLVSGIALLGLKHYKDAANNFLQVDFALPPAQYNHIASPNDIAVYGGLLA
AN1491.1  	EEQAKHQPKISAAMGLSQLHSGSYLEAANSFIATDPSLG-DTFNEVLTSNDVAVYGGLCA
          	 :     .      *:: *    * :**  *: .. .     : .  :.**:*:**.* :

MG05682.1 	LATMDRVAIQTNVLENSSFRSFLELEPHLRRAITQFVNGRYSSCLEILESYRSDYLLDMH
NCU00157.1	LATMDRHELQANLLDNDSFREFLQREPHIRRAITQFVNGRYAACIEILESYRPDYLLDIY
FG02532.1 	LATMERNELQARVLDNQSFRSFLENESHIRKAISLFVNGRYSSCLAILESVRNDYLLDVY
AN1491.1  	LASMDRNELQRRVLDNSSFRNFLELEPHIRRAISFFCNSKFRPCLEILEAYRADYLLDIH
          	**:*:*  :* .:*:*.***.**: *.*:*:**: * *.:: .*: ***: * *****::

MG05682.1 	LQRHIPELFLQIRSKCISQYLLPFSCVTIASLDESFAIDGES-----------IEEELVT
NCU00157.1	LQKHVPKLYADIRTKSIVQYLKPFSCVRLDTMQKAFNGPGPS-----------IEDELFT
FG02532.1 	LQRHISTLYSQIRNKCIVQYFIPFSCVTIESLNKAFASEGES-----------VETELVT
AN1491.1  	LQRHVQVLYNRIRTKSIQQYLIPFNRVSLESMAKIFVLGNPTSQSSQSDSKSAFVQELIS
          	**:*:  *:  **.*.* **: **. * : :: : *   . ::.::.:.:.::.  **.:

MG05682.1 	MISNGILDARINSVDK0LVTATAVKPRIQMQLAALKAAQQYETEAAERLRRMNILAAELE
NCU00157.1	MIKDGKLNARIDAINK0--------------SKALQTLENYEKQALDRIRRMNIMAADLE
FG02532.1 	MIREGDLKAHLDAKNK0LLIAVQPNPRLVMQKQALDVSRQYEQEAKDRLRRMNIIAAGLE
AN1491.1  	LIQDGTLDARIDLEKH~VLVSTQGDKRIEVQEAVLDSLDNYVREAHLRLLRSNIIRAGLE
          	:* :* *.*:::  .:    :   .     .  .*.   :*  :*  *: * **: * **

MG05682.1 	VKSNNKRG-------MTAADTEWEGSSSAMASVF----
NCU00157.1	VKGSRKPG------GMNDIPFS--MTTDDTVSLA----
FG02532.1 	IVGKRQQHSGQAGRGIDEQWYDDARTSGQQSQIEGV--
AN1491.1  	VRPLGEDRR----TKLEERGKKGHSAIGNLLRATGMKQ
          	:    :         :     .   : .      . ..
```
