# Supplementary material for: Patterns of Intron Gain and Loss in Fungi
Source: PLoS Biol. 2004 Nov 30;2(12):e422. doi: 10.1371/journal.pbio.0020422 (PMC532390; doi:10.1371/journal.pbio.0020422)
Supplement: Table S1 — Also available at http://genes.mit.edu/NielsenEtAl/. (4.3 MB ZIP). [file pbio.0020422.st001.zip › NielsenEtAl/html/1125.html]

AN5436.1.NCU05200.1.MG01824.1.FG05095.1


```
 CLUSTAL W (1.82) Multiple Sequence Alignments - Introns Inserted


Sequence 1: NCU05200.1	1026 aa
Sequence 2: MG01824.1	1151 aa
Sequence 3: FG05095.1	1012 aa
Sequence 4: AN5436.1	1458 aa
Alignment Length: 1483 aa
Number Identitical Residues: 555 aa
Alignment Score (without introns) 27361


MG01824.1 	------------------------------------------------------------
NCU05200.1	------------------------------------------------------------
FG05095.1 	------------------------------------------------------------
AN5436.1  	MDPNASGYPMQHNPGYPVPSQGTPQQFPYYPNQMPSFPQSNAPSQPSFGAVPLQPGGAMM
          	 ...::. . . ... . .:..:... .  ... .: ..:.:.:..: .: . ....:  

MG01824.1 	----------~-------------------------------------------------
NCU05200.1	----------~-------------------------------------------------
FG05095.1 	----------~-------------------------------------------------
AN5436.1  	PPAFPQQSPG1PHANFSTPFSQPPVTGPFPQPTTTTPNIPATQAQTFPPNMTSGLTKTMA
          	..: ...:.. . :. ::. :... :.. ...::::.. .::.:.: ... ::. :.: :

MG01824.1 	------------------------------------------------------------
NCU05200.1	------------------------------------------------------------
FG05095.1 	------------------------------------------------------------
AN5436.1  	PVQQPIPAQQAPIQNSAPAVQSPGVQSPAALAREKERVSVLLEINSILLQEAINLQTSGK
          	. ... .:..:. ..::.: .:.. .:.:: : ...  :   . .:   ..: . .::..

MG01824.1 	------------------------------------------------------------
NCU05200.1	------------------------------------------------------------
FG05095.1 	------------------------------------------------------------
AN5436.1  	AGGAPAQQGDSNPSPTESAPDKNIQRSPEYVNCMRRLQANLAYLATVADKGKKSGGVVPP
          	:..:.:....:..:.:.::.... . :..  ..    .:. :  :: :.....:..  ..

MG01824.1 	--------------------------------------------------------~---
NCU05200.1	--------------------------------------------------------~---
FG05095.1 	--------------------------------------------------------~---
AN5436.1  	APAIMTPPPNLPAVNDLYTKLNELFPRTAQGTANLNQSGAQGNGGPNLSPTTENAV2LFG
          	:.:  :.... .: ..  :. ..  . ::..::. ..:.:....... :.::..:    .

MG01824.1 	---------------------------MSVGEN~QTCPIS0VRK0YPPGLGTSPRRFDLA
NCU05200.1	---------------------------------~------~---~---------------
FG05095.1 	---------------------------------~------~---~---------------
AN5436.1  	HIPLVRRTLPGLTPMREGSDASRSTLFMHVSYL0SWGPVQ~KSS1SPPITFSCQRRFSLF
          	  .    : .. :.  ..:.:: ::     .   .  . .   .  ..   :.    .  

MG01824.1 	RPLEAL2SFARTSIYRLSRSNLCAAATTTSLTLTLNRAQPTSSPVPVGSLCLYSSSATTP
NCU05200.1	------~-----------------------------------------------------
FG05095.1 	------~-----------------------------------------------------
AN5436.1  	SRISLS~HIKPALPSLLSRAPFARWRPPIPTQLGRLSAAAATPDCCVTSKASSQSTLSRL
          	   .       :     : :  .   .. .       : .::.     : .  .:: :  

MG01824.1 	ATAAHHAPRDTSAIDTSKMNGPT-SQRAKRKQGSASSLDDRPPKHQRALNGAKQQSTGDN
NCU05200.1	------------------MNGTSPVARSKRKEPPQYSSDGRLAKHHRTNGDIDMSSADAN
FG05095.1 	------------------MNGAASSPRVKRKAP---GSPDLPPPAKRAIN--GKHSPNDN
AN5436.1  	PTDSESNPRGAAPDTTTNMNGDG---VAKRKRNSISAAAERPVKHLKPEN--STLTPGDT
          	.: :   . .::.  ::.***       ***  .  .         :. .     :.. .

MG01824.1 	TP-EEDMYDQGLDDEDLLAQALLPSVGPDTVEWQATIQKVVSNVVSIRFCQTCSFDTDAA
NCU05200.1	TP-TEDFDGH-YEEEPRHVLPLAP--GADTAEWQATIENVVRNVVSIRFCQTCSFDTDPA
FG05095.1 	TPDIVEFDDQSETSGDLHPHAMYIGTPGSLGEWQDTIQKVVRNVVAIRFCQTCSFDTDAA
AN5436.1  	TPANGTVYDVEDNEELGPLMAIGP-TQADSPEWQATIEEVVKSVVSIHFCQTCSFDTELS
          	**    . .    .      .:      .  *** **::** .**:*:*********: :

MG01824.1 	LTSEATGFVVDAERG2LLDTPRPLTSSPIRRYILTNRHVVGSGPFWGYCIFDNHEE~VDA
NCU05200.1	LTSEATGFVVDAERG2---------------YILTNRHVVGSGPFWGYCIFDNHEE~VDA
FG05095.1 	LTSEATGYVVDSERG2---------------YILTNRHVVGAGPFWGHCVFDNHEE~VDC
AN5436.1  	MSSQATGFVVDAERG~---------------YILTNRHVVCPGPFWGYVIFDNHEE0CDV
          	::*:***:***:***                ********* .*****: :******  * 

MG01824.1 	YPVYRDPVHDFGILKFDPKAIKYMPVAALPLRPDLAKV~GVEIRVVGNDAGEKLSILSGV
NCU05200.1	YPVYRDPVHDFGILKFDPKAIKYMPVAALPLRPDLARV~GIEIRVVGNDAGEKLSILSGV
FG05095.1 	YPVYRDPVHDFGILRYDPKAIKYMHIDGLELRPDLAKV1GTEIRVVGNDAGEKLSILSGI
AN5436.1  	YPVYRDPVHDFGFLKFDPKAIRHMKLRELKLQPDGARV~GSEIRVVGNDAGEKLSILSGV
          	************:*::*****::* :  * *:** *:* * ******************:

MG01824.1 	ISRLDRNAPEYGEGYSDFNTCYY1QASAAASGGSSGSPVVNIDGFAVALQAGGRADGAST
NCU05200.1	ISRLDRNAPEYGDGYSDFNTCYY~QASAAASGGSSGSPVVNKDGFAVALQAGGRADGAST
FG05095.1 	ISRLDRNAPEYGEGYSDFNTCYY~QANAAASGGSSGSPVVNKDGCAVALQAGGRSDGAST
AN5436.1  	ISRLDRNAPEYGDGYSDFNTNYI~QAAAAASGGSSGSPVVNIEGNAIALQAGGRADGAAT
          	************:******* *  ** ************** :* *:*******:***:*

MG01824.1 	DYFLPLDRPLRALQCLQQGKPITRGDIQCQFLLKPFDECRRLGLAPEWEAQMRKAFPKET
NCU05200.1	DYFLPLDRPLRALKCLQEGKPITRGDIQCQFVLKPFDECRRLGLTPEWEAQVRKAFPKET
FG05095.1 	DYFLPLDRPLRALQCIQNGKPVTRGDIQCQFLLKPFDECRRLGLSPEWEAAMRKQFPEET
AN5436.1  	DYFLPLDRPLRALECIRRGEPVTRGTIQTQWILKPFDECRRLGLTPEWEAAVRKAAPTET
          	*************:*::.*:*:*** ** *::************:***** :**  * **

MG01824.1 	NLLVAEIILPEGPSSNKVEEGDVLLKVNEELITEFIRLDDILDSNVGKPVKLLLQRGGED
NCU05200.1	NMLVAEIILPEGPSHKKLEEGDVLIKVNGKLLTQFIPLEETLDSSVGQTVKLMLLRGGEE
FG05095.1 	NMLVAEIVLPQGPSDKKIEEGDVLIKVNGELITQFIRLDDILDSNVGNTIKLHLQRGGED
AN5436.1  	SMLVAEIILPEGPADGKILEGDVLLQVNGELLTRFVRLDDILDSSVGQTVRLLVQRGGQD
          	.:*****:**:**:  *: *****::** :*:*.*: *:: ***.**:.::* : ***::

MG01824.1 	VEVEVDVGDLHSITPDRFVSVAGGSFHSLSYQQARLYGVACKGVYVCEATGSFRFETSDN
NCU05200.1	VEVEIEVGDLHQITPDRFVSVSGGSFHNLSYQQARLYGVACKGVYVCEAGGSFRFDNNEN
FG05095.1 	VEVELEVGDLHKITPDRFVSVAGASFHDLSYQQARLYAVAVQGVYVCESAGSFRFDNTDN
AN5436.1  	VEVECSVGDLHAITPDRFVTVAGGTFHNLSYQQARLYAIATKGVYVCEAAGSFKLENTLS
          	**** .***** *******:*:*.:**.*********.:* :******: ***:::.. .

MG01824.1 	GWILQTIDNKKVPDLETFIQVVKNIPDKARVVVTYKHLRDLHTLNTTIIYVDRHWSSKMK
NCU05200.1	GWIIQSIDQKETPDLDTFIEVMKGIPDKARVVITYKHLRDMHTLHTTVIYVDRHWAKKMK
FG05095.1 	GWIVQTVDHKKVPDLDTFIQVMKAIPDRARVVVTYKHLRDLHTLNTTVAYIDRHWASKMK
AN5436.1  	GWLIDSVDKRKTRNLDEFVEVMKTIPDRSRVVISYRHIRDLHTRGTSIVYIDRHWHPKMR
          	**:::::*:::. :*: *::*:* ***::***::*:*:**:**  *:: *:****  **:

MG01824.1 	LAVRNDDTGLWDFTDLADALPPVPPVPRKASFIQLEHTSHPAVAELVRSFVHVTCTMPMK
NCU05200.1	LAVRNDKTGLWDFSNLSDALPAVAPVPRKASFIQLENTSHPAVADLVKSFVHVSVTMPVK
FG05095.1 	LAVRNDESGVWDFTDLGDPLPPVPPTRRSASFIELEHMPHPGIANLIHSFVHINCTMPLK
AN5436.1  	LAVRNDESGLWDFSDLADPIKAVPPVPRKADFIQLDGVSQPAAAEIVRSFVRVSCTMPLK
          	******.:*:***::*.*.: .*.*. *.*.**:*:  .:*. *::::***::. ***:*

MG01824.1 	LDGFPKNRKWGMGLVIDAEKGLVVISRAIVPYDLCDISITIGESIVVEGKVVFLHPLQNY
NCU05200.1	LDGFPKNRKWGMGLVIDAEKGLVIISRAIVPYDLCDITITIADSIVVEGKVVFLHPLQNY
FG05095.1 	LDGFPKNRRWGMGLVIDAEKGLVLISRAIVPYDLCDITVTIADSIIVEGKVVFLHPLQNY
AN5436.1  	LDGFPQAKKTGFGLVVDADKGLVVVSRAIVPYNLCDINITVADSIIVAAKVVFLHPLQNY
          	*****: :: *:***:**:****::*******:****.:*:.:**:* .***********

MG01824.1 	AIIQYDPKLVDAPVQSAKLSSEEITQGASTYFIGYNRIGRVVHTATTVTEIFAVAIPANS
NCU05200.1	AVIQYDPKLVDAPVRSAKLSSEMISQGASTYFIGYNRIGRIVHTATTVTEMFAVTIPANS
FG05095.1 	AMIQYDPSLVDAPVKSARLSNEVLTQGAKTYFLGYNRIGRVVHGSTSVTEITAVAIPANS
AN5436.1  	CIVQYDPSLVQAPVQSAKLSTEYIKQGQDTIFVGFNQNFRIVVAKTAVTDITTVSIPANA
          	.::****.**:***:**:**.* :.** .* *:*:*:  *:*   *:**:: :*:****:

MG01824.1 	GAPRYRAVNVDAITVDTNLSGQCGSGVLVAPDGTVQALWLTYLGERSPSTHRDEEYHLGL
NCU05200.1	GAPRYRAVNVDAITVDTNLSGQCGSGVLVAQDGTVQALWLTYLGERNPSSHRDEEYHLGL
FG05095.1 	GAPRYRAVNVDAITIDSNLGSTCNSGVLVAPDGTVQALWLSYLGERNPHSSRDEEYYLGL
AN5436.1  	SAPRYRAINLDAVTVDTGLSGQCSNGVLVGEDGVVQALWLNYLGERTPNSHKDVEYHLGF
          	.******:*:**:*:*:.*.. *..****. **.******.*****.* : :* **:**:

MG01824.1 	ATPTLLPVVKQIQQGIVPKLRMLSVEFRSIQMAQARIMGVSEEWIQQVSLANTSHHQLFM
NCU05200.1	ATPTLLPVISQLQQGITPKLRLLSCEFRAIQMSQARIMGVSEEWIQKVSLVNTAHHQLFL
FG05095.1 	GTKTLLPVVESIQKGVNPKLRILSVEFRSIQMSQASVMGVSDEWIKKVTQANRSHHQLFM
AN5436.1  	ATPSLLPVVSKIQQGILPKLRILNMESYVVQMSQARIMGVSDEWIEKVAQANPSRHQLFM
          	.* :****:..:*:*: ****:*. *   :**:** :****:***::*: .* ::****:

MG01824.1 	VTKRTFERD-QDENSGALLEGDILLTLNDKLITRISELDIMYSHEFLGAVIVRETKELKL
NCU05200.1	VTKRTYERN-EPAG-DHLKEGDILLTLNNQLITKISELDVMYSHDYLDAVIVRNTKELHI
FG05095.1 	VSKRTFER---VNQPVSLLEGDIVLTLNGKICTTISDFDLMYSHELLDVVIVRECAELHL
AN5436.1  	VRKVDCPPPEFNSTADSFQEGDIILTLDGQLITRVSELDVMYEKEMLEALIVRNGEEMKI
          	* *      .    .  : ****:***:.:: * :*::*:**.:: * .:***:  *:::

MG01824.1 	KLPTVAADDVETDHAVSFCGAIFHRPHQAVRQQISKLYSEVYVSAR~TRGSPSYQYGLAP
NCU05200.1	KLPTVAADDAETDHAISFCGAILHRPHLAVRQQISKLFSEVYVSAR~TRGSPAYQYGLAP
FG05095.1 	QIPTVSADDIETDHAVSFCGAILHRPHQAVRQQISKLHSEVYVSSR~IRGSPAYQYGVAP
AN5436.1  	QVPTVSTEDLETDRAVVFCGAVLQKPHHAVRQQISKLHSEIYVSAR0SRGSPAYQYGLSP
          	::***:::* ***:*: ****::::** *********.**:***:*  ****:****::*

MG01824.1 	TNFITHVNGKRTPDLKTFLAAVTAIPDNTY1FRLKAVTFDSVPWVVTMKKNEHY~FPTVE
NCU05200.1	TNFVTHVNGKRTPDLKSFLDAVVGIPDNTY1FRLKCMTFDSVPWVVTMKKNEHY~FPTTE
FG05095.1 	TNFITHVNGTPTPDIPSFIAATREIPDHTY1FRLKAVTFDNVPWVITMKKNDHY~FPTME
AN5436.1  	TNFITAVNGVPTPDLDRFVEEVKKIPDNTY~FRLRAVTFDNVPWVVTMKKNDHY0FPMSE
          	***:* ***  ***:  *:  .  ***:** ***:.:***.****:*****:** **  *

MG01824.1 	WIKDSSE-DCGWRRVTYEG-GKAMEGEPSE--GVPAVEEEAGGAVDDDVPMAAVEK
NCU05200.1	LIKDPSEPLTGWRRITYEG-GKKIEGEGHEGVGVAVLGEDQGEGGEGDVDGCC---
FG05095.1 	WIKDDKE-ACGWRRVTYEG-SDAFKGEAID--GVAPVAEDAEME------------
AN5436.1  	YVKDESE-PLGWKSISHDVKGKDGVAADVANLNPDAMDEGLEGGVSDIEPEAV---
          	 :** .*   **: :::: ...   .    . .   : *      ..    .
```
