# Supplementary material for: Patterns of Intron Gain and Loss in Fungi
Source: PLoS Biol. 2004 Nov 30;2(12):e422. doi: 10.1371/journal.pbio.0020422 (PMC532390; doi:10.1371/journal.pbio.0020422)
Supplement: Table S1 — Also available at http://genes.mit.edu/NielsenEtAl/. (4.3 MB ZIP). [file pbio.0020422.st001.zip › NielsenEtAl/html/1126.html]

AN3088.1.NCU01332.1.MG02466.1.FG10002.1


```
 CLUSTAL W (1.82) Multiple Sequence Alignments - Introns Inserted


Sequence 1: NCU01332.1	147 aa
Sequence 2: MG02466.1	156 aa
Sequence 3: FG10002.1	161 aa
Sequence 4: AN3088.1	161 aa
Alignment Length: 162 aa
Number Identitical Residues: 118 aa
Alignment Score (without introns) 4606


MG02466.1 	------~MFQQ~PFFGAMGCTAAIVFTCLGASYGTAKSGVGIAAMGVLRPDLIVKN1IVP
NCU01332.1	------~----~-----MGCTAAIVFTCLGASYGTAKSGVGIAAMGVLRPDLIVKN1IVP
FG10002.1 	MVSELC2PVYS0PFFGAMGCTCAIVFTCLGASYGTAKSGVGIAAMGVLRPDLIVKN1IVP
AN3088.1  	MDLIAD~NLLQ~PFFGSLGCTSAIVFTCFGAAYGTAKAGVGVCSMGVLRPDLIVKN1IVP
          	          . .  .::***.******:**:*****:***:.:************ ***

MG02466.1 	VIMAGIIGIYGLVVSVLISDNLKQDEYALFTGFIQLGAGLAVGLAGLAAG~FAIGIVGDA
NCU01332.1	VIMAGIIGIYGLVVSVLISDALTQDHYALYTGFIQLGAGLAVGLAGLAAG~FAIGIVGDA
FG10002.1 	VIMAGIIGIYGLVVSVLISDGLKQD-LPLFTSFIQFGAGLSVGLAGLAAG~FAIGIVGDA
AN3088.1  	VVMAGIIGIYGLVVSVLIANNLGQK-VPLYTALVQLGAGLAVGLAGLAAG2FAIGIVGDA
          	*:****************:: * *.  .*:*.::*:****:********* *********

MG02466.1 	GVRGTAQQPRLFVGMILILIFAEVLG1LYGLIVALLMNSKATLNTTC
NCU01332.1	GVRGTAQQPRLFVGMILILIFAEVLG1LYGLIVALLMNSKATLNTSC
FG10002.1 	GVRGTAQQPRLFVGMILILIFAEVLG1LYGLIVALLMNSKATVDAVC
AN3088.1  	GVRGTAQQPRLYVGMILILIFAEVLG1LYGLIVALLMNSRATLEASC
          	***********:************** ************:**::: *
```
