# Supplementary material for: Patterns of Intron Gain and Loss in Fungi
Source: PLoS Biol. 2004 Nov 30;2(12):e422. doi: 10.1371/journal.pbio.0020422 (PMC532390; doi:10.1371/journal.pbio.0020422)
Supplement: Table S1 — Also available at http://genes.mit.edu/NielsenEtAl/. (4.3 MB ZIP). [file pbio.0020422.st001.zip › NielsenEtAl/html/1127.html]

AN4060.1.NCU07826.1.MG05026.1.FG02588.1


```
 CLUSTAL W (1.82) Multiple Sequence Alignments - Introns Inserted


Sequence 1: NCU07826.1	149 aa
Sequence 2: MG05026.1	152 aa
Sequence 3: FG02588.1	150 aa
Sequence 4: AN4060.1	148 aa
Alignment Length: 152 aa
Number Identitical Residues: 106 aa
Alignment Score (without introns) 4801


MG05026.1 	MPGGVNVRDVD0AHKFINAYAAFLKRQGKLPIPG1WVDTVKTGPAKEMPPQDIDWFYVRA
NCU07826.1	MPGGVTVRDVE0PHKFVNAYAAFLKRQGKLPVPG1WVDTVKTGPAKEMPPQDIDWFYVRA
FG02588.1 	MAGGVTVRDVD0AQKFITAYSAFLKRQGKLPIPG1WVDTVKTGPAKELPPQDIDWFYVRA
AN4060.1  	-MGGVTVRDVD0AQKFIVAYAAFLKRQGKLPIPG1WVDTVKTSASNELPPQDADWYYVRA
          	  ***.****: .:**: **:**********:** *******..::*:**** **:****

MG05026.1 	ASVARHVYMRKTVGVGRLRKVHGTAKNRGSRPSHHVDASGSVDRKIMQSLEKIGILEQDE
NCU07826.1	ASVARHVYLRKTVGVGRLRRVHGTAKNRGSRPSHHVEASGSVDRKVLQALEKIGVLEHDE
FG02588.1 	ASIARHVYLRKTVGVGRLRKVHGTAKNRGSRPSKHVDASGSVDRKVMQSLEKIGVLEQDE
AN4060.1  	AAVARHIYLRKTVGVGRLRKVHGSTKNRGSRPAHHVDASGAVDRKVLQSLEKIGVLEQDE
          	*::***:*:**********:***::*******::**:***:****::*:*****:**:**

MG05026.1 	EKGGRRITQAGQRDLDR1IAMTVAEAEEEEEEDDE
NCU07826.1	EKGGRRITQQGQRDLDR1IAQTVIEADEEDDE---
FG02588.1 	EKGGRRITQAGQRDLDR~IAQTTAEAEEEEDDE--
AN4060.1  	EKGGRRITQSGQRDLDR~IAKTTVDEEEEDDE---
          	********* ******* ** *. : :**:::
```
