# Supplementary material for: Patterns of Intron Gain and Loss in Fungi
Source: PLoS Biol. 2004 Nov 30;2(12):e422. doi: 10.1371/journal.pbio.0020422 (PMC532390; doi:10.1371/journal.pbio.0020422)
Supplement: Table S1 — Also available at http://genes.mit.edu/NielsenEtAl/. (4.3 MB ZIP). [file pbio.0020422.st001.zip › NielsenEtAl/html/1128.html]

AN4404.1.NCU04306.1.MG00966.1.FG10320.1


```
 CLUSTAL W (1.82) Multiple Sequence Alignments - Introns Inserted


Sequence 1: AN4404.1	448 aa
Sequence 2: FG10320.1	441 aa
Sequence 3: MG00966.1	462 aa
Sequence 4: NCU04306.1	481 aa
Alignment Length: 499 aa
Number Identitical Residues: 238 aa
Alignment Score (without introns) 10968


MG00966.1 	MAAQVPTEELK-KL~KV1AAKSDDPAAANGANGNTANDSDGSDDDDEPEDAGAAAGGEGA
NCU04306.1	MAAQAAPAEELSKL~SV1DETKPAPAAANGNDSDAE-----SGDEEAEEGAAAP--AAGA
FG10320.1 	MAAQVPTEALK-EL~NV1ADGSQKPGANAQSNTDAAGDDHGGDDSEDEADGAAP--AEGA
AN4404.1  	MAAQASEKLEKLDL1NG~QNGESAAGPAKAGQADAGEVEDESDDDADDAGAAADGAANGA
          	****..      .* .     .  ...    : ::   .  ..*.    ...* ... **

MG00966.1 	~KKKKK-RKPRKKKKKNANGQTDPPRIPISQLFPDNSYPKGQEVEYLGENTYRTTNEEKR
NCU04306.1	~AKKKKKRKPKKKKKA-PTSQSEPPRVLVSQLFPNNQYPKGEEVEYVNDNLNRVTNEEKR
FG10320.1 	~AKKKKKRKPKKKKKN-PTSQSDPPRVQVSQLFPNKSYPPGEEVEYKDENNYRTTDEEKR
AN4404.1  	1AKKKKKRKSKKKKKGGAKVQSSPPRVPVSSLFANGQYPEGEIVEYKNENSYRTTNEEKR
          	  ****.**.:**** ... *:.***: :*.**.: .** *: *** .:*  *.*:****

MG00966.1 	HLDSLNSDLLTDYRHGAEAHRQARRWAHKHVKPGMSLTDIANGIEDSVRALVGHSGLEEG
NCU04306.1	HLDNLNQEFLTDYRHAAEVHRQVRQWAQKSIKPGQTLTEIAENIEDSVRALTGHSGLEEG
FG10320.1 	HLDNLNADFLADYREAAEIHRQVRQWTQKNVKPGQTLTSIAEGIEDGVRALTGHSGLEEG
AN4404.1  	YLDRMNNDFLQEYRQGAEVHRQVRQYAQKNIKPGQTLTEIAEGIEDSVRALTGHQGLEEG
          	:** :* ::* :**..** ***.*::::* :*** :**.**:.***.****.**.*****

MG00966.1 	DAIIAGMGFPTGLSINHCAAHYTPNAGNKMVLEHDDVLKVDIGVHVNGRIVDSAFTVAFN
NCU04306.1	DALIAGMGFPTGLSINHCAAHYTPNAGNKMVLQEDDVMKVDFGVHVNGRIVDSAFTVAFN
FG10320.1 	DSLKAGMGFPCGLSLNHCAAHYTPNAGNKMVLQQQDVMKVDFGVHVNGRIVDSAFTMSFD
AN4404.1  	DNIKGGMGFPCGLSINHCAAHYTPNAGNKMVLQQGDVMKVDFGAHINGRIVDSAFTMSFD
          	* : .***** ***:*****************:. **:***:*.*:**********::*:

MG00966.1 	PRYDNLLAAVKDATNTGIR~---EAGIDARLGEIGEAIQETMESYEVEIDGETYPVKPIR
NCU04306.1	PRYDPLLEAVKAATNAGIK~---EAGIDVRVGDIGAAIQEVMESYEVEINGQMLPVKSIR
FG10320.1 	NKYDNLLQAVKEATNAGIR~---EAGIDARVGEIGGVIQETMESFEVEIDGTTYPVKSIR
AN4404.1  	PVYDPLLEAVKDATNTGIR0SLQEAGIDVRMSDIGAAIQETMESYEIELNGTTYPIKPIR
          	  ** ** *** ***:**: : .*****.*:.:** .***.***:*:*::*   *:*.**

MG00966.1 	NLNGHTIDRYTIHGGKSVPIVKSADQTKMEEGEIYAIETFGSTGLGYVRDE~GEVSHYAK
NCU04306.1	NLNGHTINHYSIHGTKSVPIVKSNDQTKMEEGDVFAIETFGSTGNGYVHEE~GEVSHYAK
FG10320.1 	NLTGHNILPYSIHGTKAVPIVKSNDQTKMEEGDVFAIETFGSTGNGYVRDD~METSHYAK
AN4404.1  	NLNGHNIDQHVIHGGKSVPIVKGSDQTKMEEGEVFAIETFGSTGKGYVRED0METSHYAL
          	**.**.*  : *** *:*****. ********:::********* ***:::  *.**** 

MG00966.1 	RADAPNVALRLTSAQKILNVINKNFGTLPFCRRYLDRLGQDKYLLG0LNNLVSNGIVEAY
NCU04306.1	RGDAAKVDLRLSSAKSLLNVITKNFGTLPFCRRYIDRLGQDKYLLG0-------GIVEAY
FG10320.1 	RGDSSHVDLRLSSAKSLLNVINKNFGTLPFCRRYLDRIGQDKYLLG0LNNLVNAGIVEAY
AN4404.1  	VANAPQVPLRLSSAKSLLNVINKNFGTLPWCRRYLDRLGQDKYLLG0LNNLVQSGIVQDY
          	 .::.:* ***:**:.:****.*******:****:**:********  ..  . ***: *

MG00966.1 	PPLVDKKGSYTA-~--------QYEH0LTHIPMAMQYLTSVSTPLEIAPTFDADFERLIS
NCU04306.1	PPLVDKKGSYTAH2WLSTRDGGQPEH~VRSFESQAGPKRSTRTPCRPHKSDPAIHNRTVP
FG10320.1 	PPLCDKKGSYTA-~--------QFEH0TILIRPTVKEVISRGD----------DY-----
AN4404.1  	PPLCDIKGSYTA-~--------QFEH0TIVLRPTVKEVISRGD----------DY-----
          	*** * ******          * **    :        *              .     

MG00966.1 	EGRY-----------------------
NCU04306.1	IQRLKNSTAFQIVNQRRRVTEAITEAE
FG10320.1 	---------------------------
AN4404.1  	---------------------------
          	
```
