# Supplementary material for: Patterns of Intron Gain and Loss in Fungi
Source: PLoS Biol. 2004 Nov 30;2(12):e422. doi: 10.1371/journal.pbio.0020422 (PMC532390; doi:10.1371/journal.pbio.0020422)
Supplement: Table S1 — Also available at http://genes.mit.edu/NielsenEtAl/. (4.3 MB ZIP). [file pbio.0020422.st001.zip › NielsenEtAl/html/1129.html]

AN5493.1.NCU08148.1.MG05467.1.FG10531.1


```
 CLUSTAL W (1.82) Multiple Sequence Alignments - Introns Inserted


Sequence 1: AN5493.1	614 aa
Sequence 2: FG10531.1	647 aa
Sequence 3: NCU08148.1	628 aa
Sequence 4: MG05467.1	662 aa
Alignment Length: 676 aa
Number Identitical Residues: 220 aa
Alignment Score (without introns) 11572


MG05467.1 	MSAPATDDKMAPRRPSAERTATAATETHSVYATPGATTTLGANTTPGAVVANTADHITPA
NCU08148.1	MSS-------ADNRGHAD-LDTIQPQYHDVTRSP----TIDAMSAHGDANN---TEILGS
FG10531.1 	MTVQEHNPDPALEPAHQH---LHEHHHHSPRAAHDNATYTTGTNPDGSIVPPQQHQHRNS
AN5493.1  	-------------MANEN---VLEG--VPPRESPQPQDFIRHSN--GHAPPAISSDNHSS
          	                 .              :          .  *        .   :

MG05467.1 	QANQQHTNAPVYNDEKASLSSRRDSDPKAAPQYMDAE-KGSSDTVPADQHQPEESPSKVK
NCU08148.1	HAHEKPVISSTDSDLKTTIP---------AGQYVDAESQHQVGSVDHAEQQPAKRYTWAW
FG10531.1 	LDEKLAADNKNHVATPPDYS---------DHEKNEAGVIDASAGTNSERSRPIGWRAKIG
AN5493.1  	YAQEAFAEKK---AIPESHP---------LDNSSEP------SGQDVEAGRKAAFKSAAR
          	  .:  .            .           :  :.              :     :   

MG05467.1 	LFYRRHRTVIRLVIEALVFVFFTV2WFVYGIVAHNGIRRSDNYGWLKPMLFYIAVVLRIF
NCU08148.1	FYFN-----FRPYIHAAIWALFTA2WWIYGALIHH---RHDHLGWLKPTLVYLAICARII
FG10531.1 	PIYR----YRRPLTHLVVFCIATA~WWIASLVLHH-----DDKNWVVPFLVWLAITLRLF
AN5493.1  	RYYKS---YFKHVLYAVIWLLFTG2WWIAGLILHR-----YDLGWLVPFLLYLVITLRLI
          	  :.      :      :: : *  *:: . : *.      . .*: * *.::.:  *::

MG05467.1 	FNHAPTSVVMTPLSIVWNNTVVRLYDVIPKKLHQPLAGLGTLGVFLVGTMVSPDEAENTR
NCU08148.1	FCYVPTSIVMRPAKIIWMHTVHRGYSLIPQKLHQPLAALGTLGVFLLGSMIPEETGENTR
FG10531.1 	FWHVPSRYISNAIKFVWAKTAVVVYDMIPAKFRTLVGASVAVAAILVGSFVSEESEDNTR
AN5493.1  	FLYVPISIFTRPVHWAWKSTASRLVSMIPEHFRIPLGALVTIATILVGSFASAEAPGNTR
          	* :.*   .  .    *  *.    .:** :::  :..  ::..:*:*:: . :   ***

MG05467.1 	ANRAISIFGLLVMIAFLTVTSRNWRAIPWHTVIGGMLTQFIIAIFVLRSQAGFDIFEFIS
NCU08148.1	ANRAISIFGLIVMIFLLTVTSRDWRKIPWHTVIGGMLTQFVIAVFVLKTKAGYDIFSFIS
FG10531.1 	ENRAVSLCGMAVFIFILWVTSRDRKAINWRTVIGGMLSQYIIGLFVLRTGVGYDIFSFIG
AN5493.1  	ADRAVSLFGLLVCIFCLWLTSRNRRKVNWHTVVVGMLVQFIVALFVLRTQVGYDIFDFIS
          	 :**:*: *: * *  * :***: : : *:**: *** *:::.:***:: .*:***.**.

MG05467.1 	FLARSLLGFANAGVTFLTDATVPE-KPWFFTGVIPAIIFFVALVQLLFYAGLIQWFIGKF
NCU08148.1	EMARTLLGFAKDGVVFLTDDTVTA-KGWFLTGVVPPIIFFVALVQLCYYLGFIQWFIGKF
FG10531.1 	YRAADLLGFARDGVAFLTNPDVAA-TPNFFFSVIPAIIFFISLVQVLYYIGFIQWFIIKF
AN5493.1  	MLARELLGFAQEGVDFLTTTDFESGHPYFLVTVIPAIIFFVSLVQLLYYTGVLQWAIRKL
          	  *  *****. ** ***   .  .   *:  *:*.****::***: :* *.:** * *:

MG05467.1 	AKFFFWSLRVSGAEAVVASATPFIGQGESAMLVKPFVPYMTKAELHQVMTCGFATIAGSV
NCU08148.1	AVFFFWTLRVSGAEAVVAAATPFIGQGESAMLIRPFVPHLTLAEIHQIMTCGFATIAGSV
FG10531.1 	ATFVFWGLGVSGAEAVVAAATPFIGQGESAMLVRPFVPHMTKAELHQIMTCGFATISGST
AN5493.1  	AVFFFWSMRVSGAEAVVAAASPFIGQGESAMLIKPFIAHCTMAEIHQIMCSGYATIAGSV
          	* *.** : *********:*:***********::**:.: * **:**:* .*:***:**.

MG05467.1 	LVAYIGLGLNRQALVSSCIMSIPASLAVSKMRYPETEESLTAGNAVVPK-EESPASNALE
NCU08148.1	LVAYIGLGLDAQALVSSCIMSIPASLAVSKMRFPETDETLTSGNVVIPEDEEHKASNALH
FG10531.1 	LVGYIGLGLNREALVSSCIMSIPASLAISKMRYPETEETLTAGRVVIPDDDEHKAENALH
AN5493.1  	LVSYLSMGVNAQALISSCVMSIPASLACSKLRWPEEEETLTAGRVIIPEETEDRPANLLD
          	**.*:.:*:: :**:***:******** **:*:** :*:**:*..::*.. *  . * *.

MG05467.1 	AFANGAWLGLKIAGMIIATLLCIIAFVAMIDGILGWIGVYFDIGYFGGEK--LSINLILG
NCU08148.1	AFANGAWLGIKIAGMIIATLLCIIAIVAFINGLLGWWGKYWGLMGQDAKHPMLSLELILG
FG10531.1 	AFANGAWLGIKIAGTIITSLLCIIAFVAFINGILTWIGSYINLRG----DYDLTLQLILG
AN5493.1  	AFSKGAWLGIKIAGMIAATLLCIISLIGLIDGLLTWWGRYLNIN-----NPTLTLDLIVG
          	**::*****:**** * ::*****:::.:*:*:* * * * .:      .  *:::**:*

MG05467.1 	YIFFPVAWLLGVPGQ-------DCLRVARLIGIK0IIQ~NEFVAFLALADQTG--EYASM
NCU08148.1	YLMYPVAWLLGVPKQ-------DLRPVGELIGIK~VII~NEFVAFSSLTNDE---PYKSM
FG10531.1 	YLLFPVSFLLGVSRTNGDNSTGDILPVARLIAQK~IIT0NEYNAFTDLTTKDPTSQYYGM
AN5493.1  	YICYPIAFLLGVSRD------GDLLKVGKLIGLK~LVA0NEFVAYSALQTDP---QYQDL
          	*: :*:::****.        .*   *..**. * ::  **: *:  *  .     * .:

MG05467.1 	SERSKLIATYAIC0GFGNVGSLGTQIGVLSQLAPSRTADVSSVAISALLAGILSTLTSAS
NCU08148.1	SPRSKLIATYAVC0GFGNIGSLGTQIGVLSQLAPGRAGDVSKVAMSALFSGVLSTLTSAS
FG10531.1 	SPRSQLIATYALC~GFGNIGSLGIQIGILSQLAPTRGGDVARLAVSALISGVLATLTSAS
AN5493.1  	SNRSRLIATYALA~GFANIGSLGNQIGVLAQLAPSRGGDVSRVAVSAMLTGAISTFTSAA
          	* **:******:. **.*:**** ***:*:**** * .**: :*:**:::* ::*:***:

MG05467.1 	VAG~MLYTESMGKALEAAAAS
NCU08148.1	VAD1SVRDDIG--LLDTI---
FG10531.1 	VAG~LVVTNQLSDFTTSQ---
AN5493.1  	IAG~LLIQNEEQYMTTATS--
          	:*.  :  :       : :
```
