# Supplementary material for: Patterns of Intron Gain and Loss in Fungi
Source: PLoS Biol. 2004 Nov 30;2(12):e422. doi: 10.1371/journal.pbio.0020422 (PMC532390; doi:10.1371/journal.pbio.0020422)
Supplement: Table S1 — Also available at http://genes.mit.edu/NielsenEtAl/. (4.3 MB ZIP). [file pbio.0020422.st001.zip › NielsenEtAl/html/113.html]

AN8261.1.NCU07580.1.MG04660.2.FG05393.1


```
 CLUSTAL W (1.82) Multiple Sequence Alignments - Introns Inserted


Sequence 1: MG04660.2	330 aa
Sequence 2: FG05393.1	323 aa
Sequence 3: NCU07580.1	337 aa
Sequence 4: AN8261.1	366 aa
Alignment Length: 385 aa
Number Identitical Residues: 235 aa
Alignment Score (without introns) 9871


MG04660.2 	----------------------------------------------MDGKRHLNSFQQLE
NCU07580.1	----------------------------------------------MDGRKHPSSFQQLE
FG05393.1 	----------------------------------------------MDGKRHPSSFQQLE
AN8261.1  	MTSQRPTSSSSSLLDIVANLASPKSLLFNAHHTNPPPSLPSQRAPTMDKSQQPSSFQQLE
          	 ::. .::::::  .  :. ::..:   .:  :....: .:. :.:**  :: .******

MG04660.2 	K~LGEGTYAT0VFKGRNRHTGELVALKEIHLDSEEGTPSTAIREISLMKELKHENIVGLH
NCU07580.1	K~LGEGTYAT0VFKGRNRQTGELVALKEIHLDSEEGTPSTAIREISLMKELKHENIVALH
FG05393.1 	K~LGEGTYAT~VFKGRNRQTGELVALKEIHLDSEEGTPSTAIREISLMKELKHENIVGLH
AN8261.1  	K0LGEGTYAT0VFKGRNRQTGELVALKEIHLDSEEGTPSTAIREISLMKELKHESIVSLY
          	* ******** *******:***********************************.**.*:

MG04660.2 	DVIHTENKLMLVFEHMDGDLKKYMDTKGDRGALQPMVIKSFMYQLLKGIDFCHQNRVLHR
NCU07580.1	DVIHTENKLMLVFEYMDGDLKKFMDTNGERGALKPHVIKSFMHQLLKGIDFCHKNRVLHR
FG05393.1 	DVIHTENKLMLVFEYMDGDLKRYMDTNGERGALKPTTIKSFMYQLLKGIDFCHQNRVLHR
AN8261.1  	DVIHTENKLMLVFEYMDKDLKKYMDTRGDRGQLDQATIKSFMHQLMSGIAFCHDNRVLHR
          	**************:** ***::***.*:** *.  .*****:**:.** ***.******

MG04660.2 	DLKPQNLLINNKGALKLGDFGLARAFGIPVNTFSNEVVTLWYRAPDVLLGSRTYNTSIDI
NCU07580.1	DLKPQNLLINSKGALKLGDFGLARAFGIPVNTFSNEVVTLWYRAPDVLLGSRTYNTSIDI
FG05393.1 	DLKPQNLLINNKGILKLGDFGLARAFGIPVNTFSNEVVTLWYRAPDVLLGSRTYNTSIDI
AN8261.1  	DLKPQNLLINKKGQLKLGDFGLARAFGIPVNTFSNEVVTLWYRAPDVLLGSRTYNTSIDI
          	**********.** **********************************************

MG04660.2 	WSAGCIMAEMFTGRPLFPGTTNEDQIVRIFRIMGTPSERTWPGFSQFPEYKKTFHTYATQ
NCU07580.1	WSAGCIMAEMFTGRPLFPGTTNEDQIVRIFRIMGTPTERTWPGLTSFPEYKPNWQMYATQ
FG05393.1 	WSAGCIMAEMFTGRPLFPGTTNEDQIVRIFRIMGTPTERTWPGITQFPEYKPTFHMYATQ
AN8261.1  	WSAGCIMAELYTGRPLFPGTTNEDQLQKIFRLMGTPSERSWPGISQLPEYRANFHVYATQ
          	*********::**************: :***:****:**:***::.:***: .:: ****

MG04660.2 	DLRNILPQIDATGIDLLGRMLQLRPEMRISAHDALKHPWFNDILMQQH---------QQQ
NCU07580.1	SLSSILPQIDRDGIDLLQRMLQLRPELRISAHDALQHHWFNDLVHQQHHHQAQQSMMQQP
FG05393.1 	DLRNILQTIDPTGIDLLQRMLQLRPELRISAHDALQHAWFNDLLVHPQ---------QQN
AN8261.1  	DLGLILPQIDPLGLDLLNRMLQLRPEMRIDAHGALQHPWFHDLP----------------
          	.*  **  **  *:*** ********:**.**.**:* **:*:                 

MG04660.2 	HAIQAQAQAHQARTQSGST1DTFAGAAV
NCU07580.1	PMMQQQPMMQQHRGYGQPQ~PNYEGY--
FG05393.1 	--LQSQARAYPQAVPSQAF~DTY-----
AN8261.1  	-QLQAQLQQQQMAGYGGMM~PPQQAY--
          	  :* *         .        .
```
