# Supplementary material for: Patterns of Intron Gain and Loss in Fungi
Source: PLoS Biol. 2004 Nov 30;2(12):e422. doi: 10.1371/journal.pbio.0020422 (PMC532390; doi:10.1371/journal.pbio.0020422)
Supplement: Table S1 — Also available at http://genes.mit.edu/NielsenEtAl/. (4.3 MB ZIP). [file pbio.0020422.st001.zip › NielsenEtAl/html/1130.html]

AN8167.1.NCU01395.1.MG04078.1.FG05979.1


```
 CLUSTAL W (1.82) Multiple Sequence Alignments - Introns Inserted


Sequence 1: NCU01395.1	501 aa
Sequence 2: MG04078.1	536 aa
Sequence 3: FG05979.1	552 aa
Sequence 4: AN8167.1	507 aa
Alignment Length: 581 aa
Number Identitical Residues: 141 aa
Alignment Score (without introns) 8370


MG04078.1 	------MKFSTLFVAGNLALITNAFWRMECRARSGLARIDPLISPGEVAQHLHSIHGGGG
NCU01395.1	------MKVSVAILAA--AGGADAFWRMECRGRLGVARLDPLVNYGEPGSHAHSIHGSSG
FG05979.1 	-----MMLKSIAAVSAGLVGSAHAFWRMECPGRVGLARLDPIIDPGTVSKHVHSIHGSSG
AN8167.1  	MPSKMRLDTSLVACLAAFSGVADAFWRLPCRGRSALARMDPLISPGEPSYHVHAVHGSKG
          	 .:.  :  *     .     :.****: * .* .:**:**::. *  . * *::**. *

MG04078.1 	1FGDNATYDDLMASDCTSCAVTQDKSAYWHPSLFFQDATTGQFEIVQQEGGMLA2YYLLF
NCU01395.1	1FSDKSSYDDLLKGDCTSCAVTQDMSAYWTPSLYFQHAD-GSFELVPQVGGMLS~YYFLY
FG05979.1 	1FADTVTTEQLLGADCTSCRVTQDKSSYWHPALYFEDSDTGKFELVPQVGGMLA2YYLLF
AN8167.1  	1FGLTADMATLLDGDCTSCGVKQDKSAYWAPALYFVDNATGDSELVDEVGGMLV2YYLLY
          	 *. .     *: .***** *.** *:** *:*:* .  :*. *:* : ****  **:*:

MG04078.1 	P---NPGEKLIAFPKGFRMIAGDSLRRNYSEALGDVSKPDPEKSIWAKLGQTSQPDLQQR
NCU01395.1	TDAANPDSGIKAFPPGFRMIAGDTNRRNYTISGTSVKDADPQKSEWASLGQTSQLDLAQR
FG05979.1 	G------DNITAFPPDFRMLSGSNDRRTYSFG--DPSKPDPEKSQWEALGQTTQSDLAER
AN8167.1  	G------DNVEAFPEDFRMVAGDPFQRNFTWP-----IPDPPKSEWTG-DQASQAALRQK
          	       . : *** .***::*.  :*.::        .** ** *   .*::*  * ::

MG04078.1 	AIGFNCLNYAKAPEGSLSRHFLPDKAYLDANCADGVRFEQMFPSCWNGVD-VDSPDHRSH
NCU01395.1	ATGFNCLNYGKQPEGTLYRHYMPDKDYLDANCADGIRAEIMFPSCWNGKD-LDSDNHRDH
FG05979.1 	ALGFNCLNYDKTPEGTLYRHYMPDKSYLDANCKDGIRLELMFPSCWKGGDAVDSENHKDH
AN8167.1  	AIGFNCLNYAKAAEPSLGRHFLPEKAYLDEHCTDGVRFEIMFPSCWNGKD-TDSDDHASH
          	* ******* * .* :* **::*:* *** :* **:* * ******:* *  ** :* .*

MG04078.1 	VAYPDLVMTGNCPKSHPKRLISLFYEIIWNTAAFKGRNGRFVISNGDIQG1FGYHADFMT
NCU01395.1	LAYPDLVMNGNCPKGFETKVPSLMYETIWATQNFIGQPGQFVFANGDVQG~FGYHADFMN
FG05979.1 	VAFPDLVMTGTCPKDYPVRLPSLMYEVIWNTAAFTDRNGRFVFANGDTTG1YGYHGDFVM
AN8167.1  	VAYPSLVMDGTCPEGFETRIVSLFFETIWNTYAFKDRDGYFALSTGDPTG~FGYHADFMH
          	:*:*.*** *.**:..  :: **::* ** *  * .: * *.::.**  * :***.**: 

MG04078.1 	GWDVDFLQNAADTCTNPSGRIQDCPLFNIQDDAKAGQCKIK----LPDMMA---NENVTG
NCU01395.1	GWDEDFLQQAVNTCTNLSGRIQDCPIFNIQSEAEQNQCTFDKNPEVSQMLKSSLNEKTTG
FG05979.1 	GWEEDFLQEAVNTCTSETGRIEDCPLFNVVSEEKAKTCEMK----IPSILE---NEDCKG
AN8167.1  	GWESGVLEEAVKTCTNPSGEVEDCPVFELQSELAQKLCSVD----IPDILS---SEDVKK
          	**: ..*::*..***. :*.::***:*:: .:     * ..    :..::    .*. . 

MG04078.1 	PGLPALPGNVPIQVGPQPATDPNPPPAISVPSLPVMSYQPGATPTG-DQYLPGQVFKQSE
NCU01395.1	VIGDSLPGGVKIAYGPEPADAANAGSHTTTVEVPTATYSQGATVTD-GNYMPGGVFKAAK
FG05979.1 	P-LKALPGSNGHSSGEKPDP-------TGLNPAPTLTYAPGQRPSNSASPLPGQIFKVSS
AN8167.1  	V-KGGLPNKIAVEWGPEYAFPIKYVGEESSTAVPTSTAPQSTSDAGLGVSLSSALSDLAG
          	    .**.      * :     .          *. :   .   :.    :.. : . : 

MG04078.1 	GVGAAAVQTPYPDPGDDGAGSVKAESEPAPAAAAPPPPPPPPPPPPSPPAPPPPPPPPVI
NCU01395.1	-FGVPAAES--------AVNSISTTTEALPTITEAP------------------------
FG05979.1 	AYEAPAPGPSSVNTEKPAPIFSKISIPAAPALLPIPTIEAVADVGVVAIESTPSVEAPVP
AN8167.1  	NIFAADAKT---------TEAPTTTSTSTSTWTPTP------------------------
          	   ..   .             .    . .:    *                        

MG04078.1 	TPPPPPPTPVTREESNDGVRTEYITMGNLVQKIIWVEDIKYVT-------QFVD-ETVVA
NCU01395.1	-------TVAAEDDGFTAIRTEYITKGNVVSMVIVKEALEYVT-------VTTTTVTAVQ
FG05979.1 	TTTPVP-EFVPVTDAKSFYSTQYITNGNVVSKILWEEEVVYVTDIKEEVVYVTVTSTTIA
AN8167.1  	-TTSYIESTVTQETVWVEQEIVVMVDENNVPLKTEVGGVDVVSTDYN--TVTRTVSSVVQ
          	 ...     ..            :.  * *        :  *:   .         :.: 

MG04078.1 	T----------------AGGPAPPPLVRRQLRHLQKHARAQ---
NCU01395.1	T----------------VQARQYSHLHRHKVRAANNRA------
FG05979.1 	TPSVGPVPGAAPVAAPPVAAPPAAAPARRRRRGAHLHGHGRPHF
AN8167.1  	VP----------------TAPAAEKRHHDHLAAHKRHQHGHAH-
          	..                 .       : :    : :  . .
```
