# Supplementary material for: Patterns of Intron Gain and Loss in Fungi
Source: PLoS Biol. 2004 Nov 30;2(12):e422. doi: 10.1371/journal.pbio.0020422 (PMC532390; doi:10.1371/journal.pbio.0020422)
Supplement: Table S1 — Also available at http://genes.mit.edu/NielsenEtAl/. (4.3 MB ZIP). [file pbio.0020422.st001.zip › NielsenEtAl/html/1131.html]

AN0559.1.NCU06632.1.MG06097.1.FG09817.1


```
 CLUSTAL W (1.82) Multiple Sequence Alignments - Introns Inserted


Sequence 1: NCU06632.1	181 aa
Sequence 2: FG09817.1	174 aa
Sequence 3: AN0559.1	169 aa
Sequence 4: MG06097.1	188 aa
Alignment Length: 190 aa
Number Identitical Residues: 55 aa
Alignment Score (without introns) 3122


MG06097.1 	-MQQNTMIWTASAAALATGLLA1YAVYFDHQRRTSSEFRRQLRRNERKQARVEKDGAEAA
NCU06632.1	MPSQAVTYTTAAVAAVATGFLA1YAVYFDYKRRNDPEFRRQLRRSARRQARQEKEYAELS
FG09817.1 	-MVQTSALVTASVATAAAAILG1YVAYFDYQRRNQAEFRRNLRRNERKQARAAKEEAEAS
AN0559.1  	--MKTSTLAVASAGTIITGLLA1YAVYFDHKRQTDPEFRKALKRNNRRLARAVKEEAEAQ
          	   :     .*:..:  :.:*. *..***::*:...***: *:*. *: **  *: **  

MG06097.1 	EGQRLELIRQAVDDIKLAGFPTGVEEKEQFFNEQVTIGEALANDP1TKTIECALHFYCAL
NCU06632.1	QQAQRQRIRQMVDEAKEEGFPTTSDEKEAYFLEQVQAGEILGQDP1TKAIDASLAFYKAL
FG09817.1 	TQQQRQSIRSRVQEANEEGFPSGVEEREAFFNEQVMAGEMLSQDP1SKALESALAFYKGL
AN0559.1  	GAQQRENIKKALQQAKEEGFPTDLEEKEAYFMGQVAKGEGLCSDG1ANKIDAALAFYKAL
          	   : : *:. ::: :  ***:  :*:* :*  **  ** * .*  :: ::.:* ** .*

MG06097.1 	KVYPSPGDLIPIYNSIVAK0PTLDVLAEMIAYDKDLKLTAAS-GPAAAAGKSGPLDDVVD
NCU06632.1	KVYPTPGDLISIYDKTVAK0PILDILAEMIAYDPSLKIGTNYTGGVDVAELMREMASAPG
FG09817.1 	KVYPAPSDLIRIYDSTVPK0PILDILAEMIAFDSSLDVR----GPASPAGIN--LSDIPN
AN0559.1  	KVYPQPKDLISIYDKTVPK~EVLEILAEMVAMDPALKLG-------TFTGES---GGADH
          	**** * *** **:. *.*   *::****:* *  *.:          :       .   

MG06097.1 	VDVDDMPPAAGLD
NCU06632.1	VGLD---------
FG09817.1 	VGLD---------
AN0559.1  	HGVE---------
          	 .::
```
