# Supplementary material for: Patterns of Intron Gain and Loss in Fungi
Source: PLoS Biol. 2004 Nov 30;2(12):e422. doi: 10.1371/journal.pbio.0020422 (PMC532390; doi:10.1371/journal.pbio.0020422)
Supplement: Table S1 — Also available at http://genes.mit.edu/NielsenEtAl/. (4.3 MB ZIP). [file pbio.0020422.st001.zip › NielsenEtAl/html/1132.html]

AN0122.1.NCU08303.1.MG07123.1.FG09725.1


```
 CLUSTAL W (1.82) Multiple Sequence Alignments - Introns Inserted


Sequence 1: NCU08303.1	937 aa
Sequence 2: FG09725.1	938 aa
Sequence 3: MG07123.1	903 aa
Sequence 4: AN0122.1	932 aa
Alignment Length: 950 aa
Number Identitical Residues: 397 aa
Alignment Score (without introns) 19958


MG07123.1 	--MAPSESSLMAIVPLASGSVLLPGVMQRIPVGAKRADIISLVGSLYGRAPAPG---RID
NCU08303.1	MAHIRAPTVTIPLLPLPKQTVLLPGVVQRVAVSSTRPDIASLLAAVYAKAASQTPNGRID
FG09725.1 	--MAKPQTANLPLIPLARGTILLPGLVQRIPVSSNRPDIPALLAHVYEQAASKGPDTRID
AN0122.1  	MGTNGGRPTKLSLVPLPKGSVLLPGATLRIPVS-NRPDLANLLSSLLDRTNAIR--RDAN
          	 .     .  :.::**.  ::****   *:.*. .*.*:  *:. :  :: :       :

MG07123.1 	TIPIACVPIASPLLGANGQRLIRDKAKSDVSTARPRRPVDYSTLQHRDVFSVGVVAKITA
NCU08303.1	TIPIACVPLASPLIGPEGHLLIENGDDKT-ETADD---VDPAKATKADLFPYGVAAKITG
FG09725.1 	SIPIACVPISSPLISGNGQRLIGDAEEIDPAAIEN---VLPGSAKKDDLFTFGVAAKIIG
AN0122.1  	SITFGCVPLCSPYLSKDGQHVIDNG-TVDEDKKEEFESLEAGQARKEDLYRYGTLGKVIG
          	:*.:.***:.** :. :*: :* :             .:  .   : *::  *. .*: .

MG07123.1 	VEAWGSEDAAILVEGVARMTLESLDKDLLRAHYEGHIIQHEE-H~V1PVNDKDLQRRFER
NCU08303.1	VEGRGTGEFTLLVEGVTRIHVEKVISD--KAYLEGKVSSYAD-P~A~LITDAALEELFMS
FG09725.1 	IDGRGTGEFALRVEGTTRVRIENFTRE--RPYFEAKVTYFHE-D~N1NVTDKQAQDLFAL
AN0122.1  	VQRRAYSEPHLLVQGVQRLTVRRVLRE--RPFFEAECILHDEKE1T~PLNDRETAELFQQ
          	::  .  :  : *:*. *: :. .  :  :.. *..   . :.     :.*      *  

MG07123.1 	LKTLARELIDLLRLTSITSRPR--LGLDPVLARRLDSYMAKRTVADAGSFADFVGTLVDC
NCU08303.1	LKLLSRQFVTILRLSSLLPQSSGTPGLSPLLARRLDFYIAKQKY--PGALADFMANIVES
FG09725.1 	LKTRSRELVTILRISSLLPRTRDGPVLSPVLTRRLEMLIMRKELHEAGLLADFMANLVES
AN0122.1  	LRQLSRELLTLLRYTSLIPNTGG-PRLSPLIARKFELIITKSDLAQAGRLADVMADIAES
          	*:  :*::: :** :*: ... .   *.*:::*:::  : :    ..* :**.:. :.:.

MG07123.1 	AYEDKLEILATFDVKQRVDKVIELLDRTIGGLKS---ITRITTVTAAVPMRITDAQNPNL
NCU08303.1	SYEEKLEILTLIDVKERVAKVIELLDRQITNIKNSMRVTTITATTLPFPMDPDAAKHGKI
FG09725.1 	THEEKLEVLAALDVKVRLTKVIELLERQVGGIKNNFKITTFTTMPIQILDRLNENQNRKP
AN0122.1  	GLEDKLRVLAAFDVKTRLERVVDILNKQNQIIRGSVKFTTISTDNIPPASVLDISQIDPR
          	  *:**.:*: :*** *: :*:::*::    ::..  .* :::            :    

MG07123.1 	PPPFIRRG---VGPFSAPHNVQ--GGQEPEEKEPDETEELQKKLDDAKLSPEAAKVAARE
NCU08303.1	KPPVKAPGHGAGMPFSPQGGFMGRGGNADEDQEPNEIEELQKRLDAARLSPEAAKVADRE
FG09725.1 	GSLPQIP----GMAFVPPNGQMPGGNDHNDDQEANELDELKRKLSSAKLPTEAAKTVDRE
AN0122.1  	IRDLLSRRGIPGASGTPPPGLG-GRNNEADEKESNELDELQQRLKDAQLSPEAQKVADKE
          	          .  .  .  .     .:  :::*.:* :**:::*. *:*..** *.. :*

MG07123.1 	MKRLKKMMPVQAEYSVLRTYLETLAEIPWSLTTDDRLDKTALGRARKQLDDDHHGLSKVK
NCU08303.1	IKRLKKIHPAQAEYAVTRTYLETLAEIPWTTTTDDRLGPDTLNRARKQLDEDHYGLDKVK
FG09725.1 	LRRLQKMQPMNQEYQVTRNWLETLAEIPWTATTDDRLGPETLHRARKQLDDDHYGLDNVK
AN0122.1  	MRRLRKMMPVNQEYGVIRTYLENLADIPWTKVTEDKLGPETLKAARKQLDDDHYGLEKIK
          	::**:*: * : ** * *.:**.**:***: .*:*:*.  :*  ******:**:**.::*

MG07123.1 	QRLVEYLAVLRLKQSANEEIDAQIKRIQEEMVPPLEGESADYPEPGLTKASQ-----ESL
NCU08303.1	KRLLEYLAVLRLKQAINDDVDSQIKQLEQELGVASESSK-EDAAQSTPIDLG---VDEKA
FG09725.1 	KRLIEYLAVLRLKQSINDEVEEKIKKAEQETVQSTANEQGSQEKQDKEIVEGSPATGDAT
AN0122.1  	KRLLEYLAVLRLKQSTNQGLEQQISILTKELDNS--GGDIEKDIPSLPESDR--------
          	:**:**********: *: :: :*.   :*   .  . . .    .              

MG07123.1 	DAAQAKIDMLKSRRMTDKSPILLLAGPPGVGKTSLAKSVATALGRKFHRISLGGVRDEAE
NCU08303.1	KAGGDKLEALKSRRMVDKSPILLLVGPPGVGKTSLARSVATALGRKFHRISLGGVRDEAE
FG09725.1 	KPEMAKLEILKSQRMVDKSPIMLLAGPPGVGKTSLARSVATALGRKFHRISLGGVRDEAE
AN0122.1  	AAIESKLNALTSKRTVDKSPILLLVGPPGTGKTSLARSVATALGRKFHRISLGGVRDEAE
          	 .   *:: *.*:* .*****:**.****.******:***********************

MG07123.1 	IRGHRRTYVAAMPGLIIQGLKKTGVSNPVILLDEIDKISQSNHHGDPSAAMLEVLDPEQN
NCU08303.1	IRGHRRTYVAAMPGLIVQGLKKVGVANPVFLLDEIDKVGGSSIHGDPSAAMLEVLDPEQN
FG09725.1 	IRGHRRTYVAAMPGLIVQGLRKVGVANPVILLDEIDKIGHASIHGDPSAAMLEVLDPEQN
AN0122.1  	IRGHRRTYVAAMPGVIVNGLKKVGVANPVFLLDEIDKIGGPNFQGDPSAAMLEVLDPEQN
          	**************:*::**:*.**:***:*******:. .. :****************

MG07123.1 	ATFVDHFINIPVDLSKVLFIATANSLETIPPPLLDRLEMIYLSGYTTLEKRHIATNHLVP
NCU08303.1	HNFTDHYVNVPIDLSKVLFIATANSLDTIPAPLLDRMETIYIPGYTTLEKRHIAMRHLVP
FG09725.1 	YNFQDHYVGMPIDLSKILFIATANSLDTIPAPLLDRMETIYIPGYTTLEKRHIAMQHLVP
AN0122.1  	HTFVDHYINIPIDLSKVLFIATANSLDTIPAPLLDRMETIQLSGYTTVEKRHIAKRHLLP
          	 .* **::.:*:****:*********:***.*****:* * :.****:****** .**:*

MG07123.1 	KQIRANGLSPEQIVFPEEVVSKIIESYTREAGVRNLEREIGSVCRAKAVEYADAKDDNRL
NCU08303.1	KQLRVNGLDESQVSFTPEVVSRIIESYTREAGVRNLEREISSVARGKAVEFADAKDSGHP
FG09725.1 	KQIRVNGLAESQVTFNREVVSKIIDCYTRESGVRNLEREIGSVCRAKAVEYAEAKDAGHI
AN0122.1  	KQIRANGLSDGQVVLSDDVIDKTTTSYTRESGVRNLERELGSICRYKAVQFADATDSAKL
          	**:*.***   *: :  :*:.:   .****:********:.*:.* ***::*:*.*  : 

MG07123.1 	DQYRAQLTVEDIEHILGSAKYEDEIAEQEGRPGVVTGLVAYSSGGNGSILFIEVADMPGD
NCU08303.1	ENYNPQLTVDDLEKFLGIEKFEEEIAEKTSRPGIVTGLVAYSSGGNGSILFIEVADMPGN
FG09725.1 	EHYRPELSVEDIEDILGIEKFEEEIAEKTSRPGIVTGLVAYSSGGNGSILFIEVADMPGN
AN0122.1  	ESYNPVVTVDDLEEILGIERFDEEIAEKHGRPGVVTGLVAYSTGGQGSILFIEVADMPGS
          	: *.. ::*:*:*.:**  ::::****: .***:********:**:*************.

MG07123.1 	GSVQHTGQLGDVLKESARVALSWVKANAFQLGLSPDPREKIMENRSIHVHCPAGAIPKDG
NCU08303.1	GSVQLTGKLGDVLKESVEVALTWVKAHAYQLGLTQSPSENIMKDRSIHVHCPSGAVPKDG
FG09725.1 	GRVQLTGKLGDVLKESVEVALTWVKAHAFELGLTPEPTTDIMKERSIHVHCPSGAIPKDG
AN0122.1  	GRVQLTGKLGDVLKESVEVALTWVKAHSFELGLTSDPNEDIMKNRSLHVHCPSGAIPKDG
          	* ** **:********..***:****::::***: .*  .**::**:*****:**:****

MG07123.1 	PSSGIAQAIALISLFSGKIVPPTMAMT0GEISLRGRVTAVGGIKEKLIGALRAGVKTVLL
NCU08303.1	PSSGISQAIALISLFSGKAVPPTMAMT0GEISLRGRITAVGGIKEKLIGALRAGVKTVLL
FG09725.1 	PSSGIGQAIALISLFSGKPVPPTMAMT0GEISLRGRVTAVGGIKEKLIGALRAGVKTVLL
AN0122.1  	PSAGLAHTIGLISLFSGKAVPPKLAMT~GEVSLRGRVMPVGGIKEKLIGALRAGVTTVLL
          	**:*:.::*.******** ***.:*** **:*****: .****************.****

MG07123.1 	PKQNEKDVQDLPQEVKDGLKIILVS2RL--------------------------
NCU08303.1	PAQNRKDAKDLPQEVKDGLEIIHVS2HIWEAIRYVWPDGQWPSEHDYPSIESRL
FG09725.1 	PAQNRKDVKDLPQEVKDGLEILHVS2HIWEAIRLVWPDSHWAEDSNYRGIESRL
AN0122.1  	PHQNRKDVKDVPEEVSNGLEIIYVK2HIWEAIRHIWPDAHWPGQHHMDFVESRL
          	* **.**.:*:*:**.:**:*: *. :: .:     ...  . .      .:
```
