# Supplementary material for: Patterns of Intron Gain and Loss in Fungi
Source: PLoS Biol. 2004 Nov 30;2(12):e422. doi: 10.1371/journal.pbio.0020422 (PMC532390; doi:10.1371/journal.pbio.0020422)
Supplement: Table S1 — Also available at http://genes.mit.edu/NielsenEtAl/. (4.3 MB ZIP). [file pbio.0020422.st001.zip › NielsenEtAl/html/1133.html]

AN3122.1.NCU04511.1.MG00106.1.FG04031.1


```
 CLUSTAL W (1.82) Multiple Sequence Alignments - Introns Inserted


Sequence 1: NCU04511.1	691 aa
Sequence 2: FG04031.1	664 aa
Sequence 3: MG00106.1	695 aa
Sequence 4: AN3122.1	576 aa
Alignment Length: 702 aa
Number Identitical Residues: 306 aa
Alignment Score (without introns) 16303


MG00106.1 	MVAPAVPE2ITEEILHEAIDARTDQLIALRELGPPDLVHLTKQQTRSPGKQ0IGVYHHVT
NCU04511.1	MVAPAVPE2ITEEILHEAVDTRTESLSSLRELGPPDLVHLVKQPLKNPGKH0YGVYHHVT
FG04031.1 	MVAPAVPE2ITEEILHESIDARTESLAGLRELGPPDLVHLLKHGVRNPAKQ0TGVYHHVT
AN3122.1  	--------~------------------------------------------~--------
          	                                                            

MG00106.1 	GVDASSSASLAAYINTLTYKDHGQAATTKIVQGVYC2CYNALSRIDMRVHVTIPGSVESY
NCU04511.1	GVDASSSASLAAYINTLTYKEFGNSATAKTVEGTYC2CYNAFSRVDMRVHAPFPGSVESY
FG04031.1 	GVDASSSASLAAYINTLTYKESGPNATNKIVEGVFC2CYNAFSRLDMRVHVSIPGTVESY
AN3122.1  	------------------------------------~---------MRVEVKIPGSLESY
          	                                              ***.. :**::***

MG00106.1 	GVDERGEKRKATDELWLETYLCSVLRAYSYADDGSGETIRKIMGVRRFNPVTSTETEHRF
NCU04511.1	CVDERGEKRKATDELWLETYLCSVLRAYSYADDGSGETIRKIMGVRRFNPVSSTETEHKF
FG04031.1 	CVDERGEKRKASEDLWLETYLCSVLRAYSYADDGSGDTIRKIMGVRRFNPVTNTETEHRF
AN3122.1  	CIDERGDKRVATDALWLETFLCGVLRAYSYADDGSGDAIRKIVGVRRFNPVTNTEMEHRF
          	 :****:** *:: *****:**.*************::****:********:.** **:*

MG00106.1 	LSAAEQLFFRG1WQLGSDSTVQVPNVVSNHLASGLLRYFQTTGRFVSGINLFEKLRSQNV
NCU04511.1	LSAAEQLFFRG1WQLGSDSVVQVPNNVSNHLTAGLLKYFSTTGRHTSGINLFEKLRSQNV
FG04031.1 	LHAAEQLFFRG1WQLGSDSVVQVPTNVSNHLTTGLLKYLETTGRYASGINLFEKLRTQSV
AN3122.1  	LDAAEKLFFLG1RQLSSDPETQVPNTVSNHLTAGLLKYIQTTGRYASGINLFEKLRTRDV
          	* ***:*** *  **.**. .***. *****::***:*:.****..**********::.*

MG00106.1 	EVASLLSKVLFLGNEEVKGVRVLHQALQEIPMDYVMLDAQAEFLLKKAKTATTPEQKEER
NCU04511.1	EVASLLAKVLFMGNEEVQGVRVLYEALKESPMDYVMLDTQAEFLLKKAETAPTPELREER
FG04031.1 	EVSSLLAKVMFMGHEEVAGVRTLHQSLKENPMDYVMLDTQAEFLLGKAKNSATPELKEER
AN3122.1  	EVSSLLAQVFRMADEEVQAVRLMYDALQDVPMDYALLDCQSAFCASKGEGE---------
          	**:***::*: :..*** .** ::::*:: ****.:** *: *   *.:           

MG00106.1 	LRMALACADRSTIAAPSEFGTWARLAEVYVAMEDWENALTILNSCPMFTYQDKDAPLMPE
NCU04511.1	LRMALGCADRSTIAAPSEFGTWARLAQVYVAMEDWENALTILNSCPMFTYQDKDAPVMPE
FG04031.1 	LKLSLGCADRATVAAPTEFSTWARLAQVYVAMEDWDNALTILNSCPMFTYQDKDTPLMPE
AN3122.1  	--MALECAKRAVTAAPSEFSTWARLAEVYVCLEKWDLALLTLNSCPMFTYQDKDTPRMPQ
          	  ::* **.*:. ***:**.******:***.:*.*: **  *************:* **:

MG00106.1 	PKDVYLPTLVETRLDEID-SEP-EGRYNEQVAEPLLNLRAAGYRGTFKHAYKILTEMTSK
NCU04511.1	PKDVNLPTLPETRLDEID-SEP-DSRFSEQVDPSLLGLRAAAYRGTFKQAYSILTEMTAK
FG04031.1 	PKEVILPTLPETRLDEID-SEP-ESRYSEQVDPSLLNLRAASYRGTFKKAYEILTEMTAK
AN3122.1  	PSRIMLPILAESMLDEIDEGQPKQGDPHDYVHPSLRRLHAATYQGTFLKAYNLLTKIAAA
          	*. : ** * *: *****..:*.:.   : *  .*  *:** *:*** :**.:**:::: 

MG00106.1 	IGWDQLLKTRSIVFVMEDEYHMERTEVRDS-TQERSASTDGLRGSSPNNTNTAAAQDGES
NCU04511.1	IGWDQLLKIRSNVFVMEDEYRNEKQEPSYP--AKRNASTDALRGSPDHTTNGETAPKEAA
FG04031.1 	IGWDQLLKIRSNVFVMEDEYRTEKQESTHPGASKRTPSTDGLRGTPDQTVN---------
AN3122.1  	IGWDQLLKVRSEVFVMEEEYRSERQHSTKPSIHSHGDSPAESAESTEPAETAETNGSREQ
          	******** ** *****:**: *: .   ..  .:  *.     :.    .  :  .   

MG00106.1 	DTDS---HIETGPAEKADSAAPN~GQGLEKPLTTADPSEVKEDQE~N0GI-VPSDHSKLN
NCU04511.1	ETDE---VEDEK--KNATLAAEQ~TEGLERPPSAIDPEVVRKAEE~N0GD-NEDHFSRLN
FG04031.1 	RDNE---AEGE----NAEKPAEE0RQ--------------RQILS2-~----DENLSKLN
AN3122.1  	ENGDGRPVESVTEIVNGDDHTQG~ENSIERPEQTMASEVVKSGND~D0PDPSHASYTQFK
          	  ... .        :.   :    :. . .  :  ..  :.  . .   .     ::::

MG00106.1 	NKRLCERWLDSLFMVLYEDLRQYTIWRTQMAQLKLQSLQYKKSAEEWEILGALAERLHHT
NCU04511.1	NKRLCERWLDSLFMVLYEDLRVYTIWRTQMAQYRAQSMQYKKSAEEWEILGSLAERLQHT
FG04031.1 	TKRLCERWLDSLFMVLYEDLRVYTIWRTQMAQYRAQQMQYKKSAEEWEILGSLAERLQHM
AN3122.1  	NKRLCERWLDNLFMVLYEDLRIYTIWRTEVAQYRQQAMEYKKSATEWEILGELAERLHHF
          	.*********.********** ******::** : * ::***** ****** *****:* 

MG00106.1 	DEAVEAYRACLSQRFSPKALTGILRAYERKPNSTRDTVAAVIRLVTWQYRWYSE~FSPEL
NCU04511.1	DEAVEAYRACLGQRFSPKALTGILKVFE-KQKSTRESVAALIRLVTWQYRWYSE~FSPEL
FG04031.1 	DEAVEAYRACLSIRFSPKALAGILRVFE-KTKSTRETVASVIRLVTWQYRWYSE~FSPEL
AN3122.1  	DEAIEAYQACLSIRFSPKAMRGILKLHE-KQNDTRGMLSALIRLIAWQYRWYSE0L----
          	***:***:***. ******: ***: .* * :.**  ::::***::******** :    

MG00106.1 	LNTIRSLIEDEGAVKVRSIIQATSLPQNVLDLTHHYAALCATFRSSGTEG
NCU04511.1	LHTIRTLIEDEGAVKVRSIIQATNLPQNVLDLTHHYAALCATFRSSGTDG
FG04031.1 	LHTIRTLIEDEGAVKVRSIIQATSLPQNVLDLTHHYAALCATFRSSGTDG
AN3122.1  	-------IEDEGAVKVRSIVQATNLPQHVLDLTHQYCQLCATFRSSGSDG
          	       ************:***.***:******:*. *********::*
```
