# Supplementary material for: Patterns of Intron Gain and Loss in Fungi
Source: PLoS Biol. 2004 Nov 30;2(12):e422. doi: 10.1371/journal.pbio.0020422 (PMC532390; doi:10.1371/journal.pbio.0020422)
Supplement: Table S1 — Also available at http://genes.mit.edu/NielsenEtAl/. (4.3 MB ZIP). [file pbio.0020422.st001.zip › NielsenEtAl/html/1134.html]

AN0817.1.NCU02621.1.MG01209.1.FG00662.1


```
 CLUSTAL W (1.82) Multiple Sequence Alignments - Introns Inserted


Sequence 1: AN0817.1	566 aa
Sequence 2: FG00662.1	699 aa
Sequence 3: NCU02621.1	709 aa
Sequence 4: MG01209.1	771 aa
Alignment Length: 857 aa
Number Identitical Residues: 140 aa
Alignment Score (without introns) 7243


MG01209.1 	----MKKGALRGWFSSSTEAPSDKGKPANTSSDSRPTRISAISSRSSSSACPSPRPSPG-
NCU02621.1	----MKR-SLKWFSSKTTSSPSNSPEPAGPSVGAGGNNSYRASPSSVSAAFRNPSPSDS-
FG00662.1 	METFFGQRFQKPSDTMGTQEPREFTSTNCTGVWDSANTIRPQGLAPSDIVPAATANASCC
AN0817.1  	---------------MNTTTPIDIATR--------QTSVSPPGQQASNLT----------
          	                 *  * :             .     .  . . .          

MG01209.1 	------------------------GAR-PSTVAAPGGKRSSDTTTTTTT-----------
NCU02621.1	------------------------TTDSPSSLLHPSSPSPSPTPQPLTP-----------
FG00662.1 	DRASSNPSACACDSRTHFVAGAAYDPDSPGSLVNSGSSSLTQSQSPSPQPGTPTTGHFLP
AN0817.1  	-----------------------------SALQKAGNTERTGSIS---------------
          	                             .::  ...   : :                 

MG01209.1 	--CNNTAAPTSTDNTTTTTTTTTTTTTTARNHDINRRYRASRIDDDN------------N
NCU02621.1	--RDSVFPRSDLSPLTASKPIDIAVSHHRSTASISPADRNP-ITFPT------------H
FG00662.1 	GAHAKTAPTPAAPAAASVLSRSITTAKAAAPQLSIGGDFDPAILSNTSIEDFDSYLNNFR
AN0817.1  	--HANGVP--------------ISMFKASAPR---------------------------K
          	    .  .               :                                   .

MG01209.1 	NNNNRGVSFSR--SISPSGLYQSTSSASPSPQPPTPTAERRASISRTKPRPIAFPGSQSP
NCU02621.1	DLDDLDINWDQGPCLADLGFDPDVDMTAGQHFDSALSRSRQESFLGTGAKPISMANPN--
FG00662.1 	DSPTADSSIYIHIPDPSGLEQQLYDHNMTTGPSLDPTMGRRDSFVSAGPKPISMNNPNRG
AN0817.1  	DS-----------------------------------IGAASTQWGNGTKPISMSGSNR-
          	:                                         :     .:**:: ..:  

MG01209.1 	SLRLSSLRESLPNFDQEDFGGFIHDDTRDDIVVIDDFFRPSSLSDPKTAADMTAGPIDSA
NCU02621.1	-------RDHVNRIRRESMAGSLRAG--------------SLMAGSLMGNGMSWGGIS--
FG00662.1 	--------DNANRNRRESLAG------------------------SLMGGGMSWGGMS--
AN0817.1  	-----------NQQRRESLAG------------------------SLVGG-MSWGGVS--
          	            .  :*.:.*                        .  .  *: * :.  

MG01209.1 	MGRSRQDS~FVSAG-PKPIS-MINPNRADNRPRRESVAGSLMGGMSWGGMSVSSFIRDE2
NCU02621.1	VGSFIRED2MMMTG-TSPYL-THQSPSFHSSSYIPKLEANFMRDFTC---------CDR~
FG00662.1 	FGSFVRDD2IMMATSPSPFGGAHQSPSFHSSSYLPKLEANFMRDFTC---------CGK~
AN0817.1  	VGSWIRDD2IIMAG-TSPFT-TFQSPSFHSSSYLPKLEANFMRDFSC---------CGV~
          	.*   ::. :: :  ..*     :.   .. .   .: ..:* .::           .  

MG01209.1 	TWPTMHDLLTHYEESHTNGAPSERNGSQMNAN-ANQQTPKTPARALASTSTFGTPQTARP
NCU02621.1	TWPTLHDLLQHYEENHHATAAPNTSNQNLNTFGTNQGNARGTASRATPTPAASRAQPGAQ
FG00662.1 	ILPNLHDLLQHYEEAHTQPSPNTARNNAFSQF-------SQMGMSSAPRMSISRADSAAP
AN0817.1  	TLPTLHDLLQHYEEAHATKSGHRPSQ-------------------TDNRAALAAAAIAQQ
          	  *.:**** **** *   :                              : . .  .  

MG01209.1 	SQGQGQQTRPGGVGAAGTAALGMGGLQQLMRQQQQQQQQNTNQKQTASLSQLQNDDLDTV
NCU02621.1	AMNGYQGQRHLSVAGAG---MGLGGIGQMMRQQQVAP----AVSKMSSMSHMN-DDMDTV
FG00662.1 	GNNSQLSSQ----------------LGQHNRGQQSPH----DLHGNSTMPSNLNDEMDAV
AN0817.1  	NQQNNNQNR----------------GLQPDRTFDMQR----KMN--QSHTPQQHSDMDTI
          	        :                  *  *  :             : .    .::*::

MG01209.1 	HDMEMDDPVGTMDLDDGEGDAGNASIQQTRQLFGQQQRNLPLNNASGL-VHQGLRTSQPS
NCU02621.1	GEMELDETVGPMEMDDNQ-----RTIQQTRQLFGQQQRSQLHLNSSGL-PHQALRTSQPP
FG00662.1 	ADMEMDDAVGTMEMDD-----SQQRMSQTRQLFGQQR---PELNMNTSGLTQGLRTSQPP
AN0817.1  	DDMELDDAMDDTDASSQF--FTPQSRDPTQGGFGTPNRGVPNLNLSMLPSHQGFKSSQPG
          	 :**:*:.:.  : ..          . *:  **  . .    * .     *.:::*** 

MG01209.1 	TPLAG--NFNFQD-PTVSSVNTPTLTTHG-LPQTTQGQFANTSFASMDSDMDEEIPGMPM
NCU02621.1	TPAAV--SFGFQNNPTVSSVNTPTLTTQGGLPQ--RGQFG------QEDDNGDDMSGMPM
FG00662.1 	TPAAA--SFGLQNNPTVSSVNTPTLTTQGQTPQ------------GQQVDMDEDLPGMPM
AN0817.1  	TPVATGLPLSLQNNPTVSSVNTPTLMPNPLQNS------------QFRGTPDSSTPGTPA
          	** * .  :.:*:.*********** .:    .                  ... .* * 

MG01209.1 	QMN---LGNMNLN---------------FGGFDTSNLNCIQDPGKRLFSPGGATQAQQQQ
NCU02621.1	KMN---IGGVNLNGGQLGGL--------AGNLAFGALGTIDDPAKRLYSPGGTTQMTSQQ
FG00662.1 	GGNTNDIGDMGFNGNQ-------------TGND--SNFCINDPGKHLFSSNG--FPPNRS
AN0817.1  	ELDDSMIGPFGELSMQNAMMQGQPQFSRFTGNNDMVDLCIDEPAKRLFSPTGGINQSNAH
          	  : . :* ..  . . .  ..... :   .        *::*.*:*:*. *     .  

MG01209.1 	QQQQQQQHQREQQILRNQQLLQQQLAQFNLETQQFPPGTNTEALLAQMREMMMP--EENK
NCU02621.1	RAFDAQ--------MQMQQQLQQHLASMNLDLNQLAPGTDPALLLQQMTALMMPPTEEHK
FG00662.1 	------------------IQAQLQQLGINQ--SQLNDPQTNKAIMQRLQSMMMP--EEHK
AN0817.1  	------------------FKLSGAQYGPNSDIARRIREQQLLAGVPDTTSILPN--EEPK
          	                     .      * .  :          :     ::    ** *

MG01209.1 	PYKCPVIGCEKAYKNQNGLK2YHKT~HGHATQQLHENGDGTFSIVNPETSAPYPGTMGME
NCU02621.1	PFRCPVIGCEKAYKNQNGLK2YHKT~HGHSTQQLHENGDGTFSIVNPETSTPYPGTLGME
FG00662.1 	PFKCPVIGCEKAYKNQNGLK2YHKT~HGHQTQQLHENGDGTFSIVNPETSAPYPGTLGME
AN0817.1  	PYRCPVIGCEKAYKNQNGLK~YHKA0HGHNNQQLHDNADGTFSIVNPETSAPYPGTLGME
          	*::***************** ***: *** .****:*.************:*****:***

MG01209.1 	KEKPFKCEACGKRYKNLNGLKY0HKAHSPFCDPELKLQQQNALAVAAAAAALNQNQLALN
NCU02621.1	KEKPFKCDVCGKRYKNLNGLKY0HKQHSPMCDPEMRAQHQNLISTMMSNPAA---LIAFQ
FG00662.1 	KEKPFSCETCGKRYKNLNGLKY0HKAHSLPCNPDFKLQALVNMN----------------
AN0817.1  	KEKPYRCEVCGKRYKNLNGLKY~HKSHSPPCNPDFQLAAARSLNYGGGVMQGQNINVAGA
          	****: *:.************* ** **  *:*:::      :    .    ..   :  

MG01209.1 	LGLIHGGGGGGLPNINEDTLL-
NCU02621.1	Q---------NLPNINEDVML-
FG00662.1 	-----------LPGIGEDQMMQ
AN0817.1  	G----------LPGIGEEGLL-
          	           **.*.*: ::
```
