# Supplementary material for: Patterns of Intron Gain and Loss in Fungi
Source: PLoS Biol. 2004 Nov 30;2(12):e422. doi: 10.1371/journal.pbio.0020422 (PMC532390; doi:10.1371/journal.pbio.0020422)
Supplement: Table S1 — Also available at http://genes.mit.edu/NielsenEtAl/. (4.3 MB ZIP). [file pbio.0020422.st001.zip › NielsenEtAl/html/1135.html]

AN2464.1.NCU00008.1.MG05189.1.FG05525.1


```
 CLUSTAL W (1.82) Multiple Sequence Alignments - Introns Inserted


Sequence 1: NCU00008.1	431 aa
Sequence 2: MG05189.1	435 aa
Sequence 3: FG05525.1	456 aa
Sequence 4: AN2464.1	426 aa
Alignment Length: 480 aa
Number Identitical Residues: 193 aa
Alignment Score (without introns) 10141


MG05189.1 	MAT---SEPFPLLSTSAD-QLHN-------------AADRVSASAVRRRRQSSGLGSELR
NCU00008.1	MAPSTKSEPFPELSSAADRQLHS-------------EAT-----AARRRRKSSGLGQALR
FG05525.1 	MSG---SEPFPLLNTAND-QLHQRHQATSSVSVSNTTATQDAINNARKRRKSSALGGEIR
AN2464.1  	---------------------------------------------MARTRKSSNLGSDIR
          	                                               : *:** **  :*

MG05189.1 	VGDTGAPGLATSIA~NMGQ~N~K0DATKRAP-----RRRKAY--FRRFKQFATKHTCVLP
NCU00008.1	VGDTGAPALATSLA~LSQK0D~S~KDVAAPPGKKYSKRRKARSLLRRAKNYAIKHTWALP
FG05525.1 	-GDTGAPAFASSRA~SLDA~T0S~DGRKRLS-----KRRRARGLIARAKQTMIKHTWVLP
AN2464.1  	-GDTSAPAMSTMNE0VAPL~A~E~KSSKSHS-----KRRRSRSFLRRFKDTCLKHTWVLP
          	 ***.**.:::           . .     .     :**:: . : * *:   *** .**

MG05189.1 	LVLLFIFLVLYAINPTESNPISRFIFLSYELPPDAPGKPPQYGKGRWDIAFVTFYTVVLS
NCU00008.1	LAILMAFLSLYAINPTESNPIHHFIFLSYKLPTSSSDEIPQYGKGLWDIAFVSFYTIVLS
FG05525.1 	AILLTLFLVGYAFNPTESNPIHNFIFLSYKLPQDDLSAPAQYGKGRWDLAFVSFYTIVLS
AN2464.1  	LLILIVLLAGYAVNPTPSNPLHYAIFLSYPEPPKTPGGPVMYGKGPKDIAFVSFYMVVLS
          	  :*  :*  **.*** ***:   *****  * .  .    ****  *:***:** :***

MG05189.1 	FTREFIMQELLRPLSRYAGVKSRGKQARFMEQAYTAIYFAILGPAGMYVMSRTPVWYFNT
NCU00008.1	FTREFIMQEVLRPMARWAGLKSRGKQARYMEQMYTALYFGIMGPVGMFVMSRTPVWYFNT
FG05525.1 	FTREFIMQELLSPLARYYGL-SRGKKARFMEQVYTAIYFGVLGPVGLWVMSHTPVWYFNT
AN2464.1  	FTREFLMQRMIRPFAVYCGIRGKGKTARFMEQVYTAIYFAIFGPYGLYVMSRTNIWYFNT
          	*****:**.:: *:: : *: .:** **:*** ***:**.::** *::***:* :*****

MG05189.1 	HGMYENFPHKTHEACFKFYYLFQAAYWAQQAIVLVLGMEKPRKDFKELIAHHIVSLALIA
NCU00008.1	VGMYENFPHKTHVAVFKFYYLFQAAYWAQQAIVLLLGMEKPRKDFRELVCHHIVSLALIG
FG05525.1 	YGMYDGFPHLTNLAPVKFYYLFQAAYWSQQAIVLLLGMEKPRKDFKELVGHHIVTLGLIA
AN2464.1  	TAMFEGFPHREHEGLFKAYYLLQASYWAQQAIVLLLQLEKPRKDFRELVGHHIITLALIA
          	 .*::.***  : . .* ***:**:**:******:* :*******:**: ***::*.**.

MG05189.1 	LSYRFHFTYIGLAVYVTHDISDFFLA0TAKLMNYIDHALTGPYFAFFMGVWIYLRHFINL
NCU00008.1	LSYRFHFTYIGLAVYITHDISDFFLA0TSKTLNYLDHALTGPYYFTFMCVWIYLRHYLNL
FG05525.1 	LSYRFHFTYIGLAVYTTHDISDFFLA~TSKTLNYIDSPLVGPYFGVFMMAWIYLRHYLNL
AN2464.1  	LSYRFHFTYLGLAVYITHDVSDFFLA0TSKTLNYLDAYITAPYFGVFVCVWIYLRHFLNL
          	*********:***** ***:****** *:* :**:*  :..**:  *: .******::**

MG05189.1 	RIIWSLLTEFQTVGPFELNWETQQYKCRLSQVITLGLLSSLQALNLFWLFFIVRIAYRFV
NCU00008.1	RIIWSLFTEFKTVGPYELNWETQQYKCSLSFVITLALLGSLQALNLFWLFFIIRIAYRFV
FG05525.1 	KIIWSLFTEFETVGPFELNWETQQYKCRIAQVITASLLCALQALNLFWLFCIARIAWRFV
AN2464.1  	KFLWAVLTEFRTVGPFELNWETQQYKCWISQYITFALLASLQAVNAFWLFLILRILKNYL
          	:::*:::***.****:*********** ::  ** .** :***:* **** * **  .::

MG05189.1 	FNDNLRDDRSDVETE---AEDSTEPAATTATANGASVANGSAIKKRK-------------
NCU00008.1	VHKVAKDDRSDDESE---LEDDVTEEKPT--------VNGSTTKR---------------
FG05525.1 	SQNDLQDDRSEDEDDGEIEDDEVASPSSKANGSTNGHANGHANGKTAADMKN--------
AN2464.1  	FSNIKKDERSDEEDE---EEEEIEQSTNSTAALATGTEPASLTARSVNVEKRTPHVLVNG
          	  .  :*:**: * :    ::.      .: .   .   .    :     . :.    ..

MG05189.1 	-----
NCU00008.1	-----
FG05525.1 	-----
AN2464.1  	QPVKR
          	.. .
```
