# Supplementary material for: Patterns of Intron Gain and Loss in Fungi
Source: PLoS Biol. 2004 Nov 30;2(12):e422. doi: 10.1371/journal.pbio.0020422 (PMC532390; doi:10.1371/journal.pbio.0020422)
Supplement: Table S1 — Also available at http://genes.mit.edu/NielsenEtAl/. (4.3 MB ZIP). [file pbio.0020422.st001.zip › NielsenEtAl/html/1136.html]

AN1216.1.NCU03068.1.MG00187.1.FG07096.1


```
 CLUSTAL W (1.82) Multiple Sequence Alignments - Introns Inserted


Sequence 1: NCU03068.1	220 aa
Sequence 2: MG00187.1	244 aa
Sequence 3: FG07096.1	240 aa
Sequence 4: AN1216.1	236 aa
Alignment Length: 249 aa
Number Identitical Residues: 96 aa
Alignment Score (without introns) 5029


MG00187.1 	MGSIQVPEGTFTAPPQQVSLDGFLFDMDGTIIDSTRA~IEMHWAS2VGKEIGVDPELILK
NCU03068.1	--------------------------MDGTIIDSTAA~VEKHWEA2IGKEIGHSPEVILQ
FG07096.1 	MGSTP----EYIQPPQELTFDGFLFDMDGTIIDSTEA~VVKHWET2IGNEIGVAPEVILE
AN1216.1  	MAATG----SFSAPPQVLTFDGLLSDFDGTIVDSTDA1IVKHWHK2IGAELGVDPKTILA
          	 .:          ...  : ..   .:****:*** * :  **   :* *:*  *: ** 

MG00187.1 	~TSHGRRSIDTLKIYCPEKATIEY1ASEMEGRLPKLYSKEAEEIPGARSLLDSIIAAKAA
NCU03068.1	2TSHGRRSIDILKELAPEKATLEY1VQHMEGLLPKKYGDDAVEIPGARALLQDLIDRKK-
FG07096.1 	~TSHGRRSIDILKILAPKKANWDY1VRDMEGRLPKYHGHEAVEIPGARSMLEALIARSS-
AN1216.1  	~TSHGRRSIDTLQLYDPAKANWEY1VSYIEGLIPKEYGSDAIEIPGARSILAALEETGA-
          	 ********* *:   * **. :* .  :** :** :. :* ******::*  :      

MG00187.1 	PWAIVTSGTKPLVNG~WLEALNLPR--PAHMITAESVENGKPDPTCYLMGLDGLGLRDRA
NCU03068.1	PWAIVTSGTLPLVSG0WLDVLSLPH--PAHLISAESVVNGKPDPACYLLGRERLGLSNPN
FG07096.1 	PWAIVTSGTVPLVTG~WLRARDLPTPLPEHLVTAESVENGKPDPACYRLGRERLGLQAED
AN1216.1  	TWGVVTSGTRALIDG~WLGVLKLTH--PDVLVVAEDVELGKPDPRCYLLGRKKMGLEHS-
          	.*.:***** .*: * ** . .*.   *  :: **.*  ***** ** :* . :**    

MG00187.1 	ADVLVLEDSPAGILAGKAAGCKVLGLVTSHTVEQVVGAGPDWVVRDLSSVRLVG-AEG-G
NCU03068.1	VEVLVLEDAPAGIRAGKAAGCKVIGLVTSHTVEQVLEAGPDWVVRDLSSVKFVPESSGEG
FG07096.1 	AQILVLEDSPAGIRAGKAAGCKVLGLVTSHTVEQVVAAEPDWVVRDLSSVEVLR-SEG-G
AN1216.1  	SSIVVLEDAPSGIKAGKAAGFTVIALTTTHTLEQLQAAGADVIVEDLRSISVKGVVDG--
          	 .::****:*:** ****** .*:.*.*:**:**:  * .* :*.** *: .    .*  

MG00187.1 	RVTLEIVDALESK-
NCU03068.1	KVTLEIRDALVLKN
FG07096.1 	KVTIKISNALRL--
AN1216.1  	RVQLEVRNAFQ---
          	:* ::: :*:
```
