# Supplementary material for: Patterns of Intron Gain and Loss in Fungi
Source: PLoS Biol. 2004 Nov 30;2(12):e422. doi: 10.1371/journal.pbio.0020422 (PMC532390; doi:10.1371/journal.pbio.0020422)
Supplement: Table S1 — Also available at http://genes.mit.edu/NielsenEtAl/. (4.3 MB ZIP). [file pbio.0020422.st001.zip › NielsenEtAl/html/1137.html]

AN5602.1.NCU04087.1.MG00814.1.FG09535.1


```
 CLUSTAL W (1.82) Multiple Sequence Alignments - Introns Inserted


Sequence 1: NCU04087.1	326 aa
Sequence 2: MG00814.1	368 aa
Sequence 3: FG09535.1	325 aa
Sequence 4: AN5602.1	359 aa
Alignment Length: 368 aa
Number Identitical Residues: 165 aa
Alignment Score (without introns) 8446


MG00814.1 	MVLHNPNNWHWVNKDVAPWAKQWFQDNLTKLEVQDGDVTAKVSKVVSMDGDVDVSQRKGK
NCU04087.1	MVLHNPNNWHWVNKDATSWAKQWFEDNLTKLEAKEGDVTAKISKVISMDGDVDVAQRKGK
FG09535.1 	MVLHNPNNWHWVNKDVSGWARSYLDEAVAKVQVEDGEVKAKIDKIQSMDGDVDVSQRKGK
AN5602.1  	MVLHNPNNWHWVNKDVSAWAKTYLKEKLRAVSAEEDGVTAKVSDVLTMDGDVDVSQRKGK
          	***************.: **: ::.: :  :..::. *.**:..: :*******:*****

MG00814.1 	VITIFDVKLVLEYSG1NIADGDEVTGTITIPEVAHDTEENEFV0FEIDIYSDSKEKQPVK
NCU04087.1	VITIFDVKLTLEYTG1STATDDNVSGTITVPEVSHELDEDEFV~FDIDIYSDANEKRPVK
FG09535.1 	VITIFDVKLVLQYSG1SAPGEDDVSGTITVPELAHDTEEDEYV0FDVDIFAESKEKQPVK
AN5602.1  	VITLFDVKLQLEYEG1KTKDEEAVSGTITIPEVAHDTEEDEYV0FDIDIYSDSPSKQPVK
          	***:***** *:* * .    : *:****:**::*: :*:*:* *::**:::: .*:***

MG00814.1 	DAVRSKLVPKLRSEFVKLAPALIAEHGKDIQHAPGSNPSSGFSTPKVHAPSGAAAKATTS
NCU04087.1	DLVRNKLVPQLRKEFLKLSPALIAEHGKDIQHAPGSNPSSGFSTPK-FVPQPASSSARAV
FG09535.1 	DLVRSKIVPQLRQEFQKLAGALIAEHGKDIQHAPGSNPSSGFSTPKVHPQS--STPKPAA
AN5602.1  	DLVRSKIVPQIRKELVQLAPALVAEHGKDIQHAPGENPSKGFTPAVSYPQTKKQEAPASK
          	* **.*:**::*.*: :*: **:************.***.**:..  .          : 

MG00814.1 	TTAQTNSTGSVVNTTTVTDQEEFRTTAAELYQTFTDPQRLAAFTREAPRVFEGAKKGGKF
NCU04087.1	TTTSSSSSSSAINTTTVVDSAEFRTSADELYATFTDPGRLAAFTRAPPKVFEGAKPGGKF
FG09535.1 	TTSTQSKTGSVVNTTTVTDNEEFRTTAEELYQTFVDPQRIAAFTRSPPKVFDGAKVGGKF
AN5602.1  	PATTTTTNKVSVNTTTVTASDEFRTTAEELFKTFTEPERLAAFTRGQPRQWDGAKVGGKY
          	.::  ...   :*****. . ****:* **: **.:* *:*****  *: ::*** ***:

MG00814.1 	QLFGGNVEGEFLDLQEPTKIVQSWRLRQWPAGHFSKLEIEFDQNDREGVTTMRVNWSGVP
NCU04087.1	VLFDGNVSGEYVELQEPTKIVQKWRLEQWPQGHYSTLKIEFDQNDVDKVTVMRVEWTGVP
FG09535.1 	ELFGGNVSGEYLELEQPKKITQSWRLNQWPAGHFSKLHMEFDQNDVDHVTVMRVKWEGVP
AN5602.1  	SIFDGNVTGEFVKLESPTLLVQKWRLAQWPEGHFSTLEINFDQNDVDGVTQMRVSWAGVP
          	 :*.*** **::.*:.*. :.*.*** *** **:*.*.::***** : ** ***.* ***

MG00814.1 	IGQEEVTKQNWLEYYVRSIKKTFG2LANSVQLRNHSLKPLFSSRWA~EGDEGVCAGGG~G
NCU04087.1	VGQEEIVKNNWNEYYVRSIKRTFG2FGTIL----------------~-----------~-
FG09535.1 	IGQEDVTKRNWLEYYVKSIKQTFG2FGTIL----------------~-----------~-
AN5602.1  	VGQEDVTKQNWELYYVRSIKQTFG2SRSFVFTWRQLLITILTLFLG0HMINLTIFSYS1C
          	:***::.*.**  ***:***:***   . :        .  :   .    .    . .  

MG00814.1 	KAFEGMLQPLTAP
NCU04087.1	-------------
FG09535.1 	-------------
AN5602.1  	HDNV---------
          	
```
