# Supplementary material for: Patterns of Intron Gain and Loss in Fungi
Source: PLoS Biol. 2004 Nov 30;2(12):e422. doi: 10.1371/journal.pbio.0020422 (PMC532390; doi:10.1371/journal.pbio.0020422)
Supplement: Table S1 — Also available at http://genes.mit.edu/NielsenEtAl/. (4.3 MB ZIP). [file pbio.0020422.st001.zip › NielsenEtAl/html/1138.html]

AN3649.1.NCU02757.1.MG05647.1.FG01290.1


```
 CLUSTAL W (1.82) Multiple Sequence Alignments - Introns Inserted


Sequence 1: NCU02757.1	354 aa
Sequence 2: MG05647.1	388 aa
Sequence 3: FG01290.1	372 aa
Sequence 4: AN3649.1	405 aa
Alignment Length: 412 aa
Number Identitical Residues: 160 aa
Alignment Score (without introns) 8416


MG05647.1 	MLQPRLRPWALGGTLSACQTAMRQCLQRGYAMSVQTPPRIVTEPQPDGSVLTFQQEPGLS
NCU02757.1	MLQPQFRP-LLAGTASFAQTVLGRTVARCYATKAATQS---ASSTSTSNTSKDAKAKIVT
FG01290.1 	MLQTGLNF--AAARQSLLRFALNRPLPRTYATIRRSNE-------TTASPKELAENP---
AN3649.1  	MLQHRVAFRGIRLPFRCVSSLLPRSYSTGIEQHTESIS-----VESSSSSIDPSLSFAPP
          	***  .               : :           :         . ..          .

MG05647.1 	KAEPTSTL-MRTYKPRTPGVRHLKRPINDHLWKGRPHLPLTIPKKGQSKGGRNNTGRITV
NCU02757.1	PYVRDAGM-MRTYKPHTPGIRHLKRPINDHLWKGRPYLPLTFPKKGQSKGGRNHSGRVTV
FG01290.1 	--EAQAAI-LRVYKPRSPGVRHLKRPINDHLWKGRPFLPLTYPKKGQAKGGRNVSGRITV
AN3649.1  	PVRQDSTINIRKYTPRTPGVRHLRRPINDHLWKGRPVHKLTFPKRGHSKGGRNNSGRVTV
          	     : :.:* *.*::**:***:************   ** **:*::***** :**:**

MG05647.1 	RHRGGGAKRRIRTVDFDRKDPGPQLVERIEYDPGRSAHIALLTHQETGKKSYIVAAEGMR
NCU02757.1	RHRGGGHKRRIRMVDFERWIPGPHTVLRIEYDPGRSAHIALVKEEATGRKSYIVAADGMR
FG01290.1 	RHRGGGAKRRIRTVDFIRNRPGPHLVERIEYDPGRSAHIALVTEKATGRHTYILAADGLR
AN3649.1  	RHHGGGHKRRIRMVDFKRDAPGPHIVERIEHDPGRSAHIALLRSKETGKLSYILAADGMR
          	**:*** ***** *** *  ***: * ***:**********:  : **: :**:**:*:*

MG05647.1 	AGDILQSYRAGIPQELLDSMGGFIDPGILASKTAFRGNCLPVHLIPSQTMIYCVGSAPDR
NCU02757.1	AGDVVQSYRSGLPQDLLDSMGGVVDPGILAARTCWRGNCLPVSMIPVGTQIYCVGSRPDG
FG01290.1 	SGDIVHSYRAGIPQDLLDSMGGIIDPGILAAKTAFRGNCLPMHMIPVGTTVFGVGSAARR
AN3649.1  	AGDVVQSYMAGIPDDLWQSMGGTVDPGVMAARTAWRGNCLPLHMVPVGSLIFNVGLRPGG
          	:**:::** :*:*::* :**** :***::*::*.:******: ::*  : :: **  .  

MG05647.1 	GAVFCRSAGTYAVIVAK--------DEEVKA-----DGSRVMTG----------KYVTVR
NCU02757.1	KAVFCRSAGTYATIISK--------EEETRE-----DGTKVMTG----------KFVNVR
FG01290.1 	GAVFCRSAGTSAVVVNK--------NEETKD-----DGTRVMTG----------KYVEVR
AN3649.1  	GGQLCRSAGTFATVISKGVNQVAAADQESKESQAAGEEKKPMTQREKQARERTLQHITIR
          	 . :****** *.:: *. .. :::::* : :.::.: .: **  ...: . : :.: :*

MG05647.1 	LQSGEIRRISKDACATVGRASNPMHQYRQLGKAGRSRWLNIRPTVRGVAMNSS1DHPHGG
NCU02757.1	LQSGEIRRVSKDACATVGIASNIMHHYRQLGKAGRSRWLNIRPTVRGLAMNAN1DHPHGG
FG01290.1 	LQSGEVRRVSKDACATIGVASNIHHHYRQLGKAGRSRWLNIRPTVRGVAMNKV1DHPHGG
AN3649.1  	LSSGEVRLIHKDCCATIGVASNPNKKYAQLGKAGRSRWLNIRPTVRGLAMNAQ~DHPHGG
          	*.***:* : **.***:* ***  ::* *******************:***   ******

MG05647.1 	GRG~KSKGRRHPTSPWGTL0AKGGFKTRRTNNPNRWVVVPRERNMGKRRAKKSSS
NCU02757.1	GRG1RQ-------------~FEGSVYHRKDAMCGYWAQ-----------------
FG01290.1 	GRG~KSKGNRHPVSPWGVP0TKSGYKTRRKHNRNKWVVVPRPRNNGKRRDKAN--
AN3649.1  	GRG~KSKGNVDPKSPWGLP0AKSGYKTRPKWKINKAVVVPRVRNQGKRRRGYH--
          	*** :...   . :. .    :..   *     .  .  .   . ..
```
