# Supplementary material for: Patterns of Intron Gain and Loss in Fungi
Source: PLoS Biol. 2004 Nov 30;2(12):e422. doi: 10.1371/journal.pbio.0020422 (PMC532390; doi:10.1371/journal.pbio.0020422)
Supplement: Table S1 — Also available at http://genes.mit.edu/NielsenEtAl/. (4.3 MB ZIP). [file pbio.0020422.st001.zip › NielsenEtAl/html/1139.html]

AN0067.1.NCU07887.1.MG06408.1.FG05409.1


```
 CLUSTAL W (1.82) Multiple Sequence Alignments - Introns Inserted


Sequence 1: NCU07887.1	410 aa
Sequence 2: FG05409.1	399 aa
Sequence 3: MG06408.1	403 aa
Sequence 4: AN0067.1	406 aa
Alignment Length: 416 aa
Number Identitical Residues: 265 aa
Alignment Score (without introns) 11521


MG06408.1 	-MIETTPSKQ0AASAIESFKM-ESPVKKLDFNVADKENQPHTES--VAEETVEKPLAKDV
NCU07887.1	MSVQTSPSKQ0VTSGIQNLNM-DSPAKKLDFGATDKENKPFDEDLAKLEAEIDAEHNANK
FG05409.1 	MAAQMTPSKQ~AASGIENLNM-ESPVKKLNFGTANKENEP-------LNQTTDTAELKTK
AN0067.1  	MSAQLTPSKQ0AASSLENLKMNDSPVKKLNFEAAGKENAPVSN----PMVDVPATKPATE
          	   : :**** .:*.::.::*.:**.***:* .:.*** *  .                 

MG06408.1 	KPVEKPAVAPTIKAFEMDEPILQENPQRF0V--------~-WQMYKKAEASFWTAEEIDL
NCU07887.1	KAAEAKKMAPTLKPEEANEPLLTENPQRF~VLFPIKYHE0IWQMYKKAEASFWTAEEIDL
FG05409.1 	IVEESKTVVS--KAEED-EPILQENPQRF~VLFPIKYHE0IWQMYKKAEASFWTAEEIDL
AN0067.1  	KPVEPSKAALDVKAIEANEPLLQENPHRF~VLFPIKYHE0IWQMYKKAEASFWTAEEIDL
          	   *    .   *. * .**:* ***:** *  . .  .  *******************

MG06408.1 	SKDLHDWNNKLTDDEKYFISHILAFFAASDGIVNENLVERFSAEVQIPEARCFYGFQIMM
NCU07887.1	SKDLHDWNNRLNDDEKFFISHILAFFAASDGIVNENLVERFSGEVQIPEARCFYGFQIMM
FG05409.1 	SKDLHDWNNRLTSDEQYFISHILAFFAASDGIVNENLVERFSGEVQIPEARCFYGFQIMM
AN0067.1  	SKDLHDWNNRLNEDERYFISRVLAFFAASDGIVNENLLERFSGEVQIPEARCFYGFQIMI
          	*********:*..**::***::***************:****.****************:

MG06408.1 	ENIHSETYSLLIDTYIKETAQRTYLFNAIDTI1PCIRKKADWALRWITDKKSTFATRLIA
NCU07887.1	ENIHSETYSLLIDTYIKEPSQRTYLFNAIDTI~PCIRKKADWALRWITDKSSTFAQRLVA
FG05409.1 	ENIHSETYSLLIDTYIKDPAQRTYLFNAVDTI~PCIRKKADWAIRWIQDKNSTFAQRLVA
AN0067.1  	ENIHAETYSLLIDTYIKEPKQRTYLFDAIDTI1PCIRKKADWAIKWIQDKESTFAQRLVA
          	****:************:. ******:*:*** **********::** **.**** **:*

MG06408.1 	FAVVEGIFFSGAFASIFWLKKRGLMPGLTFSNELISRDEGLHTDFACLLFSLLNNRPNKE
NCU07887.1	FAAVEGIFFSGAFASIFWLKKRGLMPGLTFSNELISRDEGLHTDFACLLFSHLNNRPSKQ
FG05409.1 	FAAVEGIFFSGAFASIFWLKKRGLMPGLSFSNELISRDEGLHTDFACLLHSHLKGRASKQ
AN0067.1  	FAAVEGIFFSGSFASIFWLKKRGLMPGLTFSNELISRDEGLHTDFACLLFSHLNWRPSKK
          	**.********:****************:********************.* *: *..*:

MG06408.1 	VVKEIVVDAVKIEQEFLTEALPCALLGMNANLMKQYIEFVADRLLVALGNEKVYRATNPF
NCU07887.1	LIQEIIVDAVRIEQEFLTEALPCALLGMNADLMKQYIEFVADRLLVALGNEKIYRSTNPF
FG05409.1 	MIQDIITDAVSIEQEFLTEALPCALLGMNSNLMKQYIEFVADRLLVALGNEKVYKSTNPF
AN0067.1  	VVEDIIVEAVAIEKEFLTDALPCALLGMNAKLMCQYIEFVADRLLVALGNKKYFNATNPF
          	::::*:.:** **:****:**********:.** ****************:* :.:****

MG06408.1 	DFMENISLGGKTNFFEKRVGEYQKAGVMASTKKAPVEEKTSTSPQAEDSNSGDFTFDDDF
NCU07887.1	DFMENISLGGKTNFFEKRVGDYQKAGVMNSTKKADADAEVAKN-----ENGGDFTFDEDF
FG05409.1 	DFMENISLGGKTNFFEKRVADYQKAGVLHSANKKDEE-EAPKG-----ENGGDFTFDDDF
AN0067.1  	DFMESISLAGKTNFFEKRVGDYQKAGVMASTKKDPKQDETKTS------DGNGLSFDEDF
          	****.***.**********.:******: *::*   : :. ..      :...::**:**

NCU07887.1	
FG05409.1 	
MG06408.1 	
AN0067.1  	
          	
```
