# Supplementary material for: Patterns of Intron Gain and Loss in Fungi
Source: PLoS Biol. 2004 Nov 30;2(12):e422. doi: 10.1371/journal.pbio.0020422 (PMC532390; doi:10.1371/journal.pbio.0020422)
Supplement: Table S1 — Also available at http://genes.mit.edu/NielsenEtAl/. (4.3 MB ZIP). [file pbio.0020422.st001.zip › NielsenEtAl/html/114.html]

AN5586.1.NCU06003.1.MG01288.1.FG08773.1


```
 CLUSTAL W (1.82) Multiple Sequence Alignments - Introns Inserted


Sequence 1: NCU06003.1	364 aa
Sequence 2: FG08773.1	364 aa
Sequence 3: MG01288.1	363 aa
Sequence 4: AN5586.1	351 aa
Alignment Length: 364 aa
Number Identitical Residues: 251 aa
Alignment Score (without introns) 11488


MG01288.1 	MKA1IILVGGFG-KFCPK0TLTLPKPLVEFANKPMILHQIEALSAAGVTDVVLAVNYRPE
NCU06003.1	MKA1LILVGGFGTRLRPL0TLTMPKPLVEFGNKRMILHQIEALAAAGVTDIVLAVNYRPE
FG08773.1 	MKG1LILVGGYGTRLRPL0TLSVPKPLVEFANKPMIVHQIEALVAAGVTDIVLAVNYRPE
AN5586.1  	---~----------MRPL0TLTLPKPLVEFGNRPMILHQVESLAAAGVTDIVLAVNYRPD
          	              : *  **::*******.*: **:**:*:* ******:********:

MG01288.1 	IMEKALAE0YSKKFNINITFSVETEPLGTAGPLKLAEKTLLKDDTPFFVLNADVTCEYPF
NCU06003.1	IMEKYLAE0YEKQFGINITISIESEPLGTAGPLKLAEDVLRKDDTPFFVLNSDVTCEYPF
FG08773.1 	VMEKFLAE0YEEKFGINIEFSVETEPLDTAGPLKLAERILAKDDSPFFVLNSDVICDFPF
AN5586.1  	VMVSALKK0YEEQYNVKIEFSVETEPLGTAGPLKLAESILAKDDSPFFVLNSDVICDYPF
          	:* . * : *.:::.::* :*:*:***.*********  * ***:******:** *::**

MG01288.1 	KQLADFHKAHGDEGTIVVTKVEEPSKYGVVVHKPNHPSRIDRFVEKPVQFVGNRINAGIY
NCU06003.1	KELAAFHKAHGDEGTIVVTKVEEPSKYGVVVHKPNHPSRIDRFVEKPVQFVGNRINAGLY
FG08773.1 	EDLLAFHKSHGNEGTIVVTKVEEPSKYGVVVHQPGHRSLIDRFVEKPVEFVGNRINAGLY
AN5586.1  	QQLAEFHKRHGDEGTIVVTKVDEPSKYGVVVHKPNHPSRIDRFVEKPVEFVGNRINAGMY
          	::*  *** **:*********:**********:*.* * *********:*********:*

MG01288.1 	ILNTSVLSRIELRPTSIEQETFPAMVRDAQLHSFDLEGFWMDVGQPKDFIAGTCLYLSSL
NCU06003.1	IFNPSVIDRVELRPTSIEQETFPAMVRDGQLHSFDLEGFWMDIGQPKDFLTGTCLYLSSL
FG08773.1 	IFNTSILDRIELRPTSIEKETFPAMVKDNQLHSFDLEGFWMDVGQPKDFLSGTCLYLSSL
AN5586.1  	ILNPSVLKRIELRPTSIEQETFPAIVRDGQLHSFDLEGFWMDVGQPKDFLTGTCLYLTSL
          	*:*.*::.*:********:*****:*:* *************:******::******:**

MG01288.1 	AKRNSKLLTPTSEPFVHGGNVLIDPTAKIGANCRIGPNVTIGPNVVVGDGVRLQRCVLLR
NCU06003.1	TKKGSKELAPTTLPYIHGGNVLIDPSAKIGKNCRIGPNVTIGPNVVVGDGVRLQRCVLLE
FG08773.1 	TKKGSKELTSPSEPFVHGGNVLIDPSAKIGKNCRIGPNVTIGPNVVIGDGVRLQRCVLLK
AN5586.1  	TKRNSKLLAPNSEPYVYGGNVMVDPTAKIGKNCRIGPNVVIGPNVVIGDGVRLQRCVLME
          	:*:.** *:. : *:::****::**:**** ********.******:***********:.

MG01288.1 	DSKVKDHAWIKSTIVGWNSTVGRWARLENVTVLGDDVTIGDEIYVNGGSILPHKSIKANV
NCU06003.1	GSKVKDHAWVKSTIVGWNSTVGKWARLENVTVLGDDVTIGDEIYVNGGSILPHKTIKANV
FG08773.1 	GSKVKDHAWVKSTIVGWNSTIGRWARLENVTVLGDDVTVGDEIYVNGGSVLPHKSIKANV
AN5586.1  	NSKVKDHAWIKSTIVGWNSSVGRWARLENVTVLGDDVTIADEVYVNGGSILPHKSIKQNI
          	.********:*********::*:***************:.**:******:****:** *:

MG01288.1 	DV~PAIIM
NCU06003.1	DV~PAIIM
FG08773.1 	DI~PAIIM
AN5586.1  	DV1PAIIM
          	*: *****
```
