# Supplementary material for: Patterns of Intron Gain and Loss in Fungi
Source: PLoS Biol. 2004 Nov 30;2(12):e422. doi: 10.1371/journal.pbio.0020422 (PMC532390; doi:10.1371/journal.pbio.0020422)
Supplement: Table S1 — Also available at http://genes.mit.edu/NielsenEtAl/. (4.3 MB ZIP). [file pbio.0020422.st001.zip › NielsenEtAl/html/1140.html]

AN6095.1.NCU01231.1.MG03360.1.FG05882.1


```
 CLUSTAL W (1.82) Multiple Sequence Alignments - Introns Inserted


Sequence 1: AN6095.1	514 aa
Sequence 2: FG05882.1	516 aa
Sequence 3: MG03360.1	513 aa
Sequence 4: NCU01231.1	518 aa
Alignment Length: 527 aa
Number Identitical Residues: 308 aa
Alignment Score (without introns) 13959


MG03360.1 	-MASPSQNEPADPIARGVFPAAKQAFGDLFKWKQRVVVTNEVGEQHTEWQSPAPLKNPIS
NCU01231.1	---MESTHEPADPVAKGILPTARQSWKDLFIWKQRVVVTNVYGETATEWAKPVPLKNPIS
FG05882.1 	MVQQPMAGESAGPVVKGVVPTAKQAFADLFIWKQRVVVTNEHGEETTEWRDPDPIQNPIS
AN6095.1  	---MPSQNEPAEPIPDGIFAVAKQSWGDLFRWKQRVIIENEQGESYAEWQDPEPFKNPIS
          	        *.* *:  *:...*:*:: *** *****:: *  **  :** .* *::****

MG03360.1 	LMAQLSAKDWLFFIVGFAAWSADAFDFHALSIQTKKLADYYGTTKTEITTAITLTLLLRS
NCU01231.1	LLAQLSGRDWICFLVGFCAWSADAFDFHALSIQQVKLAAYYGVSKTSVSTAITLTLLLRS
FG05882.1 	LMAQLSAKDWIFFLVGFCAWTADAFDFHALSIQTKKLSLYYDTSKTAITTAITLTLLLRS
AN6095.1  	LLMLLSARDWLFFLVGLAAWTADAFDFHALSIQQVKLADYYGKTKTDISTAITLTLLLRS
          	*:  **.:**: *:**:.**:************  **: **. :** ::***********

MG03360.1 	VGAAIFGLAGDKWGRKWPMVVNMIVLGILQIATIYSTTFQQFLAVRALFGLFMGGV2YGN
NCU01231.1	IGAAAFGLAGDRWGRKWPMVVNMIVLGILQIATIYSSTYSQFLGVRALFGLFMGGV2YGN
FG05882.1 	VGAAMFGLAGDKWGRKWPMVFNMIILGILQIATIYSTTFNQFLAVRSLFGLFMGGV~YGN
AN6095.1  	VGAAFFGLAGDRFGRKWPMVINMIVLGVLQIATIYSSTFQQFLAVRSLFGLFMGGV~YGN
          	:*** ******::*******.***:**:********:*:.***.**:********* ***

MG03360.1 	AIAMALENSP~-----VDARGLMSGILQQGYSFGYVCAACANLGVGGATDSWKTVFWIAA
NCU01231.1	AIAMALENSP~-----VDARGLMSGILQQGYAFGYVCAACANLGVGGDTDSWKTVFWIAA
FG05882.1 	AIAMALESCP2-----SNARGLMSGILQQGYSFGYVLAACANLGVGGSTESWKTVFWIAA
AN6095.1  	AIAMALEHCP2LIARSVSARGLMSGILQQGYSLGYVFAACANLGVGGGTETYKTVFWIAA
          	******* .*   : : .*************::*** ********** *:::********

MG03360.1 	~GISIGVGLIRCCFPESQQFIEARKLGKNKANPVAFWEETKKMLAAEWKLCLYCIVLMTW
NCU01231.1	~GLSIGVGIIRCFFPESKQFLEARKEGKAHANPSQFWRETKVMLRQEWKMCVYCCILMTW
FG05882.1 	1GISIAVGIVRIFFPESQQFLEARAKGKQSASPGAFWQDCKKMLLAEWKMCVYCCFLMTW
AN6095.1  	1GISIGIGLIRVLFPESKQFLEAKAAGKRSVSAGEFWRETKVMVGQEWKICVYCIFLMTW
          	 *:**.:*::*  ****:**:**:  **  ...  **.: * *:  ***:*:** .****

MG03360.1 	~FNF1Y~SHTSQDSYTTFMLSEKELDNAGASRASILMKAGACVGGTIIGYVSQWLGRRRA
NCU01231.1	~FNC~N2-HTSQDNYTTFVLRAKEMDNSAASRASIIMKAGACVGGTIIGYLSQYFGRRRT
FG05882.1 	~FNY~Y~SHTSQDSYTTFMLEQKEFKNEAASRASILMKTGACVGGTIIGYLSQFVGRRRA
AN6095.1  	0FNY~Y~SHTSQDSYTTFMLTQKELENSGASRASILMKTGACVGGTIIGYLSQFVGRRRA
          	 **    :*****.****:*  **:.* .******:**:***********:**:.****:

MG03360.1 	IILSALISACLIPAWILPAGERSLSASGFFMQFFVQGAWGVIPIHLNELSPAAFRSSFPG
NCU01231.1	IIVSSLISGCMIPAWILPNSERALSATGFFMQFFVQGAWGVIPIHLNELAPPAFRSSFPG
FG05882.1 	ICISALMSALMIPAWILPTTEGGLSASGFMIQFFIQGAWGVIPIHLNELSPPAFRSSFPG
AN6095.1  	IIISAFVSGLIIPAWILPTTERSLSATGFFMQFFVQGAWGVIPIHLNELSPPAFRSLFPG
          	* :*:::*. :*******  * .***:**::***:**************:*.**** ***

MG03360.1 	~ITYQLGNMISSPSAQIVNAIAETNFIRNHKGDIVEAYGPTMGIATAIIAMGIATWTAFG
NCU01231.1	~ITYQVGNMVSSPSAQIVNAVSEKIHIVSHTGKLVEAYGPTMGIATAIIVMGIVVTTAFG
FG05882.1 	~VTYQIGNMISSPSAQIVNAVAEKTFVTLKNGDKVEAYGPVMGVATAIIAMGIIFTTMFG
AN6095.1  	1LTYQLGNMISSPSAQIINAIAEKTFIKGPSGNPVEAYGPTMGVATAIIATGIMVTTAFG
          	 :***:***:*******:**::*. .:   .*. ******.**:*****. **   * **

MG03360.1 	PERKGRKFEQELPAGIADVPREKSIDDDLEAGHGGEKPDATT---AEKV-----
NCU01231.1	PEKRGREFEKALPAGMNLQKQHGKQVDDLEMETGHMEKVTSELDDEKREGRAVQ
FG05882.1 	PEKRGRSFEHAV-AGVRNDELPHHQKKDIETASVEQVEMDERKKAKEEV-----
AN6095.1  	PEKRGRRFETVV-VGMQ-EQNRTDKQLDLEADSKPGEETVER---VDKV-----
          	**::** **  : .*:           *:*                ..
```
