# Supplementary material for: Patterns of Intron Gain and Loss in Fungi
Source: PLoS Biol. 2004 Nov 30;2(12):e422. doi: 10.1371/journal.pbio.0020422 (PMC532390; doi:10.1371/journal.pbio.0020422)
Supplement: Table S1 — Also available at http://genes.mit.edu/NielsenEtAl/. (4.3 MB ZIP). [file pbio.0020422.st001.zip › NielsenEtAl/html/1141.html]

AN5221.1.NCU03103.1.MG03693.1.FG00672.1


```
 CLUSTAL W (1.82) Multiple Sequence Alignments - Introns Inserted


Sequence 1: NCU03103.1	1483 aa
Sequence 2: MG03693.1	1426 aa
Sequence 3: FG00672.1	1383 aa
Sequence 4: AN5221.1	1527 aa
Alignment Length: 1674 aa
Number Identitical Residues: 294 aa
Alignment Score (without introns) 16685


MG03693.1 	MPPQGDVTLPATKRARKSLSGLSPSRRAG--EKENATVDVSA-------ASLAANRKKSR
NCU03103.1	MTSQQDVTLPPTRIPRKSISGGSPTKKRA--EKDNATVDIG---------AMGAGRKKMR
FG00672.1 	MPIP---EIPPLKPRQSKIN----NSREP--TQEFSTPTLS-------------------
AN5221.1  	MASRGDSAESTRVRSRRSIAHVPRSRLTAGGDKDNATTEISTSQPLANRRSATGKEKKSR
          	*.   .   ..    : .:   . .    .. :: :*  :.::.. :.  :  .  ..  

MG03693.1 	SKSIGPGGLDALKPANGNRRQ0SIAAP---LPRSILKPTIPILPEIPPHKPKQATSRNEG
NCU03103.1	SKSMGPGSLDIFKSGNGNRRA0SLAVPSRPPPRSILKPTS--LPEIPTFKSRQAGSQVSG
FG00672.1 	---------------------~--------------------------------------
AN5221.1  	SKSLGPGGLDALQTSNGNRRK0SAAVFPLKSILKPTAPVSPIRNIPTFEETRRKTPARGG
          	:.: .... .  ......    : :  .     .   .  .     .  ..    .   .

MG03693.1 	----------------DS----TKVALRTE--------------EEQQAAAREREERERV
NCU03103.1	TDQSTSS--------FDSGEGGTRVAVRTE--------------EEQQAAAREREEKERA
FG00672.1 	---------------------GSKIAIKTE--------------EEQQAAAREREERE--
AN5221.1  	KEQASSTGLAQGGNLIDLDTHAQKSAVGTDGQTNPFDNFNADTIRDEMAAAREQEEKERR
          	...::::. :....  . .  . : *: *:..:.. .. .:.: .:: *****:**:*  

MG03693.1 	ALE-KEIKDRREARRK---~---SLANRRVSFAAEATLHTFHEIEYAQDSTTS--TDSTR
NCU03103.1	QVE-KDNNERREARRK---~---SLANRRVSFAAEATLHTFHEIELPQDSTTS--TDTTR
FG00672.1 	---------RRDARRK---~---SLANRRVSFAAEATLHTFHEVEFNQDSTTS--TDSTR
AN5221.1  	ERERKAALEHREARRKSME1LTGLQANRRVSFAPEATLHTWNVVEIPEDSTSSSASNSTR
          	  . .   .:*:****: .  :.  ********.******:: :*  :***:*:::::**

MG03693.1 	RASS-LAAPSPAPHATQQESNPAEPPSTPPEHVDDVVPDSPADQRELHQKKR---RRSSG
NCU03103.1	RASSSTAAPSPAP-STLEAPKPLPSP------------DNAEDDTIAYDSDL---EHADS
FG00672.1 	RNSTAAQQREREEEDKKNRRSSGPPQIDFHE-------SDDDTATTLYSSDS---EPADA
AN5221.1  	RVSALTNTPNQPMHTSKQSDRSSSPDIDAESDIAFSPVQYPDLERLRNQQPIGSYDGASS
          	* *:     .     . :   .  .     .       .         ..  .:   :..

MG03693.1 	ILPLDENDITMGSTVYDSDSDNGDDIIEDPDEGESDSDAG-------DGTMMTLDADEVT
NCU03103.1	VAEIQAEEMTGSS---------------------DDSDVE-------DGTMMTVEAEELT
FG00672.1 	VEEVADVEEDGDD---------------------DSSESD-------DGTMMTID--DVT
AN5221.1  	SQEMLSSPFSGSENSEDTGLHSIARDDGNDEEEEDDDDDESSTASGFDGESTAMSMDDMS
          	   :       ...  .:.  .      . .. ....:  :::::. **   ::. .:::

MG03693.1 	TASAVSAGSMSIMSSDS-TASIDEMLRLATQRAGARSAADE----------DEEVIPAFI
NCU03103.1	SASIATP--RSHFSADS-SGDLDENLRLAARRA-VTQRLDE----------DEEVIAGFA
FG00672.1 	GTTAASD--RSVDSDDGESSTLDEALRLAARRAGNQQLRDDEDD--DDLDDDEEFIPSFG
AN5221.1  	IHSGVTTQTDGSESTSG-TNRLNEALRQAAREAGTRSFEDENDEEVSMEIADQEITGAFQ
          	  : .:  : .  * .. :  ::* ** *::.*.  .  *:.... .    *:*.  .* 

MG03693.1 	GWGKK-AAPGSGMQ--EDQEIFS--SPVRSVVDE-------DDGMDMEETHAIGGIVR--
NCU03103.1	GWGKKNIAQGSSSQG-SGQHTHSNLTQADQVPDT-------DMDADMDMTTAVGGIIR--
FG00672.1 	-WGKDANKQNIAAQNQENQPPPARPQPKQDLQDATETNMSMDLDMDMDITRVVGGILKPQ
AN5221.1  	PWIKKGQRQSFDWEDISARHDQENVDPSKSMNSATS------------------------
          	 * *.    .   :. . :          .: . :.                        

MG03693.1 	---------AVDADGT----------MASE-DGMDMSMDMTHALGGIMAQ----------
NCU03103.1	---------RMSSPSP----------EGDDGDDMDMSMDVTKAIGGIIAQPIPSTIQEHS
FG00672.1 	LTQQYDPDEDMSMDVTRVLGGILKRQSQEHNPDEDMSMDVTRAFGGIVNQP------QNA
AN5221.1  	-------------------------EMASDNGDEDLSMEVTNAIGRIIPNN--------R
          	                         .  ... . *:**::*.*:* *: :          

MG03693.1 	----KRAAQREATMCVDQTMDFTRPMGGIQS---TADDEVEQQDGEGYEDMSMELTTVMG
NCU03103.1	ARPGSRSLEVSTTSRGDRTMELPTAVGGIRH---SRVSDVSQFDTDANEDMSMEITTAIG
FG00672.1 	SAPIDDQEDEDDAPMEEATMEFTTAIGGIQR---PAPIEEDD-DSDGNEDMSMELTTVIG
AN5221.1  	QSLGRRRSIAEETNYEEQTMELTNVVGGIAQSVSPAKSADANSEIDNDEEMTMEFTSVVG
          	          . :   : **::.  :***  : :.      : : :  *:*:**:*:.:*

MG03693.1 	GVWATGAAKGGKPGARR----HTTMRLDDADAEQTVGMDMTVGLGKIIT----TDKDDEG
NCU03103.1	AVLG-GTSFGNAPQESR----PTTNSGTNDDEDEASMMDMTVSVGQILR----DRPGNEA
FG00672.1 	GVLAKNKRK--TIAASR----RRSASQPDDENDDEAPMDMTLAVGQILS----KPDDDDS
AN5221.1  	GVLNRAGSQKVDEDNDTPDHGDNSTYDNPDDMDDGADMEITGAVGEILPRVQEEAEFGDG
          	.*               .. .  :      : ::   *::* .:*:*:   ..    .:.

MG03693.1 	DG--EMDMTAGMD-----MSPVALGGIIASVTRPADENEQTPQGYRSAEKAPPVNSRQSS
NCU03103.1	NDTQAMDITAVVGGIIKPAPTPQQGSTSQSITGPTAKENIRPLTSTAVIRTSPKRRRSAV
FG00672.1 	EDNADEEGDATMG----MDMTTAIGGILNKVTGGSRDLGKRVMEEEADSAANPDEAIEAA
AN5221.1  	DQTTGMDFTAAMG-----KILTPERASPDKVHSQPASSPFQESVRASPAKSPAAFHVAAV
          	: .   :  * :.            .   .:   . .         :   : .     : 

MG03693.1 	PSRPSS-----PIKNSLKSILNRNPLPDRS-------PGRSPSRSPERSASND---SGIR
NCU03103.1	VDENGS-----PGLAAFQNNGLRQSLPPASSMAAGGLSNDAPSSPLQSSPLRS---SPTR
FG00672.1 	ISKSAVSHFLQPPTASEKQTTTPSPRPSRSPASALRNTANPKIPTPQRTTRNSRTPSPVK
AN5221.1  	ASESGS-----PSLASVRSRPTRQSLSRATPTTPTSITPQEPPAQNSSKTLKQSARAGQP
          	 .. .      *   : :.    .. .  :. :.   .        . .. .. : :   

MG03693.1 	KSPGRNSPAKPSIQTAKSP-----EQPRTPLRSSPSRFIASGSKSPVR--SAGQNNKQAP
NCU03103.1	ILPTRSSPDRRPLARQPSPGKAPETQLGTPVSKPAPRRIGSRSSSPIR--AASPMAKITP
FG00672.1 	ATTTPRSAKVRTPASAKPATPQLESVVSERTATPRSTQRISRSASPKRPPSARTTRANSR
AN5221.1  	STPEHPSPFKDEGIRNASP---------KKIFQPEIQASHSQQKSPGR------------
          	  .   *.         ..              .      * . ** *            

MG03693.1 	VSPAKQRSPTRSLFQQDPATGTTTPLVALTPQRRRLSGVGADRLGLGSPRVAELLERRTS
NCU03103.1	LSLKS------KLFRQDPSTGLNTPRIVLTPQNRRLSGVGADRPGLGSPKVAEIIDRRDS
FG00672.1 	TPSPVKSTPKKSLFQNNPRNGSRTPTVTLTPQ-RRLSGLGADRPGLGSPQVTALFDRRGS
AN5221.1  	----------RSLFGSNAAGESAPLFVLRPPGPRRSSGIGIDREGLGSPKVAAMLDKRRS
          	           .** .:.     .  :  .*  ** **:* ** *****:*: ::::* *

MG03693.1 	IGEAAKEFSPGRNLELGRRGVKWDNPRIMEQEIDRQRQQEMDKEDGRKIMEREAD-ERDV
NCU03103.1	IGEAAPDFIPSQ-PDGQRRAVAFADPRVMEAELDKERREEEERENSRRILERETDGERDP
FG00672.1 	IGDMATSFVPGK------RGVMFEDPKEMTQDMDREAREEEEKENRRKILEREAD-GVEA
AN5221.1  	IGEEAGDFVPRP------QGVRFEDPIKLQEEVDREREEEESREDGHIQPP-------DP
          	**: * .* *        :.* : :*  :  ::*:: .:* .:*: :           : 

MG03693.1 	TVNLREMIQGLSPKKNPLRGRKSLHVGSAKGLLGKRPSELDDGEEE----KDGVKRLKNH
NCU03103.1	TVNLMEMIQGLTPKKKPLRGRKSLAVGSAKGLLGKRPVELDQDIDEDTPEKDGVKRLKGH
FG00672.1 	TLNLREMIDSLSPKRKPLKGRKSLHVGSAKGLLGKRPNELDD--EEEAEENYSVKRLKGH
AN5221.1  	TASLKDMISSLTPKKNKLRGRKSLHVGAARGILGKRPAELDLEDEDEG--ENTPKRLRRR
          	* .* :**..*:**:: *:***** **:*:*:***** ***   ::.   :   ***: :

MG03693.1 	HGSPVKSIKLQQPPSKEETTGRLSKSSRSQDSTKQSLEVSTPKSPIKISRTAPSPRRLSH
NCU03103.1	QGSPVKNIRLQAPPSKAETTTGRETVSESVDQTSN--NTVTPTIPSSPTQTMT-PRNQGR
FG00672.1 	QGSPVKNIRLQQPPSKEETTGRLNLSFRKSLLSST--VTPTLSSPAK-SDAATTPRHQGR
AN5221.1  	EDSPVKNVRLPPPPSKEETVGRARSPARKSMALSPSKVSTTPTQEPRVLALENSAQDASK
          	..****.::*  **** **.       ..    . :    * .          :.:  .:

MG03693.1 	FRDVEDDG---AIDLGRG------EGIDAEDLD-DDDEERISLQDFLNMTSIRFMELTTT
NCU03103.1	FKDVANDQPTITMDFDHT------GHIDANAEPRDDDGERIHLQDFLNMTSIRFMELTTT
FG00672.1 	FKDVADDRLGHQVNFDETPVKDIEQIEDEVEAAEEADGDRIHLQDFLNMTSIRFMELTTT
AN5221.1  	AASPDVEVP----------------TEGGADDNHEPEFEPIHLQDFLNMTNIHFMELTTT
          	  .   :                    .      : : : * ********.*:*******

MG03693.1 	KRRHTVAPQAKDNSGSD-------GKDDMSFERCVVAGACTVPMLELYQH~SCRELKKYI
NCU03103.1	KRRHTIAPGASRDSTSA-------EDKDVTFESCVVAGACTVPMLELYQH0SCRELKKYI
FG00672.1 	KRRHTQAPGTLENGFLDE------GEEDLSLERCVVAGACTVPMLELYQH~SCRELKKYI
AN5221.1  	KRRHTTAPDSISKRAARLSLEGDGKSSASNFDDCVAAGFCTVPMLELYQH0SCRELKSYI
          	***** ** :  .     : .... ..  .:: **.** *********** ******.**

MG03693.1 	SEGRDIVREIESETLADNPPLFREYMSASPDFRVLMDNQFKNVKLHARLLSKAMWYEWRM
NCU03103.1	SEGRRIVRDIETETFVENPPLFKEYISATPELKLLMDNQFKNVKSHARLLSKAMWYEWRM
FG00672.1 	SEGRRIVKEIENDTFEDNPQLFKEYMAATPDVKTLMDKQFMNVKNHARLLSKAMWYEWRM
AN5221.1  	SEGRQIIRSIETETYADNPPLFREYMAAAPDIRLLMDNQFRNVKTHTRLLSKATWYEWRM
          	**** *::.**.:*  :** **:**::*:*:.: ***:** *** *:****** ******

MG03693.1 	KLQEGLKEGLDKTAEGMAQDEQVLKKKQALIDSTLPALVEKFAELEKEHGLLDEAVRELA
NCU03103.1	KLQEGLKEGLFKISEGMDKDDELLRKQQELLSSVLPSLTKRYGALERELENLEAVEKELE
FG00672.1 	KLQEGLKQGLLSIDEGMEADKELLDKQKTLLDSVLPAIMDRYKSLVEESDNLEEVARELA
AN5221.1  	KLLEGLKEGLDRHVEEMKGDDNLLSKHEAILKDAMPALSAKHSSLKEEAAQLQQLADELE
          	** ****:**    * *  *.::* *:: ::...:*::  :.  * .*   *:    ** 

MG03693.1 	DCDPDELQAARDELLTATQDIDLKKQQIAELEQDLQQSEEAIGELTTKKQLCLNEIREAE
NCU03103.1	DCDPEDLEAARAELTELDKTIAEKSKKIEELRQQVEEHQTGVQSLADQKQQCLDDITAAD
FG00672.1 	DCDPADLDAARDELASLDEDVEYKKKRIAELREQFQASEVEVEDLNEQKQNYIDDIAESE
AN5221.1  	NCDQDELWNARGKLSDLEEEIAAKRQVLEELQTQIQDKTDTIETGTELKAEVMAQIQEAE
          	:**  :*  ** :*    : :  * : : **. :.:     :      *   : :*  ::

MG03693.1 	RVREECRGWRPDEIMDLRA1RVEAIEKEHGWTVTGCSDT----TISMTYNREIELVFNPT
NCU03103.1	KIREECRGWSLTEISSLKA1RVDELEEKSGWAINKIEAS----VMFMTYKREIELAFDLA
FG00672.1 	RIREECRGWTSKEVNSLKA1RVDTIEKQHGWAVTGISGT----VLSMSYKREIEIVFDIT
AN5221.1  	RVKEECRGWSAKEIRELKD1SVHRIEQRTGWSISSASSSETGLAVTMVYRHQLQLKFYPA
          	:::******   *: .*:   *. :*:. **::.  . :.:. .: * *.::::: *  :

MG03693.1 	SFAIPNSA----ASKQPTNIDLWYIAANRE--RDPVPVTTELEFFLQCIRDQVRALDQPR
NCU03103.1	SFKQQN--------KQGPTIDIRYIAHKRE--RNVIPLTPERNFFIECISSHLQALSKSS
FG00672.1 	AFQDHQ---------PNSTIDLWYIAVNRE--KNPLPKTAEKEFFLQTIRDYARALPHNR
AN5221.1  	SFYIEGESKDSLSQKENTPIELCYSPEKESGPSHARSLSPIMLLILKSLQNHIATITQSE
          	:*    .:..: :..  . *:: * . :.... .  . :.   :::: : .   :: :  

MG03693.1 	TSLRHMLDVVGACWLEAKQVSANIRSINCTFPTRVSRTSDTSMVVKATLLLSGLKTKVEV
NCU03103.1	LSVNRLLTIVSDAWDKADAAAEQIRLLNLSFPTKVTRTSDTTLEVRSSLLLAPLQTRVEV
FG00672.1 	TEASHLLRSVQAAWDKANFVSSQVERLNATFPTTVQRTSDSSISITTSILLVPLESRVEV
AN5221.1  	LTSKQLLRFVSKAWDLATKAEEEARMLGFHGVTNLQLSELEKPSLRARCTLLGTVSPPID
          	    ::*  *  .*  *  .  : . :.    * :  :.  .  : :   *    :    

MG03693.1 	LIDLS-------GQSASGTVVKPRARVVYGQRFNEERMADFLSKEIGDKVG---------
NCU03103.1	CLALEKIF-NNKNSDALDVRVTSDAKVLYGEQFTKSKLNDFLKNKLGKQVLGRKEQQQAR
FG00672.1 	KLSLS----TQRDGGGLEMILKPEAKVVYGEHFNVPKVADFLATRIGKTVG---------
AN5221.1  	STPLSKSQKRSNENCRIDVDFAVTTCIVARDDGNALGVLDIQTEVIASKVYG--------
          	   *..  . .         .   : ::  :  .   : *:    :.. * .        

MG03693.1 	------KGQMWGEVVVELYAKLLSGSNKQ-----------------------------
NCU03103.1	GNRKKQQQQEWSEVVLELHKRLMAKGAKVGHSGQTQLANGSANTQHLQGPQDQGVALK
FG00672.1 	-----AGEEQWGDVMVELQGRLIARGRKA-----------------------------
AN5221.1  	-FGTDNSKGLSEKEMRSILSKELRGNGKSGVKFGSGVWSKAVQMLEGRVF--------
          	   ..       . : .:  : :  . * . .  :   . : .
```
