# Supplementary material for: Patterns of Intron Gain and Loss in Fungi
Source: PLoS Biol. 2004 Nov 30;2(12):e422. doi: 10.1371/journal.pbio.0020422 (PMC532390; doi:10.1371/journal.pbio.0020422)
Supplement: Table S1 — Also available at http://genes.mit.edu/NielsenEtAl/. (4.3 MB ZIP). [file pbio.0020422.st001.zip › NielsenEtAl/html/1142.html]

AN5747.1.NCU07367.1.MG00330.1.FG01198.1


```
 CLUSTAL W (1.82) Multiple Sequence Alignments - Introns Inserted


Sequence 1: NCU07367.1	256 aa
Sequence 2: FG01198.1	400 aa
Sequence 3: MG00330.1	282 aa
Sequence 4: AN5747.1	339 aa
Alignment Length: 402 aa
Number Identitical Residues: 213 aa
Alignment Score (without introns) 8380


MG00330.1 	M--STDQERQHALDSYKQKLIESREWESKLKNLRIEIKGLQKDFDQTEENIKALQSVGQI
NCU07367.1	---MADQERQHALASFKAKLIESREWEAKLKNLRLELKGLQKEYDQTEENIKALQSVGQI
FG01198.1 	M--SAEEERQAALNSYRAKLIESREWEAKLKNLRLEIKDMQKEFDKTEDNIKALQSVGQI
AN5747.1  	MNGAVDPEREQALEEYKRSLLDLREWEAKLKALRMGIKDLQREFDISEENIKALQSVGQI
          	 .. .: **: ** .:: .*:: ****:*** **: :*.:*:::* :*:***********

MG00330.1 	IGEVLKQLDDERF1IVKASSGPRYVVGCRSKVDKDKLKQGTRVALDMTTLTIMRMLPREV
NCU07367.1	IGEVLKQLDDERF1IVKASSGPRYVVGCRSKVDKAKLKQGTRVALDMTTLTIMRMLPREV
FG01198.1 	IGEVLKQLDDERF1IVKASSGPRYVVGCRSKVDKVKLKQGTRVALDMTTLTIMRMLPREV
AN5747.1  	IGEVLKQLDEERF1IVKASSGPRYVVGCRSKVDRSKLKQGTRVALDMTTLTIMRMLPREV
          	*********:*** *******************: *************************

MG00330.1 	DPLVYNMSLEDPGQVSFAGIGGLNDQIRELREVIELPLKNPELFLRVGIKPPKGVLLYGP
NCU07367.1	DPLVYNMSLEDPGQVSFGGIGGLNDQIRELREVIELPLKNPELFLRVGIKPPKGVLLYGP
FG01198.1 	DPLVYNMSLEDPGQVSFAGIGGLNDQIRELREVIELPLKNPELFLRVGIKPPKGVLLYGP
AN5747.1  	DPLVYNMSLEDPGQINFAGIGGLNDQIRELREVIELPLKNPELFQRVGIKPPKGVLLYGP
          	**************:.*.************************** ***************

MG00330.1 	PGTGKTLLARAVASSLETNFLKV1VSSAIVDKYIGESARLIREMFGYAKEHEPCIIFMDE
NCU07367.1	PGTGKTLLARAVASSLETNFLKV1VSSAIVDKYIGESARLIREMFGYAKEHEPCIIFMDE
FG01198.1 	PGTGKTLLARAVASSLETNFLKV1VSSAIVDKYIGESARLIREMFGYAKEHEPCIIFMDE
AN5747.1  	PGTGKTLLARAVASSMETNFLKV1VSSAIVDKYIGESARLIREMFGYAKEHEPCIIFMDE
          	***************:******* ************************************

MG00330.1 	IDAIGGRRFSEGTN1QD-----~-------------YYG--------NQPS-------R-
NCU07367.1	IDAIGGRRFSEGTS~ADREIQP1-------------------------------------
FG01198.1 	IDAIGGRRFSEGTS~ADREIQR~TLMELLNQLDGFDYLGKTKIIMATNRPDTLDPALLRA
AN5747.1  	IDAIGGRRFSEGTS~ADR----~-------------------------------------
          	*************.  *                                           

MG00330.1 	-----------------------HTG~S-------------------GSAPCR----PS~
NCU07367.1	--------------------------~--------------------------------~
FG01198.1 	GRLDRKIEIPLPNEVGRLEILKIHSQ~SVVIDGDLDFESVVKMSDGLNGADLRNVVTEA2
AN5747.1  	---------------------EIQRT2TVQMEGDIDFESVVKMSDGLNGADLRNVVTEA~
          	                     .     :   ... . .:  . :.. ..:   .  : : 

MG00330.1 	GP---QD-RDCVAQ-------------------------~--~--~-----
NCU07367.1	---------------------------------------~--~--~-----
FG01198.1 	GLFAIKDYRESINQDDFNKAVRKVAESKKLEGKLEYQKL2ID~ER1ATIGQ
AN5747.1  	YVMPLFLYLVIANVEKGSICYQGLSRCDQ-SGRLQQGRP~QD1PR~GNEPC
          	   .          .. . .    : ... ..  .      .    ..
```
