# Supplementary material for: Patterns of Intron Gain and Loss in Fungi
Source: PLoS Biol. 2004 Nov 30;2(12):e422. doi: 10.1371/journal.pbio.0020422 (PMC532390; doi:10.1371/journal.pbio.0020422)
Supplement: Table S1 — Also available at http://genes.mit.edu/NielsenEtAl/. (4.3 MB ZIP). [file pbio.0020422.st001.zip › NielsenEtAl/html/1143.html]

AN2901.1.NCU02333.1.MG10854.1.FG10967.1


```
 CLUSTAL W (1.82) Multiple Sequence Alignments - Introns Inserted


Sequence 1: AN2901.1	324 aa
Sequence 2: FG10967.1	325 aa
Sequence 3: NCU02333.1	358 aa
Sequence 4: MG10854.1	342 aa
Alignment Length: 362 aa
Number Identitical Residues: 174 aa
Alignment Score (without introns) 8497


MG10854.1 	-MLTTMVES---KFLSSPEDLGVVAVGFSGGQVRHSWP-----WLLPR-LIYRCAETDKS
NCU02333.1	-MSPSLVDNHAAAYIAAPSSAKAPMIQKPGNTFGMSSPIESKFLSQPRDLGIVAVGFSGG
FG10967.1 	-MNTGLVNS---RFLSKPDEVGVVAVGFSGG-----------------------------
AN2901.1  	MTSPSTIKQ---RFLSKPNQLGVVAVGFNGG-----------------------------
          	   .  :..    ::: *..  .  :   *.                             

MG10854.1 	Q~CKPGVDAAPSALIDSGLLTQLHSELGYRLHGHTQVHLYGDLEPANDPPYRNMKKPLAV
NCU02333.1	Q0CKPGVDAAPSALIESGLLTQLREELGYRLHGDDEVHLYTDLVPKEDPPHRNMKNPRAV
FG10967.1 	Q~PKAGVDIGPAALIQSGLLTEIRDELGYKLFGDETVQQFEDLIPESDPDFRGMKKPRHA
AN2901.1  	Q0CKLGVEAAPMALVEAGLLDQLRDDLDYEIHYDNTVHYYEKEIPAEDPDHRGMKKPRAV
          	*  * **: .* **:::*** :::.:*.*.:. .  *: : .  * .** .*.**:*  .

MG10854.1 	SAVTRRIAEQTYEQAKEGRMVLTLGGDHSIAIGTIGGVAKAIKERFQGRREVAVIWVDAH
NCU02333.1	SNVTKRIAEQVHSHAKEGRLVLTLGGDHSIAIGTIAGSAKAIKERLG--REIAVIWVDAH
FG10967.1 	SAVTRKIASHTYEHSREGRMTLTLGGDHSIAIGTIAGTAKATRERLN--REIAVIWVDAH
AN2901.1  	SAVTETLSSQVYEHSKEGKFTLTLGGDHSIAIGSISGIAKATRERLG--REIGVIWVDAH
          	* **. ::.:.:.:::**::.************:*.* *** :**:   **:.*******

MG10854.1 	ADINTPETSDSGNIHGMPVSFLTGLATEDKDEYFGWLKDDNRISVNKLVYIGLRDVDPGE
NCU02333.1	ADINTPETSGSGNIHGMPVSFLTGLASEDKEEFFGWLKPDHLLSVKKLVYIGLRDVDPGE
FG10967.1 	ADINTPESSDSGNIHGMPVAFLTGLAKEEKEECFGWLEDDMRLNVKKLVYIGLRSVDIGE
AN2901.1  	ADINIPEMSPSGNIHGMPMAFLTRLATEEKKDIFGWLQEEHKVNLRKLVYIGLRDVDRGE
          	**** ** * ********::*** **.*:*.: ****: :  :.:.********.** **

MG10854.1 	KKILRENKIKAFSMFDIDR~HGIGRVMEMALAHIGTDTPIHLSFDVDALDPMWAPSTGTP
NCU02333.1	KRILRENGIKAFSMHDIDK2HGIGRVMEMALGHIGNDTPIHLSFDVDALDPMWAPSTGTP
FG10967.1 	KKILREHGIKAFSMHDVDR2HGIGRVVEMALAHIGNDTPIHLSFDVDALDPMWAPSTGTP
AN2901.1  	KKLLREHGIKAFSMHDVDR2HGIGRVVEMALAHIGNDTPIHLSFDVDALDPQWAPSTGTP
          	*::***: ******.*:*: ******:****.***.*************** ********

MG10854.1 	VRGGLTLREGDYICECVHETGQLVAMDLVEVNPSLSPSDGDPGAHETVRAGCSL0-----
NCU02333.1	VRGGLTLREGDFICECVHETGSLVAVDLVEVNPTLAAPN-DVGAHETVRAGCSL~VRCAL
FG10967.1 	VRGGLTLREGDFICESVHETGNLVAIDLVEVNPHL--ADTKQAEQNTIHAGCSL~VRCAL
AN2901.1  	VRGGLTLREGDFICECVHETGNLISMDLVEVNPSL--EAVG--ASDTIRTGCSL~VRSAL
          	***********:***.*****.*:::******* *          :*:::****   .: 

MG10854.1 	-----
NCU02333.1	GESLL
FG10967.1 	GETLL
AN2901.1  	GDTLL
          	..:
```
