# Supplementary material for: Patterns of Intron Gain and Loss in Fungi
Source: PLoS Biol. 2004 Nov 30;2(12):e422. doi: 10.1371/journal.pbio.0020422 (PMC532390; doi:10.1371/journal.pbio.0020422)
Supplement: Table S1 — Also available at http://genes.mit.edu/NielsenEtAl/. (4.3 MB ZIP). [file pbio.0020422.st001.zip › NielsenEtAl/html/1144.html]

AN3728.1.NCU06870.1.MG00864.1.FG07945.1


```
 CLUSTAL W (1.82) Multiple Sequence Alignments - Introns Inserted


Sequence 1: MG00864.1	519 aa
Sequence 2: FG07945.1	516 aa
Sequence 3: NCU06870.1	521 aa
Sequence 4: AN3728.1	504 aa
Alignment Length: 521 aa
Number Identitical Residues: 255 aa
Alignment Score (without introns) 13300


MG00864.1 	MDLHELQSKLTLWLDEASHAFQRVPGSAVLIRYVKSSYQNDPVRSAIELVLVVFFIRYLM
NCU06870.1	MELQELQTHLTEWLNEAITAFQKVPGSAVLIRYVRSSYQNDPVRSAIELVLVIFFIRYLM
FG07945.1 	MELDTLQAKLGDIIEQASVYFHKVPGSAVFLRYIKSSYQNDPVRSAIEAVLLLFFVRYLL
AN3728.1  	MDVQETQRLLSEYLHELANLFHRVPGSAIFLRYVKSSYQNDPIRSAVELFLFLFAVRYLL
          	*::.  *  *   :.:    *::*****:::**::*******:***:* .*.:* :***:

MG00864.1 	APSYSTHKQNFIKLRED0EIDELVEDWTPEPLVPARTAQEEAEAEKLPVIVG~PTGPKTK
NCU06870.1	APAYSTSKQNFIKLTDD0EIDELVDEWTPEPLVPNMTVLEEMESEKLPIIVG2ATGPKSK
FG07945.1 	SPSYSTHKQNFVKLRED0EIEELIDDWQPEPLVEEQTAFEASETERLPILVG2PTGPKSK
AN3728.1  	APKYST-KPGVVQLSED~EIDDLVDEWTPEPLVGKPTALEEMEIDKRPVIAG2PVGPKVR
          	:* *** * ..::* :* **::*:::* *****   *. *  * :: *::.* ..*** :

MG00864.1 	LASGRTVTNLSTYNYYNFNANEQIKEKAIQTLRTYGVGPCGPPQFYGTQDVHMKTEADIA
NCU06870.1	LANGRTVTNLASYNFYNLNANEQIKEKAIQTLRTYGVGPCGPPQFYGTQDVHMRAEADIA
FG07945.1 	LANGRTVTNLASYNFYNFNGNDQIKEKAIQVLRTYGVGPCGPPQFYGTQDVHMKTESDIA
AN3728.1  	LSNGRTVMNLGSYNFYNFNTNESIKEKAIQTLRNYGVGPCGPRGFYGTQDVHMKTEADVA
          	*:.**** **.:**:**:* *:.*******.**.********  *********::*:*:*

MG00864.1 	AYIGTEGCIIYAHAFSAVTSVIPSFCKRGDVIIADRMANYSIRKGLELSRSSIRWHGHGD
NCU06870.1	NYIGTEGCIVYAQAFSTISSVIPAFCKRGDIIVADRAVNYSIRRGLEISRSNIRWYAHND
FG07945.1 	AYLGTEGCIVYAQAFSTISSVIPSFCKRGDVIIADRNVNFSIRKGLEQSRSTIRWFEHND
AN3728.1  	SYLGTASCIIYSQAFSTISSVIPAFSKRGDIIVADKGVNFAIRKGIQISRSIVRWYEHND
          	 *:** .**:*::***:::****:*.****:*:**: .*::**:*:: *** :**. *.*

MG00864.1 	MEELEAAMAKVAKEQAKNKKLTRRFVVIEALSELLGDIADLPKL0IELKEKYKFRLILDE
NCU06870.1	LDDLERVMAKVVAEQARTKKLTRRFLVTEALFETTGEMNDLPHL~IELKEKYKFRIMLDE
FG07945.1 	MDDLQDVMKAVAKEQANAKKLTRRFVVTEGLFELSGDSIDLPRL~VELKEKYKFRVILDE
AN3728.1  	MEDLERVLAKITKEQAR-KPLTRRFIITEGLFESYGDMSDLPKI0IELKLKYKFRLILDE
          	:::*: .:  :. ***. * *****:: *.* *  *:  ***:: :*** *****::***

MG00864.1 	TWSFGVLGRTGRGLTEAQNVDPTQVDMIVGSMAGPLCAGGGFCAGSRDVVEHQRIMSTAY
NCU06870.1	TWSFGVLGRTGRGLTEAQNVDPTQVDMIVGSLAGPLCAGGGFCAGPKDVVEHQRLTAASY
FG07945.1 	TWSFGVLGRTGRGITEAQNVDPQQVDMIIGSLAGPLCAGGGFCAGPKDVVEHQRITSSAY
AN3728.1  	SWSFGVLGRTGRGITEHQNVDAAEVDMIVGSLAGPLVAGGGFCAGSEEIVHHQRISAAAY
          	:************:** ****. :****:**:**** ********..::*.***: :::*

MG00864.1 	TFSAALPAMTAVTASETLNLLQSNPE-ILSQCRENIKALRAQLDPRSDWVFCTSSIDNPI
NCU06870.1	TFSAALPAMLAIIASESLHVIQENPEATLGVCRENIRLMRAQLDPKSDWVICTSAPENPI
FG07945.1 	TFSAALPAMLAVTASETLNLLQSNPD-ILSQSRENIKAMKAQLDPRSDWVYCPSDLENPI
AN3728.1  	TFSAALPALLSTTASATINILQNSPE-TISHLRDLTKAMWAQLDPRSDWVRCTSAPENPI
          	********: :  ** :::::*..*:  :.  *:  : : *****:**** *.*  :***

MG00864.1 	LLLQLKPEVIASRRLTAEDQNRLLQECVDE0SLANGVWITRLKIQPVLNTIGPKE-NITI
NCU06870.1	LLLVLKPQVVEARRLTAEDQERLLQEVADE0CLANNILVTRLKGGPITTHMGLKDNVYTA
FG07945.1 	MLLVLKPEVVAARKLGLEDQERILCDCVEE0TLANGVLITRTKTRPYSHAVKPKDGAWFA
AN3728.1  	LVLVLKPEVVAAKRLSHEDQQYVLQDVVDE0CIANGVLITRLKC--LDDNFEPKQ---NV
          	::* ***:*: :::*  ***: :* : .:*  :**.: :** *       .  *:     

MG00864.1 	QPALKVCVTSGLSRKDIERAGTTIRHAITKVMKSNAKLSPAVAPA
NCU06870.1	TPALKVCVTSGLSKKETEKAGIAIRHAITKVMTKKGNNKLGVPTA
FG07945.1 	QPALRICVTSALSKKDIEKAGVTIRHAITKVMTR--KTSNKTV--
AN3728.1  	PAALKVCVTTGLTKKEIEKSGTIIRHAITKVLSKRK---------
          	 .**::***:.*::*: *::*  ********:.
```
