# Supplementary material for: Patterns of Intron Gain and Loss in Fungi
Source: PLoS Biol. 2004 Nov 30;2(12):e422. doi: 10.1371/journal.pbio.0020422 (PMC532390; doi:10.1371/journal.pbio.0020422)
Supplement: Table S1 — Also available at http://genes.mit.edu/NielsenEtAl/. (4.3 MB ZIP). [file pbio.0020422.st001.zip › NielsenEtAl/html/1145.html]

AN8877.1.NCU03264.1.MG09944.1.FG06859.1


```
 CLUSTAL W (1.82) Multiple Sequence Alignments - Introns Inserted


Sequence 1: MG09944.1	744 aa
Sequence 2: FG06859.1	716 aa
Sequence 3: NCU03264.1	771 aa
Sequence 4: AN8877.1	554 aa
Alignment Length: 774 aa
Number Identitical Residues: 294 aa
Alignment Score (without introns) 16167


MG09944.1 	MDQADIPALLSRLASDEDAARKMAVFKLQSSINDPAFADVFISSGGLVILRRLIMTTGGN
NCU03264.1	MDQADIPALLSRLASDEDAARKMAVFKLQSSINDPAFADVFIASGGLVVLRRLIMTTGGN
FG06859.1 	MDQADIPALLARMASDEDASRKMAVFKLQNSINDPAFADVFISSGGLVVLRRLVMTSAGN
AN8877.1  	MD-SNVSELVERLGSEEDAVRKMAVFKLQSSIGDPSFADVFIAEDGLTRLRYLTLHATGN
          	** :::. *: *:.*:*** *********.**.**:******:..**. ** * : : **

MG09944.1 	TLAYSLQSLTRLLEVDMGWDIFEGPSAGDLVER~VVELIVTNPLVNILRGAMSILVALVG
NCU03264.1	TLAYSLQSLSRLLEVDMGWDIFEGPNAGELVER~VVELIVTNPLVNILRGAMSILVGLVS
FG06859.1 	TLAYSLQSLTRLLEVDMGWDIFEGPTSSDLVER~VVELIVTNPLVNILRGAMSILVALVG
AN8877.1  	TLAYSLTSFARLLEVDKGWECVD----HELVER0VVELIVTHPLVNILRGAMSILVSIVS
          	****** *::****** **: .:     :**** *******:**************.:*.

MG09944.1 	HSQSTAR-GAAARSPGTFGFRALKPAVAVYPQFFELVIQQLQSADHALCANALALINALI
NCU03264.1	HSQSTAR-GGTPRTPGTFGFRALKPAVAVYPQFFELVIMQLQSADHALCANALMLINALI
FG06859.1 	HSQSTHQSGTTNRAPVTFGFRALKPAVAVYPQFFELVIQQLQSADHALCANALMLINALI
AN8877.1  	HPSSVGR----LSQNAVWGFRALKPAIAIYPQFLEMLVNRLSSADHALCANALQLINSLM
          	*..*. :         .:********:*:****:*::: :*.*********** ***:*:

MG09944.1 	RDAVSNDSG---STKSPSSTTTGGEEWSKLIKRLQDLGLIKAVFNLMQSSALQDLAHPLL
NCU03264.1	RDAVSNDSGPTLLVSSAKGTVPPGEEWSKFIKKLQDLGLIKAVYNLMQSSSLQDLAHSLL
FG06859.1 	RDAVSDDS----TMNGGKASAGSGEDWAKFIKRLHDLGLIKAVHRLMQSSALQDLAHPML
AN8877.1  	RDSITNDSD---------------HEWPKFIQKLQDLGVIKAVYSLMQGTALQDHAHPLI
          	**::::**.               .:*.*:*::*:***:****. ***.::*** **.::

MG09944.1 	EFQSLTKALLHRWKEVRVDLERPEHRRALKGLHLASAPDRQHATA---------------
NCU03264.1	EFQTLTKILLQKWRAVRVDLERPEHRRALKGLHLASAPDRRQTNGGLTTLSPTGRPSTAA
FG06859.1 	EFQTLTKILLRKWREVDVDLERPEHRRALKGLHLASAPERVPANG---------------
AN8877.1  	EFQSLTKILLRKWRDTALDLENPEHRRALKGIHLASSQEKGNETG---------------
          	***:*** **::*: . :***.*********:****: ::   ..               

MG09944.1 	--TGSGPDTSGSDAPPAVPEKTSRRHNPEKWRRLGFETESPAIEFESTGFLGMMDLTDYV
NCU03264.1	TVVASEEGGKETGGEPAAGRKSSRRHNPEKWRRLGFETESPASEFEVAGFLGMMDLTDYV
FG06859.1 	--HSLGQDS--HEGP---TKKGSRRHNPEKWRRLGFETESPAQEFDMTGFLGMMDLTDYV
AN8877.1  	-----------------ADMRRSKKHSPEKWRRLGFESESPVAQFEDMGFLGMMDLADYV
          	                    : *::*.**********:***. :*:  ********:***

MG09944.1 	RKNEEGFQKLLLEQATRPLEERCPIARASFAVSMVLYEHFEVDKADTEDLRA---YQTLE
NCU03264.1	RKNEDGFQKLLLEQSSRPLNERCPVARASLAVTMILYEHFEIEKCDLDDIRNGGYYQLID
FG06859.1 	RKNEDGFQKMLLEQAGKPACGRCPVARASFAATMILYDHFDVDKTDLDDVRS---YQLLE
AN8877.1  	RNHQDEFQKMLLEQSTKPARQRCPIARASLSVTSILYDHFEVDKCETEDSKT---YLILE
          	*:::: ***:****: :*   ***:****::.: :**:**:::* : :* :    *  ::

MG09944.1 	PGSKSHDRLFRPLLLQWSRLHTAGLHAFFRLWKATGAKRDDFDKVAELVRILIEQVVGQA
NCU03264.1	GGGKAHDKLFRPLLLQWSRLHTAGLHAFFRMWKATGATRYDFDKVAELVRILIDQVVGQA
FG06859.1 	--SKDHDRLFKPLLLQWSRLHTAGLHAFFRVWKLTGAEQGDFEKVAELVRILVDSVVGAS
AN8877.1  	S-RSNLDKLFKPLLLHWTRLHVAGLHSFFRLWKSTSAELEDYDKIVELVRILIESVVGGA
          	   .  *:**:****:*:***.****:***:** *.*   *::*:.******::.*** :

MG09944.1 	PRTKDVLEVEDELQEFSCARLRELQMNLLELSFEDQWGQHMYQVREDLKHEALQFVKEQR
NCU03264.1	SRNKDVLEVEDELHEYDSGRLRELQMDLLELSFEDQWGSHLFQVREELKHEALQFVKEQR
FG06859.1 	RRTKDVAEVEEEMQEYDVPRLREQQMGLLEMSFENTWGQHLQQVREELKQEALQFVKEQR
AN8877.1  	ARTKDVQDVEEDLMEFEYSRLRDLQMELLELTYEDAWGQHLRQVREELQHEAAQFVKEQR
          	 *.*** :**::: *:.  ***: ** ***:::*: **.*: ****:*::** *******

MG09944.1 	IRCLLHGSWFTRPIPKRDQPQPQHTRQDSNMTKRRLYEAKAWRFAKLSHNRRFLHYADFA
NCU03264.1	IRCLLQGSWFSKPQPHRSDHSRSESLKPGQEKGSRLYQP--WRFAKLSHNRRYLHYADFA
FG06859.1 	IRCLLQGSWFSKPTPKRD---------NNSQVKHRLFTPTPWRYAKLSHNRRYLHYADFE
AN8877.1  	IRCLLQGAWFPNE--------------NISELEGESGSPK-WRYVQLSHNRRILHFGDFE
          	*****:*:**..                 .    .   .. **:.:****** **:.** 

MG09944.1 	MQTPQDPGLEDLTEKVDLGTISSVVSNVS-APSEDARSVSSSSTLRHGPNAPNTTNSNIT
NCU03264.1	EQTAHDPGLEALTEKIDLLSTSSVVSNVSNADDAAAAGENGNQASSSLTSLTKDANSASA
FG06859.1 	ERTVSGPGLDALAEKVDLSTISSVVSNVS-APTEDMRSTFSDMMGKD------------T
AN8877.1  	SMEMNRLDLDVLPGKSEVPLQPAIFELLAN------------------------------
          	       .*: *. * ::   .::.. ::.                              

MG09944.1 	PPKATTKITIYSYVN-----AEEAARGGDAKEQAIVTLYPLNHSLASEWLDGLLMLLNQQ
NCU03264.1	TAKSTTKITIYSLSENVSPELASLATAGKTKEQPILTLHPLNHSLASEWLDGLLMLLNQA
FG06859.1 	APKTTTKITIYSFVN-----PAEASRGAEAKEQPILTLWPLSHSLASEWLDGLLMLLNQT
AN8877.1  	------------------------------------------------------------
          	                                                            

MG09944.1 	PITSETNKLVTLVSDYGLKIRLLNVRVEAVYQGPAPGAGEVPSREGLDENYYYEV
NCU03264.1	PITAETNKLVTLVSEYGLKIRLLNVRAEAVDNGPVPGAGQVPSREGLDEDYYYEI
FG06859.1 	PITAETNKLVSLVSEYGLKIRLLNVRMDSAFEGPEPGAGVIPSREGLDDDYFFEV
AN8877.1  	-------------------------------------------------------
          	
```
