# Supplementary material for: Patterns of Intron Gain and Loss in Fungi
Source: PLoS Biol. 2004 Nov 30;2(12):e422. doi: 10.1371/journal.pbio.0020422 (PMC532390; doi:10.1371/journal.pbio.0020422)
Supplement: Table S1 — Also available at http://genes.mit.edu/NielsenEtAl/. (4.3 MB ZIP). [file pbio.0020422.st001.zip › NielsenEtAl/html/1146.html]

AN4313.1.NCU06847.1.MG03205.1.FG04370.1


```
 CLUSTAL W (1.82) Multiple Sequence Alignments - Introns Inserted


Sequence 1: MG03205.1	652 aa
Sequence 2: FG04370.1	633 aa
Sequence 3: NCU06847.1	633 aa
Sequence 4: AN4313.1	578 aa
Alignment Length: 673 aa
Number Identitical Residues: 272 aa
Alignment Score (without introns) 14585


MG03205.1 	--MNEVDRELNRAEVDAMASPGSHNPQSPLNRRDSNEIERVISASSVSTASSISSVRRAT
NCU06847.1	MPLTEADREIAAAEIDASPMEFAGQES---LARRSDEVERVMSTSSVSTSSSEERQRRAR
FG04370.1 	MSGNIVDRDL--LEAERAASPQRYQ------RRDSAEIERVISASTVSSSSSENSARRRS
AN4313.1  	-----MHTTAMGENDDSFPNDQISD--------SDSQIDRTDSYSSSDTSSSDDDVECMS
          	      .      : :  .     :         . :::*. * *: .::** .  .   

MG03205.1 	GAAAAASRSRDATSLRSVATQQ--DLERHPTVLSRIHTARSQHTATVGRTRSDAGSTSRR
NCU06847.1	R-------------MSGVSTQH--DLERHPTELSRIQTQRSQHSGTVGRS----GTRNTR
FG04370.1 	RSIGQHN------SISRISTQN--DLEHHPTELSRIQTARSQHNATVG------GSLRSR
AN4313.1  	R-------------MQTAQTQPGYGLERHPTALSRIATQRSQHSATVG-------SLRPR
          	              :    ** . .**:*** **** * ****..***       :   *

MG03205.1 	SWFRRTTSKALPPFGAGKEYPPPLPDREEYVVEFDGPDDPMHAQNWPLRKK~LVTAIILG
NCU06847.1	TSRR--SEKPLPPFGAGKPYPPQLPDPDEYVVEFDGPDDPLHAMNWPFKKK2FWSAAMLG
FG04370.1 	TASR-ASRKPLPNFGAGKPYPPPLPEQEQYVVEFDGPDDPLHSQNWPLKKK~LITAAVLG
AN4313.1  	-----QSRKPLPEFGAGKPYPPPLPDKEEYVVEFVGPDDPLHPQNWPTKKN2--------
          	      : *.** ***** *** **: ::***** *****:*. *** :*:         

MG03205.1 	YTTMTSSFASSIFSAATRALAAEFDVSTEVGILGVSFFVLGFAFGPTLWAPLSELKGRRL
NCU06847.1	YTSLVAAFGSSIFSSATSSIARIYHVNQTVGILGVSFYVLGFAFGPTLWAPLSELKGRKL
FG04370.1 	FTTMTAAFTSSIFSAATMIVAADYGVDNEVGLLGTTFYVLGFAFGPSLWAPLSELKGRRL
AN4313.1  	-----------IFSSANSVVSAKFNVSTEVGTLGMSLYVLGFAFGPTLFSPLSELFGRRL
          	           ***:*.  ::  : *.  ** ** :::********:*::***** **:*

MG03205.1 	PLLISMFGFTIFSIATASGKDIQTILISRFFSGFFGACPLAVFAAVFSDMFGNRTRGIAI
NCU06847.1	PIVIGMFGMAIFSIATATAKDLQTILITRFFGGFFGACPLAVVAAVFSDMFDNRTRGIAI
FG04370.1 	PILISIFGFSLFSIACATGKDIQTILLCRFFSGFFGACPLAVVAAVFSDMFDNRTRGTAI
AN4313.1  	PILIGIFGFTVFQFGVATAENLQTVIICRFFGGFFGACPIAVVAAVFSDIYDNRHRGLAI
          	*::*.:**:::*.:. *:.:::**::: ***.*******:**.******::.** ** **

MG03205.1 	TVFSMAVFTGPFLAPFIGGFIVESYLGWRWTMWLTSIMGAVALIFNVLFLEETYPPSILV
NCU06847.1	TLFSMTVFTGPMMAPFIGGFISTSYLGWRWTEYIVSFMAFLAFGLDLLFMHETYPPQILV
FG04370.1 	TVFSMSVFTGPLLAPFIGGFIVESHLGWRWTEYLASIMGFTALILDLIFLEETYPPVILI
AN4313.1  	TIFTMMVFTGPLFAPFIGGFIVDSYLGWRWTEYLAGILGATAFVLDLFFVHETYPPIVLI
          	*:*:* *****::********  *:****** ::..::.  *: ::::*:.***** :*:

MG03205.1 	AKASELRRLTLNWGIHAKQEEIEVDFVELVNKNFSRPMRLLFTEPIVLSLSIYMAFIY--
NCU06847.1	KKASELRRRTLNWGIHAKQEEIEIDFKELVSKNFSRPLRLLFGEPIVALLSIYMAFIYGI
FG04370.1 	AKAADLRRRTKNWGIHAKQEEIEVDFKELVKKNFSRPLRLLFTEPIILLLSIYMSFIYGI
AN4313.1  	RKAEELRRRTKNWGIHAKQEEVEIDLGELIAKNFSRPVRILFSEPVLLLLSIYMAFLYGL
          	 ** :*** * **********:*:*: **: ******:*:** **::  *****:*:*. 

MG03205.1 	------A1YPLVFQGVYGFSGGIAGLAFFGMIIGQFLAGVVVLLQQPWFNRKLSANNGVA
NCU06847.1	LYLFLTA~YPIVFQGVYHMTPGVSGLTFFGMITGQILAGVTILLQQPWYMRKLNANNGVP
FG04370.1 	LYLFLTA~YPLVFVGVHGFSSGQSGLCFFGMIIGQLIAGATVIAQQPWYIRKLAANNGIP
AN4313.1  	LYLFMTA~YPIVFQRIHGFNKGVGGLPYFGLILGEFLGGFFIIAMQPWYNRKLSANGDIP
          	     :* **:**  :: :. * .** :**:* *:::.*  ::  ***: *** **..:.

MG03205.1 	IPEWRLPSVIGGAFAFTAGIFWFGW~TGYNKDIHWIVPTASGILTGFGLMSIFLQALNYL
NCU06847.1	IPEWRLPSVIAGGVAFSAGLFWFGW~SGYSGKVHWIVPTLSGILSGFGLASIFLQALNYL
FG04370.1 	VPEWRLPNIMAGGVSFAIGIFWFGW2TGYTNKIHWIVPALSGLFTGFGLMSIFLQALNYL
AN4313.1  	IPEWRLPPAIIGSMAFAAGLFWFGW~TGYTGNTHWIVPTVSGILTGFGLLCIFLQCLNYI
          	:******  : *..:*: *:***** :**. . *****: **:::**** .****.***:

MG03205.1 	VDAYLNF~AASAIAGNTFLRSLFGAAFPLFAGQM--------~FNGMGINWASTLLGCIA
NCU06847.1	VDSYLMF~AASAIAGNTFLRSLAGAGFPLFSTYM--------~FKGMGIQWASTLLGCVA
FG04370.1 	VDAYLMF~AASAIAGNTFLRSLCGAGFPLFARQM--------~FDGLGIQYAATLLGCIA
AN4313.1  	IDTYLVF2AASALAANSILRSFAGAGFPLFAPYMVRQTLSGK0FNAIGVNWTGTLLGCVA
          	:*:** * ****:*.*::***: **.****:  *  .: :.. *..:*::::.*****:*

MG03205.1 	AVMIPIPIIFYLYGHKLRARSKFAPTGSPIELQQHEQEEAARQSRVSLPTDAESKDPAPS
NCU06847.1	AALVPIPIIFLYYGHRIRAKSAYAPTFAPRAPAAEDESGDETNSEHAVG----EKDHQPS
FG04370.1 	VVLAPIPFIFYKYGAKIRQKSKYAPTAPPAPASGSSTEEEEKET---------ANDTALA
AN4313.1  	AVLMPIPLLFYLYGHRIRSKSRFAAEYTTVAKANTTATEN--------------------
          	..: ***::*  ** ::* :* :*.  ..                               

MG03205.1 	NGLSTQPHVQGKNQSPV-
NCU06847.1	S-VPRKDGDAATNSV---
FG04370.1 	AVRSRRDSITSANRKETA
AN4313.1  	------------------
          	
```
