# Supplementary material for: Patterns of Intron Gain and Loss in Fungi
Source: PLoS Biol. 2004 Nov 30;2(12):e422. doi: 10.1371/journal.pbio.0020422 (PMC532390; doi:10.1371/journal.pbio.0020422)
Supplement: Table S1 — Also available at http://genes.mit.edu/NielsenEtAl/. (4.3 MB ZIP). [file pbio.0020422.st001.zip › NielsenEtAl/html/1147.html]

AN0436.1.NCU00898.1.MG04518.1.FG00715.1


```
 CLUSTAL W (1.82) Multiple Sequence Alignments - Introns Inserted


Sequence 1: NCU00898.1	435 aa
Sequence 2: FG00715.1	431 aa
Sequence 3: AN0436.1	413 aa
Sequence 4: MG04518.1	451 aa
Alignment Length: 489 aa
Number Identitical Residues: 128 aa
Alignment Score (without introns) 7017


MG04518.1 	MSKTG---VAIPQLQKDLEGMEDKPRLSLAVDNIDRLIAKLTEAREAVATA1SDPHTASL
NCU00898.1	MTAQT---TAMEALQKELDRLNRAPGLSATEDRVDNIIEMLTSVKDQIVQS~--------
FG00715.1 	MATDNDRGSPFAQLMVELARMRNKTLKTAIFD-VDNVIDLLTKARDQIAQD1PD--RATM
AN0436.1  	----------MDLVQKEHERLSKRLKSSESIQSVQDAIDLLQSTRDKIASD1PN--QASV
          	          :  :  :   :      :   : ::  *  * ..:: :.   ..   :: 

MG04518.1 	TLTRLQNPVKEVIDRANDDLKK~VYQVQKDLGKSLDK0SFPVVKIA-TDHDALDGKSDLM
NCU00898.1	---------------------G1VTATQRKLGKTLDK~HFPLKDLP-STHDAMADQESLI
FG00715.1 	VMTTLQNPVKASFERITTDLKD~VTKAQKGFGKALDK0ALPHRELP-METDAMADHPGLI
AN0436.1  	ALAKLQNPIKLSFDAINDNLKE~THSGLNKYSKALDK0LFKDRPLPSSEHDVLSSQEHLI
          	  :  ... .   .  . . .  .    .  .*:***  :    :.:   *.: .:  *:

MG04518.1 	NKAIAMHLMREGQFSVASTFIGESQRDLHVASSTPQAFHG--------------KTRDVQ
NCU00898.1	NRAISMHLLREGQFSVASTFIEETGD--AATLENANAVVEGQDQVSASDNYDDEPMDEDR
FG00715.1 	NRAIAMHLLREGQFSVASTFLKEATD--HPPHREMHSVPR---------------TDEDG
AN0436.1  	NRAIAMHLLREGQFSVASAFLSEVAA--KRAAEKQQSFDS--------------------
          	*:**:***:*********:*: *       .    ::.                      

MG04518.1 	DSNTMAVDSDGDTDQQSQS-----SSKVPSQPNASSEVIGSSRLQQQFEVMYRILQELK-
NCU00898.1	DDDDDDDDDEDDDDDEYMSPLEGAATGLGFRQLQNLSSLQSHELEAKFSQMYTILQDIK-
FG00715.1 	DDDMDDGNDEEDTDDELEG-------------------LHSEDLQRKFSEMYYILSAVK-
AN0436.1  	-DNMDEAAALLDIGG-----------------------VPSSKVRDEFHNMYRILHELKE
          	 .:        * .                        : *  :. :*  ** **  :*.

MG04518.1 	QHNVEPAIEWALE--NSSELEARGSNLEFELCRLQYVHLFETPG----------RGPPAA
NCU00898.1	SRNLLSAIEWARS--NSGELEARGSNLEFELSRLQYVWLFKGPRVNGLPDN-ELNGTAGA
FG00715.1 	THDLIPAINWANM--NSTQLEAKGSTLEFELIKLQYVWLFKGPSVNGLPDDPARNGLGGA
AN0436.1  	NNNLLPAIEWSRKEENKVALEARGSNLEFELCRLQFVWLFHGGQEQRGPTP---EGRQAA
          	 .:: .**:*:  ..*.  ***:**.***** :**:* **.    .  .     .*  .*

MG04518.1 	FEYARTQMWRFRERHLAEITRLAGALIYAPNLADSPYATLFDSPTAFLDAASSFTREFCS
NCU00898.1	LLYAQQNFWRFGNRYIGEIQQLANAQIYARNLSESPYRHTFSTETAFADVASSFTREFCS
FG00715.1 	LNYARQHFTRFQGRHLPEIQQLCCAMAYASNLAGSPYRHIFETDSAFEDVAMSFTREFCS
AN0436.1  	LEYARREFQAFFPRYMREIQQLMGAMAFSPNLPDSPYKNIFNNPSAWSDVSHSFTREFCA
          	: **: .:  *  *:: ** :*  *  :: **. ***   *.. :*: *.: *******:

MG04518.1 	LLGLSAESPLYVAATAGAIALPRLVKWQSIAQG--AEWTTTNELA~FETPLPRSFMYHSI
NCU00898.1	LLGLSAESPLYVAVTAGALALPLLMKYQQATRAKGTEWTTTNELA~FETPLPERMLYHSI
FG00715.1 	LLGLSAESPLYVAVTAGSIALPRLIKYTTYMKEKKTEWTTENELA~FETPLPESMIYHPI
AN0436.1  	LLGLSPDSPLYIAATAGAIALPTLLKLQTIMKAKRTEWTTEHELP0VEIPLPPSYLFHSI
          	*****.:****:*.***::*** *:*     : . :**** :**. .* ***   ::*.*

MG04518.1 	FVCPVSKEQTTAANPPVILPCGHVLARDSLTNIAKG1HGNNMFWIPSFHGADWAAVELNR
NCU00898.1	FVCPVSKEQTTEQNPPMMIPCGHVLAKETLQRLLKG~TRFKCPYCPAEG-----------
FG00715.1 	FVCPVSKEQTTQDNPPMMLGCGHVICRESLQNIIKA~ARYKCPYCPTEG-----------
AN0436.1  	FVCPVSKEQTTDANPPMMMPCGHVIAEESLKRLCKG~TRFKCPYCPNES-----------
          	***********  ***::: ****:..::* .: *.    :  : *              

MG04518.1 	ATKYLAPIFLTLEL
NCU00898.1	LEKDARRIMI----
FG00715.1 	HPKDATKIRL----
AN0436.1  	HPREAKKVFL----
          	  :    : :
```
