# Supplementary material for: Patterns of Intron Gain and Loss in Fungi
Source: PLoS Biol. 2004 Nov 30;2(12):e422. doi: 10.1371/journal.pbio.0020422 (PMC532390; doi:10.1371/journal.pbio.0020422)
Supplement: Table S1 — Also available at http://genes.mit.edu/NielsenEtAl/. (4.3 MB ZIP). [file pbio.0020422.st001.zip › NielsenEtAl/html/1148.html]

AN4109.1.NCU07820.1.MG02319.1.FG11475.1


```
 CLUSTAL W (1.82) Multiple Sequence Alignments - Introns Inserted


Sequence 1: NCU07820.1	500 aa
Sequence 2: FG11475.1	492 aa
Sequence 3: MG02319.1	503 aa
Sequence 4: AN4109.1	456 aa
Alignment Length: 527 aa
Number Identitical Residues: 208 aa
Alignment Score (without introns) 11631


MG02319.1 	MVTTDPKHPVCLPIDTSDGAFDSKGTNSDYSSSSPLSVLADVNEQPKKSWRSTIWDTLDK
NCU07820.1	-MSKSTDSGVVTEGDLRG--PSSSLKSSRHEYTSDSQTTELVQEQPKRSWRSYVWDTLDK
FG11475.1 	-MAENPKQ--ISTERVRD--ANDQSVSS----DSETSALAVLQETPKRSWKSYLWDTFDK
AN4109.1  	----------MTSSKVAD------------ERATIDASETIIQETPKRRWVSYIWDTFDK
          	                 .            .  :       ::* **: * * :***:**

MG02319.1 	PKEERRFLFKLDAVILTMASLG~YFIKNLDQ~INITSAFVSGM~KEDLGLHGNELNYMIT
NCU07820.1	SPQERRFLFKLDAVILTLASLG~YFIKYLDQ~VNITNAFVSGM~KEDLKLFGNELNYMQT
FG11475.1 	SPEERRFLFKLDFALMTLASLG~YFIKYLDQ0VNINNAFVSGM~KEDMNLFGNELNYMQT
AN4109.1  	SPEERRLLTKLDAAILSFASLG1YFIKYLDQ~ININNAFVSGM2KEDLGMYQNQLNYMQA
          	. :***:* *** .::::**** **** *** :**..****** ***: :. *:**** :

MG02319.1 	CWTVGYVIGEIPS2NMLLTRIRPSIWIPTCE~VIWSVLTILLAKCTSATQIYVLRFFIG~
NCU07820.1	CWTVGYVIGEIPS2NILLTRIRPSIWIPACE~VVWAILTILLAKCKTTTQVYVLRFFIG~
FG11475.1 	CWTIGYVIGEIPS2NMLLTRIRPSIWIPACE0VTWSVLTILLIKCTNPTQLYVLRFFIG~
AN4109.1  	AWTVGYVIGEIPS~NIMLTKVRPRYWLPAME0LLWTVLTMCLSRCNKASQFYVLRFFIG1
          	.**:********* *::**::**  *:*: * : *::**: * :*...:*.******** 

MG02319.1 	LAESAFYPGMQYVIGSWYRKDELAKRSCIFHASGNIGG~MFSGYLMAAAYT-LNDTNGFR
NCU07820.1	LAESVFYPGMQYIIGSWYRKDELAKRSCVFHASGAIGT~MFSGYLMAAVHKKLAGVGGFK
FG11475.1 	LAESTFYPGMQYIVGSWYRKDELAKRSCLFHAMGNVGS~MVSGYLMAGSHN-LDGVHGYH
AN4109.1  	LAESTFYPGMQYIIGSWYRKDELAKRSCIFHTSSGIAT1CTD--------------EGYR
          	****.*******::**************:**: . :.    .               *::

MG02319.1 	GWQWLFIINTVVSLPIAIAGYFFFPDVPEITKAWWLTEA~EIDIARKRMVLEGRANRAPY
NCU07820.1	GWQWLFIIDTVISLPIAISGFFLMPDLPEITKAWYFSAD~EITLAKERMRLEGRAPRAPY
FG11475.1 	GWQWLFITNTVVSLPIAISGFFFLPDVPEITRAWYFTPE~EIAIAKRRMELEGRAKRAPY
AN4109.1  	----LFIIDGVISLPVALLGFVILPDVPEISNPWYLTKQ0EVQLCQKRMELEGRKNRGPY
          	    *** : *:***:*: *:.::**:***:..*:::   *: :.:.** ****  *.**

MG02319.1 	TKQKFKKIFSSWHIYLLPLLYI~LFNNGGGYGGQPVFALWLKSEG---YGIVAINSYPTI
NCU07820.1	TKAKFKKIFSSWHIYLLTALYI~FFNNGNGASGQPAFQLWLKSKG---HPITEVNTNPTI
FG11475.1 	TKAKFKKIFSSWHIYALVLLYI~LFNNGNGGSSQPAFPLWLKSQG---YTIRDVNLYPTI
AN4109.1  	TKAKLKKILTSWHIYFLTGLYI2TFNNANG--GQPVFQQYLKASTNPVYSVGQINSYPTT
          	** *:***::***** *  ***  ***..*  .**.*  :**:. .. : :  :*  ** 

MG02319.1 	AAAIAVVMTLAYAW~TSDTVFRGARWPPIVFAGCFKIVTETSLAVWSIPVGWKWFCFIFG
NCU07820.1	TAAITVITTLIYAW~TSDTVFRGARWPPIVFSGLVNIVIYSSLAAWNIPDGWKWACFFLA
FG11475.1 	VDVISVVTTLIYAW~TSDSLFRGARWPAIVFSGLVKIIAYVGLTVWKVPTAFTWVCFMLC
AN4109.1  	TYAVQVFTTLVYAS1-----------------------------VWDIPDGWKWTCYIMS
          	. .: *. ** **                               .*.:* .:.* *::: 

MG02319.1 	SVSSGISGLTFAWAHEICSDDNEERALVVATMNQMAYVVQAWLPLLIWQQVDQPRYQKGF
NCU07820.1	GFGGGISGLTFAWAHEICGDDNEERALVTGTMNEMAYVIQAWLPLLIWQQVEAPVYHKGY
FG11475.1 	GFGGGISGLTFAWAHEICSDDNEERALVTGAMNQMAYVFQAWLPLVIWQQVEAPSYPKGY
AN4109.1  	GAGYGLSGLCMAWAHEICSGDNEERALVVGSMNEMAYVFQAWLPQVVWQQIDAPQYRKGF
          	. . *:*** :*******..********..:**:****.***** ::***:: * * **:

MG02319.1 	ITMVFIAAGMIATAFAIRLLHHRER--ARK~AKEAEQGSS------------------
NCU07820.1	MTSIFIAVAMILTAVLVRFLHHKER--ARK~LLAEEPNA-------------------
FG11475.1 	PTMVAMAVALIGTAFAIRILHKQQIGGRRH~ALAEA----------------------
AN4109.1  	ITGTVMSVLLIIFTLSIRSLQTRENRKTRR2GGAVESEGSVGSQTEESVNVVVDTQGK
          	 *   ::. :*  :. :* *: ::    *:        .: .:.:..: .   .:...
```
