# Supplementary material for: Patterns of Intron Gain and Loss in Fungi
Source: PLoS Biol. 2004 Nov 30;2(12):e422. doi: 10.1371/journal.pbio.0020422 (PMC532390; doi:10.1371/journal.pbio.0020422)
Supplement: Table S1 — Also available at http://genes.mit.edu/NielsenEtAl/. (4.3 MB ZIP). [file pbio.0020422.st001.zip › NielsenEtAl/html/1149.html]

AN3508.1.NCU05649.1.MG02290.1.FG02084.1


```
 CLUSTAL W (1.82) Multiple Sequence Alignments - Introns Inserted


Sequence 1: MG02290.1	285 aa
Sequence 2: FG02084.1	304 aa
Sequence 3: NCU05649.1	348 aa
Sequence 4: AN3508.1	280 aa
Alignment Length: 348 aa
Number Identitical Residues: 92 aa
Alignment Score (without introns) 5167


MG02290.1 	----MEAPPKFVFYAYAPSLPAAIIFVIAFGLSTIWHIKQLCQSRTWYFIPFIIGCLF1E
NCU05649.1	MSDNNSDLATFAYYKYDPSTAANAIFVALFSITAVGHAFLLARNRTWYFIPFLVGCLF1E
FG02084.1 	-MAGSDAPQAYVFYNYNPSMVAAVIFIIVFGLSSLLHTFQLVRARTWYFIPFLIGCLF1E
AN3508.1  	--------MGYQYYMYDPSKGAAIPFAALFGLTTVVHMWQTIQNRTWYMTPFIIGGIF1E
          	          : :* * **  *   *   *.:::: *     : ****: **::* :* *

MG02290.1 	TVGYVGRAISSTESPNFNKNPYIIQSVLLLLGPTLFAASIYMTLGRIIRLLDAGQYSLVP
NCU05649.1	AVGYIGRVISAGETPDWTLTPYLIQSLLILLGPSLYAASIYMILGRLTCMLEAEAYSVIR
FG02084.1 	CVGYIGRALSANEAPDFTKNPYIIQSILLLLGPALLAASIYMVLGRLIVLLDAGHLSVIR
AN3508.1  	AIGYLCRFISATQTPNWTMYPYIGQSLLILLGPALFAASVYMLLGRIIRTLNAGSLSPIR
          	 :**: * :*: ::*::.  **: **:*:****:* ***:** ***:   *:*   * : 

MG02290.1 	ARWITKLFVLGDVLSFFAQGAG1GGLLTTAKTENDVAKGQNIILGGLGIQILFFSFFVIA
NCU05649.1	VKWLTKIFVLGDVFSFLAQGAG1GGILAKATTPKDQDLGNNIILVGLGIQIAFFGLFIIT
FG02084.1 	PNWLTKVFVTGDVLSFLAQSAG1GGMLATAKDKDAVKRGENIIVGGLIIQILFFGFFMIV
AN3508.1  	PNWLTKIFVAGDVISFFMQSGG1GGMQASAKTQDRAEMGENMILGGLFVQILFFSIFIVV
          	 .*:**:** ***:**: *..* **: :.*.  .    *:*:*: ** :** **.:*::.

MG02290.1 	TAAFHVRIYRSPTTQSLNTTTLWVPMLYVLYIASLLILIRSVFRIAEYSQGSRGLLMTHE
NCU05649.1	TIIFHLRIAANPTAKSYSVAVPWRQLLWVLYVTNTLILIRSVFRMIEYALGWNSILMKRE
FG02084.1 	TLIFHARINRNPTQKSLEIVAPWKKLLFALYAASLSILVRSVFRVAEYVMGKDSALQSQE
AN3508.1  	SIIFHRRMLSTP-MHHMGIDVPWNKYLKILYLVSFLILIRSLYRVAEYIQGKEGVLQSKE
          	:  ** *:  .*  :     . *   *  ** ..  **:**::*: **  *  . * .:*

MG02290.1 	YFLYLLDALPMFAVCALFNWMHPSKVIG----------------------RCSHVEGGGG
NCU05649.1	VYLLVLDGMLMVIVSVAFMRYHPSKFLVGYKQVGMRSAIDLEGSTVEGDYTMTSYGGGGG
FG02084.1 	FWIYIFDALLMSLVVVSLNWFHPSRVINGALDR----------------KRIVSQDEYML
AN3508.1  	VFIYVLDASLMLVCCVILNVWHPSNVVSG---------------------KQALYKHAED
          	 :: ::*.  *    . :   ***..: .                               

MG02290.1 	AGVKTES---------------RQRLGV----------------------
NCU05649.1	GGQRRPSTNLKAHDSSRLEADRRDSLMLGQHRHHRQTPSDDTSSYTPLRR
FG02084.1 	EGQRHDGE--------------RQRYSLSPVRPKGNN-------------
AN3508.1  	LEMLTNSG--------------RTNF------------------------
          	      .               *
```
