# Supplementary material for: Patterns of Intron Gain and Loss in Fungi
Source: PLoS Biol. 2004 Nov 30;2(12):e422. doi: 10.1371/journal.pbio.0020422 (PMC532390; doi:10.1371/journal.pbio.0020422)
Supplement: Table S1 — Also available at http://genes.mit.edu/NielsenEtAl/. (4.3 MB ZIP). [file pbio.0020422.st001.zip › NielsenEtAl/html/115.html]

AN3082.1.NCU06869.1.MG06570.1.FG10795.1


```
 CLUSTAL W (1.82) Multiple Sequence Alignments - Introns Inserted


Sequence 1: NCU06869.1	1002 aa
Sequence 2: MG06570.1	962 aa
Sequence 3: FG10795.1	964 aa
Sequence 4: AN3082.1	1005 aa
Alignment Length: 1047 aa
Number Identitical Residues: 411 aa
Alignment Score (without introns) 20671


MG06570.1 	MFTFSPLQGALSEAT-ASQSLLELDGGVKVLIDIGWDETFDVEKLKEVEK2QVPTLSLIL
NCU06869.1	MFSFCPLQGALSDSS-ASQSLLELDGGVKILIDVGWDETFDVEKLKELGK~QAPTLSLIL
FG10795.1 	MFTFCPLQGALSDSS-ASQSLLELDGGVKVLVDLGWDETFDVEKLKEIEK2QVTTLSLIL
AN3082.1  	MFTFTPLLGAQSSASKASQSILELDGGVKILVDVGWDDTFDPLDLVELEK2HVSTLSLIL
          	**:* ** ** *.::.****:********:*:*:***:***  .* *: * :..******

MG06570.1 	LTHATVPHLSALVHCCKNFPLFARIPIYATQPAIDLGRTLIQDLYSSTPAAATSIPDSAL
NCU06869.1	LTHATVPHLAAYAHCCKHFPPFQRIPVYATRPVIDLGRTLTQDLYASTPLAATTISSASL
FG10795.1 	VTHATASHLAAYAHCCKNIPQFTRIPVYATRPVIDLGRTLIQDLYTSSPAAATTIPQSSL
AN3082.1  	LTHATPSHIGAYVHCCKTFPLFTQIPVYATSPVIALGRTLLQDVYESAPLAATFLPKASI
          	:**** .*:.* .**** :* * :**:*** *.* ***** **:* *:* *** :..:::

MG06570.1 	AEASYS-------------FSQTQTNGHGFLLQAPSPDEIAKYFSLIQPLKYSQPHQPLA
NCU06869.1	AEVSYAS-----------GYSQAASAENTFLLQPPTPEEITKYFSLIQPLKYSQPHQPLP
FG10795.1 	TESAYS-------------LTQTATTARNLLLQSPNSEEIARYFSLIQPLKYSQPHQPLP
AN3082.1  	SEPGASTSAASAASVTEADGSADATSAGRILLQPPTTEEIARYFALIQPLKYSQPHQPIP
          	:* . ::::::::: :.:. :   :    :***.*..:**::**:*************:.

MG06570.1 	SPFSPPLNGLTITAYNAGHSLGGTIWHIQHGMESIVYAVDWNLARDNVYAGAAWMGGGHG
NCU06869.1	SPFSPPLNGLTITAYNSGRTLGGTIWHIQHGLESIVYAVDWNQARENVFAGAAWLGGNHG
FG10795.1 	SPFSPPLNGLTITAYNSGHTLGGTIWHIQHGLESIVYAVDWNQARENVFAGAAWLGG--A
AN3082.1  	SPFSPPLNGLTLTAYNAGHTVGGTIWHIQHGMESIVYAVDWNQARESVVAGAAWFGG--S
          	***********:****:*:::**********:********** **:.* *****:**  .

MG06570.1 	G-GGAEVIEQLRKPTALVCSTRTAEGGLT---RAARDKQLLDTMRMAISRGGTVLIPVDS
NCU06869.1	GAGGTQVIEQLRKPTALVCSSRTPDAALP---RAKRDEQLMESIKLCIARGGTVLIPVDS
FG10795.1 	GGGGAEVIEQLRKPTALICSSRGADRTAQPGGRTKRDEQLIDTIKACVTRGGTVLIPVDS
AN3082.1  	GASGTEVIEQLRKPTALICSTRGGDKFALPGGRKKRDEILLDMIRSTLVKGGTVLIPTDT
          	*..*::***********:**:*  :    ...*  **: *:: ::  : :*******.*:

MG06570.1 	SARVLELAYLLEHAWRSEA-STEGGGLSTAKLYLAGRSVHSTIKLAKSMFEWMDNSIVQE
NCU06869.1	SARVLELSYLLEHAWRKEV-AKDNDVFKSAKLFLAGRTISSTMKNARSMLEWMDDSIIRE
FG10795.1 	SARVLELSYLLEHAWRTDA-ASEGGVLKSAKLYLAGRNMSSTMRYARSMLEWMDDSIVQE
AN3082.1  	SARVLELAYALEHAWRDAARDTQDDVLKRGGLYLAGRKVNTTMRLARSMLEWMDESIVRE
          	*******:* ******  .  .:.. :. . *:****.: :*:: *:**:****:**::*

MG06570.1 	FEA--GADQGFRRTNGAGGNADAKGKDG---GPFDFKYLRLLDRKAQVLKLLEP-STD-E
NCU06869.1	FEA--FADESRRNNRRDEGNHQTGP------GPFDFKYLRLLERKAQIDKILQQ-SDDAE
FG10795.1 	FEA--FAEDQRRVN--GANNKKEGG------GPFDFKYLRLLERKAQIARLLSQNVENAG
AN3082.1  	FEAAEAADTAGQNNDGQRSDQRQGKTDNKGLGPFTFKHLKTVERKKKLEQLLND------
          	***:. *:   : .    .:     ..... *** **:*: ::** :: ::*.       

MG06570.1 	LRGKVILATDTSLEWGFSKDIISAIANDSRNMVILPE-------KPAESSRDNPSISRQL
NCU06869.1	PRAKVILASDTSLDWGFSKDILKSIAADARNLVILTE-------KPNLEPNQKPSISRTL
FG10795.1 	TEGRVILASDSSIEWGFSKDLIKGLAQDSRNLVILTD-------KPGLSKNGNPSIARTL
AN3082.1  	PTPKVILASDSSLDWGFAKESLRLLAGGENNLLLLTDPLHYSKFSDKHTDSHRRTLGSMI
          	   :****:*:*::***:*: :  :* . .*:::*.:.   :. .       . ::.  :

MG06570.1 	WRWWKERRDGVADEQSSGAGSAEQVFAGGRELQIRESKKVPLADSELSIYQQWLATQRQL
NCU06869.1	WEWWKERRDGVATERTSNGDTFEQVYAGNRELEIETAERKGLEGDELNVYQQWLATQRQL
FG10795.1 	WDWWKERKDGVSVEQNSNGDNIELVYAGGRELEIREPQRHALEGDELALYQQWLATQRQL
AN3082.1  	WQWYEERQDGVALEKGSDGEMLEQVHSGGRELSWTDIQRAPLEAGEQRLYQQYLATKRQF
          	* *::**:***: *: *..   * *.:*.***.    ::  *  .*  :***:***:**:

MG06570.1 	NATVQGGGASALEASADVADDVSSESSSDSDDSENEQQGKALNAS--TTQASRKKVVLQD
NCU06869.1	QATLQSGGTNLLEAPGDVLDDADSDTDSESEGSDTEQQGKALNIANTMAQASRKKVVLRD
FG10795.1 	QATQQSGGAAGLEAAADVVDDASSESSSDSEDEDGEQQGKALNVSTAIAQAGRKNVVLKD
AN3082.1  	QDTAQARGQENLDTAADALDDRSSTS---SEESDSEQQGRVLNFSTSLAHSNRNKLGLSD
          	: * *. *   *::..*. ** .* :   *: .: ****:.** :.: :::.*::: * *

MG06570.1 	EDLGVMILLKKPGVYDFPVKGKKGRERMFPLAVRRKRNDEFGELIRPEDYLRAEEREENE
NCU06869.1	EDLGVTILIKKENVYDFNVRGTKGRDRMFPVAMRRRRADEFGELIRPEDYLRAEEREDAE
FG10795.1 	EDLGINVLIKKKGVYDFDSRGKKGRERTFPLTIRRKRQDDFGELIRPEDYLRAEEKEEDG
AN3082.1  	EDLGVNVLLRRKNVYDYDVRGKKGRERMFPYVAPRKKGDEYGEIIRPEEYLRAEEREE--
          	****: :*::: .***:  :*.***:* ** .  *:: *::**:****:******:*:  

MG06570.1 	RPDTQQLQSDGQ--DGFGQKRKWDDAGSRNAANG---LNRR-----GQRGQADDADAAQA
NCU06869.1	NQEAGQVNTNNQEPEGLGKKRKWEDIGTAGRGRGGAGPNKRPHHHDRRRLSAGEADAAPF
FG10795.1 	QDSANVEMTDDK----LGKKRRWDDVVKSG--TG---ANKRPQ---AMRAGSHDGEEAGA
AN3082.1  	-IDMQQRRTESQ--LKLGQKRRWDETQSAG---G---AARKQGVDSTERKDTDMLDNLSM
          	  .     ::.:    :*:**:*::  . .   *     ::    .  *  :   :    

MG06570.1 	AS-GPAPDELDLVEDVEEEVVTGPAKLVHTSTTVSVNLRLALIDFSGLHDRRSLAMLIPL
NCU06869.1	SENGPAGDDLSDLEDEEDETLNGPAKLVVTKETIPVRLRIAFVDFSGLHDKRSLTMLIPL
FG10795.1 	GD-GFVPDELDTVEDVETEEPVGPCKLSYQTETVQANLRIAYVDFSGLHDKRSLNMLIPL
AN3082.1  	TDIGDDTDTAAAPGEEDDQAFEGPAKAIYEKATLTINARLAFVDFTGLHDKRSLEMLIPL
          	 . *   *      : : :   **.*    . *:  . *:* :**:****:*** *****

MG06570.1 	IQPRKLILVAGSADETEAVADDCRR---------------NAIEVFTPPVGAVVDASVDT
NCU06869.1	IQPRKLVLVAGGKDETLALASDVKKLL-----TAQSTGTESAIEVLTPAVGTTVDASVDT
FG10795.1 	IQPRKLILVGGERDETLSLAEDCRRALGVDKSNPDNTGSERSVDVYTPEVGVVVDASVDT
AN3082.1  	IQPRKLILVGGMKEETMALATECQKLLG--VKTGADAPSPTAAVIFTPTNGEIIDASVDT
          	******:**.*  :** ::* : ::  .   ..  .: :  :  : **  *  :******

MG06570.1 	NAWVVKLADPLVKRLKWQQVRGLGIVTVTAQLTATPAAQKNGIPLLIADDDG-ANKRQKI
NCU06869.1	NAWVLKLADPLVKGLKWQNVRGLGIVTVTGLLLPGGEFQ----PIEVGDGDGDAAKRQKL
FG10795.1 	NAWVVKLADPLVRKIKWQNVRGLGIVTITGQLLATHLNE------AAAADEDVANKRQKT
AN3082.1  	SAWTVKLSNNLVRRLKWQHVRTLGVVTLTGQLKAPEPVS------TDEDAINSPNKKQKL
          	.**.:**:: **: :***:** **:**:*. * .    .            . . *:** 

MG06570.1 	KAT-GVDDQ-EPTAEDED---------VGVMPTLDVLPVAMVSASRS-AAQVLHVGELRL
NCU06869.1	EDS-SETPT-TSTALVKTG--TNTSPTTASLPTLDLVPPTLASSLRSQAAQPLHVGELRL
FG10795.1 	EEPPSSTTL-TNTAAAIP----------SATPVLDVLPANLISAVRS-AAQPLHVGDLRL
AN3082.1  	VEETSTPEQPTPTFQPQPTEPQQTTDKPDRYPVLDILPPNMASGTRS-MTRPLHVGDLRL
          	   ..    .  *      .. .:: .    *.**::*  : *. **  :: ****:***

MG06570.1 	ADLRRTMQNLGHSADFRGEGTLLIDGTVVVRKTAA--GRVEIESVGVP-----GTGGG--
NCU06869.1	ADLRRAMLSAGHKAEFRGEGTLLIDDVVVVRKSTAQGGRIELESVGLPSDTMPGTTSGGL
FG10795.1 	ADLRRAMQSAGHTAEFRGEGTLVVDGTVAVRKTSA--GRVEVESVGMP------------
AN3082.1  	ADLRKIMQNAGHKAEFRGEGTLLIDGFVAVRKSGT--GKIEIEAAAYQAG----PSAG--
          	****: * . **.*:*******::*. *.***: :  *::*:*:..  :.    . ..  

MG06570.1 	--PAARMGGTFYAVKKTIYDGLAVVAGA
NCU06869.1	LDAAMKVGGTFYAVKKKIYEGLAVVAGA
FG10795.1 	--TARRS--TFYEVRKMIYDNLAVVAGA
AN3082.1  	---FAQGAGSFLAVKQKIYEGLAVVAGG
          	     : ..:*  *:: **:.******.
```
