# Supplementary material for: Patterns of Intron Gain and Loss in Fungi
Source: PLoS Biol. 2004 Nov 30;2(12):e422. doi: 10.1371/journal.pbio.0020422 (PMC532390; doi:10.1371/journal.pbio.0020422)
Supplement: Table S1 — Also available at http://genes.mit.edu/NielsenEtAl/. (4.3 MB ZIP). [file pbio.0020422.st001.zip › NielsenEtAl/html/1150.html]

AN5986.1.NCU01906.1.MG02921.1.FG08941.1


```
 CLUSTAL W (1.82) Multiple Sequence Alignments - Introns Inserted


Sequence 1: MG02921.1	312 aa
Sequence 2: FG08941.1	306 aa
Sequence 3: NCU01906.1	320 aa
Sequence 4: AN5986.1	314 aa
Alignment Length: 326 aa
Number Identitical Residues: 153 aa
Alignment Score (without introns) 7835


MG02921.1 	--MADKDFTLNTGAKIPAFGLG1----------TWQGDKGVIKEAVLTAIKSGYRLIDGA
NCU01906.1	--MATKTFKLNTGANIPALGLA1NPSPPPPSIGTWQGESTQVKDAVVAALKSGYRLIDTA
FG08941.1 	--MAPSTFKLNTGQEIPAVGLG1----------TWQSPAGEVEKAVTYALKDGYKLIDCA
AN5986.1  	MSLADTTYKLNTGAEIPALGLG1----------TWQSAPGEVSAAVYHALKVGYRHIDAA
          	 ::* . :.**** :***.**.           ***.    :. **  *:* **: ** *

MG02921.1 	YVYGNEEEVGQGIREAISSGIVKREDLFVVSKCWATYTTRCELGLDQSLKLLGLDYVDLY
NCU01906.1	YCYGNEEHVGAGLKEAFDQGIVKREDVFVVTKLWATYTSRAEEGLEKSLRNLGLEYVDLF
FG08941.1 	YCYGNEEEVGAGLKAAFKAG-VKREDIFVVTKAWATYNTRVELALDKSLKALGLDYVDLF
AN5986.1  	QCYGNETEVGEGIKRALSEGIVKRSEIFVTTKLWCTYHTRIQQALDLSLSKLGLDYVDLY
          	  **** .** *:: *:. * ***.::**.:* *.** :* : .*: **  ***:****:

MG02921.1 	LV0HWPILMNPEG1NDEKFPKHADGSRDIIHTHNHVDTWKLMEKLPATG--KTKAVGVSN
NCU01906.1	LV0HWPLLMNPEG1NDDRFPKLPNGERDILRDYSHVQIWKNMEKLVGSG--RTKAIGVSN
FG08941.1 	LV0HWPLLLNPDG~NDDKFPKKPDGSRDVIRDYNHVDGWKLMEKLPATG--KTRAVGVCN
AN5986.1  	LV~HWPLAMNPNG~NHDLFPKLPDGSRDLVREHSHVTTWKGMEELITNNPDKVKAIGVSN
          	** ***: :**:* *.: *** .:*.**::: :.**  ** **:*  ....:.:*:**.*

MG02921.1 	0YSKAWLEQLLPHATTVPAVNQVENHPQLPQQELVDFCKEKGIHIMAYSPLGSTGGPLLT
NCU01906.1	0YSKRYLEELLPHAKIVPAVNQIENHPQLPQQEIVDFCKEKGIHIMAYSPFGSTGSPVTS
FG08941.1 	0YSKKYLEELLPHATIVPAVNQIENHPELPQQEIVDFCKEKGIHIEAYSPLGSTGGPVMS
AN5986.1  	~YSKRYLEQLLPQAKIVPAVNQIENHPALPQQEIVDLCKEKGILITAYSPLGSTGSPLFK
          	 *** :**:***:*. ******:**** *****:**:****** * ****:****.*: .

MG02921.1 	AEPVVKIAEKHSISPAAVLLGYQI1ARGI~TVIPKSVNPDRIKANAQLKDLDAEDMKLLN
NCU01906.1	AEPVIKIAEKHGVKPTTVLLSYHL1YRGS~TVLPKSTNPERIEANAKLIELDAEDQKLLN
FG08941.1 	AEPVVKIADKKGVSASTVLLSY--~-HGN2TVLAKSVNPERITANKTIVDLDDEDMKALN
AN5986.1  	AEAIVAVAERRGVTPASVLLSWHL1ARGS~SVLAKSVTPSRIEENRKLVKLEPEDVELIG
          	**.:: :*:::.:..::***.:    :*  :*:.**..*.**  *  : .*: ** : :.

MG02921.1 	DYSEQLAKDGKLNRYVFPPFGTDFGFPDKVGQ
NCU01906.1	DYSEGLVKEGKVQRYVYPPFGVDFGFPDKS--
FG08941.1 	DYSAELQKNNQVKRYVYPPFGIDFGFPDKS--
AN5986.1  	KYSAELAATNGFQRYVYPPFGVDFGFPDKS--
          	.**  *   . .:***:**** *******
```
