# Supplementary material for: Patterns of Intron Gain and Loss in Fungi
Source: PLoS Biol. 2004 Nov 30;2(12):e422. doi: 10.1371/journal.pbio.0020422 (PMC532390; doi:10.1371/journal.pbio.0020422)
Supplement: Table S1 — Also available at http://genes.mit.edu/NielsenEtAl/. (4.3 MB ZIP). [file pbio.0020422.st001.zip › NielsenEtAl/html/1151.html]

AN4663.1.NCU04698.1.MG01296.1.FG09525.1


```
 CLUSTAL W (1.82) Multiple Sequence Alignments - Introns Inserted


Sequence 1: MG01296.1	594 aa
Sequence 2: FG09525.1	580 aa
Sequence 3: NCU04698.1	622 aa
Sequence 4: AN4663.1	558 aa
Alignment Length: 629 aa
Number Identitical Residues: 175 aa
Alignment Score (without introns) 9998


MG01296.1 	MAPK--KRTAASKGK--------DAKPAAVDIDPNTSFTP--ESFEKELKALAAKAQTET
NCU04698.1	MPPKATKRGKASKATTTTTTTPEESEPKTTATSAPDLFTKSKEAYEQELKDLAKKARSQ-
FG09525.1 	MASK--KDGQLAKAT--------GAP---------EGFTP--ERFEKELKDLAAKAKENT
AN4663.1  	-------------------------------------MAP--------FRSIYEKDATK-
          	                                     ::         :: :  *   : 

MG01296.1 	WGNAVREQAWVYVRPIALLSLLAVYSNVSQLSLSPVYGAIPSSLYHSSLVIASCFVGWSA
NCU04698.1	-KGYWAGQFAIYTHTFILLALIAIASNVSQLVLSPVYGSIPAAIWHSKLVMAGCFTGWAS
FG09525.1 	FANRATKQALVYFKTLMLLGLLGVASSASQLNLSPIYGSIPAAVTHSTALKVACFIGWAG
AN4663.1  	----------KLVVGAALLVLAAFYSYVFLLTLAPVYGSTPSHIFHGYGVGIAGVAGWFS
          	                 ** * .. * .  * *:*:**: *: : *.  :  . . ** .

MG01296.1 	NLLLQRALPRKVKLVHLVPIVALYIPMTQHLLFKYSNVFGARYGPLVTELLTLSPLIVVV
NCU04698.1	NIFLDRALP--IEPVELLPLIAMYIPPAQYLLFKASGYMGAYYGPLVTELLTLFPLITVS
FG09525.1 	NLILNMYLP--LSTMQLLPLIALNIPAIQFLTGCFTDRLGNWWGPLLIESLTIYPLAISS
AN4663.1  	KDIVDRVSG--RKAIYAIPVLAFFLPVVQYFVSQQSSALGNPAGPIFTEVLALYPLVLLS
          	: :::       . :  :*::*: :*  *.:    :. :*   **:. * *:: **    

MG01296.1 	VACCATYLEG-ADFSLLPKWLADPAPGALSWSVYRGMEVLSGHWLQNNIGQTFVATRMGA
NCU04698.1	SACVATNLEY-ADFGPLPKAIAEAVPGLGSFGFFKVAEKLSGDFIVQNIGKNFYMTRIGL
FG09525.1 	AAAVADILED-ADLSVLPKFFADAAPGIVSWSLYRLAENTSMDKLQGVIGSTFVLTRVGL
AN4663.1  	VACAGKLVQAGLNLQRHGDLVAEHIPLLGSYVIYSAGEHLIKAFLSRFIGSTVLLSRAGL
          	 *. .  :: . ::    . .*:  *   *: .:   *      :   **...  :* * 

MG01296.1 	QMVLGGLFTMLAPSKLLLLAIPGLLHTVTLNPHVMTPYAMDRLNGTLQTQNWTIIDRKES
NCU04698.1	EGLLAASYTTIAPSKFLLLGVPALLHTTLLNPHLASPMALSRLNSGLEKEGWHVLDRRDS
FG09525.1 	ELFVGAIYALMAPSKYLVLAIPALLHTAVLNTHVMTPMATESLNNTLLAQNWTLLDRRES
AN4663.1  	QILIAIFYAAAVPSKALLLAIPAFLFSVTSNTHLPLGHTTTALNNIIADDGFALVARQDS
          	: .:.  ::  .*** *:*.:*.:*.:.  *.*:    :   **. :  :.: :: *::*

MG01296.1 	LTGYISVIENKEMGFRVMRCDHSLLGGEWVRYG------RKIVSEPVYGIFVMLEAVRLV
NCU04698.1	ITGYISVVDSLNDGFRVLRCDHSLLGGEWVKFKDTPRFKGNQVAEPIYGVFAMLEAVRLV
FG09525.1 	LTGYVSVIESLEMGYRLMRCDHSLLGGQWVHVG------GRKVSEPIYGVFVMLEAVRLV
AN4663.1  	TTGYISVLDNLEDGFRVMRCDHSLLGGQWIKKR--PNYTPPAVKDPIYAVFTMLEAVRLV
          	 ***:**::. : *:*::*********:*::    .  .   * :*:*.:*.********

MG01296.1 	KREV--PVPDKDAKALIV2GMGIGTTPSALIAHGIDTTVVEIDPTVYEFAAKHFHLLPNH
NCU04698.1	QVPN--PVPDNEAKALVI2GLGIGTTPAALVAHGINTTVVEIDPVVHEFASKYFQLPSNH
FG09525.1 	ERET--PLADNEASALNI2GLGIGTTPSAFVRHGIDTTIVEIDPAVHEFAQKYFDLRENN
AN4663.1  	ETAHGIPRADAGSNALVI2GLGIGTTPGALISHGIDTTIVEIDPVVHKYALQYFDLPENH
          	:   . * .*  :.** : *:******.*:: ***:**:*****.*:::* ::*.*  *:

MG01296.1 	TPVIEDAVAYAKLLASSSD--KRFDYIVNDVFTGGAEPVELFTLEFLQDLRTLLNPDGVI
NCU04698.1	TAVIEDAVSYTDRLVNETQGVGQYEYIIHDVFTGGAEPVALFTYEFLQNLHSLLKPNGVI
FG09525.1 	PAAIHDAVSYTADLVNQS---KTFDYIVHDVFTGGAEPVDLFTLEFLQGLGDLLKPDGVI
AN4663.1  	TPIIEDARAFVQRSRNAPQ-PKQYDYIVHDVFTGGAEPVELFTYEFISGLHALLKDDGVI
          	.. *.** ::.    . ..    ::**::********** *** **:..*  **: :***

MG01296.1 	AI0NYAGDFALPPPRIVLNTIKQVFPS-SCRMYREHARNETEVAETK-VDFTNMVIFCRK
NCU04698.1	AI0NYAGDFSHPPPLLITNTIRSVFGSSSCRIFREHPRDEAQAAAHNGRDFTNMVFFCTK
FG09525.1 	AI0NYAGDFGLPTPALVYRTIKQVFP--SCRTFREHPRDEKNVEKWG-SDFTNMVIFCRK
AN4663.1  	AI0NYAGDISLYSTALSIRTIKSIFP--TCRLFREAAAPEIG------PDFTNMVIFCTK
          	** *****:.  .. :  .**:.:*   :** :** .  *         ******:** *

MG01296.1 	TETPAP-LTFRTATEKDVLDSASRVHFLLPKHEVLESEFLSN---------DTSILRRND
NCU04698.1	VSTPTQGITFRNPTPRDLLNSPSREAFLLPKFEVTDDDLLRAVNGSVEEAKKLGILRKNE
FG09525.1 	--TPGD-IKFRRPRTGDFLDSQVRRNLLLPKHEIKEQVFLDDE--------GTDILAKND
AN4663.1  	--SRGAPITFRDPVPEDFLGSRFRSRYLVPKHEVDAAQFDNVGLEDGPQGHGRRVLVDKE
          	  :    :.** .   *.*.*  *   *:**.*:    :      .  ..    :*  ::

MG01296.1 	TKILEKWHKKSAMGHWDVMRTVVPPPIWENW
NCU04698.1	TATLEKWQTESALGHWEIMRGVLPEVVWVNW
FG09525.1 	TSKVTKWHQSSAAGHWNIMREVLPGKVWEQW
AN4663.1  	VGRLHKYQDRSALEHWGIMRTVLPDRVWEGW
          	.  : *::  **  ** :** *:*  :*  *
```
