# Supplementary material for: Patterns of Intron Gain and Loss in Fungi
Source: PLoS Biol. 2004 Nov 30;2(12):e422. doi: 10.1371/journal.pbio.0020422 (PMC532390; doi:10.1371/journal.pbio.0020422)
Supplement: Table S1 — Also available at http://genes.mit.edu/NielsenEtAl/. (4.3 MB ZIP). [file pbio.0020422.st001.zip › NielsenEtAl/html/1152.html]

AN7025.1.NCU06609.1.MG08050.1.FG05254.1


```
 CLUSTAL W (1.82) Multiple Sequence Alignments - Introns Inserted


Sequence 1: MG08050.1	393 aa
Sequence 2: FG05254.1	368 aa
Sequence 3: NCU06609.1	433 aa
Sequence 4: AN7025.1	369 aa
Alignment Length: 449 aa
Number Identitical Residues: 71 aa
Alignment Score (without introns) 4914


MG08050.1 	MAIYSAVPPP-----------PESPAMSASGEAHHASGNIHHGHHHTPSMASTATLDIEA
NCU06609.1	MAITKVAGPVGHDSDDDYDEALHHPQPQQPADIIASQPLPLPVNQSTISNVSTAAVDIEA
FG05254.1 	MAIYSSVPPP-----------EQQPTTTTPTPTATNPPLAAIHVQSPPTPVKNSSIDIDA
AN7025.1  	MAIHPSFPSQ----------------------RDGRGPLSPQQAQAISAWTEQAAASLQD
          	***     .                                   :   : .. :: .:: 

MG08050.1 	WTVSALESLSVS-TEARGTGIPFSIPLDNSIPA-PRTNQ---------ARVSIDPG----
NCU06609.1	WTVAALESLRIA-NNARGAGHPLLIPLDGAAQGEAAAAAGPTDPALKLRNVVFDDGDDTY
FG05254.1 	WTLSALQSLNVS-PVARGTGIPLTIPIDEAVKVQPKSPE---------RNVDFDER----
AN7025.1  	LTITDSAPTSAGNATLRGTTVSLSIPLDDPVPAAEGGAP-------RVKTPGQGTEEARK
          	 *::   .   ..   **:  .: **:* .                       .  .   

MG08050.1 	LGGIAPPRRPPSRRDSQRRRELVAKGNEGSRQRRRWEND~RLVHVPGAQPPEPIDFQVGP
NCU06609.1	AANVAPPRRPPSRRDSMKRREALLKGKEGSRQRRRWEND1RLVGVPNVQPPLPSDWQVGP
FG05254.1 	EAPTSIPKRPLSRRDSQRKRDLVLKGKEGSRQRRRWEND1RLMHVPNVQPPLPSDYEVHP
AN7025.1  	IPAVSFRRREPLRRDSLKRREALLKGKDGSRRRQRWEND1RLLHNPWAEPPSPNDWMPQP
          	    :  :*   **** ::*: : **::***:*:***** **:  * .:** * *:   *

MG08050.1 	THVVHDYVPYQVAQLWDRGLRDQIEEDRARAAALKRKRQQQIDAGL----------LSAG
NCU06609.1	TYPIHDNMPYHLAQYWDKGLRERVEERKAAFAVHRKKTVTATSHQLGGATTAVSLVVSSS
FG05254.1 	THTIHR-VPYQLAQFWDRGVRQRVEDKTARLQAERKKQQ-----------------LKSG
AN7025.1  	THTRHEPMPYFLAPLWDKHYAHIERRSPGDAKTER-------------------------
          	*:  *  :** :*  **:   .  .   .   . :                         

MG08050.1 	AESGAVAVGRVPRELRATAKKTPALKGWVRTLEEPVRQFLVEEDARIRADAAEKERKRLE
NCU06609.1	VAKTRADVGKVPKDLRATAKKTPAVKAWLRVLEEPVREFLVQQQRGQNAAAKREQQ---Q
FG05254.1 	SATG-LGAGEVPRDLREATKRSPVVRSWVRSLEEPLRQYLASQQAVVTPSAEAAE-----
AN7025.1  	--------HHIPKELRLKLKHARAARGMLQDLEEDIRQFIERWNERQLVRQ---------
          	         .:*::**   *:: . :. :: *** :*:::   :                

MG08050.1 	KEKEKEARAASEGESGLDSEDEEIVFIGRRQATPQ-KSSGPGWKKAHRETRD----KGAI
NCU06609.1	QQKQQKARDSGNQSDELGSDEEEILFTGRKTATAAAAGGKPPIKNASSEKGTDKGIQLLP
FG05254.1 	---EDSDSAADQ----MDSDDEEIVFVGRNGAMRELREKKATWKHAHREVS-----QETV
AN7025.1  	-----KDGLADAPSSSEEDSEDEVVFVGRNGQMHDSPDRRKKLQSMRETMSS---HNERD
          	     .   :.  ..   ..::*::* **.             :            :   

MG08050.1 	DSGVVFDSLGDDES-SAFK2RWLTHSISDYYGLSSHSATTGTPPRKVVYVGIKPAGSGSK
NCU06609.1	DTRMVLDTLGDDESGASFK2RWLTHSISDYYGLDSKSVTVGNPARRVVYIGLRQVGNGPK
FG05254.1 	DSGMLFDSFGNDES-AAFK2RWLTHTISDYYGIQSRSVNLTNPSRRVVYVGLKTSQG---
AN7025.1  	GEKMVFESLVDDRA-AGFG2RWLVHSIASYYGLHTWSVTVGSPARREAYVGFYPPSSGSR
          	.  :::::: :*.: :.*  ***.*:*:.***: : *..  .*.*: .*:*:    ... 

MG08050.1 	KTTQLPPQ--------------LPRPMWELF
NCU06609.1	RRAAPTRQM------------LIPPPLWEMF
FG05254.1 	--VLPPRT--------------LPRPMWEVC
AN7025.1  	AGLLTQPPSRCRDRVLIQPGEKLPRPLWSQV
          	          . .    .... :* *:*.
```
