# Supplementary material for: Patterns of Intron Gain and Loss in Fungi
Source: PLoS Biol. 2004 Nov 30;2(12):e422. doi: 10.1371/journal.pbio.0020422 (PMC532390; doi:10.1371/journal.pbio.0020422)
Supplement: Table S1 — Also available at http://genes.mit.edu/NielsenEtAl/. (4.3 MB ZIP). [file pbio.0020422.st001.zip › NielsenEtAl/html/1153.html]

AN4558.1.NCU01478.1.MG07450.1.FG06810.1


```
 CLUSTAL W (1.82) Multiple Sequence Alignments - Introns Inserted


Sequence 1: NCU01478.1	899 aa
Sequence 2: MG07450.1	873 aa
Sequence 3: FG06810.1	893 aa
Sequence 4: AN4558.1	854 aa
Alignment Length: 948 aa
Number Identitical Residues: 373 aa
Alignment Score (without introns) 19843


MG07450.1 	MDRMG--FQMPPMGAPP--IMNPPPQIFGGY--DGMPMQLPPD--MTAHMFADHSTLLDD
NCU01478.1	MDHLHGGFQMP-MAPPP--LLNQPPQIFGGYTEHGIPIQQLPHDLAVAQMFGEHG-LLDD
FG06810.1 	MDHMA--FQVPGMAPPI--MNHPPPQVFGSY--DGIPQLHPE---IAAQMFNDSAMMLED
AN4558.1  	MDGIGEGHGPNGMGYDMPMLMNQHTPIFGAYGPEGSPVTTALP--NPSLHDEASMSIGDD
          	** :  ..    *.   . : :  . :**.*  .* *          :        : :*

MG07450.1 	ANEAKRRRIAR0------VEKKRSPPKG2AKYIEGLENRLGRMESLLR--------~---
NCU01478.1	TSEAKRRRIAR0ACDMCRKKKIKCDGKL~PACTHCINYKTDCVFTQVEKKRSPPK-~---
FG06810.1 	ANDPKRRRIAR0ACDMCRKKKIKCDGKM~PSCTHCINYKTECVFTQVEKKRAPPKG2AKY
AN4558.1  	NNDAKRRRIAR0ACDMCRKKKIKCDGKM~PKCSHCINYKTDCVFTQVEKKRNPPKG2AKY
          	 .:.******* :.. .  :* :.  *  .   . :: :   : : :...  .... :. 

MG07450.1 	----------------~LSG~LLGEDD-GATDLGTLEKRLAEKNRESRQASMAATSSPTS
NCU01478.1	---------------G2PTG~LLGDDDNGATDLGTLERKLAEKTAQSRQTSQAAASNPTS
FG06810.1 	IEGLENRLNRMEHLLR~LSG1LLDEDD---DDLGALEKRLMERQHKSRQASMAVGSGPNS
AN4558.1  	IEGLENRLGRMESLLR~LSG~LLSEDD-GKTDLGTLEKRLADRS-----LSAGGYNATNS
          	 .. ..  .  .      :* **.:** .  ***:**::* ::       * .  . ..*

MG07450.1 	PSQTASG---QDG--STPQSALASPEPRMDKDKD-------ERRPSLTPAK-----KEGE
NCU01478.1	PSQAASG---QDGNNSTPRSSLASPLPEAPRDKEG------EKRASIAPEK-----VDKE
FG06810.1 	PTHSTSAPSAIDASAMTPQSSLTSPNPIVKEDKRKSATPAPSTAPSTAPAPNANGEEVKT
AN4558.1  	PTRFSLP---LNGQPSQPASTSRHSTPRVDSHSS-------PRTAATSPES-------QK
          	*:: :      :..   * *:   . *    ..           .: :*           

MG07450.1 	--EQEVETLSEMMCSLVTNNSGETRYIG1SSSGFSIFSPRGIQWVNETTGDNSFQQMISD
NCU01478.1	ETEQEVSALSEMMCSLVTNNNGETRYIG1SSSGFSIFSPKGIQWVNEKMGDSSFQQMISD
FG06810.1 	EEPEEVEALSEMMCSLVTNNYGETRYIG1SSSGFSIFSPKGVSWVNSKTGDDSFQRTISD
AN4558.1  	ESENEVEGLSDMMCSLVTNNCGETRYIA1Y------------------------------
          	.  :**. **:********* ******.                                

MG07450.1 	VSIDDHKWTNWKPEVFQDLFRRPVFIPIPPKPEAMSLLKDYFENFNCMFPLFHQPTFMHL
NCU01478.1	VSVDDHKWTRWKPDIFGDLFRRVIFKELPPKPEAMSLLKDYFENFNCMFPLFHQPTFMHL
FG06810.1 	ISVDDHKWTNWKPEVFSDLFQRPVFRPLPPKTEALSLLQDYFDNFNCIFPLFHQPTFMHL
AN4558.1  	--IDDNKWMYWKPEIFSDVFARRVFKPLPPKEEALSLFRDFFDNFNCMFPLFHEPTFMHL
          	  :**:**  ***::* *:* * :*  :*** **:**::*:*:****:*****:******

MG07450.1 	VERQYSNDPYEGSGWWASFNCALAVAHRLRVMSNLVPQEEDDKAWKYIKNAMGVFSELTM
NCU01478.1	VERQYSPDPYQGSGWWASLNVALAIAYRLRVMSNLVPQEEDDKAWGYMKNAMAVFSELTM
FG06810.1 	VQMQYSSEPYQGSGWWASLNCALAIAHRLRVMSNLVPQDEDEKAWGYLKNAIGVFPELTM
AN4558.1  	VEKQYSRDPYEGSGWWASINVVLAISHRLRVMSNLVPQEEDKKAWLYLKNAMGVLTELTM
          	*: *** :**:*******:* .**:::***********:**.*** *:***:.*:.****

MG07450.1 	RNTDLLSVQALLAM~ALFMQGTPNPQPTFLLIATAIRLSHSIGLHKRATGFNINPIELEQ
NCU01478.1	RNTDLLSVQALLGM~SLFMQGTPNPQPSFLLIATAIRLSHTIGLHKRGTGFNLNPIEIEQ
FG06810.1 	RNTDLLSVQALLGM~ALFLQGTPNPQPTFLLVSAAMRLAQSIGLHKRGTGFNLNPIEIEQ
AN4558.1  	RNTDLLSVQALLGM0SLFLQGTPNPQPSFFLVAAAIRLSHSIGLHKRGSGFGLNPVEAEQ
          	************.* :**:********:*:*:::*:**:::******.:**.:**:* **

MG07450.1 	RKRVFWIAYMLDKD~LCLRSGRPPAQDDNDMNVELPDADPEDNIGNIPLADGKGKMNLFR
NCU01478.1	RKRVFWIAYMLDKD~LCLRSGRPPAQDDDDMNVDLPDEDPADGIGNIPLADGKGKMNLFR
FG06810.1 	RKRVFWIAYMLDKD~LCLRAGRPPAQDDDDMNVELPDADPADNIGNIPLADGKGKMNLFR
AN4558.1  	RKRVFWIAYMLDKD2ICLRSGRPPVQDDDDMNVELPSEDPPDNIGNVPLFDGKGKFNMFR
          	************** :***:****.***:****:**. ** *.***:** *****:*:**

MG07450.1 	VMCELAVIESRVYNRLYATKATKQTDGELLNTIGELDQELEDWKDRIPIDFRPEHEIKAS
NCU01478.1	KMVEISIVESKVYKRLYATKATKQSDGELLNTIGELDQELEDWKDSIPIDFRPEHEIKAS
FG06810.1 	VMCEFATIESEVYKRLYSVQATKQSDGELLNTIGELDQKLEEWKDSIPIDYRPEHDINAS
AN4558.1  	TLCKFSIIESKVYKRLYSATASKQSDGALLNTIGELDRELEEWKDSIPIDFRPEHEIKAT
          	 : ::: :**.**:***:. *:**:** *********::**:*** ****:****:*:*:

MG07450.1 	HTPLMLHVIILHFTYYNALTTIHRMSVHHGYWNSRLSNYAIQGLNAKPLNPRIFSSAALC
NCU01478.1	HTPLILHVVMLHFTYYNCLTTIHRMSIHHGYWTSRLSNFAIQGLNARPLNPRIFSSAALC
FG06810.1 	HTPLILHVVMLHLTYYNCLTTIHRMSVHHGYWTSRLANYAIQGLNARPLNPRVFSSAALC
AN4558.1  	HGPLILHIVVLHFAYYNCLTTIHRMSVHHGYWTSRLSNYAIQGLNARPLNPRVFLSAVLC
          	* **:**:::**::***.********:*****.***:*:*******:*****:* **.**

MG07450.1 	SAAARASISLLKYIPQGDMSVIW2LILYFPVSAHVTLFGNIIQNPLDQRARSDARLMSVV
NCU01478.1	TSAARASISLLKYIPQGDFSCVW2MVLYFPVSALVTLFGNILQNPLDPRARSDAKLMSVV
FG06810.1 	TAAARASVSLLKYVPQGDFACVW2MILYFPVSALVTLFGNILQNPLDPRAKSDTRLMNIV
AN4558.1  	VTAARASINLIKYIPQGDFACVW2LILYYPVSALVTLFANILQNPTDARARSDVKLMNVV
          	 :*****:.*:**:****:: :* ::**:**** ****.**:*** * **:**.:**.:*

MG07450.1 	VQFLSTLGAEAETGGVHRMLAVCNEFERIAKLVIDKAEKENAGRRKRKNNEVTAN-NSNK
NCU01478.1	VTFLSMLGHEAETGGVHRMLGICAEFERIAKVVIDKAEKENSSRRKRKMQEQASSRKAAS
FG06810.1 	VTFLSMLGQEAEQGGVHRMLGICAEFERIAKAVIDKAEKEQSSRRKRKNQDSANKSSANV
AN4558.1  	VNFLSTLVSDESNGSIKRMLGVCGEFERIAQVVLDKAERESQSKKKRKAGPEEPR-DSPQ
          	* *** *  : . *.::***.:* ******: *:****:*. .::***        .:  

MG07450.1 	SSSGNREQQSTPRPQSSSAHTPRAQHVVYTPNGSQMSPAPTHSNHSTPYHGGRMSNQSSP
NCU01478.1	STTASADHASSFNPNATMTPRPPTAGSAATPQPNSGS-VKDHLSPGVQSTRTPQSGHSPF
FG06810.1 	PAARNAAKESVRSASVSSTAHRRSSQAQLSPPSNGDS-------MGAFSVGTPMNDLSPS
AN4558.1  	AASTTSTKKNTANTSATMPFSPPPQYGADSQDSSSNA----ANGATAFTSSQTMPGTSSI
          	.:: .  : .   .. : .    .     :   .  :      .  .        . *. 

MG07450.1 	AMTPNHWPPPGDYVPGSAGMEGMEYGGNVNFAEMTGFTADIRQ-------------SVPP
NCU01478.1	SQSPSPGMTPNGWTDGPTGMEGIDYG---NWADLTGFGAIANMNPADFVMDGLADPSAGD
FG06810.1 	AMS-------AGWPQEFPVPQPHQNG---DYDSAMSYGEGSMH-------------SPGM
AN4558.1  	SDMSGTIPAMPRASQDFTEMLGPNALDGLNFNSQPPLTSTGDV----------------P
          	:  ..   .        .     :  .. :: .                           

MG07450.1 	ATAYFTQPHLPPDLYSLPVSLDWDWAEMSGGAYPSVENGAVGAMGLGQGAPPHGTQH
NCU01478.1	PRSIYQQPMLPQDLFSLPVTLDWNWAEMSGGEYPSVENGNFGSD------VPHQQ--
FG06810.1 	PVSAFQQPLLPQDLFSLPMTLDWSWAEMTTGAYPTVENGNFGGD-------------
AN4558.1  	IAQPFQQPFVPQDLWQMPMTIEWDWADMSTN-FPVFDSGPSH---------------
          	    : ** :* **:.:*::::*.**:*: . :* .:.*
```
