# Supplementary material for: Patterns of Intron Gain and Loss in Fungi
Source: PLoS Biol. 2004 Nov 30;2(12):e422. doi: 10.1371/journal.pbio.0020422 (PMC532390; doi:10.1371/journal.pbio.0020422)
Supplement: Table S1 — Also available at http://genes.mit.edu/NielsenEtAl/. (4.3 MB ZIP). [file pbio.0020422.st001.zip › NielsenEtAl/html/1154.html]

AN0210.1.NCU08027.1.MG07077.1.FG04270.1


```
 CLUSTAL W (1.82) Multiple Sequence Alignments - Introns Inserted


Sequence 1: NCU08027.1	750 aa
Sequence 2: MG07077.1	702 aa
Sequence 3: FG04270.1	671 aa
Sequence 4: AN0210.1	711 aa
Alignment Length: 764 aa
Number Identitical Residues: 262 aa
Alignment Score (without introns) 13975


MG07077.1 	MGKKW1RYGVILDAGSS~GTRLHIYRWQDPGKARKHATKEALQSLPQLETKDKWTKKTRP
NCU08027.1	--MTG~RWGVILDAGSS~GTRLHIYRWKDPEKALVDATPEELRSLPKITTKKKWTKKIRP
FG04270.1 	---MA1QYGVILDAGSS~GTRVYIYKWKNHAKAAKDASAAELKALPKIKLKE--NKKIHP
AN0210.1  	-MGKW1HYGIVLDAGSS0GTRVHVYRWLDNAVARKESGKHNLKSLPEIKTKSDWVKKIHP
          	      ::*::****** ***:::*:* :   *  .:    *::**::  *..  ** :*

MG07077.1 	G~ISTFGTSPNDVGPDHLQDLIDHALKYVPKKKQQDTPVFLMATAGMRLLPEMQQKALLK
NCU08027.1	G~ISTFGEKAGVVGQDYLKELIDHALDIIPADKIHDTPIFLMATAGMRLLPQVQQNAITG
FG04270.1 	G~VSSFAENPSQIGPDHLKQLIEIALDEVPDSKISETPVYLMATAGMRLLPKPQQSALLK
AN0210.1  	G1VSTFADRPEEFGPEHLAELLDFARDIVPDDAIKETPIFLLATAGMRLVEDVKQKLLLD
          	* :*:*.  .  .* ::* :*:: * . :* .   :**::*:*******: . :*. :  

MG07077.1 	ETCTYLRQNTEFALPDCDLHIQVIPGETEGLYGWVASNYLLGGFDNPQNHHHGKGHHTYG
NCU08027.1	AVCTYLRKNTNFSLPDCDLHIQVIPGETEGLYGWIASNYLLGGFDHPEQHAHGQDHHTYG
FG04270.1 	SMCSYLQENTEFILPDCDAHIQVISGETEGLYGWIAANYLLGGFDHPEEHDHGKNHHTYG
AN0210.1  	HICSYARENYDFALPDCNLHIQVIPGVTEGLYGWVATNYLLGSFDSPSDHDHGKGHHTYG
          	  *:* ::* :* ****: *****.* *******:*:*****.** *.:* **:.*****

MG07077.1 	FLDMGGASAQIAFAPNSTEAEKHANDLKRLRLRTLDGQASEFKVFTATWLGFGVNQARQS
NCU08027.1	FLDMGGASAQIAFAPNSTEAQKHANDLKLLRLRTLDGSPAEYKVFTTTWLGFGVNRAREA
FG04270.1 	FLDMGGASAQIAFAPNATESAKHADDLKLVRMRTLDGSPAEYKVFTATWLGFGANQARSR
AN0210.1  	FLDMGGASAQIAFAPNATESEKHANDLTLLRLRNIDGSTQEHRVFVTSWLEFGVREARRR
          	****************:**: ***:**. :*:*.:**.. *.:**.::** **...**  

MG07077.1 	YVKGLLEG-ASVDASEIPDPCMPKGLRTTPTGEVIEDAAGNKNRTLIGTGMFKECQLKTH
NCU08027.1	YVQSLSDLYTTSDAGELPDPCLPKGLRLSLDGTPVSKPKKGE-TTLIGTGAFDECLRKTY
FG04270.1 	YVERLQEHYNTDTTHELPDPCMPQGLRTTPDGELVEKISDKT--VLVGTGKFDECLRVTY
AN0210.1  	YLDSLQSALSADNVKELPDPCLPAGLRTTLDGRPLEDSETKH--YLLGTGKFDECLRGTY
          	*:. * .   :  . *:****:* *** :  *  :..        *:*** *.**   *:

MG07077.1 	PLLGKDAPCNDHPCLLNGQHVPAIDFEVNHFVGVSEYWHTTHGVFG--KGDKAYDFATYQ
NCU08027.1	PLLGKDKPCADDPCLINGQHVPAIDFDVNHFVGVSEYWHTTHGVFGG-KENEAYDFTTYQ
FG04270.1 	PLLGKDKPCDDQPCLVNGQHVPGIDFDINHFVGVSEYWHTTHGVFG--KKHKAYDLATYQ
AN0210.1  	PLLDKDAPCEDQPCLLHGVHVPAIDFDVNHFVGISEYWHTTHEVFEMGHTDKAYDFNTYQ
          	***.** ** *.***::* ***.***::*****:******** **  .: .:***: ***

MG07077.1 	KRVQDYCTQDWDAIESQLEPRKK---HSAKNAQEACFKAAWLMNVLHEGIGIPRVGLEHT
NCU08027.1	KRVKDFCGRDWNSIEPSLDAHKK---FAVKDAQEACFKASWLINILHEGIGVPRIGVEEL
FG04270.1 	NSVMEYCSRDWSDIKNDLDKRKKSPEKKAEEAQLACFKASWLINMLYDGIGIPRVGLEGG
AN0210.1  	HRVEEFCSQDWESIEQGIAEHKWGKKLDRELAYEVCFKASWIINVLHDGIGVPRVGLEST
          	: * ::* :**. *:  :  :* . .   : *  .****:*::*:*::***:**:*:*  

MG07077.1 	PK--VNVSKGALENAKDKGFIDPFKAVDEIDGVEVSWTLGKMLLYAAGQVPPPEAMQAKA
NCU08027.1	PAPGLNASKGAIETAKQKGFLDPFHPVDKIDGIEVSWTLGKMVLYAAGQVPP--KTGDDR
FG04270.1 	AN-------NTVKDDGEKSFNDPFRPVDTVDGVELSWTLGKMVLYAAGQVPP----SSSE
AN0210.1  	SASSVNGTKEVLENGMHKGFLDPFQAVNKIDSTEVSWTLGKMVLYAASQVPV---ETAES
          	. .. . :. .::   .*.* ***:.*: :*. *:*******:****.***       . 

MG07077.1 	LPVGFGSNVDG-----VPPDFNFAGSSWSPIQ----------------QEDDDHDDDWSD
NCU08027.1	FPVGFGTNIPSAGATELPPDFQYAGSTWTPLSGGASHNDSPYRPAVNGTTDDGSDWDLEA
FG04270.1 	LPVGFGSNVEKG----IAEDFQHAGS--SPIAP--------------HTGDDDDDNDDFE
AN0210.1  	LPVGFGSNVAG-----VPSDFQYPSAELLPNP-------------------ETLHGESWQ
          	:*****:*:       :. **:...:   *                     :  . :   

MG07077.1 	DIVDKVKSSKSTSGFLIFVLILFILGYFLRKRERRMRLFSKVNSMFRRSRKPGSPRKASR
NCU08027.1	ENLLGKTKAKTTHSLLIVFFIFLALLFFFRKKDRRMRFYGRVNNLFHKPRRSGLFGKMAS
FG04270.1 	DILK--KPGKSTGGVVAFIIIILLAAYLLRKPERRRKIFSMVK----RRKRSG---KSGR
AN0210.1  	DALLEGNSSRRIPGIILFVLIIILALFLLCGRTRRLKVYHKVNNLFSCGRGPRLIQQRKR
          	: :   . .:   ..: ..:*::   :::    ** :.:  *:.     : .    :   

MG07077.1 	G------LAGLRNKLFWR--TSSTSYERILEDGDAEQFALGD-----IDSDVSDGSDSSD
NCU08027.1	SGHNNGPLSSLTNKLFGRRSLSSGSYERVLEEGEAAEFELNDSHFRNSASSYFDHNNDLN
FG04270.1 	G-------SSLVSKIFGR--RSGPSYERVMEEGDYSDFELGD-----VDSDDNDHSDSSS
AN0210.1  	K-----ASAPIKPSFFGQ---RAPSYERILEEG-AQDIELGG-----SESSRSSYDHVSD
          	      . : :  .:* :    . ****::*:*   :: *..       *.  . ..  .

MG07077.1 	GGSSSSRRILATGGGRS-----SGLATPKLNVE-MFDDN-------NRSVSAIDRSGLVV
NCU08027.1	GFYSDSSDSSSSGGGRSKLGITSGLATPKLNLDGRFADLGCGNGLMTPGGSALDRAGLVV
FG04270.1 	NGSRK---------GRT-----SGLATPSAFAG-RADEL-------TRPPSAMDRAGLVV
AN0210.1  	TDSTAFLPPKRTSSWGQ-----SSPARPHFNHDNSSSATVGLGISAAPGLSSIDRNGLVV
          	           :..        *. * *     .        .       *::** ****

MG07077.1 	RTESRERLVPN---LQMSNAGRRSRAGSPTR---MKSPLVSTFQDD-
NCU08027.1	RTDSRERLGLTPTTTTMSLQGRRSRAGSPTR---LKSPLMSPLQG--
FG04270.1 	RTESRERLSPS-----LLSAGRKSRNGSPTR---AKSPFMTALQEDE
AN0210.1  	RTESRDHLAPIA--LGPTTNGRRSRAPSPSRSHSHKSPSATPLHD--
          	**:**::*   .        **:**  **:*: : ***  :.::
```
