# Supplementary material for: Patterns of Intron Gain and Loss in Fungi
Source: PLoS Biol. 2004 Nov 30;2(12):e422. doi: 10.1371/journal.pbio.0020422 (PMC532390; doi:10.1371/journal.pbio.0020422)
Supplement: Table S1 — Also available at http://genes.mit.edu/NielsenEtAl/. (4.3 MB ZIP). [file pbio.0020422.st001.zip › NielsenEtAl/html/1155.html]

AN5811.1.NCU08550.1.MG09114.1.FG06524.1


```
 CLUSTAL W (1.82) Multiple Sequence Alignments - Introns Inserted


Sequence 1: NCU08550.1	287 aa
Sequence 2: FG06524.1	293 aa
Sequence 3: MG09114.1	285 aa
Sequence 4: AN5811.1	294 aa
Alignment Length: 320 aa
Number Identitical Residues: 96 aa
Alignment Score (without introns) 5281


MG09114.1 	--MADNAPADSIVEESPAPSAPAGDVEMTESAS-----------GAVEGDESALPYVGED
NCU08550.1	--MSESFNEDIIED---SPAAPSGDVEMTEGGG----VAEGASAEDAPANKDELPFAEES
FG06524.1 	--MPSGQHEDSVE--------PTGDVEMTETTQNTRETAPENTQDDTKNGEGENENADET
AN5811.1  	MDLIPQVQVSTREE-----RAPAARAQQTPIDT--EATQATIAGEDAGPAEVETQPAEEP
          	 .:      .   .      :*:. .: *             :   .   :     . * 

MG09114.1 	GPEEPVP---~-------ARVPFVD-----YLSSPVVTLMVGSADNSDTQTILTAHQALL
NCU08550.1	ADSPPPP---~--------RVTFLQ-----YLSSPIVTLIVGTGDQE---TVLTAHQSLL
FG06524.1 	ALELAEPEV-~-----AKLRIAFAN-----YLMSPIVTLLIGSGDQ----SILSAHQGLL
AN5811.1  	QLPKPIPFPG2LARKDQRLRLLLIVTSGDRFLTSPIVELIVGSNDKR---TAMTAHQDLL
          	    . *  .  : ..   *: :  ::.. :* **:* *::*: *:    : ::*** **

MG09114.1 	TQSPYFATLCAEF-EDGT0D~RHIDLSSEDVDAVGCFLEYLYTGDYFPRKIPGQRQLDQD
NCU08550.1	VQSPWFAEACADFTDDGS0P~RQIELPNDDIDAMGCFLEFLYTGDYFPKKVPGQRALEKD
FG06524.1 	TQSPYFKDICDTFVEDGS0P~RQIELPEYDIDTVGCFLEYLYTGEYFPKKLPGQRVLESD
AN5811.1  	LESSLLSDHVKAF--DDG~P0RHIELPDDDVEAFGYFLQYLYTRDYS----PSETGADEG
          	 :*. :      *  *.    *:*:*.. *:::.* **::*** :*     *.:   :..

MG09114.1 	PSLPKVDDSGDQLLKHARVYTLAEKFQMPALRHLASSKIHCVNSTAKGEIAYARYVYQYI
NCU08550.1	PSIPEVDLTGEQLLKHARVYTIAEKFGLTNLKNLASSKIHCVNSTAKGEIAYARYVYEFT
FG06524.1 	PAIPAIDDSGDQLLKHARIYTLAEKFGVEGLKTLSSSKIHCVNSTAKGEIAYARYVYAFT
AN5811.1  	-----ADNSGDRLLKHARVYTLAEKLGISTLKSLAHSKIHRINSTSLGELEYARYVYANT
          	      * :*::******:**:***: :  *: *: **** :***: **: ******   

MG09114.1 	PRDDSHIRAPIVTFWAQRSHTLRAEAEDEFRSLCLEFPQFGYDVLT1RVLDEKLKREQ--
NCU08550.1	SKDDTTIRAPVANFWATRSHTLRAEAEDEFRNLCLEFPQFGYDVLT1RVLDEKLKRER--
FG06524.1 	NNDDAIIRAPVASFWATRSHTLRAEAEDEFKALCLEHPQFGYDVLT1RVLDYKLKRER--
AN5811.1  	KSDDVTIRKPVSNFWGMRGHILRHESEEEFRQLCLDVPQFCFDVLS~VVLDQREKRAQDA
          	  **  ** *: .**. *.* ** *:*:**: ***: *** :***:  *** : ** :.:

MG09114.1 	VSKMHPSTASGRKRPRHSNV----
NCU08550.1	NEKMHPGTGSARKRPRHSSQA---
FG06524.1 	NDKMHPATSSARKRSRHSSGSRAE
AN5811.1  	AETEFAVRGSGRKRLRSGL-----
          	 .. ..  .*.*** * .
```
