# Supplementary material for: Patterns of Intron Gain and Loss in Fungi
Source: PLoS Biol. 2004 Nov 30;2(12):e422. doi: 10.1371/journal.pbio.0020422 (PMC532390; doi:10.1371/journal.pbio.0020422)
Supplement: Table S1 — Also available at http://genes.mit.edu/NielsenEtAl/. (4.3 MB ZIP). [file pbio.0020422.st001.zip › NielsenEtAl/html/1156.html]

AN4399.1.NCU04302.1.MG00970.1.FG09935.1


```
 CLUSTAL W (1.82) Multiple Sequence Alignments - Introns Inserted


Sequence 1: NCU04302.1	157 aa
Sequence 2: MG00970.1	210 aa
Sequence 3: FG09935.1	158 aa
Sequence 4: AN4399.1	165 aa
Alignment Length: 210 aa
Number Identitical Residues: 104 aa
Alignment Score (without introns) 5160


MG00970.1 	MSLSQNRL~QEER~LVLLLSCPLVNAALLTFPCNPISKQWRKDHPFGFYAKPQRTENGTL
NCU04302.1	MALCQNRL~QEER2-----------------------KQWRKDHPFGFYARPQKNQQGVL
FG09935.1 	MALCQNRL~QEES2----------------------RKQWRRDHPFGFYAKPQRTKEGVL
AN4399.1  	MTHHEMDI1SRVE~LTG---------------TLAHRKQWRKDHPFAFYAKPHRTAQGVL
          	*:  :  : ..                       .  ****:****.***:*::. :*.*

MG00970.1 	DLKVWECGIPGKEKTIWEGGLFKMTITFPEE1YPTKPPKC~MLKMTELMVLLLLLLLVTP
NCU04302.1	DLKIWECGIPGKEKTIWEGGLFKLTVTFPDE1YPTKPPKC1-------------------
FG09935.1 	DVKNWECGIPGKDSTIWSGGLFKLTIAFPDE1YPTKPPKC1-------------------
AN4399.1  	DMKRWECGIPGKKGTIWEGGLFKLDVTFPDE1YPTKPPKC1-------------------
          	*:* ********. ***.*****: ::**:* ********                    

MG00970.1 	DIHTNALDNLGKFVPPLFHPNVYPSGTVCLSILNEEEAWKPSITVKQILIGVQDLLNDPN
NCU04302.1	-----------KFVPPLFHPNVYPSGTVCLSILNEEEAWKPAITMKQILLGIQDLLNDPN
FG09935.1 	-----------KFVPPLFHPNVYPSGTVCLSILNEDEAWKPAITVKQILLGIQDLLNDPN
AN4399.1  	-----------KFVPALFHPNVYPSGTVCLSILNEDEAWKPAITIKQILLGIQDLLDDPN
          	           ****.*******************:*****:**:****:*:****:***

MG00970.1 	PESPAQADAYNLFKKDKVEYEKRIKRVVRENPAP
NCU04302.1	PESPAQAEAYNLFKKDRQEYERRIKRVVRENAAP
FG09935.1 	PESPAQAEAYNLFKRDRAEYEKRVRRIVRENPTP
AN4399.1  	PESPAQAEAYNMYKKDRAAYEKRVKQVVKENPAL
          	*******:***::*:*:  **:*::::*:**.:
```
