# Supplementary material for: Patterns of Intron Gain and Loss in Fungi
Source: PLoS Biol. 2004 Nov 30;2(12):e422. doi: 10.1371/journal.pbio.0020422 (PMC532390; doi:10.1371/journal.pbio.0020422)
Supplement: Table S1 — Also available at http://genes.mit.edu/NielsenEtAl/. (4.3 MB ZIP). [file pbio.0020422.st001.zip › NielsenEtAl/html/1157.html]

AN6300.1.NCU06769.1.MG03202.1.FG00329.1


```
 CLUSTAL W (1.82) Multiple Sequence Alignments - Introns Inserted


Sequence 1: NCU06769.1	352 aa
Sequence 2: FG00329.1	354 aa
Sequence 3: MG03202.1	352 aa
Sequence 4: AN6300.1	352 aa
Alignment Length: 354 aa
Number Identitical Residues: 268 aa
Alignment Score (without introns) 11127


MG03202.1 	MALIVDKHRPRSLDSLTYHEELSERLRSL0AQSGDFPHLLIYGPSGAGKKTRIVATLKEL
NCU06769.1	MALIVDKHRPRSLDALTYHTELSERLRSL0AQSGDFPHLLVYGPSGAGKKTRIVATLKEL
FG00329.1 	MALIVDKHRPRSLDALTYHDELSERLRSL0AQNGDVPHLLVYGPSGAGKKTRIVATLKEL
AN6300.1  	MALLVDKHRPRSLDTLSYHHELSARLRSL0AQSGDFPHLLMYGPSGAGKKTRTIATLKEL
          	***:**********:*:** *** ***** **.**.****:*********** :******

MG03202.1 	YGPGVEKIKIDARVFQTSSNRKLEFNIVASIYHLEITPSDVGNYDRVVIQDLLKEVAQTQ
NCU06769.1	YGPGVEKIKIDARVFQTSSNRKLEFNIVASVYHLEITPSDVGNYDRVVVQDLLKEVAQTQ
FG00329.1 	YGPGVEKIKIDARVFQTSSNRKLEFNIVASIYHLEITPSDVGNYDRVVVQDLLKEVAQTQ
AN6300.1  	YGPGVEKIKIDNRVFQTTSNRKLEFNIVSSVYHLEITPSDVGTYDRVVVQELLKEIAQTQ
          	*********** *****:**********:*:***********.*****:*:****:****

MG03202.1 	QVDQGARQRFKVVVINEADHLSRDAQAALRRTMEKYSPNLRLILLANSTANIIAPIRSRT
NCU06769.1	QVDLSAKQRFKVVVINEADHLTRDAQAALRRTMEKYSPNLRLILLANSTANIIAPIRSRC
FG00329.1 	QVDQSARQKFKVVVINEADHLTRDAQAALRRTMEKYSPNLRLILLANSTANIIAPIRSRT
AN6300.1  	QVDLSAKQRFKVVVINEADHLTRDAQAALRRTMEKYSPNMRLILLANSTSNIIAPIRSRT
          	*** .*:*:************:*****************:*********:********* 

MG03202.1 	LLVRVAAPSEDEICTVLAASAKKEGWTVSTQLHERIAKESGRNLRRALLMLETVHAQN2E
NCU06769.1	LLVRVAAPTHKEICDVLASSAKKEGWPIVKGLHQRIAEESGRNLRRALLMYEAVYAQN2E
FG00329.1 	LLVRVAAPTHEEICNVLAVSAKKENWPVVQGLHQRIAEESGRNLRRALLMYEAVHAQN2D
AN6300.1  	LLVRVAAPSEEDICTILSTSAKREGWNEAPELNKRIAKESGRNLRRALLMFEAIYAQS2E
          	********:..:** :*: ***:*.*     *::***:************ *:::**. :

MG03202.1 	KVEDNTPIPPPDWEALIGQIAKEIMEEHTPARILQVRAKLYDLLTHCIPPTTILK~TLTF
NCU06769.1	KVTDSTPIPPPDWEALIGQIAKEIMEEHTPARILQVRAKLYDLLTHCIPATIILK~TLTF
FG00329.1 	KVTDSTPIPPADWEALIGQIAQEIYAEHTPARILEVRSKLYDLLTHCIPPTTILK~TLAF
AN6300.1  	KVSDDTPIPPPDWEALISVIAEEILAERSPARLLQVRARLYDLLTHCIPATTILK0TLTF
          	** *.*****.******. **:**  *::***:*:**::**********.* *** **:*

MG03202.1 	KLVPLIDDDLKVEVIKWSAFYEHRIKMGT~KVIFHLEAFVAKFMRILEM~YLMGMDM--
NCU06769.1	KLIPLIDDALKADVIYWSAFYEHRIRTGT~KVIFHLEAFVAKFMRIFEM~YLMSMDL--
FG00329.1 	KLIALVDDGLKGEVIQWAAFYEHRVKTGT~KVIFHLEAFVAKFMRIVEM~YLMSMDMDM
AN6300.1  	KLIAKIDDTLKPEVIKWSAFYEHRITQGS0KVIFHLEAFVAKFMRIYES2YLMGMDF--
          	**:. :** ** :** *:******:  *: **************** *  ***.**:
```
