# Supplementary material for: Patterns of Intron Gain and Loss in Fungi
Source: PLoS Biol. 2004 Nov 30;2(12):e422. doi: 10.1371/journal.pbio.0020422 (PMC532390; doi:10.1371/journal.pbio.0020422)
Supplement: Table S1 — Also available at http://genes.mit.edu/NielsenEtAl/. (4.3 MB ZIP). [file pbio.0020422.st001.zip › NielsenEtAl/html/1158.html]

AN0808.1.NCU02104.1.MG04651.1.FG00915.1


```
 CLUSTAL W (1.82) Multiple Sequence Alignments - Introns Inserted


Sequence 1: MG04651.1	468 aa
Sequence 2: FG00915.1	464 aa
Sequence 3: NCU02104.1	473 aa
Sequence 4: AN0808.1	477 aa
Alignment Length: 484 aa
Number Identitical Residues: 208 aa
Alignment Score (without introns) 11437


MG04651.1 	MNLLLSDDYLL-QDYPENITNTIR~SGHATCLRFNRKGDYLASGR~VDGAVVVWDLDTMG
NCU02104.1	MNLLLAEDYLL-QDYPEHITNTIR~SGHSTCVRFNRTGDFLASGR~VDGTVVIWDLETMG
FG00915.1 	MNLLLSDDYLL-QDYPENITNTIR~SGHATTLRFNRQGDYLASGR~VDGTVVVWDLDTMG
AN0808.1  	MNLSLVDPFVLAQDYPDTLSEKLR1SGHATCLRFNRKGDYLASGR0VDGTVVIFDVETNG
          	*** * : ::*:****: :::.:* ***:* :**** **:***** ***:**::*::* *

MG04651.1 	VARKLRGHSKSVTSLS~WSTCGRYLLSACQGWKAIIWDLRNGARHREVRFRAPVYIAEFN
NCU02104.1	VARKLRGHSKNITSLS~WSRCGRYLLSACQGWKAILWDLQDGSKYCEVRFRAPVYGAELH
FG00915.1 	VAIKLRGHNKSITFLS~WSRCGQYLLTTCQGWKAILWDLQDGKRLREVRFRAPVYMAELH
AN0808.1  	VARKLKGHIRQIQSLS2WSRDGRYLLSSSQDWKCILWDLKDGSRVRTVRFEAPVYIAELH
          	** **:** :.:  ** **  *:***::.*.**.*:***::* :   ***.**** **::

MG04651.1 	PRNP2LQFVASIFEDQPVVVDATN------PIDVKHILPCKPKRPA-TDDSALK----DK
NCU02104.1	PMHH2HQFAAALFEEQPMLVDVKESAQGANPVEVRHILPSVEKRSDDGADTPTK----EK
FG00915.1 	PWNH2LQFVAALFEEQPVLVDITD------PVDVKHILPSAPKRPN-TDDSALR----EK
AN0808.1  	PYNH2LLFVASLFEDQPVLVDISS------PKPVKRILPSAPFRAAPSKDEEIDPAVAAK
          	* :    *.*::**:**::** ..      *  *::***.   *.    *    .: : *

MG04651.1 	WAKEDAKQMTTVAIYDSAGDYILAGTSKGWLNIIDAKTHEVIFSKKTCALVITTMRLTHN
NCU02104.1	HAKEDARHMTTAIVYTATGEHLLAGTTKGRLNIIDATTHKIIYSEKIAGGVITTLRLTES
FG00915.1 	QAKEDAKQMTTCAVWSTTGDHILAGTNKGKLNIVDAKTYEIIYSDKICSGVITTMRMTVS
AN0808.1  	QAAQDAKHSTCVTIFTALGNHIIAGTSKGWINIIETQTCTTIHSTKLCAGVIILLRLASN
          	 * :**:: *   :: : *::::***.** :**::: *   *.* * .. **  :*:: .

MG04651.1 	GKILLLNSQDRIIRTFHMPNLAAE--DFDPDTIQLVDEHKFQDVVNRLQWNHVTFSATGE
NCU02104.1	GKELLVNAQDRTIRTFKVPDLTSA--DLDPDTIQIPLEHKFQDLVNRLSWNHAAFSSTGE
FG00915.1 	GRELLVNSQDRIIRTLRVPNLLAE--NLDLDTLQVPLEHKFQDVVNRLSWNHVTFSATGE
AN0808.1  	GRDLLVNSSDRVIRTILMPDLSQLGIDLEPTNIKLQVEHKFQDVVNRLSWNHVAFSSTGE
          	*: **:*:.** ***: :*:*   . :::  .:::  ******:****.***.:**:***

MG04651.1 	YVAASTYNNHELYIWERNHGSLVRMLEGTKEEQGTIEWHPHRAMLAACGLETGRINIWSV
NCU02104.1	YVAASTFNNHELYIWERGHGSLVRMLEGPKEEQGVIEWHPHKPLLAACGLETGRINIWSV
FG00915.1 	YVAASTYNNHELYVWERNHGSLVCMLKDPKEEQGVIEWHPTRALLAACGLETGRIYIWSV
AN0808.1  	FVTASTFMNPDIYVWERSHGSLVKILEGPREELGVVEWHPTRPFVVACGLESGCIYTWSI
          	:*:***: * ::*:***.***** :*:..:** *.:**** :.::.*****:* *  **:

MG04651.1 	TSPQRWSALAPDFVEVEENVQYVEREDEFDILEQEEIAKRRLDLEDDDVEVLAMHANDGY
NCU02104.1	TSPQRWSALAPDFVEVEENVEYIEKEDEFDIHPHEEIQKRRLDAEDEDVDVLGGGGAGGG
FG00915.1 	VSPQKWSALAPDFAEVEENVEYIEREDEFDIYAQEEIHRRRLDAEDEEVDVLTVDQSKTL
AN0808.1  	VTPQKWSALAPDFGEVEENVIYVEREDEFDIHPAEEIHQRRLDAEDEEPDVLTIEPSKSG
          	.:**:******** ****** *:*:******   *** :**** **:: :**        

MG04651.1 	TGDDTTFQVPILYNLGESDSEEEFVAVSTGTMRRRSPGD--GEGDGSVDNRATAKKATTG
NCU02104.1	D--VAPFRMPVLFNLGESDSEEEFVNVGLGTLRRKSPDDQ-DDGAPASEGRPPAKKTVTQ
FG00915.1 	D-EDESFRMPILFNLGESDSEDEFIAVSTGTMRRRSPGE--GQSDLEEKPPVVKKSAASR
AN0808.1  	D-DIESFRMPVLLDISDSESEEDIVAVGPGTMRRRSPGTGRDKSNANGDGEKDGRNGTTG
          	  .  .*::*:* ::.:*:**:::: *. **:**:**.   ...    .     :. .: 

MG04651.1 	KGRSRKKG
NCU02104.1	RTRTRKR-
FG00915.1 	RGRKR---
AN0808.1  	RGAKGRRR
          	:  .
```
