# Supplementary material for: Patterns of Intron Gain and Loss in Fungi
Source: PLoS Biol. 2004 Nov 30;2(12):e422. doi: 10.1371/journal.pbio.0020422 (PMC532390; doi:10.1371/journal.pbio.0020422)
Supplement: Table S1 — Also available at http://genes.mit.edu/NielsenEtAl/. (4.3 MB ZIP). [file pbio.0020422.st001.zip › NielsenEtAl/html/1159.html]

AN3939.1.NCU02498.1.MG07731.1.FG00359.1


```
 CLUSTAL W (1.82) Multiple Sequence Alignments - Introns Inserted


Sequence 1: NCU02498.1	838 aa
Sequence 2: FG00359.1	830 aa
Sequence 3: MG07731.1	830 aa
Sequence 4: AN3939.1	828 aa
Alignment Length: 865 aa
Number Identitical Residues: 252 aa
Alignment Score (without introns) 15309


MG07731.1 	-------MQRQNKIRPPKR0SLR~HETD----EFDQQWDVLREALTDIHN-----RN~SS
NCU02498.1	------MASRTNKIRPIRK0PIT~NRDQ---SEFEPCWALLRDAMTDIHL-----QN~AG
FG00359.1 	MISGRGGAGTRGRIRPPRR~IVR0ATESGEGSDFEVCWKMLREALDDIHR-----KN~CS
AN3939.1  	------MLSSLTAAMTAEF~APD~LSSS---SCFPPRPSNLLDSPSDSTTSASGARR1NS
          	               . .         .   : *      * ::  *   :::.::.  .

MG07731.1 	R~LLFEHLYRASYKIVLKKQGERLYTLVQEFEGKWFAEQVIPQLQAMIAPN--LINVAVE
NCU02498.1	R~LSFEQLYRASYKIVLRKKGALLYERVRDFEQEWFRDHIMPNIAALITKNLINISLLQH
FG00359.1 	K~LSFEELYRAAYKIVLKKKGELLYEKVKGFEEQWFNDHVIPEIKELFSK-----SLLDS
AN3939.1  	Q1LSFEQLYRNAYSIVKIQRAEDLYERTKELEKEWLCGEVQKRVVAAITPRLLLAKEAVD
          	: * **.*** :*.**  ::.  **  .: :* :*:  .:  .:   ::      .    

MG07731.1 	AG---TSAHERREMGDTFMKGLKEAWENHRMSMNMVADILMYL~DKGFL-KESRGTSIFV
NCU02498.1	PG---SSSHERREMGEKFLRGIRDSWTDHNRSMNMIADVLMYL~DRVYT-LETKQPSLFA
FG00359.1 	GS---RSIHEKRQTGERFLKGLRDKWEDHNMSMNMTADILMYL~DRGYTQLEAQRIPIFA
AN3939.1  	MQDQFTEATERRETGERFLSALSEVWEDHQICMKMITDVLMYM0DRVVA---LRKVSIYA
          	  ..  .  *:*: *: *: .: : * :*. .*:* :*:***: *:       :  .::.

MG07731.1 	TTIGLFRDHLVNPN---TVVGHDRTFSLFDILSTVILDHIDMEREGDVINRSLIHSCVKM
NCU02498.1	VTIGLFRNNVLRSHIGAAAEDIEQDFVVFDILCAVILDLINMERDGDIINRNLVRKITAM
FG00359.1 	TTIALFREHILRS-------SLNTNHKVIDVLISVILEQINMEREGDIIDRNLIRSCTRM
AN3939.1  	AAMALFRDHVLRSP-----VSSSNNAVVADVLKSTVLFMIQLERSGHMIDRALIHSCIKM
          	.::.***::::..       . .    : *:* :.:*  *::**.*.:*:* *::.   *

MG07731.1 	LEDLYETDEEMDADRLYLVRFEPHLLEASRTFYRSEALKLLRNGDASIWIRQTHRRLLEE
NCU02498.1	LESLYETDDEIENHRLYLTLFEPRYLEASTEFYRKECEKLVQEANCSTWLRHAQRRLNEE
FG00359.1 	LSSLYETEDEKDSDKLYSTVFEPRFLENSKAYYAAECEKLLRESDAGAWLRHTQTRLNEE
AN3939.1  	LEGLYETEAEEESSKLYLTSFEPDYLEASAAFYRAEGKRLLETVDAATFCAVVSARIAEE
          	*..****: * :  :** . ***  ** *  :*  *  :*:.  :.. :   .  *: **

MG07731.1 	EDRCKTTLSTLSIEKMTRAVEAELISAHLNDFLALENNGLRQMLDDDRVEDLAILYQLVA
NCU02498.1	RERCGTTLSIMTTDKIASVVEKELIEAKLDVFLAMEGSGLKPMIDNDRLDDLSILYQLIS
FG00359.1 	IDRCGTTIELETLPKVTSTIDQELIIKHLGEFLALEGSGLKWMIDNDKVEELSILYRLVS
AN3939.1  	KERCQYTISLLSESKIKEIVDNELIRNNLAEVVNFEGTGVRVMLDHDRIDNLSKIYALSA
          	 :**  *:.  :  *:   :: ***  :*  .: :*..*:: *:*.*::::*: :* * :

MG07731.1 	RVDPSKDLLKKGVLNRILALGAEIEKNLSTIDFSVA---QGDAAEN-PAAEKPKSQALSQ
NCU02498.1	RVDSTKSALKVILQRRVRELGLEIEKALKNTDFSVAGAAAGDGEDAGEAAEKAKPQTLNP
FG00359.1 	RVDSTKTSLREILQRRVVELGLDIEKVLKNTDFSTG---QGDGEEG----EGDKAKTLNP
AN3939.1  	RVDPKKTHLTAAVQKRIVEMGNEIN----NASFALAQAPAQPKSTGTDAGQKKEKEKEKP
          	***..*  *   : .*:  :* :*:    . .*: . :.         :.:  : :  . 

MG07731.1 	QAQQTAAAIKWVHDVLDLRAKFDVIWEKSFAQDPGLQTTMTKGFSDFIHQFGRSSEFVSL
NCU02498.1	AQQQTAAAIKWVDDVLQLKDKFDRILSDCFCDDLLLQSAITRSFSDFINSFNRSSEYVSL
FG00359.1 	AAQQTAAAIKWVDDVLRLKDKFDNLWTRCFQDDLIIQSALTKSFSDFINMFNRSSEYVSL
AN3939.1  	VNQVTSSAIKWVDDILALKKKFDGIWKDAFGSDQVLQSAITASFSSFLNSTPRSSEFLSL
          	  * *::*****.*:* *: *** :   .* .*  :*:::* .**.*::   ****::**

MG07731.1 	YIDENLKRGIRGKSDLEVTAILDRSIVMIRYLKDKDLFERYYQKHLGRRLLHSRASSEEA
NCU02498.1	FIDDNLKRGIKTKTEAEVDAVLDKAIVLLRYLTDRDMFERYYQKHLAKRLLHGKSEIH-T
FG00359.1 	FIDDNLKRGIKGKTEAEVDVVLEKAIVLIRYLQDRDLFQTYYQRHLARRLLHGKSESHDV
AN3939.1  	FFDENLKKGVKGKTDNEVDALLENGITLLRYIKDKDRFEAYYKKHLSRRLLMKRSMSMDA
          	::*:***:*:: *:: ** .:*:..*.::**: *:* *: **::**.:***  ::   ..

MG07731.1 	EKQLITMMQLELGKHFTSKFEGMFKDITISEELSTKYGEHIRSLGDVDVHHKPIDLAISV
NCU02498.1	EKEMVSRMKSEMGNHFTSKFEGMFKDMELSKDLTDNYRDHIASLGDAD--YKMVDLNINV
FG00359.1 	EKQIISRMKQELGQQFTSKFEGMFRDLVTSTELTTGYRDHIRSVGDG---TKTIDLNINV
AN3939.1  	ERQMISKMKMEVGNQFTQRLEAMFRDMTISEDLTASYKQFVREQGDPD--KKRFELDINV
          	*::::: *: *:*::**.::*.**:*:  * :*:  * :.: . ** .   * .:* *.*

MG07731.1 	LTSNSWPPDVMGRPAQVGRGDGPPAVDCNYPPEIKRLQDSFFKFYLKDRSGRVLTWIGSA
NCU02498.1	LTTNNWPPEVMGGGTSKGEG---AKLDCFYPPEIKRLQESFYKYYLKDRSGRVLTWVSSA
FG00359.1 	LTTNYWPPEVMGRTAQIGDG---SRVTCTYPPELDRLQTSFEQFYLTNRNGRKLTWIGTT
AN3939.1  	LTSTMWPMEIMSSSR---DGQ--VELPCIIPKEVETVKQSFEKFYLDKHSGRKLSWQPSM
          	**:. ** ::*.       *.    : *  * *:. :: ** ::** .:.** *:*  : 

MG07731.1 	GSADIKCVFPPVKGM-SGPLSRERRYELNVSTYGMVVLMLFNSLEDGETLSFEDIQAETS
NCU02498.1	GNADIKCVFPKVPGKETGPLSKERRYELNVSTYGMIVLMLFNDLVDGESLSFDEIQAKTN
FG00359.1 	GSSDIKCTFPAIAGK-SGPLSRERRYEINVPTFAMVVMLLFNDLEDDQSLTFEEIQAKTN
AN3939.1  	GTADIKATFHRSNGK-------VQRHELNVSTYAMFILLLFNDIPIGESLTFEEIQARTR
          	*.:***..*    *         :*:*:**.*:.*.:::***.:  .::*:*::***.* 

MG07731.1 	IPPKDLSRALASLSINPKARVLLKDPATKTIRPGDKFSFNAGFVSKAIKIKAPVINS-QS
NCU02498.1	IPAPELMRTLASLSSVPKCRVLLKEPATKNVKNTDKFSYNAQFVSKAIRIKAPVISS-IS
FG00359.1 	ISNQDLMRTLTAIAVAPKSRVLLKDPANKSVKPGDKFTFNASFQSKTIRIKAPIINA-VS
AN3939.1  	IPDNDLIRNLQSLAVAPKTRVLKKVPMSRDVKPTDKFYFNNDFQSPFMKVRIGVVSGGAN
          	*.  :* * * :::  ** *** * * .: ::  *** :*  * *  ::::  ::... .

MG07731.1 	KVEGDEERQRTEDKNDETRRHMIDAAIVRIMK~SRKELAHNALLAEVIGQLVSRFQPDVA
NCU02498.1	KVEGDEERKETERKNDQTRAHVIDAAVVRIMK2QRKLLAHTKLVNEVISQLMGRFKPDVP
FG00359.1 	KVEDTTERKTTEEKNNQTRAHIVDAAIVRIMK2SRKELSHSQLTSEVLSQLSGRFKPEVS
AN3939.1  	KVESQDQRKETEEKMNNERGGSIEAAIVRIMK2QRKTLIHSNLISEVLSQLSARFVPDVN
          	***.  :*: ** * :: *   ::**:***** .** * *. *  **:.** .** *:* 

MG07731.1 	MIKTRIEDLIAREYLER----LDDSGYKYMA
NCU02498.1	LIKKRIEDLLAREYLER--VEGDSSTYRYLA
FG00359.1 	LIKKRIEDLIAREYLERPDEDGAPSLYRYVA
AN3939.1  	MVKRRIESLIDREYLER--VSEDPPTYGYVA
          	::* ***.*: ******   .   . * *:*
```
