# Supplementary material for: Patterns of Intron Gain and Loss in Fungi
Source: PLoS Biol. 2004 Nov 30;2(12):e422. doi: 10.1371/journal.pbio.0020422 (PMC532390; doi:10.1371/journal.pbio.0020422)
Supplement: Table S1 — Also available at http://genes.mit.edu/NielsenEtAl/. (4.3 MB ZIP). [file pbio.0020422.st001.zip › NielsenEtAl/html/116.html]

AN6907.1.NCU06278.1.MG05265.1.FG02570.1


```
 CLUSTAL W (1.82) Multiple Sequence Alignments - Introns Inserted


Sequence 1: NCU06278.1	1083 aa
Sequence 2: MG05265.1	1073 aa
Sequence 3: FG02570.1	1060 aa
Sequence 4: AN6907.1	1043 aa
Alignment Length: 1299 aa
Number Identitical Residues: 453 aa
Alignment Score (without introns) 24657


MG05265.1 	-----------~------------------------------------------------
NCU06278.1	-----------~------------------------------------------------
FG02570.1 	-----------~------------------------------------------------
AN6907.1  	MSFNAKNLAYD1SKEPAFLQRLRSQFGNSSGGLERPSLRPRRVHDDKDDDAPTYVDAESN
          	 : .:.. : . :...:  .   :. ..::.. . .:  .    ......:.:  .:.:.

MG05265.1 	------------------------------------------------------------
NCU06278.1	------------------------------------------------------------
FG02570.1 	------------------------------------------------------------
AN6907.1  	EVISKEDYEAMVKCGDSETKQPKDGEEDEDAVANQDGDSQKAETAIMKQNLAEIGGPRKR
          	.  :... .:  ....:.:...........: :.....:..:.::  ... :. ... . 

MG05265.1 	----------------------~-------------------------------------
NCU06278.1	----------------------~-------------------------------------
FG02570.1 	----------------------~-------------------------------------
AN6907.1  	KQAKVVGDEVEGDDVEGVEKEE0FSKSQDFVRSEVINERVVLNVLEVKYFFLSAVPDSEA
          	..:.  ... .... .. ....  :.:..   :.  ..    .  . .    :: ..:.:

MG05265.1 	-----------------------------MPVVSPEKLAQLQQHADNVRN~ICILAHV0D
NCU06278.1	-----------------------------MPVVQPEKLAKLQQNADDVRN0ICILAHV0D
FG02570.1 	-----------------------------MPVVTPDKLASLQRQSSDIRN0ICILAHV~D
AN6907.1  	TLTRQAFLDWKLPRTSSFVAGQTRHLPSKMPVVSVEDLIRLQRKPDDIRN0ICILAHV0D
          	: : .:  . . . :::  :..:   .:.****  :.*  **::..::** ******* *

MG05265.1 	HGKTSLTDALLATNGIISPKLAGKIRYMDSRPDEQARGITMESSAISLLFSMLRRSSPDA
NCU06278.1	HGKTSLTDALLATNGIISPKLAGKIRYLDSRPDEQQRGITMESSAISLYFSMLRRSAPDA
FG02570.1 	HGKTSLTDALLATNGIISPKLAGKIRYLDSRPDEQTRGITMESSAISLYFAMRRKAAADA
AN6907.1  	HGKTSLTDSLIATNGIISPKLAGKIRYLDSRPDEQLRGITMESSAISLFFSMMRRSAPDA
          	********:*:****************:******* ************ *:* *:::.**

MG05265.1 	APVAADYLINLIDSPGHIDFSSEVSTASRLCDGAVVLVDAVEGVCSQTVTVLRQTWTEKL
NCU06278.1	TPEAKEYLINLIDSPGHIDFSSEVSTASRLCDGAVVLVDAVEGVCSQTVTVLRQAWTEKL
FG02570.1 	EPEDKEYLVNLIDSPGHIDFSSEVSTASRLCDGAVVLVDAVEGVCSQTVTVLRQTWTEKL
AN6907.1  	QPEAKEYLINLIDSPGHIDFSSEVSTASRLCDGAVVLVDAVEGVCSQTVTVLRQTWVEQL
          	 *   :**:*********************************************:*.*:*

MG05265.1 	KPLLVINKIDRLVTELKMSPSEANVHLSKILEQVNAVLGSFFQGERMEEDLNWRERIEER
NCU06278.1	KPLLVINKIDRLVTELKMTPGEAYIHLSKILEQVNAVLGSFFQGERMEEDLNWRDRMEER
FG02570.1 	KPLLVINKIDRLVTELKMTPGEAYIHLNKLLEQVNAVLGSFFQGERMEEDLNWRERMEER
AN6907.1  	KPILVINKMDRLITELQMTSAEAYSHLSRLLEQVNAVIGSFYQGERMEEDLQWRERMEER
          	**:*****:***:***:*:..**  **.::*******:***:*********:**:*:***

MG05265.1 	VAAAAAREAQAAGQQDEEAGD-LSFQEKDDEDLYFAPEKNNVIFASAIDGWAFTVRQFAG
NCU06278.1	VAAAAAKEAQIAAGQ-PDSGE-LQFQEKDDEEIYFAPEKNNVIFGSAIDGWAFTVRQFAG
FG02570.1 	VNAATAAKESAIADQVSESGE-IHFEERDDEDIYFAPEKNNVIFSSAIDGWAFTCRQFAA
AN6907.1  	VNAAAQQKQAQDGEPAEGGVDGAQYVERDDEDLYFAPEKNNVIFCSAVDGWAFTVRQFAA
          	* **:  :    .     . :.  : *:***::*********** **:****** ****.

MG05265.1 	LYEKKLGIKRSIMEKVLWGNFYLDPKTKKVLGPKHLKGRALKPMFVQLVLEPIWTVYAAT
NCU06278.1	MYEKKLGIKRGLLEKVLWGNFYMDPKTKKVLGPKHLKGRPLKPIFVQLVLEPIWAVYQAT
FG02570.1 	MYEKKLGIKRGIMEKVLWGNFYLDPKTKKILGPKHLKGRNLKPMFVQLVLEPVWTVYQAT
AN6907.1  	IYEKKLGIKRAILERVLWGDYYLDPKTKRVLGQKHLKGRALKPMFVQLVLDSVWAAYEAT
          	:*********.::*:****::*:*****::** ****** ***:******:.:*:.* **

MG05265.1 	MGKEYNGHS~DAALLEKITKSLNINVPAHILRARDPRLLLTTVFASWLPLSVALLVSVVE
NCU06278.1	VGGDS-GKG~DPALLEKITKSLNLSVPPHILRSRDPKLLLTTVFASWLPLSTALLVSVVE
FG02570.1 	VGGDN-GQG~DRELLEKVTKSLGIKITPHMLKSRDQKLLMNTVFAGWLPLSTALLVSVIE
AN6907.1  	TGGGK-GKG2DPALLEKITKSLGINIPPYILRSRDPRNIMTTLFSQWLPLSTALLVSVIE
          	 *    *:. *  ****:****.:.:..::*::** : ::.*:*: *****.******:*

MG05265.1 	SLPSPKAAQAERLPELLRSCPGPDSIDSKIRDAMVQFKSSGDDPVVAYVSKMVSVPASEL
NCU06278.1	SLPSPKAAQADRLPDLLASVPGADHIDPKVKDAMVSFKKDPSEPMVAYVSKMISVPESEL
FG02570.1 	SLPSPPAAQAARLPEMLEESPGADHIDQTIKDSMISFKHEKSDPVVAYVSKMVSIPESEL
AN6907.1  	YLPSPRTAQSSRLPPMIEESPGSKYVDAAVKDAMIQFKTGPKEPVVAYVSKMVSIPESEL
          	 **** :**: *** :: . **.. :*  ::*:*:.**   .:*:*******:*:* ***

MG05265.1 	PENKRRN-GPLSPEEARDIARKKRAEAAKAQAGASNG----IDDLTSAIGSTSLDDDPIS
NCU06278.1	PENRRR--GPLSPEEARELARKKRAEAIRAQGGADGDP--DMNDLANAFTSTSLTDNAVP
FG02570.1 	PENKRRAGAQMSGEEARELARKKRAEAARAQAAAGEN---GVESMVTSMDAINLDDY-AP
AN6907.1  	SSSKKKT-GTMSADEARELARRKREEIAKMQAEASTNQADDFSRVTSAFERVQIDDENQP
          	...:::  . :* :***::**:** *  : *. *. . :.... :..::   .: *   .

MG05265.1 	PAEPEAEAEAEHLIGFARIYSGSLSVGDSVYVLPPKFSPATPNADPKPQKVTVEALYMLM
NCU06278.1	ELE-EKPVEAEHLIGFARIYSGTLSVGDEIYVLPPKFSPADPHAQPVPKKVTVTALYMLM
FG02570.1 	ELE-EKKVDPEHLIGFARIYSGTLSVGDKIYVIPPKWSPAEPDAEPAPQEVTVTALYMLM
AN6907.1  	AEQ-EQKEDPEHLIGFARLYSGTLSVGDSVYVLPPKFSPENAHASPEPQKVIVTDLYLLM
          	  : *   :.********:***:*****.:**:***:**  ..*.* *::* *  **:**

MG05265.1 	GRSLESLTTVPAGVVFGIKGLEGSGILKSGTLCGQLEGAVNLAGVGSAVGRPIVRVALEP
NCU06278.1	GRNLEALPSVPAGVVFGIRGLEGSGLLKSGTLCSQLEGSVNLAGIANLAGRPIVRVALEP
FG02570.1 	GRNLEALESVPAGCVFGISGLEG-KILKSGTLCSRREGAVNLAGVT-MLGKPIVRVALEP
AN6907.1  	GRSLEPLQSVPAGVVFGIGGLSG-HILKTGTLCSQLEGGINLAGVS-LDTPPIVRVALEP
          	**.**.* :**** **** **.*  :**:****.: **.:****:      *********

MG05265.1 	ENPADLDKMIAGLKLLVQSDPCAEYEQFASGEHVLLTAGELHLERCLTDLRERFARCEIQ
NCU06278.1	VNPYDLDKMIQGLHLLVQSDPCAEYEHFSSGEHVLSTAGELHLERCLTDLRERFARCEIQ
FG02570.1 	VNPADLDKMIQGLKLLVQSDPCAEYELLSSGEHVLLTAGELHLERCLTDLKERFALCDIQ
AN6907.1  	VNPSDLSKMVTGLRLLEQSDPCALYEVLPNGEHVILTAGELHLERCLKDLRERFAKCEIQ
          	 ** **.**: **:** ****** ** :..****: ***********.**:**** *:**

MG05265.1 	AGAPIVPYRETIVKAEEMRPPANKELGRGLVVAVSSSKQATITLKVSPLPKDVTDFLLKQ
NCU06278.1	AGAPIVPYRETIVRAEEMRPPVNKELGRGVVVGVTSSRQVTVTLRVRPLPAPVTEFLQKN
FG02570.1 	PGAPIVPYRETIIRADEMRPPVNKELGRGAVVATTSSKQVTITIRVLPIPDKVTDFLVKN
AN6907.1  	TGQTIVPYRETIVRAAEMAAPKNPDLGRGGVLAVSASKQLTVRLKVIPLPEAVTDFFTKH
          	.* .********::* ** .* * :**** *:..::*:* *: ::* *:*  **:*: *:

MG05265.1 	SAAIKQLSSDPASNNDSASESGDKVEESQADEAFAG---IEE-------AKTLSPDEFKS
NCU06278.1	SAGIKRLYTDRKAAGVEDGTESPSVEEATLENGTPSDVKIEEDEEVSQAATTLTPEELKK
FG02570.1 	GDAVKKVYDRKAGTGEEG-------EEIVAEADVAAG-------------NTLSVEDFKK
AN6907.1  	VGTIKRLQSQKRIIRAEGENATLSSESTQQDEAADATGEARE-------VSLLSLDDFRK
          	   :*::         .      . *.   :    .  .  .        . *: ::::.

MG05265.1 	QLKEKLEFGK-GREAWKSAAERIVSFGPRRTGPNILVDGTASGILPRIFGDDADKADAPS
NCU06278.1	QLQAQLDGVK-DKEAWKDVIDRIASFGPRRTGANILVDATKDQLFSKAFSADKIRNQAPT
FG02570.1 	QLKEKLEEGK-GKEVWKDCIDKIVAFGPRRTGPNLLIDSTADGIFARAFAPEKAVETAPR
AN6907.1  	ELSKTFEEVKEDKELWKDVVDKIIASVLGGQGP---------------------------
          	:*.  ::  *..:* **.  ::* :      *.                           

MG05265.1 	ADERLKASHLSDKITYGFQLAMAQGPLCHEPVQGVAVVIENVEIADAAQD--SSARDAIG
NCU06278.1	GDEKLHPAHFSDKIMYGFQLATQQGPLCNEPVQGIAVFVEDVLVAQTEDDEASSARDRIG
FG02570.1 	ADESLHPSHLTDKISYAFQLASAQGPLCNEPIQGVAVVVEDITLNLAEDD--SSARDKLG
AN6907.1  	-----------------MSLSTPQ------------------------------------
          	                 :.*:  *                                    

MG05265.1 	RLTGEVIKAVQQGIHKGFLDWSPRLMLAMYSCEIQAST1EVLGRVYDVLTRRRGNVLSEA
NCU06278.1	RLTGEVIKTVQQSIHKGFLDWSPRLMLAMYSCEIQAST1EVLGRVYDVLTRRRGRVQSEA
FG02570.1 	RLTGELIRTFQSSLRAGFLDWSPRLMLAMYSVEIQAST~EVLGRVYDVLTRRRGRVNAEL
AN6907.1  	--------------------------------------~---------------------
          	                                                            

MG05265.1 	MKEGTPFFTIQSLLPAAESFGFADEMRKRTSGAAQPQLIFTGFEILDEDPFWVPFTEDDL
NCU06278.1	MNEGTPFFTIQALLPVAESFGFADDMRKRTSGAAQPQLIFTGYEILDEDPFWVPFTEDDL
FG02570.1 	MKEGTPFFTIQALVPVAESFGFADEMRKRTSGAAQPQLIFAGFEILNEDPFWVPFTEDDL
AN6907.1  	------------------------------------------------------------
          	                                                            

MG05265.1 	EDLGEYGDRENVAKRYMDSVRRRKGLLVEGRNVARDAEKQKNMKR
NCU06278.1	EDLGEFGDRENVAKRYMDSVRRRKGLLVEGRNVATNAEKQKTLKR
FG02570.1 	EDLGEFGDKENVAKRYMDGVRRKKGLLVEGRNVATDAEKQKTLKR
AN6907.1  	---------------------------------------------
          	
```
