# Supplementary material for: Patterns of Intron Gain and Loss in Fungi
Source: PLoS Biol. 2004 Nov 30;2(12):e422. doi: 10.1371/journal.pbio.0020422 (PMC532390; doi:10.1371/journal.pbio.0020422)
Supplement: Table S1 — Also available at http://genes.mit.edu/NielsenEtAl/. (4.3 MB ZIP). [file pbio.0020422.st001.zip › NielsenEtAl/html/1160.html]

AN1341.1.NCU01822.1.MG02524.1.FG01552.1


```
 CLUSTAL W (1.82) Multiple Sequence Alignments - Introns Inserted


Sequence 1: NCU01822.1	239 aa
Sequence 2: MG02524.1	190 aa
Sequence 3: FG01552.1	202 aa
Sequence 4: AN1341.1	200 aa
Alignment Length: 242 aa
Number Identitical Residues: 104 aa
Alignment Score (without introns) 5275


MG02524.1 	-MAFLILVIGDLHIPDRAL~DIPPK0FKKLLAPGKIGQTLCLGNLTDRTTYEYLRSVAPD
NCU01822.1	-MAFLILVIGDLHIPDRAL~DIPAK0FKKLLAPGKISQTLCLGNLTDRSTYEYLRTIAPD
FG01552.1 	-MAFLILVIGDLHIPDRAL~DIPAK0FKKLLSPGKISQTLCLGNLTDKHTYEYLRSVSPD
AN1341.1  	MTSRLVLVIGDLFIPDRAP0DLPAK0FRKLLTPGKIGQILCLGNLTDRSTFEFLRQVAPD
          	  : *:******.*****  *:*.* *:***:****.* ********: *:*:** ::**

MG02524.1 	LKIVKGRLDVEATSLPLSQVVTHGSIRIGFLEGFTLVSN-EPDLLLAEANKLDVDVLCWG
NCU01822.1	LKIVRGRMDVEATSLPLTSVVTHGNLRIGFLEGFTLVSN-EPDLLLAEANRLDVDVLCWG
FG01552.1 	LKIVKGRYDVEATSLPLTQVVTHGSLRIGFLEGFTLVSN-EPDLLLAEANKLDVDVLCWG
AN1341.1  	LQLVKGDFDVDSPNLPLSKVVTHGSLRIGFTHGHTIIPQGDADALLIAARQMDVDILLWG
          	*::*:*  **::..***:.*****.:**** .*.*::.:.:.* **  *.::***:* **

MG02524.1 	GTHRFECFEYMDKFFINPGSATGAFTT---------------------GWGTE-EDIVPS
NCU01822.1	GTHKFECFEYMDKFFVNPGSATGAFTTTAASWAVNLGSNGEGQKEQDKGFDED-EEVVPS
FG01552.1 	GTHRFDAFEYMDKFFVNPGSATGAFME---------------------GFSQEADEPTPS
AN1341.1  	GTHRFEAFELEGRFFVNPGSATGALST---------------------GYWPEGEEPTPS
          	***:*:.**  .:**:********:                       *:  :.:: .**

MG02524.1 	FCLMD0------------LRKDENGVENVAVEKVTYTKVVEPP--------TTASAS---
NCU01822.1	FCLMD~VQGISLTLYVYQLRKDEKGVENVAVEKVTYTKPVEPTGAGGAAAGAGAAASGAA
FG01552.1 	FCLMD0VQGISLTLYVYQLRKDDKGNENVAVEKVTYTKPVEPS---------GGASS---
AN1341.1  	FCLMD0IQGDVLVLYVYQLKTDSNGVETVAVEKVSYRK-------------NSVLSS---
          	*****  ..        .*:.*.:* *.******:* *                 :*   

MG02524.1 	-----
NCU01822.1	SVSSS
FG01552.1 	-----
AN1341.1  	-----
          	
```
