# Supplementary material for: Patterns of Intron Gain and Loss in Fungi
Source: PLoS Biol. 2004 Nov 30;2(12):e422. doi: 10.1371/journal.pbio.0020422 (PMC532390; doi:10.1371/journal.pbio.0020422)
Supplement: Table S1 — Also available at http://genes.mit.edu/NielsenEtAl/. (4.3 MB ZIP). [file pbio.0020422.st001.zip › NielsenEtAl/html/1161.html]

AN8119.1.NCU01388.1.MG08182.1.FG07370.1


```
 CLUSTAL W (1.82) Multiple Sequence Alignments - Introns Inserted


Sequence 1: MG08182.1	391 aa
Sequence 2: FG07370.1	394 aa
Sequence 3: NCU01388.1	393 aa
Sequence 4: AN8119.1	395 aa
Alignment Length: 395 aa
Number Identitical Residues: 213 aa
Alignment Score (without introns) 11709


MG08182.1 	--MARPVRVLATVAIFMWCVFLYMIFRPSSPLL-VADEFSNFQRDPMHDP1TGEPEGILR
NCU01388.1	MAIARPVRALALAAALVWCFFIWQLLSPS-GTAMTKDKYLSFERDPNLDP1TGEPEGKLI
FG07370.1 	MTVARPVRALIAGGCIIWCFFMWQIFAPSWGLSGPGDRYSNFERDPMLDP1TDEPEGKLH
AN8119.1  	MAVARPIRMLSAACVVLVIFLVFQMKRSPSYVGMGPGEYNGMTADPLNDP1TGEPDGYLW
          	 ::***:* *     .:  .::: :  ..       ..: .:  **  ** *.**:* * 

MG08182.1 	RVSP-EYAPDANPTERINATLLALVRNEELDGMLQAMGDLERTWNSKFNYPWTFFNDVPF
NCU01388.1	RASE-DYAPGAKNSARINATLLALVRNEELEGMLQAMRDLERTWNHKFNYPWTFFNDVPF
FG07370.1 	RTSP-RYAHDAQKTERIDATLLALVRNEEVDAMVMSMRDLERTWNSKFNYPWTFFNDKPF
AN8119.1  	RADEHDYAPDSTNSARTNAAIISLVRNEELNELLPSMRDLERTWNHKFNYPWIFFNDVPF
          	*..   ** .:  : * :*::::******:: :: :* ******* ****** **** **

MG08182.1 	SKEFKQKTQAMTKAKCNYE1IIPKEHWDMPSWINKDIYDESVKILKENKIQYADKISYHQ
NCU01388.1	SDEFKKRTRAVTKAEVRYE1LIPKEHWEMPSWINQDLYHESVKILEEKKIQYADKISYHQ
FG07370.1 	TEEFKRKTRAATKAKINYE1IIPDEHWKMPSWIDEQIFEESAKILEKNGVQYASKISYHQ
AN8119.1  	TEEFKKRTQAETKAKCQYE1LVPKEHWEVPSFIDMNLFKESAALLKEKGLQYADKISYHQ
          	:.***::*:* ***: .** ::*.***.:**:*: :::.**. :*::: :***.******

MG08182.1 	MCRWNSGLFYKHPALKDVQYYWRVEPKVHFFCDIDYDVFRFMQDNNKTYGFTINLYDAPE
NCU01388.1	MCRWNSGMFYKHPALANTQYYWRVEPNVHFFCDVDYDVFRYMHDNNKTYGFTINLYDAPA
FG07370.1 	MCRWNSGLFYKHPALKDIRYYWRVEPNVHFFCDVDYDVFRYMHDNNKTYGFTINLYDDPK
AN8119.1  	MCRWNSGMFYKHPALKDYRYYWRVEPKVQFFCDVDYDVFRFMEDRNKTYGFTINLFDAPE
          	*******:******* : :*******:*:****:******:*.*.**********:* * 

MG08182.1 	SIPTLWPETEKFLAEHPQYKHPNNALDWLTDKEKRPEHNRKANGYSTCHFWSNFEVADMN
NCU01388.1	SITTLWPETEKFLAEHPEYVHENSAMNWLTDKTRRPDHTKDANGYSTCHFWSNFEIGDMN
FG07370.1 	TLPSLWPETVKFLAEHPGYIHQNSAVGWVTDDIRRPQSNRKAQGYSTCHFWSNFEIGDME
AN8119.1  	SIPSLWPTTQEFLAANPSYLSDNNMMDWLTDDQLRPDHTRDANGYSTCHFWSNFEIGDME
          	::.:*** * :*** :* *   *. :.*:**.  **: .:.*:************:.**:

MG08182.1 	FWRSKTYEDYFNHLDRAGGFFYERWGDAPVHSIALGLFEDASKIHW2FRDIGYQHIPFFN
NCU01388.1	FWRSKAYEDYFNHLDRAGGFFYERWGDAPVHSIALGLFEDSSKIHW2FRDIGYQHIPFFN
FG07370.1 	FWRSKTYEDYFNHLDRAGGFFYERWGDAPVHSIALGLFEDQSKIHW2FRDIGYQHIPFFN
AN8119.1  	FFRGDKYSAYFDFLDHAGGFFYERWGDAPVHSIGLGLFEDKNKVHW2FRDIGYRHIPYFN
          	*:*.. *. **:.**:*****************.****** .*:** ******:***:**

MG08182.1 	CPNSPKCKGCVAGRFTDGEKWLHREDCRPNWFKMHGMG
NCU01388.1	CPNSPKCKGCVTGRFTDGEAWLNREDCRPNWFKYVGMG
FG07370.1 	CPNSPKCKGCVTGRLTDGEAWLHREDCRPNWFKYVGMG
AN8119.1  	CPNSPKCSACTPGKFYEGASFLAKEDCRPSYFKHVGTH
          	*******..*..*:: :*  :* :*****.:**  *
```
