# Supplementary material for: Patterns of Intron Gain and Loss in Fungi
Source: PLoS Biol. 2004 Nov 30;2(12):e422. doi: 10.1371/journal.pbio.0020422 (PMC532390; doi:10.1371/journal.pbio.0020422)
Supplement: Table S1 — Also available at http://genes.mit.edu/NielsenEtAl/. (4.3 MB ZIP). [file pbio.0020422.st001.zip › NielsenEtAl/html/1162.html]

AN7451.1.NCU00461.1.MG05247.1.FG04117.1


```
 CLUSTAL W (1.82) Multiple Sequence Alignments - Introns Inserted


Sequence 1: NCU00461.1	1050 aa
Sequence 2: MG05247.1	1045 aa
Sequence 3: FG04117.1	1055 aa
Sequence 4: AN7451.1	1096 aa
Alignment Length: 1108 aa
Number Identitical Residues: 587 aa
Alignment Score (without introns) 27665


MG05247.1 	------------------------------------------MASPI--NSTAQKALGTN
NCU00461.1	------------------------------------------MDSPS--APVPAHKLVDR
FG04117.1 	-MSPSQSFP-----------------------------EYSEPTSIM--TAISANPVANV
AN7451.1  	MVSPASSLQSLPDVHVNGNEPNKLPVRSAPKLYGSNDGASSGTGTPIGFQRQPHNKILDS
          	  :.:.:  : ..   ....... .  ::..  .:...  :   :  .    . : :   

MG05247.1 	GDDQ--RHPSPQPTHFAVSKLKGNG---NRTLRSATVGYIAPEFKGKAEQMLEV1KKVIQ
NCU00461.1	LKDQTPRHPSPQPTHVSYPKVNGNG---HRVLRSATVGYVAPVFQGKAEQMKQV1KNIIV
FG04117.1 	KATEASRGPSPQPTHFSVPLQNGNGGNGHRILRSATVGYIAPEFTGKSEQKKTV~KSLIW
AN7451.1  	VAGSNVRMPSPQPTHLAIP---GSP---HRVLSEEDPGYIAAKFEGKEHQMEEV1MDQLE
          	   .  * *******.: .   *.    :* * .   **:*. * ** .*   *  . : 

MG05247.1 	ENGWIPDKVIDEQVAWFYNELGIDDVYFQLERPAVVANHITSLYAAKVAAFSREDKREEI
NCU00461.1	QGGWIPETLVDGQIAWFYNELGIDDVYFQLENPQAVANHITSLYAAKVAAFSREDKREEI
FG04117.1 	AAGYVPEPQIDEQIEWFYENLGIDDVYFELESPDVISSHITSLYAAKVASFAREDKQEEI
AN7451.1  	KKGFIPPEFIVGETEWFYNQLGIDDTYFQTETVEAIVTQILSLYAAKVAAYARDDKKLEI
          	  *::*   :  :  ***::*****.**: *   .: .:* ********:::*:**: **

MG05247.1 	RLDMEASDHAIYIDTSEPGRSAIEGPRYETRLEAKYLDGND-SQRFRVETFRSPGVLGQN
NCU00461.1	RLDMEASDHAIYIDTSEPGMTSFDGPRYEHRLESKYLDGDDTSKRFRVETFRSPGVLGQK
FG04117.1 	RLDMEANDHAIYIDTSVAGRTNIAGPRYEERLEAKYIDHPG-SSKYRVETFRSPALLSPQ
AN7451.1  	RLDKEAEDHAVYIDTSKPGFSSVNGPGYEQRIDKKYIDGSTHDHSYRIETFRSPTPVPGD
          	*** **.***:***** .* : . ** ** *:: **:*    .  :*:******  :  .

MG05247.1 	TNGGGASLRCYFVYQCTFVEP--NADPDETRLEVISDRMFLAKATKNTLQIYQEIIELAV
NCU00461.1	ENSK-AALRCYFVYQCLFVDS--NADPKETRLEVISDRMFLAKATKNTKQIYQDIIQVAV
FG04117.1 	---SKATLRCYFVYQCRFATPPEETDPKETNLELIADHGFLQKATVNTKQIYQDIIELAV
AN7451.1  	---DGQQLRCYFVYKCQFANP--NPGPQETNIDIIGEKRFLQKATPNTKAIYQEIISNAV
          	       *******:* *. .  :..*.**.:::*.:: ** *** **  ***:**. **

MG05247.1 	NRTGPVIEVFDIEDSPEKRLVVAFRSRTARGMFSAISDLYHYYGVTSSRKYVEQFSNGIT
NCU00461.1	SRHGPVIEVFDIEGSEEMRLVVAFRSRTAKGIFSALSDLYHYYGVTSSRKYVEQFSNGIT
FG04117.1 	NRAGPVIEVFDIEGTDEKRMVLAFRSRTAQGLFSALSDLYHYYGVTSTRKYLEQFSNGIT
AN7451.1  	ARAGPVIEMFEIEGSREKRLVIAYRQGSAMGLFSALSDLYHYYRLTSSRKYLENFSNGIT
          	 * *****:*:**.: * *:*:*:*. :* *:***:******* :**:***:*:******

MG05247.1 	VMSIYLRPASNLDG--KYPSIEQSIHQITKETSLLYCIPLNKLHHLFATGELSLQEAIYG
NCU00461.1	VMSIYLRPAANIDG--KHPPLEQSIHQITKEISLLYCLPQNKFHNMFASGELSLQETIYA
FG04117.1 	VMSVYLRPASETTENADQFSWDESIDQISKEVSLLYCLPHNKFHNLFLDGQLSLQESVYA
AN7451.1  	VISLYLRPLKNAEVAAKFPPIEAAVHQIIKEISLLYCIPQNRFQHHFASGRLSLQETIYA
          	*:*:****  :    :.  . : ::.** ** *****:* *:::: *  *.*****::*.

MG05247.1 	HCAWVFVQHFLNRLGSEYATLSDALDPKNNVHAELLSKLKRRLRTETFTPDYILEIIGSY
NCU00461.1	HCVWVFVQHFLNRLGTEYTSLIAALDPKNNSHVEILSKMKKRLRTETFTPDYILEIISSH
FG04117.1 	HSAWVFVQHFLNRLGPEYASLAELLDIKNNAQQALLSNLKRRLRSETFTPDYIYEIIQNY
AN7451.1  	HCAWVFVQQFLNRLGSEYTSLTDLLDSNNSTHAELLAKIKKRLRTETFTSDYIAEIVNKY
          	*..*****:******.**::*   ** :*. :  :*:::*:***:****.*** **: .:

MG05247.1 	PGLVRLLYAAFASVHLNTDAK-EKGTITPTP------GVEVLSDEALKERITREVSNEHE
NCU00461.1	PQLVRALYASFASVHLRVGSDYDRHLIAPTP------VMEVLSDARLKEKITKDVSNEHE
FG04117.1 	PGLVRALYASFANVHLVKDQEDPVKVVSSSL------SVEVLSDDALKDKISKNVNNEHD
AN7451.1  	PDLIHKLYLDFANTHYVQTQGPTEDDFLPTLSYLRLQVDQVLDSRQLKQLVSSTAANEHD
          	* *:: **  **..*           . .: :    .  :**..  **: ::  . ***:

MG05247.1 	EMVMTAFRVFNSAILKTNYFTPTKVALSFRLDASFLPEIEYPTPLYGMFLVITSESRGFH
NCU00461.1	EMVMTAFRVFNNAVLKTNFFTPTKVALSFRLNPSFLPEVEYPKPLYGMFLVITSESRGFH
FG04117.1 	EMVLTAFRVFNNAVLKTNYFTPTKVALSFRLDPSFLPDVEYPKPLYGMFLVISSESRGFH
AN7451.1  	EMVMSAFRVFNASILKTNFFTPTKVALSFRLDPHFLPEHEYPQRLYGMFLVISSEFRGFH
          	***::****** ::****:************:. ***: ***  ********:** ****

MG05247.1 	LRFKDVARGGIRIVKSRSKEAYSINARNLFDENYGLASTQQRKNKDIPEGGSKGVILLDA
NCU00461.1	LRFKDIARGGIRIVKSRSKEAYQINARNLFDENYGLASTQQRKNKDIPEGGSKGVILLDP
FG04117.1 	LRFKDISRGGIRIVKSRNKEAYGINARSLFDENYGLASTQQRKNKDIPEGGSKGVILLDP
AN7451.1  	LRFRDIARGGIRIVKSRNKEAYSINARSLFDENYNLANTQQRKNKDIPEGGAKGVILLDV
          	***:*::**********.**** ****.******.**.*************:******* 

MG05247.1 	KQQDKAREAFEKYIDSILDLLLPAETPGIKNPIVDLYGKPEILFMGPDENTADLVDWATE
NCU00461.1	KQQDRHREAFEKYIDSILDLLLKAETPGIKNPIVDLYGKEEILFMGPDENTADLVDWATE
FG04117.1 	KQQNRAREAFEKYIDSILDLLLPAETPGIKNPIVDLYGKEEILFLGPDENTAELVDWATE
AN7451.1  	NHQDKARVAFEKYIDSILDLLLPPASPGIKDPIVDLYGKDEILFMGPDENTAELVDWATE
          	::*:: * ************** . :****:******** ****:*******:*******

MG05247.1 	HARSRNAPWWKSFFTGKSPKLGGIPHDTYGMTTLSVREYVKGIYRKLELDPSKVRKMQTG
NCU00461.1	HARARGAPWWKSFFTGKSPRLGGIPHDSYGMTTLSVREYVKGIYRKLELDPSKIRKMQTG
FG04117.1 	HARSRGAPWWKSFFTGKSPKLGGIPHDTYGMTTLSVREYVKGIYRKLELDPSTIRKMQTG
AN7451.1  	HARNRGAPWWKSFFTGLSPRLGGIPHDTYGMTTLSVRQYVLGIYRKLKIDPSTIRKLQTG
          	*** *.********** **:*******:*********:** ******::***.:**:***

MG05247.1 	GPDGDLGSNEILLSNEMYTSIVDGSGVLCDPNGIDIDELRRLAKQRVMISNFDMSKLSKD
NCU00461.1	GPDGDLGSNEILLSNETYTAIVDGSGVLCDPNGIDKDELRRLAKARAMISNFDIAKLSKD
FG04117.1 	GPDGDLGSNEIKLGNEKYTAIVDGSGVLVDPKGLDREELLRLAHGRKMIIEYDVSKLSAE
AN7451.1  	GPDGDLGSNEILLANEKYTAIVDGSGVIVDPQGLNREELVRLAKKRATISEFDVSKLSPN
          	*********** *.** **:*******: **:*:: :** ***: *  * ::*::*** :

MG05247.1 	GYRVLCEDVNVTLPNGQVVANGTAFRNTYHLLDTGLTDVFVPCGGRPESIDLVSVAKIIK
NCU00461.1	GYRVLCDDTNVTLPNGEVVHNGTAFRNTYHLRDNGITDMFVPCGGRPESIDLSSVNKLIK
FG04117.1 	GYRVLCEDVNLTLPSGEVVNNGTSFRNTFHLRDTGAVDVFVPCGGRPASIDLISVNRLIK
AN7451.1  	GYRVLVDESNVHLPSGELVHNGMVFRNMFHLRKELTYDTFVPCGGRPESIDLSNVGKLIE
          	***** :: *: **.*::* **  *** :** .    * ******** **** .* ::*:

MG05247.1 	DGRSTIPYIVEGANLFCSEPSRMRLENAGCIFIKD2ASANKGGVTSSSLEVLASLAFDDE
NCU00461.1	DGKSTIPYIVEGANLFITQDAKLRLEEAGCIVYKD~ASANKGGVTSSSLEVLASLSFDDK
FG04117.1 	DGKSVVPYLVEGANLFITQEAKLRLEAAGCILYKD~ASANKGGVTSSSLEVLASLSFDDE
AN7451.1  	NGKSTIPYIVEGANLFITQDSKLRLEKAGCILFKD~ASANKGGVTSSSLEVLASLSFDDQ
          	:*:*.:**:******* :: :::*** ****. ** *******************:***:

MG05247.1 	GFVEHMCVDAAGNAPEFYKAYVKQVQE1TIQRNAYLEFEAIWRENAETGVPRPVLSDKLS
NCU00461.1	GFVTHMCHDSRGNAPEFYQAYVKEVQN~KIQDNARLEFEAIWREHEQTGLPRSVLSDKLS
FG04117.1 	GFVQNMCHDANGQAPQFYQDYVKQVQL~KIQENARLEFEAIWREHEQTGTPRSILSDKLS
AN7451.1  	GFVQNMCVGDDDSVPEFYREYVKQVQE~VIKENATLEFEAIWREHEQTGIPRSVLSDRLS
          	*** :** .  ...*:**: ***:**   *: ** *********: :** **.:***:**

MG05247.1 	VAITTLDA0ELQHSELWENEKIRVSVLKDALPNLLIEKIGLENIIAR~VPDNYLRAIFGS
NCU00461.1	LAITSLDE~DLQRSELWDNEKIRRSVLADALPNLLINKIGLDTIIER~VPDSYLRAIFGS
FG04117.1 	VAITDLDE~KLQHSDLWDNEKIRRSILEDALPRLLLEKIGLDTLIAR~IPDSYLRSIFGS
AN7451.1  	VAITQLDE~ELQKTELWDNVELRRSVLNDALPKLLLDKIGLDTILQR0VPENYLRAIFGS
          	:*** **  .**:::**:* ::* *:* ****.**::****:.:: * :*:.***:****

MG05247.1 	YLASRFVY2TYGTTSNSFSFYNF~MTAKMAQIQ--
NCU00461.1	YLASRFVY~EFGSSPSQFAFYDF2MSKRMGNINKE
FG04117.1 	YLASRFVY~EFGSSPSQFAFFDL~-----------
AN7451.1  	HLASRFVY~EYGSSPSQFSFFNF2MTKRLAQSKA-
          	:*******  :*::...*:*:::  :   .. .
```
