# Supplementary material for: Patterns of Intron Gain and Loss in Fungi
Source: PLoS Biol. 2004 Nov 30;2(12):e422. doi: 10.1371/journal.pbio.0020422 (PMC532390; doi:10.1371/journal.pbio.0020422)
Supplement: Table S1 — Also available at http://genes.mit.edu/NielsenEtAl/. (4.3 MB ZIP). [file pbio.0020422.st001.zip › NielsenEtAl/html/1163.html]

AN0747.1.NCU02549.1.MG03600.1.FG00863.1


```
 CLUSTAL W (1.82) Multiple Sequence Alignments - Introns Inserted


Sequence 1: NCU02549.1	476 aa
Sequence 2: MG03600.1	473 aa
Sequence 3: FG00863.1	474 aa
Sequence 4: AN0747.1	479 aa
Alignment Length: 480 aa
Number Identitical Residues: 306 aa
Alignment Score (without introns) 13761


MG03600.1 	MASRRLALNLSKGLRARAG--LSMPMRRGFATPVP-TPAGTQTTTLKNGLT0VATQYSPY
NCU02549.1	MASRRLALNLAQGVKARAGG-VINPFRRGLATPHSGTGIKTQTTTLKNGLT0VASQYSPY
FG00863.1 	MASRRLALNLSRGLRNRAGFSAAVPFTRGFATPST--VGKTQTTTLKNGLT0VATEHSPF
AN0747.1  	MASRRLAYNFNQALRSRAALKSIQPVKRGFASPVA-LPSTTQSTTLSNGFT0IATEYSPW
          	******* *: :.:: **. .   *. **:*:* .     **:***.**:* :*:::**:

MG03600.1 	AQTSTVGMWIDAGSRAETNENNGTAHFLEHLAFK0GTQRRTQHQLELEIENMGAHLNAYT
NCU02549.1	AQTSTVGMWIDAGSRAETDETNGTAHFLEHLAFK0GTTKRTQQQLELEIENMGAHLNAYT
FG00863.1 	SQTSTVGVWIDAGSRAETDENNGTAHFLEHLAFK0GTAKRTQQQLELEIENMGGHLNAYT
AN0747.1  	AQTSTVGVWIDAGSRAETDKTNGTAHFLEHLAFK0GTSKRSQHQLELEIENMGAHLNAYT
          	:******:**********::.************* ** :*:*:**********.******

MG03600.1 	S0RENTVYFAKSLNEDAPKCVDILADILQNSKLDEAAIERERDVILRESEEVEKQLEEVV
NCU02549.1	S0RENTVYFAKALNEDVPKCVDILQDILQNSKLEESAIERERDVILRESEEVEKQLEEVV
FG00863.1 	S0RENTVYFAKAFNSDVPQCVDILSDILQNSKLEESAIERERDVILRESEEVEKQVEEVV
AN0747.1  	S0RENTVYYAKSFNNDVPKAVDILADILQNSKLESAAIERERDVILREQEEVDKQLEEVV
          	* ******:**::*.*.*:.**** ********:.:************.***:**:****

MG03600.1 	FDHLHATAFQHQPLGRTILGPRENIRDITRTELVNYIKQNYTADRMVLAAAGGVPHEQLV
NCU02549.1	FDHLHATAYQHQPLGRTILGPRENIRDITRTELVNYIKNNYTADRMVLVGAGGVPHEQLV
FG00863.1 	FDHLHATAFQHQPLGRTILGPRQNIRDITRTELTDYIKNNYTADRMVLVGAGGIPHEQLV
AN0747.1  	FDHLHATAYQHQPLGRTILGPKENIQTITRDNLTDYIKTNYTADRMVLVGAGGIPHEQLV
          	********:************::**: *** :*.:*** *********..***:******

MG03600.1 	ELADKYFANLPGETAKTS-AYIQSKAK--PDFIGSDVRIRDDTIPTANIAIAVEGVSWSD
NCU02549.1	EMADKYFSKLPATAPVSS-ASILSKKK--PDFIGSDIRIRDDTIPTANIAIAVEGVSWSD
FG00863.1 	QLAEKHFAGLPSSGPQTG-AYLRSKQK--ADFMGSDVRVRDDNMPTANIALAVEGVSWNS
AN0747.1  	KLAEQHFGSLPSKPPTSALAALTAEQKRQPEFIGSEIRIRDDTLPTAHIALAVEGVSWKD
          	::*:::*. **.  . :. * : :: * ..:*:**::*:***.:***:**:*******..

MG03600.1 	DDYFTALVTQAIVGNYDKAMGNAPHQGSKLSGFVHSNDLANSFMSFSTSYSDTG2LWGIY
NCU02549.1	DDYFTGLVTQAIVGNYDKALGNAPHQGSKLSGFVHKHDLATSFMSFSTSYSDTG2LWGIY
FG00863.1 	EDYFTALVAQAIVGNYDKAVGQAPHQGSKLSGWVHKHDLANSFMSFSTSYNDTG2LWGIY
AN0747.1  	DDYFTALVAQAIVGNWDRAMGNSPYLGSKLSSFVERNNLANSFMSFSTSYSDTG2LWGIY
          	:****.**:******:*:*:*::*: *****.:*. ::**.*********.*** *****

MG03600.1 	LVTDKLTRVDDLVHFALREWSRLSQSVSEAEVERAKAQLKASILLSLDGTTAVAEDIGRQ
NCU02549.1	LVTDKLDRVDDLVHFSLREWTRLCSNVSEAEVERAKAQLKASILLSLDGTTAVAEDIGRQ
FG00863.1 	LVSDKPDRVDDLVHFAIREWMRLCTNVSASETERAKAQLKASILLSLDGTTAVAEDIGRQ
AN0747.1  	LVSENMTGLDDLIHFALREWSRLSFNVTAAEVERAKAQLKASILLSLDGTTAIAEDIGRQ
          	**:::   :***:**::*** **. .*: :*.********************:*******

MG03600.1 	IVTTGRRMNPAEIERVIDAVTAKDVMSFAQRKLWDKDVAVSAVGSIEGLFDYARIRGDMS
NCU02549.1	IVTTGRRMSPAEIERIIDAVSAKDVMDFANKKIWDQDIAISAVGSIEGLFDYARIRGDMS
FG00863.1 	LVTTGRRMAPNEIERKIDAITEKDIMDFANRKLWDRDIAVSAVGTIEGLFDYQRLRNTMK
AN0747.1  	IITTGRRLSPEDIERTIGQITEKDVMDFANRKLWDQDIAMSAVGSIEGILDYNRIRSDMS
          	::*****: * :*** *. :: **:*.**::*:**:*:*:****:***::** *:*. *.

MG03600.1 	RNF-
NCU02549.1	RNAF
FG00863.1 	PKF-
AN0747.1  	RNAY
          	 :
```
