# Supplementary material for: Patterns of Intron Gain and Loss in Fungi
Source: PLoS Biol. 2004 Nov 30;2(12):e422. doi: 10.1371/journal.pbio.0020422 (PMC532390; doi:10.1371/journal.pbio.0020422)
Supplement: Table S1 — Also available at http://genes.mit.edu/NielsenEtAl/. (4.3 MB ZIP). [file pbio.0020422.st001.zip › NielsenEtAl/html/1164.html]

AN3581.1.NCU08352.1.MG01284.1.FG00871.1


```
 CLUSTAL W (1.82) Multiple Sequence Alignments - Introns Inserted


Sequence 1: AN3581.1	356 aa
Sequence 2: FG00871.1	330 aa
Sequence 3: NCU08352.1	334 aa
Sequence 4: MG01284.1	349 aa
Alignment Length: 385 aa
Number Identitical Residues: 188 aa
Alignment Score (without introns) 8831


MG01284.1 	--------~---------------------~-MYEGFMANGTAAGGQLTTTTDVENFPGF
NCU08352.1	-MHSKVVI~IGSGPAAHTAAIYLARAELKP1VLYEGFMANGIAAGGQLTTTTEIENFPGF
FG00871.1 	-MHSKVVI1IGSGPAAHTAAVYLARAELKP1VLYEGFMANGIAAGGQLTTTTEVENFPGF
AN3581.1  	MVHSKVVI1IGSGPAAHTAAIYLSRAELKP~VLYEGMLANGTAAGGQLTTTTDVENFPGF
          	   :.     .:..:: :::   : :. ..  :***::*** **********::******

MG01284.1 	PKGIGGQELM0DAMREQSSRFETEIISETVAKVDLSSRPFKYCTEWSP----EVMHTADT
NCU08352.1	PDGIMGQELM~DKMKAQSERFGTQIISETVAKVDLSARPFKYATEWSP----EEYHTADS
FG00871.1 	PKGIMGGELM0DNMRAQSERFGTEIITDTVATLDLSSRPYKYTTEFSP----EETHTAET
AN3581.1  	PDGIGGSELM~DAMRKQSIRFGTEVITETISRVDLSQRPFKLWTEWNDGPDNEPARTADA
          	*.** * *** * *: ** ** *::*::*:: :*** **:*  **:. ....*  :**::

MG01284.1 	LVIATGASARRLGLPGEDKYWQNGISACAVCDGAVPIFRNKPLFVIGGGDSAAEEAMFLT
NCU08352.1	IILATGASARRLHLPGEEKYWQNGISACAVCDGAVPIFRNKHLVVIGGGDSAAEEAMYLT
FG00871.1 	VILATGASARRLNLPGEDKYWQNGVSACAVCDGAVPIFRNKPLFVIGGGDSAAEEATFLT
AN3581.1  	VIIATGANARRLNLPGEDVYWQNGISACAVCDGAVPIFRNKPLFVIGGGDSAAEEAIFLT
          	:::****.**** ****: *****:**************** *.************ :**

MG01284.1 	KYGSHVTVLVRKDKLRASSIMAKRLLSHPKVTVKFNSVGVEVKGGE--DGLMSHMVIKDV
NCU08352.1	KYGSHVTVLVRKDKLRASSIMAHRLLNHEKVTVRFNTVGVEVKGDD--KGLMSHLVVKDV
FG00871.1 	KYASHVTVLVRRDVLRASRTMANRLLNHPKCTVLFNSGATEIRGGE--DGLMSHLVVKNN
AN3581.1  	KYGSSVTVLVRRDKLRASKAMASRLLANPKVTVRFNTVATEVLGEKKLNGLMTHLRVKNV
          	**.* ******:* ****  ** *** : * ** **: ..*: * .. .***:*: :*: 

MG01284.1 	VTGKEETLEANGLFYAIGHDPATQLVKGQLETDEEGYIVTKPGTPLTSVEGVFACGDVQD
NCU08352.1	TTGKEETLEANGLFYAIGHDPATALVKGQLETDADGYVVTKPGTTLTSVEGVFAAGDVQD
FG00871.1 	KTGEEKVHEANGLFYAIGHDPATTLVKGQVDMDEDGYIKTIPGTTYTNVEGVFAAGDVQD
AN3581.1  	LTGEEETLEANGLFYAVGHDPATALVKGQVELDEDGYIATKPGTSFTSVEGVFACGDVQD
          	 **:*:. ********:****** *****:: * :**: * ***. *.******.*****

MG01284.1 	KRYRQAITSAG1SGCMAAMDAEKFLAEQEDVEPDREARI2FYREGTPSG0SAKLKL~P0S
NCU08352.1	KRYRQAITSAG1TGCMAALDAEKFLSEHE----------~----ETPAE~HRDTSA~V~Q
FG00871.1 	KRYRQAITSAG~TGCMAALEAEKFLADHE-DD-------~----ERADE~RPNPN-~-~-
AN3581.1  	KRYRQAITSAG~SGCIAALEAERFIGESESNE-------~----EIPPA~HANPAL2V~E
          	*********** :**:**::**:*:.: *. .              .     .      .

MG01284.1 	WQGLSLASNTLVLAMYGERDNAVRITLTFQKWA
NCU08352.1	GNL------------------------------
FG00871.1 	---------------------------------
AN3581.1  	VNAIYFSN---------LKDHASR---------
          	 .    :.           . :
```
