# Supplementary material for: Patterns of Intron Gain and Loss in Fungi
Source: PLoS Biol. 2004 Nov 30;2(12):e422. doi: 10.1371/journal.pbio.0020422 (PMC532390; doi:10.1371/journal.pbio.0020422)
Supplement: Table S1 — Also available at http://genes.mit.edu/NielsenEtAl/. (4.3 MB ZIP). [file pbio.0020422.st001.zip › NielsenEtAl/html/1165.html]

AN9063.1.NCU00579.1.MG03047.1.FG08586.1


```
 CLUSTAL W (1.82) Multiple Sequence Alignments - Introns Inserted


Sequence 1: NCU00579.1	1238 aa
Sequence 2: MG03047.1	1283 aa
Sequence 3: FG08586.1	1279 aa
Sequence 4: AN9063.1	1243 aa
Alignment Length: 1308 aa
Number Identitical Residues: 572 aa
Alignment Score (without introns) 27571


MG03047.1 	MSEAGDS2SHKRSKSKSSARLSILRRKERKSGDEDDGISDDGSSLGQTHQSRNSTSLAPP
NCU00579.1	MSETGES2LHKRSKS---AARSLLSR--RKPPVDDDMASDDGR--------RASGSYSTP
FG08586.1 	MSETGDS2THKRSKS--AAALSLLRR---NNTRDEESGSEDGR----LRQAPSSSNPTLT
AN9063.1  	MSEKVEG2-HKRSKS--ALALAILHRDKSKDSHEEVGSRENGSPDSAPSSPVKVNNNSSP
          	***  :.  ******  :   ::* *.. :   ::    ::* . .   ..    . : .

MG03047.1 	LTSSLSQTSTTMSLQQSIAPPSSRGHTRVSSTSIPSYQQGPAS-ASKSNMTLVDK--AAS
NCU00579.1	QNIQIPSQQASRGHSSRLSTTGS-----VLGRTVSNHQASPSVGGARSPSMLFDKGAAAS
FG08586.1 	PINPMAHQQSPRGHAAKQSVSSAG-----LSPTASNPPQSKPQPVLTSP--SADKS-TAS
AN9063.1  	SVSAISTHRSPSLRRSRQETSPP-----AMSADSSSDPAALGREGDVIEKLPSLTPVDVT
          	    :.   :.         . .       .   ..   .              .   .:

MG03047.1 	LEQSVRKFRVVEALRNGDSASISKAIRESADGGRRGSTVSLTGGSGLILDDTTVLHLAIQ
NCU00579.1	LESSVRKFRIVEALRNGDTASISRAIRDTAEHNPRMSISSAITGP---LEDTTILHLAIQ
FG08586.1 	LEQSVRKFRFVEALRSGDTSSISRAIRETAENAPRPSISSVSGSSSGTLDDTTILHLAIQ
AN9063.1  	LNQSVRTFRLFEILRSGDTTAISKAIKESRDPDGVNGLS-----------GTTILHLAVQ
          	*:.***.**..* **.**:::**:**::: :     .             .**:****:*

MG03047.1 	CAELTVVEYVLSDGAGYIDINSRDKDGNTPLHIAAQQGRTQIVRMLLEREGINDSIANFQ
NCU00579.1	CAEQTVVEYVLSDGAGSLDINARDKDGNTPLHIAAQQGRTHIVRQLLEHKDINDAIANHQ
FG08586.1 	CAEFPVIEYVLSDGQGSIDVNARDKDGNTPLHLAAIQGRTTVVKLLLEQKDINDAIANSQ
AN9063.1  	CAEPQVVEYVLSAG-NDIDINARDREGNTPLHLAAQLGRGPVVRELLNRPEINDSIVNYR
          	***  *:***** * . :*:*:**::******:**  **  :*: **::  ***:*.* :

MG03047.1 	GRLPIDLARNPDIFQQLQLSRSIFVDEKVKEVQQLIAASDYKKLEELLEEPRVKLVLDIN
NCU00579.1	GRLPIDLARNPDIFQQLQLARSLFAEDKVRQVQDLILHGDFKTLEEVLEEPRFKTVLDIN
FG08586.1 	GKLPLDVARNPEIFQLLQLSRSLFAEAKVKQVQELIARGNYAALAGVLEEHRVKTVLDIN
AN9063.1  	GQTALEASRAPEIFQQLQLARSLFIDSKTQEIQSLIAKGEYDKLEKLLEEPRVEGILDVN
          	*: .:: :* *:*** ***:**:* : *.:::*.**  .::  *  :*** *.: :**:*

MG03047.1 	GTEFVSDPITSQSGGTLLHEAARRKNNKLIQVLLLHGADPFRRDRKGKLPQDVTKDDVTR
NCU00579.1	STEFAAESATVESGGTLLHEAARRRNTKLIQVLLLHGADPFRRDRNGKLPQDVTKDEITK
FG08586.1 	SPEFASEPVTVQTGGTLLHEAARKKNTNLIQVLLLHGADPFRRDRKGKLPQSVTTDDATK
AN9063.1  	ALDLVTDPTTLHSGGTLLHEGARKKDTRLIQILLMHGADPFRRDKKGKLPQDVTKDDRTR
          	. ::.::. * .:*******.**:::..***:**:*********::*****.**.*: *:

MG03047.1 	AMLKKSPAAVAAQRGIQEKTVLGQAASQGVAS--ASPGDPLAGREAREMKGYLKKWTNYR
NCU00579.1	AMLKKSPAAVAAQRGIQEKAVLG-SATHGAAA--AASGDPMAGREAREMKGYLKKWTNYR
FG08586.1 	GILKKSPAAVAAQRGIQEKAVLGQAASQGTAG--SASSDPLAGREAREMKGYLKKWTNYR
AN9063.1  	AIVKKSPAAVIAQRGIQEKAILGTSSGQGVSGRPGAGEASFAGKDSREMKGYLKKWTNYT
          	.::******* ********::** :: :*.:. ..:   .:**:::************* 

MG03047.1 	KGYQLRWFVLEDGVLSYYKHQD~DTGSACRGAINMRIARLHMSPDEKTKFEIIGKSSVKY
NCU00579.1	KGYQLRWFVLEDGVLSYYKHQD~DAGSACRGAINMRIAKLHMTPDEKTKFEIIGKNSVKY
FG08586.1 	KGYQLRWFVLEDGVLSYYKHQD~DAGSACRGAINMRIAKLHMSPDEKTKFEIHGKSSVKY
AN9063.1  	SGYKLRWFVLEDGVLSYYKHQD1DTGSACRGAINMKIARLNMDSQDKTRFEIYGKSSVKY
          	.**:****************** *:**********:**:*:* .::**:*** **.****

MG03047.1 	TLKANHEVEAKRWFWALNNSIQFSKDQQKEEDRQRARDAELLAQAKAGHNHGGSIAGDVS
NCU00579.1	TLKANHEVEAKRWFWALNNSIQWTKDQAKEEERQRVRNAELLKQAKAEHAHSVSDAG--S
FG08586.1 	TLKANHEVEAKRWFWALNNSIQWSKDQAKEEEKRAARGAELLRQAKADPST-LSLQESHS
AN9063.1  	HLKANHVVEAKRWFWTLNNAIQYAKDEAKEEEKRQTKHAEALRQAKLDQAE-GRPSENPS
          	 ***** ********:***:**::**: ***::: .: ** * ***           . *

MG03047.1 	EAASVAESKSRTSLQINR---MQSTASHRPSEMGASTAGSEAGDAPVNDGGFENEGGKGT
NCU00579.1	DNASFVEHR-RQSVQLSR---MHSTARASRASYVASGNGSNEEDDFVDAG---TEADKGE
FG08586.1 	EGTSVTDLRRNSSQIPSRSLSKISSVDQRPSHTSPGTSGSIEEDEFVDVET--DAGTSRV
AN9063.1  	ESPSFRSRGPLSLGVPSTSNTKLSTYTSRTTLDGVPADDDGSMYGSLEQGP-SQSDINRV
          	: .*. .         . : :  *:     :       ..      ::    .    .  

MG03047.1 	GTAPTMYDTLDDDDDEDNNSSGAGGQPPVTKDAFNITAQSARLQLETLASVNAAMIAESS
NCU00579.1	------HHHADDDDDDYGEGSSGQDVPSANKDAFNITAQSARLQLDTMAQVTAALMAETN
FG08586.1 	HRNGAPTNNDMDDDDDYGDDMSLHEEPTATKDALNITAQSAKLQLETMSHVHQALLNELN
AN9063.1  	ASHVTTAPDLEGDDDDYGDYASSRETPPTDKDAMNITAQSVKLQLDILASVASSLQKT--
          	    :      .***: .:  .    *.. ***:******.:***: :: *  ::     

MG03047.1 	KQSSLSLSDPQVTQALQAYDAAVRSLTGLVGDLLRISRDRDAYWQHRLDREADMRRMWED
NCU00579.1	KNSDLRLSDPKASQALATYDAAIRSLTGLVGDLLRISKDRDAYWQYRLDRESEMRQMWEE
FG08586.1 	QNPSTPLSDNSVSQALGTYDGAIRSLSTLVADLLRISKDRDAYWQYRLDREANMRRMWEE
AN9063.1  	-DQSTALSDQAVAQALTAYEEAVSSLKDLVQNLLKISRDRDSYWQYRLNREAHLRKMWEE
          	 : .  ***  .:*** :*: *: **. ** :**:**:***:***:**:**:.:*:***:

MG03047.1 	SMAQVAQEQEELKARVGEAEEKKKAAKRILKEVIASGGLEEKAAAGLGIDSSPALIQS--
NCU00579.1	SMAQVAREQEALEARVGEAEAKRKITKRILKEALGSG----------IIDEGQVKATA--
FG08586.1 	SMAQVAREQEVLEARVGEAEKKRKATKRALREVIESGIPVGAELPVQDAHVEKELHESRE
AN9063.1  	SMARIAQEHEELQSKMGESEEKRRRTKRALKEALENTPNSISRTAVKAASVEAGLDGE--
          	***::*:*:* *::::**:* *:: :** *:*.: .        .               

MG03047.1 	-EAVEVNANDTPKPAADAAFTRAKSPVLSIQRRQTALAQVADLSDSETEDEDEFFDAIDA
NCU00579.1	-PTVAAAAEATDEADAEQADARPQSPVQSIRRQKTIRDQVAELSDSDS-DEEEFFDAVDA
FG08586.1 	KEDEEGQFEDVPTKPSALASASAQPKSPTTKTVRRKPTIHVDLSESESEQEDEFFDAVDA
AN9063.1  	-EDLSPQPPQIVEVASHIEEQKSRQPQLHRKNSALSNISSLYDSESDNDDEDEFFDAIDA
          	               :      .:      :            *:*:..:*:*****:**

MG03047.1 	GEVEFDPAMPPSETSPT---EENNKIVVSSGVDISSSFKGYENGIRTKLKIEADNRPKIS
NCU00579.1	GTVEVS-QLPPSEPVAS---QSDTQLVISDGTDISDSFKGYENGIRTKLKMDADNRPTIS
FG08586.1 	GQVEVS-ELPADEIQPK---SQD--IVVSGGMDISSSFKGYENGIRTRLKMDADDRPKIS
AN9063.1  	GEIEVVDRTAPEVHEDEETIPDEDKLRAVRRSEIAPSFKGYEEPIRERLKMDYDNRPKIS
          	* :*.    ...     .:  .: .:      :*: ******: ** :**:: *:**.**

MG03047.1 	LW0GILKSMIGKDMTKMTLPVTFNEPTSLLYRCGEDMEYADLLDLAAERSDSIERLVYVA
NCU00579.1	LW0GILKSMIGKDMTKMTLPVSFNEPTSLLYRCAEDMEYADLLDLAADRADSIERLIYVS
FG08586.1 	LW0GILKSMIGKDMTKMTLPVSFNEPTSLLYRAGEDMEYADLLDLAADRADSIERLIYVA
AN9063.1  	LW0GILKSMIGKDMTKMTLPVSFNEPTSLLQRVAEDLEYTDLLDVAADRTDSMERLVYVA
          	** ******************:******** * .**:**:****:**:*:**:***:**:

MG03047.1 	AFAASEYASTIDRVAKPFNPLLGETFEYVRPDKNYRFFIEQVSHHPPVGAAWAESPNWTY
NCU00579.1	AFAASEYASTIGRVAKPFNPLLGETFEYVRPDKNYRFFIEQVSHHPPIGAAWAESPKWTY
FG08586.1 	AFAASEYASTIGRVAKPFNPLLGETFEYVRPDKGYRFFIEQVSHHPPVGAAHAEALKWSY
AN9063.1  	AYAASEYASTIGRVAKPFNPLLGETFEYVRPDKGYRFFVEQVSHHPPIGVALAESPKWDY
          	*:*********.*********************.****:********:*.* **: :* *

MG03047.1 	W~GESAVKSKFYGRSFDVNPLGTWFLRLRPKSGGKEDLYTWKKVTTSVVGIMTGNPVVDN
NCU00579.1	Y~GESAVKSKFYGKSFDVNPLGTWFLKLRPTSGGKEDLYTWKKVTSSVIGIITGNPVVDN
FG08586.1 	W~GESAVRSKFYGKSFDINPLGTWFLKLRPTAGGKEELYTWKKVTSSVIGIITGNPTVDN
AN9063.1  	W0GESSLKSKFYGKSFDINLLGTWFLKLRPVSGG-EELYTWKKVTSSVIGIITGNPTVDN
          	: ***:::*****:***:* ******:*** :** *:********:**:**:****.***

MG03047.1 	YGPMEIKNWTTGEVCHLEFKARGWKASSAYLISGKIVDADGRVRVSLGGRWNSRLYARLT
NCU00579.1	YGVMEIKNWTTGEVSYVEFKPRGWTKSSAYLIGGKILDANGQVRVSLGGRWNSKFYARLT
FG08586.1 	YGVMEIKNWTTGEVAHVEFKPRGWKASSAYQVSGKVTDASGKVRVSLGGRWNSKLYARLT
AN9063.1  	YGLMEIKNWTTGEICYLDFKPRGWKASSAYQVTGKVVDKEGSPRWSIGGRWNDKIYARHT
          	** **********:.:::**.***. **** : **: * .*  * *:*****.::*** *

MG03047.1 	PGYEATVEEPKNADTVGVYRGSMTDTNKAFLVWQANPRPKDIPFNLTPFVLTFNHIDDKL
NCU00579.1	PGYEATIEEKSGNETV--HQGSINDPSKAFLIWQANPRPTGIPFNLTPFVVTFNHLDDNL
FG08586.1 	PGYEAAVDEPKESGGDMAHSG-LSDPNRAYLIWKANERPTGIPFNLTPFVLTFNHIDDQL
AN9063.1  	PGFEATVSGPEQ---------------KAQLVWQAHPRPTGIPFNLTPFVITLNALTDSL
          	**:**::.  .                :* *:*:*: **..*********:*:* : *.*

MG03047.1 	EPWLAPTDSRLRPDQRAMEDGRYDEAGEEKNRLEEAQRARRRKREENGEEFVPKWFEKAR
NCU00579.1	KPWLAPTDSRFRPDQRAMEEGEYDFAATEKNRLEEAQRARRKARESKGEEFKPAWFTKAR
FG08586.1 	RPWLPPTDSRLRPDQRAMEDGEYDFAAEEKNRLENAQRSRRRLREERGEEFVPAWFQKAR
AN9063.1  	RPQLPPTDTRLRPDQRAMEEGEYDFAATEKHRVEEKQRAKRREREANGEEYKPKWFSKAK
          	.* *.***:*:********:*.** *. **:*:*: **::*: ** .***: * ** **:

MG03047.1 	CEITGEEYWRFNGGYWEARERVAVEGK--KAWEGCEPIYEDAS---------
NCU00579.1	CEITGEEYWQFNGEYWNRRAKAGPNGDP-SAWQGLEPIFQDA----------
FG08586.1 	CEITGEEYWQFNGKYWQQREKAGPQGDPQAAWEGLEPIYEDHVDENQGSVQY
AN9063.1  	CPITGEEYWAHTGDYWGCRARQD--------WSKCEDIF-------------
          	* ******* ..* **  * :          *.  * *:
```
