# Supplementary material for: Patterns of Intron Gain and Loss in Fungi
Source: PLoS Biol. 2004 Nov 30;2(12):e422. doi: 10.1371/journal.pbio.0020422 (PMC532390; doi:10.1371/journal.pbio.0020422)
Supplement: Table S1 — Also available at http://genes.mit.edu/NielsenEtAl/. (4.3 MB ZIP). [file pbio.0020422.st001.zip › NielsenEtAl/html/1166.html]

AN5677.1.NCU09537.1.MG09531.1.FG07433.1


```
 CLUSTAL W (1.82) Multiple Sequence Alignments - Introns Inserted


Sequence 1: NCU09537.1	879 aa
Sequence 2: FG07433.1	842 aa
Sequence 3: MG09531.1	891 aa
Sequence 4: AN5677.1	890 aa
Alignment Length: 934 aa
Number Identitical Residues: 421 aa
Alignment Score (without introns) 20384


MG09531.1 	MPEFSDSFWSSDYAAG1LGVLFGKLQQGVLENRQVLTIARLRAEAEEAYGNKLSEIAPSA
NCU09537.1	MPGFADSFWSNDYAAG1LGVLFGKLQQGVVENRQLLTIARMRAEAEDVYGQRLSEIAPAV
FG07433.1 	MPGFADSFWSSDYAAG~LGVLFTKLQQGVHENRQVLTIARLRAEAEETYGQRLGDIAPAA
AN5677.1  	MPGFADSFWTPDYATG~LGVLYGKLQQGIVENKQILTIASMRADAEEIYSSKLGDIAPTV
          	** *:****: ***:* ****: *****: **:*:**** :**:**: *..:*.:***:.

MG09531.1 	DRIQGGFGRDDGATLRK0AYEGVRTEMQEGAKNHKKIAQSIRDLVVNPFSRWCDAHEQRV
NCU09537.1	DKIPNGFNRDDGASVRK0AYEGVRTEMEDASKNHKKIAQNIRDLVVNPFTRWCDAHESRL
FG07433.1 	DKVAGGFSRDDGATVRK0AFDGMRNEMQDAARNHRRIAQSIRDLVVNPFSRWCDSHEARI
AN5677.1  	DKMIAGFAKDDGASVRK0AYGGTRTEMVEASRNHKKIASNIRELVVSPFKRWCDQHEARI
          	*::  ** :****::** *: * *.** :.::**::**..**:***.**.**** ** *:

MG09531.1 	QDSQDELQSRIKAHDKQAELVKKLRSNYFNKCRLVEDLEEENKLAFQDPEN-SPSKTPTN
NCU09537.1	QNSQEELQAKIKAHDKQAELVKKLRSNYFNKCRLVEDLEEENKLAFQDPESAASPKPKQP
FG07433.1 	QDSQDELQVRIKAHDRQAEAVKKLRSVYFNKCRLVEDLEEENKLAFQDPE--TSPKAGQN
AN5677.1  	ENSHDDLQARIKEHTKQVDLVKKLRSQYFNKCRVLEDLEEENKLAFQAPET--SPKIKP-
          	::*:::** :** * :*.: ****** ******::************ **.  ..*    

MG09531.1 	VPEIKVDK-ENEVDDEDEMYEIGDEMFTRDQIKKIVAHMLDHIKMGETKVPILGTYLNTS
NCU09537.1	IPEIKVEP---EEEEDDEPYEIGDETYSPDQIKKILAHMLNNIKMGETKVPILGTYQNTS
FG07433.1 	IPEIKVQPHKEEEPEEEELYEIGDDTYQPEQVKKIISQMLSSIKMGETKVPILGTYLNTS
AN5677.1  	TPKIILPD---KEPVEDEPVELGDRVYTPDDLKKLLVHMLETIPQGEIKVPIIGTYQNTS
          	 *:* :     :   ::*  *:**  :  :::**:: :**. *  ** ****:*** ***

MG09531.1 	AGSDIVEYLQRNMGTSSVSYAERIGQDLVNHKFLRLIGNVGNTFANSSRMFYQWQSKAFT
NCU09537.1	AGTDIVEYLQRHMGTTSISYAERIGQDLITHGFLRLIGNVGNTFANSSRMSYQWRPQAFK
FG07433.1 	SGSDIVEYLQRSMGNINVAYAERIGQDLVNNGFLRLIGNVGSTFANSSKMFYQWQSKAFT
AN5677.1  	TGADIVEYTQKYLNATSISYAERIGQDLVDNGFLRLVGNMGSTFANSSKLRYQWRSKCFQ
          	:*:***** *: :.  .::*********: : ****:**:*.******:: ***:.:.* 

MG09531.1 	IAGVPEKKTQLNRTLSM-PSTTSEGSDSPVVGAVSEYLEKWNL--VNSGPPNETPAERLR
NCU09537.1	LAGVPEKKAPVGRTFSMPLSNGSEGNDSPVVGVVSEYLANWNIPGVNNGRPNETPSERMR
FG07433.1 	MAGVPEKKS-INRTFSL-ASTGSEGAESP-VGTVSEYLANWKV--LNNSHPGETPSQRMQ
AN5677.1  	ISGIPEKTTALMRVTSV--ATSEDGIDSP-ISSVSEMLAGWNP--LNNPHPNETPAEKLR
          	::*:***.: : *. *:  :. .:* :** :. *** *  *:   :*.  *.***:::::

MG09531.1 	REQRESDERYKVGVRKLDELRCDLEEQIFVHLRFLERCELDRLKAIKTVILDFSGTISNV
NCU09537.1	REAREADEKYKAAVQKLDEMRCELEEAIFLHLKFLERCELDRLKAIKTVVLDFSGTISNV
FG07433.1 	REAREADEKYREGVRKLDELRCELEEAIHLHLKFLERCELDRLKAVKTVILDFSGTIGNV
AN5677.1  	REAREADERYKAAVRKLDLIRCKLEEEIVANLRFMEQCELDRLKAIKAVVLDFSGAISNV
          	** **:**:*: .*:*** :**.*** *  :*:*:*:********:*:*:*****:*.**

MG09531.1 	IPSLQSAVDHMMLYQETVQPLSDLRYLLENYRTGSFIPKVVTYENYYNRVDD~QTFGVDL
NCU09537.1	IPSLQSTVDNMMLYQETVQPLGDLRYLLENYRTGSFVPKVVTYENYYNKVDE~QTFGVDL
FG07433.1 	IPSLQSTVDQMMLFQETIQPQNDLRYLVETYRTGSFVPKVVVYENYYNKVDE~QTFGIDL
AN5677.1  	IPNLQSTVDHMMLYQETIQPLGDLRYLLENYRTGGFIPKVQAYENYYGSVGD1QIFGVDL
          	**.***:**:***:***:** .*****:*.****.*:*** .*****. *.: * **:**

MG09531.1 	EARARADKKRVPIIITTILTYLDHH~YPDLEGDEARRGVWLVEVPLSQTHRLRNRVNNGR
NCU09537.1	EARARADKKRVPIIVTTLLTYLDHH~YPDLEGDEARRGVWLHEVSLKDTHKLRAKVNNGK
FG07433.1 	EARARADKKRVPMIVTTLLTFLDHH~YPDLEGDEARRGVWLLEVPLSQSHSLRAKVNDGK
AN5677.1  	EARARADRKRVPVIVTTLLTYLDNC1YPELEGDESRRAIWLYDVPLGATHHLRHALNNRK
          	*******:****:*:**:**:**:  **:*****:**.:** :*.*  :* **  :*: :

MG09531.1 	VFSSDVFADFDIPTVASLLKLYLLELPD1SLVSSHV~YEIIRTIYQQPATEGSENSRIAI
NCU09537.1	PPSLEVFAEFDVPTVASLLKLYLLELPD1SLVSSHV~YEIIRTIYNT-TQDSSEDARIPV
FG07433.1 	PVSPDVFDDFDIPTIASLLKIYLLELPD1SLVSSHV~YEIIRTIYSTPSTDADESSRIAA
AN5677.1  	GDFFEVLQKYEIPVVASVLKLYLLELP-~------V1YEIIKTIYSTTANETTEEGRVKV
          	    :*: .:::*.:**:**:******        * ****:***. .: :  *..*:  

MG09531.1 	LQQTLSQLRLTNIATLDACMNHFTRLIDLTSADEAYVTALATILAPCILRPKVETSLTME
NCU09537.1	LQQTLSQLRLTNIATLDACMNHFTRLIELTSADEEYVAKLATTLAPCILRPRTETSLTME
FG07433.1 	LQSTLSQLRLTNIATLDACMNHFTRLIDLTSADETYVASLASVLAPCILRPRTETSLTME
AN5677.1  	LQSTLGQLRLNNIATLDAIMTHFTRLIDLTSADETYVSALAQSLAPCILRPRSENSLTMD
          	**.**.****.******* *.******:****** **: **  ********: *.****:

MG09531.1 	EKHAYRLIRDLFQHKDPIFSELKRMSTLSHSASVGSRGSTPAATDAPSDSKNDRRPRAIS
NCU09537.1	EKHAYRLIRDLFAHKDAIFSELKRMSTLGASATIS---------------GNNNRPRAIS
FG07433.1 	EKHAYRLVRDLFAHKDAIFSALKRMSMVTHSTSV----------------GSNNRPRAIS
AN5677.1  	ERHSYRLIRDLFAHKDTIFGELKRQS----SGLVG----------------SAPRPRAIS
          	*:*:***:**** ***.**. *** *    *  :.                .  ******

MG09531.1 	TDESNRRANMEERNRLVLEKAVGSRSRATSPAPGPRGHRRDRSSGGPETRFPVSPT----
NCU09537.1	TDESNRRANMEERNRLILEKANGGRSRATSPAPGPRAHRRDRSVGGPETRFPISPSGITS
FG07433.1 	TDESNRKALMEERNRALLEKASASRGRDKSPAPGPRGHRRDRSTGGPETRFPIASP--TA
AN5677.1  	TDESNRRAAMEARNRAIMD-----RSRAHSPAP-PRKHRRDRSSGPEPGRFPINVSGSKT
          	******:* ** *** :::     *.*  **** ** ****** *    ***:  .. .:

MG09531.1 	----ATHHPRQSVGPIMGI-KRSSLEVPGGEFPGSAGSNENSHAGSNGTPPTGLAESPVI
NCU09537.1	PTSSQQHGKRPSLGPVLP--KRSSLEVP--EEAGSAISHPADGNSNGAAPAEAGTPKSET
FG07433.1 	ASAVDRH--RTSLGGVI---KRQSLEVP--EPD-------------SAAPVNGEAEK-DK
AN5677.1  	PTTTRNSLEVPGSDSPTGAGAVSTAATTADELSTVNIHSPEPITNGTTAPSPATAESSVV
          	.::        . .     .  .:  ....*             ..  :*  . : ..  

MG09531.1 	TEMISDSN--------VNKRNSLGRSGARFVGGRRVTGPSVAGGAIPETS----------
NCU09537.1	ADPLADKRSSL-----VEKRNSLGRSGARISVGRRIP-------VVASTT----------
FG07433.1 	SDADSDKR---------DSRDSTGRTPTKFVGGKRVP-------VVPSTP----------
AN5677.1  	QEPPSEPTGSPSPPPETQSDDSATPTPTPHVEESSSTTSSLNRSSMSRSSGIHTRKPGLG
          	 :  ::  .: :.... :. :*   : :        . .:   . :. :..  : ... .

MG09531.1 	----------ATDG-AAGGDIERIRDSMHGVTLVDKPMDD
NCU09537.1	----------AASQQPSGTEKENLSGGHAPVTLVDAPMDD
FG07433.1 	----------STP--PSDSARG--------VQLEDAPMED
AN5677.1  	SRSSFPIIHGETNTDSKRSSIAESATELKGVTLEDKPMDD
          	: :: .   . :  ..              * * * **:*
```
