# Supplementary material for: Patterns of Intron Gain and Loss in Fungi
Source: PLoS Biol. 2004 Nov 30;2(12):e422. doi: 10.1371/journal.pbio.0020422 (PMC532390; doi:10.1371/journal.pbio.0020422)
Supplement: Table S1 — Also available at http://genes.mit.edu/NielsenEtAl/. (4.3 MB ZIP). [file pbio.0020422.st001.zip › NielsenEtAl/html/1167.html]

AN4647.1.NCU04228.1.MG06880.1.FG10257.1


```
 CLUSTAL W (1.82) Multiple Sequence Alignments - Introns Inserted


Sequence 1: NCU04228.1	329 aa
Sequence 2: MG06880.1	316 aa
Sequence 3: FG10257.1	305 aa
Sequence 4: AN4647.1	313 aa
Alignment Length: 341 aa
Number Identitical Residues: 152 aa
Alignment Score (without introns) 7627


MG06880.1 	MSSSTP-APGLSPTQLDFFHQNGYLIVPDALPPSTVSSLLAETSRLLESLDLSTHPMTKF
NCU04228.1	MASEPPTSDGLTPTQLAFFHQNGYLIIPRALPPTTVSRLLSETQKLLSDFDLTTHPLTRF
FG10257.1 	-MADTP---GLSPEQLEAFSRDGYLILPGALSSSTVKSLLDETHNLLENFSLDDHPLTRF
AN4647.1  	----MP---GLTPAQVTSFHENGYLVLPDYLTPQQINALITETTSLLTSFDLSTHPLTQF
          	     *   **:* *:  * .:***::*  *..  :. *: **  ** .:.*  **:*:*

MG06880.1 	RTGGE--DGKDHVGDDYFLGSGDKIRFFFEEDAVDDA-GNLTK-----DKSRAVNKIGHY
NCU04228.1	RTGGS--SGHDHVGDDYFLSSGDKIRFFFEEDAFDDASGNLIK-----PKDKAINKIGHY
FG10257.1 	STG----EKRDHVGDDYFLTSGDKVRFFFEEDAFDDE-GKLIK-----PKARAVNKIGHY
AN4647.1  	TTGDDEKDNKPHVGDDYFLTSGDKIRFFFEPDAFTPDPSNPTKSILSRPKEKAINKIGHA
          	 **..... : ******** ****:***** **.   ..:  *:  :  * :*:***** 

MG06880.1 	LHALSPPFAKLLGND-----GAVA--KASPAAVARDLGFRDPRCLQSMVICKQPEIGGAV
NCU04228.1	LHGLNPAFASLLATSPDDERGWVQDLKARPAAVARDLGFKDPRCLQSMVICKQPEIGGAV
FG10257.1 	LHALSPPFAHLLDHDTS---------KVSPPAVARSLGFKDPRCLQSMVICKQPEIGGAV
AN4647.1  	LHSLSKPFEAVSLNE-------------QNAEIARSLGFRDPRVLQSMVICKQPGIGGAV
          	**.*. .*  :   .               . :**.***:*** ********** *****

MG06880.1 	PPHQ~DSTFLYTNPPSAVGFWYALEDATLENGCLSFLPGSH--RWAPVERRLVRS---AG
NCU04228.1	PPHQ~DSTFLYTNPPSAVGFWYALEDATLENGCLSFLPGSH--LWAPIEKRLVRK---EG
FG10257.1 	PPHQ0DSTFLYTNPPSAVGFWYALEDATLENGCLSFLPGSH--RWAPVENRLVRK---EG
AN4647.1  	PPHK~DSEFLYTSPPSAVGFWFALQDAGVGNATLAMYKGSHKKRSGEVQRRFVRKYNEAG
          	***: ** ****.********:**:** : *. *::  ***..  . ::.*:**. .. *

MG06880.1 	DAAGTEMADNDGPKFPVGEQYGEDKKP---AGADADAYVPGEVKAGSLVLIHGNLLHKSE
NCU04228.1	AKEGTEMVDNDGPRFPPGEQYGEDKKPEEVKGREEEAYVPGEVKAGDLVLIHGNILHRSE
FG10257.1 	N-AGTEMVNNDGPRFPATDGYGKDEPE------DKHDYIPGEVKAGDLVLIHGNLLHKSE
AN4647.1  	QLCGTGFVSNEGEKFPRDLEVGEDEGE--------PEVEVLDVKAGSLVLIHGNVLHKSE
          	   ** :..*:* :**     *:*:                :****.*******:**:**

MG06880.1 	KNLSNKGRIIYTFHVIEGQD-AIYDERNWLQPPAEGFTELYR
NCU04228.1	RNLSQKGRIIYTFHVIEGEEGREYDERNWLQPPQEGFTRLYA
FG10257.1 	KNTSQKGRIIYTFHIIEGQD-RDYDAKNWLQPPEEGFTKLYA
AN4647.1  	KNTGDKSRFAYTFHVIEGAEGWEYDSRNWLQPPEGGFSRLYQ
          	:* .:*.*: ****:*** :.  ** :******  **:.**
```
