# Supplementary material for: Patterns of Intron Gain and Loss in Fungi
Source: PLoS Biol. 2004 Nov 30;2(12):e422. doi: 10.1371/journal.pbio.0020422 (PMC532390; doi:10.1371/journal.pbio.0020422)
Supplement: Table S1 — Also available at http://genes.mit.edu/NielsenEtAl/. (4.3 MB ZIP). [file pbio.0020422.st001.zip › NielsenEtAl/html/1168.html]

AN0252.1.NCU09119.1.MG09916.1.FG06875.1


```
 CLUSTAL W (1.82) Multiple Sequence Alignments - Introns Inserted


Sequence 1: NCU09119.1	299 aa
Sequence 2: MG09916.1	302 aa
Sequence 3: FG06875.1	301 aa
Sequence 4: AN0252.1	304 aa
Alignment Length: 310 aa
Number Identitical Residues: 177 aa
Alignment Score (without introns) 7892


MG09916.1 	MLSRAARPALRAGASVPSR2VVATPQNAANYATLREIEGRLKSIKNIEKITKTMKIVAST
NCU09119.1	MLSRAARPALRAGAAVSSR2AAAP--GAATFATLREIETRLKSIRNIEKITNTMKIVAST
FG06875.1 	MLSRAARPALRAAVAANAR2ATAVPSTAATYATLREIEDRLKSIRNIEKITNTMKIVAST
AN0252.1  	MLTRAVRPAVRAGAAAVTR2TAPP--NAANFATLREIEGRLKSIKNIKKITNTMKVIAST
          	**:**.***:**..:. :* ...    **.:******* *****:**:***:***::***

MG09916.1 	KLNRAQRAMTDSRSYGQTSNAVFESAETKPLEAEGKKELYVICSSDKGLCGGIHSGLSRY
NCU09119.1	KLNRAQRAMTESRGYGATSNEVFTSAETKPLEAEGKKKLVVVCSSDKGLCGGVHSGLARF
FG06875.1 	KLNRAQRAMNNSRTYGQTSNEVYESAETKALETEEKKTLIIVCSSDKGLCGGIHSGLSRY
AN0252.1  	RLTRAQKAMDDSRAYGQTSNTLFEQAETKALED--KKTLLVVASSDKGLCGGIHSGLSKA
          	:*.***:** :** ** *** :: .****.**   ** * ::.*********:****:: 

MG09916.1 	VRKMSAEKPGTFDLVIVGEKCKAQLSRTNAKDIQISFAGVGKDIPTFADASAIADQIVRL
NCU09119.1	IRRRAATEP-EFDIVIIGEKAKAQLSRTNAKDIVLNFSGVGKDIPTFVEASAIADQITQL
FG06875.1 	IRRLSANGE-KFDLVLVGEKAKAQLSRTNAKSIQLTFAGIGKDVPTFADAQAIADQVIQL
AN0252.1  	ARRALEANP-NSDLVVLGEKAKAQLSRTNPNAIVLSFANVCKDIPTFADAQAVADQIALL
          	 *:         *:*::***.********.: * :.*:.: **:***.:*.*:***:  *

MG09916.1 	PGEYSSIKILYNKFINAQSYEATPIEAFSEEAIAASP1NFSAFEVEEGILPNLREYALAN
NCU09119.1	KGDYSSVEIVYNKFINATSYEPTVIEAFSEDAILASP1NFSAFEVDEEVLGNLREYALAN
FG06875.1 	PTEYTDVKILYNAFVNAQTYEASLIEAFSEEAIQQSP1NFSAFEVDDEVLGSLREYGLAN
AN0252.1  	PTDYASVKVIYNKFVNAQSYEPDTVEAYSEEAITQSP1NFSAYEADEETLSNLREYALAN
          	  :*:.::::** *:** :**.  :**:**:**  ** ****:*.::  * .****.***

MG09916.1 	SLYWALAEGHACEQS~ARRNAMD0--------~NASKNAGEMITK~YQILFNRTRQAVIT
NCU09119.1	SLYWALCEGHACEIS~ARRNAMD0--------~NASKNAGEMISK~YQILFNRTRQAVIT
FG06875.1 	SLYWALAEGHACEQS~ARRNAMD0--------~NASKNAGEMINK~YQILFNRTRQAVIT
AN0252.1  	SLFWAMAEGHACEIS0ARRNAME~ASILEERV0NASKNAGEMIDK2FQILYNRQRQAAIT
          	**:**:.****** * ******: ::  ..   ********** * :***:** ***.**

MG09916.1 	GELVEIITGATASADM
NCU09119.1	GELVEIITGATASADM
FG06875.1 	GELVEIITGATASADM
AN0252.1  	GELVEIITGAAASAE-
          	**********:***:
```
