# Supplementary material for: Patterns of Intron Gain and Loss in Fungi
Source: PLoS Biol. 2004 Nov 30;2(12):e422. doi: 10.1371/journal.pbio.0020422 (PMC532390; doi:10.1371/journal.pbio.0020422)
Supplement: Table S1 — Also available at http://genes.mit.edu/NielsenEtAl/. (4.3 MB ZIP). [file pbio.0020422.st001.zip › NielsenEtAl/html/1169.html]

AN8713.1.NCU07440.1.MG02804.1.FG08658.1


```
 CLUSTAL W (1.82) Multiple Sequence Alignments - Introns Inserted


Sequence 1: MG02804.1	960 aa
Sequence 2: FG08658.1	912 aa
Sequence 3: NCU07440.1	876 aa
Sequence 4: AN8713.1	942 aa
Alignment Length: 1006 aa
Number Identitical Residues: 376 aa
Alignment Score (without introns) 21278


MG02804.1 	MSAAKAPAQPPPVKLSLPL~EYQQSVFQELRADDQLIIIARGLGLMRLVTNLLHSYDAAG
NCU07440.1	-------------------~----------------------------------------
FG08658.1 	MSANNAPQ---PVKLSLPL0EYQQSLFQELRAEDELVVIARGLGLMRLVNNLLHSYDAAG
AN8713.1  	---MPPERQNVPVKLSLPL0QFQQDIFTELRGEDELVILARGLGLLRLITNLLHFYDAAG
          	     .  .  . . : .  . ...   .  ....    : . .     ..     .::.

MG02804.1 	NNLVLVVNADEREIGWIGEALAEHAAISASPLARGLTVVNT~DFTNVGQRERLYAGGGIF
NCU07440.1	----------------------------MSPKARGLTVVNT~DFTSVGTREKMYAQGGIF
FG08658.1 	NNLIVVVGADDRENGWIGEALAEHAAISMSPKARGLTVVNT~DSQSVGAREKMYTKGGIF
AN8713.1  	NNLVLLVGANDRENEWIG---------------------EV1YPSSVVPDVKLLTR----
          	..     .:.. .    .                     :.    .*    :: :     

MG02804.1 	SVTSRILVVDLLTGLLDPVSITGIVLLHADK~VVATSLEAFILRIYRQKNKVGFLKALSD
NCU07440.1	SITSRILVVDLLTNLLNPETITGMLVLHADR~IVATSLEAFIFRIYRQKNKVGFLKAFSD
FG08658.1 	SITSRILVVDLLTSLLNPESITGLVVLHADR~VIATSLEAFILRVYRQKNKIGFLKAFSD
AN8713.1  	------------SELLDPERVTGLVVLHADK2IVATSTEAFIIRIYRNANKSGFLKAFSD
          	            : **:*  :**:::****: ::*** ****:*:**: ** *****:**

MG02804.1 	NPEAFTYGFAPLATMMRNMFLRKASLWPRFHISVAKSLEGKKTAEVVELEVQMTEAMNTI
NCU07440.1	NPDPFTVGFSPLATMMRNMFLRKASLWPRFHVQVAQSLEGKKKAEVIELEVPMTDSMREI
FG08658.1 	NPDPFTTGFSPLATMMRNLFLRKASLWPRFHVTVAQSLEGKKKAEVIELEVPMTDSMRDI
AN8713.1  	SPEPFTTGFAPLANSLRNLFLRKASLWPRFHVTVAESLEGHRKAEVIELEVPMSDKMREI
          	.*:.** **:***. :**:************: **:****::.***:**** *:: *. *

MG02804.1 	QTAIME~CVEVSIHELKKGNSGQLLDMEDWNLDSALTQNFDVSIRRQLNPNWHRVSWKTK
NCU07440.1	QTAIME~CVEISIHELKKENTG--LEMEDWNLDSALTRNFDRMVRRQLEPNWHRVSWKTK
FG08658.1 	QTAIMD2---------------------------ALLKNFDVIVRRQLDPNWHRVSWKTK
AN8713.1  	QNAVLE~CVELCIGELKKANTG--LDMADWTLDSALHRSFDISIRRQLDPMWHRVSFRTK
          	*.*::: . . .  . .. .:.   .  . . .:** :.**  :****:* *****::**

MG02804.1 	QIVGDLTVLRGMLH~SVVSLDAVSFLQLLDTIHAAHKPPPGSTRQTQSPWLFLDAAQTIF
NCU07440.1	QIAGDLTVLRGMLQ~SLLALDAVSFLQQLDTIHAAHKPAPGTTRQTESPWLFSDAAQTIF
FG08658.1 	QIVNDLTVLRGMLN~SILSYDAVSFLQHLDTIHAAHSPPPGSTRQNQSPWLFLDAAQTIF
AN8713.1  	QIVSDLSDLRAILH2ALLTYDAVSFVKYLDTIVTAHSPPPGSTRHNYSPWLFLDAAHVLF
          	**..**: **.:*: :::: *****:: **** :**.*.**:**:. ***** ***:.:*

MG02804.1 	ETAKKRVYAASNTRSVTSGTEIGTNASLDSLQPVLEEQPKWSVLADVLTEIERDTYFEPT
NCU07440.1	ETARQRVYSSK--------QKAGPNSTIESLKPVLEEQPKWAVLADVLEEIDRDLYFEPA
FG08658.1 	DTARRRVYSAS-------ARDAAREDNIDSLRPVLEELPKWALLAEVLEEIDRDLYFEPP
AN8713.1  	QTAKSRVYEGKIG---NELSRSSMTSLPTTLRPVLEEQPKWDVLAEILEEIEMDAYHNPA
          	:**: *** ..     ..    .      :*:***** *** :**::* **: * *.:*.

MG02804.1 	SRGDSNGTILIMCSDTNTCRQLRDFLQFMYVKPRSERKLDKSAAIKGEDDEEPSAAYLMR
NCU07440.1	VRDDSNGTILVMCADTDTCRQLRDYLQTMHIRPRTAKKVEE---VYDPEEDRPSGAFLMR
FG08658.1 	VRDDSNGTILVMCSNTDTCRQLRDFLQTMHVKPKTEKRVSE----DEEDEDKRSAAFMMR
AN8713.1  	STGESNNTVLIMCTDQRTCCQLREYLGTMNTKVEDERQEVADG-SEDKQEKKRSGEVMLR
          	  .:**.*:*:**::  ** ***::*  *  : .  ::   ..     ::.. *.  ::*

MG02804.1 	RKLRNYLKWKREFAQVNATLFA----ENQRALSNATDSRG------SGRAPPNKRRRVRG
NCU07440.1	RKLRNYLNWKREFAQVNATLFS----ENQKALSGAVDPRLPQAR-GRGGAPANKRRRVRG
FG08658.1 	RRLRNYLKWKRQFAQISATLFS----ENQKALNGATDTRPGFGGLRGGKAPANKRRRMRG
AN8713.1  	RKLREYVNWKRSLVNVNKNLTAKPVNEEPRTGSGRDSPRPTTQQ---GRAPPNKRRRVRG
          	*:**:*::***.:.::. .* :.. .*: :: ..  ..*        * **.*****:**

MG02804.1 	GGS-VNVGGARADNGSINQYFEKPGEVAELMASVQLNGEEEE-QAEQKVDVMAVPDALEN
NCU07440.1	GGGGVGSNPSRHENGSIVQHFEKPNEVADLMSEIQITEDDAGGQAAEEIITFATADPLED
FG08658.1 	GGN-AGTSMGRAENGSIMQYIEKPREVADLMAEVQITEEEAQ-QKEE-----VVSDPLDN
AN8713.1  	GAA--SVAAPRQPNSSVQADTEPSEQMSVLLDVIQPTEVEESLKEE------IIIDDLED
          	*.   .    *  *.*:    * . ::: *:  :* .  :   :           * *::

MG02804.1 	MDDYYELYEMQDLVVVHAYDGDQDEHVLEEVKPRYVVMYEPDASFIRRVEVYRSSHNDRN
NCU07440.1	MDDYYQLYDMQDLVVIHAYEGDQDEHVLEEVKPKYIIMYEPDASFIRRVEVYRSSHNDRN
FG08658.1 	MEEYFKMYDMQDLVVVHAYDGDQDEHVLEETKPRYIIMYEPDAAFIRRVEVYRSSHNDRN
AN8713.1  	MGDIYELYDMDDLVMVHPFDGDMDEHILEEVRPRYIIMYEPDPAFIRRVEVYRSSHVGRD
          	* : :::*:*:***::*.::** ***:***.:*:*::*****.:************ .*:

MG02804.1 	VRVYFMYYGGSVEEQRYLSTIRREKDSFTKLLKERA~SMAITITTDPHGAPDDPQEAFLR
NCU07440.1	VRVYFLYYGGSVEEQRYLSSVRREKDAFTKLIRERA~SMSIVMTTDSHG-VEDPESAFLR
FG08658.1 	VRVYFMYYGGSVEEQKYLSSVRREKDAFTKVIKERA~SMSLVMTVDPT---EDPEEAFLR
AN8713.1  	VRVYFMYYGGSVEEQRYLSAVRREKDAFTKLIKEKS0NMAVTLTHDKS--AEDPQEQFLR
          	*****:*********:***::*****:***:::*:: .*::.:* *     :**:. ***

MG02804.1 	TVNTRIAGGGRMTTKATAEPPRVVVDVREFRSSLPSLIHGRSLVVVPCMLTVGDYILSPN
NCU07440.1	TINTRIAGGGKLAK-ATAQPPRVVVDVREFRSSLPSLLHGRSMVIVPCMLTVGDYILSPN
FG08658.1 	TVNTRIAGGGRLA--ATAEPPRVVIDVREFRSSLPSLLHGRSMVIVPCMLTVGDYILSPN
AN8713.1  	TVNTRIAGGGRLT--ATASPPRVVVDVREFRSALPSLLHGNNMVIVPCQITVGDYILTPD
          	*:********:::  ***.*****:*******:****:**..:*:*** :*******:*:

MG02804.1 	ICIERKSISDLISSFKDGRLFGQCESMFQHYKNPMLLIEFDQNKSFTLEPFADLSG----
NCU07440.1	ICVERKSVSDLISSFKDGRLYAQCETMFQHYRNPMLLIEFDQNKSFTLEPFADLSG----
FG08658.1 	ICVERKSISDLISSFKDGRLYSQAETMFQYYKNVMLLIEFDQNKSFTLEPFADLSG----
AN8713.1  	ICVERKSVRDLISSLRNGRLYNQAETMTQHYKSPLLLIEFDENKSFTFDAFTSATTPGTT
          	**:****: *****:::***: *.*:* *:*:. :******:*****::.*:. : ..::

MG02804.1 	-----------------TLNSIRPSAQ-PSDLQSRLVLLTLAFPKLRIIWSSSPYETAEI
NCU07440.1	-----------------SLRSVNPENAGANDLQSKIVLLTLAFPKLRIIWSSSPYETAEI
FG08658.1 	-----------------SLNSVAPTNM-SSDLQSKLVLLTIAFPKLRIIWSSSPYQTAEI
AN8713.1  	FLTDFGFSSSGTVTTSLSSSSALINPSAPKSAQHLLVLLTLAFPRLKIIWSSSPYQTAEI
          	  :. . :::.: ::: :  *      .... *  :****:***:*:********:****

MG02804.1 	FERLKAQEPEPDPIAAVRAGLDGSTG-----------VGDDQ-PFNTEPQEMLGIVPGIT
NCU07440.1	FERLKALEEEPDPVAAVRAGLGEGESPEDGVNEGKGAVNGGS-TFNMEAQEMLGKVPGVT
FG08658.1 	FETLKTQEEEPDPIAAVRAGLDKDT------------RAEEQ-AFNQEPQDMLAIVPGVT
AN8713.1  	FAELKKNAPEPDPVRAVQIGLDVNISDDLGSGDLMTASGIEHRTFNLLPQEMLRAVPGVS
          	*  **    ****: **: **. . . . . ..   :      .**  .*:**  ***::

MG02804.1 	PKNIKAITAQVDNLRDIANMPLPELEVVAGKEVSRQVYGFFNKDVIEDVDDD
NCU07440.1	PKNIRNITAEAENVREVANMEVEELGRLVGREAAGKIVEFFKKDVLEDYSG-
FG08658.1 	PQNIKNLVLKTESIREIANMSVQELTPLVGAASGRQIHGFFTRNVMEEDD--
AN8713.1  	PQVLERLILETGNISEIANMSVEELDPFMGREAARQVVGFFRKSVFDGD---
          	*: :. :  :. .: ::*** : **  . *   . ::  ** :.*::
```
