# Supplementary material for: Patterns of Intron Gain and Loss in Fungi
Source: PLoS Biol. 2004 Nov 30;2(12):e422. doi: 10.1371/journal.pbio.0020422 (PMC532390; doi:10.1371/journal.pbio.0020422)
Supplement: Table S1 — Also available at http://genes.mit.edu/NielsenEtAl/. (4.3 MB ZIP). [file pbio.0020422.st001.zip › NielsenEtAl/html/117.html]

AN6591.1.NCU04104.1.MG03994.1.FG07169.1


```
 CLUSTAL W (1.82) Multiple Sequence Alignments - Introns Inserted


Sequence 1: NCU04104.1	959 aa
Sequence 2: MG03994.1	1036 aa
Sequence 3: FG07169.1	958 aa
Sequence 4: AN6591.1	961 aa
Alignment Length: 1045 aa
Number Identitical Residues: 353 aa
Alignment Score (without introns) 20901


MG03994.1 	MFEQTCRRGRD1GSHAIKTKLVGS2KGQGSEVWEGIQAD1RGKKAKSGLR0FCRQRKLAW
NCU04104.1	-----------~------------~--------------~----------~---------
FG07169.1 	-----------~------------~--------------~----------~---------
AN6591.1  	-----------~------------~--------------~----------~---------
          	                                                            

MG03994.1 	PTVSAKPDLNYAIGPS2HKSSPGKMAVVIGEVAQLLDATLDPQHHKKA1EAALKIEERKP
NCU04104.1	----------------~-------MAANLDHLAQLLQATLDARHHRKA1ETALKEEAKKP
FG07169.1 	----------------~-------MAADIGQIAQLLDATLDPTEHRKA1ESALKQEAAKP
AN6591.1  	----------------~--------MADLQSIAQLLAASSDRTQTKQA1EAALRQQESNP
          	                          . :  :**** *: *  . ::* *:**: :  :*

MG03994.1 	QFSLTLLQIVNSDALPSKTRLAAALCFKNFIRGNYV0DEDGKYKLPEDEVATLKQELVGL
NCU04104.1	KYSLSLLSIVANDAQPSNIRLAAALAFKNFIRHNYV~DEEGNYKLPADEVATIKQELVGL
FG07169.1 	QYSLSLLNIVNSDTLPLKTRLAAALAFKNFIRTSYV0DEEGNYKLPQDEVQVIKERLIGL
AN6591.1  	NFPISLLQITASDSYPLGTRLSSAILFKNVIRRNWT~DEDGNYKLPLEVVGTLKQELINL
          	::.::**.*. .*: *   **::*: ***.** .:. **:*:**** : * .:*:.*:.*

MG03994.1 	MISSPPNIQAQLGDAISIIADSDFWERWQTLIP0DLVSRLSTSDFKITNGVLEVAHSIFV
NCU04104.1	MISSPPTIQTQLGEAISIIADSDFWERWDTLTQ~DLVSRLSTTDPKVTNGVLEVAHSIFA
FG07169.1 	MISSPANIQAQLGDAISVIADSDFWRRWDTLTQ0ELVSRFSATDPKVNVGVLEVAHSIFA
AN6591.1  	MISVPQVLQTQLGEAVSVIADSDFWERWDTLVN0DLVSKLQPDNPSVNIGVLQVAHSIFK
          	*** *  :*:***:*:*:*******.**:**   :***::.. : .:. ***:****** 

MG03994.1 	RWRPLFSSNELYTEINHVLSHFGEPFLKLLD~STHQRIEAAKGDATQLKGWLQTMDLLVK
NCU04104.1	RWRPLFSSNALNIEVNHVVNTFGDSFIQMLG~VADQQIEANKTNEKALKGWLETMSLLTR
FG07169.1 	RWRPLFRTDELYMEINHVIETFGQAFLELLV0TTDKKIAENNDKKDVLRGWFETLDLQIK
AN6591.1  	RWRPLFRSDDLYIEINHVLERFGTPFLTLFQ0GLDTYLETNKSNKDQLTQGFTQLNLMVK
          	****** :: *  *:***:. ** .*: ::    .  :   : .   *   :  :.*  :

MG03994.1 	ILFDLSCQDLPPIIESNIASLCTLLQTYLSYSNTLLD-GDDEEETVIEMVKSDICSVLTL
NCU04104.1	IFFDLSCQDLPPIIETNLQPITMVLHKYLSYANPLFD-DEEDEATPIEILKSDICDALHL
FG07169.1 	ILHDMSCHDLPPIFDENLGSISELLHKYLTYSNPLLETDDDDETSIVDTVKADICEVLEL
AN6591.1  	LVYDLSCHDLPPMFEENMSGLAQILLKYLTYDNQLLHTDDDAESGQLEYVRAGIFEVLTL
          	:..*:**:****::: *:  :  :* .**:* * *:.:.:: *   :: :::.* ..* *

MG03994.1 	YFSKFDDDFGNTAQEFIPAVWHLLSSIGMEKRYDGLVSKALQFLTTVAGNPRHAPHFNSE
NCU04104.1	FVTKYDDDFGGYVQDFTSNVWNVLSSVGPQKRYDVLVSKALHFLTAVASVHRHAQIFNNE
FG07169.1 	FTVKFDEDFSKYCQPFIEKAWNLLSSTGPETKYDVIVSKALHFLTAIASSAQHSGIFNSE
AN6591.1  	YVQKYGDEFQPYIQQFVESSWNFLTTIGQETKYDILVSRALKFLTSIAGMPQHAQIFQAE
          	:  *:.::*    * *    *:.*:: * :.:** :**:**:***::*.  :*:  *: *

MG03994.1 	TVIKEIVEKVVLPNISLRESDIEMFEDEPIEFIRRDLEGSDSDSRRRAATDFLRRLQEND
NCU04104.1	EILGTIVEKVILPNVTLRESDIELFEDEPIEFIRRDLEGSDTDSRRKAATDFLRKLLDDF
FG07169.1 	EVLTQIVEKVILPNVALRESDVELFEDEPIEFIRRDLEGSDTDSRRRSATDFLRKLQERF
AN6591.1  	STLAQVIEKVVLPNVSLRESDEELFEDEPIEFIRRDLEGSDSDTRRRAATDFLKQLNANF
          	  :  ::***:***::***** *:*****************:*:**::*****::*    

MG03994.1 	DKLVTQVVGQYINHY---LGQAD---WKSKDTAVYLYLSIAAKGAVTAARGVQTVNPHVN
NCU04104.1	EALVTQVVSKYINHYL-EMGKTD---WKAKDTAVYLFLAIAAKGAVTAAQGVKTVNSFVN
FG07169.1 	EAPVTTVVSKYISHYL-SQGSSD---WKAKDTAIYLFLSIAAKGAVTAAQGVKTVNPLVN
AN6591.1  	EASVTKAVLQYIEHYLNEYGKSPQLNWKAKDTATYLFIAIAAKGVATATHGVTTTNSLIS
          	:  ** .* :**.** .. *.: . .**:**** **:::*****..**::** *.*. :.

MG03994.1 	VVDFFQQHIAGDLIKDEGVEPISKVNAIKYLHNFRSQLTKEQWSGAIQPLIVNMASSNYV
NCU04104.1	VIDFFQQHIAADLVAT-GGEPIPKVDAIKFLYNFRSQLSKEQWGGAINPLIQNLASPNYV
FG07169.1 	VVEFFEQHIAQDLINSQGVEHISKVDAIKYLYTFRSQLSKEQWKVALGPLIQNLNSDNYV
AN6591.1  	ITDFFQKNLAADLVSGDGVHPILKVDAIKYLYLFRSLITKEQWQEVFPLLVNHLGSSNFV
          	: :**::::* **:  .* . * **:***:*: *** ::****  .:  *: :: * *:*

MG03994.1 	VYTYAVITVERVLFLTNEQGQHLFTRADIEPLAKDLLEHLFNLVEKDRSPTKMQENEFLM
NCU04104.1	VYTYAATTLERVLFLTDDQGQHILSRADIQPYAKDLLQHLFALVEKDTSAAKLQENEFLM
FG07169.1 	VYSYAAIAVERVLFLTDDAGNAMFPRADIEPFAKDLLTHLFKLIEKESSPAKLQENEFLM
AN6591.1  	VYTYAAIAVERVLYFTDNQGQPIVSPDTIRPLAKDLLEHIFSLIQKNPAPEKVQENEFIM
          	**:**. ::****::*:: *: :..   *.* ***** *:* *::*: :. *:*****:*

MG03994.1 	RCIMRVLIVIKDGAVP-LLDTVLDRLISITNVIKQNPSNPRFYYYHFEAVGALIR~YCAA
NCU04104.1	RCIMRVLIVIKDGVLECDIDNILDHLINITNVIKENPSNPRFYYFHFEAIGAIVR~YCSN
FG07169.1 	RCVMRILIVIKDGATP-LLDNVLTHLILITNVMKQNPSNPRFYYYHFEAIGALVR~YCAP
AN6591.1  	KCAMRVLIVIKEGVVP-ITDNVLAHLINITQIISGNPSNPRFYYYHFETLGAFIR2FAAP
          	:* **:*****:*.     *.:* :** **:::. *********:***::**::* :.: 

MG03994.1 	TDASKLEAKLWEPLSSILNEDVTE1FVPYVFQLFAALLESSPNTVAPNNFLNLLKPVLSH
NCU04104.1	APQVDLLSRLWAPFTYILNEDVTE~FVPYVFQIFTQLLDLNKSGSIPGDFKALIDAVLAP
FG07169.1 	SNAALFNEKLWSPFHQILVEDVTE1FMQYVFQILAQLLESSPSETISDNYKALLGPLLSP
AN6591.1  	SNPDKLEQALYPPFSAVLQADIAE1FVPYIFQLFAALLEANPSGTLPTYYHGLIAPILAP
          	:    :   *: *:  :*  *::* *: *:**::: **: . .   .  :  *: .:*: 

MG03994.1 	TVWETRGNVPGCARFLSAIVPKVAEGIVAEGHLEAILGIFQRLLASKKTEPNAFDILEAI
NCU04104.1	GPWETRGNIPPLAKFIAAIIPKATEEIVKENKLEPILSIFQSLLNGKKTDQNAFDILESV
FG07169.1 	TLWETRGNVPACTRLLSAVIPRASQAIQAENQLEPVLGIFQRLLNSKKSELLAFDILDSI
AN6591.1  	QVWESKGNIPALVRLLSSIIARGSQHILENNQLINTLGIFQKLLSSKTNEGYGFDLLEAV
          	  **::**:*  .:::::::.: :: *  :.:*   *.*** ** .*..:  .**:*:::

MG03994.1 	VGSFPA2SALDQYFGTIQSLLFKKFETDVPDSFKQRFVRFYHLVSARGVEAGFGADYFIK
NCU04104.1	ICSFPA2SVLEPYFGTILTLIFTKLQKNPSDSYKTRVASFYHLVSARSGEAGLGTDYFIK
FG07169.1 	IKTFEP2TGLEQYFATILRLIYTKLQGSPSDAFKLRFVRFYHLVSAR-LEAGYGADYFIK
AN6591.1  	IEHFPS2AALEPFFKDIMQIILTRLQNHKTESLTLRFVRFYHFMCAN-DAKGYSADFVIQ
          	:  * . : *: :*  *  :: .:::   .:: . *.. ***::.*.    * .:*:.*:

MG03994.1 	HAEALQAK~VFVPLYLNYVLPVTAGFARPVDRKLGVISYTKTLCDSTAFAETYAKGWGFT
NCU04104.1	HAETIQSG~VFTPFYLQVVIPTTREFARPSDRKLAVISYSKTLVESKAFAERYMKGWGFT
FG07169.1 	QSNTIDQG~VFAQVYPAFVLAETERLARPVDRKVAVVSLTKTLCDSQAFSQQFMKGWANS
AN6591.1  	VIDKVQEG2LYVQLYLNIILPESQKLARPMDRKTAVISFTKTLANSEAFAVKYKKGWGFT
          	  : ::   ::. .*   ::. :  :*** *** .*:* :*** :* **:  : ***. :

MG03994.1 	CNHLLELLSNPPKVTTGAGDEFITEADVDDIGFGVGYTPLNTCKRGPRDDFPEI-TNVQT
NCU04104.1	CNALLELLKNPPKVSAGAGDEILNEADVDDIGFGIGFTPLSTCKRPPRDEFPEI-TDVQQ
FG07169.1 	CRKLLSLLVNPPTVNVG-GDEVVAEADVDDIGFGMSFTALNTCKPLAKDDFPEI-LDVTK
AN6591.1  	CEALLKLLELPPLPASK--DDIIAEHDVEDMAFGVGFTALVTIRPQARDPWPDTGADLKL
          	*. **.**  **       *:.: * **:*:.**:.:*.* * :  .:* :*: . ::  

MG03994.1 	WVSEYMKSANQRTGGKLATFVQQRLEDASKAELAKYLS
NCU04104.1	WVGDFLKASDKAHNGLITKYASERLNDEAKAVLAPILM
FG07169.1 	WVKEYMVSANQRHGGAVEGFIGARLNPEEQEAIVKYIR
AN6591.1  	WVGKYLKEADQRHGGKISGFVQERLGEQAKAMLSSYIA
          	** .::  :::  .* :  :   **    :  :   :
```
