# Supplementary material for: Patterns of Intron Gain and Loss in Fungi
Source: PLoS Biol. 2004 Nov 30;2(12):e422. doi: 10.1371/journal.pbio.0020422 (PMC532390; doi:10.1371/journal.pbio.0020422)
Supplement: Table S1 — Also available at http://genes.mit.edu/NielsenEtAl/. (4.3 MB ZIP). [file pbio.0020422.st001.zip › NielsenEtAl/html/1170.html]

AN4956.1.NCU07982.1.MG06868.1.FG01086.1


```
 CLUSTAL W (1.82) Multiple Sequence Alignments - Introns Inserted


Sequence 1: NCU07982.1	610 aa
Sequence 2: MG06868.1	682 aa
Sequence 3: AN4956.1	697 aa
Sequence 4: FG01086.1	690 aa
Alignment Length: 715 aa
Number Identitical Residues: 383 aa
Alignment Score (without introns) 17761


MG06868.1 	-MLRTVGRKALRGSSKGCSRTISTLKPATATIAKPGSRTLS-----TPATATAT2APRTK
NCU07982.1	------------------------------------------------------~-----
FG01086.1 	MLRSRPTRQAIR--AFGHARARTTLPATQRTKAT-------------ATTSAAP2QTRPV
AN4956.1  	MMPLRPSKSALR--TLHYQRYIASGRRAFASAVSTPHRFSTQKRDQSTATATAS2TSRPR
          	        .:    :       ::   :  :  ..     :.. ..:.:::::.  . . 

MG06868.1 	PSASFN----ARRDPQPLVNPRSGEADES2----------~FIGKTGGEIFHEMMLRQNV
NCU07982.1	-------------------------MDES2----------~FIGKTGGEIFHEMMLRHGV
FG01086.1 	PSPAFNLEPESRRHVQPLVNRSNPEMDES2----------~FIGKSGGEIFHEMMLRHDV
AN4956.1  	PSPAFNQEP-SRNEVSPLQNRQLPELDDS~YGILILFVQI2FVGLSGGEIFHEMMLRLGV
          	.:.: . .. :    ..  .    . *:*  .      .  *:* :*********** .*

MG06868.1 	KHIF1GYPGGAILPVFDAIYNSKHIDFVLPKHEQGAGHMAEG~YARASGKPGVVLVTSGP
NCU07982.1	KHIF~GYPGGAILPVFDAIYNSPHFDFVLPRHEQGAGHMAEG~YARASGKPGVVLVTSGP
FG01086.1 	KHIF1GYPGGAILPVFDAIYNSKHFDFILPRHEQGAGHMAQG1YARATGKPGVVLVTSGP
AN4956.1  	KHVF1GYPGGAILPVFDAIYNSKHFDFILPRHEQGAGHMAEG~YARASGKPGVVLVTSGP
          	**:* ***************** *:**:**:*********:* ****:************

MG06868.1 	GATNVITPMADALADGTPLVVFSGQVVTSAIGSDAFQEADVIGISRSCTKWNVMVKSVDE
NCU07982.1	GATNVVTPMADALADGTPMVVFSGQVPTTAIGSDAFQEADVIGISRACTKWNVMVKSVAE
FG01086.1 	GATNVITPMQDALSDGTPIVVFTGQVVTSAIGSDAFQECDTVGISRSACKWNCMVTSIAE
AN4956.1  	GATNVITPMQDAFSDGTPMVVFCGQVVTTSIGTDSFQEADVVGISRACTKWNVMVKSVAE
          	*****:*** **::****:*** *** *::**:*:***.*.:****:. *** **.*: *

MG06868.1 	LPRRINEAFEIATSGRPGPVLVDLPKDVTASVLRRAIPTETSIP-SISAAARAVQEAGRK
NCU07982.1	LPRRINEAFEIATSGRPGPVLVDLPKDITAGILRRAIPTDTAIPTSPSAASRAAIELSRK
FG01086.1 	LPRRINEAFEIATSGRPGPVLIDLPKDITAGILRKAIPTQTRLPSIPSMASQTAKQVMEK
AN4956.1  	LPRRIQEAFEIATSGRPGPVLVDLPKDITAGILRKPIPMQSTLPSRPSAATLAAKELSEK
          	*****:***************:*****:**.:**:.** :: :*:  * *: :. :  .*

MG06868.1 	QLEHSIKRVADLVNIAKKPVIYAGQGVILSEGGVELLKALADKASIPVTTTLHGLGAFDE
NCU07982.1	QLDASIQRVAKLVNIAKKPVIYAGHGVVQSKGGPALLRALSEKASIPVTTTLHGLGAFDE
FG01086.1 	QLNASLSRAADLIKIAKQPIIYAGQGIIQSEGGTELLRELADKCSIPVTTTLQGLGAFDE
AN4956.1  	QLHSTINRVARLVNVAKKPVLYVGQGILENPDGPKLLKELADKACIPVTTTLQGLGGFDE
          	**. ::.*.* *:::**:*::*.*:*:: . .*  **: *::*..*******:***.***

MG06868.1 	LDEKALHMLGMHGSAYANMSMQEADLIIALGGRFDDRVTGSIPKFAPAAKLAAAEGRGGI
NCU07982.1	LDEKSLHMLGMHGAAYANMAVQEADLIICLGGRFDDRVTLNLNKFAPAAKAAAAEGRGGI
FG01086.1 	RDEKSLQMLGMHGMAYANMSMQEADLIIALGARFDDRVVLNTSKFAPNAKAAAAEKRGGI
AN4956.1  	LDPKSLHMLGMHGSAYANLAMQEADLIIALGARFDDRVTGSIAKFAPQAKLAASENRGGI
          	 * *:*:****** ****:::*******.**.******. .  **** ** **:* ****

MG06868.1 	VHFEIMPKNINKVVQATEAIEGDVASNLKLLLPKIEQRSMTDRKEWFDQIKEWKEKWPLS
NCU07982.1	VHFEILPKNINKVVQATEAVEGDVATNIELLIPQVDAKTMADRKEWFGKINEWKSKWPLS
FG01086.1 	IHFDILPKNINKVIQATEAIEGDVATNLKMLMPMLTPTSMEQRSGWFNKISEWKAKWPLS
AN4956.1  	VHFEIMPKNINKVVQANEAVEGDCAENIRHLLPLVEP--VSERPEWFAQINDWKTRFPLS
          	:**:*:*******:**.**:*** * *:. *:* :    : :*  ** :*.:** ::***

MG06868.1 	HYERAER---SGLIKPQTLIEELSNLTADRKDMTYITTGVGQH~QMWTAQHFRWRHPRSM
NCU07982.1	DYERAER---TGLIKPQTLIEELSKLTEGRKENTYIATGVGQH~QMWTAQHFRWRHPRTM
FG01086.1 	NYEHSDAPGGSGLIKPQTLIEELSDLTSKIDKKTIISTGVGQH0QMWVAQHYLWREPRSF
AN4956.1  	LYEKQTP---DGPIKPQALIEKLSDLTAHMKDRTIITTGVGQH~QMWAAQHFRWRRPRSM
          	 **:       * ****:***:**.**   .. * *:****** ***.***: **.**::

MG06868.1 	ITSGGLG~TMGYGLPAAIGAKVARPDALVIDIDGDASFNMTLTELSTAAQFNIGVKVIVL
NCU07982.1	ITSGGLG~TMGFGLPAAIGAKVAKPDALVIDIDGDASFGMTLTELSTAAQFNIGVKVIVL
FG01086.1 	VTSGGLG1TMGFGLPSAIGIAVAKPDALVIDIDGDASFNMTLTELSTAAQFNIGVKVIIL
AN4956.1  	ITSGGLG~TMGYGLPAAIGAKVARPDCLVIDIDGDASFNMTLTELTTAAQFNIGIKVLLL
          	:****** ***:***:***  **:**.***********.******:********:**::*

MG06868.1 	NNEEQGMVTQWQNLFYEDRYSHTHQRNPDFMKLADAMDVQHRRVSKPDDVVDALTWLINT
NCU07982.1	NNEEQGMVTQWQNLFYDDRYSHTHQKNPDFVKLADAMGVQSKRIIKPEEVVEGLKWLIDS
FG01086.1 	NNEEQGMVTQWQNLFYEDRYAHTHQKNPDFIKLADAMGVQARKVTKPDDVRASLEWLINT
AN4956.1  	NNEEQGMVTQWQNLFYEDRYSHTHQKNPDFVRLAEAMGVAAERCTKPSEVESKLKWLIES
          	****************:***:****:****::**:**.*  .:  **.:*   * ***::

MG06868.1 	DGPALLEVMTDKKVPVLPMVPGGNGLHEFITFDAS1KDKQRRELMRAR-TNGLHG-----
NCU07982.1	EGPALLEVVTDKKVPVLPMVPAGCGLDEFITFDQE~RDRKRRELMVKR-TGGVHGL----
FG01086.1 	DGPALLEVVTDKKVPLLPMVPAGAGLHEFLVYDKA1FDYREGQGSTSANARANQWSSRFL
AN4956.1  	DGPALLEVFTDRKVPVLPMVPGGSGLHEFLVFDEA1KDRERRALMKKRNPNGV-------
          	:*******.**:***:*****.* **.**:.:*    * ..       .. .        

MG06868.1 	---
NCU07982.1	---
FG01086.1 	IKK
AN4956.1  	---
          	
```
