# Supplementary material for: Patterns of Intron Gain and Loss in Fungi
Source: PLoS Biol. 2004 Nov 30;2(12):e422. doi: 10.1371/journal.pbio.0020422 (PMC532390; doi:10.1371/journal.pbio.0020422)
Supplement: Table S1 — Also available at http://genes.mit.edu/NielsenEtAl/. (4.3 MB ZIP). [file pbio.0020422.st001.zip › NielsenEtAl/html/1171.html]

AN8183.1.NCU03794.1.MG07465.1.FG07379.1


```
 CLUSTAL W (1.82) Multiple Sequence Alignments - Introns Inserted


Sequence 1: NCU03794.1	899 aa
Sequence 2: FG07379.1	893 aa
Sequence 3: MG07465.1	900 aa
Sequence 4: AN8183.1	876 aa
Alignment Length: 943 aa
Number Identitical Residues: 492 aa
Alignment Score (without introns) 24174


MG07465.1 	MKTDFK0-------------------------FSNLLGTVYCRGNLLFSPDGTHLYSPVG
NCU03794.1	MKTDFK~-------------------------FSNLLGTVYCQGNLLFSPDGTHLFSPVG
FG07379.1 	MKTDFK0-------------------------FSNLLGTVYCQGNLLYSPDGTHLFSPVG
AN8183.1  	MKTDFK~VSTSSKRTDGFANWLWGSSKLTKKQFSNLLGTVYRKGNLLFTPDGNCLISPVG
          	******  ::::. :.. :.   .::. :...********* :****::***. * ****

MG07465.1 	NRVTVFNLVD2NKSYTLPFAHRKNIVRLDLTPRGNLLLSVDEDGHAILTNVVRRISIYHF
NCU03794.1	NRVTVFNLVD2NKSYTLPFSHRKNIARIGLTPQGNLLLSIDEDGQAILTNVPRRVVLYHF
FG07379.1 	NRVTVFNLVE2NKSYTLPFAHRKNIARIGLTPRGNLLLSIDEDGHAILTNVPRRISIYHF
AN8183.1  	NRVTVFDLVN2NTSYTLPFAHRTNIHQLDLTPRGNILLSVDEHGRAILTNLLRRVQIYNF
          	******:**: *.******:**.** ::.***:**:***:**.*:*****: **: :*:*

MG07465.1 	SFRTAVTALSFSPSGRHFAVGIGRRIEVWQVPQTPDSATGDDGLEFAPFVRHHSHAAHFD
NCU03794.1	SFKAPVTALSFSPSGRHFIVGLGRKIEVWHVPSTPD-ANADGELEFAPFVKHHTHVQHFD
FG07379.1 	SFRSSVTSLSFSPSGRHFAVGLGRRIEVWHVPSTPD-ASAEGELEFAPFVRYHSHTGHFD
AN8183.1  	SFKGRVSALKFSPSGRHFAVGVGRRLQIWHTPETPT-VGGDGEIDFAPFVLHRDLAGHFD
          	**:  *::*.******** **:**::::*:.*.**  . .:. ::***** ::  . ***

MG07465.1 	EVRHIEWSRDSRFFLSASKDLTARIWSLNQEEGFTPTVLSGHRQGVVGAWFSMDQET~IY
NCU03794.1	DVRHIEWSHDSRFFLTSSKDLTARIWSVDQEEGFTPTVLSGHRQGVVGAWFSKDQET~IY
FG07379.1 	TVTNIEWSSDSRFFLTTSKDLTARIWSLTPEEGFTPTVLSGHKQAVIGAWFSENQET~IY
AN8183.1  	EVQHIEWSSDSRFLLTASKDLTARVWSLDPEDGFEPTTLSGHRQGVRAAFFSADQES0IY
          	 * :**** ****:*::*******:**:  *:** **.****:*.* .*:** :**: **

MG07465.1 	TVSKDGAVFDWQYVGPQD--KEDDDMDGADESDLRWRIVKRHYFMQNNASLKCAAFHAES
NCU03794.1	TVSKDGAVFDWQYVAKPG--QDED-MVDDD--DLAWRIVNKHYFMQNSATVRCAAFHPES
FG07379.1 	TISKDGAVFDWQYVKPINRIEDEDKMQDDDDEDMRWRIVQRHYFMQGSAHVRCASFHPET
AN8183.1  	TVSRDCALFRWEYVSKKD----PDTMEDVA--EPRWRIVKKDFFQQPHATVKCAAFHAAS
          	*:*:* *:* *:**   .     * * .    :  ****::.:* *  * ::**:**. :

MG07465.1 	NLLVAGFSNGIFGLYEMPDFNLIHTLS~ISQNGIDFVSINKSGEWLAFGASKLGQLLVWE
NCU03794.1	NLLVAGFSNGIFGLYEMPDFNMIHTLS~ISQNEIDFVTINKSGEWLAFGASKLGQLLVWE
FG07379.1 	NLLVAGFSNGLFGLYEMPDFNMIHKLS~ISQNDIDFVTINKSGEWLAFGASKLGQLLVWE
AN8183.1  	NLLVVGFSNGLFGLYELPEFNTIHLLS2ISQSNIDVVSINKTGEWLAFGSSKFGQLLVWE
          	****.*****:*****:*:** ** ** ***. **.*:***:*******:**:*******

MG07465.1 	WQSESYILKQQGHFDSMNALAYSPDGKRIVTAADDGKLKVWDIESGFCIVTFTEHTSGVT
NCU03794.1	WQSESYILKQQGHFDSMNSLVYSPDGQRIVTVADDGKIKVWDTESGFCIVTFTEHTSGIT
FG07379.1 	WQSESYILKQQGHFDAMNSLVYSPDGQRIITCADDGKIKVWDIQSGFCIVTFTEHTSGVT
AN8183.1  	WQSESYILKQQGHLDSMNALAYSPDGQRIVTAADDGKIKVWDVKSGFCLVTFTEHTSGVT
          	*************:*:**:*.*****:**:* *****:**** :****:*********:*

MG07465.1 	ACQFAKKGNVLFTASLDGSIRAWDLIRYRNFRTFTAPTRLSFSCMAVDPSGEVVAAGSLD
NCU03794.1	ACEFSKKGNVLFTSSLDGSIRAWDLIRYRNFRTFTAPERLSFSCMAVDPSGEIVAAGSVD
FG07379.1 	ACEFAKKGNVLFTSSLDGSIRAWDLIRYRNFRTFTAPTRLSFSCMAVDPSGEVVAAGSLD
AN8183.1  	ACQFSKKGNVLFTSSLDGSVRAWDLIRYRNFRTFTAPSRLSFSSLAVDPSGEVICAGSPD
          	**:*:********:*****:***************** *****.:*******::.*** *

MG07465.1 	SFDVHIWSVQTGQLLDQLSGHEGPVSAVAFAPDGGLL~VSGSWDKTARIWSVFNRTQTSE
NCU03794.1	SFDIHIWSVQTGQLLDRLSGHEGPVSSLAFAPNGGLL~VSGSWDRTARIWSIFNRTQTSE
FG07379.1 	SFDIHIWSVQTGQLLDQLSGHEGPVSSLAFTPNGNSL~ISGSWDRTARIWSIFSRTQTSE
AN8183.1  	SFDIHVWSVQTGQLLDQLSGHEGPVSALAFAADGNHL0LT--------------------
          	***:*:**********:*********::**:.:*. * ::                    

MG07465.1 	PLQLQADVLSVAVRPDSSQLAVSTLDGQISFWSVTEAQQVSGVDGRRDVSGGRKITDRRT
NCU03794.1	PLQLNSDVLDIAFRPDSLQIAISTLDGNLSFWSVSEAEQQAGLDGRRDVSGGRKIGDRRT
FG07379.1 	PLQLQADVLDIAVRPDSLQLAISTLDGQLTFWSVTDAEQTSGVDGRRDVSGGRKLTDRRT
AN8183.1  	------------------------------FWSVADAVQVAGIDGRRDISGGRKITDRTT
          	                              ****::* * :*:*****:*****: ** *

MG07465.1 	AANAGGTKAFHTIQYSMDGSCLIAGGNSKYMCLYSTTTMVLLKKFTVSVNLSLSGTQEFL
NCU03794.1	AANVAGTKAFNTIRYSTDGSCLLAGGNSKYICLYSVTTMVLLKKYTVSVNLSIQGTQEFL
FG07379.1 	AANMAGTKSFNTIRYSTDGSCLLAGGNSKYICLYSVTTMVLLKKFTVSVNLSLSGTQEFL
AN8183.1  	AANAAGTKSFHCITYSADGSCILAAGNSKYICLYDVLTGSLVKKYTVSVNTSLDGTQEYL
          	*** .***:*: * ** ****::*.*****:***.. *  *:**:***** *:.****:*

MG07465.1 	NSKQLTEAGPQELLDDH-DASDREDRVNRSLPGSKRGGDPSARTTHAEVKVSGVSFAPDG
NCU03794.1	NSKLLTEAGPQGLLDEQGEASDFEDRIDRSLPGSKRG-DPSARRKNPEVRVNGVAFSPNG
FG07379.1 	NSKLLTEAGPAGELDDQ-EASDREDRVDSTLPGSKRG-DPSARKKVPEVRVTGIGFSPAG
AN8183.1  	NSRDLTEAGARGLIDETGEASDHEERIDRSLPGAKRG-DAGSRTTRPEVRVSCVDFSSTG
          	**: *****.   :*: .:*** *:*:: :***:*** *..:* . .**:*. : *:. *

MG07465.1 	AAFCAASTEGLLIYSLDHTVQFDPFDLNMEITPASTLAVLE------------NEKDYLK
NCU03794.1	SAFCAASTEGLLIYSLDTTIQFDPFDLNMEITPTSTLAVLE------------KEKDYLK
FG07379.1 	TAFCAASTEGLLIYSLDQDIQFDPFDLNMEITPASTLAVLE------------TEQDYLK
AN8183.1  	RSFCAASTEGLLIYSLDTEFLFDPFDLDISITPASILSTVEGAKKAAATGDANNDDTYLK
          	 :***************  . ******::.***:* *:.:*.:..::::..:..:. ***

MG07465.1 	ALVMAFRLNEAGLIKRVYQAIPHRDIPLVVEQFPPVYVARLLRFVAAQTEESPHIEFCLL
NCU03794.1	ALVMAFRLNEAGLIQRVFQAIPYTDIPLVVEQFPNVYVARLLRYVAAQTEQSPHVEFCLL
FG07379.1 	ALVMAFRLNEAGLIKRVFQAIPSHEIPLVVADLPTIYVSRLLRFVAAQTEESPHIEFCLV
AN8183.1  	ALVMAFRLNEAKLIRAVHEAIPPSDIAHVVRSVPTVYLPRLLRYVAHACEETPHLEFNLL
          	*********** **: *.:***  :*. ** ..* :*:.****:**   *::**:** *:

MG07465.1 	WIKALVDKHGKWLAANRAKVDVELRVVARAIARMRDEIRRLADENVYMVDYLLGQAENKP
NCU03794.1	WIKALVDKHGAWLSANRGKVDVELRVVARAVSKMRDEIRKLADENVYMVDYLLGQASAAK
FG07379.1 	WIKAIVDKHGAWLSANRAKADIELRVVARAVAKMRDEIRRLADENVYMVDYLLGQAQEKT
AN8183.1  	WIESLFSSHGRYLKDNAGSLGPELRAVQRALDDINENIKRLSQKNVHNLNYLLAKP--VL
          	**:::...** :*  * .. . ***.* **:  :.::*::*:::**: ::***.:.    

MG07465.1 	KAIEGTSIDDILKPLPAKGAN---AALMDANMAQEDESSDEDGWIGLD
NCU03794.1	ETNTTKTLALEWATTGSDEQPGAGGMSLNDVMQQDEGNASEDEWIGLV
FG07379.1 	EKKDVTALD-----SGKDVD-----VKLMAADGDEESDAGSDEWIGLD
AN8183.1  	TGRKAPALPKAIDTLDAAATN-------GDATMSDAGTDAEGEWHGLE
          	      ::     .                   .:     .. * **
```
