# Supplementary material for: Patterns of Intron Gain and Loss in Fungi
Source: PLoS Biol. 2004 Nov 30;2(12):e422. doi: 10.1371/journal.pbio.0020422 (PMC532390; doi:10.1371/journal.pbio.0020422)
Supplement: Table S1 — Also available at http://genes.mit.edu/NielsenEtAl/. (4.3 MB ZIP). [file pbio.0020422.st001.zip › NielsenEtAl/html/1172.html]

AN5925.1.NCU06365.1.MG06067.1.FG01945.1


```
 CLUSTAL W (1.82) Multiple Sequence Alignments - Introns Inserted


Sequence 1: NCU06365.1	1175 aa
Sequence 2: MG06067.1	1693 aa
Sequence 3: FG01945.1	1644 aa
Sequence 4: AN5925.1	1720 aa
Alignment Length: 1825 aa
Number Identitical Residues: 530 aa
Alignment Score (without introns) 28718


MG06067.1 	---------MADDTPLTS-R~ALSQAGSPKEDAETKATREELKQTAISEKPAAAEQEGQM
NCU06365.1	--------------------~--------------------------------------M
FG01945.1 	---------MADDAPDASPK~NEPLNVETKEDAETRATRRELKQSSISDPP---------
AN5925.1  	MAPEMRKEVLDVENGVTNQT1PADDSQTSDNDGGERPVRNKLKKTTITSAPNQRDDSSRA
          	 :..  ..    .   :.          .....   .   . ..:: :. .   ....  

MG06067.1 	AKAPKLSAVDTT----------------KPTDDALKEQVSSPKKKRAHDQLDENHEPSSS
NCU06365.1	ASSHHSANATTT----------------DPLKGLKRKRLS--------------------
FG01945.1 	-TSGPEDAANTS----------------DAPDKDMKEQITSPKKKRAHDQLDGSKEEEEN
AN5925.1  	SSRGRKRSYNSDEENPEDDSGHRRKRSRDSNTEEMADAAPAKETTAVTLKLDAQEEQPEQ
          	:.        : .......:.   . : ..      .  .: ...    . . . .  ..

MG06067.1 	ESAGSLSPKDGTNRTDRLEPEKKRARDELVEA~EAEDESG0ASTTESSKKTTEPTDTNEP
NCU06365.1	--------------------------------~-------~----------TEPEASTEP
FG01945.1 	DTNSVASSDSAKDRSLRLEPEKKRHRDEDTDL0QSGAASS~EEATKTSQTANSPTKKSQP
AN5925.1  	PQAQAQGSASGLKKKRSRDQLDKDEAKVEDKA~AKAEVKD~SSENRESAEKTAATATAEG
          	      .. .. . .   .  ..   .   .       ..  . .  :   . .  . : 

MG06067.1 	KDPSLSARLQKPAAGKDD~AAPTTTSASAFAKSGFAKLSGTASPFGTLGATGGASPFGSL
NCU06365.1	R-----------------~------HDAAFAGN---------------------------
FG01945.1 	Q-----------------~-----TSASAFAASGFGKLSSGSSPFASLGASQGSSPFASA
AN5925.1  	EPEK--KRHRDVLGEREP0APLPSPFANTSSVSPFGSIAASSSKQAEDAKPATSPAAFAS
          	.  .      .  .  .  :. .:.   : : .  .. :. ::  .  . .  :..  : 

MG06067.1 	GSSSNTASGFGALSSSASPSPFGALGASSGTIPAPPKLTFGPPSDGSSSASPFASLNGGA
NCU06365.1	------------------------------------------------------------
FG01945.1 	AAGKPSISSFASPPASTAAQPAAAPKLTFGS---------------SGGASPFAGLSTGT
AN5925.1  	SSLAAFAGSEQSPFGSLGASTPSVFKSSTESVPAGSDKPAATGFASAAKSSGFAGLGGGF
          	.:     ..  :  .: .... .    :  : .: .. . ..   .:. ::  :. . . 

MG06067.1 	SKPFGSAFGSGFGGGLGASKLTSFAKPGGVLKDDKPARPFGAPETDNEASENGESDTETG
NCU06365.1	------------------------------------------PSCDSQES----------
FG01945.1 	NGSPFGGSTFGSAFGAGKPLSSFAAPGAEAPKSEKPSKPFGAPDSDVEEEEDEDNEAEET
AN5925.1  	SG--FSGGFGAAAASTGGGLTSFAAPGGSALLGTSSAKPFG-AEADSDEGDDKDDEGETG
          	.    ..   . . . .    :  :  .    . ..: . . .. * :  .. ... .  

MG06067.1 	EDGVAQEESESEEKAKDEDGEADQTKPVSVEDKKKATKLQKV1VV~DDGEAGEATIFLAR
NCU06365.1	------------------------------------------~--~--------------
FG01945.1 	EQPAETERAASPEKESDE---------------KKKLKLQKI1EV~NDGEAGEATVVSVR
AN5925.1  	PAEFEQD--------------------------KTDERFYER~QI1ETGEEQEKTYFTCK
          	      .                          ..     .     . ..  . :     

MG06067.1 	AKVYHLDKESGAWKERGAGILKVNVPEATIDFDQSGAALTASFDAS-----------MLD
NCU06365.1	------------------------------------------------------------
FG01945.1 	AKMFYHDKEAG-WKERGAGMLKINVPQACVEYDDSGAVIPGSFDAS-----------ALE
AN5925.1  	AKLFHFSNKE--WRERGLGTFKVNVKVTDDVEDKKGARMIMRADGVGRVMLNTPLFKGMK
          	:.    ...     . . .  . .   :    ....:      .. .    .:.  .  .

MG06067.1 	AGDSDS---KTPKVVRLIMRQDHTLRVVINT~AVLAGTEFVR~REMMKAVGFLF~TALEG
NCU06365.1	-------------------------------~----------~-----------~-----
FG01945.1 	VDEEAAGGSQGHKVARLIMRQDQTHRVILNT~ALVAAMKFQE~KASLKSVGILF~TAFEG
AN5925.1  	VGDAAGNEPKSTKQIHLASLEDNRSVPLLLR0QSAREQPTVP0AAILHSEWDWE0HSFVY
          	 ..  .. ..  .       ..                          :       :   

MG06067.1 	PDARPVTLQVK0QALSQQKGWAI~LN1VGGGLAYLSRIIVKLRPKSHLLAYF1NKSGKRK
NCU06365.1	-----------~-----------~--~-------------------------~-------
FG01945.1 	EQSKPVSITMR0TTPNTKTWCDY1QV~QSSLAEFFDSSNEMTIHPEPSRPDL~NESGTTA
AN5925.1  	GRSVHQPQWGA~QMARQKTKKTA~SF~TGAGPAENRNHAERTSSQHNLTTGV~SGCALQV
          	  :   .          ..         ..                   .   . ..   

MG06067.1 	R----SQIADALA-----------------PGECNHGIDTPEKP-AAGKARRTAKSTSKA
NCU06365.1	------------------------------SGSCGPATTGPKK-----RRGRPPKLVSEP
FG01945.1 	KQSSSSQQGKRKR-----------------NVQDDPAVGSPTSSQSSNSKTRKGGKQGKG
AN5925.1  	QQSAEDTNAARQKRKLDETVSETLNSQENIDDNVAPVEEESAQSKIAGSMSATKQPGKRR
          	 .::..  .     . ..: :.: .:...   .       . ... :.          . 

MG06067.1 	SNAQQPDPASVQEECVIEWPDWFKILDKTYRALNLVVTFCCTRKHLATTFDTIKSTVESH
NCU06365.1	TIAESD----------IEWPAWFKELEKTHRALNLVYTFCSTRKHLATTFETIRSTVESH
FG01945.1 	RPSLNKEASSVVIPCVIEWPEEFKQIERTHRALNLVYTFCTTRKHLATTFDTIKSSVEGH
AN5925.1  	KSAASSERQQQPRKPSPPWPDHFKHLSRTHRALNLIYTFCCTRKHFATTFDNIKKAVQAQ
          	  : . .  .        **  ** :.:*:*****: *** ****:****:.*:.:*:.:

MG06067.1 	ISRQLLIEDVAAIATLRPDGMRFAYVDEVMLQLDARGAERDDTFRSSRNFLTS-QSRPPD
NCU06365.1	TKRSLTIEDVAAIVALRPESIQFAYVDELMLQLDARGAEKDEAFKTGRYAGKSSQSFVPD
FG01945.1 	IKRELRIDEIASMVALRPEGLYFAYVDETMLQLDVKGTEKDEIFRTGKSYRSQ--APAYD
AN5925.1  	TGTELTIEDIARVRVLIPRAVRFEYVNEARLDVLSAGEREVKGWGGAIDNGNG--DEMNG
          	   .* *:::* : .* * .: * **:*  *::   * .. . :  .    .       .

MG06067.1 	SSVGGFTGREGLQTRGQIENGD---VDLTGKEVLYVEFLDGDLKREVQCKKTGEPTRPTR
NCU06365.1	ASVGGMTGLEGLGDAGPDHTGEDEQESKSGREVLYFEFIDEDLKRQVQDKKTGVPTKPTR
FG01945.1 	ASVGGYTGLESLDKS---HNRD---LEPMGREVLFLEFIDGDLKRQVQGK-GGEPVKPNR
AN5925.1  	EADAKLDG---------------------VMYALLFEFLDGDLKKEKKSLTSGTRDK---
          	 : .   *                        .* .**:* ***:: :  . *   :   

MG06067.1 	KLRDEELKMPVYSQKQMANLIEKRNQRFTSAVNSFINRCVTESTDPVMALKSESERFIPV
NCU06365.1	RLRHEHVKMPVYSQKQMTALIEKRNTKFANAVNIFLNRCAAESLDPEMALRSESEPFVPR
FG01945.1 	KLRDEQLKMPVFSQKQMTTLIERRNQKFTNAINIFLNKCIEDGLEPLETLKEQTKSCIPV
AN5925.1  	---DEDLRMPVYSQKQMLGLIEKRNGKFADAVDAFLVRCEDEGVDAVERLEREKDGFIPV
          	   .*.::***:*****  ***:** :*:.*:: *: :*  :. :.   *. :..  :* 

MG06067.1 	P--TAATQPSPPPETSTIPESIPNERKSIPEIVQELKESPFYTGQIVPDGHRVFEPQEPV
NCU06365.1	P--STPRASTPQTKPSTLPASIPKERKGIPEIVQELKDSPWYTGQIVPDGHRVNEAQEPV
FG01945.1 	P--SAKEEFAPEKAAETIPESIPKERKTIPEIVQELRESPWYTGQIVPDGHRVFEQQDPV
AN5925.1  	LPDIGSKEGLADGLSSKVRGPIPKDRKTMAEIIEEIRCLDWYTGQIVPDGHRAFDAQPAI
          	 ..       .   ...:  .**::** :.**::*::   :***********. : * .:

MG06067.1 	FGDLNFLLSQDLVNALYNAKGITQFYAHQTEAINGLIDGHHVVVATSTSSGKSLIYQLPV
NCU06365.1	YGYLNFLLSQDLVNALYNTKGITQFYAHQADAINALHDGRHVVVSTSTSSGKSLIYQLPV
FG01945.1 	YGDLNFLLSQNLVNALYNAKGITQFFAHQSEALNSLHDGKHVVVSTSTSSGKSLIYQLPV
AN5925.1  	YGDLRFALSQDLVNALYNTKGITRFYSHQAEAINHLHDGKNVIVSTSTSSGKSLIYQVPM
          	:* *.* ***:*******:****:*::**::*:* * **::*:*:************:*:

MG06067.1 	LHALEKDHETRAMYIFPTKALAQDQKRSLKEMLAYMPGLEHLIVDTFDGDTPMTARNLIR
NCU06365.1	IHALEQDRNTRAMYIFPTKALAQDQKRSLKEVMSLMPGLEEMIVETFDGDTPMHDRNIIR
FG01945.1 	IRALEEDYNSRAIYIFPTKALAQDQKRSLKDMMRYMPGLEETMIETFDGDTPMTERNDIR
AN5925.1  	LHELEQDSDSRGMYIFPTKALAQDQKRSMQELLQYLNSLQGTMVETFDGDTPMANRNLIR
          	:: **:* ::*.:***************:::::  : .*:  :::********  ** **

MG06067.1 	DEARIIFTNPDMLHITILPQEERWRTFLKNLRYVVV~DELHYYNGLMGSHVALIMRRLRR
NCU06365.1	DDARIIFTNPDMLHITILPQEERWRSFLQNLKYVVV1DELHYYNGLMGSHVAFIMRRLRR
FG01945.1 	EQARVIFTNPDMLHITILPQEEQWRSFLKNLKYVVV1DELHYYNGQMGSHMSFIMRRLRR
AN5925.1  	DEARIIFTNPDMLHITILPQESSWRTFLQNLKFVVV~DELHVYNGLFGSHVALIMRRLRR
          	::**:****************. **:**:**::*** **** *** :***:::*******

MG06067.1 	ICAAVGNRHVKFVSCSATVANPREHFKTIFGISDVRLVDFDGSPSGRKEFLCWNTPYRVP
NCU06365.1	ILAALGNTHVLFISCSATVANPKEHFTTIFGVEDVKLIDFNGSPSGRKEFLCWNTPYKDP
FG01945.1 	ICAAVGNRRVKFISCSATVANPGQHFRTIFGIENVQLIDYDGSPSGRKEFLCWNTPYKDP
AN5925.1  	ICAAVGNRHVRFISCSATVANPEEHMRAIFGVDDVQLIDFDGSPCGRKEFLCWNTPFKDP
          	* **:** :* *:********* :*: :***:.:*:*:*::***.***********:: *

MG06067.1 	GDPSSGRGDAKYECARLFCQLVLRGVRVIAFCRVREQCEKLVGAVKAELERLGRPECIAR
NCU06365.1	GDPSSGRGNAKLECSRLFCELILRGVRVIAFCRVREHCEKLVNAIKQELEHRGRSECMSR
FG01945.1 	GDPASGRGSTKFECARLFCALMLRGVRIIAFCRVRAQCELLVTTIRQELENLGRPECTNL
AN5925.1  	GDPTSGRGDSVAEAARLFCQLILRGARVIAFCRIRKLCEVLLQAVRSECNRLERPEVGNM
          	***:****.:  *.:**** *:***.*:*****:*  ** *: ::: * :.  *.*    

MG06067.1 	VMGYRGGYTPQDRRKIEAEMFEGKLVGIVATTALELGVDIGSLDCVLTWGFPYTIANLRQ
NCU06365.1	VMGYRGGYTAQDRRRIETEMFEGKLMGIVATTALEIGIDIGNLDCVITWGFPYTIANLRQ
FG01945.1 	VMGYRGGYTAQDRRRIETEMFQGQLLGIVATTALELGIDIGSLDCVMTWGFPYTIANLRQ
AN5925.1  	IMGYRGGYSPQDRRRIEAEMFQGQLLGIVATNALELGVDIGSLDAVITLGFPYSISNLRQ
          	:*******:.****:**:***:*:*:*****.***:*:***.**.*:* ****:*:****

MG06067.1 	QSGRAGRRNLDSLSILVGDSFPTDQHYMQNPDEIFTQPNCALQVDLENMLVREGHIQCAA
NCU06365.1	QSGRAGRRNRDSLSILLGDSFATDQYYMQNPDELFTKPNCELSIDMDNMLVKEGHIQCAA
FG01945.1 	QSGRAGRRNKDSLSILVGDGFATDQHYMQNPDDLFTKPNCELQVDLENMLVREGHIQCAA
AN5925.1  	QSGRAGRRNKDSLSILIGERYPTDQFYMRNPEELFSKPNCELQVDLTNELVLEGHVQCAA
          	********* ******:*: :.***.**:**:::*::*** *.:*: * ** ***:****

MG06067.1 	YEMPIRPDEDAQYFGADIHKICIERLVPDELGYYHCHDRFRPMPSKFVTIRDTEDEHFAI
NCU06365.1	HEMPILPAKDSKYFGDDLATVCEERLLKGDRGFYHCHDKFRPRPSQFVSIRDIEEDHFAI
FG01945.1 	YEMPIRPREDAKYFGKDLPKICVERLIRDDMGFFHCHDRFRPIPAKYVAIRDTEDDHFAI
AN5925.1  	FELPIKPDDDQIYFGPQLSEFASTRLVRDAMGFYHCHERFRPQPSRCVPIRDTEDQHFAV
          	.*:** * .*  *** ::  ..  **: .  *::***::*** *:: *.*** *::***:

MG06067.1 	IDISYGRNVVLEELEASRATFTLYDGAIFLHQGTTYLVRDFDPEKHMARVEKVKVDWTTE
NCU06365.1	VDVTNNRNVVLEELEASRATFTIYDGAIFLHQGNTYLVRDFNPDNKMARVEKVKVDWTTQ
FG01945.1 	VDITNGRNIVLEELEASRATFTLYDGAIFLHQGNPYLVRDFQPDKGMARVERVKVEWTTV
AN5925.1  	IDTTNARNVVLEEVEASRAFFTLYEGGIFLHQGQTYLVKELNPDRFFARVVCVTVDWNTM
          	:* :  **:****:***** **:*:*.****** .***::::*:. :***  *.*:*.* 

MG06067.1 	QRDFTDIDPIETEAIKRIEGSP-----------------------CRAYHGTIRIKQVVF
NCU06365.1	QRDYTDIDPVETEAIRRIKGSK-----------------------SLAYHGVIRITQVVF
FG01945.1 	QRDYTDIDPTETEAIRTISDSR-----------------------SHAYYGTIKIQQNVF
AN5925.1  	QRDFTDIDPVETEHMRLITSSSPTKATKITDSSSTRSLEREKERAIRAFFGPIRIHAIVY
          	***:***** *** :: * .* .:.::. :.:::: : . ... :  *:.* *:*   *:

MG06067.1 	GYFKVDKRNRVLDAVHVDNPPVVRFSKGTWLDIPRRALEILDSRRLNAAAAIHAAEHCVM
NCU06365.1	GYFKVDKKNRILDAIQVDNPPVIRYSKGTWLDVPKSSLAILTSRRLHVGGAIHAAQHAIM
FG01945.1 	GFWKVDKKNRVLDAVQVDNPPVIRFSKGMWLDVPKTAMSILQERRLHIAAAIHAAEHAIM
AN5925.1  	GFFKIDKRGRVLDAVAVDNPPITIMTKGMWLDVPKVALDILESRRLNIAAAIHAAEHAIL
          	*::*:**:.*:***: *****:   :** ***:*: :: ** .***: ..*****:*.::

MG06067.1 	SLVPNFVISMPGDVRTECKNSLKEFAR---------------------KETSRKRPARLT
NCU06365.1	SLMPNFVMSMPGDVRTECKNHLKEFAK---------------------RETQRKRPARLT
FG01945.1 	SLLPAFVISMPGDVRTECKTAVKEFAK---------------------QESQRKRPARLT
AN5925.1  	SLLPSFVISSPGDVRTECKVAKKELGKDLQKVVRRGGGVGDQDNIPVLKPPHRQRPARLT
          	**:* **:* *********   **:.:. ..    ... ..... .  : . *:******

MG06067.1 	FYDAKGGAGGSGINIKAFEHVDMLLQQAVARVAGCGCMG--GCLECVASETCKQHNAVMS
NCU06365.1	FYDAKGGAGGSGINIKAFEFIDTLLRKALGRVLACQCRE--GCIECVCSELCKEANSVIS
FG01945.1 	FYDAKGGAGGSGISTKAFDHVDQLLRDALKRVENCRCER--GCVECVASEQCKQANEVMS
AN5925.1  	FYDAKGGSCGSGIARKAFEFIDSLLKRAVARIEACACVTPKGCLECVCDERCKEMNSVMS
          	*******: ****  ***:.:* **: *: *:  * *  ..**:***..* **: * *:*

MG06067.1 	KAGAEVVLKSLLGLEIDVDRLPMGPEDN--------SPAGVETIVLAKPVPPKDVRLWKD
NCU06365.1	KAGAEVILKSLLDEEIDVESLPMGPEEI--------SPAGVETVILAKPVPPRNRKTVKI
FG01945.1 	KAGSQVILKTLLNVEVDMDSLPMGPETN--------IPMGTETVVLAQPVPYRAKEAVFE
AN5925.1  	KAGAGVVLRCLLGWEVDVEALPWGEIDQDDGGEMGELAGGLETVVLAREVPYRGSE----
          	***: *:*: **. *:*:: ** *    ..... .. . * **::**: ** :  .    

MG06067.1 	YGSLS-DDEDEAHEGGIVDGELGPTSHGDI---------
NCU06365.1	IGLAG-EEIEIQVNDEQDDGEEEVDGS------------
FG01945.1 	NGINNRATVFEESEEGAKPGDSGTTDGGGGFEQWLADSI
AN5925.1  	---------------------------------------
          	
```
