# Supplementary material for: Patterns of Intron Gain and Loss in Fungi
Source: PLoS Biol. 2004 Nov 30;2(12):e422. doi: 10.1371/journal.pbio.0020422 (PMC532390; doi:10.1371/journal.pbio.0020422)
Supplement: Table S1 — Also available at http://genes.mit.edu/NielsenEtAl/. (4.3 MB ZIP). [file pbio.0020422.st001.zip › NielsenEtAl/html/1173.html]

AN5199.1.NCU06923.1.MG08139.1.FG01397.1


```
 CLUSTAL W (1.82) Multiple Sequence Alignments - Introns Inserted


Sequence 1: NCU06923.1	377 aa
Sequence 2: MG08139.1	406 aa
Sequence 3: FG01397.1	400 aa
Sequence 4: AN5199.1	402 aa
Alignment Length: 416 aa
Number Identitical Residues: 219 aa
Alignment Score (without introns) 11228


MG08139.1 	MTAEPPPKRKCLGADCENDAGSLQCPTCLKLGIKDSYFCSQDCFKKNW~VSITSILRLAL
NCU06923.1	MTEQPPAKKKCLGADCENDAGSLQCPKCLSLGIKGSFFCSQDCFKKNW0NTHKELHKLAS
FG01397.1 	MATDAPAK-QCMGADCSNDAGSLQCPTCLKLGIKDSFFCSQECFKRNW0GIHKTMHKSQS
AN5199.1  	MAAEVASR-KCLGTDCGKDAGSLQCPTCLKMGL-DSFFCSQDCFKRSW0SDHKALHKKSN
          	*: : ..: :*:*:** :********.**.:*: .*:****:***:.*    . : :   

MG08139.1 	~PVAPDRIPCHNHTSD2HLFPPKVVSKPDPETGLFNPFPAFNFSGPLRPVYPLSERRKVP
NCU06923.1	1---------------~------------PET--YNPFPTFSYTGPLRPVYPLSPKRVVP
FG01397.1 	~-----------NILH~HLKAPKAIS-PDPATGYYNPFPNFPYSGSLRPVYPLSPHRTLP
AN5199.1  	~------------FLT~NLFPPKVVSEPDPATGLFNPFPSFGFTGSLRPVYPLSPMRTVP
          	                    ...  :...* *. :**** * ::*.********  * :*

MG08139.1 	ASIPHPDYAGDGIPKHGRSLVRSNKIECLDAKAQEGMRKVCRLAREVLDIAAAAIHPGIT
NCU06923.1	KSIPHPDYAESGIPGGGR--TRSNKIEQLDEKGREGMRKVCRLAREVLDIAAAAIRPGIT
FG01397.1 	QSIPHPVWWQDGNPRYSRSLTNRNKIEILDKAGQDAMRKSCRLAREVLDIAAAAAKPGVT
AN5199.1  	KSIPHPDYAKDGIPRSEQKFVGRHNITILNKAEQEGMRKVCRLAREVLDIAARELRPGVT
          	 ***** :  .* *   :. .  ::*  *:   ::.*** ************   :**:*

MG08139.1 	TDEIDKIVHDACIERN0SYPSPLNYNHFPKSVCTSLNEVICHGIPDKRPLVDGDILNLDV
NCU06923.1	TDDLDKIVHEACIERN~SYPSPLNYNFFPKSVCTSLNEVICHGIPDKRVLLDGDILNLDV
FG01397.1 	TDYIDELVHKACIERN0SYPSPLNYNNFPKSCCTSVNEVICHGIPDQRVLLDGDILNIDV
AN5199.1  	TDYIDEVVHKACIERN0SYPSPLNYVHFPKSVCTSINETICHGIPDQRPLEDGDIINIDV
          	** :*::**.****** ********  **** ***:**.*******:* * ****:*:**

MG08139.1 	TLYHEGYHGDLNETYYVGDKALADPDVVRLVETTRECLDEAIKLVKPGTLFREFGNVIEK
NCU06923.1	TLYHEGYHGDLNETYYVGDKAKADADSVRVVETARECLEEAIKLVKPGTLFRDFGNVIEA
FG01397.1 	SLYHEGYHADLNETYYIGDKAKADPDTVRVVETARQCLDESIKAVKPGTLIREFGNIIEK
AN5199.1  	TLYHEGFHGDINETYYVGEKARSNPDAVRVVETARECLDKSIEIVKPGMLFRDPGNVIEK
          	:*****:*.*:*****:*:** ::.* **:***:*:**:::*: **** *:*: **:** 

MG08139.1 	HAKANNCSVIRTYVGHGINSVFHCPPNIPHYAKNKAVGECKPGMTFTIEPMLALGKYRDI
NCU06923.1	HAKSRGCSVIRTYVGHGINKTFHCPPNIPHYAKNRAVGECKPGMTFTIEPMIALGKYRDV
FG01397.1 	HAKQHNCSVIRTYCGHGVGKLFHCPPNVPHYAKNKTVGECKPGMTFTIEPMIALGKYRDI
AN5199.1  	HAKSRNCSVVKSYCGHGINQLFHCAPNVPHYAKNKAVGTAKPGMCFTIEPMINIGTHRDR
          	*** ..***:::* ***:.. ***.**:******::** .**** ******: :*.:** 

MG08139.1 	TWPDNWTSTTIDGKRSAQFE1HTLLVTETGVEVLTARKADSPGGPVPLPTA---------
NCU06923.1	TWPDNWTSTTIDGKRTAQFE1HTLLVTETGVEILTARTADSPGGPVPMPTAAA-------
FG01397.1 	TWPDNWTSTTIDGKMTAQFE1HTLLVTEDGVEILTARQENSPGGALPMPGTENGDAKA--
AN5199.1  	LWPDDWTSTTADGSLSAQFE~HTLLVTEDGVEVLTARLPDSPGGPIPMPGTEAAGEVKTD
          	 ***:***** **. :**** ******* ***:****  :****.:*:* :  ..   :.

MG08139.1 	-
NCU06923.1	-
FG01397.1 	-
AN5199.1  	A
          	:
```
