# Supplementary material for: Patterns of Intron Gain and Loss in Fungi
Source: PLoS Biol. 2004 Nov 30;2(12):e422. doi: 10.1371/journal.pbio.0020422 (PMC532390; doi:10.1371/journal.pbio.0020422)
Supplement: Table S1 — Also available at http://genes.mit.edu/NielsenEtAl/. (4.3 MB ZIP). [file pbio.0020422.st001.zip › NielsenEtAl/html/1174.html]

AN4189.1.NCU06419.1.MG06482.1.FG07295.1


```
 CLUSTAL W (1.82) Multiple Sequence Alignments - Introns Inserted


Sequence 1: MG06482.1	515 aa
Sequence 2: FG07295.1	524 aa
Sequence 3: NCU06419.1	587 aa
Sequence 4: AN4189.1	502 aa
Alignment Length: 647 aa
Number Identitical Residues: 278 aa
Alignment Score (without introns) 13073


MG06482.1 	MKNPISSLDVPTP--~------PATTIPTLSS---PAPLLRPAIPGAR--SAGARTPRLG
NCU06419.1	--------MAPAP--~--------------------APLLRPAIPGAR--PGGRGPPRLG
FG07295.1 	MADQQPQGNDPTPQP0QQSAAPTPISLPTHNSGPSPVPLLRPAIPGAR--NGGARTPRLG
AN4189.1  	--------MSSSP--~--------------------VPLLKAPVPGNRGSSNSPRPPKLT
          	          .:*                       .***:..:** *.:  .  .*:* 

MG06482.1 	LAIPPSPNVKPVG--------------GAPGRPPLPTLHLATPMGSSVTPHEQPPGRPSI
NCU06419.1	LAIPPSLSVKPVGN-----------PGAPPARAAPPQLKLATPMGSTTIPHEQPAGRPGA
FG07295.1 	LAIPPSPNAKPVGNQ----------PIAAPSRPPLPTLHLATPMGSQVAPQEQLP-RSQC
AN4189.1  	LGIPPSATSRPVAGNGVPIMSDAPQPQRPSGRPAPPQLRLATPMGSTQDVPPQRNVRPMP
          	*.**** . :**.... .  :.:...  ...*.. * *:*******      *   *.  

MG06482.1 	VTQQGQSASGGSESSAAHSRSGSFGPLDGRTSNPTSAG-SQYSALSFASHFGIGSTRPQG
NCU06419.1	QGY---SASGASDSSAAHSRSGSFGP----ESNPTSAD-TRYSNVSF-----IGAQRPHG
FG07295.1 	VTQ--ASAGGGSESSAAHSRSGSFGPLDGRASNPTSAG-SQYSALSFASQYGIGVSRPQG
AN4189.1  	PPL----ATTGLNDANGHSRSGSSTHLDGKGSGPASASSSNYSNLSFAMG---GLRQPHG
          	       *  . :.: .******    ..  *.*:**.::.** :**:     *  :*:*

MG06482.1 	-TPDPASAVGSIYSERSDGGAGMDKDGNLKG-LENFDKLTIDKARTADVEDLDVEGWKIA
NCU06419.1	-TPDPVSAVGSLYSNASEGGVGMERENSLHG-LEAFDKLTLEKARTLDVEELDDDGWRIA
FG07295.1 	-TPDPVSAVGSMYSERSEGGVSMERDGSLQG-LEAFDKLTIEKARTLDVDDLDEEGWRIA
AN4189.1  	GTPDPSSAISSVYSDR-EGGVQMERDNSVNGLIPDLDKLSLEKGRALDVEDLDDQGWLAA
          	.**** **:.*:**:  :**. *:::..::* :  :***:::*.*: **::** :**  *

MG06482.1 	SMEKRIVELGGLGEGAGGAVTRCKLTGGKTVFALK0VITANPDPDVKKQIMRELDFNIQC
NCU06419.1	MMEKRVEELGPLGEGAGGAVTKAKLKGGKTVFALK0IITANPDKDVAKQIVRELGFNKQC
FG07295.1 	SLEKRIVEIGNLGEGAGGAVTRCKLKGGNTVFALK0VITTNPDPDVKKQILRELGFNKEC
AN4189.1  	SEQKKIVELGSLGEGAGGAVTRCKLKEGKTVFALK0IITTDPNPDVKKQIIRELNFNKDC
          	  :*:: *:* **********:.**. *:****** :**::*: ** ***:***.** :*

MG06482.1 	ASEHICRYYGAFEDPSTATISIAMEFCEGGSLDSIYKEVKRLGGRTGEKVLGKIAEGVLR
NCU06419.1	ASEHICRYFGAVVDTQSATISIAMEYCEGGSLDSVYKEVKKLGGRTGERVLGKIAEGVLH
FG07295.1 	ASDHICKYYGAFVDPSTATISIAMEFCEGGSLDSIYKEVKRLGGRTGEKVLGKIAEGVLG
AN4189.1  	ASEHICRYYGAFMDKSTGTISIAMEFCEGGSLDSIYKEVKKLGGRTGEKVLGKVAEGVLN
          	**:***:*:**. * .:.*******:********:*****:*******:****:***** 

MG06482.1 	GLTYLNSKKIIHRD~IKPSNILLCRNGDVKLCDFGVSGDFGTKGEANTFIGTSYYMAPER
NCU06419.1	GLTYLHSKKIIHRD~IKPSNILLCRNGEVKLCDFGVSGDYGTNGAANTFIGTSYYMAPER
FG07295.1 	GLTYLHTRRIIHRD~IKPSNILLCRDGAVKLCDFGVSGDFGTKGEANTFIGTSYYMAPER
AN4189.1  	GLTYLHGRKIIHRD1IKPSNILLCRNGQVKLCDFGVSGEFGTKGDANTFIGTSYYMAPER
          	*****: ::***** **********:* **********::**:* ***************

MG06482.1 	ITGQSYTITSDVWSTGVTLLEVAQHRFPFPADGTEMAPRAGLIDLLTYIVRQPIPKLKDE
NCU06419.1	ITGQSYTITSDVWSLGVTLLEVAQHRFPFPADGTDSQPRAGLIDLLTYIVRQPVPKLKDE
FG07295.1 	ITGQSYTITSDVWSTGVTLLEVAQHRFPFPADGTEMQPRANLIDLLTYIVRQDVPKLKDE
AN4189.1  	ITGQSYTITSDVWSLGVTLLEVAQHRFPFPADGTEMQPRAGLIDLLTYIVRQPIPKLKDE
          	************** *******************:  ***.*********** :******

MG06482.1 	PSAQISWSENFKYFIECC2--LEKDPQRRASPWRMLEHPWMVDMKSKRVNMTRYLAQ~VW
NCU06419.1	PDANIFWTDKFKYFIDCC2--LEKDPNRRASPWRMLDHPWMLEIRSRRVNVARFLAT0TW
FG07295.1 	PDMDVYWSNNFKYFIECC2--LEKQPNRRASPWKMMEHPWMVEMRSKRVNMVKYLSY~VW
AN4189.1  	PENGIKWSSNFKYFIECC2NSLEKEPPRRATPWRMLEHPWVLDMKNKKVNMANFVKQ0SF
          	*.  : *:.:*****:** .:***:* ***:**:*::***:::::.::**:..::    :

MG06482.1 	G-------------------------------WDDKGEAKPAE-----------------
NCU06419.1	RNHRTVSITQLVPAPVGEDPFGRRHLNPGRVIEEDADKDEQYEIDKILGRRTNFKHGRPI
FG07295.1 	G-------------------------------WGDQPKDS--------------------
AN4189.1  	D-------------------------------------STVL------------------
          	                                                            

MG06482.1 	----------------------------------------------------~
NCU06419.1	LEYLIKWKGYRNQWNSYVLRPSLLQNASDLVYDYDTKNPVDDKEPKPKKPRK0
FG07295.1 	----------------------------------------------------~
AN4189.1  	----------------------------------------------------~
          	
```
