# Supplementary material for: Patterns of Intron Gain and Loss in Fungi
Source: PLoS Biol. 2004 Nov 30;2(12):e422. doi: 10.1371/journal.pbio.0020422 (PMC532390; doi:10.1371/journal.pbio.0020422)
Supplement: Table S1 — Also available at http://genes.mit.edu/NielsenEtAl/. (4.3 MB ZIP). [file pbio.0020422.st001.zip › NielsenEtAl/html/1175.html]

AN4342.1.NCU10034.1.MG07181.1.FG04395.1


```
 CLUSTAL W (1.82) Multiple Sequence Alignments - Introns Inserted


Sequence 1: NCU10034.1	214 aa
Sequence 2: MG07181.1	215 aa
Sequence 3: FG04395.1	207 aa
Sequence 4: AN4342.1	208 aa
Alignment Length: 226 aa
Number Identitical Residues: 67 aa
Alignment Score (without introns) 4068


MG07181.1 	MPSLLILIFTIEVAVELINTIGAATINNL0LWRIFNALPTKLSAQFAEQRKLQQDYLKVR
NCU10034.1	MPSLLVVIFVIELFVQLVNTIGAATINNL0LWRIALSLPLPLSAQFAAQRKKQKEYLAIR
FG04395.1 	MASLMLSVFVVEVIVNLVNTIGATAINNL0LWTLINFLPISTSKAAGEQRKLQADYLKVR
AN4342.1  	MISLIWTIFILHIAIFLVNTIGAATIDNL0LWLLYLKLPTSLYQTAQEQTKLKREVVQLK
          	* **:  :* :.: : *:*****::*:** ** :   **         * * : : : ::

MG07181.1 	RELNATSSQDEFAKWAKLRRQHDKLLEQLEKK-----------K1AALDSTKGNFDKYIT
NCU10034.1	RELNATSSQDEFAKWARLRRQHDKLLEDLEKR-----------K1KELDAAKTKFDRTLT
FG04395.1 	RDLNATSSQDEFARWAKLRRQHDKLLEQLEKT-----------K1KTTEASRSNFDRVLT
AN4342.1  	RDMNNTSSQDEFAKWAKLRRRHDKALSEYEALSMLPLSFSSLFY1QKLSSQKGSFDWFVK
          	*::* ********:**:***:*** *.: *  :  . : ::       .: : .**  :.

MG07181.1 	GIRWVGTQGLRYFLPFWYAKVPMFWLPYGWFPYYAEWLVSFPRAPMGSVSIASWQLACTG
NCU10034.1	TVRVVATRGLQWFLPFWYSREPMFWLPYGWFPYYVEWFASFPRAPLGSVSIVVWQWACTG
FG04395.1 	VVRIVVTRAPQYFLPFWYATEPMFWLPHGWFPYWAEWILSFPRAPIGSVSIASWQLACTG
AN4342.1  	IARWLSTTGLKIFIQFRYSKTPVFELPGGWLPYPVEWVLAFPRAPQGSVSVQVWNSVCAT
          	  * : * . : *: * *:  *:* ** **:** .**. :***** ****:  *: .*: 

MG07181.1 	FVVLIKDAITALVVFVMGMRQSNVKQAVPVKAVSGEKASDEKEGKKEL
NCU10034.1	VIKLVIETVMAVVGLIVAARQKQQEKQKAKQAVPAAGGGDSKAEEAK-
FG04395.1 	VIALLSDLIAGTAGLLFGTKEAKEAPITSEKVVAEEK---KKS-----
AN4342.1  	AVTVIAEIITGLALQVKGS--AQAVPATAKKA----------------
          	 : :: : : . .  : .    :     . :.
```
