# Supplementary material for: Patterns of Intron Gain and Loss in Fungi
Source: PLoS Biol. 2004 Nov 30;2(12):e422. doi: 10.1371/journal.pbio.0020422 (PMC532390; doi:10.1371/journal.pbio.0020422)
Supplement: Table S1 — Also available at http://genes.mit.edu/NielsenEtAl/. (4.3 MB ZIP). [file pbio.0020422.st001.zip › NielsenEtAl/html/1176.html]

AN7029.1.NCU04481.1.MG10169.1.FG09624.1


```
 CLUSTAL W (1.82) Multiple Sequence Alignments - Introns Inserted


Sequence 1: NCU04481.1	555 aa
Sequence 2: MG10169.1	721 aa
Sequence 3: FG09624.1	616 aa
Sequence 4: AN7029.1	653 aa
Alignment Length: 824 aa
Number Identitical Residues: 240 aa
Alignment Score (without introns) 11283


MG10169.1 	MLSLKTLAVTSALALQAAAVNIRIDVGKNGLVFSPNSTNAAIGDVLEFHFYARNHSVVQG
NCU04481.1	---------------------------------------------------MRVTSVPLG
FG09624.1 	-----------------------------------------------MRGALRCSTVIRH
AN7029.1  	---------------------------------------------MSGPSARLCAHHIAG
          	                                              .             

MG10169.1 	RFDQP0CAPFPNNPFYSGFIPASEGNEN0ATVFQVTVASTAPAVYYCSQTQHCARGMYAV
NCU04481.1	-----~-LPTTAKPLFR-----------~------------------SSTARSAR---II
FG09624.1 	-----~--ARVVGPLTD-----------~--------------------SGRYAR-----
AN7029.1  	---AP~SRPALIR---------------~------------------PVAVRTSKP----
          	    . . .                                      . : : ::     

MG10169.1 	INPSGQNTLQQYGSRVTANITAIDPPAGIRVPNGGVYSIQSSQQTSTASSATNTPTGATT
NCU04481.1	TNTS------------TPRRTQCTSACGHRNVN------ESRALAHAARPAARIIFPATF
FG09624.1 	----------------TFRTTAVSHALRI------------GSCCRKIRPELAIQGANIS
AN7029.1  	----------------VFVAGCRRNFHGLR-----------TAPSRKHNGYGWCNPGFVN
          	                .                                           

MG10169.1 	TAGAGRPTMTNNPTSTKSGFAVPTQ~APVAGVVGVALAALI2RFAAHVMVSAWVPGTPLQ
NCU04481.1	TAGQRR-----RATTATAAAAVHNA1NPEN------LGPIQ~EYDRRVAN----------
FG09624.1 	TSCPNR---------SRSMATVVDA~EPIHG-----GGPIP~EYDRRVAA----------
AN7029.1  	HATHQR---------RSAATAAEVA~TPVKASAAEAAGPLA~EYNARVEQ----------
          	 :   *           :  :.     *  .  . : ..:  .:  :*            

MG10169.1 	LPLLRTCSSAKLRHSSSNSVMNLLSVAFKSQT1NKDATS~PMQEYDRRVDEGILRNDEHQ
NCU04481.1	---------GELRN------------------~DDHQRG1IIQNLQHLHEE--LRNYAAP
FG09624.1 	---------GRLRN------------------~DEHQRG1IIQNFQNLYHE--LERYDAP
AN7029.1  	---------GRLRD------------------~DPYQRQ~IIEQLQDLYER--LKSYKAP
          	         ..**.                   :       ::: :   ..  *.     

MG10169.1 	RV1KHPSLEALKAPAQKSLFGSFFGSKGPKKAAIGDIPANLPRGLYLYGDVGSGKTMLMD
NCU04481.1	PV~VHPTLESLKP--QKSLF-SFFGGKS--KSAIAEIPANLPRGLYLYGDVGCGKTMLMD
FG09624.1 	PV~EHPTIESLKP-TKKSIFSSLFGSSG-KKSAIGTISSDLPKGLYLHGDVGCGKTMLMD
AN7029.1  	AV~VRPSIESLDAAPKKSFFGSLFGKAP--AKPESSIPEDLPKGLYMYGDVGCGKTMLMD
          	 *  :*::*:*....:**:*.*:**       . . *. :**:***::****.*******

MG10169.1 	LFYDTLPPSVKSKTRIHFHNFMQDVHKRMHKMKMQHGNDLDAVPLIAADIAEQGNVLCFD
NCU04481.1	LFYDTLPHNIKSKTRIHFHNFMQDVHKRLHKIKMQYGNDVDAVPFVAADIAQQGSVLCFD
FG09624.1 	LLYDTLPPSVKSKSRIHFNNFMQDVHKRLHKFKMEHGNDIDGVPYVAADIAQQGNVLCFD
AN7029.1  	LFYETLPSNIRSKSRIHFHNFMQDVHRRMHVVKMKFGNDFDALPLVAADIAEKSSVLCFD
          	*:*:*** .::**:****:*******:*:* .**:.***.*.:* :*****::..*****

MG10169.1 	EFQCTDVADAMILRR~LLEALMSHGVVLVTTSNRHPDDLYKNGVQRESFIPAIELLKSRL
NCU04481.1	EFQCTDVADAMILRR~LLEALMSHGVVLVTTSNRHPDELYINGVQRESFIPAIELLKNRL
FG09624.1 	EFQCTDVADAMILRR2LLESLMANGVVLVTTSNRKPDELYKNGVQRESFIPAIELLKNRL
AN7029.1  	EFQCTDVADAMILRR2LLESLMSHGVVLVTTSNRHPDDLYLNGIQRQSFIPCITLLKTVL
          	*************** ***:**::**********:**:** **:**:****.* ***. *

MG10169.1 	HVINLDSPTDYRKIPRPPSGVYHTPLDKHAQSHAEKWFAFLGDASDPG-HPETQTVWGRK
NCU04481.1	HVINLNSNTDYRKIPRPPSGVYHTALDAHAASHAEKWFRFLGDPENPEPHPEVQTVWGRE
FG09624.1 	HVINLDSPTDYRKIPRPPSDVYHTSLDTHAQAHAEKWFRFLGDPEQPEPRPEIQKVWGRE
AN7029.1  	TVINLNSPTDYRKIPRPPSGVYHHPLGPEADQHAQKWFDYLGDPINDPPHPTTQEVWGRK
          	 ****:* ***********.*** .*. .*  **:*** :***. :  .:*  * ****:

MG10169.1 	IHVPRVSGRCACFTFDELIGRPTGAADYIELVRSYDAFVVTDVPGMTYRQRDLARRFITF
NCU04481.1	IHVPRVSGRCAWFTFDELIGQPTGAADYIELMRSYDAFIVTDIPGMTYRQRDLARRFITF
FG09624.1 	IYVPRVSGRAAWFTFDELIRQPKSAADFIELVRSYEAFIVTDIPGMTHQQRDLARRFITF
AN7029.1  	IQVPLASGKAAKFSFQQLIGAATGAADYLELVRNYEAFIVTDVPGMTLHQRDLARRFITF
          	* ** .**:.* *:*::**  ...***::**:*.*:**:***:**** :***********

MG10169.1 	IDAVYESH~AKLVLTTAAPLGELFVSREEMRESLAATRKKDAGREEPDDGDVEGAMGHMM
NCU04481.1	IDAVYESH~AKLVLTAAVPLTELFVSRQEIEESLKKQ-----GKALDQTHSVADVMSHMM
FG09624.1 	IDVVYEGN~AKLVLTTEKPLSELFVSRDEIAESLMKQ-----GVKGADAEKAADSH----
AN7029.1  	IDAVYESR0AKLVLTTATPLTNLFISESEVKTSLDDN---------GEHTDLSDAMRMMM
          	**.***.. ******:  ** :**:*..*:  **             :  .  .      

MG10169.1 	EDLDSNVDKLRNSNLFSGDEEAFAFARALSRLSHMGSKEWVERG----MGLESAGGKKEK
NCU04481.1	DDLDQNADKLSKSNLFSGDEEAFAFARALSRLTEMGSKMWVERG----MGLEDKGGKPEH
FG09624.1 	-DLVHNVDKLKDSNLFAGTEEAFAFARALSRLRHMESKEWVERG----MGLEAQGGQPDK
AN7029.1  	DDLGLSMKALKTSSLFSGDEERFAFARALSRLSEMGSKEWVERGLLGKSGLDAEGKEKEK
          	.**  . . *  *.**:* ** ********** .* ** *****  .. **:  * : ::

MG10169.1 	---DDWAKVRS~KQMEDSM~--------------~-----~-------------------
NCU04481.1	---DSWTKTRS~RQMEDSM~--------------~-----~-------------------
FG09624.1 	---DNWVKTRT1LSAHQAR~E----LLGMHDTGL0LAYGY~RILLFRKVKPSYDDEHSEL
AN7029.1  	NEHEAYLKARS~RWSEDNI2FNPRLHIAICPVTF~QFYAS1LWLSISGLAPDEADTCSFF
          	.. : : *.*:    .:    ..    .          .           ..  .  :  

MG10169.1 	-------------------------~--------------------------------
NCU04481.1	-------------------------~--------------------------------
FG09624.1 	IRNVFKRPLGSEVSTKMNCRWIVQF0VMRILGISKQRRDESRGI--------------
AN7029.1  	ILEQGQNPAALLGASDQEYWWLYKG~LTGIMHLEISKLSFRYQLKFSVGQFPLVTASL
          	  .  . . .   ::. .     .         . .  .     . : .. .  :::
```
