# Supplementary material for: Patterns of Intron Gain and Loss in Fungi
Source: PLoS Biol. 2004 Nov 30;2(12):e422. doi: 10.1371/journal.pbio.0020422 (PMC532390; doi:10.1371/journal.pbio.0020422)
Supplement: Table S1 — Also available at http://genes.mit.edu/NielsenEtAl/. (4.3 MB ZIP). [file pbio.0020422.st001.zip › NielsenEtAl/html/1177.html]

AN2435.1.NCU06783.1.MG06720.1.FG06039.1


```
 CLUSTAL W (1.82) Multiple Sequence Alignments - Introns Inserted


Sequence 1: NCU06783.1	481 aa
Sequence 2: FG06039.1	489 aa
Sequence 3: MG06720.1	486 aa
Sequence 4: AN2435.1	485 aa
Alignment Length: 490 aa
Number Identitical Residues: 353 aa
Alignment Score (without introns) 15237


MG06720.1 	MSAKSITEADGKAILNYHLTRAPVIKPSPLPAAATHNPPPRLASLYFAEDADVNGVLDQA
NCU06783.1	MSAKSILEADGKAILNYHLTRAPVIKPSTLPNPTKHNPPPRLASLHFAEDADVNGVLSQA
FG06039.1 	MSAKSILEADGKAILNYHLTRAPVIKASPLPAPTTHNPPSRLASLHFPEDANVADILNQA
AN2435.1  	MSAKSIFEADGKAILNYHLTRAPVIKPTPLPPSNTHNPPPKLASLYFPDDLSVKDVLDQA
          	****** *******************.:.** . .****.:****:*.:* .* .:*.**

MG06720.1 	EVTYPWLLQPGAKFVAKPDQLIKRRGKSGLLALNKTWAEAKAWVAERAGKEQKVEHVTGV
NCU06783.1	EVTYPWLLQEGARFVAKPDQLIKRRGKSGLLALNKTWAEAKAWIAERAGKPQKVEHTEGV
FG06039.1 	EVTYPWLLQPDAKFVAKPDQLIKRRGKSGLLALNKSWPEAKAWVAERAGKEQQVEHTTGV
AN2435.1  	EVTYPWLLTPGSKFVAKPDQLIKRRGKSGLLALNKTWAEAREWIEARATKEQQVETVVGV
          	********  .::**********************:*.**: *:  ** * *:** . **

MG06720.1 	LRNFLVEPFVPHPDGTEYYIN~INSVRD0GDWILFTHEGGVDVGDVDEKAEKLLIPVDLS
NCU06783.1	LRQFLVEPFVPHPQETEYYIN0------~GDWILFYHEGGVDVGDVDAKAEKILIPVDLS
FG06039.1 	LRQFLVEPFVPHPQDTEYYIN~INSVRD0GDWILFTHEGGVDVGDVDAKAEKLLIPVDLA
AN2435.1  	LRHFLVEPFVPHPQETEYYIN~IHSVRE0GDWILFTHEGGVDVGDVDAKAEKLLIPVNLK
          	**:**********: ******   :  . ****** *********** ****:****:* 

MG06720.1 	EYPSNEEIASALLKKVPKGIHNVLVDFI~TRLYAVYVDCQFTYLEINPLVVIPNEDATSA
NCU06783.1	QYPSNEELASTLLKHVPKGIHNVLVDFI1ARLYAVYVDCQFTYLEINPLVVIPNEDATSA
FG06039.1 	EYPSNEEIAATLLKKVPQGVHNVLVDFI~TRLYAVYVDCQFTYLEINPLVVIPNEDKTSA
AN2435.1  	NYPSNEEIASALLSKVPKGIHNVLVDFI~SRLYAVYVDCQFTYLEINPLVVIPNADATSA
          	:******:*::**.:**:*:******** :************************ * ***

MG06720.1 	AVHFLDLAAKLDQTADFECGVKWAIARSPAALGLA---ATASSKINIDAGPPMEFPAPFG
NCU06783.1	EVHFLDLAAKLDQTADFECGNKWAIARSPAALGIV--AQSSNTGVNIDAGPPIEFPAPFG
FG06039.1 	EVHFLDLAAKLDQTADFECGVKWAIARSPAALGLTNIAPSADGKISIDAGPPMEFPAPFG
AN2435.1  	DVHFLDLAAKLDQTAEFECGTKWAVARSPANLGLA--ALPTSDKVNIDAGPPMEFPAPFG
          	 **************:**** ***:***** **:.  : .:.  :.******:*******

MG06720.1 	RELTKEEAYIAELDAKTGASLKLTVLNGNGRVWTLVAGGGASVVYADAIASAGFADQLAN
NCU06783.1	RELSKEEAYIAELDAKTGASLKLTVLNPNGRIWTLVAGGGASVVYADAIASAGFADELAN
FG06039.1 	RELTKEEAYIADLDAKTGASLKLTVLNAKGRIWTLVAGGGASVVYADAIASAGFADELAN
AN2435.1  	RELSKEEKFISDMDAKTGASLKLTVLNPNGRVWTLVAGGGASVVYADAIASAGFVSELAN
          	***:*** :*:::************** :**:**********************..:***

MG06720.1 	YGEYSGAPTESQTYHYARTVLDLMLRAPLAPEGKVLFIGGGIANFTNVASTFKGVIKALR
NCU06783.1	YGEYSGAPTESQTYHYARTVLDLMLRAPVSEKGKVLFIGGGIANFTNVASTFKGVIKALR
FG06039.1 	YGEYSGAPTESQTYHYARTVLDLLLRAPKTEEGKVLFIGGGIANFTNVASTFKGVIRALR
AN2435.1  	YGEYSGAPTETQTFNYARTILDLMLRSPIHPDGKVLFIGGGIANFTNVASTFKGVIRALR
          	**********:**::****:***:**:*   .************************:***

MG06720.1 	EYAKALNEHNVSIWVRRAGPNYQEGLRNMKAATQELGLNAKIFGPEMHVSGIVPLALIPG
NCU06783.1	EYGKALIEHNTQIWVRRAGPNYQEGLKNLKAATQELGLNAKIFGPEMHVSGIVPLALVPG
FG06039.1 	DFAPKLIEHKTAIWVRRAGPNYQEGLKNMKAATQELGLDAKIFGPEMHVSGIVPLALVPG
AN2435.1  	EVAPVLNEHKVQIWVRRAGPNYQEGLKNIKAVGEELGLNMHVYGPEMHVSGIVPLALQG-
          	: .  * **:. **************:*:**. :****: :::**************   

MG06720.1 	KWEEGNFEEFKG-
NCU06783.1	KWEESGAVEFQA-
FG06039.1 	KWEESKAQEFQA-
AN2435.1  	--KQTDIKEFGTA
          	  ::    **  :
```
