# Supplementary material for: Patterns of Intron Gain and Loss in Fungi
Source: PLoS Biol. 2004 Nov 30;2(12):e422. doi: 10.1371/journal.pbio.0020422 (PMC532390; doi:10.1371/journal.pbio.0020422)
Supplement: Table S1 — Also available at http://genes.mit.edu/NielsenEtAl/. (4.3 MB ZIP). [file pbio.0020422.st001.zip › NielsenEtAl/html/1178.html]

AN4611.1.NCU00564.1.MG03019.1.FG00168.1


```
 CLUSTAL W (1.82) Multiple Sequence Alignments - Introns Inserted


Sequence 1: NCU00564.1	113 aa
Sequence 2: MG03019.1	113 aa
Sequence 3: FG00168.1	113 aa
Sequence 4: AN4611.1	113 aa
Alignment Length: 113 aa
Number Identitical Residues: 71 aa
Alignment Score (without introns) 3309


MG03019.1 	MSMFRAKKLDLGCIIRTRIVRDHTKRKTFEAFEPER2QALRYIIRNTTLPARTRAEAQLQ
NCU00564.1	MSMFRAKKLDLGCFTNVRVLRDHSKRKAFLEAEPER2QALRYVIRNTTLPARTRAVAQLQ
FG00168.1 	MSMFRAKKLDLGCFVNVRTLRDHTKRKVFEAHETER2QALRYIIRNTTLPPRVRAEAQLQ
AN4611.1  	MSQFRAKKLDIGGFINIRVIRDHTKRKVFEQYEPER2QALRYIIRNTTLPQRVRAQAQLQ
          	** *******:* : . * :***:***.*   *.** *****:******* *.** ****

MG03019.1 	LTQMHAYTRPTQIRNRCILGGKTRGIFRDFKMSR0YNFRLQALAGSIPGVKKASW
NCU00564.1	LTQMHAYTRPTQIRNRCILGGKSRGILRDFKMTR0YNFRMNALMGNIPGVKKASW
FG00168.1 	LTQMHCYTRPTQIRNRCIMGGQGRGILSDFKLSR0YNFRMEAMAGNLPGVKRASW
AN4611.1  	LSQMHAYTRSTQIKNRCVAGGTARSVFRDFRIGR0YQFRQQALAGELPGVKKASW
          	*:***.***.***:***: **  *.:: **:: * *:** :*: *.:****:***
```
